# Monoglyceride-structured rubber seed oil oleogels as a partial cocoa butter replacement in chocolate: physical properties, oxidative stability and applications

**DOI:** 10.1016/j.fochx.2026.104594

**Published:** 2026-10-11

**Authors:** Zhi Huang, Yaru Tang, Xinru Yang, Dongyan Liu, Yang Liu, Yangfan Ren, Yun Wang, Beibei Zhang, Ji Ma

**Affiliations:** a Faculty of Life Science and Technology, Kunming University of Science and Technology, Kunming 650500, China; b Natural Rubber Health Industry Technology Innovation Research Institute, Xishuangbanna 666100, China; c China National Research Center for Food Safety Inspection and Testing (China National Research Center for Food Safety HACCP Application), Beijing 100037, China

**Keywords:** Rubber seed oil, Oleogel, Monoglycerides, Physical properties, Thermal behavior, Chocolate

## Abstract

In this study, rubber seed oil (RSO) was structured into oleogels (RSOOs) using monoglycerides (MG) as a food-grade gelator. The RSO fatty-acid profile was determined by GC/Q-MS. The profile was dominated by C16-C18 species, principally linoleic acid (C18:2n-6, 40.39%), oleic acid (C18:1n-9, 22.86%) and alpha-linolenic acid (C18:3n-3, 21.72%), together with palmitic (8.23%) and stearic (6.07%) acids; this gave class totals of 14.65% saturated, 23.15% monounsaturated and 62.20% polyunsaturated fatty acids and n-6/n-3 = 1.863. The critical gelation concentration was 5.0 wt% MG at 25 °C. Across the 1–12 wt% MG series, RSOO hardness increased from 20.045 to 1127.846 g and oil-binding capacity (OBC) from 24.92% to 99.87%. FTIR showed no new or lost absorption bands on gelation; small C—H band shifts and an intensified O—H envelope were consistent with van der Waals and hydrogen-bonding interactions supporting a physical network. XRD showed β/β’ short spacings whose intensity increased with MG content while polymorph identity remained unchanged. RSOOs were solid-like and thermoreversible. Under accelerated storage (40 °C, dark, 21 days), the 12% MG-RSO accumulated secondary oxidation products (TBARS) less than pure RSO and DPPH radical-scavenging capacity higher than pure RSO, an effect attributed to physical immobilization of the oil and compositional dilution rather than demonstrated intrinsic antioxidant activity. A 7 wt% MG RSOO, selected on the hardness-OBC plateau and on economic grounds, was used to replace 0–100% cocoa butter (CB) in chocolate. The chocolate melt peak shifted from 21.93 °C to 17.84 °C. A practical replacement window of 20–60% retained acceptable thermal stability and sensory quality, with the 20% formulation receiving the highest flavor-balance score. Above 60%, chocolates failed structurally at 30 °C, and 100% replacement collapsed at 25 °C. The results support RSOO as a partial CB replacement within a defined, conservative window.

## Highlights

• Rubber seed oil contains relatively high levels of polyunsaturated fatty acids and the ratio of ω-6 to ω-3 unsaturated fatty acids in rubber seed oil is 1.8631.
• Monoglycerides forms rubber seed oil oleogels at 25 °C with a critical gelation concentration of 5.0 wt%.
• Monoglycerides concentration enhances rubber seed oil oleogels' hardness, oil-binding capacity and thermal stability via crystalline networks.
• Rubber seed oil oleogels show thermoreversibility, viscoelasticity and improved oxidative stability (test by TBARS way).
• 7 wt% monoglycerides - rubber seed oil oleogel is successfully applied in chocolate products, and has improved the silky taste of chocolate.

## Graphical abstract

**Figure:**
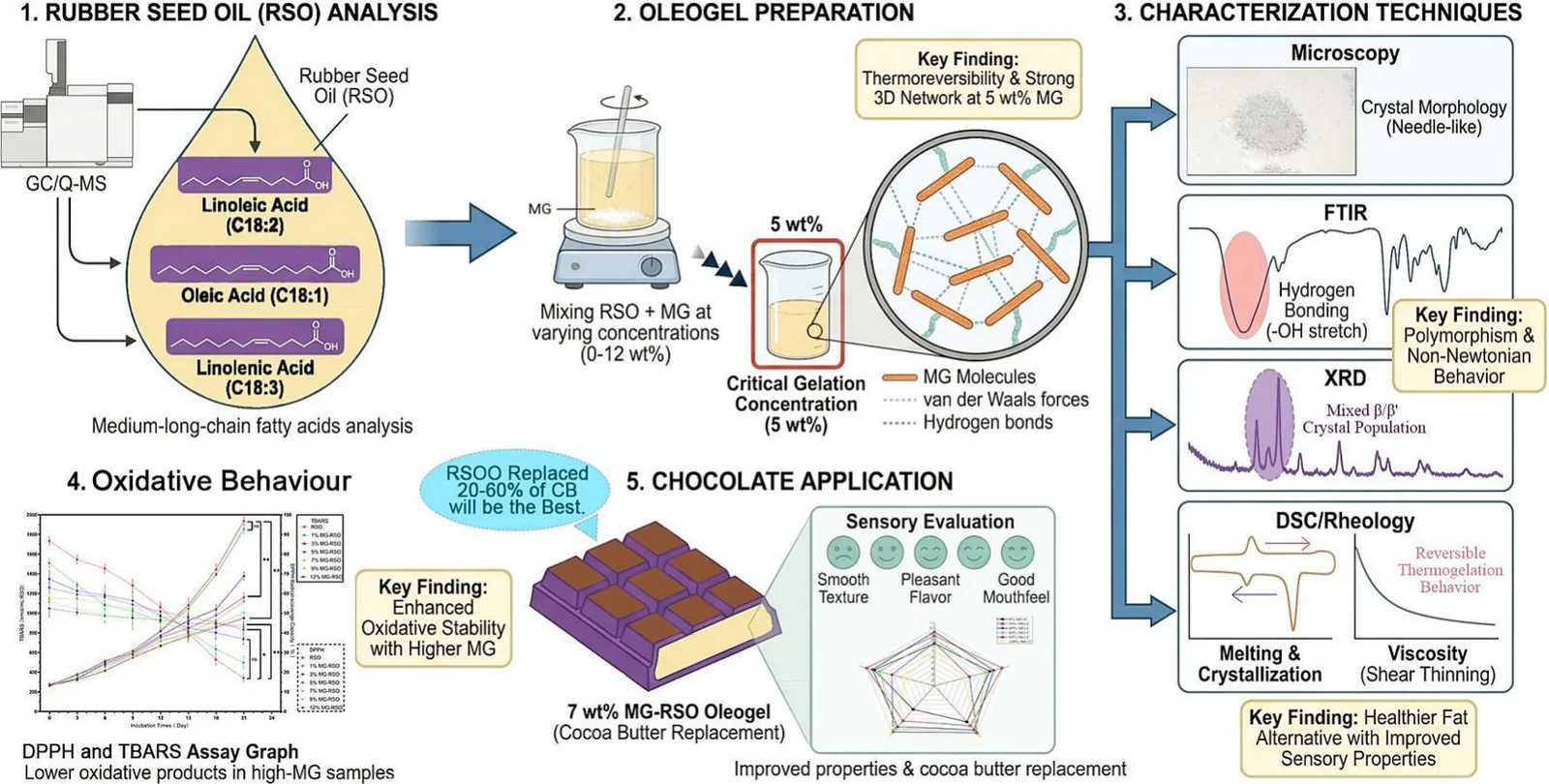

## 1 Introduction

Rubber tree (*Hevea brasiliensis*) seeds are rich in oil, with an oil content ranging from 40% to 50%. The seeds are abundant in various essential fatty acids for humans. Rubber seed oil (RSO) extracted from rubber seeds has long been used as a traditional edible oil in rubber-growing regions worldwide (Trinh et al., 2018). In China, RSO has a long history of consumption in Hainan Island, Xishuangbanna of Yunnan Province, and other areas (Agbai et al., 2021). Modern studies have demonstrated that the contents of oleic acid and linoleic acid in RSO are 25.26% and 37.26% (Liu et al., 2022), respectively, which are 3–4 times higher than those in soybean oil and rapeseed oil, and nearly 10 times higher than those in peanut oil and sunflower oil. Thus, RSO can provide essential nutritional support for daily diets (Ukhun & Uwatse, 1988). Cold-pressed RSO is a high-quality, health-promoting oil characterized by a high content of unsaturated fatty acids (up to 81%) and an unsaponifiable matter content of approximately 1.53%. In pharmacological studies by using cell models, RSO can effectively protect endothelial cells (Awlqadr et al., 2026) and reduce cellular inflammation (Liu et al., 2022), thereby affecting atherosclerosis and thrombosis. In conclusion, RSO, which has high content of linoleic acid and certain health effects, is an ideal candidate ingredient for nutritional or functional foods. However, there is still a lack of reliable clinical human research evidence to support its intervention effect on hyperlipidemia and cardiovascular disease. However, there is still a lack of reliable clinical human research evidence to support its intervention effect on hyperlipidemia and cardiovascular disease.

Oleogels are a class of semisolid colloidal systems formed by structuring liquid vegetable oils mediated by gelators (Awlqadr et al., 2026). Their core characteristic lies in the construction of a continuous three-dimensional network through specific interactions among gelator molecules, enabling efficient entrapment and immobilization of liquid oils (Yang, Hua, Li, et al., 2026). From the perspective of nutrition and health benefits, oleogels employ unsaturated vegetable oils as the main matrix, and can substitute solid lipid raw materials rich in trans fats and saturated fats widely used in food processing, such as hydrogenated vegetable oils and animal fats (Ferro et al., 2019; Papadaki et al., 2020; Perța-Crișan et al., 2022). The application of oleogels aligns closely with the global dietary guidelines proposed by the World Health Organization (WHO) for the total elimination of industrially produced trans fats and the gradual reduction of the proportion of dietary saturated fats. It provides key technical support for the development of low-fat and healthy foods, matching the escalating demand for healthy diets among consumers and the nutritional transformation trend of the food industry (Sivakanthan et al., 2022).

Among various gelators, low-molecular-weight crystalline gelators are the most widely applied in the food industry (Kavya et al., 2024). Representative examples that have been used on an industrial scale include glycerol monostearate, beeswax, and rice bran wax (Wang et al., 2023a). These gelators offer distinct advantages such as readily available sources, cost-effectiveness, and robust gelation performance. Their network formation mechanism exhibits clear physical crystallization characteristics. During processing, the gelator is heated and homogeneously dissolved in the liquid vegetable oil phase. As the system temperature is progressively lowered, gelator molecules crystallize and align to form fibrous, needle-like, or lamellar crystal aggregates. These crystals intertwine into a compact 3D network via intermolecular physical entanglement, van der Waals forces, and capillary effects, thereby achieving efficient immobilization of liquid oil (Guo et al., 2020; Li et al., 2025). At elevated gelator concentrations, the oil retention rate of oleogels generally exceeds 85%, thereby effectively suppressing oil migration and leakage while ensuring textural stability during food processing (Chen et al., 2025). To date, oleogels have been industrially applied in many fields related to food processing (Savchina et al., 2025).

Cocoa butter (CB) is a natural plant fat extracted from cocoa beans, which is a relatively expensive ingredient. Therefore, in chocolate production, most manufacturers seek substitutes for CB (Zhou et al., 2026), known as cocoa butter replacement (CBR). Among them, oleogels, as a kind of CBR (Zhang et al., 2023), will not affect the quality of chocolate while simulating the physical and sensory properties of saturated fat (Valdivia-Culqui et al., 2024). In general, oleogels, which are semi-solidified by constructing a 3D network (Sanpedro-Díaz et al., 2025) with gelators using liquid vegetable oil as the matrix, have significant advantages when used as CBRs: converting high-quality liquid vegetable oils into solid fats through physical gelation to replace traditionally hydrogenated and solidified fats can largely retain the active components of high-quality vegetable oils, avoid the formation of trans fatty acids during the heating and hydrogenation process, and ensure the nutritional quality of the oils (Awlqadr et al., 2026; Yang, Hua, Li, Qu, et al., 2026).

Glycerol monostearate, also known as monoglycerides (MG), is a commonly used emulsifier in food and can also act as a gelator to impart structure to liquid vegetable oils (Shuai et al., 2023). Also, RSO is an agricultural and industrial by-product that has not been fully utilized; Compared with traditional edible oils (such as sunflower seed oil, avocado oil, macadamia oil), its application as oil gel matrix is rarely explored. The purpose of this study was to prepare rubber seed oil oleogels (RSOOs) using easily accessible and low-cost MG as the gelator and expand the application of RSO in food. Meanwhile, the structure and physicochemical properties of RSOO, including macroscopic appearance, crystal type, thermal properties, hardness, oil-binding capacity and rheological behavior (Colella et al., 2023), were characterized for the first time. Furthermore, the thirty-seven fatty acid composition of RSO and the effect of oleogel formation on the oxidative stability of RSO were also investigated, as these properties can directly affect the shelf life of oleogels. In addition, the feasibility of using RSOOs as CBRs was studied, and the rheological properties and thermal behavior of chocolate based on RSOO were characterized (Santos et al., 2025). The results of this study will provide certain scientific guidance for the preparation of RSOOs and their application in the food industry.

## 2 Materials and methods

### 2.1 Materials and chemical reagents

Cold-pressed RSO was purchased from Xishuangbanna Huakun Biotechnology Co., Ltd. (Xishuangbanna, China), and third-party testing confirmed the absence of cyanogenic glycosides and pesticide residues (**S1**). Monoglycerides and 37 fatty acid methyl ester standards were purchased from Sigma-Aldrich Co., Ltd. (Shanghai, China). Cocoa butter and cocoa powder were obtained from Valrhona Sinodis Food (Shanghai) Co., Ltd. (Shanghai, China). Icing sugar was produced by Swire Sugar Holdings Limited (Hong Kong, China). All other analytical grade chemicals were purchased from Aladdin Biotechnology Co., Ltd. (Shanghai, China).

### 2.2 Determination of fatty acid content in RSO

Gas chromatography coupled with quadrupole mass spectrometry (GC/Q-MS) was used to identify the 37 fatty acids in the prepared RSO (Li et al., 2015), of these, 16 were detected and quantified in the present samples. Briefly, 37 common fatty acid standards (Table 1) were accurately weighed separately, and each was dissolved in chloroform to prepare a single standard stock solution with a concentration of 10 mg/mL. Aliquots of each single standard stock solution were mixed to prepare a mixed standard solution, which was stored for subsequent use. The samples were analyzed by GC/Q-MS according to the concentration gradients shown in the following table (Table 2).

**Table 1:** Standards for the determination of fatty acids.

No.	Name	Abbreviation	Molecular formula	CAS
1	Butyric acid	C4:0	C_4_H_8_O_2_	107-92-6
2	Hexanoic acid	C6:0	C_6_H_12_O_2_	142-62-1
3	Octanoic acid	C8:0	C_8_H_16_O_2_	124-07-2
4	Decanoic acid	C10:0	C_10_H_20_O_2_	334-48-5
5	Undecanoic acid	C11:0	C_11_H_22_O_2_	112-37-8
6	Lauric acid	C12:0	C_12_H_24_O_2_	143-07-7
7	Tridecanoic acid	C13:0	C_13_H_26_O_2_	638-53-9
8	Myristic acid	C14:0	C_14_H_28_O_2_	544-63-8
9	Myristoleic acid	C14:1	C_14_H_26_O_2_	544-64-9
10	Pentadecanoic acid	C15:0	C_15_H_30_O_2_	1002-84-2
11	cis-10-Pentadecenoic acid	C15:1	C_15_H_28_O_2_	84743-29-3
12	Palmitic acid	C16:0	C_16_H_32_O_2_	57-10-3
13	Palmitoleic acid	C16:1	C_16_H_30_O_2_	373-49-9
14	Heptadecanoic acid	C17:0	C_17_H_34_O_2_	506-12-7
15	cis-10-heptadecenoic acid	C17:1	C_17_H_32_O_2_	29743-97-3
16	Stearic acid	C18:0	C_18_H_36_O_2_	57-11-4
17	Elaidic acid	C18:1n9t	C_18_H_34_O_2_	112-79-8
18	Oleic acid	C18:1n9c	C_18_H_34_O_2_	112-80-1
19	Linolelaidic acid	C18:2n6t	C_18_H_32_O_2_	506-21-8
20	Linoleic acid	C18:2n6c	C_18_H_32_O_2_	60-33-3
21	Arachidic acid	C20:0	C_20_H_40_O_2_	506-30-9
22	Gamma-Linolenic acid	C18:3n6	C_18_H_30_O_2_	506-26-3
23	11-Eicosenoic acid	C20:1	C_20_H_38_O_2_	5561-99-9
24	Linolenic acid	C18:3n3	C_18_H_30_O_2_	463-40-1
25	Henicosanoic acid	C21:0	C_21_H_42_O_2_	2363-71-5
26	11C,14C-Eicosadienoic acid	C20:2	C_20_H_36_O_2_	2091-39-6
27	Docosanoic acid	C22:0	C_22_H_44_O_2_	112-85-6
28	cis-8,11,14-Eicosatrienoic acid	C20:3n6	C_20_H_34_O_2_	1783-84-2
29	Erucic acid	C22:1n9	C_22_H_42_O_2_	112-86-7
30	cis-11,14,17-Eicosatrienoic acid	C20:3n3	C_20_H_34_O_2_	17046-59-2
31	Tricosanoic acid	C23:0	C_23_H_46_O_2_	2433-96-7
32	Arachidonic acid	C20:4n6	C_20_H_32_O_2_	506-32-1
33	13C,16C-Docosadienoic acid	C22:2	C_22_H_40_O_2_	17735-98-7
34	Lignoceric acid	C24:0	C_24_H_48_O_2_	557-59-5
35	Eicosapentaenoic acid	C20:5n3	C_20_H_30_O_2_	10417-94-4
36	Nervonic acid	C24:1	C_24_H_46_O_2_	506-37-6
37	cis-4,7,10,13,16,19-Docosahexaenoic acid	C22:6n3	C_22_H_32_O_2_	6217-54-5

**Fig. 1:**
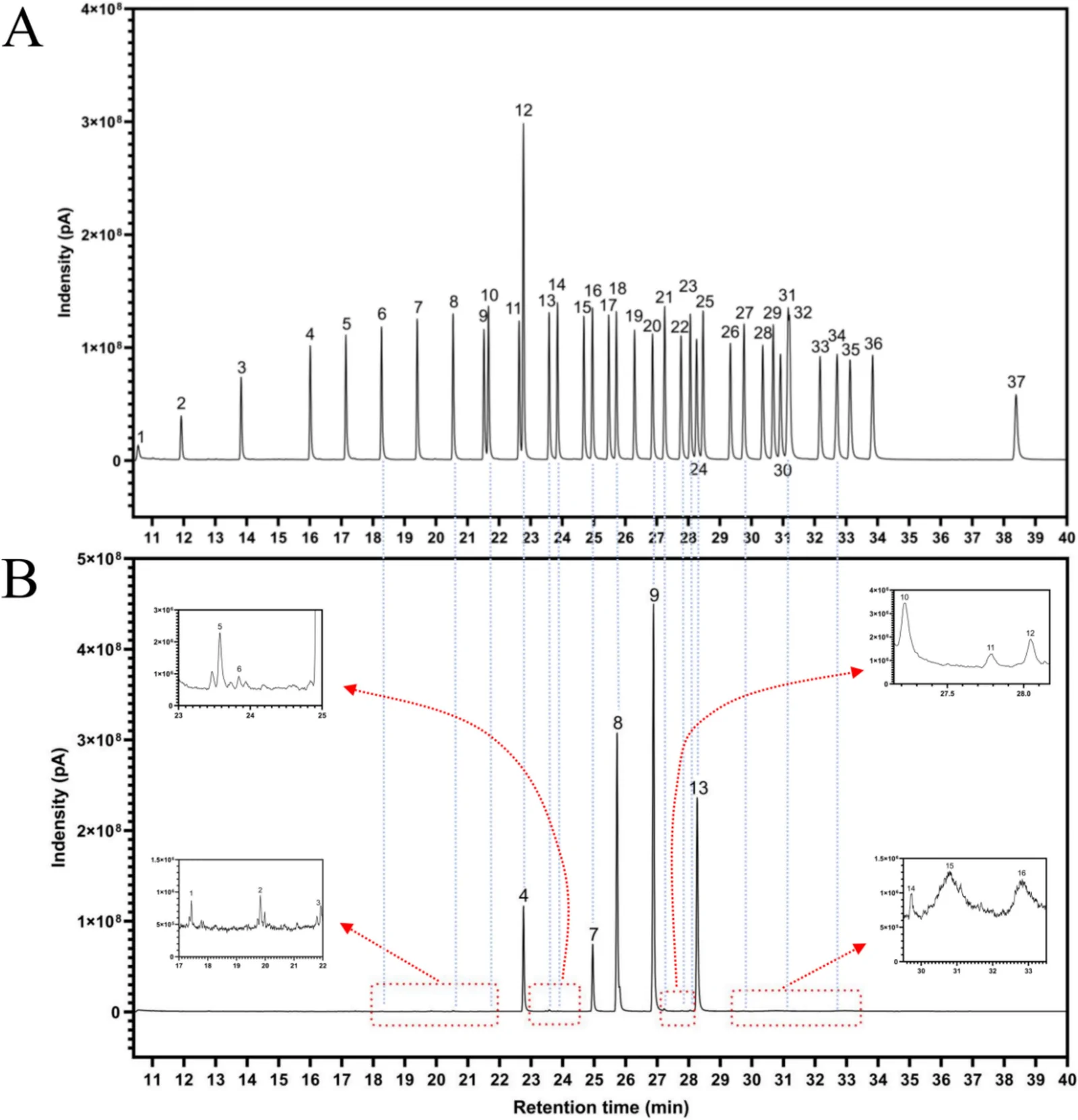
**A.** Gas Chromatogram of 37 Fatty Acids Standards; B. Gas Chromatogram of 37 Fatty Acids in RSO.

**Table 2:** The concentration gradient of standard for fatty acid determination.

Compound	Concentration (μg/mL)							
Palmitic acid	5.26	10.52	26.3	52.6	105.2	210.4	263	315.6
Other fatty acids	2.63	5.26	13.15	26.3	52.6	105.2	131.5	157.8

A specific mass of the sample was weighed into a centrifuge tube, 4 mL of chloroform was added, and the mixture was vortexed. Then, 0.9% sodium chloride solution was added and vortexed again. The mixture was centrifuged at 3500 rpm for 15 min at room temperature, and the lower phase was transferred to a new tube. Next, 2 mL of dichloromethane was added, vortexed, and centrifuged for 15 min. The lower phase was collected, and the extraction was repeated twice. The lower phases were combined, mixed, and evaporated to dryness under nitrogen. After drying, 2 mL of methanol (with 5% sulfuric acid) was added, vortexed, and incubated in a water bath at 80 °C for 2 h. After cooling, 2 mL of n-hexane and 1 mL of aqueous solution were added, vortexed for 30 s, and centrifuged at 2000 rpm for 5 min. The supernatant was collected, another 1 mL of aqueous solution was added, vortexed for 30 s, and centrifuged at 2000 rpm for 5 min. The resulting supernatant was evaporated to dryness under nitrogen, and the residue was reconstituted in isooctane. After vortexing and phase separation, the sample was analyzed instrumentally.

GC/Q-MS analysis was performed on an Agilent 7820 (Agilent Technologies, USA) gas chromatography system with a CP-Sil 88 capillary column (100 m × 0.25 mm × 0.25 μm, Agilent, USA). The injection volume was 1 μL at a 10:1 split ratio, with high-purity helium as carrier gas at 1.0 mL/min. The oven temperature programme was: 100 °C held for 5.0 min, ramped at 4 °C/min to 240 °C and held for 15 min. The inlet temperature was 260 °C and the quadrupole temperature 150 °C, mass spectra were acquired by electron impact (EI) in selected-ion-monitoring (SIM) mode over an *m*/*z* range of 30–550 using the MassHunter workstation.

Quantification was by external-standard calibration: a mixed standard containing all 37 fatty acids was analyzed at eight calibration levels (C16:0 at 5.26–315.6 μg/mL; the remaining 36 fatty acids at 2.63–157.8 μg/mL, Table 2). Each calibration curve was linear and forced through the origin, with coefficients of determination of R^2^ = 0.9906–0.9957 (mean 0.9929) across the 37 curves. No internal standard was used. The limits of detection (LOD) and quantification (LOQ), estimated at signal-to-noise ratios of 3 and 10 from the lowest calibration levels, ranged from 0.05 to 0.50 μg/mL and 0.15 to 1.50 μg/mL, respectively, depending on the fatty acid. Method precision was evaluated by three quality-control (QC) replicates per fatty acid within a single analytical run; the intra-day relative standard deviations were RSD = 6.49–11.13%. Inter-day precision and matrix-matched spiked-recovery experiments were not performed in this study. All fatty acids quantitative determinations were performed in four independent experimental repetitions (*n* = 4). (The detailed data are provided in **S2**)

### 2.3 Preparation of RSOOs

A precise quantity of RSO was accurately weighed, and MG were added to achieve concentrations of 0, 1.0, 3.0, 5.0, 7.0, 9.0, and 12.0 wt% (Shuai et al., 2023). The mixture was maintained at a constant temperature of 72 °C and magnetically stirred at 500 rpm until MG was completely dissolved in RSO. After cooling to room temperature and storing overnight in a refrigerator at 4 °C, RSOOs with different MG concentrations was obtained.

### 2.4 Characterization of RSOOs

#### 2.4.1 Microscopic observation

Macroscopic images of oleogels were captured in a controlled lighting environment using a digital camera (Nikon Z8, Nikon Inc.). The microstructure of the samples was examined using a microscope equipped with a digital camera (MTR3CMOS20000KPA).

A small drop of molten oleogel was deposited on a glass slide, covered with a coverslip, and then stored at 4 °C overnight. Subsequently, micrographs were acquired using the camera system.

#### 2.4.2 Fourier transform infrared (FTIR) spectroscopy analysis

The FTIR spectra of RSOOs and MG were recorded on an FTIR spectrometer (Thermo Scientific™ Nicolet iS10) equipped with an attenuated total reflection (ATR) accessory. The measurements were performed following the research method proposed by Wang (Wang et al., 2023b).

#### 2.4.3 X-ray diffraction (XRD) analysis

X-ray diffraction patterns of RSOOs and MG were obtained using an X-ray diffractometer. The detailed procedure followed the method reported by Colella MF (Colella et al., 2023). The crystalline morphology of the oleogels was analyzed using a Malvern PANalytical X'Pert3 X-ray diffractometer with Cu-Kα radiation at 40 kV and 30 mA. An appropriate amount of sample was placed in the sample holder. The 2θ scanning range was set from 5° to 50° at a scanning speed of 2°/min with a step size of 0.01°. All data were analyzed using OriginPro 2026b (64-bit) Beta2 (10.3.5.621991-2026 OriginLab Corporation, USA).

#### 2.4.4 Thermal behavior analysis

The thermal behavior curves of RSOOs and MG were recorded using a differential scanning calorimeter (Nanjing DZ-DSC 300C). Thermal analysis was performed according to the method described by Santos PHS (Santos et al., 2025), with slight modifications. Specifically, 5–10 mg of oleogel samples were sealed in an aluminum crucible, with an empty aluminum crucible serving as a control. The samples were heated to 85 °C at a rate of 5 °C/min under a continuous nitrogen flow of 30 mL/min and held for 10 min to eliminate the crystal memory effect, followed by cooling to 0 °C at a rate of 5 °C/min. Finally, the changes in enthalpy during the crystallization process (85–0 °C 5 °C/min) and melting process (0–85 °C, 5 °C/min) of the samples were analyzed by measuring heat flow as a function of temperature during the cooling and heating processes.

#### 2.4.5 Oil binding capacity (OBC) analysis of RSOOs

The OBC was analyzed by the centrifugation method adapted from Li (Li et al., 2024). Approximately 1 g of oleogel was placed into a pre-weighed centrifuge tube (m1), subsequently, the tube containing the sample was weighed (m2).

The samples were centrifuged twice at 12,000 r/min for 15 min each time. Subsequently, the centrifuge tube was inverted for 15 min to drain excess oil, and then weighed again (m3). The experiment was performed in triplicate, and the results were reported as mean values. The formula for calculating OBC is as follows:

(1) $$\mathrm{OBC}=\frac{m3-m1}{m2-m1}\times 100\%$$

The analysis results were presented as the percentage of the mass of oil separated by centrifugation relative to the total mass of the samples.

#### 2.4.6 Hardness analysis

The hardness of the RSOOs was analyzed using a texture analyzer (SMS TA.XT Plus) equipped with a cylindrical probe (P/0.5). Firstly, 15–20 g of melted sample was placed into a small beaker and stored overnight in a refrigerator at 4 °C to ensure that the thickness of the solidified and shaped sample was greater than 20 mm. Subsequently, the sample was equilibrated at 25 °C, and texture analysis was performed under the following conditions: a pre-test speed of 2 mm/s, a test speed of 1 mm/s, a test distance of 10 mm, a post-test speed of 2 mm/s, and a trigger force of 5 g. Each group of samples was tested in triplicate, and the results were expressed as the mean values. The final results were represented by the maximum trigger force at the specified test distance.

#### 2.4.7 Rheological properties

Following the method described by Hong (Hong et al., 2022), the rheological properties of RSOOs were analyzed using a dynamic shear rheometer equipped with a temperature control system (Kinexus ultra+). The measuring unit used in the experiment consisted of parallel plates with a diameter of 40 mm and a plate gap of 1 mm.

First, a stress sweep was performed at 25 °C to determine the linear viscoelastic region (LVR) of the oleogels; results indicated that an applied stress of 1 Pa fell within the LVR. Subsequently, a dynamic frequency sweep was conducted, during which variations in complex viscosity, storage modulus (G′), and loss modulus (G″) were recorded in the frequency range of 0.1 to 25 Hz. In addition, under fixed frequency (1 Hz) and strain (0.01%) conditions, the RSOOs were subjected to heating and cooling temperature scans from 5 to 85 °C at a constant rate of 5 °C/min to evaluate its thermal sensitivity.

#### 2.4.8 Oxidative behavior and stability

The oxidative behavior of RSOOs was evaluated by an accelerated oxidation test. Oleogel test samples with different MG concentrations were transferred to test tubes and stored in a dark environment at 40 °C. Subsequently, thiobarbituric acid reactive substances (TBARS) levels were measured using a commercial kit (Beijing Solarbio Science & Technology Co., Ltd., Malondialdehyde (MDA) Content Assay Kit (TBARS Method), Cat. BC6415, 100T/96S) and DPPH free-radical-scavenging capacity was determined using a commercial kit (Nanjing Jiancheng Bioengineering Institute, DPPH kit, cat. A153-1-1, 48T) at intervals of 0, 3, 6, 9, 12, 15, 18, and 21 days. DPPH reports radical-scavenging capacity whereas TBARS reports secondary oxidation products, the two assays were therefore used as complementary indicators of oxidative behavior.

After the 90 days storage at 4 °C, RSOOs' OBC was re-measured by the same centrifugation protocol described in Section 2.4.5 to track long-term physical stability of the crystal network.

### 2.5 Application of RSOOs: preparation of oleogel-based chocolates

The translation of oleogel properties from the bulk gel to a real food matrix is a critical step that determines practical utility, because performance depends not only on the gel itself but also on its interactions with cocoa solids, sugar, milk powder and emulsifiers. Recent work has demonstrated that pectin-LDL oleogels can successfully replace fat in gluten-free bread, improving both structural and textural characteristics (Abou-Elsoud et al., 2026), confirming that oleogel-based fat replacers can function in complex food systems. In the present study, the 7 wt% MG RSOO was evaluated as a partial CBR in a model chocolate formulation. A general chocolate formula was referenced and adapted accordingly. The experimental chocolate formulation (per 100 g) consisted of 50 g cocoa liquor, 10 g cocoa powder, a combined total of 25 g comprising 7.0 wt% MG-RSO oleogel and CB at varying ratios (0, 20%, 40%, 60%, 80%, and 100%), along with 2 g soybean lecithin, 1 g vanillin, 8 g skimmed milk powder, 1 g milk flavoring, and 3 g maple syrup.

According to the above formula, the mixture of CB, oleogel, and cocoa powder was heated in a water bath at 80 °C. Once completely melted, the mixture was stirred continuously to ensure homogeneity, yielding a cocoa slurry. Sweetener and skimmed milk powder were added in small portions, and the mixture was fully stirred until the components were uniformly distributed to obtain chocolate slurry. Then, the chocolate slurry at 80 °C was cooled to 35 °C in cold water, taken out and continuously stirred to 27 °C, then heated to 32 °C. The operations of cooling to 27 °Cand heating to 32 °C were repeated several times until the chocolate slurry became smooth and viscous. Finally, the smooth chocolate slurry at 27 °C was poured into molds, allowed to cool to room temperature, and subsequently stored in a refrigerator at −4 °C for 60 min. Upon demolding, oleogel-based chocolates containing varying concentrations of RSO were obtained.

### 2.6 Characterization of oleogel-based chocolates

#### 2.6.1 Thermal behavior analysis

To obtain the melting curves of the oleogel-based chocolates, the differential scanning calorimeter described above (2.3.4) was used. The heat flow changes were measured when the chocolates were heated from 10 °C to 85 °C at a rate of 5 °C/min and cooled from 85 °C to 10 °C at the same rate to characterize their thermal behavior.

#### 2.6.2 Thermal tolerance of RSOO based chocolates analysis

According to the method described by Shuai (Shuai et al., 2023) with slight modifications: the Chocolate samples were stored in an oven (Jiyi instrument, 101-0B) at different temperatures (20, 25, 30, 35 and 40 °C) for 15 min, and then the shape changes of the samples were recorded by taking photos in the studio equipped with a digital camera to characterize the thermal tolerance of MG-RSOO-based chocolates.

#### 2.6.3 Rheological properties

The rheological properties of oleogel-based chocolate were analyzed using an MCR302 rheometer equipped with a temperature control system. A 40-mm-diameter parallel plate geometry was used with a gap setting of 1 mm.

To determine the LVR of the samples, a stress sweep test was performed at 40 °C. The test results showed that a stress of 1 Pa was within the LVR, which was therefore used for subsequent experiments. Subsequently, a frequency sweep was conducted, and the storage modulus (G′) and loss modulus (G′′) were recorded at frequencies ranging from 0.1 to 100 Hz. The obtained data were fitted to a power-law model to evaluate the frequency dependence of G′. The samples were stabilized in a water bath at 40 °C for 30 min, followed by shear and frequency sweep analyses. The shear rate range was from 0.01 to 150 s^−1^.

#### 2.6.4 Sensory evaluation analysis

The sensory evaluation of chocolate samples was conducted with reference to the quantitative descriptive analysis method proposed by Richardson (Richardson et al., 2021). Table 3 shows the sensory evaluation items and scoring criteria.

**Table 3:** The sensory evaluation items and scoring criteria for regular chocolate and RSO oleogel-based chocolate at different concentrations.

Item	Definition	Scoring criteria
Appearance	Whether the appearance is smooth and uniform in color, without blooming or cracking; whether the texture is firm, without looseness or caking.	12–10 points: Smooth appearance, uniform color, no blooming or cracking, firm and delicate texture; 9–7 points: Relatively smooth appearance, relatively uniform color, no obvious blooming or cracking, relatively firm texture; 6–4 points: Basically smooth appearance, basically uniform color, slight blooming, relatively soft texture; 3–1 points: Rough appearance, uneven color, obvious blooming and cracking, loose or caked texture.
Flavor Coordination	Whether there is a peculiar odor unique to RSO (similar to vegetable oil odor or green taste).	12–10 points: No peculiar odor of RSO, rich and pure cocoa flavor, highly coordinated flavor; 9–7 points: Extremely slight peculiar odor of RSO, negligible, relatively pure cocoa flavor, basically coordinated with oleogel flavor; 6–4 points: Slight peculiar odor of RSO, average cocoa flavor, average coordination with oleogel flavor, and RSO has a slight impact on flavor but basically meets the qualified line of chocolate flavor; 3–1 points: Obvious peculiar odor of RSO, weak cocoa flavor, seriously uncoordinated with oleogel flavor, which seriously affects the taste.
Taste Delicacy	Whether there is a uniform and delicate taste in the mouth after the sample melts, without caking or gritty feeling.	12–10 points: Uniform and delicate taste, no caking or gritty feeling at all, melts in the mouth instantly, overall taste is coordinated, and the particle size is extremely small; 9–7 points: Relatively delicate taste, occasional extremely fine caking, no gritty feeling; 6–4 points: Average taste, slight caking, no gritty feeling, moderate particle size; 3–1 points: Rough taste, obvious caking and gritty feeling.
Melting Rate	Whether the melting rate is moderate; whether the melting process is stable.	12–10 points: Completely melts within 10–15 s in the mouth, no any particle feeling, blockage, smooth and delicate melting process, fits the oral mucosa without friction, smooth swallowing without residue, excellent softness; 9–7 points: Melts within 15–25 s in the mouth, occasional fine particle feeling, relatively smooth melting, no obvious friction feeling, basically smooth swallowing, extremely light residue feeling; 6–4 points: Melts within 25–35 s in the mouth, slight particle feeling, blockage, basically smooth melting process, slight sticky throat feeling when swallowing, average softness; 3–1 points: Not completely melted after more than 35 s in the mouth, obvious particle feeling, blockage, rough melting, strong friction feeling, serious sticky throat when swallowing, no softness at all.
Aftertaste	The degree of fragrance remaining in the mouth after eating and swallowing the sample.	12–10 points: After swallowing, the mouth still retains a rich and pure cocoa flavor, with a light and non-abrupt RSO flavor remaining; 9–7 points: After swallowing, the mouth still retains a certain cocoa flavor and RSO flavor, and the coordination is good; 6–4 points: After swallowing, the mouth still retains a light cocoa flavor, with an abrupt RSO flavor remaining in the mouth, and the flavor coordination is average; 3–1 points: Obvious RSO flavor remains in the mouth for a long time, and the cocoa flavor is weak, which seriously affects the aftertaste process of chocolate.

Thirty testers (15 males and 15 females) were required to refrain from consuming spicy, sour-sweet, and strong-flavored foods, as well as alcoholic and caffeinated beverages, within 1 h before the evaluation to maintain the sensitivity of taste and smell. After tasting the samples, the testers were asked to indicate their preference for the test samples on a scale of 0 to 12 points. Prior to evaluation, chocolate samples were cut into uniform cubes (2 cm × 2 cm × 2 cm), equilibrated at room temperature (25 ± 1 °C) for 15 min, and presented with random numerical codes.

The sensory test was conducted in the same controlled environment under white light. Between samples, the testers rinsed their mouths with clean water to minimize carryover effects. Finally, mean scores for each sensory attribute were calculated.

### 2.7 Data analysis

All samples were analyzed in triplicate, and the results were expressed as mean ± standard deviation (SD). Statistical analysis was performed using OriginPro 2026b (64-bit) Beta2, GraphPad Prism 10 (64-bit) 10.1.2(324) and IBM SPSS Statistics 22.0.0.0 (64-bit). One-way analysis of variance (ANOVA) was used to determine significant differences among groups (*P* < 0.05).

## 3 Results and discussion

### 3.1 Fatty acid content in RSO

After comparing the obtained gas chromatogram of fatty acids in RSO against the liquid chromatogram of fatty acid standards, the fatty acid content in RSO was determined and is presented in the following table:

Thirty-seven fatty acids were screened by GC/Q-MS, of which 16 were detected and quantified in RSO (Table 4). The profile was dominated by long-chain C16-C18 species, which together accounted for 99.54% of the detected fatty acids; shorter and longer chain lengths occurred only as traces. Linoleic acid (C18:2n-6, 40.39%), oleic acid (C18:1n-9, 22.86%) and alpha-linolenic acid (C18:3n-3, 21.72%) were the major unsaturated species, while palmitic (C16:0, 8.23%) and stearic (C18:0, 6.07%) acids predominated among the saturated fraction. Summation of the detected species gave class totals of 14.65% saturated fatty acids (SFA), 23.15% monounsaturated fatty acids (MUFA) and 62.20% polyunsaturated fatty acids (PUFA), i.e. 85.35% total unsaturated fatty acids, with an n-6/n-3 ratio of 1.863. This composition is broadly consistent with earlier reports on RSO (Agbai et al., 2021; Liu et al., 2022).

**Table 4:** 37 fatty acids content (FAC) in RSO.

No. (in Fig. 1.B.)	Name	Abbreviation	FAC (ug/mL) ± SD	Proportion %
1	Lauric acid	**C12:0**	0.0713 ± 0.0284	0.0048
2	Myristic acid	**C14:0**	1.0921 ± 0.2058	0.0742
3	Pentadecanoic acid	**C15:0**	0.0603 ± 0.0221	0.0041
4	Palmitic acid	**C16:0**	121.0276 ± 24.1905	8.2273
5	Palmitoleic acid	**C16:1**	2.7380 ± 0.5101	0.1861
6	Heptadecanoic acid	**C17:0**	0.4324 ± 0.1204	0.0294
7	Stearic acid	**C18:0**	89.3068 ± 19.3889	6.0710
8	Oleic acid	**C18:1n9c**	336.3193 ± 39.1009	22.8626
9	Linoleic acid	**C18:2n6c**	594.1202 ± 105.5922	40.3877
10	Arachidic acid	**C20:0**	2.5449 ± 0.6077	0.1730
11	Gamma-Linolenic acid	**C18:3n6**	1.2703 ± 0.1028	0.0864
12	11-Eicosenoic acid	**C20:1**	1.5303 ± 0.4594	0.1040
13	Linolenic acid	**C18:3n3**	319.5631 ± 55.2917	21.7236
14	Docosanoic acid	**C22:0**	0.5276 ± 0.1453	0.0359
15	Tricosanoic acid	**C23:0**	0.1632 ± 0.0513	0.0111
16	Lignoceric acid	**C24:0**	0.2765 ± 0.1032	0.0188

Attention: Data of FAC (ug/mL) are expressed as mean ± standard deviation, *n* = 4.

These values describe the molecular make-up of the oil and should not, by themselves, be read as evidence of a demonstrated physiological or health benefit. A high PUFA content and an n-6/n-3 ratio close to 2 are compositional features that are commonly discussed as nutritionally desirable (Simopoulos, 2008; Zhang, Huang, et al., 2024). In the absence of validated, clinically controlled evidence for RSO specifically.

### 3.2 Oleogel characterization

#### 3.2.1 Appearance and microscopic observation of oleogels

The formation of oleogels is primarily governed by the type and concentration of the gelator used (Dimakopoulou-Papazoglou et al., 2023). MG is a low-molecular-weight organogelator composed of a polar head group (glycerol) and non-polar tail groups (stearic acid) (Awlqadr et al., 2026). Studies have shown that MG can transform liquid oil into semi-solid fat by dissolving the gelator in hot oil and then cooling to induce crystallization, thereby forming a 3D crystal network (Dimakopoulou-Papazoglou et al., 2023) (Fig. 2).

**Fig. 2:**
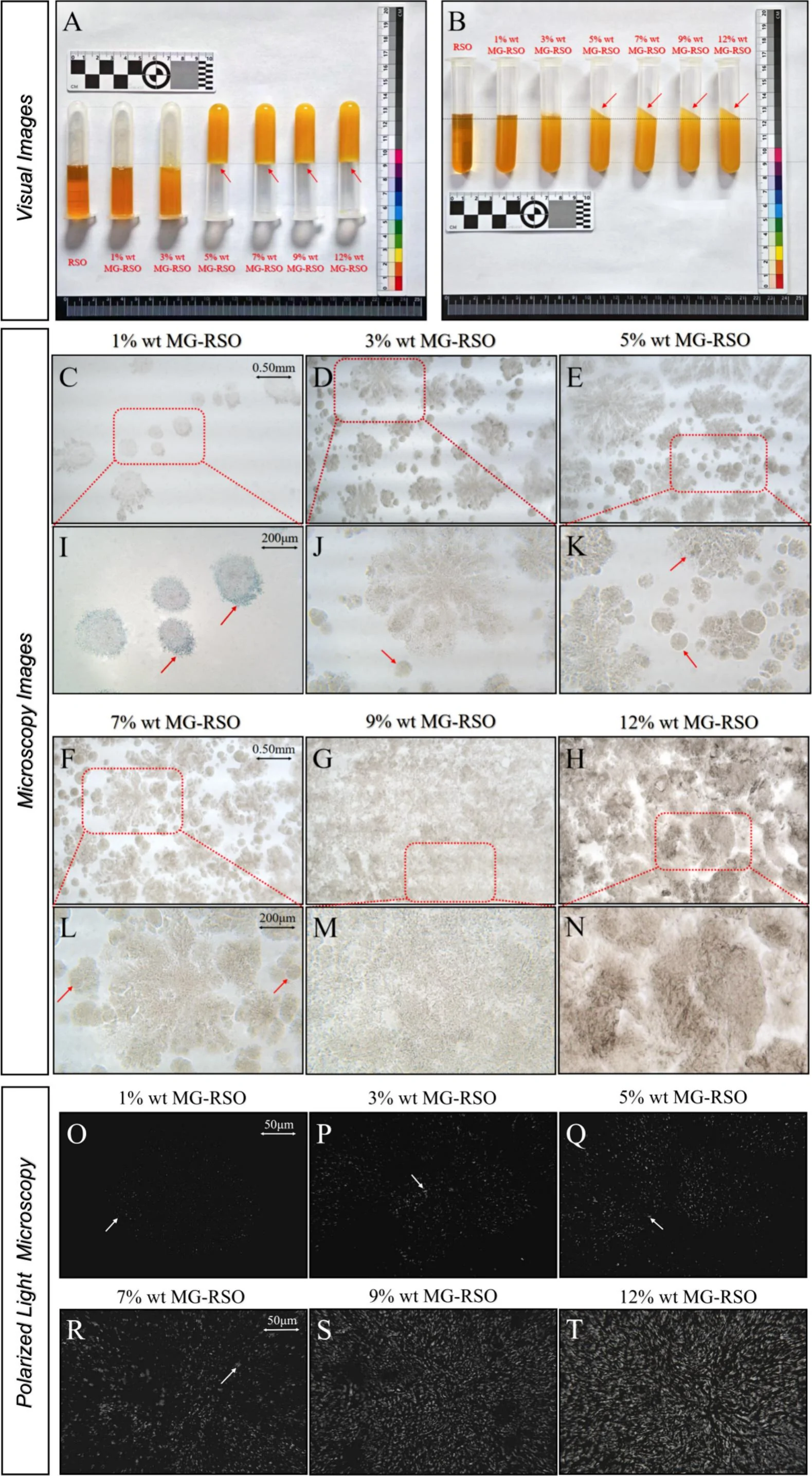
**A-B**. Appearance images of RSOO; **C—N.** Microscopic images of RSOO with different gelator contents; **O-T**. Polarized microscopic images of RSOO with different gelator contents.

In this study, the MG content required for RSO to form self-supporting oleogels was determined based on macroscopic appearance and microscopic morphology. The method of appearance observation was as follows: test tubes containing RSOOs with different gelator contents were inverted or placed sideways at 45°, and then placed vertically after the oleogels solidified. As shown in Fig. 2A-B, the minimum MG content required to form a self-supporting RSO oleogel was determined by the inverted-tube method at 25 °C. At MG contents below 5.0 wt%, the samples flowed upon inversion and could not support their own weight. At 5.0 wt% MG and above, the mixtures set into opaque, non-flowing semi-solids that retained their shape on inversion. The original micrographs are provided in **S3**.

Fig. 2C-N shows the morphological structure of RSOOs under a microscope. Quantitative image analysis (Fig. 2C-H) was performed on the light micrographs using adaptive thresholding and connected-component labelling (minimum area = 30 μm^2^; scale calibrated at 0.615 μm/pixel with a stage micrometer). The results are summarised in Table 5.

**Table 5:** Quantitative analysis of MG-RSO oleogel microstructures by light microscopy.

MG concentration (wt%)	1%	3%	5%	7%	9%	12%
Crystal number (per field)	1863	1830	1454	1505	773	682
Number density (crystals/mm^2^)	249	244	194	201	103	91
Network coverage (%)	10.2	19.1	26.9	31.5	43.3	44.5
Mean equivalent diameter (μm)	15.5 ± 15.8	20.9 ± 23.3	25.5 ± 33.2	27.9 ± 34.7	23.7 ± 69.1	21.7 ± 75.7
Median diameter (μm)	11.5	13.8	15.3	14.8	14.1	12.9
Aspect ratio (L/W)	1.37	1.45	1.45	1.43	1.42	1.46
Circularity (4πA/P^2^)	0.57	0.54	0.47	0.48	0.52	0.49
Dominant morphology	Small, sparse, mostly round/oval	Round aggregates + early dendritic clusters	Rosette/dendritic aggregates, moderate density	Dendritic aggregates, partial network formation	Fine, continuous crystalline network	Dense, continuous network with small crystallites

Network coverage increased monotonically with MG concentration, from 10.2% at 1 wt% MG to 44.5% at 12 wt% MG, confirming progressive development of the crystalline network. Mean equivalent crystal diameter rose from 15.5 μm (1% MG) to a maximum of 27.9 μm at 7 wt% MG, then decreased slightly at higher concentrations as individual aggregates merged into a continuous network. Crystal number density was highest at low MG (249 crystals/mm^2^ at 1%) and declined to 91 crystals/mm^2^ at 12%, consistent with coarsening and fusion of crystallites. Aspect ratios ranged from 1.37 to 1.46 and circularities from 0.47 to 0.57, indicating predominantly irregular, rosette- or dendritic-shaped aggregates rather than perfect spheres. At MG concentrations ≥ 9 wt%, the crystals formed a visually continuous, fine-meshed network rather than discrete particles, which accounts for the reduced particle count and increased standard deviation in equivalent diameter at these concentrations. These microstructural trends are consistent with the hardness, oil-binding capacity, XRD and rheological data reported in subsequent sections.

Polarized light microscopy was used to observe the microstructure of RSOOs prepared with different MG concentrations (Fig. 2O-T). It was found that high concentrations of MG formed needle-like crystals in the oil phase, which were uniformly distributed throughout the oil phase. At MG concentrations below 5%, the gelator content was insufficient to establish a stable 3D crystal network within the RSO; consequently, the samples failed to support their own weight and exhibited flow. With the increase of MG concentration, the size and number of crystals in the oleogel system increased. Once the concentration reached a critical threshold, the MG crystal network percolated throughout the entire oil phase, thereby imparting solid-like properties to the samples. The higher the MG concentration, the higher the degree of supersaturation of the hot MG-RSO mixed solution during the cooling process, which would promote nucleation and crystallization, thereby forming a stronger crystal network.

#### 3.2.2 Fourier transform infrared (FTIR) spectroscopy analysis

Fourier transform infrared (FTIR) spectroscopy is widely used to analyze the molecular structure and intermolecular interactions in composite materials (An et al., 2026). Therefore, FTIR spectra of the MG and RSOOs were recorded, as shown in Fig. 3A.

**Fig. 3:**
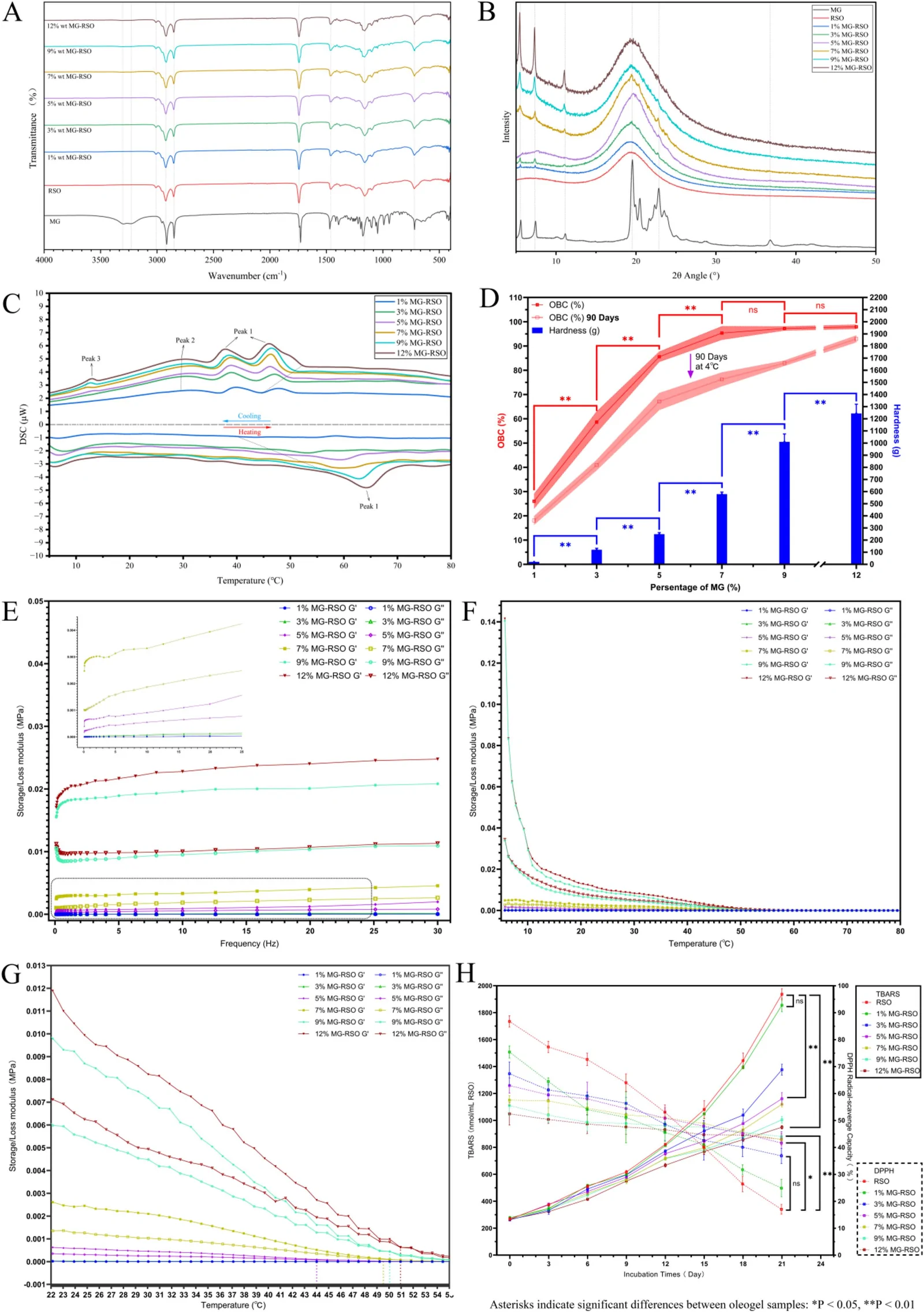
**A.** FTIR spectra of RSOOs with different gelator contents; **B.** XRD spectra of RSOOs with different gelator contents; **C.** DSC curves of RSOOs with different gelator contents; **D.** Line charts of hardness and OBC (0/90 Days) of RSOOs with different gelator contents; **E.** frequency sweep curves of storage modulus (G′) and loss modulus (G″) of RSOOs with different gelator contents; **F.** Temperature sweep curves of storage modulus (G′) and loss modulus (G″) of RSOOs with different gelator contents; **G.** Schematic diagram of melting temperature (Tm) intersection point of RSOOs with different gelator contents; **H.** Oxidation process of RSOOs with different gelator contents (TBARS and DPPH). Different gelator contents means RSOOs prepared with 1.0, 3.0, 5.0, 7.0, 9.0 and 12.0 wt% of MG. (*n* = 3, *: *P* < 0.05, **: *P* < 0.01).

In RSO and MG, absorption peaks were observed around 2922.59, 2853.17 and 1742.85 cm^−1^, which were attributed to the stretching vibrations of C—H and C—O groups. In RSO, a small and sharp absorption peak was observed at 3009.37 cm^−1^, which was attributed to the stretching vibration of -CH. The absorption peaks observed in the range of 1000–1500 cm^−1^ were mainly attributed to the C—H bending vibrations of -CH_2_ (around 1464 cm^−1^) and -CH_3_ (around 1377 cm^−1^), as well as the C—O stretching vibrations of C-OH (around 1164 cm^−1^) and C-O-C (around 1093 cm^−1^). The peak observed at 720 cm^−1^ was attributed to the rocking vibration of long methylene chains [(CH2)_n_], indicative of the alkyl chain structure.

Compared with the FTIR spectra of RSO and MG powder, no new peaks emerged nor did any existing peaks disappear in the oleogel samples, indicating that no covalent bond changes occurred during the formation of oleogels. As MG content increased, the C—H stretching bands shifted slightly, from 2922.59 and 2853.17 cm^−1^ to 2920.18 and 2851.72 cm^−1^, and the broad O—H envelope in the 3031–3454 cm^−1^ region increased in intensity. These small shifts and the intensification of the O—H region are consistent with van der Waals contacts between acyl chains and intermolecular hydrogen bonding involving MG hydroxyl groups; they are therefore interpreted as supportive of a physically assembled (non-covalent) crystal network.

#### 3.2.3 X-ray diffraction (XRD) analysis

XRD analysis was used to provide information about the physical state of RSOOs. The diffraction patterns of oleogels formed with different MG concentrations are shown in Fig. 3B.

Diffraction short spacings appeared at 4.4–4.7 Å (corresponding to 2θ of 18.7–20.1°) and at 3.8–4.0 Å (2θ of 22.0–23.2°). In monoglyceride and fat-crystal systems, a short spacing near 4.5 Å is conventionally assigned to the triclinic β polymorph, whereas short spacings near 3.8–4.3 Å are characteristic of the orthorhombic β’ polymorph (Pratama et al., 2022). On this basis, the 4.4–4.7 Å band is attributed mainly to β and the 3.8–4.0 Å band to β′ crystals, i.e. the RSOOs contain a mixed β/β′ crystal population. The smaller crystals associated with the β’ form generally favour a denser network and greater oil entrapment (Bharti et al., 2022). As MG content increased, the intensity of both characteristic reflections increased, but neither the d-spacings nor the 2θ positions shifted. This indicates that increasing MG loading increased the amount of β/β’ crystals without changing the polymorphic form adopted. This interpretation agrees with the polarized-light microscopy observation of more numerous birefringent crystals, and is consistent with monoglyceride oleogels in sunflower (Zhang, Geng, et al., 2024) and avocado (Moreno-Caballero et al., 2026) oils.

#### 3.2.4 Thermal behavior analysis

The thermal properties of oleogels with different MG concentrations were analyzed by differential scanning calorimetry (DSC) (Giacomozzi et al., 2020; Meng et al., 2018; Zhao et al., 2020). Fig. 3C shows the DSC curves of oleogels during the cooling and heating processes.

Except for pure oil, the cooling curves of all samples showed three peaks: a small peak appeared at 11–18 °C, and the other two larger peaks appeared at 36–43 °C and 45–49 °C. The presence of these peaks indicates that MG exists in different polymorphic forms, which is similar to the previous research results on other oleogels induced by MG (López-Martínez et al., 2014). The melting curves of all samples showed two larger peaks, which appeared at 4–8 °C and 55–65 °C, respectively.

During the cooling process, it was observed that with the increase of MG content, both the transition temperature (Tm) and enthalpy change (ΔHm) of the first crystallization peak (Peak 1) increased. Specifically, when the MG content increased from 1% to 12%, Tm_1_ increased from 45.18 °C to 52.00 °C, and ΔHm_1_ increased from 5.7508 J/g to 14.5730 J/g. The transition temperature of the second crystallization peak (Peak 2) remained relatively stable despite increases in MG content; Tm_2_ stayed approximately at 30 °C, and ΔHm_2_ did not exceed 5.0 J/g. The third crystallization peak (Peak 3) observed during the cooling process appeared with the increase of MG content, and both the transition temperature and enthalpy change of this peak were relatively low, with ΔHm_3_ not exceeding 1.5 J/g (Table 6). The increase in enthalpy change of crystallization Peak 1 can be attributed to more MG molecules participating in the crystal formation process. The increase in crystallization temperature of Peak 1 with the increase of MG content may be due to the increased supersaturation promoting nucleation. However, the unchanged transition temperature of Peak 2 with MG content and the low enthalpy change of Peak 3 may be related to the polymorphic transition of existing crystals involved in the two peaks.

**Table 6:** The thermal behavior of RSOOs determined by differential scanning calorimetry (DSC).

Sample	Cooling						Heating	
	Peak 1		Peak 2		Peak 3		Peak 1	
	Tm / °C	△Hm / J/g	Tm / °C	△Hm / J/g	Tm / °C	△Hm / J/g	Tm / °C	△Hm / J/g
1% MG-RSO	45.18	5.7508	31.45	1.6894	/	/	39.24	3.8947
3% MG-RSO	42.72	4.0270	28.78	2.4339	/	/	47.31	1.2644
5% MG-RSO	48.81	7.4906	30.38	2.9532	/	/	50.75	8.1660
7% MG-RSO	48.60	8.3521	31.22	3.1935	13.56	0.3979	57.51	9.2373
9% MG-RSO	49.63	11.6688	30.69	3.4756	13.69	0.7132	54.63	16.3554
12% MG-RSO	52.00	14.5730	30.38	4.1544	13.89	1.2907	58.93	19.0850

A similar trend was observed during the melting process. Both the transition temperature (Tm) and enthalpy change (ΔHm) of the peak (Peak 1) appearing during the heating process increased significantly with the increase of MG concentration, from 39.24 °C and 3.8947 J/g to 58.93 °C and 19.0850 J/g, respectively. This result indicates that with the increase of MG concentration in the oleogel, the melting point of MG-RSO crystals also increases, which is similar to the results of relevant oleogel studies (Shuai et al., 2023; Simopoulos, 2008). These characteristics render RSOOs more suitable for transport in high-temperature environments; alternatively, MG-induced oleogels could replace conventional RSO to enhance the thermal stability of related products (Sena et al., 2022).

#### 3.2.5 Hardness and OBC of RSOOs

Hardness and oil binding capacity (OBC) reflect the mechanical strength and oil retention performance of oleogels, respectively. These parameters depend on the fat crystal network of oleogels (Tanislav et al., 2022). Fig. 3D illustrates the hardness and OBC of oleogel samples prepared with varying MG concentrations.

As the MG concentration increased from 1.0% to 12.0%, the hardness of RSOOs increased significantly from 20.045 g to 1127.846 g (*P* < 0.005), and the OBC value also increased markedly from 24.92% to 99.87% (*P* < 0.005). At the molecular level, this improved OBC is consistent with the principle that strengthened intermolecular interactions - particularly hydrogen bonding between MG hydroxyl groups and van der Waals contacts between alkyl chains—enhance network cohesiveness and reduce oil migration. A recent study of foam-template oleogels demonstrated that deliberately increasing polysaccharide molecular interactions produced a corresponding gain in oil-binding capacity (Wang et al., 2026), supporting the interpretation that the OBC improvement observed here is driven by interaction-mediated network strengthening rather than simple filler addition, also corresponds to the FTIR results. In addition, other researchers have pointed out that the hardness of oleogels is positively correlated with their OBC (Awlqadr et al., 2026; Shuai et al., 2023).

#### 3.2.6 Rheological properties

Additionally, this study examined the effect of MG content on the rheological properties (Kwon & Chang, 2022; Shuai et al., 2026) of RSOOs (Fig. 3).

Frequency sweep test results (Fig. 3E) showed that at 25 °C, in the frequency range of 0.1 to 25 Hz, the storage modulus (G′) of all oleogel samples was greater than the loss modulus (G′′), which was consistent with the characteristic of mainly exhibiting solid-like behavior. When the MG concentration increased from 3% to 12%, the G′ values all showed an upward trend; and they were always higher than the G′ values of oleogels with low concentrations of MG, which was similar to the previous XRD patterns. Similarly, G′′ values exhibited an upward trend as the MG concentration increased from 3% to 12%, remaining consistently higher than those of oleogels with lower MG concentrations. These results indicate that as the MG concentration increased, a stronger gel network structure was formed, attributed to a higher density of MG crystals forming a 3D network throughout the oil phase (Calligaris et al., 2018). This conclusion is consistent with the previously discussed results of hardness, oil binding capacity and polarized light microscopy.

In the temperature range of 5–85 °C, under the conditions of fixed frequency (1 Hz) and strain (0.01%), the changes of G′ and G′′ with temperature during heating were measured (Fig. 3F). As shown in the figure, G′ > G′′ in the solid state, while G′ and G′′ converged in the liquid region. During heating, both G′ and G′′ decreased sharply, indicating oleogel melting; the process concluded at approximately 55–60 °C, consistent with the DSC results. When the MG content was higher than 5%, the melting temperature of the oleogel (defined as the temperature where G′ = G′′) increased with the increase of MG concentration (Fig. 3G), which was consistent with the appearance observation and DSC results. These results indicate that the oleogel exhibits reversible thermogelation behavior: gelation occurs during cooling and melting occurs during heating. This temperature-responsive behavior provides potential application value as a plastic fat substitute and nutrient delivery carrier (Djaafar et al., 2025).

#### 3.2.7 Oxidative behavior and stability

Lipid oxidation is a principal cause of quality loss in oils rich in unsaturated fatty acids (Xiao et al., 2024). Here, oxidation was monitored by two complementary indicators: TBARS, which quantifies malondialdehyde (MDA), a secondary oxidation product (Ujihara et al., 2026), and the DPPH assay, which reports radical-scavenging capacity.

Fig. 3H shows the changes in TBARS values of RSOOs with different MG concentrations during accelerated oxidation storage: MDA, a lipid oxidation marker, increased over time, indicating that lipid oxidation was still in progress. Compared with pure rubber seed oil, the addition of MG reduced the growth rate of TBARS values. In addition, under the same conditions, these values gradually decreased with the increase of MG concentration. This decrease can be partially attributed to the replacement of some unsaturated fatty acids by MG, but the extent of these changes was much greater than a simple dilution effect. For example, after RSO was stored at 40 °C in the dark for 21 days, its TBARS value increased by about 7.21 times (from 268.60 to 1936.55 nmol/mL oil, *P* < 0.01). In contrast, in the oleogel containing 12% MG, this value only increased by 3.58 times (from 264.92 to 949.24 nmol/mL oil, *P* < 0.01). In parallel, DPPH radical-scavenging capacity declined over the storage period in all samples, consistent with progressive depletion of endogenous antioxidants (e.g., tocopherols) in RSO. Pure RSO exhibited the most rapid loss, decreasing from 86.79% at day 0 to 17.03% at day 21 (an 80.38% relative reduction). MG oleogels retained significantly more scavenging capacity: after 21 days, residual DPPH activity was 24.89 ± 3.24% (1% MG), 36.89 ± 2.82% (3% MG), 41.66 ± 4.15% (5% MG), 42.86 ± 1.25% (7% MG), 44.04 ± 1.85% (9% MG) and 42.99 ± 2.12% (12% MG).

Notably, DPPH scavenging at day 0 was lower in oleogels than in pure RSO and decreased with increasing MG concentration, which may be attributed to dilution of the antioxidant-rich RSO phase by the non-radical-scavenging MG rather than to any pro-oxidant effect. However, this differential oxidation rate is consistent with the framework established by recent work showing that fatty-acid unsaturation governs baseline oxidation susceptibility, while the crystalline gel network can partially offset this susceptibility by restricting oxygen diffusion and limiting the mobility of radical-propagating lipid species (Mahmoud Abou-Elsoud et al., 2026). The fact that all oleogel formulations - regardless of MG concentration - showed slower MDA accumulation than pure RSO supports the view that physical immobilization, rather than compositional change alone, contributes to the observed stability. The delay of RSO oxidation by MG may be due to the oil being immobilized in the crystal network, which reduces the migration of oxygen (Li et al., 2023), free radicals and lipid oxidation products from one position to another, thereby slowing down the oxidation process. Also, the effect of MG as an gelator on delaying the oxidation of RSO may also be due to the dilution effect of components. The total content of oxidizable polyunsaturated fatty acids was reduced by partially replacing unsaturated RSO with saturated monoglyceride.

As Fig. 3D shows, for long-term reference, the RSOOs' OBC after 3 months at 4 °C (12,000 r/min, 15 min) of 18.11% at 1% MG-RSOO and 92.94% at 12% MG-RSOO, this confirms that the MG 3D network has long-term oil holding stability.

### 3.3 Characterization of oleogel chocolate

Fig. 4A shows the steps of making rubber tree seed oil gel and oil gel based chocolate, and Fig. 4B shows the finished product of RSOO based chocolates prepared by the above steps (photographed at 20 °C).

**Fig. 4:**
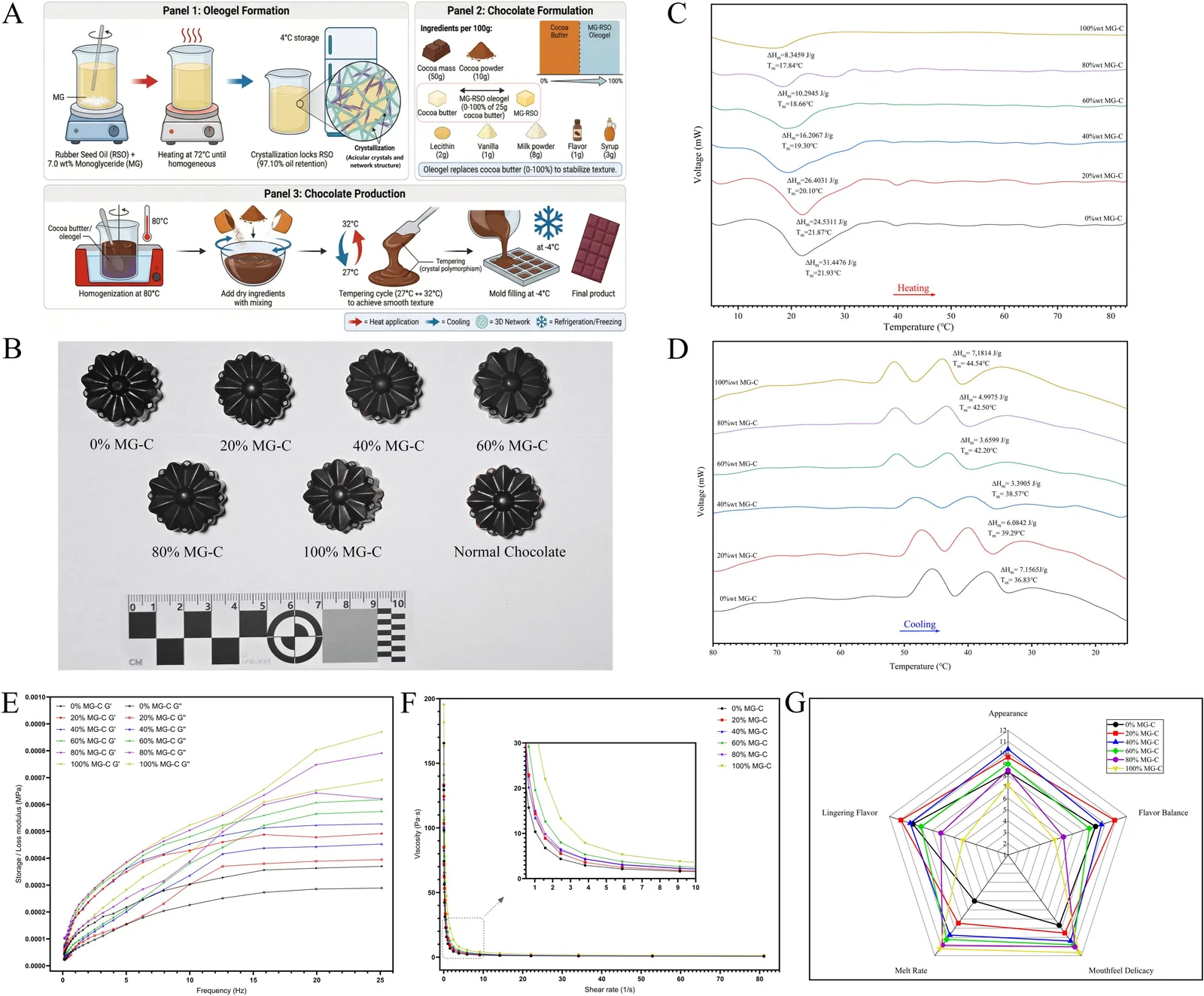
**A.** Diagram of the production process of RSOO chocolate; **B.** Image of RSOO Chocolate Finished Product (when CB is replaced with different RSOO proportions: 0%, 20%, 40%, 60%, 80% and 100%); **C–D.** Melting (**C.**) and cooling (**D.**) curves of DSC of RSOO chocolate when CB is replaced with different proportions (0%, 20%, 40%, 60%, 80% and 100%); ***E*-F.** Frequency sweep curves of storage modulus and loss modulus (**E.**), shear sweep curves (**F.**) of RSOO chocolates when CB is replaced with different proportions (0%, 20%, 40%, 60%, 80% and 100%); **G.** Radar chart of sensory evaluation scores of chocolates with different replacement ratios of CB by RSOO.

#### 3.3.1 Thermal behavior analysis of RSOO chocolates

The melting behavior of chocolate is critical for its functionality and taste (Li et al., 2023). Therefore, DSC was used to characterize the melting profiles (10–85 °C) and cooling profiles (80–15 °C) of the RSOO chocolates, respectively.

The research results indicate that chocolate should maintain a solid-like state at 20 to 30 °C, but should melt completely when the temperature reaches human body temperature to ensure good taste and flavor. Fig. 4C shows the DSC curves of chocolate during the heating process. On heating, the CB control (0% replacement) showed a single sharp melting peak at 21.93 °C with enthalpy 31.4476 J/g. With increasing RSOO replacement, the melting peak shifted to lower temperature and its enthalpy decreased progressively; at 100% replacement the melt peak occurred at 17.84 °C with enthalpy 23.1017 J/g. Thus partial CB replacement lowers the chocolate melting temperature, consistent with easier in-mouth melting.

On cooling, all chocolates showed solidification events whose onset shifted to higher temperature as RSOO content increased: the 0% control crystallized at 36.83 °C, whereas the 100% replacement crystallized at 44.54 °C. The enthalpy of solidification changed little overall but decreased after intermediate (40–60%) replacement. The earlier crystallization on cooling and the lower melting point on heating reflect the incorporation of the MG crystal network from the oleogel into the CB fat matrix.

#### 3.3.2 Thermal tolerance of RSOO chocolates

In addition, the thermal tolerance of RSOO Chocolates was also studied (Fig. 5).

**Fig. 5:**
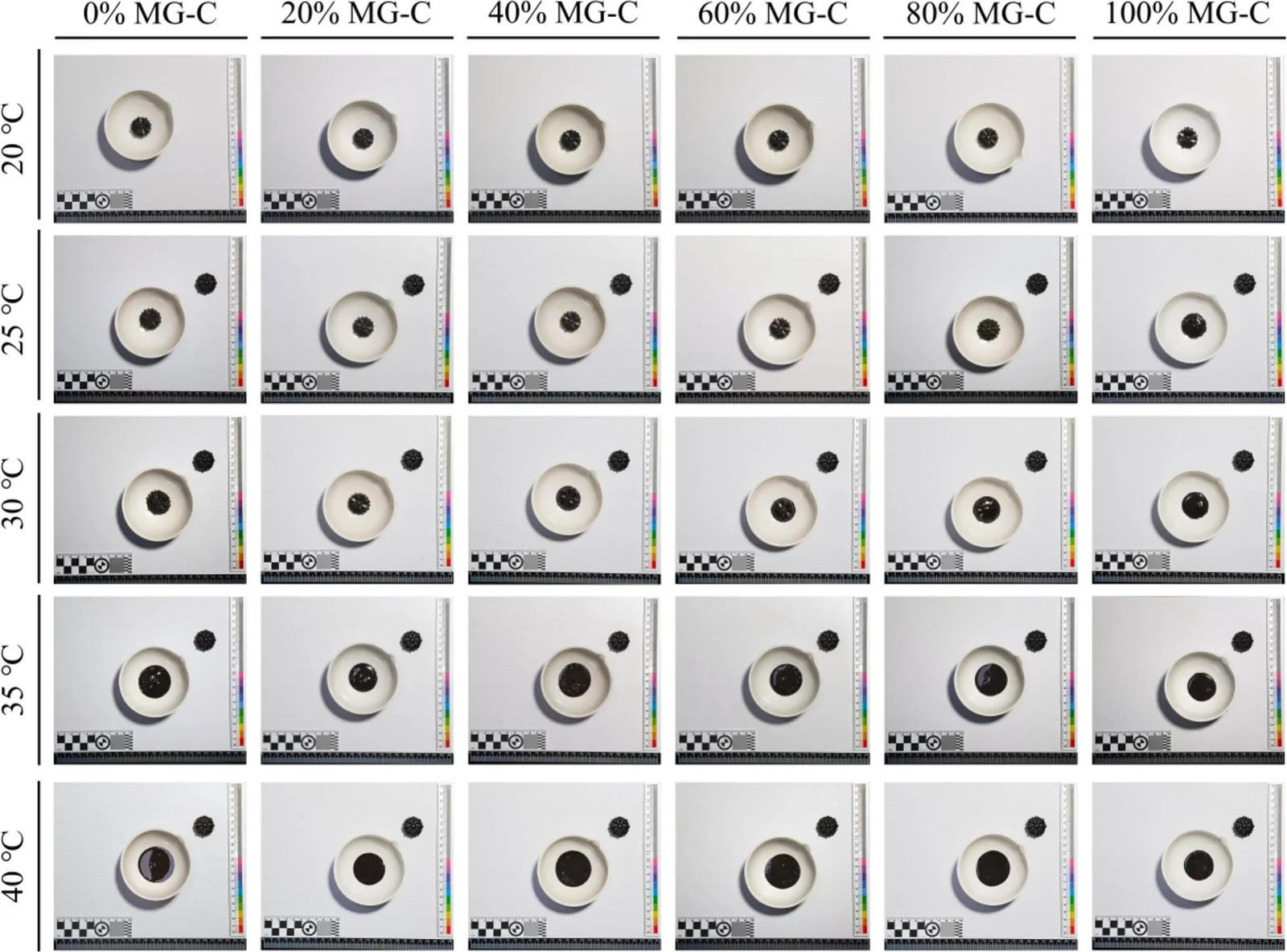
Thermal tolerance results of RSOO chocolates when CB is replaced with different proportions (0%, 20%, 40%, 60%, 80% and 100%).

The results showed that all chocolate samples softened at about 35 °C, while ordinary chocolate sample (0% MG-C) could still distinguish the original shape; All the chocolate has completely collapsed at 40 °C. Interestingly, chocolate samples containing more than 60% RSOO could not maintain its shape at a low temperature (30 °C), especially after RSOO completely replaced CB, the chocolate had collapsed at 25 °C.

#### 3.3.3 Rheological properties of RSOO chocolates

The rheological properties of chocolate play a crucial role in determining the quality of the final product (Chakraborty et al., 2026). Flow curves of chocolate samples, in which 0% to 100% of CB was replaced by RSOO (in 20% increments), were investigated at 40 °C.

The frequency sweep test results of the chocolate samples (Fig. 4E) showed that at 40 °C and in the frequency range of 0.1 to 25 Hz, the storage modulus (G′) of all chocolate samples was greater than the loss modulus (G′′), without any crossover point observed. This indicates that all chocolate samples exhibited solid-like elastic behavior. When the concentration of RSOO increased from 0% to 100%, the G′ values showed an upward trend, which was consistent with the previous XRD patterns of oleogels. Similarly, the G′′ values also increased with rising MG-RSO oleogel concentration from 0% to 100%, which may be attributed to the enhanced crystal network structure in chocolate resulting from oleogel replacement.

The shear rate sweep test results (Fig. 4F) demonstrated that the apparent viscosity of all chocolate samples gradually decreased with increasing shear rate (0–80 s^−1^), showing a typical non-Newtonian shear-thinning behavior. Furthermore, the apparent viscosity of chocolate samples increased with the increase of RSOO replacement ratio. This phenomenon is attributed to the fact that a higher proportion of oleogel in chocolate provides more solid crystals (at temperatures above the melting point of CB), thereby forming a stronger and denser 3D network structure that enhances flow resistance.

#### 3.3.4 Sensory evaluation of RSOO chocolates

Sensory evaluation results of the RSOO chocolate are shown in the Fig. 4G, it shows that the CB chocolate had a balanced taste but could not melt rapidly in the mouth; it was delicate and smooth when entering the mouth, and exhibited a strong cocoa aftertaste after swallowing. Compared with CB chocolate, the melting rate of RSOO chocolates was higher, consistent with the finding that the melting point of RSOO chocolates was lower than that of CB chocolate.

The scores of flavor balance of RSOO chocolates followed the order: 20% MG-C (10.89) > 40% MG-C (9.67) > 0% MG-C (9.12) > 60% MG-C (8.54) > 80% MG-C (6.14) > 100% MG-C (5.32). This indicates that the flavor of RSOO chocolates with partial replacement of CB was acceptable, but excessive oleogel would lead to the unique astringent taste of vegetable oil affecting the cocoa flavor, resulting in a decrease in its score (Yang et al., 2025).

Notably, the taste delicacy scores of RSOO chocolates ranged from 9.52 to 11.67, all higher than that of CB chocolate (8.67). This indicates that the RSOO chocolates melted instantly in the mouth with a smooth taste, which was consistent with the melting rate scores and corresponded to the previous DSC curve results of RSOO chocolates. However, with the increase of oleogel content, the melting time of chocolate in the mouth was shortened, and its aftertaste was also reduced (Morais Ferreira et al., 2017).

Based on the data in Fig. 5, when the degree of substitution exceeds 60%, it can lead to unacceptable thermal instability at room temperature (25–30 °C), while the optimal actual degree of substitution range is 20–60% to achieve a balance between nutrition, sensory experience, and storage stability.

In summary, the RSOO chocolates with 20–60% CBR showed better performance in all indicators than CB chocolate, offering testers a novel sensory experience characterized by instant melting in the mouth and a distinctive flavor, which is similar to sunflower oil oleogel chocolate (Ma et al., 2025).

## 4 Conclusions

In this study, the gelation of rubber seed oil was achieved by the addition of a relatively low concentration (5.0 wt%) of MG. The addition of MG formed a 3D aggregated crystal network, providing the oleogel with a stable solid structure, high oil binding capacity, good thermal reversibility, and strong oxidation resistance. Hydrogen bonds and van der Waals forces played key roles in the formation of these oleogels, and the RSOOs exhibited thermoreversible viscoelastic solid behavior.

Secondly, we demonstrated that RSOOs could partially replace CB in chocolate. This approach not only mitigates the issues associated with the high caloric value and saturated fatty acid content of CB but also addresses the high trans-unsaturated fatty acid content often found in hydrogenated oil substitutes, thereby ensuring the health-promoting qualities of the resulting chocolate. Furthermore, adding RSOO to chocolate has changed its melting behavior. When the substitution concentration is higher than 60%, chocolate has poor heat resistance under room temperature storage conditions (25–30 °C), which limits its application to directly replace all cocoa butter (CB) under conventional retail temperature conditions. Therefore, RSOO is more suitable for partially replacing CB (20–60%) to balance nutrition, taste, and thermal stability.

In this study, we only use the TBARS and DPPH indicator to evaluate the degree of oxidation. Also, a high RSOO substitution rate (>60%) can lead to poor thermal stability of chocolates. Therefore, we suggest to further explore the application of composite gelator (such as the mixture of multiple ogelator with different concentrations) in RSOO in the follow-up study. Finally, the results of this study provide some scientific guidance for the preparation of RSOOs and their application in improving chocolate quality.

## Patents

This research has produced a Chinese invention patent application (Publication No. CN120937961A, published on November 14, 2025): A rubber tree seed oil oleogel and its preparation method and application. The official publication document of the patent is provided as **S4**. The patent application covers the overall preparation framework of RSOO, and all quantitative experimental data, structural characterization, chocolate application results, and systematic mechanism discussions in this paper are original and unpublished data, which do not overlap with the protection scope of the patent claims. There is no intellectual property conflict.

## CRediT authorship contribution statement

**Zhi Huang:** Writing – review & editing, Writing – original draft, Methodology, Investigation, Data curation, Conceptualization. **Yaru Tang:** Visualization, Software, Methodology, Investigation. **Xinru Yang:** Formal analysis, Data curation. **Dongyan Liu:** Methodology, Investigation. **Yang Liu:** Formal analysis, Data curation. **Yangfan Ren:** Supervision, Funding acquisition. **Yun Wang:** Supervision, Funding acquisition. **Beibei Zhang:** Supervision. **Ji Ma:** Funding acquisition, Conceptualization.

## Informed consent statement

Not applicable.

## Institutional review board statement

Not applicable.

## Funding

This study was funded by Enterprise-University Cooperation Research Project: Kunming University of Science and Technology & Xishuangbanna Huakun Biotechnology Co., Ltd.'s horizontal project-Research on Rubber Seed Oil Processing Technology and Process (Grant No.: 202453103004805).

## Declaration of competing interest

The authors declare the following financial interests/personal relationships which may be considered as potential competing interests: Ji Ma reports financial support was provided by Kunming University of Science and Technology. Ji ma has patent pending to 202511211307.0. If there are other authors, they declare that they have no known competing financial interests or personal relationships that could have appeared to influence the work reported in this paper.

## Data Availability

Data will be made available on request.
