# Supplementary material for: Monoglyceride-structured rubber seed oil oleogels as a partial cocoa butter replacement in chocolate: physical properties, oxidative stability and applications
Source: Food Chem X. 2026 Oct 11;39:104594. doi: 10.1016/j.fochx.2026.104594 (PMC13683454; doi:10.1016/j.fochx.2026.104594)
Supplement: Supplementary file 1 [file mmc1.pdf]

Supplementary Material S1

Compilation of RSO Safety and Quality Evidence

**1**	Q/XHK 0001 S-2024 企业标准 食用橡胶树种子油<br>Enterprise Standard: Edible Rubber Seed Oil
**2**	Q/XSBNHK 0020-2015 企业标准 橡胶树种子油原油<br>Enterprise Standard: Crude Rubber Seed Oil
**3**	LS/T 3262-2019 粮食行业标准 食用橡胶籽油<br>Industry Standard: Edible Rubber Seed Oil
**4**	华测检验认证集团股份有限公司检验报告 A2240625007101001C（橡胶树种子油）<br>CTI (Centre Testing International) Test Report A2240625007101001C
**5**	SGS 检测报告 CDF25-0005759-01（橡胶树种子农药残留）<br>SGS Report CDF25-0005759-01: Pesticide Residues in Rubber Tree Seeds
**6**	SGS 检测报告 CDF25-0001880-01（橡胶树种子油农药残留）<br>SGS Report CDF25-0001880-01: Pesticide Residues in Rubber Seed Oil

These documents together support the statement in Section 2.1 on the safety and compliance of the rubber seed oil (RSO) used in this study, confirming conformity with relevant national, industry and enterprise standards, and the absence of cyanogenic glycosides and pesticide residues as verified by third-party testing (CTI, SGS).

Q/XHK

西双版纳华坤生物科技有限责任公司企业标准

Q/XHK 0001 S—2024

代替 Q/XHK 0001 S—2023

企业标准信息公共服务平台
公开 2025年08月26日 16点19分

食用橡胶树种子油

企业标准信息公共服务平台
公开 2025年08月26日 16点19分

2024－7-15 发布　　2024－7- 19 实施

西双版纳华坤生物科技有限责任公司 发布

前　言

我公司生产的食用橡胶树种子油是以橡胶树种子为原料，经过清洗、烘干、脱壳、色选、压榨、过滤、精练、包装等加工工艺制成。根据相关法律法制定本标准，作为企业组织生产、检验、贸易和仲裁的依据。

本标准安全性指标按照GB 2762-2022《食品安全国家标准 食品中污染物限量》、GB 2716-2018《食品安全国家标准 植物油》的规定制定，其中铅的含量指标严于食品安全国家标准，其余指标根据产品实际制定。在本标准中新增了总三萜的含量特征指标，在附录A中例出。

本标准代替Q/XHK 0001 S—2023《食用橡胶树种子油》。

本标准由西双版纳华坤生物科技有限责任公司提出、起草并解释。

本标准主要起草人：毕天飞、王云、刘佳、吴玉成、杨勇、李银源、左美蓉、王亮亮、付学磊。

企业标准信息公共服务平台
公开　2025年08月26日 16点19分
企业标准信息公共服务平台
公开　2025年08月26日 16点19分

食用橡胶树种子油

1 范围

本标准规定了食用橡胶树种子油的技术要求、检验规则、标志、包装、运输和贮存。

本标准适用于以橡胶树种子为主要原料，经过清洗、烘干、脱壳、色选、压榨、过滤、精练、包装等加工工艺制成的橡胶树种子油。

企业标准信息公共服务平台
公开
2025年08月26日 16点19分

2 规范性引用文件

本标准中所引用文件对于本文件的应用是必不可少的。凡是注日期的引用文件，仅所注日期的版本适用于本文件。凡是不注日期的引用文件，其最新版本（包括所有的修改单）适用于本文件。

3 产品分类

按产品质量等级分为：特级、优级、一级。

4 技术要求

4.1 原辅料要求

4.1.1 橡胶树种子：种子应饱满，种壳完整，外观无发芽、无霉变、无虫蛀，应符合相应的食品标准和有关规定。优级油选用原料为落地 7 天以内有生命力的种子；特级油选用原料为落地 7 天以内有生命力且为大树的种子。

4.1.2 其他原辅料：应符合相应的食品标准和有关规定，不得使用非食品用原料和辅料。

企业标准信息公共服务平台
公开
2025年08月26日 16点19分

4.2 感官要求

应符合表1的规定。

表 1 感官要求

项 目	要 求			检验方法
	特级	优级	一级	
外观	具有橡胶树种子油应有的色泽			GB/T 5009.37
气味、滋味	具有橡胶树种子油固有的浓郁坚果香味		无异味、口感良好	GB/T 5525
透明度	允许有少量沉淀物		澄清、透明	

4.3 特征指标

应符合表2的规定。

表 2　特征指标

项　目	指　标			检验方法
	特级	优级	一级	
折光指数，n^{40}	1.467～1.471			GB/T 5527
相对密度，d_{20}^{20}	0.900～0.926			GB/T 5526
碘值（以 I 计），g/100g	120～145			GB/T 5532
皂化值（以 KOH 计），mg /g	175～197			GB/T 5534
不皂化物，g/kg　≤	20			GB/T 5535.1、GB/T 5535.2
脂肪酸组成，%				GB 5009.168
棕榈酸　C16:0	7.0～12.0			
硬脂酸　C18:0	6.0～10.0			
油酸　C18:1	20.0～30.0			
亚油酸　C18:2	36.0～42.0	30.0～42.0		
亚麻酸　C18:3	18.0～21.0	14.0～30.0		

4.4　理化指标

应符合表3的规定。

表 3　理化指标

项　目	指　标			检验方法
	特级	优级	一级	
水分及挥发物，g/100g　≤	0.2			GB 5009.236
酸价（KOH），mg/g　≤	3.0			GB 5009.229
不溶性杂质，g/100g　≤	0.1			GB/T 15688
过氧化值，g/100g　≤	0.25			GB 5009.227

4.5　污染物限量

应符合GB 2762 、 GB 2716的规定；严于食品安全国家标准的指标应符合表4的规定。

表 4　污染物限量

项　目	指　标			检验方法
	特级	优级	一级	
总 砷（以 As 计），mg/kg　≤	0.08			GB 5009.11
铅（以 Pb 计），mg/kg　≤	0.06			GB 5009.12

4.6　净含量

应符合《定量包装商品计量监督管理办法》的规定，按JJF1070规定的方法检验。

4.7　食品添加剂

食品添加剂的使用应符合GB 2760的规定。

4.8　生产加工过程卫生要求

企业标准信息公共服务平台 公开 2025年08月26日 16点19分

应符合GB 8955的规定。

5 检验规则

5.1 批组

以同一批原料，同一工艺、同设备加工生产的同一产品为一批。

5.2 抽样

所抽样品须为同一批次保质期内的产品，抽样基数不得少于50桶（瓶），总量不少于80kg。每批随机抽取2个包装桶（瓶），总量不少于2kg，样品分成2份，1份检验，1份备查。

5.3 出厂检验

每批产品出厂前，必须检验部门检验合格后方可出厂。出厂检验项目按相关规定和要求执行。

5.4 型式检验

正常情况下，型式检验每年进行一次，检验项目为本标准规定的全部项目。有下列情况之一时，亦要进行型式检验：

a) 原料、生产工艺、生产设备发生较大变化时；
b) 停产半年以上重新恢复生产时；
c) 出厂检验结果与上次型式检验结果有较大差异时；
d) 国家安全监管部门提出型式检验要求时。

5.5 判定规则

该产品检验结果若有不合格项时，允许用留样进行复检，以复检结果为准。

6 标志、包装、运输、贮存

6.1 标志

6.1.1 产品标识应该符合 GB 7718、GB 2716 和 GB 28050 的规定。
6.1.2 包装储运图示标志应符合 GB/T 191 的规定。

6.2 包装

包装材料和容器应该符合相应的食品安全标准和有关规定，封口严密，包装牢固。

6.3 运输

运输工具应洁净、干燥、卫生、产品不得与有毒、有害、有腐蚀性、易挥发或有异味的物品混装运输。运输时应防止日晒雨淋，装卸时应轻搬、轻放，不得抛掷，重压和挤压。

6.4 贮存

产品应离墙离地贮存在避光、阴凉通风、干燥的室内，并有防尘、防蝇、防虫、防鼠设施。不得与有毒、有害、易污染的物品混贮。仓库内的产品，按不同品种和等级分类堆码整齐。

企业标准信息公共服务平台 公开 2025年08月26日 16点19分
企业标准信息公共服务平台 公开 2025年08月26日 16点19分

附　录　A
（规范性附录）
总三萜含量划分

项　目	指　标			检验方法
	特级	优级	一级	
总三萜，g/100g　≥	10	-	-	保健食品理化及卫生指标检验与评价技术指导原则（2020 年版）第二部分

企业标准信息公共服务平台
公开　2025年08月26日 16点19分
企业标准信息公共服务平台
公开　2025年08月26日 16点19分

ICS 67.200.10

33

Q/XSBNHK

西双版纳华坤生物科技有限责任公司企业标准

Q/XSBNHK 0020—2015

企业标准信息公共服务平台
备案
2017年03月21日 17点49分

橡胶树种子油原油

Crude rubber seed oil

企业标准信息公共服务平台
备案
2017年03月21日 17点49分

2015－8－20 发布　　　　2015－11－20 实施

西双版纳华坤生物科技有限责任公司

发 布

目　　次

前言 II
1　范围 1
2　规范性引用文件 1
3　术语和定义 1
4　分类 3
5　质量要求及检验方法 3
6　检验规则 4
7　标签标识 4
8　包装、运输和储存 5

企业标准信息公共服务平台
备案
2017年03月21日 17点49分

企业标准信息公共服务平台
备案
2017年03月21日 17点49分

前　　言

本标准按照GB/T 1.1给出的规则起草。

本标准由西双版纳华坤生物科技有限责任公司提出并归口。

本标准起草单位：西双版纳华坤生物科技有限责任公司。

本标准主要起草人：朱义鑫、张娜娜、李爱萍、王云

企业标准信息公共服务平台
备案
2017年03月21日 17点49分

企业标准信息公共服务平台
备案
2017年03月21日 17点49分

橡胶树种子油原油

1 范围

本标准规定了橡胶树种子油原油的术语和定义、分类、质量要求及检验方法、检验规则、标签标识、包装、运输和储存等要求。

本标准适用于橡胶树种子油原油，包括压榨橡胶树种子油原油和浸出橡胶树种子油原油。

橡胶树种子油原油的质量指标仅适用于橡胶树种子油原油贸易。

企业标准信息公共服务平台 备案 2017年03月21日 17点49分

2 规范性引用文件

下列文件对于本文件的应用是必不可少的。凡是注日期的引用文件，仅所注日期的版本适用于本文件。凡是不注日期的引用文件，其最新版本（包括所有的修改单）适用于本文件。（模版生成）

下列标准中的条款通过本标准的引用而成为本标准的条款，凡是注日期的引用文件，其随后所有的修改单（不包括勘误的内容）或修订版均不适用于本标准，然而鼓励根据本标准达成协议的各方研究是否可使用这些文件的最新版本。凡是不注日期的引用文件，其最新版本适用与本标准。（原标准）

GB/T 191 包装储运图示标志

GB 2716 食用植物油卫生标准

GB 2760 食用添加剂使用卫生标准

GB/T 5009.37 食用植物油卫生标准的分析方法

GB/T 5490 粮食、油料及植物油脂检验一般原则

GB/T 5524 食用油脂检验扦样、分样法

GB/T 5525 植物油脂检验透明度、色泽、气味、滋味鉴定法

GB/T 5526 植物油脂检验比重测定法

GB/T 5527 动植物油脂折光指数的测定

GB/T 5528 动植物油脂水分及挥发物含量测定法

GB/T 5529 植物油脂检验杂质测定法

GB/T 5530 动物油脂检验酸值和酸度测定

GB/T 5534 动植物油脂皂化值的测定

GB/T 5535 动植物油脂不皂化物测定

GB/T 5538 动植物油脂过氧化值测定

GB 7718 食品标签通用标准

GB/T 17374 食用植物油销售包装

GB/T 17375 动植物油脂 灰分测定

GB/T 17376 动植物油脂脂肪酸甲脂制备

GB/T 17377 动植物油脂脂肪酸甲脂的气象色谱分析

企业标准信息公共服务平台 备案 2017年03月21日 17点49分

3 术语和定义

下列术语和定义适用于本标准。

3.1

橡胶树种子油 rubber seed oil

以巴西三叶橡胶树（*Hevea brasiliensis*）种子为原料制取的植物油。

3.2

橡胶树种子油原油 crude rubber seed oil

未经任何处理的不能直接供人类食用的橡胶树种子油。

3.3

成品压榨橡胶树种子油 finished pressing rubber seed oil

橡胶树种子经过压榨制取的符合标准成品质量指标和卫生要求的直接供人类食用的橡胶树种子油。

3.4

成品浸出橡胶树种子油 finished solvent extraction rubber seed oil

橡胶树种子经过浸出工艺制取的符合标准成品质量指标和卫生要求的直接供人类食用的橡胶树种子油。

3.5

折光指数 refractive index

光线从空气中射入油脂时，入射角与折射角的正弦之比值。

3.6

相对密度 specific gravity

20℃植物油的质量与同体积20℃蒸馏水的质量之比值。

3.7

碘值 iodine value

在规定条件下与100g油脂发生加成反应所需碘的克数。

3.8

皂化值 saponification value

皂化1g油脂所需的氢氧化钾毫克数。

3.9

不皂化物 unsaponifiable matter

油脂中不与碱起作用、溶于醚、不溶于水的物质，包括甾醇、脂溶性维生素和色素等。

3.10

脂肪酸 fatty acid

脂肪酸一元羧酸的总称，通式为R-COOH。

3.11

水分及挥发物 moisture and volatile matter

在一定温度条件下，油脂中所含的微量水分和挥发物

3.12

不溶性杂质 insoluble impurities

油脂中不溶于石油醚等有机溶剂的物质。

3.13

酸值 acid value

中和1g油脂中所含游离脂肪酸需要的氢氧化钾毫克数。

3.14

过氧化值 peioxide value

企业标准信息公共服务平台 备案 2017年03月21日 17点49分
企业标准信息公共服务平台 备案 2017年03月21日 17点49分

1kg 油脂中过氧化物的毫摩尔数。

3.15

溶剂残留量 residual solvent content in oil

1kg 油脂中残留的溶剂毫克数。

4 分类

橡胶树种子油分为：橡胶树种子油原油，成品压榨橡胶树种子油，成品浸出橡胶树种子油三类。

5 质量要求及检验方法

5.1 特征指标及检验方法

表1 橡胶树种子油原油特征指标及检验方法

项 目		特征指标	检验方法
折光指数（n^{40}）:		1.467-1.471	按照 GB/T 5527 规定的方法执行
相对密度（[illegible]）:		0.919-0.926	按照 GB/T 5526 规定的方法执行
碘值(I)/(g/100g):		130-145	按照 GB/T 5532 规定的方法执行
皂化值(KOH)/(mg/g):		175-185	按照 GB/T 5534 规定的方法执行
不皂化物/(g/kg):		≤20	按照 GB/T 5535 规定的方法执行
脂肪酸组成/(%):	豆蔻酸 $C_{14:0}$	ND-0.5	按照 GB/T 17375-17377 规定的方法执行
	棕榈酸 $C_{16:0}$	8.0-11.0	
	棕榈油酸 $C_{16:1}$	ND-0.7	
	硬脂酸 $C_{18:0}$	7.0-9.5	
	油酸 $C_{18:1}$	22.0-28.0	
	亚油酸 $C_{18:2}$	33.0-41.0	
	亚麻酸 $C_{18:3}$	15.0-22.0	
	花生酸 $C_{20:0}$	ND-1.5	
	花生一烯酸 $C_{20:1}$	ND-0.7	
	其它	ND-0.8	

注 1：ND 表示未检出，定义为 0.05%。

5.2 质量等级指标及检验方法

表2 橡胶树种子油原油质量指标及检验方法

项 目		质量指标	检验方法
气味滋味		具有橡胶树种子油特有的气味和滋味，无异味	按照 GB/T 5525 规定的方法执行
水分及挥发物/(%)	≤	0.2	按照 GB/T 5528 规定的方法执行
不溶性杂质/(%)	≤	0.2	按照 GB/T 5529 规定的方法执行

企业标准信息公共服务平台 备案 2017年03月21日 17点49分

**酸值(KOH)/(mg/g)　　≤**	**30**	按照 GB/T 5530 规定的方法执行

表 2　续

项　目	质量指标	检验方法
**过氧化值/(mmol/kg)　　≤**	**7.5**	按照 GB/T5538 规定的方法执行
**溶剂残留量/(mg/kg)　　≤**	**100**	按照 GB/T 5009.37 规定的方法执行
注：黑体部分指标强制		

5.3　卫生指标及及检验方法

按照GB2716、GB2760、GB/T 5009.37和国家有关规定执行。

5.4　其它

橡胶树种子油原油不得直接用于食用油，必须经过精炼符合标准后才可食用。

6　检验规则

6.1　扦样、分样

橡胶树种子油抽样方法按照 GB/T 5524 的要求执行。

6.2　产品组批

以同一班次、同一次投料、同一工艺、同一生产线生产的同一品种、同一规格的产品为一批。

6.3　出厂检验

每批产品必须经公司质量检验部门按照本标准的规定进行检查，检查合格并出具合格证后方能出厂。出厂检验按照技术要求规定，逐批检查。

6.4　型式检查

每半年进行一次，型式检查项目为本标准规定的所有项目，发生下列情况之一时亦应进行检验。

1)　更改主要原辅料或更改关键工艺；
2)　停产半年以上，再恢复生产时；
3)　出产检验结果与上一次检验结果差异较大时；
4)　国家质量监督机构提出型式检查要求时。

6.5　判定规则

产品的卫生指标和各等级指标中有一项不合格，则判该产品不合格。其余指标如有不符合项，可以从该产品中加倍取样进行复检，以复检结果为准。

7　标签标识

7.1　预销售包装的标签除了符合 GB 7718 的规定以及要求之外，还应标注产品的加工方式，如“压榨’’、“浸出”等字样。

企业标准信息公共服务平台
备案
2017年03月21日 17点49分
企业标准信息公共服务平台
备案
2017年03月21日 17点49分

7.2 非零售包装应注明产品的名称、标准号、原料、生产日期、保质期、生产单位名称、地址、电话等。

7.3 应注明产品原料原产国。

8 包装、运输和储存

8.1 包装

应符合 GB/T 17374 及国家的有关规定和要求，外包装储运图示标识应符合 GB/T 191 规定要求。

8.2 运输

运输中应注意安全，防止日晒、雨淋、渗漏、污染和标签脱落。散装运输有专车，保持车辆清洁、卫生，不得与其他有毒有害的物质混装混运。装运时要轻拿、轻放、轻装、倾卸，防止重压。

8.3 储存

成品要保持清洁卫生、应贮存于阴凉、干燥及避光处。不得与有害、有毒物品或食用植物油一同存放。

企业标准信息公共服务平台
备案
2017年03月21日 17点49分

企业标准信息公共服务平台
备案
2017年03月21日 17点49分

ICS 67.200.10
X 14

LS

中华人民共和国粮食行业标准

LS/T 3262—2019

食用橡胶籽油

Edible rubber seed oil

2019-06-06 发布

2019-12-06 实施

国家粮食和物资储备局　　发布

中 华 人 民 共 和 国 粮 食
行 业 标 准
食用橡胶籽油
LS/T 3262—2019
*
中 国 标 准 出 版 社 出 版 发 行
北京市朝阳区和平里西街甲 2 号(100029)
北京市西城区三里河北街 16 号(100045)
网址 www.spc.net.cn
总编室:(010)68533533 发行中心:(010)51780238
读者服务部:(010)68523946
中国标准出版社秦皇岛印刷厂印刷
各地新华书店经销
*
开本 880×1230 1/16 印张 0.5 字数 10 千字
2019 年 7 月第一版 2019 年 7 月第一次印刷
*
书号:155066・2-34435 定价 14.00 元

如有印装差错 由本社发行中心调换
版权专有 侵权必究
举报电话:(010)68510107

前　言

本标准按照 GB/T 1.1—2009 给出的规则起草。

本标准由国家粮食和物资储备局提出。

本标准由全国粮油标准化技术委员会(SAC/TC 270)归口。

本标准起草单位:海南省粮油科学研究所、武汉轻工大学、国家粮食和物资储备局标准质量中心、海南侯臣生物科技有限公司、西双版纳华坤生物科技有限责任公司、云南摩尔农庄生物科技开发有限公司。

本标准主要起草人:郑联合、何东平、杨卫民、谭家安、王涛、张世宏、陈艳、赵阔、张红建、王云、朱义鑫、张跃进。

食用橡胶籽油

1 范围

本标准规定了食用橡胶籽油的术语和定义、分类、质量要求、检验方法、检验规则、标签标识以及包装、储存、运输和销售的要求。

本标准适用于食用橡胶籽原油和成品食用橡胶籽油。

食用橡胶籽原油的质量指标仅适用于原油的贸易。

2 规范性引用文件

下列文件对于本文件的应用是必不可少的。凡是注日期的引用文件，仅注日期的版本适用于本文件。凡是不注日期的引用文件，其最新版本(包括所有的修改单)适用于本文件。

GB/T 191 包装储运图示标志

GB/T 5009.37 食用植物油卫生标准的分析方法

GB/T 5009.168 食品安全国家标准 食品中脂肪酸的测定

GB 5009.229 食品安全国家标准 食品中酸价的测定

GB 5009.236 食品安全国家标准 动植物油脂水分及挥发物的测定

GB/T 5490 粮油检验 一般规则

GB/T 5524 动植物油脂 扦样

GB/T 5525 植物油脂 透明度、气味、滋味鉴定法

GB/T 5526 植物油脂检验 比重测定法

GB/T 5527 动植物油脂 折光指数的测定

GB/T 5532 动植物油脂 碘值的测定

GB/T 5534 动植物油脂 皂化值的测定

GB 7718 食品安全国家标准 预包装食品标签通则

GB/T 15688 动植物油脂 不溶性杂质含量的测定

GB/T 17374 食用植物油销售包装

GB 28050 食品安全国家标准 预包装食品营养标签通则

GB/T 30354 食用植物油散装运输规范

3 术语和定义

下列术语和定义适用于本文件。

3.1

食用橡胶籽油 edible rubber seed oil

又称食用橡胶树种子油，采用橡胶籽为原料加工制成的油脂。

3.2

食用橡胶籽原油 crude edible rubber seed oil

橡胶籽经压榨、浸出法制取的未经精炼处理，不能直接食用的橡胶籽油。

3.3

成品食用橡胶籽油　finished product of edible rubber seed oil

由食用橡胶籽原油精炼制成，可供人类食用的油脂。

4　分类

食用橡胶籽油分为食用橡胶籽原油和成品食用橡胶籽油两类。

5　质量要求

5.1　基本组成和主要物理参数

食用橡胶籽油基本组成和主要物理参数见表 1。

表 1　食用橡胶籽油基本组成和主要物理参数

<table>
<tr><th colspan="2">项目</th><th>指标</th></tr>
<tr><td colspan="2">折光指数(n^{40})</td><td>1.467～1.472</td></tr>
<tr><td colspan="2">相对密度(d_{20}^{20})</td><td>0.919～0.926</td></tr>
<tr><td colspan="2">碘值(I_2)/(g/100 g)</td><td>120～145</td></tr>
<tr><td colspan="2">皂化值(KOH)/(mg/g)</td><td>175～197</td></tr>
<tr><td rowspan="5">主要脂肪酸组成/%</td><td>棕榈酸(C16:0)</td><td>7.8～10.6</td></tr>
<tr><td>硬脂酸(C18:0)</td><td>6.5～9.3</td></tr>
<tr><td>油酸(C18:1)</td><td>16.2～28.7</td></tr>
<tr><td>亚油酸(C18:2)</td><td>33.6～42.9</td></tr>
<tr><td>亚麻酸(C18:3)</td><td>14.0～25.0</td></tr>
</table>

5.2　质量指标

5.2.1　食用橡胶籽原油质量指标

食用橡胶籽原油质量指标见表 2。

表 2　食用橡胶籽原油质量指标

项目	质量指标
气味、滋味	具有食用橡胶籽原油固有的气味和滋味，无异味
水分及挥发物含量/%　　≤	0.50
不溶性杂质含量/%　　≤	0.50

5.2.2　成品食用橡胶籽油质量指标

成品食用橡胶籽油质量指标见表 3。

表 3 成品食用橡胶籽油质量指标

项目		质量指标	
		一级	二级
气味、滋味		具有食用橡胶籽油固有的气味和滋味，无异味	
色泽		淡黄色至棕黄色	
透明度(20 ℃)		透明	允许微浊
水分及挥发物含量/%	≤	0.20	
不溶性杂质含量/%	≤	0.05	
酸价(KOH)/(mg/g)	≤	1.0	3.0

5.3 食品安全要求

按食品安全国家标准和法律法规有关规定执行。

6 检验方法

6.1 透明度、气味、滋味检验：按照 GB/T 5525 执行。
6.2 色泽检验：按照 GB/T 5009.37 执行。
6.3 相对密度检验：按照 GB/T 5526 执行。
6.4 折光指数检验：按照 GB/T 5527 执行。
6.5 水分及挥发物含量检验：按照 GB 5009.236 执行。
6.6 不溶性杂质含量检验：按照 GB/T 15688 执行。
6.7 酸价检验：按照 GB 5009.229 执行。
6.8 碘值检验：按照 GB/T 5532 执行。
6.9 皂化值检验：按照 GB/T 5534 执行。
6.10 脂肪酸组成检验：按照 GB 5009.168 执行。

7 检验规则

7.1 检验一般规则

按照 GB/T 5490 的要求执行。

7.2 扦样

按照 GB/T 5524 的要求执行。

7.3 出厂检验

7.3.1 应逐批检验，并出具检验报告。
7.3.2 按 5.2 规定的项目检验。

7.4 型式检验

7.4.1 当原料、设备、工艺有较大变化或监督部门提出要求时，均应进行型式检验。

7.4.2 按本标准第5章的规定检验。当检测结果与表1的规定不符合时，可用生产该批产品的橡胶籽原料进行检验，并佐证。

7.5 判定规则

7.5.1 产品未标明质量等级时，按不合格判定。

7.5.2 产品经检验，有一项不符合表2、表3规定值时，判定为不符合该等级的产品。

8 标签标识

8.1 预包装产品应符合 GB 7718、GB 28050 要求。

8.2 非预包装产品根据分类标注产品名称。

9 包装、储存、运输和销售

9.1 包装

9.1.1 应符合 GB/T 17374 及国家的有关规定和要求。

9.1.2 包装储运图示标志应符合 GB/T 191 的规定。

9.2 储存

应储存于卫生、阴凉、干燥及避光处，不得与有害、有毒物品一同存放，尤其要避开有异常气味的物品。

9.3 运输

运输中应注意安全，防止日晒、雨淋、渗漏、污染和标签脱落。散装运输符合 GB/T 30354 的要求。

9.4 销售

预包装的成品食用橡胶籽油在零售终端不得脱离原包装散装销售。

LS/T 3262-2019

版权专有 侵权必究

*

书号：155066・2-34435

定价： 14.00 元

报告编号： A2240625007101001C

检 验 报 告

Test Report

样 品 名 称 ： 橡胶树种子油

委 托 单 位 ： 西双版纳华坤生物科技有限责任公司

检 验 类 型 ： 委托检验

云南华测检测认证有限公司

Centre Testing International（Yunnan）Co., Ltd

www.cti-cert.com

验证码：O04X

检验报告

样品信息	样品名称	橡胶树种子油	CTI 样品编号	KQ13138001
	商标	/	型号/规格/等级	/
	生产日期	/	批号	/
	样品数量	1.5L(3 瓶)	样品状态	液体
	生产商	西双版纳华坤生物科技有限责任公司		
	生产商地址	云南省西双版纳州景洪市嘎洒镇曼迈村委会曼景栋村		
客户信息	委托单位	西双版纳华坤生物科技有限责任公司		
	委托单位地址	云南省西双版纳州景洪市嘎洒镇曼迈村委会曼景栋村		
检验信息	样品接收日期	2024 年 10 月 12 日	样品检验日期	2024 年 10 月 12 日 ~ 2024 年 10 月 23 日
	检验项目	气味、滋味,外观,不溶性杂质,不皂化物,碘值（以 I 计）等 18 项。		
	检验依据	GB 2761-2017《食品安全国家标准 食品中真菌毒素限量》,GB 2762-2022《食品安全国家标准 食品中污染物限量》,Q/XHK 0001 S-2024 《食用橡胶树种子油》。		
检验结论	经检测，第 1 项至第 17 项符合 GB 2761-2017《食品安全国家标准 食品中真菌毒素限量》，GB 2762-2022《食品安全国家标准 食品中污染物限量》，Q/XHK 0001 S-2024 《食用橡胶树种子油》要求，第 18 项仅提供检测数据。<br>（检验检测专用章）<br>批准日期：2024 年 10 月 23 日			
备注	-----			

云南华测检测认证有限公司 检验检测专用章

主检： 审核： 批准：

授权签字人：李艳梅

CTI 华测检测

检验报告

检验结果：

序号	检验项目	单位	检验结果	标准要求	结论	检验方法
1	气味、滋味	/	无异味、口感良好	无异味、口感良好	符合	GB/T 5525-2008 6
2	外观	/	黄色油状液体	浅黄至黄色油状液体	符合	GB/T 5009.37-2003 3.1
3	不溶性杂质	g/100g	0.06	≤0.1	符合	GB/T 15688-2008
4	不皂化物[#1]	g/kg	5.90	≤20	符合	GB/T 5535.1-2008
5	碘值（以 I 计）[#1]	g/100g	144	120 ~ 145	符合	GB/T 5532-2022
6	过氧化值	g/100g	0.051	≤0.25	符合	GB 5009.227-2023 第一法
7	水分及挥发物	g/100g	0.08	≤0.2	符合	GB 5009.236-2016 第二法
8	酸价(KOH)	mg/g	2.1	≤3.0	符合	GB 5009.229-2016 第二法
9	透明度	/	澄清、透明	澄清、透明	符合	GB/T 5525-2008 5
10	相对密度(d_{20}^{20})	/	0.9215	0.900 ~ 0.926	符合	GB/T 5526-1985 2
11	皂化值（以 KOH 计）[#1]	mg/g	195	175 ~ 197	符合	GB/T 5534-2008
12	折光指数(n^{40})	/	1.4682	1.467 ~ 1.471	符合	GB/T 5527-2010
13	铅（以 Pb 计）	mg/kg	未检出(定量限：0.05mg/kg)	≤0.06	符合	GB 5009.12-2023 第二法
14	总砷(以 As 计)	mg/kg	未检出(定量限：0.005mg/kg)	≤0.08	符合	GB 5009.11-2024 第一篇 第二法
15	黄曲霉毒素 B_1	μg/kg	4.04	≤10	符合	GB 5009.22-2016 第一法
16	苯并[a]芘	μg/kg	未检出(定量限：0.5μg/kg)	≤10	符合	GB 5009.27-2016
17	脂肪酸组成					
	亚麻酸(C18:3)	%	21.5	14.0 ~ 30.0	符合	GB 5009.168-2016 第三法
	亚油酸(C18:2)	%	37.7	30.0 ~ 42.0	符合	GB 5009.168-2016 第三法
	硬脂酸(C18:0)	%	7.06	6.0 ~ 10.0	符合	GB 5009.168-2016 第三法

检验报告

报告编号：A2240625007101001C

检验结果：

序号	检验项目	单位	检验结果	标准要求	结论	检验方法
	油酸(C18:1)	%	24.2	20.0 ~ 30.0	符合	GB 5009.168-2016 第三法
	棕榈酸(C16:0)	%	8.69	7.0 ~ 12.0	符合	GB 5009.168-2016 第三法
18	甾醇总量[*1#1]	mg/100g	290	/	/	GB/T 25223-2010
以下空白						

备注：

1. *1 表示该项目/方法不在 CMA 资质认定范围内。
2. #1 此项目由青岛市华测检测技术有限公司完成，CMA 资质证书编号 241520340452。
3. Q/XHK 0001 S-2024 《食用橡胶树种子油》由委托单位提供。

声明：

1.报告无批准人签字、检验检测专用章及报告骑缝章，或经涂改，以及复印报告未加盖红色检验检测专用章均视作无效。

2.未经本公司批准，不得部分复制本报告。

3.样品信息由客户提供，本报告检测结果仅对受检样品负责。

4.不得擅自使用检测结果进行不当宣传。

5.如果对检测结果有异议，请于收到报告之日起 7 个工作日内提出，逾期不予受理。

6.扫描报告首页二维码，或登陆官方网站 https://mycti.cti-cert.com 输入报告编号和报告首页验证码，即可查询报告真伪；如有疑问，请联系邮箱：fdd.checkreport@cti-cert.com。

*** 报告结束 ***

中国认可
国际互认
检测
TESTING
CNAS L10479

检测报告

CDF25-0005759-01 发布日期：2025-10-22

客户名称： 西双版纳华坤生物科技有限责任公司

客户地址： 景洪市嘎洒镇曼迈村委会曼景栋村

样品名称： 橡胶树种子

样品批号： /

生产日期： /

生产商： /

以上样品及信息由客户提供及确认, SGS 不承担证实客户提供信息的准确性、适当性和(或)完整性责任。

样品接收日期： 2025-10-16

检测周期： 2025-10-16 ~ 2025-10-22

检测要求： 根据客户要求检测

检测方法： 请参见下一页。

检测结果： 请参见下一页。

检验检测专用章
Inspection & Testing Services
通标标准技术服务有限公司四川分公司
SGS-CSTC Standards Technical Services Co., Ltd. Sichuan Branch

SGS 授权签字人

扫码查看在线报告
CDF25-0005759-01
报告验真请访问：
check.sgsonline.com.cn

通标标准技术服务有限公司四川分公司

除非另有书面协议，本报告由本公司依据《 服务通用条款 》出具。
《 服务通用条款 》印刷在正本报告纸背面，或通过 https://www.sgs.com/en/Terms-and-Conditions 查询。请特别关注其中涉及责任限定，赔偿以及司法管辖的相关条款。任何报告的持有方需知悉，此报告内容仅反映SGS受限于客户指示下，且在当时所得结论。SGS仅对其客户负责，并且此报告不能免除交易各方根据交易文件所享有的权利和应履行的义务。未获得本公司书面批准，本文件不得进行复制，全文除外。任何未经授权对报告内容及形式进行的修改、伪造或扭曲都是违法行为，违法者将会被追究法律责任。除非另有声明，本测试报告所示结果仅涉及受测试样品。
注意：检测/检验报告或证书的真实性，请通过电话 (86 –755) 83071443 或邮箱 CN.Doccheck@sgs.com 查询。

No.1, 5/F., Building D1, No. 1666, Section 2 of Chenglong Road, Economic and Technological Development Zone, Longquanyi District, Chengdu, Sichuan, China 610100 t (86–28) 8481 0338 www.sgsgroup.com.cn
中国・四川・成都市龙泉驿区经济技术开发区成龙大道二段1666号 D1栋05层1号 邮编：610100 t (86–28) 8481 0338 sgs.china@sgs.com

Member of the SGS Group (SGS SA)

检测报告

CDF25-0005759-01　　　　发布日期：2025-10-22

检测样品描述：

样品编号	SGS 样品 ID	样品描述
1	CDF25-0005759-0001	袋装样品　1 袋

检测结果：

农药残留

检测方法：　**BS EN 15662:2018 HPLC-MS/MS**

编号	检测项目	单位	CAS No.	检测结果 1	定量限
1	#混杀威（3,4,5-混杀威）	mg/kg	2686-99-9	ND	0.01
2	阿维菌素	mg/kg	71751-41-2	ND	0.01
3	乙酰甲胺磷	mg/kg	30560-19-1	ND	0.01
4	啶虫脒	mg/kg	135410-20-7	ND	0.01
5	#活化酯/苯并噻二唑	mg/kg	135158-54-2	ND	0.01
6	涕灭威	mg/kg	116-06-3	ND	0.01
7	#砜灭威（涕灭威砜）	mg/kg	1646-88-4	ND	0.01
8	#涕灭威亚砜	mg/kg	1646-87-3	ND	0.01
9	#酰嘧磺隆	mg/kg	120923-37-7	ND	0.01
10	#吲唑磺菌胺	mg/kg	348635-87-0	ND	0.01
11	#莎稗磷	mg/kg	64249-01-0	ND	0.01
12	#杀螨特	mg/kg	140-57-8	ND	0.01
13	#莠去津	mg/kg	1912-24-9	ND	0.01
14	#氧环唑	mg/kg	60207-31-0	ND	0.01
15	#甲基吡恶磷	mg/kg	35575-96-3	ND	0.01
16	谷硫磷（保棉磷）	mg/kg	86-50-0	ND	0.01
17	嘧菌酯（腈嘧菌酯）	mg/kg	131860-33-8	ND	0.01
18	苯霜灵	mg/kg	71626-11-4	ND	0.01
19	#解草嗪	mg/kg	98730-04-2	ND	0.01
20	#苄嘧磺隆	mg/kg	83055-99-6	ND	0.01
21	#地散磷	mg/kg	741-58-2	ND	0.01
22	#灭草松	mg/kg	25057-89-0	ND	0.01
23	#苯螨特	mg/kg	29104-30-1	ND	0.01
24	联苯三唑醇	mg/kg	55179-31-2	ND	0.01
25	啶酰菌胺	mg/kg	188425-85-6	ND	0.01
26	#除草定	mg/kg	314-40-9	ND	0.01
27	#溴丁酰草胺	mg/kg	74712-19-9	ND	0.01
28	溴苯腈	mg/kg	1689-84-5	ND	0.01
29	乙嘧酚磺酸酯	mg/kg	41483-43-6	ND	0.01
30	噻嗪酮	mg/kg	69327-76-0	ND	0.01

除非另有书面协议，本报告由本公司依据《 服务通用条款 》出具。
《 服务通用条款 》印刷在正本报告纸背面，或通过 https://www.sgs.com/en/Terms-and-Conditions 查询。请特别关注其中涉及责任限定，赔偿以及司法管辖的相关条款。任何报告的持有方需知悉，此报告内容仅反映SGS受限于客户指示下，且在当时所得结论。SGS仅对其客户负责，并且此报告不能免除交易各方根据交易文件所享有的权利和应履行的义务。未获得本公司书面批准，本文件不得进行复制，全文除外。任何未经授权对报告内容及形式进行的修改、伪造或扭曲都是违法行为，违法者将会被追究法律责任。除非另有声明，本测试报告所示结果仅涉及受测试样品。
注意：检测/检验报告或证书的真实性，请通过电话(86－755) 83071443或邮箱 CN.Doccheck@sgs.com 查询。

No.1, 5/F., Building D1, No. 1666, Section 2 of Chenglong Road, Economic and Technological Development Zone, Longquanyi District, Chengdu, Sichuan, China 610100　t (86–28) 8481 0338　www.sgsgroup.com.cn
中国・四川・成都市龙泉驿区经济技术开发区成龙大道二段1666号 D1栋05层1号　邮编：610100　t (86–28) 8481 0338　sgs.china@sgs.com

Member of the SGS Group (SGS SA)

编号	检测项目	单位	CAS No.	检测结果 1	定量限
31	#氟丙嘧草酯	mg/kg	134605-64-4	ND	0.01
32	#丁酮威	mg/kg	34681-10-2	ND	0.01
33	#丁酮砜威	mg/kg	34681-23-7	ND	0.01
34	甲萘威	mg/kg	63-25-2	ND	0.01
35	多菌灵	mg/kg	10605-21-7	ND	0.01
36	呋喃丹（虫螨威/克百威）	mg/kg	1563-66-2	ND	0.01
37	3-羟基虫螨威（3-羟基克百威）	mg/kg	16655-82-6	ND	0.01
38	萎锈灵	mg/kg	5234-68-4	ND	0.01
39	#唑草酮	mg/kg	128639-02-1	ND	0.01
40	#加普胺	mg/kg	104030-54-8	ND	0.01
41	#氯虫苯甲酰胺	mg/kg	500008-45-7	ND	0.01
42	#灭幼脲	mg/kg	57160-47-1	ND	0.01
43	#氯草灵	mg/kg	1967-16-4	ND	0.01
44	#杀虫脒	mg/kg	6164-98-3	ND	0.01
45	#氟啶脲	mg/kg	71422-67-8	ND	0.01
46	#氯草敏	mg/kg	1698-60-8	ND	0.01
47	#氯嘧磺隆	mg/kg	90982-32-4	ND	0.01
48	#绿麦隆	mg/kg	15545-48-9	ND	0.01
49	#枯草隆	mg/kg	1982-47-4	ND	0.01
50	#氯磺隆	mg/kg	64902-72-3	ND	0.01
51	#环虫酰肼	mg/kg	143807-66-3	ND	0.01
52	#吲哚酮草酯	mg/kg	142891-20-1	ND	0.01
53	#环庚草醚	mg/kg	87818-31-3	ND	0.01
54	#醚磺隆	mg/kg	94593-91-6	ND	0.01
55	#烯草酮	mg/kg	99129-21-2	ND	0.01
56	四螨嗪	mg/kg	74115-24-5	ND	0.01
57	#氯甲酰草胺	mg/kg	84496-56-0	ND	0.01
58	#调果酸（调采酸）	mg/kg	101-10-0	ND	0.01
59	#氯酯磺草胺	mg/kg	147150-35-4	ND	0.01
60	噻虫胺（可尼丁）	mg/kg	210880-92-5	ND	0.01
61	#氰草津	mg/kg	21725-46-2	ND	0.01
62	氰霜唑	mg/kg	120116-88-3	ND	0.01
63	#环丙酸酰胺	mg/kg	113136-77-9	ND	0.01
64	#环草敌（草灭特）	mg/kg	1134-23-2	ND	0.01
65	#环丙嘧磺隆	mg/kg	136849-15-5	ND	0.01
66	#环氟菌胺	mg/kg	180409-60-3	ND	0.01
67	霜脲氰	mg/kg	57966-95-7	ND	0.01
68	嘧菌环胺	mg/kg	121552-61-2	ND	0.01
69	#杀草隆	mg/kg	42609-52-9	ND	0.01
70	#内吸磷	mg/kg	8065-48-3	ND	0.01

除非另有书面协议，本报告由本公司依据《 服务通用条款 》出具。
《 服务通用条款 》印刷在正本报告纸背面，或通过 https://www.sgs.com/en/Terms-and-Conditions 查询。请特别关注其中涉及责任限定，赔偿以及司法管辖的相关条款。任何报告的持有方需知悉，此报告内容仅反映SGS受限于客户指示下，且在当时所得结论。SGS仅对其客户负责，并且此报告不能免除交易各方根据交易文件所享有的权利和应履行的义务。未获得本公司书面批准，本文件不得进行复制，全文除外。任何未经授权对报告内容及形式进行的修改、伪造或扭曲都是违法行为，违法者将会被追究法律责任。除非另有声明，本测试报告所示结果仅涉及受测试样品。
注意：检测/检验报告或证书的真实性，请通过电话 (86－755) 83071443 或邮箱 CN.Doccheck@sgs.com 查询。

No.1, 5/F., Building D1, No. 1666, Section 2 of Chenglong Road, Economic and Technological Development Zone, Longquanyi District, Chengdu, Sichuan, China 610100 t (86–28) 8481 0338 www.sgsgroup.com.cn
中国・四川・成都市龙泉驿区经济技术开发区成龙大道二段1666号 D1栋05层1号 邮编：610100 t (86–28) 8481 0338 sgs.china@sgs.com

Member of the SGS Group (SGS SA)

编号	检测项目	单位	CAS No.	检测结果 1	定量限
71	甲基砜内吸磷	mg/kg	17040-19-6	ND	0.01
72	#亚砜磷	mg/kg	301-12-2	ND	0.01
73	#甜菜胺（甜菜安）	mg/kg	13684-56-5	ND	0.01
74	#丁醚脲（丁嘧脲）	mg/kg	80060-09-9	ND	0.01
75	#燕麦敌（丁变敌）	mg/kg	2303-16-4	ND	0.01
76	#2,4-滴丙酸	mg/kg	120-36-5	ND	0.01
77	#双氯氰菌胺	mg/kg	139920-32-4	ND	0.01
78	#双氯磺草胺	mg/kg	145701-21-9	ND	0.01
79	百治磷	mg/kg	141-66-2	ND	0.01
80	乙霉威	mg/kg	87130-20-9	ND	0.01
81	苯醚甲环唑	mg/kg	119446-68-3	ND	0.01
82	除虫脲	mg/kg	35367-38-5	ND	0.01
83	#吡氟酰草胺	mg/kg	83164-33-4	ND	0.01
84	#哌草丹	mg/kg	61432-55-1	ND	0.01
85	#异戊乙净（阔草净）	mg/kg	22936-75-0	ND	0.01
86	#二甲嘧酚	mg/kg	5221-53-4	ND	0.01
87	乐果	mg/kg	60-51-5	ND	0.01
88	烯酰吗啉	mg/kg	110488-70-5	ND	0.01
89	烯唑醇	mg/kg	83657-24-3	ND	0.01
90	#敌螨普	mg/kg	39300-45-3	ND	0.01
91	#丁诺特呋喃（呋虫胺）	mg/kg	165252-70-0	ND	0.01
92	#特乐酚	mg/kg	1420-07-1	ND	0.01
93	敌草隆	mg/kg	330-54-1	ND	0.01
94	#甲氨基阿维菌素苯甲酸盐	mg/kg	155569-91-8	ND	0.01
95	#苯氧菌胺	mg/kg	133408-50-1	ND	0.01
96	氟环唑	mg/kg	106325-08-0 /133855-98-8	ND	0.01
97	#戊草丹	mg/kg	85785-20-2	ND	0.01
98	#乙硫苯威	mg/kg	29973-13-5	ND	0.01
99	#乙硫苯威砜	mg/kg	53380-23-7	ND	0.01
100	#乙硫苯威亚砜	mg/kg	53380-22-6	ND	0.01
101	#乙虫清	mg/kg	181587-01-9	ND	0.01
102	#乙嘧酚	mg/kg	23947-60-6	ND	0.01
103	#乙氧嘧磺隆	mg/kg	126801-58-9	ND	0.01
104	#乙氧苯草胺	mg/kg	79540-50-4	ND	0.01
105	#乙螨唑	mg/kg	153233-91-1	ND	0.01
106	咪唑菌酮	mg/kg	161326-34-7	ND	0.01
107	喹螨醚	mg/kg	120928-09-8	ND	0.01
108	腈苯唑	mg/kg	114369-43-6	ND	0.01
109	#甲呋酰胺	mg/kg	24691-80-3	ND	0.01

除非另有书面协议，本报告由本公司依据《 服务通用条款 》出具。
《 服务通用条款 》印刷在正本报告纸背面，或通过 https://www.sgs.com/en/Terms-and-Conditions 查询。请特别关注其中涉及责任限定，赔偿以及司法管辖的相关条款。任何报告的持有方需知悉，此报告内容仅反映SGS受限于客户指示下，且在当时所得结论。SGS仅对其客户负责，并且此报告不能免除交易各方根据交易文件所享有的权利和应履行的义务。未获得本公司书面批准，本文件不得进行复制，全文除外。任何未经授权对报告内容及形式进行的修改、伪造或扭曲都是违法行为，违法者将会被追究法律责任。除非另有声明，本测试报告所示结果仅涉及受测试样品。
注意：检测/检验报告或证书的真实性，请通过电话 (86－755) 83071443 或邮箱 CN.Doccheck@sgs.com 查询。

通标标准技术服务有限公司四川分公司
检验检测专用章
Inspection & Testing Services
农产食品实验室

No.1, 5/F, Building D1, No. 1666, Section 2 of Chenglong Road, Economic and Technological Development Zone, Longquanyi District, Chengdu, Sichuan, China 610100 t (86–28) 8481 0338 www.sgsgroup.com.cn
中国・四川・成都市龙泉驿区经济技术开发区成龙大道二段1666号 D1栋05层1号 邮编：610100 t (86–28) 8481 0338 sgs.china@sgs.com

Member of the SGS Group (SGS SA)

编号	检测项目	单位	CAS No.	检测结果 1	定量限
110	环酰菌胺	mg/kg	126833-17-8	ND	0.01
111	#仲丁威	mg/kg	3766-81-2	ND	0.01
112	#稻瘟酰胺（氰菌胺）	mg/kg	115852-48-7	ND	0.01
113	#恶唑禾草灵	mg/kg	66441-23-4	ND	0.01
114	双氧威（苯醚威）	mg/kg	72490-01-8 /79127-80-3	ND	0.01
115	苯锈啶	mg/kg	67306-00-7	ND	0.01
116	丁苯吗啉	mg/kg	67564-91-4	ND	0.01
117	唑螨酯	mg/kg	111812-58-9	ND	0.01
118	倍硫磷	mg/kg	55-38-9	ND	0.01
119	#倍硫磷砜	mg/kg	3761-42-0	ND	0.01
120	#倍硫磷亚砜	mg/kg	3761-41-9	ND	0.01
121	#四唑酰草胺（四唑啉酮）	mg/kg	158237-07-1	ND	0.01
122	#嘧菌腙	mg/kg	89269-64-7	ND	0.01
123	氟虫腈	mg/kg	120068-37-3	ND	0.01
124	#氟虫腈亚砜	mg/kg	120067-83-6	ND	0.01
125	#氟虫腈砜	mg/kg	120068-36-2	ND	0.01
126	#甲基麦草氟异丙酯（麦草氟甲酯）	mg/kg	52756-25-9	ND	0.01
127	#啶嘧磺隆	mg/kg	104040-78-0	ND	0.01
128	氟啶虫酰胺	mg/kg	158062-67-0	ND	0.01
129	#双氟磺草胺	mg/kg	145701-23-1	ND	0.01
130	#吡氟禾草灵和精吡氟禾草灵	mg/kg	69806-50-4 /79241-46-6	ND	0.01
131	#氟啶胺	mg/kg	79622-59-6	ND	0.01
132	#氟虫双酰胺（氟苯虫酰胺）	mg/kg	272451-65-7	ND	0.01
133	#氟唑磺隆	mg/kg	181274-17-9	ND	0.01
134	咯菌腈	mg/kg	131341-86-1	ND	0.01
135	#氟噻草胺	mg/kg	142459-58-3	ND	0.01
136	氟虫脲	mg/kg	101463-69-8	ND	0.01
137	#丁虫腈	mg/kg	704886-18-0	ND	0.01
138	#唑嘧磺草胺	mg/kg	98967-40-9	ND	0.01
139	#丙炔氟草胺	mg/kg	103361-09-7	ND	0.02
140	#氟吗啉	mg/kg	211867-47-9	ND	0.01
141	#伏草隆（氟草隆）	mg/kg	2164-17-2	ND	0.01
142	氟吡菌胺	mg/kg	239110-15-7	ND	0.01
143	#氯氟吡氧乙酸异辛酯	mg/kg	81406-37-3	ND	0.01
144	#磺菌胺	mg/kg	106917-52-6	ND	0.01
145	#氟噻乙草酯（氟赛乙草酯）	mg/kg	117337-19-6	ND	0.01
146	粉唑醇	mg/kg	76674-21-0	ND	0.01
147	#氟磺胺草醚	mg/kg	72178-02-0	ND	0.01
148	#氯吡脲	mg/kg	68157-60-8	ND	0.01

通标标准技术服务有限公司四川分公司 检验检测专用章 Inspection & Testing Services
通标标准技术服务有限公司四川分公司 农产品实验室

除非另有书面协议，本报告由本公司依据《 服务通用条款 》出具。
《 服务通用条款 》印刷在正本报告纸背面，或通过 https://www.sgs.com/en/Terms-and-Conditions 查询。请特别关注其中涉及责任限定，赔偿以及司法管辖的相关条款。任何报告的持有方需知悉，此报告内容仅反映SGS受限于客户指示下，且在当时所得结论。SGS仅对其客户负责，并且此报告不能免除交易各方根据交易文件所享有的权利和应履行的义务。未获得本公司书面批准，本文件不得进行复制，全文除外。任何未经授权对报告内容及形式进行的修改、伪造或扭曲都是违法行为，违法者将会被追究法律责任。除非另有声明，本测试报告所示结果仅涉及受测试样品。
注意：检测/检验报告或证书的真实性，请通过电话 (86-755) 83071443 或邮箱 CN.Doccheck@sgs.com 查询。
No.1, 5/F., Building D1, No. 1666, Section 2 of Chenglong Road, Economic and Technological Development Zone, Longquanyi District, Chengdu, Sichuan, China 610100 t (86–28) 8481 0338 www.sgsgroup.com.cn
中国・四川・成都市龙泉驿区经济技术开发区成龙大道二段1666号 D1栋05层1号 邮编：610100 t (86–28) 8481 0338 sgs.china@sgs.com
Member of the SGS Group (SGS SA)

编号	检测项目	单位	CAS No.	检测结果 1	定量限
149	#福拉比（呋比菌胺）	mg/kg	123572-88-3	ND	0.01
150	#呋线威	mg/kg	65907-30-4	ND	0.01
151	#氯吡嘧磺隆	mg/kg	100784-20-1	ND	0.01
152	#氟吡禾灵	mg/kg	69806-34-4	ND	0.01
153	#氟吡乙禾灵	mg/kg	87237-48-7	ND	0.01
154	#氟吡甲禾灵	mg/kg	69806-40-2	ND	0.01
155	己唑醇	mg/kg	79983-71-4	ND	0.01
156	#氟铃脲	mg/kg	86479-06-3	ND	0.01
157	噻螨酮	mg/kg	78587-05-0	ND	0.01
158	抑霉唑（烯菌灵）	mg/kg	35554-44-0	ND	0.01
159	#醚草酸甲酯	mg/kg	81405-85-8	ND	0.01
160	#甲氧咪草烟	mg/kg	114311-32-9	ND	0.01
161	#甲咪唑烟酸	mg/kg	104098-48-8	ND	0.01
162	#咪唑喹啉酸（灭草喹）	mg/kg	81335-37-7	ND	0.01
163	#亚胺唑	mg/kg	86598-92-7	ND	0.01
164	吡虫啉	mg/kg	138261-41-3	ND	0.01
165	#氯噻啉	mg/kg	105843-36-5	ND	0.01
166	#茚草酮	mg/kg	133220-30-1	ND	0.01
167	茚虫威（安打）	mg/kg	173584-44-6	ND	0.01
168	#甲基碘磺隆（钠盐）	mg/kg	144550-36-7	ND	0.01
169	#碘苯腈	mg/kg	1689-83-4	ND	0.01
170	#异稻瘟净	mg/kg	26087-47-8	ND	0.01
171	异菌脲	mg/kg	36734-19-7	ND	0.01
172	缬霉威	mg/kg	140923-17-7	ND	0.01
173	#异丙威	mg/kg	2631-40-5	ND	0.01
174	#稻瘟灵	mg/kg	50512-35-1	ND	0.01
175	#异丙隆	mg/kg	34123-59-6	ND	0.01
176	#异唑隆（异恶隆）	mg/kg	55861-78-4	ND	0.01
177	#双苯恶唑酸	mg/kg	163520-33-0	ND	0.01
178	#异恶氟草（异恶唑草酮）	mg/kg	141112-29-0	ND	0.01
179	醚菌酯（苯氧菊酯）	mg/kg	143390-89-0	ND	0.01
180	#乳氟禾草灵	mg/kg	77501-63-4	ND	0.01
181	#环草定（环草啶）	mg/kg	2164-08-1	ND	0.01
182	利谷隆	mg/kg	330-55-2	ND	0.01
183	虱螨脲（氟丙氧脲）	mg/kg	103055-07-8	ND	0.01
184	马拉硫磷	mg/kg	121-75-5	ND	0.01
185	双炔酰菌胺	mg/kg	374726-62-2	ND	0.01
186	#2 甲 4 氯	mg/kg	94-74-6	ND	0.01
187	#苯噻酰草胺	mg/kg	73250-68-7	ND	0.01
188	#吡咯解草酯（吡咯二酸二乙酯）	mg/kg	135590-91-9	ND	0.01

通标标准技术服务有限公司四川分公司 检验检测专用章 Inspection & Testing Services SGS-CSTC Standards Technical Services Co., Ltd. Sichuan Branch
通标标准技术服务有限公司四川分公司 农产食品实验室

除非另有书面协议，本报告由本公司依据《 服务通用条款 》出具。
《 服务通用条款 》印刷在正本报告纸背面，或通过 https://www.sgs.com/en/Terms-and-Conditions 查询。请特别关注其中涉及责任限定，赔偿以及司法管辖的相关条款。任何报告的持有方需知悉，此报告内容仅反映SGS受限于客户指示下，且在当时所得结论。SGS仅对其客户负责，并且此报告不能免除交易各方根据交易文件所享有的权利和应履行的义务。未获得本公司书面批准，本文件不得进行复制，全文除外。任何未经授权对报告内容及形式进行的修改、伪造或扭曲都是违法行为，违法者将会被追究法律责任。除非另有声明，本测试报告所示结果仅涉及受测试样品。
注意：检测/检验报告或证书的真实性，请通过电话(86－755) 83071443或邮箱 CN.Doccheck@sgs.com 查询。

No.1, 5/F., Building D1, No. 1666, Section 2 of Chenglong Road, Economic and Technological Development Zone, Longquanyi District, Chengdu, Sichuan, China 610100 t (86–28) 8481 0338 www.sgsgroup.com.cn
中国 • 四川 • 成都市龙泉驿区经济技术开发区成龙大道二段1666号 D1栋05层1号 邮编：610100 t (86–28) 8481 0338 sgs.china@sgs.com

Member of the SGS Group (SGS SA)

编号	检测项目	单位	CAS No.	检测结果 1	定量限
189	嘧菌胺	mg/kg	110235-47-7	ND	0.01
190	#甲基二磺隆	mg/kg	208465-21-8	ND	0.01
191	#硝磺草酮	mg/kg	104206-82-8	ND	0.01
192	#甲霜灵和精甲霜灵	mg/kg	57837-19-1 /70630-17-0	ND	0.01
193	甲霜灵（阿普隆）（瑞毒霜）	mg/kg	57837-19-1	ND	0.01
194	苯嗪草酮（灭它通）	mg/kg	41394-05-2	ND	0.01
195	#叶菌唑	mg/kg	125116-23-6	ND	0.01
196	#甲草苯噻隆（甲草苯隆）	mg/kg	18691-97-9	ND	0.01
197	甲胺磷	mg/kg	10265-92-6	ND	0.01
198	杀扑磷	mg/kg	950-37-8	ND	0.01
199	甲硫威（灭虫威）	mg/kg	2032-65-7	ND	0.01
200	灭多威	mg/kg	16752-77-5	ND	0.01
201	甲氧虫酰肼	mg/kg	161050-58-4	ND	0.01
202	异丙甲草胺	mg/kg	51218-45-2	ND	0.01
203	#速灭威	mg/kg	1129-41-5	ND	0.01
204	苯菌酮	mg/kg	220899-03-6	ND	0.01
205	#甲磺隆	mg/kg	74223-64-6	ND	0.01
206	#禾草敌	mg/kg	2212-67-1	ND	0.01
207	久效磷	mg/kg	6923-22-4	ND	0.01
208	腈菌唑（灭克落）	mg/kg	88671-89-0	ND	0.01
209	#萘草胺	mg/kg	132-66-1	ND	0.01
210	#烟嘧磺隆	mg/kg	111991-09-4	ND	0.01
211	#氟草敏（哒草伏）	mg/kg	27314-13-2	ND	0.01
212	#氟酰脲	mg/kg	116714-46-6	ND	0.01
213	#呋酰胺	mg/kg	58810-48-3	ND	0.01
214	氧乐果	mg/kg	1113-02-6	ND	0.01
215	#嘧苯胺磺隆	mg/kg	213464-77-8	ND	0.01
216	#丙炔噁草酮	mg/kg	39807-15-3	ND	0.01
217	恶草酮	mg/kg	19666-30-9	ND	0.01
218	恶霜灵	mg/kg	77732-09-3	ND	0.01
219	杀线威	mg/kg	23135-22-0	ND	0.01
220	#氯恶嗪草	mg/kg	153197-14-9	ND	0.01
221	#氧化萎锈灵	mg/kg	5259-88-1	ND	0.01
222	纹枯脲（戊菌隆）	mg/kg	66063-05-6	ND	0.01
223	#五氟磺草胺	mg/kg	219714-96-2	ND	0.01
224	甜菜宁	mg/kg	13684-63-4	ND	0.01
225	伏杀硫磷	mg/kg	2310-17-0	ND	0.01
226	#硫环磷	mg/kg	947-02-4	ND	0.01
227	#甲基硫环磷	mg/kg	5120-23-0	ND	0.01

通标标准技术服务有限公司四川分公司
检验检测专用章
Inspection & Testing Services
农产品食品实验室

除非另有书面协议，本报告由本公司依据《 服务通用条款 》出具。
《 服务通用条款 》印刷在正本报告纸背面，或通过 https://www.sgs.com/en/Terms-and-Conditions 查询。请特别关注其中涉及责任限定，赔偿以及司法管辖的相关条款。任何报告的持有方需知悉，此报告内容仅反映SGS受限于客户指示下，且在当时所得结论。SGS仅对其客户负责，并且此报告不能免除交易各方根据交易文件所享有的权利和应履行的义务。未获得本公司书面批准，本文件不得进行复制，全文除外。任何未经授权对报告内容及形式进行的修改、伪造或扭曲都是违法行为，违法者将会被追究法律责任。除非另有声明，本测试报告所示结果仅涉及受测试样品。
注意：检测/检验报告或证书的真实性，请通过电话 (86－755) 83071443 或邮箱 CN.Doccheck@sgs.com 查询。

No.1, 5/F., Building D1, No. 1666, Section 2 of Chenglong Road, Economic and Technological Development Zone, Longquanyi District, Chengdu, Sichuan, China 610100 t (86–28) 8481 0338 www.sgsgroup.com.cn
中国・四川・成都市龙泉驿区经济技术开发区成龙大道二段1666号 D1栋05层1号 邮编：610100 t (86–28) 8481 0338 sgs.china@sgs.com

Member of the SGS Group (SGS SA)

编号	检测项目	单位	CAS No.	检测结果 1	定量限
228	亚胺硫磷	mg/kg	732-11-6	ND	0.01
229	辛硫磷	mg/kg	14816-18-3	ND	0.01
230	#氟吡草胺	mg/kg	137641-05-5	ND	0.01
231	#啶氧菌酯	mg/kg	117428-22-5	ND	0.01
232	抗蚜威	mg/kg	23103-98-2	ND	0.01
233	#烯丙苯噻唑	mg/kg	27605-76-1	ND	0.01
234	#咪鲜胺	mg/kg	67747-09-5	ND	0.01
235	#茉莉酮（茉莉酸导体）	mg/kg	158474-72-7	ND	0.01
236	#猛杀威	mg/kg	2631-37-0	ND	0.01
237	#毒草胺	mg/kg	1918-16-7	ND	0.01
238	霜霉威	mg/kg	24579-73-5	ND	0.01
239	#恶草酸	mg/kg	111479-05-1	ND	0.01
240	炔螨特	mg/kg	2312-35-8	ND	0.01
241	#异丙草胺	mg/kg	86763-47-5	ND	0.01
242	残杀威	mg/kg	114-26-1	ND	0.01
243	苄草丹	mg/kg	52888-80-9	ND	0.01
244	#氟磺隆	mg/kg	94125-34-5	ND	0.01
245	吡蚜酮（吡嗪酮）	mg/kg	123312-89-0	ND	0.01
246	吡唑醚菌酯（唑菌胺酯）（百克敏）	mg/kg	175013-18-0	ND	0.01
247	#唑菌酯	mg/kg	862588-11-2	ND	0.01
248	#吡唑特	mg/kg	58011-68-0	ND	0.01
249	#吡菌磷	mg/kg	13457-18-6	ND	0.01
250	#乙基吡嘧磺隆（百速隆）	mg/kg	93697-74-6	ND	0.01
251	#苄草唑	mg/kg	71561-11-0	ND	0.01
252	#稗草丹	mg/kg	88678-67-5	ND	0.01
253	哒螨灵	mg/kg	96489-71-3	ND	0.01
254	#啶虫丙醚	mg/kg	179101-81-6	ND	0.01
255	#哒嗪硫磷	mg/kg	119-12-0	ND	0.01
256	啶斑肟（比芬诺）	mg/kg	88283-41-4	ND	0.01
257	#环酯草醚	mg/kg	135186-78-6	ND	0.01
258	嘧霉胺	mg/kg	53112-28-0	ND	0.01
259	#嘧螨醚	mg/kg	105779-78-0	ND	0.01
260	#吡丙醚（蚊蝇醚）	mg/kg	95737-68-1	ND	0.01
261	#咯哇酮（百快隆）	mg/kg	57369-32-1	ND	0.01
262	#灭藻醌	mg/kg	2797-51-5	ND	0.01
263	喹氧灵	mg/kg	124495-18-7	ND	0.01
264	#喹禾灵和精喹禾灵	mg/kg	100646-51-3 /76578-14-8	ND	0.01
265	#砜嘧磺隆	mg/kg	122931-48-0	ND	0.01
266	#鱼藤酮	mg/kg	83-79-4	ND	0.01

除非另有书面协议，本报告由本公司依据《 服务通用条款 》出具。
《 服务通用条款 》印刷在正本报告纸背面，或通过 https://www.sgs.com/en/Terms-and-Conditions 查询。请特别关注其中涉及责任限定，赔偿以及司法管辖的相关条款。任何报告的持有方需知悉，此报告内容仅反映SGS受限于客户指示下，且在当时所得结论。SGS仅对其客户负责，并且此报告不能免除交易各方根据交易文件所享有的权利和应履行的义务。未获得本公司书面批准，本文件不得进行复制，全文除外。任何未经授权对报告内容及形式进行的修改、伪造或扭曲都是违法行为，违法者将会被追究法律责任。除非另有声明，本测试报告所示结果仅涉及受测试样品。
注意：检测/检验报告或证书的真实性，请通过电话 (86 –755) 83071443 或邮箱 CN.Doccheck@sgs.com 查询。

No.1, 5/F., Building D1, No. 1666, Section 2 of Chenglong Road, Economic and Technological Development Zone, Longquanyi District, Chengdu, Sichuan, China 610100 t (86–28) 8481 0338 www.sgsgroup.com.cn
中国・四川・成都市龙泉驿区经济技术开发区成龙大道二段1666号 D1栋05层1号 邮编：610100 t (86–28) 8481 0338 sgs.china@sgs.com

通标标准技术服务有限公司四川分公司 检验检测专用章 Inspection & Testing Services
通标标准技术服务有限公司四川分公司 农产食品实验室

Member of the SGS Group (SGS SA)

检测报告

CDF25-0005759-01　　发布日期：2025-10-22

编号	检测项目	单位	CAS No.	检测结果 1	定量限
267	#单甲脒	mg/kg	33089-74-6	ND	0.01
268	#稀禾定	mg/kg	74051-80-2	ND	0.01
269	#西玛津	mg/kg	122-34-9	ND	0.01
270	#硅氟唑	mg/kg	149508-90-7	ND	0.01
271	#西草净	mg/kg	1014-70-6	ND	0.01
272	螺螨酯	mg/kg	148477-71-8	ND	0.01
273	#螺虫乙酯	mg/kg	203313-25-1	ND	0.01
274	螺恶茂胺（螺环菌胺）	mg/kg	118134-30-8	ND	0.01
275	#磺草酮	mg/kg	99105-77-8	ND	0.01
276	#乙黄隆（磺酰磺隆）	mg/kg	141776-32-1	ND	0.01
277	#氟啶虫胺腈	mg/kg	946578-00-3	ND	0.01
278	戊唑醇	mg/kg	107534-96-3	ND	0.01
279	虫酰肼	mg/kg	112410-23-8	ND	0.01
280	吡螨胺	mg/kg	119168-77-3	ND	0.02
281	#特丁隆	mg/kg	34014-18-1	ND	0.01
282	氟苯脲	mg/kg	83121-18-0	ND	0.01
283	#噻吩草胺（甲氧噻草胺）	mg/kg	96491-05-3	ND	0.01
284	噻菌灵	mg/kg	148-79-8	ND	0.01
285	噻虫啉	mg/kg	111988-49-9	ND	0.01
286	噻虫嗪	mg/kg	153719-23-4	ND	0.01
287	#噻唑烟酸（噻草啶）	mg/kg	117718-60-2	ND	0.01
288	#赛苯隆	mg/kg	51707-55-2	ND	0.01
289	#噻吩磺隆（阔叶散）	mg/kg	79277-27-3	ND	0.01
290	#噻呋酰胺	mg/kg	130000-40-7	ND	0.01
291	#禾草丹	mg/kg	28249-77-6	ND	0.01
292	#硫双威	mg/kg	59669-26-0	ND	0.01
293	#久效威砜	mg/kg	39184-59-3	ND	0.01
294	#久效威亚砜	mg/kg	39184-27-5	ND	0.01
295	#甲基硫菌灵	mg/kg	23564-05-8	ND	0.01
296	#噻酰菌胺	mg/kg	223580-51-6	ND	0.01
297	#唑虫酰胺	mg/kg	129558-76-5	ND	0.01
298	#苯草酮（甲氧基草酮）	mg/kg	87820-88-0	ND	0.01
299	#三唑酮	mg/kg	43121-43-3	ND	0.01
300	三唑醇	mg/kg	55219-65-3	ND	0.01
301	#醚苯磺隆	mg/kg	82097-50-5	ND	0.01
302	三唑磷	mg/kg	24017-47-8	ND	0.01
303	#苯磺隆	mg/kg	101200-48-0	ND	0.01
304	#敌百虫	mg/kg	52-68-6	ND	0.01
305	#氯啶菌酯	mg/kg	902760-40-1	ND	0.01
306	三环唑	mg/kg	41814-78-2	ND	0.01

除非另有书面协议，本报告由本公司依据《服务通用条款》出具。
《服务通用条款》印刷在正本报告纸背面，或通过 https://www.sgs.com/en/Terms-and-Conditions 查询。请特别关注其中涉及责任限定，赔偿以及司法管辖的相关条款。任何报告的持有方需知悉，此报告内容仅反映SGS受限于客户指示下，且在当时所得结论。SGS仅对其客户负责，并且此报告不能免除交易各方根据交易文件所享有的权利和应履行的义务。未获得本公司书面批准，本文件不得进行复制，全文除外。任何未经授权对报告内容及形式进行的修改、伪造或扭曲都是违法行为，违法者将会被追究法律责任。除非另有声明，本测试报告所示结果仅涉及受测试样品。
注意：检测/检验报告或证书的真实性，请通过电话 (86－755) 83071443 或邮箱 CN.Doccheck@sgs.com 查询。

通标标准技术服务有限公司四川分公司 检验检测专用章 Inspection & Testing Services
通标标准技术服务有限公司四川分公司 农产食品实验室

No.1, 5/F., Building D1, No. 1666, Section 2 of Chenglong Road, Economic and Technological Development Zone, Longquanyi District, Chengdu, Sichuan, China 610100 t (86–28) 8481 0338 www.sgsgroup.com.cn
中国・四川・成都市龙泉驿区经济技术开发区成龙大道二段1666号 D1栋05层1号 邮编：610100 t (86–28) 8481 0338 sgs.china@sgs.com

Member of the SGS Group (SGS SA)

编号	检测项目	单位	CAS No.	检测结果 1	定量限
307	#十三吗啉	mg/kg	24602-86-6	ND	0.01
308	肟菌酯	mg/kg	141517-21-7	ND	0.01
309	氟菌唑	mg/kg	68694-11-1	ND	0.01
310	杀铃脲	mg/kg	64628-44-0	ND	0.01
311	#氟胺磺隆	mg/kg	126535-15-7	ND	0.01
312	#抗倒酯	mg/kg	95266-40-3	ND	0.01
313	#灭菌唑	mg/kg	131983-72-7	ND	0.01
314	#烯效唑和高效烯效唑	mg/kg	83657-17-4 /83657-22-1	ND	0.01
315	#蚜灭磷（完灭硫磷）	mg/kg	2275-23-2	ND	0.01
316	#灭除威	mg/kg	2655-14-3	ND	0.01
317	苯酰菌胺（苯酰草胺）	mg/kg	156052-68-5	ND	0.01
318	#三唑酮和三唑醇之和	mg/kg	-	ND	-
319	#灭多威（灭多威和硫双威之和）	mg/kg	-	ND	-
320	克百威(克百威和 3-羟基克百威之和，以克百威表示)	mg/kg	-	ND	-
321	#氟虫腈(氟虫腈、氟虫腈砜及氟虫腈亚砜之和，以氟虫腈表示)	mg/kg	-	ND	-
322	#涕灭威(涕灭威、涕灭威亚砜及涕灭砜威之和，以涕灭威表示)	mg/kg	-	ND	-
323	#倍硫磷(倍硫磷、倍硫磷亚砜及倍硫磷砜之和，以倍硫磷表示)	mg/kg	-	ND	-
324	#乙硫甲威（乙硫甲威,乙硫甲威-砜,乙硫甲威-亚砜之和，以乙硫甲威表示）	mg/kg	-	ND	-
325	#环丙氨嗪(灭蝇胺)	mg/kg	66215-27-8	ND	0.01
326	#二硫酮砜	mg/kg	2497-06-5	ND	0.01
327	#二硫代亚砜	mg/kg	2497-07-6	ND	0.01
328	#氟甲腈	mg/kg	205650-65-3	ND	0.01
329	#噁唑菌酮	mg/kg	131807-57-3	ND	0.01
330	#咪鲜胺-脱氨基咪唑	mg/kg	139520-94-8	ND	0.01
331	#咪鲜胺-脱咪唑甲酰胺基	mg/kg	139542-32-8	ND	0.01
332	#丁氟螨酯	mg/kg	400882-07-7	ND	0.01
333	#苯嘧磺草胺	mg/kg	372137-35-4	ND	0.01
334	#氟唑环菌胺	mg/kg	874967-67-6	ND	0.01
335	#胺苯吡菌酮	mg/kg	473798-59-3	ND	0.01
336	#消螨酚	mg/kg	131-89-5	ND	0.01
337	#戊硝酚	mg/kg	4097-36-3	ND	0.01
338	#烯啶虫胺	mg/kg	120738-89-8 /150824-47-8	ND	0.01
339	#联苯肼酯	mg/kg	149877-41-8	ND	0.01
340	#吡噻菌胺	mg/kg	183675-82-3	ND	0.01

除非另有书面协议，本报告由本公司依据《 服务通用条款 》出具。
《 服务通用条款 》印刷在正本报告纸背面，或通过 https://www.sgs.com/en/Terms-and-Conditions 查询。请特别关注其中涉及责任限定，赔偿以及司法管辖的相关条款。任何报告的持有方需知悉，此报告内容仅反映SGS受限于客户指示下，且在当时所得结论。SGS仅对其客户负责，并且此报告不能免除交易各方根据交易文件所享有的权利和应履行的义务。未获得本公司书面批准，本文件不得进行复制，全文除外。任何未经授权对报告内容及形式进行的修改、伪造或扭曲都是违法行为，违法者将会被追究法律责任。除非另有声明，本测试报告所示结果仅涉及受测试样品。
注意：检测/检验报告或证书的真实性，请通过电话(86－755) 83071443或邮箱 CN.Doccheck@sgs.com查询。

No.1, 5/F., Building D1, No. 1666, Section 2 of Chenglong Road, Economic and Technological Development Zone, Longquanyi District, Chengdu, Sichuan, China 610100 t (86–28) 8481 0338 www.sgsgroup.com.cn
中国・四川・成都市龙泉驿区经济技术开发区成龙大道二段1666号 D1栋05层1号 邮编：610100 t (86–28) 8481 0338 sgs.china@sgs.com

通标标准技术服务有限公司四川分公司 检验检测专用章 Inspection & Testing Services
通标标准技术服务有限公司四川分公司 农产食品实验室

Member of the SGS Group (SGS SA)

编号	检测项目	单位	CAS No.	检测结果 1	定量限
341	2,4-滴 & 2,4-滴钠盐	mg/kg	2702-72-9 /94-75-7	ND	0.01
342	#伊维菌素	mg/kg	70288-86-7	ND	0.01
343	#除虫菊素 I	mg/kg	121-21-1	ND	0.01
344	#除虫菊素 II	mg/kg	121-29-9	ND	0.01
345	#氟咯草酮	mg/kg	61213-25-0	ND	0.01
346	#扑草净	mg/kg	7287-19-6	ND	0.01
347	多杀霉素	mg/kg	168316-95-8	ND	0.01
348	#胺苯磺隆	mg/kg	97780-06-8	ND	0.01
349	#唑嘧菌胺	mg/kg	865318-97-4	ND	0.01
350	#噻草酮	mg/kg	101205-02-1	ND	0.01
351	#唑胺菌酯	mg/kg	915410-70-7	ND	0.01
352	#丁吡吗啉	mg/kg	868390-90-3	ND	0.01
353	#啶菌噁唑	mg/kg	847749-37-5	ND	0.01
354	#乙基多杀菌素 J	mg/kg	187166-40-1	ND	0.01
355	#咪鲜胺(咪鲜胺、咪鲜胺-脱氨基咪唑及咪鲜胺-脱咪唑甲酰胺基之和，以咪鲜胺表示)	mg/kg	-	ND	-
356	#胺鲜酯	mg/kg	10369-83-2	ND	0.01
357	#苯菌灵和多菌灵	mg/kg	-	ND	0.01
358	#苯并烯氟菌唑	mg/kg	1072957-71-1	ND	0.01
359	#烯草酮亚砜	mg/kg	111031-14-2	ND	0.01
360	#烯草酮砜	mg/kg	111031-17-5	ND	0.01
361	#螺虫乙酯-酮基-羟基	mg/kg	1172134-11-0	ND	0.01
362	#螺虫乙酯-烯醇-葡萄糖苷	mg/kg	1172614-86-6	ND	0.01
363	#螺虫乙酯-单-羟基	mg/kg	1172134-12-1	ND	0.01
364	#甲磺草胺	mg/kg	122836-35-5	ND	0.01
365	#丰索磷砜	mg/kg	14255-72-2	ND	0.01
366	#螺虫乙酯-烯醇	mg/kg	203312-38-3	ND	0.01
367	#甲硫威砜	mg/kg	2179-25-1	ND	0.01
368	#甲硫威亚砜	mg/kg	2635-10-1	ND	0.01
369	#烯肟菌酯	mg/kg	238410-11-2	ND	0.01
370	#N-二甲基甲酰基甲硫基甲醛肟	mg/kg	30558-43-1	ND	0.01
371	#烯肟菌胺	mg/kg	366815-39-6	ND	0.01
372	#阿苯达唑	mg/kg	54965-21-8	ND	0.01
373	#丙嗪嘧磺隆	mg/kg	570415-88-2	ND	0.01
374	#丰索磷-氧化砜	mg/kg	6132-17-8	ND	0.01
375	#氟节胺	mg/kg	62924-70-3	ND	0.01

除非另有书面协议，本报告由本公司依据《服务通用条款》出具。
《服务通用条款》印刷在正本报告纸背面，或通过 https://www.sgs.com/en/Terms-and-Conditions 查询。请特别关注其中涉及责任限定，赔偿以及司法管辖的相关条款。任何报告的持有方需知悉，此报告内容仅反映SGS受限于客户指示下，且在当时所得结论。SGS仅对其客户负责，并且此报告不能免除交易各方根据交易文件所享有的权利和应履行的义务。未获得本公司书面批准，本文件不得进行复制，全文除外。任何未经授权对报告内容及形式进行的修改、伪造或扭曲都是违法行为，违法者将会被追究法律责任。除非另有声明，本检测报告所示结果仅涉及受测试样品。
注意：检测／检验报告或证书的真实性，请通过电话(86－755) 83071443或邮箱 CN.Doccheck@sgs.com 查询。

通标标准技术服务有限公司四川分公司 检验检测专用章 Inspection & Testing Services 农产食品实验室

No.1, 5/F., Building D1, No. 1666, Section 2 of Chenglong Road, Economic and Technological Development Zone, Longquanyi District, Chengdu, Sichuan, China 610100 t (86–28) 8481 0338 www.sgsgroup.com.cn
中国・四川・成都市龙泉驿区经济技术开发区成龙大道二段1666号 D1栋05层1号 邮编：610100 t (86–28) 8481 0338 sgs.china@sgs.com

Member of the SGS Group (SGS SA)

编号	检测项目	单位	CAS No.	检测结果 1	定量限
376	#丰索磷-氧	mg/kg	6552-21-2	ND	0.01
377	#丁香菌酯	mg/kg	850881-70-8	ND	0.01
378	#嗪吡嘧磺隆	mg/kg	868680-84-6	ND	0.01
379	#吡唑萘菌胺	mg/kg	881685-58-1	ND	0.01
380	#螺虫乙酯(螺虫乙酯、螺虫乙酯-烯醇、螺虫乙酯-烯醇-葡萄糖苷、螺虫乙酯-酮基-羟基及螺虫乙酯-单-羟基之和，以螺虫乙酯表示)	mg/kg	-	ND	0.01
381	#甲硫威(甲硫威、甲硫威亚砜及甲硫威砜之和，以甲硫威表示)	mg/kg	-	ND	0.01
382	#亚砜磷(亚砜磷、甲基内吸磷及砜吸磷之和，以亚砜磷表示)	mg/kg	-	ND	-
383	#烯草酮(烯草酮、烯草酮亚砜及烯草酮砜之和，以烯草酮表示)	mg/kg	-	ND	-
384	#丰索磷(丰索磷、氧丰索磷、氧丰索磷砜及丰索磷砜，以丰索磷表示)	mg/kg	-	ND	-
385	#除虫菊素(除虫菊素 I 和除虫菊素 II 之和)	mg/kg	-	ND	-

检测方法： EN 12393-2013

编号	检测项目	单位	CAS No.	检测结果 1	定量限
386	#2,4-滴丁酯	mg/kg	94-80-4	ND	0.01
387	邻苯基苯酚（2-苯基苯酚）（OPP）	mg/kg	90-43-7	ND	0.01
388	#氟丙菊酯	mg/kg	101007-06-1	ND	0.01
389	甲草胺	mg/kg	15972-60-8	ND	0.01
390	艾氏剂	mg/kg	309-00-2	ND	0.01
391	莠灭净	mg/kg	834-12-8	ND	0.02
392	#蒽醌	mg/kg	84-65-1	ND	0.01
393	益棉磷（乙基保棉磷）（乙基谷硫磷）	mg/kg	2642-71-9	ND	0.02
394	噁虫威	mg/kg	22781-23-3	ND	0.01
395	乙丁氟灵	mg/kg	1861-40-1	ND	0.01
396	#呋草黄（呋草磺）	mg/kg	68505-69-1	ND	0.01
397	联苯菊酯	mg/kg	82657-04-3	ND	0.01
398	溴硫磷	mg/kg	2104-96-3	ND	0.01
399	乙基溴硫磷	mg/kg	4824-78-6	ND	0.01
400	溴螨酯	mg/kg	18181-80-1	ND	0.01
401	#丁草胺	mg/kg	23184-66-9	ND	0.01
402	#抑草磷	mg/kg	36335-67-8	ND	0.01
403	仲丁灵	mg/kg	33629-47-9	ND	0.01

除非另有书面协议，本报告由本公司依据《 服务通用条款 》出具。
《 服务通用条款 》印刷在正本报告纸背面，或通过 https://www.sgs.com/en/Terms-and-Conditions 查询。请特别关注其中涉及责任限定，赔偿以及司法管辖的相关条款。任何报告的持有方需知悉，此报告内容仅反映SGS受限于客户指示下，且在当时所得结论。SGS仅对其客户负责，并且此报告不能免除交易各方根据交易文件所享有的权利和应履行的义务。未获得本公司书面批准，本文件不得进行复制，全文除外。任何未经授权对报告内容及形式进行的修改、伪造或扭曲都是违法行为，违法者将会被追究法律责任。除非另有声明，本测试报告所示结果仅涉及受测试样品。
注意：检测/检验报告或证书的真实性，请通过电话(86-755) 83071443或邮箱 CN.Doccheck@sgs.com 查询。

通标标准技术服务有限公司四川分公司 检验检测专用章 Inspection & Testing Services
通标标准技术服务有限公司四川分公司 农产食品实验室

No.1, 5/F., Building D1, No. 1666, Section 2 of Chenglong Road, Economic and Technological Development Zone, Longquanyi District, Chengdu, Sichuan, China 610100 t (86–28) 8481 0338 www.sgsgroup.com.cn
中国・四川・成都市龙泉驿区经济技术开发区成龙大道二段1666号 D1栋05层1号 邮编: 610100 t (86–28) 8481 0338 sgs.china@sgs.com

Member of the SGS Group (SGS SA)

编号	检测项目	单位	CAS No.	检测结果 1	定量限
404	硫线磷	mg/kg	95465-99-9	ND	0.02
405	三硫磷	mg/kg	786-19-6	ND	0.01
406	杀螨醚（氯杀螨）	mg/kg	103-17-3	ND	0.01
407	虫螨腈（溴虫腈）	mg/kg	122453-73-0	ND	0.01
408	杀螨酯	mg/kg	80-33-1	ND	0.01
409	毒虫畏（杀螟威）	mg/kg	470-90-6	ND	0.01
410	乙酯杀螨醇（克氯苯）	mg/kg	510-15-6	ND	0.01
411	氯苯甲醚	mg/kg	2675-77-6	ND	0.02
412	氯苯胺灵	mg/kg	101-21-3	ND	0.01
413	毒死蜱	mg/kg	2921-88-2	ND	0.01
414	甲基毒死蜱	mg/kg	5598-13-0	ND	0.01
415	氯酞酸甲酯	mg/kg	1861-32-1	ND	0.01
416	乙菌利（克氯得）	mg/kg	84332-86-5	ND	0.01
417	顺式-氯丹（α-氯丹）	mg/kg	5103-71-9	ND	0.01
418	炔草酯（炔草酸）	mg/kg	105512-06-9	ND	0.01
419	异噁草松（异噁草酮）	mg/kg	81777-89-1	ND	0.01
420	#苯腈磷（苯腈硫磷）	mg/kg	13067-93-1	ND	0.01
421	杀螟腈（杀虫腈）	mg/kg	2636-26-2	ND	0.01
422	#氰氟草酯	mg/kg	122008-85-9	ND	0.01
423	环丙唑醇	mg/kg	94361-06-5	ND	0.01
424	1,2,4-三丁基三硫磷酸酯（脱叶磷）	mg/kg	78-48-8	ND	0.01
425	溴氰菊酯	mg/kg	52918-63-5	ND	0.01
426	二嗪磷（二嗪农）	mg/kg	333-41-5	ND	0.01
427	除线磷	mg/kg	97-17-6	ND	0.01
428	#苯氟磺胺（抑菌灵）	mg/kg	1085-98-9	ND	0.01
429	#烯丙酰草胺（二氯丙烯胺）	mg/kg	37764-25-3	ND	0.01
430	敌敌畏	mg/kg	62-73-7	ND	0.01
431	#苄氯三唑醇	mg/kg	75736-33-3	ND	0.01
432	禾草灵	mg/kg	51338-27-3	ND	0.01
433	氯硝胺	mg/kg	99-30-9	ND	0.01
434	狄氏剂	mg/kg	60-57-1	ND	0.01
435	#噻节因	mg/kg	55290-64-7	ND	0.01
436	#甲基毒虫畏	mg/kg	2274-67-1	ND	0.01
437	#蔬果磷	mg/kg	3811-49-2	ND	0.01
438	二苯胺	mg/kg	122-39-4	ND	0.01
439	#氟硫草定	mg/kg	97886-45-8	ND	0.01
440	硫丹硫酸酯	mg/kg	1031-07-8	ND	0.01
441	异狄氏剂	mg/kg	72-20-8	ND	0.01
442	苯硫磷	mg/kg	2104-64-5	ND	0.01
443	#乙丁烯氟灵	mg/kg	55283-68-6	ND	0.01

通标标准技术服务有限公司四川分公司 检验检测专用章 Inspection & Testing Services 农产食品实验室

除非另有书面协议，本报告由本公司依据《服务通用条款》出具。
《服务通用条款》印刷在正本报告纸背面，或通过 https://www.sgs.com/en/Terms-and-Conditions 查询。请特别关注其中涉及责任限定，赔偿以及司法管辖的相关条款。任何报告的持有方需知悉，此报告内容仅反映SGS受限于客户指示下，且在当时所得结论。SGS仅对其客户负责，并且此报告不能免除交易各方根据交易文件所享有的权利和应履行的义务。未获得本公司书面批准，本文件不得进行复制，全文除外。任何未经授权对报告内容及形式进行的修改、伪造或扭曲都是违法行为，违法者将会被追究法律责任。除非另有声明，本测试报告所示结果仅涉及受测试样品。
注意：检测/检验报告或证书的真实性，请通过电话(86－755) 83071443或邮箱 CN.Doccheck@sgs.com查询。

No.1, 5/F., Building D1, No. 1666, Section 2 of Chenglong Road, Economic and Technological Development Zone, Longquanyi District, Chengdu, Sichuan, China 610100 t (86–28) 8481 0338 www.sgsgroup.com.cn
中国・四川・成都市龙泉驿区经济技术开发区成龙大道二段1666号 D1栋05层1号 邮编：610100 t (86–28) 8481 0338 sgs.china@sgs.com

Member of the SGS Group (SGS SA)

编号	检测项目	单位	CAS No.	检测结果 1	定量限
444	乙硫磷	mg/kg	563-12-2	ND	0.01
445	醚菊酯	mg/kg	80844-07-1	ND	0.01
446	乙氧呋草黄	mg/kg	26225-79-6	ND	0.01
447	灭线磷	mg/kg	13194-48-4	ND	0.01
448	乙嘧硫磷	mg/kg	38260-54-7	ND	0.01
449	皮蝇磷	mg/kg	299-84-3	ND	0.01
450	#敌草腈	mg/kg	1194-65-6	ND	0.01
451	杀螟硫磷（杀螟松）	mg/kg	122-14-5	ND	0.01
452	甲氰菊酯	mg/kg	39515-41-8 /64257-84-7	ND	0.01
453	丰索磷	mg/kg	115-90-2	ND	0.01
454	氰戊菊酯和 S-氰戊菊酯	mg/kg	51630-58-1 /66230-04-4	ND	0.01
455	嘧螨酯	mg/kg	229977-93-9	ND	0.01
456	氟氰戊菊酯	mg/kg	70124-77-5	ND	0.01
457	氟哇唑（氟硅唑）	mg/kg	85509-19-9	ND	0.01
458	#氟酰胺	mg/kg	66332-96-5	ND	0.01
459	地虫硫磷	mg/kg	944-22-9	ND	0.01
460	#安硫磷（安果磷）	mg/kg	2540-82-1	ND	0.01
461	呋霜灵	mg/kg	57646-30-7	ND	0.01
462	#苄螨醚（苄满醚）	mg/kg	111872-58-3	ND	0.01
463	七氯	mg/kg	76-44-8	ND	0.01
464	环氧七氯（B 型，顺式）	mg/kg	1024-57-3 /28044-83-9	ND	0.01
465	庚烯磷	mg/kg	23560-59-0	ND	0.01
466	六氯苯	mg/kg	118-74-1	ND	0.01
467	异艾氏剂	mg/kg	465-73-6	ND	0.01
468	异柳磷	mg/kg	25311-71-1	ND	0.01
469	甲基异柳磷	mg/kg	99675-03-3	ND	0.01
470	γ-六六六（林丹）	mg/kg	58-89-9	ND	0.01
471	#灭蚜磷	mg/kg	2595-54-2	ND	0.01
472	虫螨畏	mg/kg	62610-77-9	ND	0.01
473	甲氧氯（甲氧滴滴涕）	mg/kg	72-43-5	ND	0.01
474	#五氯苯甲硫醚（甲基五氯苯硫醚）	mg/kg	1825-19-0	ND	0.01
475	嗪草酮	mg/kg	21087-64-9	ND	0.01
476	速灭磷	mg/kg	7786-34-7	ND	0.01
477	灭蚁灵	mg/kg	2385-85-5	ND	0.01
478	敌草胺	mg/kg	15299-99-7	ND	0.01
479	除草醚	mg/kg	1836-75-5	ND	0.01
480	酞菌酯	mg/kg	10552-74-6	ND	0.01
481	o,p'-滴滴涕	mg/kg	789-02-6	ND	0.01

通标标准技术服务有限公司四川分公司
检验检测专用章
Inspection & Testing Services

除非另有书面协议，本报告由本公司依据《 服务通用条款 》出具。
《 服务通用条款 》印刷在正本报告纸背面，或通过 https://www.sgs.com/en/Terms-and-Conditions 查询。请特别关注其中涉及责任限定，赔偿以及司法管辖的相关条款。任何报告的持有方需知悉，此报告内容仅反映SGS受限于客户指示下，且在当时所得结论。SGS仅对其客户负责，并且此报告不能免除交易各方根据交易文件所享有的权利和应履行的义务。未获得本公司书面批准，本文件不得进行复制，全文除外。任何未经授权对报告内容及形式进行的修改、伪造或扭曲都是违法行为，违法者将会被追究法律责任。除非另有声明，本测试报告所示结果仅涉及受测试样品。
注意：检测/检验报告或证书的真实性，请通过电话 (86－755) 83071443 或邮箱 CN.Doccheck@sgs.com 查询。

No.1, 5/F., Building D1, No. 1666, Section 2 of Chenglong Road, Economic and Technological Development Zone, Longquanyi District, Chengdu, Sichuan, China 610100 t (86–28) 8481 0338 www.sgsgroup.com.cn
中国・四川・成都市龙泉驿区经济技术开发区成龙大道二段1666号 D1栋05层1号 邮编：610100 t (86–28) 8481 0338 sgs.china@sgs.com

Member of the SGS Group (SGS SA)

编号	检测项目	单位	CAS No.	检测结果 1	定量限
482	p,p'-滴滴滴	mg/kg	72-54-8	ND	0.01
483	p,p'-滴滴伊	mg/kg	72-55-9	ND	0.01
484	p,p'-滴滴涕	mg/kg	50-29-3	ND	0.01
485	多效唑	mg/kg	76738-62-0	ND	0.01
486	乙基对硫磷（对硫磷）	mg/kg	56-38-2	ND	0.01
487	甲基对硫磷	mg/kg	298-00-0	ND	0.01
488	戊菌唑	mg/kg	66246-88-6	ND	0.01
489	二甲戊灵（二甲戊乐灵）	mg/kg	40487-42-1	ND	0.01
490	五氯苯胺	mg/kg	527-20-8	ND	0.01
491	五氯甲氧基苯（五氯茴香醚）	mg/kg	1825-21-4	ND	0.01
492	#戊基恶唑酮	mg/kg	110956-75-7	ND	0.01
493	氯菊酯	mg/kg	52645-53-1	ND	0.01
494	#苯醚菊酯	mg/kg	26002-80-2	ND	0.01
495	稻丰散	mg/kg	2597-03-7	ND	0.01
496	甲拌磷	mg/kg	298-02-2	ND	0.01
497	磷胺	mg/kg	13171-21-6	ND	0.02
498	#四氯苯酞	mg/kg	27355-22-2	ND	0.01
499	增效醚	mg/kg	51-03-6	ND	0.01
500	#哌草磷	mg/kg	24151-93-7	ND	0.01
501	嘧啶磷（乙基嘧啶磷）	mg/kg	23505-41-1	ND	0.01
502	甲基虫螨磷（甲基嘧啶磷）	mg/kg	29232-93-7	ND	0.01
503	腐霉利	mg/kg	32809-16-8	ND	0.01
504	丙溴磷（溴氯磷）	mg/kg	41198-08-7	ND	0.01
505	#丙虫磷	mg/kg	7292-16-2	ND	0.01
506	胺丙畏（巴胺磷）（烯虫磷）	mg/kg	31218-83-4	ND	0.01
507	苯胺灵	mg/kg	122-42-9	ND	0.01
508	丙环唑	mg/kg	60207-90-1	ND	0.01
509	炔苯酰草胺（拿草特）	mg/kg	23950-58-5	ND	0.01
510	丙硫磷（普硫松）	mg/kg	34643-46-4	ND	0.01
511	#嘧草醚	mg/kg	136191-64-5	ND	0.01
512	喹硫磷	mg/kg	13593-03-8	ND	0.01
513	五氯硝基苯	mg/kg	82-68-8	ND	0.01
514	八氯二丙醚	mg/kg	127-90-2	ND	0.01
515	#硅醚菊酯	mg/kg	105024-66-6	ND	0.01
516	治螟磷	mg/kg	3689-24-5	ND	0.01
517	硫丙磷	mg/kg	35400-43-2	ND	0.01
518	四氯硝基苯	mg/kg	117-18-0	ND	0.01
519	七氟菊酯	mg/kg	79538-32-2	ND	0.01
520	特丁硫磷	mg/kg	13071-79-9	ND	0.01
521	杀虫畏	mg/kg	22248-79-9	ND	0.02

除非另有书面协议，本报告由本公司依据《 服务通用条款 》出具。
《 服务通用条款 》印刷在正本报告纸背面，或通过 https://www.sgs.com/en/Terms-and-Conditions 查询。请特别关注其中涉及责任限定，赔偿以及司法管辖的相关条款。任何报告的持有方需知悉，此报告内容仅反映SGS受限于客户指示下，且在当时所得结论。SGS仅对其客户负责，并且此报告不能免除交易各方根据交易文件所享有的权利和应履行的义务。未获得本公司书面批准，本文件不得进行复制，全文除外。任何未经授权对报告内容及形式进行的修改、伪造或扭曲都是违法行为，违法者将会被追究法律责任。除非另有声明，本测试报告所示结果仅涉及受测试样品。
注意：检测/检验报告或证书的真实性，请通过电话(86－755) 83071443或邮箱 CN.Doccheck@sgs.com 查询。

通标标准技术服务有限公司四川分公司 检验检测专用章 Inspection & Testing Services
农产品实验室

No.1, 5/F., Building D1, No. 1666, Section 2 of Chenglong Road, Economic and Technological Development Zone, Longquanyi District, Chengdu, Sichuan, China 610100 t (86–28) 8481 0338 www.sgsgroup.com.cn
中国・四川・成都市龙泉驿区经济技术开发区成龙大道二段1666号 D1栋05层1号 邮编：610100 t (86–28) 8481 0338 sgs.china@sgs.com

Member of the SGS Group (SGS SA)

编号	检测项目	单位	CAS No.	检测结果 1	定量限
522	四氟醚唑（氟醚唑）	mg/kg	112281-77-3	ND	0.01
523	三氯杀螨砜	mg/kg	116-29-0	ND	0.01
524	胺菊酯	mg/kg	7696-12-0	ND	0.01
525	甲基立枯磷	mg/kg	57018-04-9	ND	0.01
526	甲苯氟磺胺	mg/kg	731-27-1	ND	0.02
527	六六六总量（α-六六六,β-六六六,γ-六六六,δ-六六六）	mg/kg	319-84-6 /319-85-7 /319-86-8 /58-89-9	ND	-
528	（顺式-氯丹,反式-氯丹）总量	mg/kg	-	ND	-
529	滴滴涕总量（o,p'-滴滴涕,p,p'-滴滴滴,p,p'-滴滴伊,p,p'-滴滴涕）	mg/kg	-	ND	-
530	硫丹总量 （α-硫丹,β-硫丹和硫丹硫酸酯之和）	mg/kg	1031-07-8 /33213-65-9 /959-98-8	ND	-
531	七氯和环氧七氯总量	mg/kg	-	ND	-
532	#氟氯氰菊酯和高效氟氯氰菊酯	mg/kg	1820573-27-0 /68359-37-5	ND	0.01
533	#氯氟氰菊酯和高效氯氟氰菊酯	mg/kg	68085-85-8 /91465-08-6	ND	0.01
534	反式氯丹	mg/kg	5103-74-2	ND	0.01
535	野麦畏	mg/kg	2303-17-5	ND	0.01
536	氟乐灵	mg/kg	1582-09-8	ND	0.01
537	乙烯菌核利	mg/kg	50471-44-8	ND	0.01
538	α-六六六	mg/kg	319-84-6	ND	0.01
539	α-硫丹	mg/kg	959-98-8	ND	0.01
540	β-六六六	mg/kg	319-85-7	ND	0.01
541	β-硫丹	mg/kg	33213-65-9	ND	0.01
542	δ-六六六	mg/kg	319-86-8	ND	0.01
543	氟胺氰菊酯	mg/kg	102851-06-9	ND	0.01
544	苯线磷	mg/kg	22224-92-6	ND	0.05
545	氯氰菊酯	mg/kg	52315-07-8	ND	0.01
546	#二甲吩草胺	mg/kg	87674-68-8	ND	0.01
547	#水胺硫磷	mg/kg	24353-61-5	ND	0.01
548	p,p'-三氯杀螨醇	mg/kg	115-32-2	ND	0.01
549	#（甲拌磷,甲拌磷砜,甲拌磷亚砜）总量	mg/kg	-	ND	-
550	#氯唑磷	mg/kg	42509-80-8	ND	0.01
551	#甲基乙拌磷（二甲基硫吸磷）	mg/kg	640-15-3	ND	0.01
552	#乙拌磷	mg/kg	298-04-4	ND	0.01
553	#氯苯嘧啶醇	mg/kg	60168-88-9	ND	0.01
554	#乙草胺	mg/kg	34256-82-1	ND	0.01

通标标准技术服务有限公司四川分公司 检验检测专用章 Inspection & Testing Services
通标标准技术服务有限公司四川分公司 农产食品实验室

除非另有书面协议，本报告由本公司依据《 服务通用条款 》出具。
《 服务通用条款 》印刷在正本报告纸背面，或通过 https://www.sgs.com/en/Terms-and-Conditions 查询。请特别关注其中涉及责任限定，赔偿以及司法管辖的相关条款。任何报告的持有方需知悉，此报告内容仅反映SGS受限于客户指示下，且在当时所得结论。SGS仅对其客户负责，并且此报告不能免除交易各方根据交易文件所享有的权利和应履行的义务。未获得本公司书面批准，本文件不得进行复制，全文除外。任何未经授权对报告内容及形式进行的修改、伪造或扭曲都是违法行为，违法者将会被追究法律责任。除非另有声明，本测试报告所示结果仅涉及受测试样品。
注意：检测/检验报告或证书的真实性，请通过电话(86－755) 83071443或邮箱 CN.Doccheck@sgs.com 查询。

No.1, 5/F., Building D1, No. 1666, Section 2 of Chenglong Road, Economic and Technological Development Zone, Longquanyi District, Chengdu, Sichuan, China 610100 t (86–28) 8481 0338 www.sgsgroup.com.cn
中国・四川・成都市龙泉驿区成都经济技术开发区成龙大道二段1666号 D1栋05层1号 邮编：610100 t (86–28) 8481 0338 sgs.china@sgs.com

Member of the SGS Group (SGS SA)

编号	检测项目	单位	CAS No.	检测结果 1	定量限
555	#敌瘟磷（敌瘟散）	mg/kg	17109-49-8	ND	0.01
556	#噻唑硫磷（噻唑磷）	mg/kg	98886-44-3	ND	0.01
557	#敌稗	mg/kg	709-98-8	ND	0.01
558	#蝇毒磷	mg/kg	56-72-4	ND	0.01
559	#氟喹唑	mg/kg	136426-54-5	ND	0.01
560	#苯硫威	mg/kg	62850-32-2	ND	0.01
561	#吡草醚（霸草灵）	mg/kg	129630-19-9	ND	0.01
562	#吡唑硫磷	mg/kg	77458-01-6	ND	0.01
563	#丙草胺	mg/kg	51218-49-6	ND	0.01
564	#丙烯菊酯	mg/kg	584-79-2	ND	0.01
565	#丁草敌（丁草特）	mg/kg	2008-41-5	ND	0.01
566	#丁基嘧啶磷	mg/kg	96182-53-5	ND	0.01
567	#恶唑磷	mg/kg	18854-01-8	ND	0.01
568	#氟啶草酮	mg/kg	59756-60-4	ND	0.01
569	#胺氟草酯（氟烯草酸）	mg/kg	87546-18-7	ND	0.01
570	#环嗪酮	mg/kg	51235-04-2	ND	0.01
571	#甲基异内吸磷	mg/kg	919-86-8	ND	0.01
572	#解草酯（解毒喹）	mg/kg	99607-70-2	ND	0.01
573	#灭锈胺	mg/kg	55814-41-0	ND	0.01
574	#扑灭津	mg/kg	139-40-2	ND	0.01
575	#双苯酰草胺	mg/kg	957-51-7	ND	0.01
576	#特草定	mg/kg	5902-51-2	ND	0.01
577	#特丁净（特丁草净）	mg/kg	886-50-0	ND	0.01
578	#土菌灵	mg/kg	2593-15-9	ND	0.01
579	#吲熟酯	mg/kg	27512-72-7	ND	0.01
580	#特丁津	mg/kg	5915-41-3	ND	0.01
581	#甲拌磷亚砜	mg/kg	2588-03-6	ND	0.01
582	#甲拌磷砜	mg/kg	2588-04-7	ND	0.01
583	#丙酯杀螨醇	mg/kg	5836-10-2	ND	0.01
584	#燕麦灵	mg/kg	101-27-9	ND	0.05
585	#氟除草醚	mg/kg	13738-63-1	ND	0.01
586	#三氟硝草醚	mg/kg	15457-05-3	ND	0.01
587	#绿谷隆	mg/kg	1746-81-2	ND	0.01
588	#草枯醚	mg/kg	1836-77-7	ND	0.01
589	#百菌清	mg/kg	1897-45-6	ND	0.01
590	#螺甲螨酯	mg/kg	283594-90-1	ND	0.01
591	#烯虫酯	mg/kg	40596-69-8	ND	0.01
592	#甲羧除草醚	mg/kg	42576-02-3	ND	0.01
593	#野燕枯（硫酸二甲酯）	mg/kg	43222-48-6	ND	0.01
594	#灭菌磷	mg/kg	5131-24-8	ND	0.01

检验检测专用章
Inspection & Testing Services
通标标准技术服务有限公司四川分公司
农产食品实验室

除非另有书面协议，本报告由本公司依据《 服务通用条款 》出具。
《 服务通用条款 》印刷在正本报告纸背面，或通过 https://www.sgs.com/en/Terms-and-Conditions 查询。请特别关注其中涉及责任限定，赔偿以及司法管辖的相关条款。任何报告的持有方需知悉，此报告内容仅反映SGS受限于客户指示下，且在当时所得结论。SGS仅对其客户负责，并且此报告不能免除交易各方根据交易文件所享有的权利和应履行的义务。未获得本公司书面批准，本文件不得进行复制，全文除外。任何未经授权对报告内容及形式进行的修改、伪造或扭曲都是违法行为，违法者将会被追究法律责任。除非另有声明，本测试报告所示结果仅涉及受测试样品。
注意：检测/检验报告或证书的真实性，请通过电话(86－755) 83071443或邮箱 CN.Doccheck@sgs.com 查询。

No.1, 5F., Building D1, No. 1666, Section 2 of Chenglong Road, Economic and Technological Development Zone, Longquanyi District, Chengdu, Sichuan, China 610100 t (86–28) 8481 0338 www.sgsgroup.com.cn
中国・四川・成都市龙泉驿区经济技术开发区成龙大道二段1666号 D1栋05层1号 邮编：610100 t (86–28) 8481 0338 sgs.china@sgs.com

Member of the SGS Group (SGS SA)

检测报告

CDF25-0005759-01 发布日期：2025-10-22

编号	检测项目	单位	CAS No.	检测结果<br>1	定量限
595	#氯氰菊酯&Zeta-氯氰菊酯	mg/kg	-	ND	0.01
596	#苯草醚	mg/kg	74070-46-5	ND	0.01
597	#巴毒磷	mg/kg	7700-17-6	ND	0.01
598	#乙羧氟草醚	mg/kg	77501-90-7	ND	0.01
599	#敌噁磷（敌杀磷）	mg/kg	78-34-2	ND	0.01
600	#联苯	mg/kg	92-52-4	ND	0.01
601	#二丙烯草胺	mg/kg	93-71-0	ND	0.01
602	#格螨酯	mg/kg	97-16-5	ND	0.01

备注:
- ND = 未检出
- #检测项目不在本实验室的 CNAS 认可范围内。

注意事项：
除非另有说明，本检测结果仅与被检测物品有关。未经检验机构书面同意，委托人不得擅自使用检测结果进行不当宣传。本检测报告仅作为科研、教学或内部质量控制之用。未经检验机构批准，不得复制（全文复制除外）报告。

结束

除非另有书面协议，本报告由本公司依据《服务通用条款》出具。
《服务通用条款》印刷在正本报告纸背面，或通过 https://www.sgs.com/en/Terms-and-Conditions 查询。请特别关注其中涉及责任限定，赔偿以及司法管辖的相关条款。任何报告的持有方需知悉，此报告内容仅反映SGS受限于客户指示下，且在当时所得结论。SGS仅对其客户负责，并且此报告不能免除交易各方根据交易文件所享有的权利和应履行的义务。未获得本公司书面批准，本文件不得进行复制，全文除外。任何未经授权对报告内容及形式进行的修改、伪造或扭曲都是违法行为，违法者将会被追究法律责任。除非另有声明，本测试报告所示结果仅涉及受测试样品。
注意：检测/检验报告或证书的真实性，请通过电话 (86-755) 83071443 或邮箱 CN.Doccheck@sgs.com 查询。

No.1, 5/F., Building D1, No. 1666, Section 2 of Chenglong Road, Economic and Technological Development Zone, Longquanyi District, Chengdu, Sichuan, China 610100 t (86–28) 8481 0338 www.sgsgroup.com.cn
中国・四川・成都市龙泉驿区经济技术开发区成龙大道二段1666号 D1栋05层1号 邮编: 610100 t (86–28) 8481 0338 sgs.china@sgs.com

Member of the SGS Group (SGS SA)

通标标准技术服务有限公司四川分公司
农产品食品实验室

检测报告

CDF25-0001880-01 发布日期：2025-05-06

客户名称： 西双版纳华坤生物科技有限责任公司

客户地址： 景洪市嘎洒镇曼迈村委会曼景栋村

样品名称： 橡胶树种子油

样品批号： /

生产日期： 20250423

生产商： 西双版纳华坤生物科技有限责任公司

以上样品及信息由客户提供及确认, SGS 不承担证实客户提供信息的准确性、适当性和(或)完整性责任。

样品接收日期： 2025-04-27

检测周期： 2025-04-27 ~ 2025-05-06

检测要求： 根据客户要求检测

检测方法： 请参见下一页。

检测结果： 请参见下一页。

SGS 授权签字人

扫码查看在线报告
CDF25-0001880-01
报告验真请访问：
check.sgsonline.com.cn

除非另有书面协议，本报告由本公司依据《 服务通用条款 》出具。
《 服务通用条款 》印刷在正本报告纸背面，或通过 https://www.sgs.com/en/Terms-and-Conditions 查询。请特别关注其中涉及责任限定，赔偿以及司法管辖的相关条款。任何报告的持有方需知悉，此报告内容仅反映SGS受限于客户指示下，且在当时所得结论。SGS仅对其客户负责，并且此报告不能免除交易各方根据交易文件所享有的权利和应履行的义务。未获得本公司书面批准，本文件不得进行复制，全文除外。任何未经授权对报告内容及形式进行的修改、伪造或扭曲都是违法行为，违法者将会被追究法律责任。除非另有声明，本测试报告所示结果仅涉及受测试样品。
注意：检测/检验报告或证书的真实性，请通过电话 (86－755) 83071443 或邮箱 CN.Doccheck@sgs.com 查询。

No.1, 5/F., Building D1, No. 1666, Section 2 of Chenglong Road, Economic and Technological Development Zone, Longquanyi District, Chengdu, Sichuan, China 610100 t (86–28) 8481 0338 www.sgsgroup.com.cn
中国・四川・成都市龙泉驿区经济技术开发区成龙大道二段1666号 D1栋05层1号 邮编：610100 t (86–28) 8481 0338 sgs.china@sgs.com

Member of the SGS Group (SGS SA)

检测报告

CDF25-0001880-01　　　　发布日期：2025-05-06

检测样品描述：

样品编号	SGS 样品 ID	样品描述
1	CDF25-0001880-0001	瓶装样品 1 瓶

检测结果：

农药残留

检测方法： BS EN 15662:2018
EN 12393-2013

检测结果：

编号	检测项目	单位	CAS No.	检测结果 1	定量限
1	#联苯	mg/kg	92-52-4	ND	0.01
2	#混杀威（3,4,5-混杀威）	mg/kg	2686-99-9	ND	0.01
3	阿维菌素	mg/kg	71751-41-2	ND	0.01
4	乙酰甲胺磷	mg/kg	30560-19-1	ND	0.01
5	啶虫脒	mg/kg	135410-20-7	ND	0.01
6	#活化酯/苯并噻二唑	mg/kg	135158-54-2	ND	0.01
7	涕灭威	mg/kg	116-06-3	ND	0.01
8	#砜灭威（涕灭威砜）	mg/kg	1646-88-4	ND	0.01
9	#涕灭威亚砜	mg/kg	1646-87-3	ND	0.01
10	#酰嘧磺隆	mg/kg	120923-37-7	ND	0.01
11	#吲唑磺菌胺	mg/kg	348635-87-0	ND	0.05
12	#莎稗磷	mg/kg	64249-01-0	ND	0.01
13	#杀螨特	mg/kg	140-57-8	ND	0.01
14	#莠去津	mg/kg	1912-24-9	ND	0.01
15	#氧环唑	mg/kg	60207-31-0	ND	0.01
16	#甲基吡恶磷	mg/kg	35575-96-3	ND	0.01
17	谷硫磷（保棉磷）	mg/kg	86-50-0	ND	0.01
18	嘧菌酯（腈嘧菌酯）	mg/kg	131860-33-8	ND	0.01
19	苯霜灵	mg/kg	71626-11-4	ND	0.01
20	#解草嗪	mg/kg	98730-04-2	ND	0.01
21	#苄嘧磺隆	mg/kg	83055-99-6	ND	0.01
22	#地散磷	mg/kg	741-58-2	ND	0.01
23	#灭草松	mg/kg	25057-89-0	ND	0.01
24	#苯螨特	mg/kg	29104-30-1	ND	0.01
25	联苯三唑醇	mg/kg	55179-31-2	ND	0.01
26	啶酰菌胺	mg/kg	188425-85-6	ND	0.01
27	#除草定	mg/kg	314-40-9	ND	0.01
28	#溴丁酰草胺	mg/kg	74712-19-9	ND	0.01

通标标准技术服务有限公司四川分公司 检验检测专用章 Inspection & Testing Services

除非另有书面协议，本报告由本公司依据《 服务通用条款 》出具。
《 服务通用条款 》印刷在正本报告纸背面，或通过 https://www.sgs.com/en/Terms-and-Conditions 查询。请特别关注其中涉及责任限定，赔偿以及司法管辖的相关条款。任何报告的持有方需知悉，此报告内容仅反映SGS受限于客户指示下，且在当时所得结论。SGS仅对其客户负责，并且此报告不能免除交易各方根据交易文件所享有的权利和应履行的义务。未获得本公司书面批准，本文件不得进行复制，全文除外。任何未经授权对报告内容及形式进行的修改、伪造或扭曲都是违法行为，违法者将会被追究法律责任。除非另有声明，本测试报告所示结果仅涉及受测试样品。
注意：检测/检验报告或证书的真实性，请通过电话(86－755) 83071443或邮箱 CN.Doccheck@sgs.com查询。

No.1, 5/F, Building D1, No. 1666, Section 2 of Chenglong Road, Economic and Technological Development Zone, Longquanyi District, Chengdu, Sichuan, China 610100 t (86–28) 8481 0338 www.sgsgroup.com.cn
中国・四川・成都市龙泉驿区经济技术开发区成龙大道二段1666号 D1栋05层1号 邮编：610100 t (86–28) 8481 0338 sgs.china@sgs.com

Member of the SGS Group (SGS SA)

编号	检测项目	单位	CAS No.	检测结果 1	定量限
29	溴苯腈	mg/kg	1689-84-5	ND	0.01
30	乙嘧酚磺酸酯	mg/kg	41483-43-6	ND	0.01
31	噻嗪酮	mg/kg	69327-76-0	ND	0.01
32	#氟丙嘧草酯	mg/kg	134605-64-4	ND	0.01
33	#丁酮威	mg/kg	34681-10-2	ND	0.01
34	#丁酮砜威	mg/kg	34681-23-7	ND	0.01
35	甲萘威	mg/kg	63-25-2	ND	0.01
36	多菌灵	mg/kg	10605-21-7	ND	0.01
37	呋喃丹（虫螨威/克百威）	mg/kg	1563-66-2	ND	0.01
38	3-羟基虫螨威（3-羟基克百威）	mg/kg	16655-82-6	ND	0.01
39	萎锈灵	mg/kg	5234-68-4	ND	0.01
40	#唑草酮	mg/kg	128639-02-1	ND	0.01
41	#加普胺	mg/kg	104030-54-8	ND	0.01
42	#氯虫苯甲酰胺	mg/kg	500008-45-7	ND	0.01
43	#灭幼脲	mg/kg	57160-47-1	ND	0.01
44	#氯草灵	mg/kg	1967-16-4	ND	0.01
45	#杀虫脒	mg/kg	6164-98-3	ND	0.01
46	#氟啶脲	mg/kg	71422-67-8	ND	0.01
47	#氯草敏	mg/kg	1698-60-8	ND	0.01
48	#氯嘧磺隆	mg/kg	90982-32-4	ND	0.01
49	#绿麦隆	mg/kg	15545-48-9	ND	0.01
50	#枯草隆	mg/kg	1982-47-4	ND	0.01
51	#氯磺隆	mg/kg	64902-72-3	ND	0.01
52	#环虫酰肼	mg/kg	143807-66-3	ND	0.01
53	#吲哚酮草酯	mg/kg	142891-20-1	ND	0.01
54	#环庚草醚	mg/kg	87818-31-3	ND	0.01
55	#醚磺隆	mg/kg	94593-91-6	ND	0.01
56	#烯草酮	mg/kg	99129-21-2	ND	0.01
57	四螨嗪	mg/kg	74115-24-5	ND	0.01
58	#氯甲酰草胺	mg/kg	84496-56-0	ND	0.01
59	#调果酸（调采酸）	mg/kg	101-10-0	ND	0.01
60	#氯酯磺草胺	mg/kg	147150-35-4	ND	0.01
61	噻虫胺（可尼丁）	mg/kg	210880-92-5	ND	0.01
62	#氰草津	mg/kg	21725-46-2	ND	0.01
63	氰霜唑	mg/kg	120116-88-3	ND	0.01
64	#环丙酸酰胺	mg/kg	113136-77-9	ND	0.01
65	#环草敌（草灭特）	mg/kg	1134-23-2	ND	0.01
66	#环丙嘧磺隆	mg/kg	136849-15-5	ND	0.01
67	#环氟菌胺	mg/kg	180409-60-3	ND	0.01
68	霜脲氰	mg/kg	57966-95-7	ND	0.01

除非另有书面协议，本报告由本公司依据《服务通用条款》出具。《服务通用条款》印刷在正本报告纸背面，或通过 https://www.sgs.com/en/Terms-and-Conditions 查询。请特别关注其中涉及责任限定，赔偿以及司法管辖的相关条款。任何报告的持有方需知悉，此报告内容仅反映SGS受限于客户指示下，且在当时所得结论。SGS仅对其客户负责，并且此报告不能免除交易各方根据交易文件所享有的权利和应履行的义务。未获得本公司书面批准，本文件不得进行复制，全文除外。任何未经授权对报告内容及形式进行的修改、伪造或扭曲都是违法行为，违法者将会被追究法律责任。除非另有声明，本测试报告所示结果仅涉及受测试样品。
注意：检测/检验报告或证书的真实性，请通过电话(86-755) 83071443或邮箱 CN.Doccheck@sgs.com查询。
No.1, 5F, Building D1, No. 1666, Section 2 of Chenglong Road, Economic and Technological Development Zone, Longquanyi District, Chengdu, Sichuan, China 610100 t (86-28) 8481 0338 www.sgsgroup.com.cn
中国・四川・成都市龙泉驿区经济技术开发区成龙大道二段1666号 D1栋05层1号 邮编：610100 t (86-28) 8481 0338 sgs.china@sgs.com
Member of the SGS Group (SGS SA)

编号	检测项目	单位	CAS No.	检测结果 1	定量限
69	嘧菌环胺	mg/kg	121552-61-2	ND	0.01
70	#杀草隆	mg/kg	42609-52-9	ND	0.01
71	#内吸磷	mg/kg	8065-48-3	ND	0.01
72	甲基砜内吸磷	mg/kg	17040-19-6	ND	0.01
73	#亚砜磷	mg/kg	301-12-2	ND	0.01
74	#甜菜胺（甜菜安）	mg/kg	13684-56-5	ND	0.01
75	#丁醚脲（丁嗪脲）	mg/kg	80060-09-9	ND	0.01
76	#燕麦敌（丁变敌）	mg/kg	2303-16-4	ND	0.01
77	#2,4-滴丙酸	mg/kg	120-36-5	ND	0.01
78	#双氯氰菌胺	mg/kg	139920-32-4	ND	0.01
79	#双氯磺草胺	mg/kg	145701-21-9	ND	0.01
80	百治磷	mg/kg	141-66-2	ND	0.01
81	乙霉威	mg/kg	87130-20-9	ND	0.01
82	苯醚甲环唑	mg/kg	119446-68-3	ND	0.01
83	除虫脲	mg/kg	35367-38-5	ND	0.01
84	#吡氟酰草胺	mg/kg	83164-33-4	ND	0.01
85	#哌草丹	mg/kg	61432-55-1	ND	0.01
86	#异戊乙净（阔草净）	mg/kg	22936-75-0	ND	0.01
87	#二甲嘧酚	mg/kg	5221-53-4	ND	0.01
88	乐果	mg/kg	60-51-5	ND	0.01
89	烯酰吗啉	mg/kg	110488-70-5	ND	0.01
90	烯唑醇	mg/kg	83657-24-3	ND	0.01
91	#敌螨普	mg/kg	39300-45-3	ND	0.01
92	#丁诺特呋喃（呋虫胺）	mg/kg	165252-70-0	ND	0.01
93	#特乐酚	mg/kg	1420-07-1	ND	0.01
94	敌草隆	mg/kg	330-54-1	ND	0.01
95	#甲氨基阿维菌素苯甲酸盐	mg/kg	155569-91-8	ND	0.01
96	#苯氧菌胺	mg/kg	133408-50-1	ND	0.01
97	氟环唑	mg/kg	106325-08-0 /133855-98-8	ND	0.01
98	#戊草丹	mg/kg	85785-20-2	ND	0.01
99	#乙硫苯威	mg/kg	29973-13-5	ND	0.01
100	#乙硫苯威砜	mg/kg	53380-23-7	ND	0.01
101	#乙硫苯威亚砜	mg/kg	53380-22-6	ND	0.01
102	#乙虫清	mg/kg	181587-01-9	ND	0.01
103	#乙嘧酚	mg/kg	23947-60-6	ND	0.01
104	#乙氧嘧磺隆	mg/kg	126801-58-9	ND	0.01
105	#乙氧苯草胺	mg/kg	79540-50-4	ND	0.01
106	#乙螨唑	mg/kg	153233-91-1	ND	0.01
107	咪唑菌酮	mg/kg	161326-34-7	ND	0.01

除非另有书面协议，本报告由本公司依据《 服务通用条款 》出具。
《 服务通用条款 》印刷在正本报告纸背面，或通过 https://www.sgs.com/en/Terms-and-Conditions 查询。请特别关注其中涉及责任限定，赔偿以及司法管辖的相关条款。任何报告的持有方需知悉，此报告内容仅反映SGS受限于客户指示下，且在当时所得结论。SGS仅对其客户负责，并且此报告不能免除交易各方根据交易文件所享有的权利和应履行的义务。未获得本公司书面批准，本文件不得进行复制，全文除外。任何未经授权对报告内容及形式进行的修改、伪造或扭曲都是违法行为，违法者将会被追究法律责任。除非另有声明，本测试报告所示结果仅涉及受测试样品。
注意：检测/检验报告或证书的真实性，请通过电话 (86－755) 83071443 或邮箱 CN.Doccheck@sgs.com 查询。
No.1, 5/F., Building D1, No. 1666, Section 2 of Chenglong Road, Economic and Technological Development Zone, Longquanyi District, Chengdu, Sichuan, China 610100 t (86–28) 8481 0338 www.sgsgroup.com.cn
中国・四川・成都市龙泉驿区经济技术开发区成龙大道二段1666号 D1栋05层1号 邮编：610100 t (86–28) 8481 0338 sgs.china@sgs.com
Member of the SGS Group (SGS SA)

编号	检测项目	单位	CAS No.	检测结果 1	定量限
108	喹螨醚	mg/kg	120928-09-8	ND	0.01
109	腈苯唑	mg/kg	114369-43-6	ND	0.01
110	#甲呋酰胺	mg/kg	24691-80-3	ND	0.01
111	环酰菌胺	mg/kg	126833-17-8	ND	0.01
112	#仲丁威	mg/kg	3766-81-2	ND	0.01
113	#稻瘟酰胺（氰菌胺）	mg/kg	115852-48-7	ND	0.01
114	#恶唑禾草灵	mg/kg	66441-23-4	ND	0.01
115	双氧威（苯醚威）	mg/kg	72490-01-8 /79127-80-3	ND	0.01
116	苯锈啶	mg/kg	67306-00-7	ND	0.01
117	丁苯吗啉	mg/kg	67564-91-4	ND	0.01
118	唑螨酯	mg/kg	111812-58-9	ND	0.01
119	倍硫磷	mg/kg	55-38-9	ND	0.01
120	#倍硫磷砜	mg/kg	3761-42-0	ND	0.01
121	#倍硫磷亚砜	mg/kg	3761-41-9	ND	0.01
122	#四唑酰草胺（四唑啉酮）	mg/kg	158237-07-1	ND	0.01
123	#嘧菌腙	mg/kg	89269-64-7	ND	0.01
124	氟虫腈	mg/kg	120068-37-3	ND	0.01
125	#氟虫腈亚砜	mg/kg	120067-83-6	ND	0.01
126	#氟虫腈砜	mg/kg	120068-36-2	ND	0.01
127	#甲基麦草氟异丙酯（麦草氟甲酯）	mg/kg	52756-25-9	ND	0.01
128	#啶嘧磺隆	mg/kg	104040-78-0	ND	0.01
129	氟啶虫酰胺	mg/kg	158062-67-0	ND	0.01
130	#双氟磺草胺	mg/kg	145701-23-1	ND	0.01
131	#吡氟禾草灵和精吡氟禾草灵	mg/kg	69806-50-4 /79241-46-6	ND	0.01
132	#氟啶胺	mg/kg	79622-59-6	ND	0.01
133	#氟虫双酰胺（氟苯虫酰胺）	mg/kg	272451-65-7	ND	0.01
134	#氟唑磺隆	mg/kg	181274-17-9	ND	0.01
135	咯菌腈	mg/kg	131341-86-1	ND	0.01
136	#氟噻草胺	mg/kg	142459-58-3	ND	0.01
137	氟虫脲	mg/kg	101463-69-8	ND	0.01
138	#丁虫腈	mg/kg	704886-18-0	ND	0.01
139	#唑嘧磺草胺	mg/kg	98967-40-9	ND	0.01
140	#丙炔氟草胺	mg/kg	103361-09-7	ND	0.02
141	#氟吗啉	mg/kg	211867-47-9	ND	0.01
142	#伏草隆（氟草隆）	mg/kg	2164-17-2	ND	0.01
143	氟吡菌胺	mg/kg	239110-15-7	ND	0.01
144	#氯氟吡氧乙酸异辛酯	mg/kg	81406-37-3	ND	0.01
145	#磺菌胺	mg/kg	106917-52-6	ND	0.01
146	#氟噻乙草酯（氟赛乙草酯）	mg/kg	117337-19-6	ND	0.05

除非另有书面协议，本报告由本公司依据《服务通用条款》出具。
《服务通用条款》印刷在正本报告纸背面，或通过 https://www.sgs.com/en/Terms-and-Conditions 查询。请特别关注其中涉及责任限定，赔偿以及司法管辖的相关条款。任何报告的持有方需知悉，此报告内容仅反映SGS受限于客户指示下，且在当时所得结论。SGS仅对其客户负责，并且此报告不能免除交易各方根据交易文件所享有的权利和应履行的义务。未获得本公司书面批准，本文件不得进行复制，全文除外。任何未经授权对报告内容及形式进行的修改、伪造或扭曲都是违法行为，违法者将会被追究法律责任。除非另有声明，本测试报告所示结果仅涉及受测试样品。
注意：检测/检验报告或证书的真实性，请通过电话 (86-755) 83071443 或邮箱 CN.Doccheck@sgs.com 查询。

No.1, 5F, Building D1, No. 1666, Section 2 of Chenglong Road, Economic and Technological Development Zone, Longquanyi District, Chengdu, Sichuan, China 610100 t (86–28) 8481 0338 www.sgsgroup.com.cn
中国・四川・成都市龙泉驿区经济技术开发区成龙大道二段1666号 D1栋05层1号 邮编：610100 t (86–28) 8481 0338 sgs.china@sgs.com

Member of the SGS Group (SGS SA)

检测报告

CDF25-0001880-01　　发布日期：2025-05-06

编号	检测项目	单位	CAS No.	检测结果 1	定量限
147	粉唑醇	mg/kg	76674-21-0	ND	0.01
148	#氟磺胺草醚	mg/kg	72178-02-0	ND	0.01
149	#氯吡脲	mg/kg	68157-60-8	ND	0.01
150	#福拉比（呋比菌胺）	mg/kg	123572-88-3	ND	0.01
151	#呋线威	mg/kg	65907-30-4	ND	0.01
152	#氯吡嘧磺隆	mg/kg	100784-20-1	ND	0.01
153	#氟吡禾灵	mg/kg	69806-34-4	ND	0.01
154	#氟吡乙禾灵	mg/kg	87237-48-7	ND	0.01
155	#氟吡甲禾灵	mg/kg	69806-40-2	ND	0.01
156	己唑醇	mg/kg	79983-71-4	ND	0.01
157	#氟铃脲	mg/kg	86479-06-3	ND	0.01
158	噻螨酮	mg/kg	78587-05-0	ND	0.01
159	抑霉唑（烯菌灵）	mg/kg	35554-44-0	ND	0.01
160	#醚草酸甲酯	mg/kg	81405-85-8	ND	0.01
161	#甲氧咪草烟	mg/kg	114311-32-9	ND	0.01
162	#甲咪唑烟酸	mg/kg	104098-48-8	ND	0.01
163	#咪唑喹啉酸（灭草喹）	mg/kg	81335-37-7	ND	0.01
164	#亚胺唑	mg/kg	86598-92-7	ND	0.01
165	吡虫啉	mg/kg	138261-41-3	ND	0.01
166	#氯噻啉	mg/kg	105843-36-5	ND	0.01
167	#茚草酮	mg/kg	133220-30-1	ND	0.01
168	茚虫威（安打）	mg/kg	173584-44-6	ND	0.01
169	#甲基碘磺隆（钠盐）	mg/kg	144550-36-7	ND	0.01
170	#碘苯腈	mg/kg	1689-83-4	ND	0.01
171	#异稻瘟净	mg/kg	26087-47-8	ND	0.01
172	异菌脲	mg/kg	36734-19-7	ND	0.01
173	缬霉威	mg/kg	140923-17-7	ND	0.01
174	#异丙威	mg/kg	2631-40-5	ND	0.01
175	#稻瘟灵	mg/kg	50512-35-1	ND	0.01
176	#异丙隆	mg/kg	34123-59-6	ND	0.01
177	#异唑隆（异恶隆）	mg/kg	55861-78-4	ND	0.01
178	#双苯恶唑酸	mg/kg	163520-33-0	ND	0.01
179	#异恶氟草（异恶唑草酮）	mg/kg	141112-29-0	ND	0.01
180	醚菌酯（苯氧菊酯）	mg/kg	143390-89-0	ND	0.01
181	#乳氟禾草灵	mg/kg	77501-63-4	ND	0.01
182	#环草定（环草啶）	mg/kg	2164-08-1	ND	0.01
183	利谷隆	mg/kg	330-55-2	ND	0.01
184	虱螨脲（氟丙氧脲）	mg/kg	103055-07-8	ND	0.01
185	马拉硫磷	mg/kg	121-75-5	ND	0.01
186	双炔酰菌胺	mg/kg	374726-62-2	ND	0.01

除非另有书面协议，本报告由本公司依据《服务通用条款》出具。
《服务通用条款》印刷在正本报告纸背面，或通过 https://www.sgs.com/en/Terms-and-Conditions 查询。请特别关注其中涉及责任限定，赔偿以及司法管辖的相关条款。任何报告的持有方需知悉，此报告内容仅反映SGS受限于客户指示下，且在当时所得结论。SGS仅对其客户负责，并且此报告不能免除交易各方根据交易文件所享有的权利和应履行的义务。未获得本公司书面批准，本文件不得进行复制，全文除外。任何未经授权对报告内容及形式进行的修改、伪造或扭曲都是违法行为，违法者将会被追究法律责任。除非另有声明，本测试报告所示结果仅涉及受测试样品。
注意：检测/检验报告或证书的真实性，请通过电话 (86－755) 83071443 或邮箱 CN.Doccheck@sgs.com 查询。

No.1, 5F, Building D1, No. 1666, Section 2 of Chenglong Road, Economic and Technological Development Zone, Longquanyi District, Chengdu, Sichuan, China 610100 t (86–28) 8481 0338 www.sgsgroup.com.cn
中国・四川・成都市龙泉驿区经济技术开发区成龙大道二段1666号 D1栋05层1号 邮编: 610100 t (86–28) 8481 0338 sgs.china@sgs.com

Member of the SGS Group (SGS SA)

通标标准技术服务有限公司四川分公司 检验检测专用章 Inspection & Testing Services

编号	检测项目	单位	CAS No.	检测结果 1	定量限
187	#2 甲 4 氯	mg/kg	94-74-6	ND	0.01
188	#苯噻酰草胺	mg/kg	73250-68-7	ND	0.01
189	#吡咯解草酯（吡咯二酸二乙酯）	mg/kg	135590-91-9	ND	0.01
190	嘧菌胺	mg/kg	110235-47-7	ND	0.01
191	#甲基二磺隆	mg/kg	208465-21-8	ND	0.01
192	#硝磺草酮	mg/kg	104206-82-8	ND	0.01
193	#甲霜灵和精甲霜灵	mg/kg	57837-19-1 /70630-17-0	ND	0.01
194	甲霜灵（阿普隆）（瑞毒霜）	mg/kg	57837-19-1	ND	0.01
195	苯嗪草酮（灭它通）	mg/kg	41394-05-2	ND	0.01
196	#叶菌唑	mg/kg	125116-23-6	ND	0.01
197	#甲草苯噻隆（甲草苯隆）	mg/kg	18691-97-9	ND	0.01
198	甲胺磷	mg/kg	10265-92-6	ND	0.01
199	杀扑磷	mg/kg	950-37-8	ND	0.01
200	甲硫威（灭虫威）	mg/kg	2032-65-7	ND	0.01
201	灭多威	mg/kg	16752-77-5	ND	0.01
202	甲氧虫酰肼	mg/kg	161050-58-4	ND	0.05
203	异丙甲草胺	mg/kg	51218-45-2	ND	0.01
204	#速灭威	mg/kg	1129-41-5	ND	0.01
205	苯菌酮	mg/kg	220899-03-6	ND	0.01
206	#甲磺隆	mg/kg	74223-64-6	ND	0.05
207	#禾草敌	mg/kg	2212-67-1	ND	0.01
208	久效磷	mg/kg	6923-22-4	ND	0.01
209	腈菌唑（灭克落）	mg/kg	88671-89-0	ND	0.01
210	#萘草胺	mg/kg	132-66-1	ND	0.01
211	#烟嘧磺隆	mg/kg	111991-09-4	ND	0.01
212	#氟草敏（哒草伏）	mg/kg	27314-13-2	ND	0.01
213	#氟酰脲	mg/kg	116714-46-6	ND	0.01
214	#呋酰胺	mg/kg	58810-48-3	ND	0.01
215	氧乐果	mg/kg	1113-02-6	ND	0.01
216	#嘧苯胺磺隆	mg/kg	213464-77-8	ND	0.01
217	#丙炔噁草酮	mg/kg	39807-15-3	ND	0.01
218	恶草酮	mg/kg	19666-30-9	ND	0.01
219	恶霜灵	mg/kg	77732-09-3	ND	0.01
220	杀线威	mg/kg	23135-22-0	ND	0.01
221	#氯恶嗪草	mg/kg	153197-14-9	ND	0.01
222	#氧化萎锈灵	mg/kg	5259-88-1	ND	0.01
223	纹枯脲（戊菌隆）	mg/kg	66063-05-6	ND	0.01
224	#五氟磺草胺	mg/kg	219714-96-2	ND	0.01
225	甜菜宁	mg/kg	13684-63-4	ND	0.01

通标标准技术服务有限公司四川分公司 检验检测专用章 Inspection & Testing Services

除非另有书面协议，本报告由本公司依据《服务通用条款》出具。
《服务通用条款》印刷在正本报告纸背面，或通过 https://www.sgs.com/en/Terms-and-Conditions 查询。请特别关注其中涉及责任限定，赔偿以及司法管辖的相关条款。任何报告的持有方需知悉，此报告内容仅反映SGS受限于客户指示下，且在当时所得结论。SGS仅对其客户负责，并且此报告不能免除交易各方根据交易文件所享有的权利和应履行的义务。未获得本公司书面批准，本文件不得进行复制，全文除外。任何未经授权对其报告内容及形式进行的修改、伪造或扭曲都是违法行为，违法者将会被追究法律责任。除非另有声明，本测试报告所示结果仅涉及受测试样品。
注意：检测/检验报告或证书的真实性，请通过电话 (86-755) 83071443 或邮箱 CN.Doccheck@sgs.com 查询。
No.1, 5F, Building D1, No. 1666, Section 2 of Chenglong Road, Economic and Technological Development Zone, Longquanyi District, Chengdu, Sichuan, China 610100 t (86–28) 8481 0338 www.sgsgroup.com.cn
中国・四川・成都市龙泉驿区经济技术开发区成龙大道二段1666号 D1栋05层1号 邮编：610100 t (86–28) 8481 0338 sgs.china@sgs.com
Member of the SGS Group (SGS SA)

编号	检测项目	单位	CAS No.	检测结果 1	定量限
226	伏杀硫磷	mg/kg	2310-17-0	ND	0.01
227	#硫环磷	mg/kg	947-02-4	ND	0.01
228	#甲基硫环磷	mg/kg	5120-23-0	ND	0.01
229	亚胺硫磷	mg/kg	732-11-6	ND	0.01
230	辛硫磷	mg/kg	14816-18-3	ND	0.01
231	#氟吡草胺	mg/kg	137641-05-5	ND	0.01
232	#啶氧菌酯	mg/kg	117428-22-5	ND	0.01
233	抗蚜威	mg/kg	23103-98-2	ND	0.01
234	#烯丙苯噻唑	mg/kg	27605-76-1	ND	0.01
235	#咪鲜胺	mg/kg	67747-09-5	ND	0.01
236	#茉莉酮（茉莉酸导体）	mg/kg	158474-72-7	ND	0.01
237	#猛杀威	mg/kg	2631-37-0	ND	0.01
238	#毒草胺	mg/kg	1918-16-7	ND	0.01
239	霜霉威	mg/kg	24579-73-5	ND	0.01
240	#恶草酸	mg/kg	111479-05-1	ND	0.01
241	炔螨特	mg/kg	2312-35-8	ND	0.01
242	#异丙草胺	mg/kg	86763-47-5	ND	0.01
243	残杀威	mg/kg	114-26-1	ND	0.01
244	苄草丹	mg/kg	52888-80-9	ND	0.01
245	#氟磺隆	mg/kg	94125-34-5	ND	0.01
246	吡蚜酮（吡嗪酮）	mg/kg	123312-89-0	ND	0.01
247	吡唑醚菌酯（唑菌胺酯）（百克敏）	mg/kg	175013-18-0	ND	0.01
248	#唑菌酯	mg/kg	862588-11-2	ND	0.01
249	#吡唑特	mg/kg	58011-68-0	ND	0.01
250	#吡菌磷	mg/kg	13457-18-6	ND	0.01
251	#乙基吡嘧磺隆（百速隆）	mg/kg	93697-74-6	ND	0.01
252	#苄草唑	mg/kg	71561-11-0	ND	0.01
253	#稗草丹	mg/kg	88678-67-5	ND	0.01
254	哒螨灵	mg/kg	96489-71-3	ND	0.01
255	#啶虫丙醚	mg/kg	179101-81-6	ND	0.01
256	#哒嗪硫磷	mg/kg	119-12-0	ND	0.01
257	啶斑肟（比芬诺）	mg/kg	88283-41-4	ND	0.01
258	#环酯草醚	mg/kg	135186-78-6	ND	0.01
259	嘧霉胺	mg/kg	53112-28-0	ND	0.01
260	#嘧螨醚	mg/kg	105779-78-0	ND	0.01
261	#吡丙醚（蚊蝇醚）	mg/kg	95737-68-1	ND	0.01
262	#咯喹酮（百快隆）	mg/kg	57369-32-1	ND	0.01
263	#灭藻醌	mg/kg	2797-51-5	ND	0.01
264	喹氧灵	mg/kg	124495-18-7	ND	0.01
265	#喹禾灵和精喹禾灵	mg/kg	100646-51-3	ND	0.01

通标标准技术服务有限公司四川分公司 检验检测专用章 Inspection & Testing Services
通标标准技术服务有限公司四川分公司 农产食品实验室

除非另有书面协议，本报告由本公司依据《服务通用条款》出具。
《服务通用条款》印刷在正本报告纸背面，或通过 https://www.sgs.com/en/Terms-and-Conditions 查询。请特别关注其中涉及责任限定，赔偿以及司法管辖的相关条款。任何报告的持有方需知悉，此报告内容仅反映SGS受限于客户指示下，且在当时所得结论。SGS仅对其客户负责，并且此报告不能免除交易各方根据交易文件所享有的权利和应履行的义务。未获得本公司书面批准，本文件不得进行复制，全文除外。任何未经授权对报告内容及形式进行的修改、伪造或扭曲都是违法行为，违法者将会被追究法律责任。除非另有声明，本测试报告所示结果仅涉及受测试样品。
注意：检测/检验报告或证书的真实性，请通过电话(86－755) 83071443或邮箱 CN.Doccheck@sgs.com查询。
No.1, 5F, Building D1, No. 1666, Section 2 of Chenglong Road, Economic and Technological Development Zone, Longquanyi District, Chengdu, Sichuan, China 610100 t (86–28) 8481 0338 www.sgsgroup.com.cn
中国・四川・成都市龙泉驿区经济技术开发区成龙大道二段1666号 D1栋05层1号 邮编：610100 t (86–28) 8481 0338 sgs.china@sgs.com
Member of the SGS Group (SGS SA)

编号	检测项目	单位	CAS No.	检测结果 1	定量限
			/76578-14-8		
266	#砜嘧磺隆	mg/kg	122931-48-0	ND	0.01
267	#鱼藤酮	mg/kg	83-79-4	ND	0.01
268	#单甲脒	mg/kg	33089-74-6	ND	0.01
269	#稀禾定	mg/kg	74051-80-2	ND	0.01
270	#西玛津	mg/kg	122-34-9	ND	0.01
271	#硅氟唑	mg/kg	149508-90-7	ND	0.01
272	#西草净	mg/kg	1014-70-6	ND	0.01
273	螺螨酯	mg/kg	148477-71-8	ND	0.01
274	#螺虫乙酯	mg/kg	203313-25-1	ND	0.01
275	螺恶茂胺（螺环菌胺）	mg/kg	118134-30-8	ND	0.01
276	#磺草酮	mg/kg	99105-77-8	ND	0.01
277	#乙黄隆（磺酰磺隆）	mg/kg	141776-32-1	ND	0.05
278	#氟啶虫胺腈	mg/kg	946578-00-3	ND	0.01
279	戊唑醇	mg/kg	107534-96-3	ND	0.01
280	虫酰肼	mg/kg	112410-23-8	ND	0.01
281	吡螨胺	mg/kg	119168-77-3	ND	0.02
282	#特丁隆	mg/kg	34014-18-1	ND	0.01
283	氟苯脲	mg/kg	83121-18-0	ND	0.01
284	#噻吩草胺（甲氧噻草胺）	mg/kg	96491-05-3	ND	0.01
285	噻菌灵	mg/kg	148-79-8	ND	0.01
286	噻虫啉	mg/kg	111988-49-9	ND	0.01
287	噻虫嗪	mg/kg	153719-23-4	ND	0.01
288	#噻唑烟酸（噻草啶）	mg/kg	117718-60-2	ND	0.01
289	#赛苯隆	mg/kg	51707-55-2	ND	0.01
290	#噻吩磺隆（阔叶散）	mg/kg	79277-27-3	ND	0.01
291	#噻呋酰胺	mg/kg	130000-40-7	ND	0.01
292	#禾草丹	mg/kg	28249-77-6	ND	0.01
293	#硫双威	mg/kg	59669-26-0	ND	0.01
294	#久效威砜	mg/kg	39184-59-3	ND	0.01
295	#久效威亚砜	mg/kg	39184-27-5	ND	0.01
296	#甲基硫菌灵	mg/kg	23564-05-8	ND	0.01
297	#噻酰菌胺	mg/kg	223580-51-6	ND	0.01
298	#唑虫酰胺	mg/kg	129558-76-5	ND	0.01
299	#苯草酮（甲氧基草酮）	mg/kg	87820-88-0	ND	0.01
300	#三唑酮	mg/kg	43121-43-3	ND	0.01
301	三唑醇	mg/kg	55219-65-3	ND	0.01
302	#醚苯磺隆	mg/kg	82097-50-5	ND	0.01
303	三唑磷	mg/kg	24017-47-8	ND	0.01
304	#苯磺隆	mg/kg	101200-48-0	ND	0.01

除非另有书面协议，本报告由本公司依据《 服务通用条款 》出具。
《 服务通用条款 》印刷在正本报告纸背面，或通过 https://www.sgs.com/en/Terms-and-Conditions 查询。请特别关注其中涉及责任限定，赔偿以及司法管辖的相关条款。任何报告的持有方需知悉，此报告内容仅反映SGS受限于客户指示下，且在当时所得结论。SGS仅对其客户负责，并且此报告不能免除交易各方根据交易文件所享有的权利和应履行的义务。未获得本公司书面批准，本文件不得进行复制，全文除外。任何未经授权对报告内容及形式进行的修改、伪造或扭曲都是违法行为，违法者将会被追究法律责任。除非另有声明，本测试报告所示结果仅涉及受测试样品。
注意：检测/检验报告或证书的真实性，请通过电话 (86 – 755) 83071443 或邮箱 CN.Doccheck@sgs.com 查询。
No.1, 5/F, Building D1, No. 1666, Section 2 of Chenglong Road, Economic and Technological Development Zone, Longquanyi District, Chengdu, Sichuan, China 610100 t (86–28) 8481 0338 www.sgsgroup.com.cn
中国・四川・成都市龙泉驿区经济技术开发区成龙大道二段1666号 D1栋05层1号 邮编：610100 t (86–28) 8481 0338 sgs.china@sgs.com
Member of the SGS Group (SGS SA)

编号	检测项目	单位	CAS No.	检测结果 1	定量限
305	#敌百虫	mg/kg	52-68-6	ND	0.01
306	#氯啶菌酯	mg/kg	902760-40-1	ND	0.01
307	三环唑	mg/kg	41814-78-2	ND	0.01
308	#十三吗啉	mg/kg	24602-86-6	ND	0.01
309	肟菌酯	mg/kg	141517-21-7	ND	0.01
310	氟菌唑	mg/kg	68694-11-1	ND	0.01
311	杀铃脲	mg/kg	64628-44-0	ND	0.01
312	#氟胺磺隆	mg/kg	126535-15-7	ND	0.01
313	#抗倒酯	mg/kg	95266-40-3	ND	0.01
314	#灭菌唑	mg/kg	131983-72-7	ND	0.01
315	#烯效唑和高效烯效唑	mg/kg	83657-17-4 /83657-22-1	ND	0.01
316	#蚜灭磷（完灭硫磷）	mg/kg	2275-23-2	ND	0.01
317	#灭除威	mg/kg	2655-14-3	ND	0.01
318	苯酰菌胺（苯酰草胺）	mg/kg	156052-68-5	ND	0.01
319	#三唑酮和三唑醇之和	mg/kg	-	ND	-
320	#灭多威（灭多威和硫双威之和）	mg/kg	-	ND	-
321	克百威(克百威和 3-羟基克百威之和，以克百威表示)	mg/kg	-	ND	-
322	#氟虫腈(氟虫腈、氟虫腈砜及氟虫腈亚砜之和，以氟虫腈表示)	mg/kg	-	ND	-
323	#涕灭威(涕灭威、涕灭威亚砜及涕灭砜威之和，以涕灭威表示)	mg/kg	-	ND	-
324	#倍硫磷(倍硫磷、倍硫磷亚砜及倍硫磷砜之和，以倍硫磷表示)	mg/kg	-	ND	-
325	#乙硫甲威（乙硫甲威,乙硫甲威-砜,乙硫甲威-亚砜之和，以乙硫甲威表示）	mg/kg	-	ND	-
326	#环丙氨嗪(灭蝇胺)	mg/kg	66215-27-8	ND	0.01
327	#二硫酮砜	mg/kg	2497-06-5	ND	0.01
328	#二硫代亚砜	mg/kg	2497-07-6	ND	0.01
329	#氟甲腈	mg/kg	205650-65-3	ND	0.01
330	#噁唑菌酮	mg/kg	131807-57-3	ND	0.01
331	#咪鲜胺-脱氨基咪唑	mg/kg	139520-94-8	ND	0.05
332	#咪鲜胺-脱咪唑甲酰胺基	mg/kg	139542-32-8	ND	0.05
333	#丁氟螨酯	mg/kg	400882-07-7	ND	0.01
334	#苯嘧磺草胺	mg/kg	372137-35-4	ND	0.01
335	#氟唑环菌胺	mg/kg	874967-67-6	ND	0.01
336	#胺苯吡菌酮	mg/kg	473798-59-3	ND	0.01
337	#消螨酚	mg/kg	131-89-5	ND	0.01
338	#戊硝酚	mg/kg	4097-36-3	ND	0.01
339	#烯啶虫胺	mg/kg	120738-89-8	ND	0.01

通标标准技术服务有限公司四川分公司 检验检测专用章 Inspection & Testing Services 农产食品实验室

除非另有书面协议，本报告由本公司依据《 服务通用条款 》出具。
《 服务通用条款 》印刷在正本报告纸背面，或通过 https://www.sgs.com/en/Terms-and-Conditions 查询。请特别关注其中涉及责任限定，赔偿以及司法管辖的相关条款。任何报告的持有方需知悉，此报告内容仅反映SGS受限于客户指示下，且在当时所得结论。SGS仅对其客户负责，并且此报告不能免除交易各方根据交易文件所享有的权利和应履行的义务。未获得本公司书面批准，本文件不得进行复制，全文除外。任何未经授权对报告内容及形式进行的修改、伪造或扭曲都是违法行为，违法者将会被追究法律责任。除非另有声明，本测试报告所示结果仅涉及受测试样品。
注意：检测/检验报告或证书的真实性，请通过电话 (86－755) 83071443 或邮箱 CN.Doccheck@sgs.com 查询。

No.1, 5F, Building D1, No. 1666, Section 2 of Chenglong Road, Economic and Technological Development Zone, Longquanyi District, Chengdu, Sichuan, China 610100 t (86–28) 8481 0338 www.sgsgroup.com.cn
中国・四川・成都市龙泉驿区经济技术开发区成龙大道二段1666号 D1栋05层1号 邮编：610100 t (86–28) 8481 0338 sgs.china@sgs.com

Member of the SGS Group (SGS SA)

编号	检测项目	单位	CAS No.	检测结果 1	定量限
			/150824-47-8		
340	#联苯肼酯	mg/kg	149877-41-8	ND	0.01
341	#吡噻菌胺	mg/kg	183675-82-3	ND	0.01
342	2,4-滴 & 2,4-滴钠盐	mg/kg	2702-72-9 /94-75-7	ND	0.01
343	#伊维菌素	mg/kg	70288-86-7	ND	0.05
344	#除虫菊素 I	mg/kg	121-21-1	ND	0.01
345	#除虫菊素 II	mg/kg	121-29-9	ND	0.01
346	#氟咯草酮	mg/kg	61213-25-0	ND	0.01
347	#扑草净	mg/kg	7287-19-6	ND	0.01
348	多杀霉素	mg/kg	168316-95-8	ND	0.01
349	#胺苯磺隆	mg/kg	97780-06-8	ND	0.01
350	#唑嘧菌胺	mg/kg	865318-97-4	ND	0.01
351	#噻草酮	mg/kg	101205-02-1	ND	0.01
352	#唑胺菌酯	mg/kg	915410-70-7	ND	0.01
353	#丁吡吗啉	mg/kg	868390-90-3	ND	0.01
354	#啶菌噁唑	mg/kg	847749-37-5	ND	0.01
355	#乙基多杀菌素 J	mg/kg	187166-40-1	ND	0.01
356	#咪鲜胺(咪鲜胺、咪鲜胺-脱氨基咪唑及咪鲜胺-脱咪唑甲酰胺基之和，以咪鲜胺表示)	mg/kg	-	ND	-
357	#胺鲜酯	mg/kg	10369-83-2	ND	0.05
358	#苯菌灵和多菌灵	mg/kg	-	ND	0.01
359	#苯并烯氟菌唑	mg/kg	1072957-71-1	ND	0.05
360	#烯草酮亚砜	mg/kg	111031-14-2	ND	0.01
361	#烯草酮砜	mg/kg	111031-17-5	ND	0.01
362	#螺虫乙酯-酮基-羟基	mg/kg	1172134-11-0	ND	0.01
363	#螺虫乙酯-烯醇-葡萄糖苷	mg/kg	1172614-86-6	ND	0.01
364	#螺虫乙酯-单-羟基	mg/kg	1172134-12-1	ND	0.01
365	#甲磺草胺	mg/kg	122836-35-5	ND	0.05
366	#丰索磷砜	mg/kg	14255-72-2	ND	0.01
367	#螺虫乙酯-烯醇	mg/kg	203312-38-3	ND	0.01
368	#甲硫威砜	mg/kg	2179-25-1	ND	0.01
369	#甲硫威亚砜	mg/kg	2635-10-1	ND	0.01
370	#烯肟菌酯	mg/kg	238410-11-2	ND	0.01
371	#N-二甲基甲酰基甲硫基甲醛肟	mg/kg	30558-43-1	ND	0.01
372	#烯肟菌胺	mg/kg	366815-39-6	ND	0.01
373	#阿苯达唑	mg/kg	54965-21-8	ND	0.01

除非另有书面协议，本报告由本公司依据《服务通用条款》出具。
《服务通用条款》印刷在正本报告纸背面，或通过 https://www.sgs.com/en/Terms-and-Conditions 查询。请特别关注其中涉及责任限定，赔偿以及司法管辖的相关条款。任何报告的持有方需知悉，此报告内容仅反映SGS受限于客户指示下，且在当时所得结论。SGS仅对其客户负责，并且此报告不能免除交易各方根据交易文件所享有的权利和应履行的义务。未获得本公司书面批准，本文件不得进行复制，全文除外。任何未经授权对报告内容及形式进行的修改、伪造或扭曲都是违法行为，违法者将会被追究法律责任。除非另有声明，本测试报告所示结果仅涉及受测试样品。
注意：检测/检验报告或证书的真实性，请通过电话 (86-755) 83071443 或邮箱 CN.Doccheck@sgs.com 查询。
No.1, 5F, Building D1, No. 1666, Section 2 of Chenglong Road, Economic and Technological Development Zone, Longquanyi District, Chengdu, Sichuan, China 610100 t (86-28) 8481 0338 www.sgsgroup.com.cn
中国·四川·成都市龙泉驿区经济技术开发区成龙大道二段1666号 D1栋05层1号 邮编：610100 t (86-28) 8481 0338 sgs.china@sgs.com
Member of the SGS Group (SGS SA)

编号	检测项目	单位	CAS No.	检测结果 1	定量限
374	#丙嗪嘧磺隆	mg/kg	570415-88-2	ND	0.01
375	#丰索磷-氧化砜	mg/kg	6132-17-8	ND	0.01
376	#氟节胺	mg/kg	62924-70-3	ND	0.05
377	#丰索磷-氧	mg/kg	6552-21-2	ND	0.01
378	#丁香菌酯	mg/kg	850881-70-8	ND	0.01
379	#嗪吡嘧磺隆	mg/kg	868680-84-6	ND	0.05
380	#吡唑萘菌胺	mg/kg	881685-58-1	ND	0.01
381	#螺虫乙酯(螺虫乙酯、螺虫乙酯-烯醇、螺虫乙酯-烯醇-葡萄糖苷、螺虫乙酯-酮基-羟基及螺虫乙酯-单-羟基之和，以螺虫乙酯表示)	mg/kg	-	ND	0.01
382	#甲硫威(甲硫威、甲硫威亚砜及甲硫威砜之和，以甲硫威表示)	mg/kg	-	ND	-
383	#亚砜磷(亚砜磷、甲基内吸磷及砜吸磷之和，以亚砜磷表示)	mg/kg	-	ND	-
384	#烯草酮(烯草酮、烯草酮亚砜及烯草酮砜之和，以烯草酮表示)	mg/kg	-	ND	-
385	#丰索磷(丰索磷、氧丰索磷、氧丰索磷砜及丰索磷砜，以丰索磷表示)	mg/kg	-	ND	-
386	#除虫菊素(除虫菊素 I 和除虫菊素 II 之和)	mg/kg	-	ND	-
387	#乙草胺	mg/kg	34256-82-1	ND	0.01
388	#氟丙菊酯	mg/kg	101007-06-1	ND	0.1
389	甲草胺	mg/kg	15972-60-8	ND	0.01
390	艾氏剂	mg/kg	309-00-2	ND	0.01
391	#丙烯菊酯	mg/kg	584-79-2	ND	0.01
392	#二丙烯草胺	mg/kg	93-71-0	ND	0.01
393	莠灭净	mg/kg	834-12-8	ND	0.02
394	#蒽醌	mg/kg	84-65-1	ND	0.01
395	益棉磷（乙基保棉磷）（乙基谷硫磷）	mg/kg	2642-71-9	ND	0.02
396	#苯草醚	mg/kg	74070-46-5	ND	0.01
397	α-六六六	mg/kg	319-84-6	ND	0.01
398	#燕麦灵	mg/kg	101-27-9	ND	0.05
399	噁虫威	mg/kg	22781-23-3	ND	0.01
400	乙丁氟灵	mg/kg	1861-40-1	ND	0.01
401	#呋草黄（呋草磺）	mg/kg	68505-69-1	ND	0.01
402	β-六六六	mg/kg	319-85-7	ND	0.01
403	#甲羧除草醚	mg/kg	42576-02-3	ND	0.01
404	联苯菊酯	mg/kg	82657-04-3	ND	0.01
405	乙基溴硫磷	mg/kg	4824-78-6	ND	0.01
406	溴硫磷	mg/kg	2104-96-3	ND	0.01

通标标准技术服务有限公司四川分公司 检验检测专用章 Inspection & Testing Services 农产食品实验室

除非另有书面协议，本报告由本公司依据《 服务通用条款 》出具。
《 服务通用条款 》印刷在正本报告纸背面，或通过 https://www.sgs.com/en/Terms-and-Conditions 查询。请特别关注其中涉及责任限定，赔偿以及司法管辖的相关条款。任何报告的持有方需知悉，此报告内容仅反映SGS受限于客户指示下，且在当时所得结论。SGS仅对其客户负责，并且此报告不能免除交易各方根据交易文件所享有的权利和应履行的义务。未获得本公司书面批准，本文件不得进行复制，全文除外。任何未经授权对报告内容及形式进行的修改、伪造或扭曲都是违法行为，违法者将会被追究法律责任。除非另有声明，本测试报告所示结果仅涉及受测试样品。
注意：检测/检验报告或证书的真实性，请通过电话 (86－755) 83071443 或邮箱 CN.Doccheck@sgs.com 查询。
No.1, 5F, Building D1, No. 1666, Section 2 of Chenglong Road, Economic and Technological Development Zone, Longquanyi District, Chengdu, Sichuan, China 610100 t (86–28) 8481 0338 www.sgsgroup.com.cn
中国・四川・成都市龙泉驿区经济技术开发区成龙大道二段1666号 D1栋05层1号 邮编：610100 t (86–28) 8481 0338 sgs.china@sgs.com
Member of the SGS Group (SGS SA)

编号	检测项目	单位	CAS No.	检测结果 1	定量限
407	溴螨酯	mg/kg	18181-80-1	ND	0.01
408	#丁草胺	mg/kg	23184-66-9	ND	0.01
409	#抑草磷	mg/kg	36335-67-8	ND	0.01
410	仲丁灵	mg/kg	33629-47-9	ND	0.01
411	#丁草敌（丁草特）	mg/kg	2008-41-5	ND	0.01
412	δ-六六六	mg/kg	319-86-8	ND	0.01
413	γ-六六六（林丹）	mg/kg	58-89-9	ND	0.01
414	硫线磷	mg/kg	95465-99-9	ND	0.02
415	三硫磷	mg/kg	786-19-6	ND	0.01
416	杀螨醚（氯杀螨）	mg/kg	103-17-3	ND	0.01
417	虫螨腈（溴虫腈）	mg/kg	122453-73-0	ND	0.01
418	杀螨酯	mg/kg	80-33-1	ND	0.01
419	毒虫畏（杀螟威）	mg/kg	470-90-6	ND	0.01
420	乙酯杀螨醇（克氯苯）	mg/kg	510-15-6	ND	0.01
421	氯苯甲醚	mg/kg	2675-77-6	ND	0.02
422	#百菌清	mg/kg	1897-45-6	ND	0.01
423	氯苯胺灵	mg/kg	101-21-3	ND	0.01
424	毒死蜱	mg/kg	2921-88-2	ND	0.01
425	甲基毒死蜱	mg/kg	5598-13-0	ND	0.01
426	氯酞酸甲酯	mg/kg	1861-32-1	ND	0.01
427	乙菌利（克氯得）	mg/kg	84332-86-5	ND	0.01
428	顺式-氯丹（α-氯丹）	mg/kg	5103-71-9	ND	0.01
429	炔草酯（炔草酸）	mg/kg	105512-06-9	ND	0.01
430	杀螟腈（杀虫腈）	mg/kg	2636-26-2	ND	0.01
431	异噁草松（异噁草酮）	mg/kg	81777-89-1	ND	0.01
432	#解草酯（解毒喹）	mg/kg	99607-70-2	ND	0.01
433	#蝇毒磷	mg/kg	56-72-4	ND	0.01
434	#氟氯氰菊酯和高效氟氯氰菊酯	mg/kg	1820573-27-0 /68359-37-5	ND	0.01
435	#氰氟草酯	mg/kg	122008-85-9	ND	0.01
436	#氯氟氰菊酯和高效氯氟氰菊酯	mg/kg	68085-85-8 /91465-08-6	ND	0.01
437	氯氰菊酯	mg/kg	52315-07-8	ND	0.01
438	环丙唑醇	mg/kg	94361-06-5	ND	0.01
439	#草枯醚	mg/kg	1836-77-7	ND	0.01
440	#丙酯杀螨醇	mg/kg	5836-10-2	ND	0.01
441	#巴毒磷	mg/kg	7700-17-6	ND	0.01
442	#2,4-滴丁酯	mg/kg	94-80-4	ND	0.01
443	溴氰菊酯	mg/kg	52918-63-5	ND	0.01
444	二嗪磷（二嗪农）	mg/kg	333-41-5	ND	0.01

除非另有书面协议，本报告由本公司依据《服务通用条款》出具。
《服务通用条款》印刷在正本报告纸背面，或通过 https://www.sgs.com/en/Terms-and-Conditions 查询。请特别关注其中涉及责任限定，赔偿以及司法管辖的相关条款。任何报告的持有方需知悉，此报告内容仅反映SGS受限于客户指示下，且在当时所得结论。SGS仅对其客户负责，并且此报告不能免除交易各方根据交易文件所享有的权利和应履行的义务。未获得本公司书面批准，本文件不得进行复制，全文除外。任何未经授权对报告内容及形式进行的修改、伪造或扭曲都是违法行为，违法者将会被追究法律责任。除非另有声明，本测试报告所示结果仅涉及受测试样品。
注意：检测/检验报告或证书的真实性，请通过电话 (86－755) 83071443 或邮箱 CN.Doccheck@sgs.com 查询。

通标标准技术服务有限公司四川分公司 检验检测专用章 Inspection & Testing Services

No.1, 5/F, Building D1, No. 1666, Section 2 of Chenglong Road, Economic and Technological Development Zone, Longquanyi District, Chengdu, Sichuan, China 610100 t (86–28) 8481 0338 www.sgsgroup.com.cn
中国・四川・成都市龙泉驿区经济技术开发区成龙大道二段1666号 D1栋05层1号 邮编：610100 t (86–28) 8481 0338 sgs.china@sgs.com

Member of the SGS Group (SGS SA)

编号	检测项目	单位	CAS No.	检测结果 1	定量限
445	#敌草腈	mg/kg	1194-65-6	ND	0.01
446	除线磷	mg/kg	97-17-6	ND	0.01
447	#苯氟磺胺（抑菌灵）	mg/kg	1085-98-9	ND	0.01
448	#烯丙酰草胺（二氯丙烯胺）	mg/kg	37764-25-3	ND	0.01
449	敌敌畏	mg/kg	62-73-7	ND	0.01
450	#苄氯三唑醇	mg/kg	75736-33-3	ND	0.01
451	禾草灵	mg/kg	51338-27-3	ND	0.01
452	氯硝胺	mg/kg	99-30-9	ND	0.01
453	p,p'-三氯杀螨醇	mg/kg	115-32-2	ND	0.01
454	狄氏剂	mg/kg	60-57-1	ND	0.01
455	#二甲吩草胺	mg/kg	87674-68-8	ND	0.01
456	#噻节因	mg/kg	55290-64-7	ND	0.01
457	#甲基毒虫畏	mg/kg	2274-67-1	ND	0.01
458	#敌噁磷（敌杀磷）	mg/kg	78-34-2	ND	0.01
459	#双苯酰草胺	mg/kg	957-51-7	ND	0.01
460	二苯胺	mg/kg	122-39-4	ND	0.01
461	#乙拌磷	mg/kg	298-04-4	ND	0.01
462	#氟硫草定	mg/kg	97886-45-8	ND	0.01
463	#甲基异内吸磷	mg/kg	919-86-8	ND	0.01
464	#灭菌磷	mg/kg	5131-24-8	ND	0.01
465	α-硫丹	mg/kg	959-98-8	ND	0.01
466	β-硫丹	mg/kg	33213-65-9	ND	0.01
467	#敌瘟磷（敌瘟散）	mg/kg	17109-49-8	ND	0.01
468	硫丹硫酸酯	mg/kg	1031-07-8	ND	0.02
469	异狄氏剂	mg/kg	72-20-8	ND	0.01
470	苯硫磷	mg/kg	2104-64-5	ND	0.01
471	#乙丁烯氟灵	mg/kg	55283-68-6	ND	0.01
472	乙硫磷	mg/kg	563-12-2	ND	0.01
473	乙氧呋草黄	mg/kg	26225-79-6	ND	0.01
474	灭线磷	mg/kg	13194-48-4	ND	0.01
475	#吲熟酯	mg/kg	27512-72-7	ND	0.01
476	醚菊酯	mg/kg	80844-07-1	ND	0.01
477	#土菌灵	mg/kg	2593-15-9	ND	0.01
478	乙嘧硫磷	mg/kg	38260-54-7	ND	0.01
479	苯线磷	mg/kg	22224-92-6	ND	0.05
480	#氯苯嘧啶醇	mg/kg	60168-88-9	ND	0.01
481	皮蝇磷	mg/kg	299-84-3	ND	0.01
482	杀螟硫磷（杀螟松）	mg/kg	122-14-5	ND	0.01
483	#苯硫威	mg/kg	62850-32-2	ND	0.01
484	甲氰菊酯	mg/kg	39515-41-8	ND	0.01

检验检测专用章 Inspection & Testing Services
通标标准技术服务有限公司四川分公司 农产食品实验室

除非另有书面协议，本报告由本公司依据《服务通用条款》出具。
《服务通用条款》印刷在正本报告纸背面，或通过 https://www.sgs.com/en/Terms-and-Conditions 查询。请特别关注其中涉及责任限定，赔偿以及司法管辖的相关条款。任何报告的持有方需知悉，此报告内容仅反映SGS受限于客户指示下，且在当时所得结论。SGS仅对其客户负责，并且此报告不能免除交易各方根据交易文件所享有的权利和应履行的义务。未获得本公司书面批准，本文件不得进行复制，全文除外。任何未经授权对报告内容及形式进行的修改、伪造或扭曲都是违法行为，违法者将会被追究法律责任。除非另有声明，本测试报告所示结果仅涉及受测试样品。
注意：检测/检验报告或证书的真实性，请通过电话(86－755) 83071443或邮箱 CN.Doccheck@sgs.com 查询。

编号	检测项目	单位	CAS No.	检测结果 1	定量限
			/64257-84-7		
485	丰索磷	mg/kg	115-90-2	ND	0.01
486	氰戊菊酯和 S-氰戊菊酯	mg/kg	51630-58-1 /66230-04-4	ND	0.01
487	嘧螨酯	mg/kg	229977-93-9	ND	0.01
488	氟氰戊菊酯	mg/kg	70124-77-5	ND	0.01
489	#胺氟草酯（氟烯草酸）	mg/kg	87546-18-7	ND	0.01
490	#氟喹唑	mg/kg	136426-54-5	ND	0.01
491	#氟啶草酮	mg/kg	59756-60-4	ND	0.01
492	氟哇唑（氟硅唑）	mg/kg	85509-19-9	ND	0.01
493	#四氯苯酞	mg/kg	27355-22-2	ND	0.01
494	#氟酰胺	mg/kg	66332-96-5	ND	0.01
495	地虫硫磷	mg/kg	944-22-9	ND	0.01
496	#安硫磷（安果磷）	mg/kg	2540-82-1	ND	0.01
497	#噻唑硫磷（噻唑磷）	mg/kg	98886-44-3	ND	0.01
498	#乙羧氟草醚	mg/kg	77501-90-7	ND	0.01
499	呋霜灵	mg/kg	57646-30-7	ND	0.01
500	#三氟硝草醚	mg/kg	15457-05-3	ND	0.01
501	#氟除草醚	mg/kg	13738-63-1	ND	0.01
502	#格螨酯	mg/kg	97-16-5	ND	0.01
503	#苄螨醚（苄满醚）	mg/kg	111872-58-3	ND	0.01
504	七氯	mg/kg	76-44-8	ND	0.01
505	环氧七氯（B 型，顺式）	mg/kg	1024-57-3 /28044-83-9	ND	0.01
506	庚烯磷	mg/kg	23560-59-0	ND	0.01
507	六氯苯	mg/kg	118-74-1	ND	0.01
508	#环嗪酮	mg/kg	51235-04-2	ND	0.01
509	#氯唑磷	mg/kg	42509-80-8	ND	0.01
510	#水胺硫磷	mg/kg	24353-61-5	ND	0.01
511	异艾氏剂	mg/kg	465-73-6	ND	0.01
512	异柳磷	mg/kg	25311-71-1	ND	0.01
513	甲基异柳磷	mg/kg	99675-03-3	ND	0.01
514	#恶唑磷	mg/kg	18854-01-8	ND	0.01
515	#灭蚜磷	mg/kg	2595-54-2	ND	0.01
516	#灭锈胺	mg/kg	55814-41-0	ND	0.01
517	虫螨畏	mg/kg	62610-77-9	ND	0.01
518	#烯虫酯	mg/kg	40596-69-8	ND	0.01
519	甲氧氯（甲氧滴滴涕）	mg/kg	72-43-5	ND	0.01
520	#五氯苯甲硫醚（甲基五氯苯硫醚）	mg/kg	1825-19-0	ND	0.01
521	嗪草酮	mg/kg	21087-64-9	ND	0.01
522	速灭磷	mg/kg	7786-34-7	ND	0.01

通标标准技术服务有限公司四川分公司 检验检测专用章 Inspection & Testing Services

除非另有书面协议，本报告由本公司依据《 服务通用条款 》出具。
《 服务通用条款 》印刷在正本报告纸背面，或通过 https://www.sgs.com/en/Terms-and-Conditions 查询。请特别关注其中涉及责任限定，赔偿以及司法管辖的相关条款。任何报告的持有方需知悉，此报告内容仅反映SGS受限于客户指示下，且在当时所得结论。SGS仅对其客户负责，并且此报告不能免除交易各方根据交易文件所享有的权利和应履行的义务。未获得本公司书面批准，本文件不得进行复制，全文除外。任何未经授权对报告内容及形式进行的修改、伪造或扭曲都是违法行为，违法者将会被追究法律责任。除非另有声明，本测试报告所示结果仅涉及受测试样品。
注意：检测/检验报告或证书的真实性，请通过电话 (86 –755) 83071443 或邮箱 CN.Doccheck@sgs.com 查询。

No.1, 5/F., Building D1, No. 1666, Section 2 of Chenglong Road, Economic and Technological Development Zone, Longquanyi District, Chengdu, Sichuan, China 610100 t (86–28) 8481 0338 www.sgsgroup.com.cn
中国・四川・成都市龙泉驿区经济技术开发区成龙大道二段1666号 D1栋05层1号 邮编：610100 t (86–28) 8481 0338 sgs.china@sgs.com

Member of the SGS Group (SGS SA)

编号	检测项目	单位	CAS No.	检测结果 1	定量限
523	灭蚁灵	mg/kg	2385-85-5	ND	0.01
524	#绿谷隆	mg/kg	1746-81-2	ND	0.01
525	敌草胺	mg/kg	15299-99-7	ND	0.01
526	除草醚	mg/kg	1836-75-5	ND	0.01
527	酞菌酯	mg/kg	10552-74-6	ND	0.01
528	o,p'-滴滴涕	mg/kg	789-02-6	ND	0.01
529	邻苯基苯酚（2-苯基苯酚）（OPP）	mg/kg	90-43-7	ND	0.01
530	八氯二丙醚	mg/kg	127-90-2	ND	0.01
531	p,p'-滴滴滴	mg/kg	72-54-8	ND	0.01
532	p,p'-滴滴伊	mg/kg	72-55-9	ND	0.01
533	p,p'-滴滴涕	mg/kg	50-29-3	ND	0.01
534	多效唑	mg/kg	76738-62-0	ND	0.01
535	乙基对硫磷（对硫磷）	mg/kg	56-38-2	ND	0.01
536	甲基对硫磷	mg/kg	298-00-0	ND	0.01
537	戊菌唑	mg/kg	66246-88-6	ND	0.01
538	二甲戊灵（二甲戊乐灵）	mg/kg	40487-42-1	ND	0.01
539	五氯苯胺	mg/kg	527-20-8	ND	0.01
540	五氯甲氧基苯（五氯茴香醚）	mg/kg	1825-21-4	ND	0.01
541	#戊基恶唑酮	mg/kg	110956-75-7	ND	0.01
542	氯菊酯	mg/kg	52645-53-1	ND	0.01
543	#苯醚菊酯	mg/kg	26002-80-2	ND	0.01
544	稻丰散	mg/kg	2597-03-7	ND	0.01
545	甲拌磷	mg/kg	298-02-2	ND	0.01
546	#甲拌磷砜	mg/kg	2588-04-7	ND	0.01
547	#甲拌磷亚砜	mg/kg	2588-03-6	ND	0.01
548	磷胺	mg/kg	13171-21-6	ND	0.02
549	增效醚	mg/kg	51-03-6	ND	0.01
550	#哌草磷	mg/kg	24151-93-7	ND	0.01
551	嘧啶磷（乙基嘧啶磷）	mg/kg	23505-41-1	ND	0.01
552	甲基虫螨磷（甲基嘧啶磷）	mg/kg	29232-93-7	ND	0.01
553	#丙草胺	mg/kg	51218-49-6	ND	0.01
554	腐霉利	mg/kg	32809-16-8	ND	0.01
555	丙溴磷（溴氯磷）	mg/kg	41198-08-7	ND	0.01
556	#敌稗	mg/kg	709-98-8	ND	0.01
557	#丙虫磷	mg/kg	7292-16-2	ND	0.01
558	#扑灭津	mg/kg	139-40-2	ND	0.01
559	苯胺灵	mg/kg	122-42-9	ND	0.01
560	丙环唑	mg/kg	60207-90-1	ND	0.01
561	炔苯酰草胺（拿草特）	mg/kg	23950-58-5	ND	0.01
562	丙硫磷（普硫松）	mg/kg	34643-46-4	ND	0.01

通标标准技术服务有限公司四川分公司
检验检测专用章
Inspection & Testing Services

除非另有书面协议，本报告由本公司依据《 服务通用条款 》出具。
《 服务通用条款 》印刷在正本报告纸背面，或通过 https://www.sgs.com/en/Terms-and-Conditions 查询。请特别关注其中涉及责任限定，赔偿以及司法管辖的相关条款。任何报告的持有方需知悉，此报告内容仅反映SGS受限于客户指示下，且在当时所得结论。SGS仅对其客户负责，并且此报告不能免除交易各方根据交易文件所享有的权利和应履行的义务。未获得本公司书面批准，本文件不得进行复制，全文除外。任何未经授权对报告内容及形式进行的修改、伪造或扭曲都是违法行为，违法者将会被追究法律责任。除非另有声明，本测试报告所示结果仅涉及受测试样品。
注意：检测/检验报告或证书的真实性，请通过电话（86－755）83071443或邮箱 CN.Doccheck@sgs.com 查询。

No.1, 5/F., Building D1, No. 1666, Section 2 of Chenglong Road, Economic and Technological Development Zone, Longquanyi District, Chengdu, Sichuan, China 610100 t (86–28) 8481 0338 www.sgsgroup.com.cn
中国・四川・成都市龙泉驿区经济技术开发区成龙大道二段1666号 D1栋05层1号 邮编：610100 t (86–28) 8481 0338 sgs.china@sgs.com

Member of the SGS Group (SGS SA)

编号	检测项目	单位	CAS No.	检测结果 1	定量限
563	#吡唑硫磷	mg/kg	77458-01-6	ND	0.01
564	#吡草醚（霸草灵）	mg/kg	129630-19-9	ND	0.01
565	#嘧草醚	mg/kg	136191-64-5	ND	0.01
566	喹硫磷	mg/kg	13593-03-8	ND	0.01
567	五氯硝基苯	mg/kg	82-68-8	ND	0.01
568	胺丙畏（巴胺磷）（烯虫磷）	mg/kg	31218-83-4	ND	0.01
569	#蔬果磷	mg/kg	3811-49-2	ND	0.01
570	#硅醚菊酯	mg/kg	105024-66-6	ND	0.01
571	治螟磷	mg/kg	3689-24-5	ND	0.01
572	硫丙磷	mg/kg	35400-43-2	ND	0.01
573	#苯腈磷（苯腈硫磷）	mg/kg	13067-93-1	ND	0.01
574	#螺甲螨酯	mg/kg	283594-90-1	ND	0.01
575	氟胺氰菊酯	mg/kg	102851-06-9	ND	0.01
576	#丁基嘧啶磷	mg/kg	96182-53-5	ND	0.01
577	四氯硝基苯	mg/kg	117-18-0	ND	0.01
578	七氟菊酯	mg/kg	79538-32-2	ND	0.01
579	#特草定	mg/kg	5902-51-2	ND	0.01
580	特丁硫磷	mg/kg	13071-79-9	ND	0.01
581	#特丁净（特丁草净）	mg/kg	886-50-0	ND	0.01
582	#特丁津	mg/kg	5915-41-3	ND	0.01
583	杀虫畏	mg/kg	22248-79-9	ND	0.02
584	四氟醚唑（氟醚唑）	mg/kg	112281-77-3	ND	0.01
585	三氯杀螨砜	mg/kg	116-29-0	ND	0.01
586	胺菊酯	mg/kg	7696-12-0	ND	0.01
587	#甲基乙拌磷（二甲基硫吸磷）	mg/kg	640-15-3	ND	0.01
588	甲基立枯磷	mg/kg	57018-04-9	ND	0.01
589	甲苯氟磺胺	mg/kg	731-27-1	ND	0.02
590	反式氯丹	mg/kg	5103-74-2	ND	0.01
591	野麦畏	mg/kg	2303-17-5	ND	0.01
592	1,2,4-三丁基三硫磷酸酯（脱叶磷）	mg/kg	78-48-8	ND	0.01
593	氟乐灵	mg/kg	1582-09-8	ND	0.01
594	乙烯菌核利	mg/kg	50471-44-8	ND	0.01
595	（顺式-氯丹,反式-氯丹）总量	mg/kg	-	ND	-
596	七氯和环氧七氯总量	mg/kg	-	ND	-
597	滴滴涕总量（o,p'-滴滴涕,p,p'-滴滴滴,p,p'-滴滴伊,p,p'-滴滴涕）	mg/kg	-	ND	-
598	#（甲拌磷,甲拌磷砜,甲拌磷亚砜）总量	mg/kg	-	ND	-
599	硫丹总量 （α-硫丹,β-硫丹和硫丹硫酸酯之和）	mg/kg	1031-07-8 /33213-65-9 /959-98-8	ND	-

除非另有书面协议，本报告由本公司依据《 服务通用条款 》出具。
《 服务通用条款 》印刷在正本报告纸背面，或通过 https://www.sgs.com/en/Terms-and-Conditions 查询。请特别关注其中涉及责任限定，赔偿以及司法管辖的相关条款。任何报告的持有方需知悉，此报告内容仅反映SGS受限于客户指示下，且在当时所得结论。SGS仅对其客户负责，并且此报告不能免除交易各方根据交易文件所享有的权利和应履行的义务。未获得本公司书面批准，本文件不得进行复制，全文除外。任何未经授权对报告内容及形式进行的修改、伪造或扭曲都是违法行为，违法者将会被追究法律责任。除非另有声明，本测试报告所示结果仅涉及受测试样品。
注意：检测/检验报告或证书的真实性，请通过电话(86－755) 83071443或邮箱 CN.Doccheck@sgs.com查询。

No.1,5F, Building D1, No. 1666, Section 2 of Chengdong Road, Economic and Technological Development Zone, Longquanyi District, Chengdu, Sichuan, China 610100 t (86–28) 8481 0338 www.sgsgroup.com.cn
中国・四川・成都市龙泉驿区经济技术开发区成龙大道二段1666号 D1栋05层1号 邮编：610100 t (86–28) 8481 0338 sgs.china@sgs.com

Member of the SGS Group (SGS SA)

检测报告

CDF25-0001880-01　　　　发布日期：2025-05-06

编号	检测项目	单位	CAS No.	检测结果<br>1	定量限
600	六六六总量（α-六六六,β-六六六,γ-六六六,δ-六六六）	mg/kg	319-84-6<br>/319-85-7<br>/319-86-8<br>/58-89-9	ND	-
601	#氯氰菊酯&Zeta-氯氰菊酯	mg/kg	-	ND	0.01
602	#野燕枯（硫酸二甲酯）	mg/kg	43222-48-6	ND	0.01

备注:
1.ND = 未检出
2.#检测项目不在本实验室的 CNAS 认可范围内。

注意事项：
除非另有说明，本检测结果仅与被检测物品有关。未经检验机构书面同意，委托人不得擅自使用检测结果进行不当宣传。本检测报告仅作为科研、教学或内部质量控制之用。未经检验机构批准，不得复制（全文复制除外）报告。

结束

除非另有书面协议，本报告由本公司依据《 服务通用条款 》出具。
《 服务通用条款 》印刷在正本报告纸背面，或通过 https://www.sgs.com/en/Terms-and-Conditions 查询。请特别关注其中涉及责任限定，赔偿以及司法管辖的相关条款。任何报告的持有方需知悉，此报告内容仅反映SGS受限于客户指示下，且在当时所得结论。SGS仅对其客户负责，并且此报告不能免除交易各方根据交易文件所享有的权利和应履行的义务。未获得本公司书面批准，本文件不得进行复制，全文除外。任何未经授权对报告内容及形式进行的修改、伪造或扭曲都是违法行为，违法者将会被追究法律责任。除非另有声明，本测试报告所示结果仅涉及受测试样品。
注意：检测/检验报告或证书的真实性，请通过电话 (86－755) 83071443 或邮箱 CN.Doccheck@sgs.com 查询。

No.1, 5/F., Building D1, No. 1666, Section 2 of Chenglong Road, Economic and Technological Development Zone, Longquanyi District, Chengdu, Sichuan, China 610100　t (86–28) 8481 0338　www.sgsgroup.com.cn
中国・四川・成都市龙泉驿区经济技术开发区成龙大道二段1666号 D1栋05层1号　邮编：610100　t (86–28) 8481 0338　sgs.china@sgs.com

Member of the SGS Group (SGS SA)
