# Supplementary material for: Monoglyceride-structured rubber seed oil oleogels as a partial cocoa butter replacement in chocolate: physical properties, oxidative stability and applications
Source: Food Chem X. 2026 Oct 11;39:104594. doi: 10.1016/j.fochx.2026.104594 (PMC13683454; doi:10.1016/j.fochx.2026.104594)
Supplement: Supplementary file 2 [file mmc2.pdf]

ICS 67.200.10
33

Q/XSBNHK

西双版纳华坤生物科技有限责任公司企业标准

Q/XSBNHK 0020—2015

企业标准信息公共服务平台
备案
2017年03月21日 17点49分

橡胶树种子油原油

Crude rubber seed oil

企业标准信息公共服务平台
备案
2017年03月21日 17点49分

2015－8－20 发布　　2015－11－20 实施

西双版纳华坤生物科技有限责任公司
发 布

目　　次

前言 II
1　范围 1
2　规范性引用文件 1
3　术语和定义 1
4　分类 3
5　质量要求及检验方法 3
6　检验规则 4
7　标签标识 4
8　包装、运输和储存 5

企业标准信息公共服务平台
备案
2017年03月21日 17点49分

企业标准信息公共服务平台
备案
2017年03月21日 17点49分

前　　言

本标准按照GB/T 1.1给出的规则起草。

本标准由西双版纳华坤生物科技有限责任公司提出并归口。

本标准起草单位：西双版纳华坤生物科技有限责任公司。

本标准主要起草人：朱义鑫、张娜娜、李爱萍、王云

企业标准信息公共服务平台
备案
2017年03月21日 17点49分

企业标准信息公共服务平台
备案
2017年03月21日 17点49分

橡胶树种子油原油

1 范围

本标准规定了橡胶树种子油原油的术语和定义、分类、质量要求及检验方法、检验规则、标签标识、包装、运输和储存等要求。

本标准适用于橡胶树种子油原油，包括压榨橡胶树种子油原油和浸出橡胶树种子油原油。

橡胶树种子油原油的质量指标仅适用于橡胶树种子油原油贸易。

2 规范性引用文件

下列文件对于本文件的应用是必不可少的。凡是注日期的引用文件，仅所注日期的版本适用于本文件。凡是不注日期的引用文件，其最新版本（包括所有的修改单）适用于本文件。（模版生成）

下列标准中的条款通过本标准的引用而成为本标准的条款，凡是注日期的引用文件，其随后所有的修改单（不包括勘误的内容）或修订版均不适用于本标准，然而鼓励根据本标准达成协议的各方研究是否可使用这些文件的最新版本。凡是不注日期的引用文件，其最新版本适用与本标准。(原标准)

GB/T 191 包装储运图示标志

GB 2716 食用植物油卫生标准

GB 2760 食用添加剂使用卫生标准

GB/T 5009.37 食用植物油卫生标准的分析方法

GB/T 5490 粮食、油料及植物油脂检验一般原则

GB/T 5524 食用油脂检验扦样、分样法

GB/T 5525 植物油脂检验透明度、色泽、气味、滋味鉴定法

GB/T 5526 植物油脂检验比重测定法

GB/T 5527 动植物油脂折光指数的测定

GB/T 5528 动植物油脂水分及挥发物含量测定法

GB/T 5529 植物油脂检验杂质测定法

GB/T 5530 动物油脂检验酸值和酸度测定

GB/T 5534 动植物油脂皂化值的测定

GB/T 5535 动植物油脂不皂化物测定

GB/T 5538 动植物油脂过氧化值测定

GB 7718 食品标签通用标准

GB/T 17374 食用植物油销售包装

GB/T 17375 动植物油脂 灰分测定

GB/T 17376 动植物油脂脂肪酸甲脂制备

GB/T 17377 动植物油脂脂肪酸甲脂的气象色谱分析

3 术语和定义

企业标准信息公共服务平台 备案 2017年03月21日 17点49分

企业标准信息公共服务平台 备案 2017年03月21日 17点49分

下列术语和定义适用于本标准。

3.1

橡胶树种子油 rubber seed oil

以巴西三叶橡胶树（*Hevea brasiliensis*）种子为原料制取的植物油。

3.2

橡胶树种子油原油 crude rubber seed oil

未经任何处理的不能直接供人类食用的橡胶树种子油。

3.3

成品压榨橡胶树种子油 finished pressing rubber seed oil

橡胶树种子经过压榨制取的符合标准成品质量指标和卫生要求的直接供人类食用的橡胶树种子油。

3.4

成品浸出橡胶树种子油 finished solvent extraction rubber seed oil

橡胶树种子经过浸出工艺制取的符合标准成品质量指标和卫生要求的直接供人类食用的橡胶树种子油。

3.5

折光指数 refractive index

光线从空气中射入油脂时，入射角与折射角的正弦之比值。

3.6

相对密度 specific gravity

20℃植物油的质量与同体积20℃蒸馏水的质量之比值。

3.7

碘值 iodine value

在规定条件下与100g油脂发生加成反应所需碘的克数。

3.8

皂化值 saponification value

皂化1g油脂所需的氢氧化钾毫克数。

3.9

不皂化物 unsaponifiable matter

油脂中不与碱起作用、溶于醚、不溶于水的物质，包括甾醇、脂溶性维生素和色素等。

3.10

脂肪酸 fatty acid

脂肪酸一元羧酸的总称，通式为R-COOH。

3.11

水分及挥发物 moisture and volatile matter

在一定温度条件下，油脂中所含的微量水分和挥发物

3.12

不溶性杂质 insoluble impurities

油脂中不溶于石油醚等有机溶剂的物质。

3.13

酸值 acid value

中和1g油脂中所含游离脂肪酸需要的氢氧化钾毫克数。

3.14

过氧化值 peioxide value

企业标准信息公共服务平台 备案 2017年09月21日 17点49分
企业标准信息公共服务平台 备案 2017年09月21日 17点49分

1kg 油脂中过氧化物的毫摩尔数。

3.15

溶剂残留量 residual solvent content in oil

1kg 油脂中残留的溶剂毫克数。

4 分类

橡胶树种子油分为：橡胶树种子油原油，成品压榨橡胶树种子油，成品浸出橡胶树种子油三类。

企业标准信息公共服务平台 备案 2017年03月21日 17点49分

5 质量要求及检验方法

5.1 特征指标及检验方法

表1 橡胶树种子油原油特征指标及检验方法

项 目		特征指标	检验方法
折光指数（n^{40}）：		1.467-1.471	按照 GB/T 5527 规定的方法执行
相对密度（[illegible]）：		0.919-0.926	按照 GB/T 5526 规定的方法执行
碘值(I)/(g/100g)：		130-145	按照 GB/T 5532 规定的方法执行
皂化值(KOH)/(mg/g)：		175-185	按照 GB/T 5534 规定的方法执行
不皂化物/(g/kg)：		≤20	按照 GB/T 5535 规定的方法执行
脂肪酸组成/(%)：	豆蔻酸 $C_{14:0}$	ND-0.5	按照 GB/T 17375-17377 规定的方法执行
	棕榈酸 $C_{16:0}$	8.0-11.0	
	棕榈油酸 $C_{16:1}$	ND-0.7	
	硬脂酸 $C_{18:0}$	7.0-9.5	
	油酸 $C_{18:1}$	22.0-28.0	
	亚油酸 $C_{18:2}$	33.0-41.0	
	亚麻酸 $C_{18:3}$	15.0-22.0	
	花生酸 $C_{20:0}$	ND-1.5	
	花生一烯酸 $C_{20:1}$	ND-0.7	
	其它	ND-0.8	
注 1：ND 表示未检出，定义为 0.05%。			

企业标准信息公共服务平台 备案 2017年03月21日 17点49分

5.2 质量等级指标及检验方法

表2 橡胶树种子油原油质量指标及检验方法

项 目	质量指标	检验方法
气味滋味	具有橡胶树种子油特有的气味和滋味，无异味	按照 GB/T 5525 规定的方法执行
水分及挥发物/(%) ≤	0.2	按照 GB/T 5528 规定的方法执行
不溶性杂质/(%) ≤	0.2	按照 GB/T 5529 规定的方法执行

**酸值(KOH)/(mg/g) ≤**	**30**	按照 GB/T 5530 规定的方法执行

表 2 续

项　目	质量指标	检验方法
**过氧化值/(mmol/kg) ≤**	**7.5**	按照 GB/T5538 规定的方法执行
**溶剂残留量/(mg/kg) ≤**	**100**	按照 GB/T 5009.37 规定的方法执行
注：黑体部分指标强制		

企业标准信息公共服务平台 备案 2017年03月21日 17点49分

5.3　卫生指标及及检验方法

按照GB2716、GB2760、GB/T 5009.37和国家有关规定执行。

5.4　其它

橡胶树种子油原油不得直接用于食用油，必须经过精炼符合标准后才可食用。

6　检验规则

6.1　扦样、分样

橡胶树种子油抽样方法按照 GB/T 5524 的要求执行。

6.2　产品组批

以同一班次、同一次投料、同一工艺、同一生产线生产的同一品种、同一规格的产品为一批。

6.3　出厂检验

每批产品必须经公司质量检验部门按照本标准的规定进行检查，检查合格并出具合格证后方能出厂。出厂检验按照技术要求规定，逐批检查。

企业标准信息公共服务平台 备案 2017年03月21日 17点49分

6.4　型式检查

每半年进行一次，型式检查项目为本标准规定的所有项目，发生下列情况之一时亦应进行检验。

1)　更改主要原辅料或更改关键工艺；
2)　停产半年以上，再恢复生产时；
3)　出产检验结果与上一次检验结果差异较大时；
4)　国家质量监督机构提出型式检查要求时。

6.5　判定规则

产品的卫生指标和各等级指标中有一项不合格，则判该产品不合格。其余指标如有不符合项，可以从该产品中加倍取样进行复检，以复检结果为准。

7　标签标识

7.1　预销售包装的标签除了符合 GB 7718 的规定以及要求之外，还应标注产品的加工方式，如“压榨’’、“浸出”等字样。

7.2 非零售包装应注明产品的名称、标准号、原料、生产日期、保质期、生产单位名称、地址、电话等。

7.3 应注明产品原料原产国。

8 包装、运输和储存

8.1 包装

应符合 GB/T 17374 及国家的有关规定和要求，外包装储运图示标识应符合 GB/T 191 规定要求。

8.2 运输

运输中应注意安全，防止日晒、雨淋、渗漏、污染和标签脱落。散装运输有专车，保持车辆清洁、卫生，不得与其他有毒有害的物质混装混运。装运时要轻拿、轻放、轻装、倾卸，防止重压。

8.3 储存

成品要保持清洁卫生、应贮存于阴凉、干燥及避光处。不得与有害、有毒物品或食用植物油一同存放。

企业标准信息公共服务平台
备案
2017年03月21日 17点49分

企业标准信息公共服务平台
备案
2017年03月21日 17点49分
