# Supplementary material for: Monoglyceride-structured rubber seed oil oleogels as a partial cocoa butter replacement in chocolate: physical properties, oxidative stability and applications
Source: Food Chem X. 2026 Oct 11;39:104594. doi: 10.1016/j.fochx.2026.104594 (PMC13683454; doi:10.1016/j.fochx.2026.104594)
Supplement: Supplementary file 3 [file mmc3.pdf]

报告编号： A2240625007101001C

# 检 验 报 告

# Test Report

样 品 名 称 ： 橡胶树种子油

委 托 单 位 ： 西双版纳华坤生物科技有限责任公司

检 验 类 型 ： 委托检验

云南华测检测认证有限公司

Centre Testing International（Yunnan）Co., Ltd

www.cti-cert.com

验证码：O04X

# 检验报告



| | | | | |
|---|---|---|---|---|
| 样品信息 | 样品名称 | 橡胶树种子油 | CTI 样品编号 | KQ13138001 |
| | 商标 | / | 型号/规格/等级 | / |
| | 生产日期 | / | 批号 | / |
| | 样品数量 | 1.5L(3 瓶) | 样品状态 | 液体 |
| | 生产商 | 西双版纳华坤生物科技有限责任公司 | | |
| | 生产商地址 | 云南省西双版纳州景洪市嘎洒镇曼迈村委会曼景栋村 | | |
| 客户信息 | 委托单位 | 西双版纳华坤生物科技有限责任公司 | | |
| | 委托单位地址 | 云南省西双版纳州景洪市嘎洒镇曼迈村委会曼景栋村 | | |
| 检验信息 | 样品接收日期 | 2024 年 10 月 12 日 | 样品检验日期 | 2024 年 10 月 12 日 ~ 2024 年 10 月 23 日 |
| | 检验项目 | 气味、滋味,外观,不溶性杂质,不皂化物,碘值（以 I 计）等 18 项。 | | |
| | 检验依据 | GB 2761-2017《食品安全国家标准 食品中真菌毒素限量》,GB 2762-2022《食品安全国家标准 食品中污染物限量》,Q/XHK 0001 S-2024 《食用橡胶树种子油》。 | | |
| 检验结论 | 经检测，第 1 项至第 17 项符合 GB 2761-2017《食品安全国家标准 食品中真菌毒素限量》，GB 2762-2022《食品安全国家标准 食品中污染物限量》，Q/XHK 0001 S-2024 《食用橡胶树种子油》要求，第 18 项仅提供检测数据。<br>（检验检测专用章）<br>批准日期：2024 年 10 月 23 日 | | | |
| 备注 | ----- | | | |

云南华测检测认证有限公司 检验检测专用章

主检： 审核： 批准：

授权签字人：李艳梅

# 检验报告



**检验结果：**

| 序号 | 检验项目 | 单位 | 检验结果 | 标准要求 | 结论 | 检验方法 |
|---|---|---|---|---|---|---|
| 1 | 气味、滋味 | / | 无异味、口感良好 | 无异味、口感良好 | 符合 | GB/T 5525-2008 6 |
| 2 | 外观 | / | 黄色油状液体 | 浅黄至黄色油状液体 | 符合 | GB/T 5009.37-2003 3.1 |
| 3 | 不溶性杂质 | g/100g | 0.06 | ≤0.1 | 符合 | GB/T 15688-2008 |
| 4 | 不皂化物[#1] | g/kg | 5.90 | ≤20 | 符合 | GB/T 5535.1-2008 |
| 5 | 碘值（以 I 计）[#1] | g/100g | 144 | 120 ~ 145 | 符合 | GB/T 5532-2022 |
| 6 | 过氧化值 | g/100g | 0.051 | ≤0.25 | 符合 | GB 5009.227-2023 第一法 |
| 7 | 水分及挥发物 | g/100g | 0.08 | ≤0.2 | 符合 | GB 5009.236-2016 第二法 |
| 8 | 酸价(KOH) | mg/g | 2.1 | ≤3.0 | 符合 | GB 5009.229-2016 第二法 |
| 9 | 透明度 | / | 澄清、透明 | 澄清、透明 | 符合 | GB/T 5525-2008 5 |
| 10 | 相对密度($d_{20}^{20}$) | / | 0.9215 | 0.900 ~ 0.926 | 符合 | GB/T 5526-1985 2 |
| 11 | 皂化值（以 KOH 计）[#1] | mg/g | 195 | 175 ~ 197 | 符合 | GB/T 5534-2008 |
| 12 | 折光指数($n^{40}$) | / | 1.4682 | 1.467 ~ 1.471 | 符合 | GB/T 5527-2010 |
| 13 | 铅（以 Pb 计） | mg/kg | 未检出(定量限：0.05mg/kg) | ≤0.06 | 符合 | GB 5009.12-2023 第二法 |
| 14 | 总砷(以 As 计) | mg/kg | 未检出(定量限：0.005mg/kg) | ≤0.08 | 符合 | GB 5009.11-2024 第一篇 第二法 |
| 15 | 黄曲霉毒素 $B_1$ | μg/kg | 4.04 | ≤10 | 符合 | GB 5009.22-2016 第一法 |
| 16 | 苯并[a]芘 | μg/kg | 未检出(定量限：0.5μg/kg) | ≤10 | 符合 | GB 5009.27-2016 |
| 17 | 脂肪酸组成 | | | | | |
| | 亚麻酸(C18:3) | % | 21.5 | 14.0 ~ 30.0 | 符合 | GB 5009.168-2016 第三法 |
| | 亚油酸(C18:2) | % | 37.7 | 30.0 ~ 42.0 | 符合 | GB 5009.168-2016 第三法 |
| | 硬脂酸(C18:0) | % | 7.06 | 6.0 ~ 10.0 | 符合 | GB 5009.168-2016 第三法 |

# 检验报告

报告编号：A2240625007101001C　　　　　　　　　　　　　第 4 页 共 4 页

**检验结果：**

| 序号 | 检验项目 | 单位 | 检验结果 | 标准要求 | 结论 | 检验方法 |
|---|---|---|---|---|---|---|
| | 油酸(C18:1) | % | 24.2 | 20.0 ~ 30.0 | 符合 | GB 5009.168-2016 第三法 |
| | 棕榈酸(C16:0) | % | 8.69 | 7.0 ~ 12.0 | 符合 | GB 5009.168-2016 第三法 |
| 18 | 甾醇总量*1#1 | mg/100g | 290 | / | / | GB/T 25223-2010 |
| 以下空白 | | | | | | |

备注：　1. *1 表示该项目/方法不在 CMA 资质认定范围内。

2. #1 此项目由青岛市华测检测技术有限公司完成，CMA 资质证书编号 241520340452。

3. Q/XHK 0001 S-2024 《食用橡胶树种子油》由委托单位提供。


声明：

1.报告无批准人签字、检验检测专用章及报告骑缝章，或经涂改，以及复印报告未加盖红色检验检测专用章均视作无效。

2.未经本公司批准，不得部分复制本报告。

3.样品信息由客户提供，本报告检测结果仅对受检样品负责。

4.不得擅自使用检测结果进行不当宣传。

5.如果对检测结果有异议，请于收到报告之日起 7 个工作日内提出，逾期不予受理。

6.扫描报告首页二维码，或登陆官方网站 https://mycti.cti-cert.com 输入报告编号和报告首页验证码，即可查询报告真伪；如有疑问，请联系邮箱：fdd.checkreport@cti-cert.com。


*** 报告结束 ***
