# Supplementary material for: Monoglyceride-structured rubber seed oil oleogels as a partial cocoa butter replacement in chocolate: physical properties, oxidative stability and applications
Source: Food Chem X. 2026 Oct 11;39:104594. doi: 10.1016/j.fochx.2026.104594 (PMC13683454; doi:10.1016/j.fochx.2026.104594)
Supplement: Supplementary file 4 [file mmc4.pdf]

检测报告

CDF25-0005759-01　　发布日期：2025-10-22

客户名称：　西双版纳华坤生物科技有限责任公司

客户地址：　景洪市嘎洒镇曼迈村委会曼景栋村

样品名称：　橡胶树种子

样品批号：　/

生产日期：　/

生产商：　/

以上样品及信息由客户提供及确认, SGS 不承担证实客户提供信息的准确性、适当性和(或)完整性责任。

样品接收日期：　2025-10-16

检测周期：　2025-10-16 ~ 2025-10-22

检测要求：　根据客户要求检测

检测方法：　请参见下一页。

检测结果：　请参见下一页。

检验检测专用章
Inspection & Testing Services

SGS 授权签字人

扫码查看在线报告
CDF25-0005759-01
报告验真请访问:
check.sgsonline.com.cn

通标标准技术服务有限公司四川分公司

除非另有书面协议，本报告由本公司依据《 服务通用条款 》出具。
《 服务通用条款 》印刷在正本报告纸背面，或通过 https://www.sgs.com/en/Terms-and-Conditions 查询。请特别关注其中涉及责任限定，赔偿以及司法管辖的相关条款。任何报告的持有方需知悉，此报告内容仅反映SGS受限于客户指示下，且在当时所得结论。SGS仅对其客户负责，并且此报告不能免除交易各方根据交易文件所享有的权利和应履行的义务。未获得本公司书面批准，本文件不得进行复制，全文除外。任何未经授权对报告内容及形式进行的修改、伪造或扭曲都是违法行为，违法者将会被追究法律责任。除非另有声明，本测试报告所示结果仅涉及受测试样品。
注意：检测/检验报告或证书的真实性，请通过电话(86－755) 83071443或邮箱 CN.Doccheck@sgs.com 查询。

No.1, 5/F., Building D1, No. 1666, Section 2 of Chenglong Road, Economic and Technological Development Zone, Longquanyi District, Chengdu, Sichuan, China 610100　t (86–28) 8481 0338　www.sgsgroup.com.cn
中国・四川・成都市龙泉驿区经济技术开发区成龙大道二段1666号 D1栋05层1号　邮编：610100　t (86–28) 8481 0338　sgs.china@sgs.com

Member of the SGS Group (SGS SA)

检测报告

CDF25-0005759-01　　　　发布日期：2025-10-22

检测样品描述：

样品编号	SGS 样品 ID	样品描述
1	CDF25-0005759-0001	袋装样品　1 袋

检测结果：

农药残留

检测方法：　BS EN 15662:2018 HPLC-MS/MS

编号	检测项目	单位	CAS No.	检测结果 1	定量限
1	#混杀威（3,4,5-混杀威）	mg/kg	2686-99-9	ND	0.01
2	阿维菌素	mg/kg	71751-41-2	ND	0.01
3	乙酰甲胺磷	mg/kg	30560-19-1	ND	0.01
4	啶虫脒	mg/kg	135410-20-7	ND	0.01
5	#活化酯/苯并噻二唑	mg/kg	135158-54-2	ND	0.01
6	涕灭威	mg/kg	116-06-3	ND	0.01
7	#砜灭威（涕灭威砜）	mg/kg	1646-88-4	ND	0.01
8	#涕灭威亚砜	mg/kg	1646-87-3	ND	0.01
9	#酰嘧磺隆	mg/kg	120923-37-7	ND	0.01
10	#吲唑磺菌胺	mg/kg	348635-87-0	ND	0.01
11	#莎稗磷	mg/kg	64249-01-0	ND	0.01
12	#杀螨特	mg/kg	140-57-8	ND	0.01
13	#莠去津	mg/kg	1912-24-9	ND	0.01
14	#氧环唑	mg/kg	60207-31-0	ND	0.01
15	#甲基吡恶磷	mg/kg	35575-96-3	ND	0.01
16	谷硫磷（保棉磷）	mg/kg	86-50-0	ND	0.01
17	嘧菌酯（腈嘧菌酯）	mg/kg	131860-33-8	ND	0.01
18	苯霜灵	mg/kg	71626-11-4	ND	0.01
19	#解草嗪	mg/kg	98730-04-2	ND	0.01
20	#苄嘧磺隆	mg/kg	83055-99-6	ND	0.01
21	#地散磷	mg/kg	741-58-2	ND	0.01
22	#灭草松	mg/kg	25057-89-0	ND	0.01
23	#苯螨特	mg/kg	29104-30-1	ND	0.01
24	联苯三唑醇	mg/kg	55179-31-2	ND	0.01
25	啶酰菌胺	mg/kg	188425-85-6	ND	0.01
26	#除草定	mg/kg	314-40-9	ND	0.01
27	#溴丁酰草胺	mg/kg	74712-19-9	ND	0.01
28	溴苯腈	mg/kg	1689-84-5	ND	0.01
29	乙嘧酚磺酸酯	mg/kg	41483-43-6	ND	0.01
30	噻嗪酮	mg/kg	69327-76-0	ND	0.01

除非另有书面协议，本报告由本公司依据《 服务通用条款 》出具。
《 服务通用条款 》印刷在正本报告纸背面，或通过 https://www.sgs.com/en/Terms-and-Conditions 查询。请特别关注其中涉及责任限定，赔偿以及司法管辖的相关条款。任何报告的持有方需知悉，此报告内容仅反映SGS受限于客户指示下，且在当时所得结论。SGS仅对其客户负责，并且此报告不能免除交易各方根据交易文件所享有的权利和应履行的义务。未获得本公司书面批准，本文件不得进行复制，全文除外。任何未经授权对报告内容及形式进行的修改、伪造或扭曲都是违法行为，违法者将会被追究法律责任。除非另有声明，本测试报告所示结果仅涉及受测试样品。
注意：检测/检验报告或证书的真实性，请通过电话 (86 – 755) 83071443 或邮箱 CN.Doccheck@sgs.com 查询。

No.1, 5/F., Building D1, No. 1666, Section 2 of Chenglong Road, Economic and Technological Development Zone, Longquanyi District, Chengdu, Sichuan, China 610100　t (86–28) 8481 0338　www.sgsgroup.com.cn
中国・四川・成都市龙泉驿区经济技术开发区成龙大道二段1666号 D1栋05层1号　邮编：610100　t (86–28) 8481 0338　sgs.china@sgs.com

Member of the SGS Group (SGS SA)

通标标准技术服务有限公司四川分公司
检验检测专用章
Inspection & Testing Services
通标标准技术服务有限公司四川分公司
农产食品实验室

编号	检测项目	单位	CAS No.	检测结果 1	定量限
31	#氟丙嘧草酯	mg/kg	134605-64-4	ND	0.01
32	#丁酮威	mg/kg	34681-10-2	ND	0.01
33	#丁酮砜威	mg/kg	34681-23-7	ND	0.01
34	甲萘威	mg/kg	63-25-2	ND	0.01
35	多菌灵	mg/kg	10605-21-7	ND	0.01
36	呋喃丹（虫螨威/克百威）	mg/kg	1563-66-2	ND	0.01
37	3-羟基虫螨威（3-羟基克百威）	mg/kg	16655-82-6	ND	0.01
38	萎锈灵	mg/kg	5234-68-4	ND	0.01
39	#唑草酮	mg/kg	128639-02-1	ND	0.01
40	#加普胺	mg/kg	104030-54-8	ND	0.01
41	#氯虫苯甲酰胺	mg/kg	500008-45-7	ND	0.01
42	#灭幼脲	mg/kg	57160-47-1	ND	0.01
43	#氯草灵	mg/kg	1967-16-4	ND	0.01
44	#杀虫脒	mg/kg	6164-98-3	ND	0.01
45	#氟啶脲	mg/kg	71422-67-8	ND	0.01
46	#氯草敏	mg/kg	1698-60-8	ND	0.01
47	#氯嘧磺隆	mg/kg	90982-32-4	ND	0.01
48	#绿麦隆	mg/kg	15545-48-9	ND	0.01
49	#枯草隆	mg/kg	1982-47-4	ND	0.01
50	#氯磺隆	mg/kg	64902-72-3	ND	0.01
51	#环虫酰肼	mg/kg	143807-66-3	ND	0.01
52	#吲哚酮草酯	mg/kg	142891-20-1	ND	0.01
53	#环庚草醚	mg/kg	87818-31-3	ND	0.01
54	#醚磺隆	mg/kg	94593-91-6	ND	0.01
55	#烯草酮	mg/kg	99129-21-2	ND	0.01
56	四螨嗪	mg/kg	74115-24-5	ND	0.01
57	#氯甲酰草胺	mg/kg	84496-56-0	ND	0.01
58	#调果酸（调采酸）	mg/kg	101-10-0	ND	0.01
59	#氯酯磺草胺	mg/kg	147150-35-4	ND	0.01
60	噻虫胺（可尼丁）	mg/kg	210880-92-5	ND	0.01
61	#氰草津	mg/kg	21725-46-2	ND	0.01
62	氰霜唑	mg/kg	120116-88-3	ND	0.01
63	#环丙酸酰胺	mg/kg	113136-77-9	ND	0.01
64	#环草敌（草灭特）	mg/kg	1134-23-2	ND	0.01
65	#环丙嘧磺隆	mg/kg	136849-15-5	ND	0.01
66	#环氟菌胺	mg/kg	180409-60-3	ND	0.01
67	霜脲氰	mg/kg	57966-95-7	ND	0.01
68	嘧菌环胺	mg/kg	121552-61-2	ND	0.01
69	#杀草隆	mg/kg	42609-52-9	ND	0.01
70	#内吸磷	mg/kg	8065-48-3	ND	0.01

除非另有书面协议，本报告由本公司依据《 服务通用条款 》出具。
《 服务通用条款 》印刷在正本报告纸背面，或通过 https://www.sgs.com/en/Terms-and-Conditions 查询。请特别关注其中涉及责任限定，赔偿以及司法管辖的相关条款。任何报告的持有方需知悉，此报告内容仅反映SGS受限于客户指示下，且在当时所得结论。SGS仅对其客户负责，并且此报告不能免除交易各方根据交易文件所享有的权利和应履行的义务。未获得本公司书面批准，本文件不得进行复制，全文除外。任何未经授权对报告内容及形式进行的修改、伪造或扭曲都是违法行为，违法者将会被追究法律责任。除非另有声明，本测试报告所示结果仅涉及受测试样品。
注意：检测/检验报告或证书的真实性，请通过电话(86–755) 83071443或邮箱 CN.Doccheck@sgs.com查询。

通标标准技术服务有限公司四川分公司
检验检测专用章
Inspection & Testing Services
农产品食品实验室

No.1, 5/F., Building D1, No. 1666, Section 2 of Chenglong Road, Economic and Technological Development Zone, Longquanyi District, Chengdu, Sichuan, China 610100　t (86–28) 8481 0338　www.sgsgroup.com.cn
中国・四川・成都市龙泉驿区经济技术开发区成龙大道二段1666号 D1栋05层1号　邮编：610100　t (86–28) 8481 0338　sgs.china@sgs.com

Member of the SGS Group (SGS SA)

编号	检测项目	单位	CAS No.	检测结果 1	定量限
71	甲基砜内吸磷	mg/kg	17040-19-6	ND	0.01
72	#亚砜磷	mg/kg	301-12-2	ND	0.01
73	#甜菜胺（甜菜安）	mg/kg	13684-56-5	ND	0.01
74	#丁醚脲（丁嘧脲）	mg/kg	80060-09-9	ND	0.01
75	#燕麦敌（丁变敌）	mg/kg	2303-16-4	ND	0.01
76	#2,4-滴丙酸	mg/kg	120-36-5	ND	0.01
77	#双氯氰菌胺	mg/kg	139920-32-4	ND	0.01
78	#双氯磺草胺	mg/kg	145701-21-9	ND	0.01
79	百治磷	mg/kg	141-66-2	ND	0.01
80	乙霉威	mg/kg	87130-20-9	ND	0.01
81	苯醚甲环唑	mg/kg	119446-68-3	ND	0.01
82	除虫脲	mg/kg	35367-38-5	ND	0.01
83	#吡氟酰草胺	mg/kg	83164-33-4	ND	0.01
84	#哌草丹	mg/kg	61432-55-1	ND	0.01
85	#异戊乙净（阔草净）	mg/kg	22936-75-0	ND	0.01
86	#二甲嘧酚	mg/kg	5221-53-4	ND	0.01
87	乐果	mg/kg	60-51-5	ND	0.01
88	烯酰吗啉	mg/kg	110488-70-5	ND	0.01
89	烯唑醇	mg/kg	83657-24-3	ND	0.01
90	#敌螨普	mg/kg	39300-45-3	ND	0.01
91	#丁诺特呋喃（呋虫胺）	mg/kg	165252-70-0	ND	0.01
92	#特乐酚	mg/kg	1420-07-1	ND	0.01
93	敌草隆	mg/kg	330-54-1	ND	0.01
94	#甲氨基阿维菌素苯甲酸盐	mg/kg	155569-91-8	ND	0.01
95	#苯氧菌胺	mg/kg	133408-50-1	ND	0.01
96	氟环唑	mg/kg	106325-08-0 /133855-98-8	ND	0.01
97	#戊草丹	mg/kg	85785-20-2	ND	0.01
98	#乙硫苯威	mg/kg	29973-13-5	ND	0.01
99	#乙硫苯威砜	mg/kg	53380-23-7	ND	0.01
100	#乙硫苯威亚砜	mg/kg	53380-22-6	ND	0.01
101	#乙虫清	mg/kg	181587-01-9	ND	0.01
102	#乙嘧酚	mg/kg	23947-60-6	ND	0.01
103	#乙氧嘧磺隆	mg/kg	126801-58-9	ND	0.01
104	#乙氧苯草胺	mg/kg	79540-50-4	ND	0.01
105	#乙螨唑	mg/kg	153233-91-1	ND	0.01
106	咪唑菌酮	mg/kg	161326-34-7	ND	0.01
107	喹螨醚	mg/kg	120928-09-8	ND	0.01
108	腈苯唑	mg/kg	114369-43-6	ND	0.01
109	#甲呋酰胺	mg/kg	24691-80-3	ND	0.01

通标标准技术服务有限公司四川分公司
检验检测专用章
Inspection & Testing Services
通标标准技术服务有限公司四川分公司
农产品食品实验室

除非另有书面协议，本报告由本公司依据《 服务通用条款 》出具。
《 服务通用条款 》印刷在正本报告纸背面，或通过 https://www.sgs.com/en/Terms-and-Conditions 查询。请特别关注其中涉及责任限定，赔偿以及司法管辖的相关条款。任何报告的持有方需知悉，此报告内容仅反映SGS受限于客户指示下，且在当时所得结论。SGS仅对其客户负责，并且此报告不能免除交易各方根据交易文件所享有的权利和应履行的义务。未获得本公司书面批准，本文件不得进行复制，全文除外。任何未经授权对报告内容及形式进行的修改、伪造或扭曲都是违法行为，违法者将会被追究法律责任。除非另有声明，本测试报告所示结果仅涉及受测试样品。
注意：检测/检验报告或证书的真实性，请通过电话 (86－755) 83071443 或邮箱 CN.Doccheck@sgs.com 查询。

No.1, 5/F., Building D1, No. 1666, Section 2 of Chenglong Road, Economic and Technological Development Zone, Longquanyi District, Chengdu, Sichuan, China 610100 t (86–28) 8481 0338 www.sgsgroup.com.cn
中国・四川・成都市龙泉驿区经济技术开发区成龙大道二段1666号 D1栋05层1号 邮编：610100 t (86–28) 8481 0338 sgs.china@sgs.com

Member of the SGS Group (SGS SA)

编号	检测项目	单位	CAS No.	检测结果 1	定量限
110	环酰菌胺	mg/kg	126833-17-8	ND	0.01
111	#仲丁威	mg/kg	3766-81-2	ND	0.01
112	#稻瘟酰胺（氰菌胺）	mg/kg	115852-48-7	ND	0.01
113	#恶唑禾草灵	mg/kg	66441-23-4	ND	0.01
114	双氧威（苯醚威）	mg/kg	72490-01-8 /79127-80-3	ND	0.01
115	苯锈啶	mg/kg	67306-00-7	ND	0.01
116	丁苯吗啉	mg/kg	67564-91-4	ND	0.01
117	唑螨酯	mg/kg	111812-58-9	ND	0.01
118	倍硫磷	mg/kg	55-38-9	ND	0.01
119	#倍硫磷砜	mg/kg	3761-42-0	ND	0.01
120	#倍硫磷亚砜	mg/kg	3761-41-9	ND	0.01
121	#四唑酰草胺（四唑啉酮）	mg/kg	158237-07-1	ND	0.01
122	#嘧菌腙	mg/kg	89269-64-7	ND	0.01
123	氟虫腈	mg/kg	120068-37-3	ND	0.01
124	#氟虫腈亚砜	mg/kg	120067-83-6	ND	0.01
125	#氟虫腈砜	mg/kg	120068-36-2	ND	0.01
126	#甲基麦草氟异丙酯（麦草氟甲酯）	mg/kg	52756-25-9	ND	0.01
127	#啶嘧磺隆	mg/kg	104040-78-0	ND	0.01
128	氟啶虫酰胺	mg/kg	158062-67-0	ND	0.01
129	#双氟磺草胺	mg/kg	145701-23-1	ND	0.01
130	#吡氟禾草灵和精吡氟禾草灵	mg/kg	69806-50-4 /79241-46-6	ND	0.01
131	#氟啶胺	mg/kg	79622-59-6	ND	0.01
132	#氟虫双酰胺（氟苯虫酰胺）	mg/kg	272451-65-7	ND	0.01
133	#氟唑磺隆	mg/kg	181274-17-9	ND	0.01
134	咯菌腈	mg/kg	131341-86-1	ND	0.01
135	#氟噻草胺	mg/kg	142459-58-3	ND	0.01
136	氟虫脲	mg/kg	101463-69-8	ND	0.01
137	#丁虫腈	mg/kg	704886-18-0	ND	0.01
138	#唑嘧磺草胺	mg/kg	98967-40-9	ND	0.01
139	#丙炔氟草胺	mg/kg	103361-09-7	ND	0.02
140	#氟吗啉	mg/kg	211867-47-9	ND	0.01
141	#伏草隆（氟草隆）	mg/kg	2164-17-2	ND	0.01
142	氟吡菌胺	mg/kg	239110-15-7	ND	0.01
143	#氯氟吡氧乙酸异辛酯	mg/kg	81406-37-3	ND	0.01
144	#磺菌胺	mg/kg	106917-52-6	ND	0.01
145	#氟噻乙草酯（氟赛乙草酯）	mg/kg	117337-19-6	ND	0.01
146	粉唑醇	mg/kg	76674-21-0	ND	0.01
147	#氟磺胺草醚	mg/kg	72178-02-0	ND	0.01
148	#氯吡脲	mg/kg	68157-60-8	ND	0.01

除非另有书面协议，本报告由本公司依据《 服务通用条款 》出具。
《 服务通用条款 》印刷在正本报告纸背面，或通过 https://www.sgs.com/en/Terms-and-Conditions 查询。请特别关注其中涉及责任限定，赔偿以及司法管辖的相关条款。任何报告的持有方需知悉，此报告内容仅反映SGS受限于客户指示下，且在当时所得结论。SGS仅对其客户负责，并且此报告不能免除交易各方根据交易文件所享有的权利和应履行的义务。未获得本公司书面批准，本文件不得进行复制，全文除外。任何未经授权对报告内容及形式进行的修改、伪造或扭曲都是违法行为，违法者将会被追究法律责任。除非另有声明，本测试报告所示结果仅涉及受测试样品。
注意：检测/检验报告或证书的真实性，请通过电话 (86－755) 83071443 或邮箱 CN.Doccheck@sgs.com 查询。

检验检测专用章 Inspection & Testing Services
通标标准技术服务有限公司四川分公司 农产品实验室

No.1, 5/F., Building D1, No. 1666, Section 2 of Chenglong Road, Economic and Technological Development Zone, Longquanyi District, Chengdu, Sichuan, China 610100 t (86–28) 8481 0338 www.sgsgroup.com.cn
中国・四川・成都市龙泉驿区经济技术开发区成龙大道二段1666号 D1栋05层1号 邮编：610100 t (86–28) 8481 0338 sgs.china@sgs.com

Member of the SGS Group (SGS SA)

编号	检测项目	单位	CAS No.	检测结果<br>1	定量限
149	#福拉比（呋比菌胺）	mg/kg	123572-88-3	ND	0.01
150	#呋线威	mg/kg	65907-30-4	ND	0.01
151	#氯吡嘧磺隆	mg/kg	100784-20-1	ND	0.01
152	#氟吡禾灵	mg/kg	69806-34-4	ND	0.01
153	#氟吡乙禾灵	mg/kg	87237-48-7	ND	0.01
154	#氟吡甲禾灵	mg/kg	69806-40-2	ND	0.01
155	己唑醇	mg/kg	79983-71-4	ND	0.01
156	#氟铃脲	mg/kg	86479-06-3	ND	0.01
157	噻螨酮	mg/kg	78587-05-0	ND	0.01
158	抑霉唑（烯菌灵）	mg/kg	35554-44-0	ND	0.01
159	#醚草酸甲酯	mg/kg	81405-85-8	ND	0.01
160	#甲氧咪草烟	mg/kg	114311-32-9	ND	0.01
161	#甲咪唑烟酸	mg/kg	104098-48-8	ND	0.01
162	#咪唑喹啉酸（灭草喹）	mg/kg	81335-37-7	ND	0.01
163	#亚胺唑	mg/kg	86598-92-7	ND	0.01
164	吡虫啉	mg/kg	138261-41-3	ND	0.01
165	#氯噻啉	mg/kg	105843-36-5	ND	0.01
166	#茚草酮	mg/kg	133220-30-1	ND	0.01
167	茚虫威（安打）	mg/kg	173584-44-6	ND	0.01
168	#甲基碘磺隆（钠盐）	mg/kg	144550-36-7	ND	0.01
169	#碘苯腈	mg/kg	1689-83-4	ND	0.01
170	#异稻瘟净	mg/kg	26087-47-8	ND	0.01
171	异菌脲	mg/kg	36734-19-7	ND	0.01
172	缬霉威	mg/kg	140923-17-7	ND	0.01
173	#异丙威	mg/kg	2631-40-5	ND	0.01
174	#稻瘟灵	mg/kg	50512-35-1	ND	0.01
175	#异丙隆	mg/kg	34123-59-6	ND	0.01
176	#异唑隆（异恶隆）	mg/kg	55861-78-4	ND	0.01
177	#双苯恶唑酸	mg/kg	163520-33-0	ND	0.01
178	#异恶氟草（异恶唑草酮）	mg/kg	141112-29-0	ND	0.01
179	醚菌酯（苯氧菊酯）	mg/kg	143390-89-0	ND	0.01
180	#乳氟禾草灵	mg/kg	77501-63-4	ND	0.01
181	#环草定（环草啶）	mg/kg	2164-08-1	ND	0.01
182	利谷隆	mg/kg	330-55-2	ND	0.01
183	虱螨脲（氟丙氧脲）	mg/kg	103055-07-8	ND	0.01
184	马拉硫磷	mg/kg	121-75-5	ND	0.01
185	双炔酰菌胺	mg/kg	374726-62-2	ND	0.01
186	#2 甲 4 氯	mg/kg	94-74-6	ND	0.01
187	#苯噻酰草胺	mg/kg	73250-68-7	ND	0.01
188	#吡咯解草酯（吡咯二酸二乙酯）	mg/kg	135590-91-9	ND	0.01

除非另有书面协议，本报告由本公司依据《 服务通用条款 》出具。
《 服务通用条款 》印刷在正本报告纸背面，或通过 https://www.sgs.com/en/Terms-and-Conditions 查询。请特别关注其中涉及责任限定，赔偿以及司法管辖的相关条款。任何报告的持有方需知悉，此报告内容仅反映SGS受限于客户指示下，且在当时所得结论。SGS仅对其客户负责，并且此报告不能免除交易各方根据交易文件所享有的权利和应履行的义务。未获得本公司书面批准，本文件不得进行复制，全文除外。任何未经授权对报告内容及形式进行的修改、伪造或扭曲都是违法行为，违法者将会被追究法律责任。除非另有声明，本测试报告所示结果仅涉及受测试样品。
注意：检测/检验报告或证书的真实性，请通过电话 (86 –755) 83071443 或邮箱 CN.Doccheck@sgs.com 查询。

No.1, 5/F., Building D1, No. 1666, Section 2 of Chenglong Road, Economic and Technological Development Zone, Longquanyi District, Chengdu, Sichuan, China 610100 t (86–28) 8481 0338 www.sgsgroup.com.cn
中国・四川・成都市龙泉驿区经济技术开发区成龙大道二段1666号 D1栋05层1号 邮编：610100 t (86–28) 8481 0338 sgs.china@sgs.com

Member of the SGS Group (SGS SA)

通标标准技术服务有限公司四川分公司 检验检测专用章 Inspection & Testing Services
通标标准技术服务有限公司四川分公司 农产食品实验室

编号	检测项目	单位	CAS No.	检测结果 1	定量限
189	嘧菌胺	mg/kg	110235-47-7	ND	0.01
190	#甲基二磺隆	mg/kg	208465-21-8	ND	0.01
191	#硝磺草酮	mg/kg	104206-82-8	ND	0.01
192	#甲霜灵和精甲霜灵	mg/kg	57837-19-1 /70630-17-0	ND	0.01
193	甲霜灵（阿普隆）（瑞毒霜）	mg/kg	57837-19-1	ND	0.01
194	苯嗪草酮（灭它通）	mg/kg	41394-05-2	ND	0.01
195	#叶菌唑	mg/kg	125116-23-6	ND	0.01
196	#甲草苯噻隆（甲草苯隆）	mg/kg	18691-97-9	ND	0.01
197	甲胺磷	mg/kg	10265-92-6	ND	0.01
198	杀扑磷	mg/kg	950-37-8	ND	0.01
199	甲硫威（灭虫威）	mg/kg	2032-65-7	ND	0.01
200	灭多威	mg/kg	16752-77-5	ND	0.01
201	甲氧虫酰肼	mg/kg	161050-58-4	ND	0.01
202	异丙甲草胺	mg/kg	51218-45-2	ND	0.01
203	#速灭威	mg/kg	1129-41-5	ND	0.01
204	苯菌酮	mg/kg	220899-03-6	ND	0.01
205	#甲磺隆	mg/kg	74223-64-6	ND	0.01
206	#禾草敌	mg/kg	2212-67-1	ND	0.01
207	久效磷	mg/kg	6923-22-4	ND	0.01
208	腈菌唑（灭克落）	mg/kg	88671-89-0	ND	0.01
209	#萘草胺	mg/kg	132-66-1	ND	0.01
210	#烟嘧磺隆	mg/kg	111991-09-4	ND	0.01
211	#氟草敏（哒草伏）	mg/kg	27314-13-2	ND	0.01
212	#氟酰脲	mg/kg	116714-46-6	ND	0.01
213	#呋酰胺	mg/kg	58810-48-3	ND	0.01
214	氧乐果	mg/kg	1113-02-6	ND	0.01
215	#嘧苯胺磺隆	mg/kg	213464-77-8	ND	0.01
216	#丙炔噁草酮	mg/kg	39807-15-3	ND	0.01
217	恶草酮	mg/kg	19666-30-9	ND	0.01
218	恶霜灵	mg/kg	77732-09-3	ND	0.01
219	杀线威	mg/kg	23135-22-0	ND	0.01
220	#氯恶嗪草	mg/kg	153197-14-9	ND	0.01
221	#氧化萎锈灵	mg/kg	5259-88-1	ND	0.01
222	纹枯脲（戊菌隆）	mg/kg	66063-05-6	ND	0.01
223	#五氟磺草胺	mg/kg	219714-96-2	ND	0.01
224	甜菜宁	mg/kg	13684-63-4	ND	0.01
225	伏杀硫磷	mg/kg	2310-17-0	ND	0.01
226	#硫环磷	mg/kg	947-02-4	ND	0.01
227	#甲基硫环磷	mg/kg	5120-23-0	ND	0.01

检验检测专用章 Inspection & Testing Services

除非另有书面协议，本报告由本公司依据《 服务通用条款 》出具。
《 服务通用条款 》印刷在正本报告纸背面，或通过 https://www.sgs.com/en/Terms-and-Conditions 查询。请特别关注其中涉及责任限定，赔偿以及司法管辖的相关条款。任何报告的持有方需知悉，此报告内容仅反映SGS受限于客户指示下，且在当时所得结论。SGS仅对其客户负责，并且此报告不能免除交易各方根据交易文件所享有的权利和应履行的义务。未获得本公司书面批准，本文件不得进行复制，全文除外。任何未经授权对报告内容及形式进行的修改、伪造或扭曲都是违法行为，违法者将会被追究法律责任。除非另有声明，本测试报告所示结果仅涉及受测试样品。
注意：检测/检验报告或证书的真实性，请通过电话 (86－755) 83071443 或邮箱 CN.Doccheck@sgs.com 查询。

No.1, 5/F., Building D1, No. 1666, Section 2 of Chenglong Road, Economic and Technological Development Zone, Longquanyi District, Chengdu, Sichuan, China 610100 t (86–28) 8481 0338 www.sgsgroup.com.cn
中国・四川・成都市龙泉驿区经济技术开发区成龙大道二段1666号 D1栋05层1号 邮编：610100 t (86–28) 8481 0338 sgs.china@sgs.com

Member of the SGS Group (SGS SA)

编号	检测项目	单位	CAS No.	检测结果 1	定量限
228	亚胺硫磷	mg/kg	732-11-6	ND	0.01
229	辛硫磷	mg/kg	14816-18-3	ND	0.01
230	#氟吡草胺	mg/kg	137641-05-5	ND	0.01
231	#啶氧菌酯	mg/kg	117428-22-5	ND	0.01
232	抗蚜威	mg/kg	23103-98-2	ND	0.01
233	#烯丙苯噻唑	mg/kg	27605-76-1	ND	0.01
234	#咪鲜胺	mg/kg	67747-09-5	ND	0.01
235	#茉莉酮（茉莉酸导体）	mg/kg	158474-72-7	ND	0.01
236	#猛杀威	mg/kg	2631-37-0	ND	0.01
237	#毒草胺	mg/kg	1918-16-7	ND	0.01
238	霜霉威	mg/kg	24579-73-5	ND	0.01
239	#恶草酸	mg/kg	111479-05-1	ND	0.01
240	炔螨特	mg/kg	2312-35-8	ND	0.01
241	#异丙草胺	mg/kg	86763-47-5	ND	0.01
242	残杀威	mg/kg	114-26-1	ND	0.01
243	苄草丹	mg/kg	52888-80-9	ND	0.01
244	#氟磺隆	mg/kg	94125-34-5	ND	0.01
245	吡蚜酮（吡嗪酮）	mg/kg	123312-89-0	ND	0.01
246	吡唑醚菌酯（唑菌胺酯）（百克敏）	mg/kg	175013-18-0	ND	0.01
247	#唑菌酯	mg/kg	862588-11-2	ND	0.01
248	#吡唑特	mg/kg	58011-68-0	ND	0.01
249	#吡菌磷	mg/kg	13457-18-6	ND	0.01
250	#乙基吡嘧磺隆（百速隆）	mg/kg	93697-74-6	ND	0.01
251	#苄草唑	mg/kg	71561-11-0	ND	0.01
252	#稗草丹	mg/kg	88678-67-5	ND	0.01
253	哒螨灵	mg/kg	96489-71-3	ND	0.01
254	#啶虫丙醚	mg/kg	179101-81-6	ND	0.01
255	#哒嗪硫磷	mg/kg	119-12-0	ND	0.01
256	啶斑肟（比芬诺）	mg/kg	88283-41-4	ND	0.01
257	#环酯草醚	mg/kg	135186-78-6	ND	0.01
258	嘧霉胺	mg/kg	53112-28-0	ND	0.01
259	#嘧螨醚	mg/kg	105779-78-0	ND	0.01
260	#吡丙醚（蚊蝇醚）	mg/kg	95737-68-1	ND	0.01
261	#咯喹酮（百快隆）	mg/kg	57369-32-1	ND	0.01
262	#灭藻醌	mg/kg	2797-51-5	ND	0.01
263	喹氧灵	mg/kg	124495-18-7	ND	0.01
264	#喹禾灵和精喹禾灵	mg/kg	100646-51-3 /76578-14-8	ND	0.01
265	#砜嘧磺隆	mg/kg	122931-48-0	ND	0.01
266	#鱼藤酮	mg/kg	83-79-4	ND	0.01

除非另有书面协议，本报告由本公司依据《 服务通用条款 》出具。
《 服务通用条款 》印刷在正本报告纸背面，或通过 https://www.sgs.com/en/Terms-and-Conditions 查询。请特别关注其中涉及责任限定，赔偿以及司法管辖的相关条款。任何报告的持有方需知悉，此报告内容仅反映SGS受限于客户指示下，且在当时所得结论。SGS仅对其客户负责，并且此报告不能免除交易各方根据交易文件所享有的权利和应履行的义务。未获得本公司书面批准，本文件不得进行复制，全文除外。任何未经授权对报告内容及形式进行的修改、伪造或扭曲都是违法行为，违法者将会被追究法律责任。除非另有声明，本测试报告所示结果仅涉及受测试样品。
注意：检测/检验报告或证书的真实性，请通过电话(86 –755) 83071443或邮箱 CN.Doccheck@sgs.com 查询。

No.1, 5/F., Building D1, No. 1666, Section 2 of Chenglong Road, Economic and Technological Development Zone, Longquanyi District, Chengdu, Sichuan, China 610100 t (86–28) 8481 0338 www.sgsgroup.com.cn
中国・四川・成都市龙泉驿区经济技术开发区成龙大道二段1666号 D1栋05层1号 邮编：610100 t (86–28) 8481 0338 sgs.china@sgs.com

通标标准技术服务有限公司四川分公司 检验检测专用章 Inspection & Testing Services
通标标准技术服务有限公司四川分公司 农产食品实验室

编号	检测项目	单位	CAS No.	检测结果 1	定量限
267	#单甲脒	mg/kg	33089-74-6	ND	0.01
268	#稀禾定	mg/kg	74051-80-2	ND	0.01
269	#西玛津	mg/kg	122-34-9	ND	0.01
270	#硅氟唑	mg/kg	149508-90-7	ND	0.01
271	#西草净	mg/kg	1014-70-6	ND	0.01
272	螺螨酯	mg/kg	148477-71-8	ND	0.01
273	#螺虫乙酯	mg/kg	203313-25-1	ND	0.01
274	螺恶茂胺（螺环菌胺）	mg/kg	118134-30-8	ND	0.01
275	#磺草酮	mg/kg	99105-77-8	ND	0.01
276	#乙黄隆（磺酰磺隆）	mg/kg	141776-32-1	ND	0.01
277	#氟啶虫胺腈	mg/kg	946578-00-3	ND	0.01
278	戊唑醇	mg/kg	107534-96-3	ND	0.01
279	虫酰肼	mg/kg	112410-23-8	ND	0.01
280	吡螨胺	mg/kg	119168-77-3	ND	0.02
281	#特丁隆	mg/kg	34014-18-1	ND	0.01
282	氟苯脲	mg/kg	83121-18-0	ND	0.01
283	#噻吩草胺（甲氧噻草胺）	mg/kg	96491-05-3	ND	0.01
284	噻菌灵	mg/kg	148-79-8	ND	0.01
285	噻虫啉	mg/kg	111988-49-9	ND	0.01
286	噻虫嗪	mg/kg	153719-23-4	ND	0.01
287	#噻唑烟酸（噻草啶）	mg/kg	117718-60-2	ND	0.01
288	#赛苯隆	mg/kg	51707-55-2	ND	0.01
289	#噻吩磺隆（阔叶散）	mg/kg	79277-27-3	ND	0.01
290	#噻呋酰胺	mg/kg	130000-40-7	ND	0.01
291	#禾草丹	mg/kg	28249-77-6	ND	0.01
292	#硫双威	mg/kg	59669-26-0	ND	0.01
293	#久效威砜	mg/kg	39184-59-3	ND	0.01
294	#久效威亚砜	mg/kg	39184-27-5	ND	0.01
295	#甲基硫菌灵	mg/kg	23564-05-8	ND	0.01
296	#噻酰菌胺	mg/kg	223580-51-6	ND	0.01
297	#唑虫酰胺	mg/kg	129558-76-5	ND	0.01
298	#苯草酮（甲氧基草酮）	mg/kg	87820-88-0	ND	0.01
299	#三唑酮	mg/kg	43121-43-3	ND	0.01
300	三唑醇	mg/kg	55219-65-3	ND	0.01
301	#醚苯磺隆	mg/kg	82097-50-5	ND	0.01
302	三唑磷	mg/kg	24017-47-8	ND	0.01
303	#苯磺隆	mg/kg	101200-48-0	ND	0.01
304	#敌百虫	mg/kg	52-68-6	ND	0.01
305	#氯啶菌酯	mg/kg	902760-40-1	ND	0.01
306	三环唑	mg/kg	41814-78-2	ND	0.01

通标标准技术服务有限公司四川分公司 检验检测专用章 Inspection & Testing Services
通标标准技术服务有限公司四川分公司 农产品食品实验室

除非另有书面协议，本报告由本公司依据《 服务通用条款 》出具。
《 服务通用条款 》印刷在正本报告纸背面，或通过 https://www.sgs.com/en/Terms-and-Conditions 查询。请特别关注其中涉及责任限定，赔偿以及司法管辖的相关条款。任何报告的持有方需知悉，此报告内容仅反映SGS受限于客户指示下，且在当时所得结论。SGS仅对其客户负责，并且此报告不能免除交易各方根据交易文件所享有的权利和应履行的义务。未获得本公司书面批准，本文件不得进行复制，全文除外。任何未经授权对报告内容及形式进行的修改、伪造或扭曲都是违法行为，违法者将会被追究法律责任。除非另有声明，本测试报告所示结果仅涉及受测试样品。
注意：检测/检验报告或证书的真实性，请通过电话(86－755) 83071443或邮箱 CN.Doccheck@sgs.com查询。

No.1, 5/F., Building D1, No. 1666, Section 2 of Chenglong Road, Economic and Technological Development Zone, Longquanyi District, Chengdu, Sichuan, China 610100　t (86–28) 8481 0338　www.sgsgroup.com.cn
中国・四川・成都市龙泉驿区经济技术开发区成龙大道二段1666号 D1栋05层1号　邮编：610100　t (86–28) 8481 0338　sgs.china@sgs.com

Member of the SGS Group (SGS SA)

编号	检测项目	单位	CAS No.	检测结果 1	定量限
307	#十三吗啉	mg/kg	24602-86-6	ND	0.01
308	肟菌酯	mg/kg	141517-21-7	ND	0.01
309	氟菌唑	mg/kg	68694-11-1	ND	0.01
310	杀铃脲	mg/kg	64628-44-0	ND	0.01
311	#氟胺磺隆	mg/kg	126535-15-7	ND	0.01
312	#抗倒酯	mg/kg	95266-40-3	ND	0.01
313	#灭菌唑	mg/kg	131983-72-7	ND	0.01
314	#烯效唑和高效烯效唑	mg/kg	83657-17-4 /83657-22-1	ND	0.01
315	#蚜灭磷（完灭硫磷）	mg/kg	2275-23-2	ND	0.01
316	#灭除威	mg/kg	2655-14-3	ND	0.01
317	苯酰菌胺（苯酰草胺）	mg/kg	156052-68-5	ND	0.01
318	#三唑酮和三唑醇之和	mg/kg	-	ND	-
319	#灭多威（灭多威和硫双威之和）	mg/kg	-	ND	-
320	克百威(克百威和 3-羟基克百威之和，以克百威表示)	mg/kg	-	ND	-
321	#氟虫腈(氟虫腈、氟虫腈砜及氟虫腈亚砜之和，以氟虫腈表示)	mg/kg	-	ND	-
322	#涕灭威(涕灭威、涕灭威亚砜及涕灭砜威之和，以涕灭威表示)	mg/kg	-	ND	-
323	#倍硫磷(倍硫磷、倍硫磷亚砜及倍硫磷砜之和，以倍硫磷表示)	mg/kg	-	ND	-
324	#乙硫甲威（乙硫甲威,乙硫甲威-砜,乙硫甲威-亚砜之和，以乙硫甲威表示）	mg/kg	-	ND	-
325	#环丙氨嗪(灭蝇胺)	mg/kg	66215-27-8	ND	0.01
326	#二硫酮砜	mg/kg	2497-06-5	ND	0.01
327	#二硫代亚砜	mg/kg	2497-07-6	ND	0.01
328	#氟甲腈	mg/kg	205650-65-3	ND	0.01
329	#噁唑菌酮	mg/kg	131807-57-3	ND	0.01
330	#咪鲜胺-脱氨基咪唑	mg/kg	139520-94-8	ND	0.01
331	#咪鲜胺-脱咪唑甲酰胺基	mg/kg	139542-32-8	ND	0.01
332	#丁氟螨酯	mg/kg	400882-07-7	ND	0.01
333	#苯嘧磺草胺	mg/kg	372137-35-4	ND	0.01
334	#氟唑环菌胺	mg/kg	874967-67-6	ND	0.01
335	#胺苯吡菌酮	mg/kg	473798-59-3	ND	0.01
336	#消螨酚	mg/kg	131-89-5	ND	0.01
337	#戊硝酚	mg/kg	4097-36-3	ND	0.01
338	#烯啶虫胺	mg/kg	120738-89-8 /150824-47-8	ND	0.01
339	#联苯肼酯	mg/kg	149877-41-8	ND	0.01
340	#吡噻菌胺	mg/kg	183675-82-3	ND	0.01

除非另有书面协议，本报告由本公司依据《 服务通用条款 》出具。
《 服务通用条款 》印刷在正本报告纸背面，或通过 https://www.sgs.com/en/Terms-and-Conditions 查询。请特别关注其中涉及责任限定，赔偿以及司法管辖的相关条款。任何报告的持有方需知悉，此报告内容仅反映SGS受限于客户指示下，且在当时所得结论。SGS仅对其客户负责，并且此报告不能免除交易各方根据交易文件所享有的权利和应履行的义务。未获得本公司书面批准，本文件不得进行复制，全文除外。任何未经授权对报告内容及形式进行的修改、伪造或扭曲都是违法行为，违法者将会被追究法律责任。除非另有声明，本测试报告所示结果仅涉及受测试样品。
注意：检测/检验报告或证书的真实性，请通过电话(86－755) 83071443或邮箱 CN.Doccheck@sgs.com 查询。

通标标准技术服务有限公司四川分公司 检验检测专用章 Inspection & Testing Services
通标标准技术服务有限公司四川分公司 农产食品实验室

No.1, 5/F., Building D1, No. 1666, Section 2 of Chenglong Road, Economic and Technological Development Zone, Longquanyi District, Chengdu, Sichuan, China 610100 t (86–28) 8481 0338 www.sgsgroup.com.cn
中国・四川・成都市龙泉驿区经济技术开发区成龙大道二段1666号 D1栋05层1号 邮编：610100 t (86–28) 8481 0338 sgs.china@sgs.com

Member of the SGS Group (SGS SA)

编号	检测项目	单位	CAS No.	检测结果 1	定量限
341	2,4-滴 & 2,4-滴钠盐	mg/kg	2702-72-9 /94-75-7	ND	0.01
342	#伊维菌素	mg/kg	70288-86-7	ND	0.01
343	#除虫菊素 I	mg/kg	121-21-1	ND	0.01
344	#除虫菊素 II	mg/kg	121-29-9	ND	0.01
345	#氟咯草酮	mg/kg	61213-25-0	ND	0.01
346	#扑草净	mg/kg	7287-19-6	ND	0.01
347	多杀霉素	mg/kg	168316-95-8	ND	0.01
348	#胺苯磺隆	mg/kg	97780-06-8	ND	0.01
349	#唑嘧菌胺	mg/kg	865318-97-4	ND	0.01
350	#噻草酮	mg/kg	101205-02-1	ND	0.01
351	#唑胺菌酯	mg/kg	915410-70-7	ND	0.01
352	#丁吡吗啉	mg/kg	868390-90-3	ND	0.01
353	#啶菌噁唑	mg/kg	847749-37-5	ND	0.01
354	#乙基多杀菌素 J	mg/kg	187166-40-1	ND	0.01
355	#咪鲜胺(咪鲜胺、咪鲜胺-脱氨基咪唑及咪鲜胺-脱咪唑甲酰胺基之和，以咪鲜胺表示)	mg/kg	-	ND	-
356	#胺鲜酯	mg/kg	10369-83-2	ND	0.01
357	#苯菌灵和多菌灵	mg/kg	-	ND	0.01
358	#苯并烯氟菌唑	mg/kg	1072957-71-1	ND	0.01
359	#烯草酮亚砜	mg/kg	111031-14-2	ND	0.01
360	#烯草酮砜	mg/kg	111031-17-5	ND	0.01
361	#螺虫乙酯-酮基-羟基	mg/kg	1172134-11-0	ND	0.01
362	#螺虫乙酯-烯醇-葡萄糖苷	mg/kg	1172614-86-6	ND	0.01
363	#螺虫乙酯-单-羟基	mg/kg	1172134-12-1	ND	0.01
364	#甲磺草胺	mg/kg	122836-35-5	ND	0.01
365	#丰索磷砜	mg/kg	14255-72-2	ND	0.01
366	#螺虫乙酯-烯醇	mg/kg	203312-38-3	ND	0.01
367	#甲硫威砜	mg/kg	2179-25-1	ND	0.01
368	#甲硫威亚砜	mg/kg	2635-10-1	ND	0.01
369	#烯肟菌酯	mg/kg	238410-11-2	ND	0.01
370	#N-二甲基甲酰基甲硫基甲醛肟	mg/kg	30558-43-1	ND	0.01
371	#烯肟菌胺	mg/kg	366815-39-6	ND	0.01
372	#阿苯达唑	mg/kg	54965-21-8	ND	0.01
373	#丙嗪嘧磺隆	mg/kg	570415-88-2	ND	0.01
374	#丰索磷-氧化砜	mg/kg	6132-17-8	ND	0.01
375	#氟节胺	mg/kg	62924-70-3	ND	0.01

除非另有书面协议，本报告由本公司依据《 服务通用条款 》出具。
《 服务通用条款 》印刷在正本报告纸背面，或通过 https://www.sgs.com/en/Terms-and-Conditions 查询。请特别关注其中涉及责任限定，赔偿以及司法管辖的相关条款。任何报告的持有方需知悉，此报告内容仅反映SGS受限于客户指示下，且在当时所得结论。SGS仅对其客户负责，并且此报告不能免除交易各方根据交易文件所享有的权利和应履行的义务。未获得本公司书面批准，本文件不得进行复制，全文除外。任何未经授权对报告内容及形式进行的修改、伪造或扭曲都是违法行为，违法者将会被追究法律责任。除非另有声明，本检测报告所示结果仅涉及受测试样品。
注意：检测/检验报告或证书的真实性，请通过电话 (86－755) 83071443 或邮箱 CN.Doccheck@sgs.com 查询。

通标标准技术服务有限公司四川分公司 检验检测专用章 Inspection & Testing Services 农产食品实验室

No.1, 5/F., Building D1, No. 1666, Section 2 of Chenglong Road, Economic and Technological Development Zone, Longquanyi District, Chengdu, Sichuan, China 610100 t (86–28) 8481 0338 www.sgsgroup.com.cn
中国・四川・成都市龙泉驿区经济技术开发区成龙大道二段1666号 D1栋05层1号 邮编：610100 t (86–28) 8481 0338 sgs.china@sgs.com

Member of the SGS Group (SGS SA)

编号	检测项目	单位	CAS No.	检测结果 1	定量限
376	#丰索磷-氧	mg/kg	6552-21-2	ND	0.01
377	#丁香菌酯	mg/kg	850881-70-8	ND	0.01
378	#嗪吡嘧磺隆	mg/kg	868680-84-6	ND	0.01
379	#吡唑萘菌胺	mg/kg	881685-58-1	ND	0.01
380	#螺虫乙酯(螺虫乙酯、螺虫乙酯-烯醇、螺虫乙酯-烯醇-葡萄糖苷、螺虫乙酯-酮基-羟基及螺虫乙酯-单-羟基之和，以螺虫乙酯表示)	mg/kg	-	ND	0.01
381	#甲硫威(甲硫威、甲硫威亚砜及甲硫威砜之和，以甲硫威表示)	mg/kg	-	ND	0.01
382	#亚砜磷(亚砜磷、甲基内吸磷及砜吸磷之和，以亚砜磷表示)	mg/kg	-	ND	-
383	#烯草酮(烯草酮、烯草酮亚砜及烯草酮砜之和，以烯草酮表示)	mg/kg	-	ND	-
384	#丰索磷(丰索磷、氧丰索磷、氧丰索磷砜及丰索磷砜，以丰索磷表示)	mg/kg	-	ND	-
385	#除虫菊素(除虫菊素 I 和除虫菊素 II 之和)	mg/kg	-	ND	-

检测方法：　EN 12393-2013

编号	检测项目	单位	CAS No.	检测结果 1	定量限
386	#2,4-滴丁酯	mg/kg	94-80-4	ND	0.01
387	邻苯基苯酚（2-苯基苯酚）（OPP）	mg/kg	90-43-7	ND	0.01
388	#氟丙菊酯	mg/kg	101007-06-1	ND	0.01
389	甲草胺	mg/kg	15972-60-8	ND	0.01
390	艾氏剂	mg/kg	309-00-2	ND	0.01
391	莠灭净	mg/kg	834-12-8	ND	0.02
392	#蒽醌	mg/kg	84-65-1	ND	0.01
393	益棉磷（乙基保棉磷）（乙基谷硫磷）	mg/kg	2642-71-9	ND	0.02
394	噁虫威	mg/kg	22781-23-3	ND	0.01
395	乙丁氟灵	mg/kg	1861-40-1	ND	0.01
396	#呋草黄（呋草磺）	mg/kg	68505-69-1	ND	0.01
397	联苯菊酯	mg/kg	82657-04-3	ND	0.01
398	溴硫磷	mg/kg	2104-96-3	ND	0.01
399	乙基溴硫磷	mg/kg	4824-78-6	ND	0.01
400	溴螨酯	mg/kg	18181-80-1	ND	0.01
401	#丁草胺	mg/kg	23184-66-9	ND	0.01
402	#抑草磷	mg/kg	36335-67-8	ND	0.01
403	仲丁灵	mg/kg	33629-47-9	ND	0.01

通标标准技术服务有限公司四川分公司
检验检测专用章
Inspection & Testing Services
农产品食品实验室

除非另有书面协议，本报告由本公司依据《 服务通用条款 》出具。
《 服务通用条款 》印刷在正本报告纸背面，或通过 https://www.sgs.com/en/Terms-and-Conditions 查询。请特别关注其中涉及责任限定，赔偿以及司法管辖的相关条款。任何报告的持有方需知悉，此报告内容仅反映SGS受限于客户指示下，且在当时所得结论。SGS仅对其客户负责，并且此报告不能免除交易各方根据交易文件所享有的权利和应履行的义务。未获得本公司书面批准，本文件不得进行复制，全文除外。任何未经授权对报告内容及形式进行的修改、伪造或扭曲都是违法行为，违法者将会被追究法律责任。除非另有声明，本测试报告所示结果仅涉及受测试样品。
注意：检测/检验报告或证书的真实性，请通过电话(86－755) 83071443或邮箱 CN.Doccheck@sgs.com查询。

No.1, 5/F., Building D1, No. 1666, Section 2 of Chenglong Road, Economic and Technological Development Zone, Longquanyi District, Chengdu, Sichuan, China 610100　t (86–28) 8481 0338　www.sgsgroup.com.cn
中国・四川・成都市龙泉驿区经济技术开发区成龙大道二段1666号 D1栋05层1号　邮编：610100　t (86–28) 8481 0338　sgs.china@sgs.com

Member of the SGS Group (SGS SA)

编号	检测项目	单位	CAS No.	检测结果 1	定量限
404	硫线磷	mg/kg	95465-99-9	ND	0.02
405	三硫磷	mg/kg	786-19-6	ND	0.01
406	杀螨醚（氯杀螨）	mg/kg	103-17-3	ND	0.01
407	虫螨腈（溴虫腈）	mg/kg	122453-73-0	ND	0.01
408	杀螨酯	mg/kg	80-33-1	ND	0.01
409	毒虫畏（杀螟威）	mg/kg	470-90-6	ND	0.01
410	乙酯杀螨醇（克氯苯）	mg/kg	510-15-6	ND	0.01
411	氯苯甲醚	mg/kg	2675-77-6	ND	0.02
412	氯苯胺灵	mg/kg	101-21-3	ND	0.01
413	毒死蜱	mg/kg	2921-88-2	ND	0.01
414	甲基毒死蜱	mg/kg	5598-13-0	ND	0.01
415	氯酞酸甲酯	mg/kg	1861-32-1	ND	0.01
416	乙菌利（克氯得）	mg/kg	84332-86-5	ND	0.01
417	顺式-氯丹（α-氯丹）	mg/kg	5103-71-9	ND	0.01
418	炔草酯（炔草酸）	mg/kg	105512-06-9	ND	0.01
419	异噁草松（异噁草酮）	mg/kg	81777-89-1	ND	0.01
420	#苯腈磷（苯腈硫磷）	mg/kg	13067-93-1	ND	0.01
421	杀螟腈（杀虫腈）	mg/kg	2636-26-2	ND	0.01
422	#氰氟草酯	mg/kg	122008-85-9	ND	0.01
423	环丙唑醇	mg/kg	94361-06-5	ND	0.01
424	1,2,4-三丁基三硫磷酸酯（脱叶磷）	mg/kg	78-48-8	ND	0.01
425	溴氰菊酯	mg/kg	52918-63-5	ND	0.01
426	二嗪磷（二嗪农）	mg/kg	333-41-5	ND	0.01
427	除线磷	mg/kg	97-17-6	ND	0.01
428	#苯氟磺胺（抑菌灵）	mg/kg	1085-98-9	ND	0.01
429	#烯丙酰草胺（二氯丙烯胺）	mg/kg	37764-25-3	ND	0.01
430	敌敌畏	mg/kg	62-73-7	ND	0.01
431	#苄氯三唑醇	mg/kg	75736-33-3	ND	0.01
432	禾草灵	mg/kg	51338-27-3	ND	0.01
433	氯硝胺	mg/kg	99-30-9	ND	0.01
434	狄氏剂	mg/kg	60-57-1	ND	0.01
435	#噻节因	mg/kg	55290-64-7	ND	0.01
436	#甲基毒虫畏	mg/kg	2274-67-1	ND	0.01
437	#蔬果磷	mg/kg	3811-49-2	ND	0.01
438	二苯胺	mg/kg	122-39-4	ND	0.01
439	#氟硫草定	mg/kg	97886-45-8	ND	0.01
440	硫丹硫酸酯	mg/kg	1031-07-8	ND	0.01
441	异狄氏剂	mg/kg	72-20-8	ND	0.01
442	苯硫磷	mg/kg	2104-64-5	ND	0.01
443	#乙丁烯氟灵	mg/kg	55283-68-6	ND	0.01

除非另有书面协议，本报告由本公司依据《服务通用条款》出具。
《服务通用条款》印刷在正本报告纸背面，或通过 https://www.sgs.com/en/Terms-and-Conditions 查询。请特别关注其中涉及责任限定，赔偿以及司法管辖的相关条款。任何报告的持有方需知悉，此报告内容仅反映SGS受限于客户指示下，且在当时所得结论。SGS仅对其客户负责，并且此报告不能免除交易各方根据交易文件所享有的权利和应履行的义务。未获得本公司书面批准，本文件不得进行复制，全文除外。任何未经授权对报告内容及形式进行的修改、伪造或扭曲都是违法行为，违法者将会被追究法律责任。除非另有声明，本测试报告所示结果仅涉及受测试样品。
注意：检测/检验报告或证书的真实性，请通过电话(86-755) 83071443或邮箱 CN.Doccheck@sgs.com 查询。

通标标准技术服务有限公司四川分公司 检验检测专用章 Inspection & Testing Services 农产食品实验室

No.1, 5/F., Building D1, No. 1666, Section 2 of Chenglong Road, Economic and Technological Development Zone, Longquanyi District, Chengdu, Sichuan, China 610100 t (86–28) 8481 0338 www.sgsgroup.com.cn
中国・四川・成都市龙泉驿区经济技术开发区成龙大道二段1666号 D1栋05层1号 邮编：610100 t (86–28) 8481 0338 sgs.china@sgs.com

Member of the SGS Group (SGS SA)

编号	检测项目	单位	CAS No.	检测结果 1	定量限
444	乙硫磷	mg/kg	563-12-2	ND	0.01
445	醚菊酯	mg/kg	80844-07-1	ND	0.01
446	乙氧呋草黄	mg/kg	26225-79-6	ND	0.01
447	灭线磷	mg/kg	13194-48-4	ND	0.01
448	乙嘧硫磷	mg/kg	38260-54-7	ND	0.01
449	皮蝇磷	mg/kg	299-84-3	ND	0.01
450	#敌草腈	mg/kg	1194-65-6	ND	0.01
451	杀螟硫磷（杀螟松）	mg/kg	122-14-5	ND	0.01
452	甲氰菊酯	mg/kg	39515-41-8 /64257-84-7	ND	0.01
453	丰索磷	mg/kg	115-90-2	ND	0.01
454	氰戊菊酯和 S-氰戊菊酯	mg/kg	51630-58-1 /66230-04-4	ND	0.01
455	嘧螨酯	mg/kg	229977-93-9	ND	0.01
456	氟氰戊菊酯	mg/kg	70124-77-5	ND	0.01
457	氟哇唑（氟硅唑）	mg/kg	85509-19-9	ND	0.01
458	#氟酰胺	mg/kg	66332-96-5	ND	0.01
459	地虫硫磷	mg/kg	944-22-9	ND	0.01
460	#安硫磷（安果磷）	mg/kg	2540-82-1	ND	0.01
461	呋霜灵	mg/kg	57646-30-7	ND	0.01
462	#苄螨醚（苄满醚）	mg/kg	111872-58-3	ND	0.01
463	七氯	mg/kg	76-44-8	ND	0.01
464	环氧七氯（B 型，顺式）	mg/kg	1024-57-3 /28044-83-9	ND	0.01
465	庚烯磷	mg/kg	23560-59-0	ND	0.01
466	六氯苯	mg/kg	118-74-1	ND	0.01
467	异艾氏剂	mg/kg	465-73-6	ND	0.01
468	异柳磷	mg/kg	25311-71-1	ND	0.01
469	甲基异柳磷	mg/kg	99675-03-3	ND	0.01
470	γ-六六六（林丹）	mg/kg	58-89-9	ND	0.01
471	#灭蚜磷	mg/kg	2595-54-2	ND	0.01
472	虫螨畏	mg/kg	62610-77-9	ND	0.01
473	甲氧氯（甲氧滴滴涕）	mg/kg	72-43-5	ND	0.01
474	#五氯苯甲硫醚（甲基五氯苯硫醚）	mg/kg	1825-19-0	ND	0.01
475	嗪草酮	mg/kg	21087-64-9	ND	0.01
476	速灭磷	mg/kg	7786-34-7	ND	0.01
477	灭蚁灵	mg/kg	2385-85-5	ND	0.01
478	敌草胺	mg/kg	15299-99-7	ND	0.01
479	除草醚	mg/kg	1836-75-5	ND	0.01
480	酞菌酯	mg/kg	10552-74-6	ND	0.01
481	o,p'-滴滴涕	mg/kg	789-02-6	ND	0.01

通标标准技术服务有限公司四川分公司 检验检测专用章 Inspection & Testing Services SGS-CSTC Standards Technical Services Co., Ltd. Sichuan Branch

通标标准技术服务有限公司四川分公司 农产食品实验室

除非另有书面协议，本报告由本公司依据《 服务通用条款 》出具。
《 服务通用条款 》印刷在正本报告纸背面，或通过 https://www.sgs.com/en/Terms-and-Conditions 查询。请特别关注其中涉及责任限定，赔偿以及司法管辖的相关条款。任何报告的持有方需知悉，此报告内容仅反映SGS受限于客户指示下，且在当时所得结论。SGS仅对其客户负责，并且此报告不能免除交易各方根据交易文件所享有的权利和应履行的义务。未获得本公司书面批准，本文件不得进行复制，全文除外。任何未经授权对报告内容及形式进行的修改、伪造或扭曲都是违法行为，违法者将会被追究法律责任。除非另有声明，本测试报告所示结果仅涉及受测试样品。
注意：检测/检验报告或证书的真实性，请通过电话（86－755）83071443或邮箱 CN.Doccheck@sgs.com 查询。

No.1, 5/F., Building D1, No. 1666, Section 2 of Chenglong Road, Economic and Technological Development Zone, Longquanyi District, Chengdu, Sichuan, China 610100　t (86–28) 8481 0338　www.sgsgroup.com.cn
中国・四川・成都市龙泉驿区经济技术开发区成龙大道二段1666号 D1栋05层1号　邮编：610100　t (86–28) 8481 0338　sgs.china@sgs.com

Member of the SGS Group (SGS SA)

编号	检测项目	单位	CAS No.	检测结果 1	定量限
482	p,p'-滴滴滴	mg/kg	72-54-8	ND	0.01
483	p,p'-滴滴伊	mg/kg	72-55-9	ND	0.01
484	p,p'-滴滴涕	mg/kg	50-29-3	ND	0.01
485	多效唑	mg/kg	76738-62-0	ND	0.01
486	乙基对硫磷（对硫磷）	mg/kg	56-38-2	ND	0.01
487	甲基对硫磷	mg/kg	298-00-0	ND	0.01
488	戊菌唑	mg/kg	66246-88-6	ND	0.01
489	二甲戊灵（二甲戊乐灵）	mg/kg	40487-42-1	ND	0.01
490	五氯苯胺	mg/kg	527-20-8	ND	0.01
491	五氯甲氧基苯（五氯茴香醚）	mg/kg	1825-21-4	ND	0.01
492	#戊基恶唑酮	mg/kg	110956-75-7	ND	0.01
493	氯菊酯	mg/kg	52645-53-1	ND	0.01
494	#苯醚菊酯	mg/kg	26002-80-2	ND	0.01
495	稻丰散	mg/kg	2597-03-7	ND	0.01
496	甲拌磷	mg/kg	298-02-2	ND	0.01
497	磷胺	mg/kg	13171-21-6	ND	0.02
498	#四氯苯酞	mg/kg	27355-22-2	ND	0.01
499	增效醚	mg/kg	51-03-6	ND	0.01
500	#哌草磷	mg/kg	24151-93-7	ND	0.01
501	嘧啶磷（乙基嘧啶磷）	mg/kg	23505-41-1	ND	0.01
502	甲基虫螨磷（甲基嘧啶磷）	mg/kg	29232-93-7	ND	0.01
503	腐霉利	mg/kg	32809-16-8	ND	0.01
504	丙溴磷（溴氯磷）	mg/kg	41198-08-7	ND	0.01
505	#丙虫磷	mg/kg	7292-16-2	ND	0.01
506	胺丙畏（巴胺磷）（烯虫磷）	mg/kg	31218-83-4	ND	0.01
507	苯胺灵	mg/kg	122-42-9	ND	0.01
508	丙环唑	mg/kg	60207-90-1	ND	0.01
509	炔苯酰草胺（拿草特）	mg/kg	23950-58-5	ND	0.01
510	丙硫磷（普硫松）	mg/kg	34643-46-4	ND	0.01
511	#嘧草醚	mg/kg	136191-64-5	ND	0.01
512	喹硫磷	mg/kg	13593-03-8	ND	0.01
513	五氯硝基苯	mg/kg	82-68-8	ND	0.01
514	八氯二丙醚	mg/kg	127-90-2	ND	0.01
515	#硅醚菊酯	mg/kg	105024-66-6	ND	0.01
516	治螟磷	mg/kg	3689-24-5	ND	0.01
517	硫丙磷	mg/kg	35400-43-2	ND	0.01
518	四氯硝基苯	mg/kg	117-18-0	ND	0.01
519	七氟菊酯	mg/kg	79538-32-2	ND	0.01
520	特丁硫磷	mg/kg	13071-79-9	ND	0.01
521	杀虫畏	mg/kg	22248-79-9	ND	0.02

除非另有书面协议，本报告由本公司依据《服务通用条款》出具。
《服务通用条款》印刷在正本报告纸背面，或通过 https://www.sgs.com/en/Terms-and-Conditions 查询。请特别关注其中涉及责任限定，赔偿以及司法管辖的相关条款。任何报告的持有方需知悉，此报告内容仅反映SGS受限于客户指示下，且在当时所得结论。SGS仅对其客户负责，并且此报告不能免除交易各方根据交易文件所享有的权利和应履行的义务。未获得本公司书面批准，本文件不得进行复制，全文除外。任何未经授权对报告内容及形式进行的修改、伪造或扭曲都是违法行为，违法者将会被追究法律责任。除非另有声明，本测试报告所示结果仅涉及受测试样品。
注意：检测/检验报告或证书的真实性，请通过电话 (86–755) 83071443 或邮箱 CN.Doccheck@sgs.com 查询。

通标标准技术服务有限公司四川分公司 检验检测专用章 Inspection & Testing Services 农产食品实验室

No.1, 5/F., Building D1, No. 1666, Section 2 of Chenglong Road, Economic and Technological Development Zone, Longquanyi District, Chengdu, Sichuan, China 610100 t (86–28) 8481 0338 www.sgsgroup.com.cn
中国・四川・成都市龙泉驿区经济技术开发区成龙大道二段1666号 D1栋05层1号 邮编：610100 t (86–28) 8481 0338 sgs.china@sgs.com

Member of the SGS Group (SGS SA)

编号	检测项目	单位	CAS No.	检测结果 1	定量限
522	四氟醚唑（氟醚唑）	mg/kg	112281-77-3	ND	0.01
523	三氯杀螨砜	mg/kg	116-29-0	ND	0.01
524	胺菊酯	mg/kg	7696-12-0	ND	0.01
525	甲基立枯磷	mg/kg	57018-04-9	ND	0.01
526	甲苯氟磺胺	mg/kg	731-27-1	ND	0.02
527	六六六总量（α-六六六,β-六六六,γ-六六六,δ-六六六）	mg/kg	319-84-6 /319-85-7 /319-86-8 /58-89-9	ND	-
528	（顺式-氯丹,反式-氯丹）总量	mg/kg	-	ND	-
529	滴滴涕总量（o,p'-滴滴涕,p,p'-滴滴滴,p,p'-滴滴伊,p,p'-滴滴涕）	mg/kg	-	ND	-
530	硫丹总量 （α-硫丹,β-硫丹和硫丹硫酸酯之和）	mg/kg	1031-07-8 /33213-65-9 /959-98-8	ND	-
531	七氯和环氧七氯总量	mg/kg	-	ND	-
532	#氟氯氰菊酯和高效氟氯氰菊酯	mg/kg	1820573-27-0 /68359-37-5	ND	0.01
533	#氯氟氰菊酯和高效氯氟氰菊酯	mg/kg	68085-85-8 /91465-08-6	ND	0.01
534	反式氯丹	mg/kg	5103-74-2	ND	0.01
535	野麦畏	mg/kg	2303-17-5	ND	0.01
536	氟乐灵	mg/kg	1582-09-8	ND	0.01
537	乙烯菌核利	mg/kg	50471-44-8	ND	0.01
538	α-六六六	mg/kg	319-84-6	ND	0.01
539	α-硫丹	mg/kg	959-98-8	ND	0.01
540	β-六六六	mg/kg	319-85-7	ND	0.01
541	β-硫丹	mg/kg	33213-65-9	ND	0.01
542	δ-六六六	mg/kg	319-86-8	ND	0.01
543	氟胺氰菊酯	mg/kg	102851-06-9	ND	0.01
544	苯线磷	mg/kg	22224-92-6	ND	0.05
545	氯氰菊酯	mg/kg	52315-07-8	ND	0.01
546	#二甲吩草胺	mg/kg	87674-68-8	ND	0.01
547	#水胺硫磷	mg/kg	24353-61-5	ND	0.01
548	p,p'-三氯杀螨醇	mg/kg	115-32-2	ND	0.01
549	#（甲拌磷,甲拌磷砜,甲拌磷亚砜）总量	mg/kg	-	ND	-
550	#氯唑磷	mg/kg	42509-80-8	ND	0.01
551	#甲基乙拌磷（二甲基硫吸磷）	mg/kg	640-15-3	ND	0.01
552	#乙拌磷	mg/kg	298-04-4	ND	0.01
553	#氯苯嘧啶醇	mg/kg	60168-88-9	ND	0.01
554	#乙草胺	mg/kg	34256-82-1	ND	0.01

通标标准技术服务有限公司四川分公司 检验检测专用章 Inspection & Testing Services
通标标准技术服务有限公司四川分公司
农产食品实验室

除非另有书面协议，本报告由本公司依据《 服务通用条款 》出具。
《 服务通用条款 》印刷在正本报告纸背面，或通过 https://www.sgs.com/en/Terms-and-Conditions 查询。请特别关注其中涉及责任限定，赔偿以及司法管辖的相关条款。任何报告的持有方需知悉，此报告内容仅反映SGS受限于客户指示下，且在当时所得结论。SGS仅对其客户负责，并且此报告不能免除交易各方根据交易文件所享有的权利和应履行的义务。未获得本公司书面批准，本文件不得进行复制，全文除外。任何未经授权对报告内容及形式进行的修改、伪造或扭曲都是违法行为，违法者将会被追究法律责任。除非另有声明，本测试报告所示结果仅涉及受测试样品。
注意：检测/检验报告或证书的真实性，请通过电话(86－755) 83071443或邮箱 CN.Doccheck@sgs.com查询。

No.1, 5/F., Building D1, No. 1666, Section 2 of Chenglong Road, Economic and Technological Development Zone, Longquanyi District, Chengdu, Sichuan, China 610100 t (86–28) 8481 0338 www.sgsgroup.com.cn
中国・四川・成都市龙泉驿区经济技术开发区成龙大道二段1666号 D1栋05层1号 邮编：610100 t (86–28) 8481 0338 sgs.china@sgs.com

Member of the SGS Group (SGS SA)

编号	检测项目	单位	CAS No.	检测结果 1	定量限
555	#敌瘟磷（敌瘟散）	mg/kg	17109-49-8	ND	0.01
556	#噻唑硫磷（噻唑磷）	mg/kg	98886-44-3	ND	0.01
557	#敌稗	mg/kg	709-98-8	ND	0.01
558	#蝇毒磷	mg/kg	56-72-4	ND	0.01
559	#氟喹唑	mg/kg	136426-54-5	ND	0.01
560	#苯硫威	mg/kg	62850-32-2	ND	0.01
561	#吡草醚（霸草灵）	mg/kg	129630-19-9	ND	0.01
562	#吡唑硫磷	mg/kg	77458-01-6	ND	0.01
563	#丙草胺	mg/kg	51218-49-6	ND	0.01
564	#丙烯菊酯	mg/kg	584-79-2	ND	0.01
565	#丁草敌（丁草特）	mg/kg	2008-41-5	ND	0.01
566	#丁基嘧啶磷	mg/kg	96182-53-5	ND	0.01
567	#恶唑磷	mg/kg	18854-01-8	ND	0.01
568	#氟啶草酮	mg/kg	59756-60-4	ND	0.01
569	#胺氟草酯（氟烯草酸）	mg/kg	87546-18-7	ND	0.01
570	#环嗪酮	mg/kg	51235-04-2	ND	0.01
571	#甲基异内吸磷	mg/kg	919-86-8	ND	0.01
572	#解草酯（解毒喹）	mg/kg	99607-70-2	ND	0.01
573	#灭锈胺	mg/kg	55814-41-0	ND	0.01
574	#扑灭津	mg/kg	139-40-2	ND	0.01
575	#双苯酰草胺	mg/kg	957-51-7	ND	0.01
576	#特草定	mg/kg	5902-51-2	ND	0.01
577	#特丁净（特丁草净）	mg/kg	886-50-0	ND	0.01
578	#土菌灵	mg/kg	2593-15-9	ND	0.01
579	#吲熟酯	mg/kg	27512-72-7	ND	0.01
580	#特丁津	mg/kg	5915-41-3	ND	0.01
581	#甲拌磷亚砜	mg/kg	2588-03-6	ND	0.01
582	#甲拌磷砜	mg/kg	2588-04-7	ND	0.01
583	#丙酯杀螨醇	mg/kg	5836-10-2	ND	0.01
584	#燕麦灵	mg/kg	101-27-9	ND	0.05
585	#氟除草醚	mg/kg	13738-63-1	ND	0.01
586	#三氟硝草醚	mg/kg	15457-05-3	ND	0.01
587	#绿谷隆	mg/kg	1746-81-2	ND	0.01
588	#草枯醚	mg/kg	1836-77-7	ND	0.01
589	#百菌清	mg/kg	1897-45-6	ND	0.01
590	#螺甲螨酯	mg/kg	283594-90-1	ND	0.01
591	#烯虫酯	mg/kg	40596-69-8	ND	0.01
592	#甲羧除草醚	mg/kg	42576-02-3	ND	0.01
593	#野燕枯（硫酸二甲酯）	mg/kg	43222-48-6	ND	0.01
594	#灭菌磷	mg/kg	5131-24-8	ND	0.01

除非另有书面协议，本报告由本公司依据《 服务通用条款 》出具。
《 服务通用条款 》印刷在正本报告纸背面，或通过 https://www.sgs.com/en/Terms-and-Conditions 查询。请特别关注其中涉及责任限定，赔偿以及司法管辖的相关条款。任何报告的持有方需知悉，此报告内容仅反映SGS受限于客户指示下，且在当时所得结论。SGS仅对其客户负责，并且此报告不能免除交易各方根据交易文件所享有的权利和应履行的义务。未获得本公司书面批准，本文件不得进行复制，全文除外。任何未经授权对报告内容及形式进行的修改、伪造或扭曲都是违法行为，违法者将会被追究法律责任。除非另有声明，本测试报告所示结果仅涉及受测试样品。
注意：检测/检验报告或证书的真实性，请通过电话(86－755) 83071443或邮箱 CN.Doccheck@sgs.com 查询。

No.1, 5/F., Building D1, No. 1666, Section 2 of Chenglong Road, Economic and Technological Development Zone, Longquanyi District, Chengdu, Sichuan, China 610100 t (86–28) 8481 0338 www.sgsgroup.com.cn
中国・四川・成都市龙泉驿区经济技术开发区成龙大道二段1666号 D1栋05层1号 邮编：610100 t (86–28) 8481 0338 sgs.china@sgs.com

Member of the SGS Group (SGS SA)

检测报告

CDF25-0005759-01　　发布日期：2025-10-22

编号	检测项目	单位	CAS No.	检测结果 1	定量限
595	#氯氰菊酯&Zeta-氯氰菊酯	mg/kg	-	ND	0.01
596	#苯草醚	mg/kg	74070-46-5	ND	0.01
597	#巴毒磷	mg/kg	7700-17-6	ND	0.01
598	#乙羧氟草醚	mg/kg	77501-90-7	ND	0.01
599	#敌噁磷（敌杀磷）	mg/kg	78-34-2	ND	0.01
600	#联苯	mg/kg	92-52-4	ND	0.01
601	#二丙烯草胺	mg/kg	93-71-0	ND	0.01
602	#格螨酯	mg/kg	97-16-5	ND	0.01

备注:
- ND = 未检出
- #检测项目不在本实验室的 CNAS 认可范围内。

注意事项：
除非另有说明，本检测结果仅与被检测物品有关。未经检验机构书面同意，委托人不得擅自使用检测结果进行不当宣传。本检测报告仅作为科研、教学或内部质量控制之用。未经检验机构批准，不得复制（全文复制除外）报告。

结束

除非另有书面协议，本报告由本公司依据《 服务通用条款 》出具。
《 服务通用条款 》印刷在正本报告纸背面，或通过 https://www.sgs.com/en/Terms-and-Conditions 查询。请特别关注其中涉及责任限定，赔偿以及司法管辖的相关条款。任何报告的持有方需知悉，此报告内容仅反映SGS受限于客户指示下，且在当时所得结论。SGS仅对其客户负责，并且此报告不能免除交易各方根据交易文件所享有的权利和应履行的义务。未获得本公司书面批准，本文件不得进行复制，全文除外。任何未经授权对报告内容及形式进行的修改、伪造或扭曲都是违法行为，违法者将会被追究法律责任。除非另有声明，本测试报告所示结果仅涉及受测试样品。
注意：检测/检验报告或证书的真实性，请通过电话 (86 –755) 83071443 或邮箱 CN.Doccheck@sgs.com 查询。

No.1, 5/F., Building D1, No. 1666, Section 2 of Chenglong Road, Economic and Technological Development Zone, Longquanyi District, Chengdu, Sichuan, China 610100　t (86–28) 8481 0338　www.sgsgroup.com.cn
中国・四川・成都市龙泉驿区经济技术开发区成龙大道二段1666号 D1栋05层1号　邮编: 610100　t (86–28) 8481 0338　sgs.china@sgs.com

Member of the SGS Group (SGS SA)
