# Supplementary material for: Monoglyceride-structured rubber seed oil oleogels as a partial cocoa butter replacement in chocolate: physical properties, oxidative stability and applications
Source: Food Chem X. 2026 Oct 11;39:104594. doi: 10.1016/j.fochx.2026.104594 (PMC13683454; doi:10.1016/j.fochx.2026.104594)
Supplement: Supplementary file 5 [file mmc5.pdf]

检测报告

CDF25-0001880-01　　　　**发布日期：**2025-05-06

客户名称：　西双版纳华坤生物科技有限责任公司
客户地址：　景洪市嘎洒镇曼迈村委会曼景栋村

样品名称：　橡胶树种子油
样品批号：　/
生产日期：　20250423
生产商：　西双版纳华坤生物科技有限责任公司

以上样品及信息由客户提供及确认, SGS 不承担证实客户提供信息的准确性、适当性和(或)完整性责任。

样品接收日期：　2025-04-27
检测周期：　2025-04-27 ~ 2025-05-06
检测要求：　根据客户要求检测
检测方法：　请参见下一页。
检测结果：　请参见下一页。

SGS 授权签字人

扫码查看在线报告
CDF25-0001880-01
报告验真请访问:
check.sgsonline.com.cn

除非另有书面协议，本报告由本公司依据《 服务通用条款 》出具。
《 服务通用条款 》印刷在正本报告纸背面，或通过 https://www.sgs.com/en/Terms-and-Conditions 查询。请特别关注其中涉及责任限定，赔偿以及司法管辖的相关条款。任何报告的持有方需知悉，此报告内容仅反映SGS受限于客户指示下，且在当时所得结论。SGS仅对其客户负责，并且此报告不能免除交易各方根据交易文件所享有的权利和应履行的义务。未获得本公司书面批准，本文件不得进行复制，全文除外。任何未经授权对报告内容及形式进行的修改、伪造或扭曲都是违法行为，违法者将会被追究法律责任。除非另有声明，本测试报告所示结果仅涉及受测试样品。
注意：检测/检验报告或证书的真实性，请通过电话 (86－755) 83071443 或邮箱 CN.Doccheck@sgs.com 查询。

No.1, 5/F., Building D1, No. 1666, Section 2 of Chenglong Road, Economic and Technological Development Zone, Longquanyi District, Chengdu, Sichuan, China 610100　t (86–28) 8481 0338　www.sgsgroup.com.cn
中国・四川・成都市龙泉驿区经济技术开发区成龙大道二段1666号 D1栋05层1号　邮编：610100　t (86–28) 8481 0338　sgs.china@sgs.com

Member of the SGS Group (SGS SA)

检测报告

CDF25-0001880-01 发布日期：2025-05-06

检测样品描述：

样品编号	SGS 样品 ID	样品描述
1	CDF25-0001880-0001	瓶装样品 1 瓶

检测结果：

农药残留

检测方法： BS EN 15662:2018
EN 12393-2013

检测结果：

编号	检测项目	单位	CAS No.	检测结果<br>1	定量限
1	#联苯	mg/kg	92-52-4	ND	0.01
2	#混杀威（3,4,5-混杀威）	mg/kg	2686-99-9	ND	0.01
3	阿维菌素	mg/kg	71751-41-2	ND	0.01
4	乙酰甲胺磷	mg/kg	30560-19-1	ND	0.01
5	啶虫脒	mg/kg	135410-20-7	ND	0.01
6	#活化酯/苯并噻二唑	mg/kg	135158-54-2	ND	0.01
7	涕灭威	mg/kg	116-06-3	ND	0.01
8	#砜灭威（涕灭威砜）	mg/kg	1646-88-4	ND	0.01
9	#涕灭威亚砜	mg/kg	1646-87-3	ND	0.01
10	#酰嘧磺隆	mg/kg	120923-37-7	ND	0.01
11	#吲唑磺菌胺	mg/kg	348635-87-0	ND	0.05
12	#莎稗磷	mg/kg	64249-01-0	ND	0.01
13	#杀螨特	mg/kg	140-57-8	ND	0.01
14	#莠去津	mg/kg	1912-24-9	ND	0.01
15	#氧环唑	mg/kg	60207-31-0	ND	0.01
16	#甲基吡恶磷	mg/kg	35575-96-3	ND	0.01
17	谷硫磷（保棉磷）	mg/kg	86-50-0	ND	0.01
18	嘧菌酯（腈嘧菌酯）	mg/kg	131860-33-8	ND	0.01
19	苯霜灵	mg/kg	71626-11-4	ND	0.01
20	#解草嗪	mg/kg	98730-04-2	ND	0.01
21	#苄嘧磺隆	mg/kg	83055-99-6	ND	0.01
22	#地散磷	mg/kg	741-58-2	ND	0.01
23	#灭草松	mg/kg	25057-89-0	ND	0.01
24	#苯螨特	mg/kg	29104-30-1	ND	0.01
25	联苯三唑醇	mg/kg	55179-31-2	ND	0.01
26	啶酰菌胺	mg/kg	188425-85-6	ND	0.01
27	#除草定	mg/kg	314-40-9	ND	0.01
28	#溴丁酰草胺	mg/kg	74712-19-9	ND	0.01

除非另有书面协议，本报告由本公司依据《 服务通用条款 》出具。
《 服务通用条款 》印刷在正本报告纸背面，或通过 https://www.sgs.com/en/Terms-and-Conditions 查询。请特别关注其中涉及责任限定，赔偿以及司法管辖的相关条款。任何报告的持有方需知悉，此报告内容仅反映SGS受限于客户指示下，且在当时所得结论。SGS仅对其客户负责，并且此报告不能免除交易各方根据交易文件所享有的权利和应履行的义务。未获得本公司书面批准，本文件不得进行复制，全文除外。任何未经授权对报告内容及形式进行的修改、伪造或扭曲都是违法行为，违法者将会被追究法律责任。除非另有声明，本测试报告所示结果仅涉及受测试样品。
注意：检测/检验报告或证书的真实性，请通过电话 (86－755) 83071443 或邮箱 CN.Doccheck@sgs.com 查询。

No.1, 5/F., Building D1, No. 1666, Section 2 of Chenglong Road, Economic and Technological Development Zone, Longquanyi District, Chengdu, Sichuan, China 610100 t (86–28) 8481 0338 www.sgsgroup.com.cn
中国・四川・成都市龙泉驿区经济技术开发区成龙大道二段1666号 D1栋05层1号 邮编：610100 t (86–28) 8481 0338 sgs.china@sgs.com

Member of the SGS Group (SGS SA)

编号	检测项目	单位	CAS No.	检测结果 1	定量限
29	溴苯腈	mg/kg	1689-84-5	ND	0.01
30	乙嘧酚磺酸酯	mg/kg	41483-43-6	ND	0.01
31	噻嗪酮	mg/kg	69327-76-0	ND	0.01
32	#氟丙嘧草酯	mg/kg	134605-64-4	ND	0.01
33	#丁酮威	mg/kg	34681-10-2	ND	0.01
34	#丁酮砜威	mg/kg	34681-23-7	ND	0.01
35	甲萘威	mg/kg	63-25-2	ND	0.01
36	多菌灵	mg/kg	10605-21-7	ND	0.01
37	呋喃丹（虫螨威/克百威）	mg/kg	1563-66-2	ND	0.01
38	3-羟基虫螨威（3-羟基克百威）	mg/kg	16655-82-6	ND	0.01
39	萎锈灵	mg/kg	5234-68-4	ND	0.01
40	#唑草酮	mg/kg	128639-02-1	ND	0.01
41	#加普胺	mg/kg	104030-54-8	ND	0.01
42	#氯虫苯甲酰胺	mg/kg	500008-45-7	ND	0.01
43	#灭幼脲	mg/kg	57160-47-1	ND	0.01
44	#氯草灵	mg/kg	1967-16-4	ND	0.01
45	#杀虫脒	mg/kg	6164-98-3	ND	0.01
46	#氟啶脲	mg/kg	71422-67-8	ND	0.01
47	#氯草敏	mg/kg	1698-60-8	ND	0.01
48	#氯嘧磺隆	mg/kg	90982-32-4	ND	0.01
49	#绿麦隆	mg/kg	15545-48-9	ND	0.01
50	#枯草隆	mg/kg	1982-47-4	ND	0.01
51	#氯磺隆	mg/kg	64902-72-3	ND	0.01
52	#环虫酰肼	mg/kg	143807-66-3	ND	0.01
53	#吲哚酮草酯	mg/kg	142891-20-1	ND	0.01
54	#环庚草醚	mg/kg	87818-31-3	ND	0.01
55	#醚磺隆	mg/kg	94593-91-6	ND	0.01
56	#烯草酮	mg/kg	99129-21-2	ND	0.01
57	四螨嗪	mg/kg	74115-24-5	ND	0.01
58	#氯甲酰草胺	mg/kg	84496-56-0	ND	0.01
59	#调果酸（调采酸）	mg/kg	101-10-0	ND	0.01
60	#氯酯磺草胺	mg/kg	147150-35-4	ND	0.01
61	噻虫胺（可尼丁）	mg/kg	210880-92-5	ND	0.01
62	#氰草津	mg/kg	21725-46-2	ND	0.01
63	氰霜唑	mg/kg	120116-88-3	ND	0.01
64	#环丙酸酰胺	mg/kg	113136-77-9	ND	0.01
65	#环草敌（草灭特）	mg/kg	1134-23-2	ND	0.01
66	#环丙嘧磺隆	mg/kg	136849-15-5	ND	0.01
67	#环氟菌胺	mg/kg	180409-60-3	ND	0.01
68	霜脲氰	mg/kg	57966-95-7	ND	0.01

除非另有书面协议，本报告由本公司依据《服务通用条款》出具。
《服务通用条款》印刷在正本报告纸背面，或通过 https://www.sgs.com/en/Terms-and-Conditions 查询。请特别关注其中涉及责任限定，赔偿以及司法管辖的相关条款。任何报告的持有方需知悉，此报告内容仅反映SGS受限于客户指示下，且在当时所得结论。SGS仅对其客户负责，并且此报告不能免除交易各方根据交易文件所享有的权利和应履行的义务。未获得本公司书面批准，本文件不得进行复制，全文除外。任何未经授权对报告内容及形式进行的修改、伪造或扭曲都是违法行为，违法者将会被追究法律责任。除非另有声明，本测试报告所示结果仅涉及受测试样品。
注意：检测/检验报告或证书的真实性，请通过电话 (86－755) 83071443 或邮箱 CN.Doccheck@sgs.com 查询。
检验检测专用章 Inspection & Testing Services
通标标准技术服务有限公司四川分公司 农产食品实验室

No.1, 5F, Building D1, No. 1666, Section 2 of Chenglong Road, Economic and Technological Development Zone, Longquanyi District, Chengdu, Sichuan, China 610100 t (86–28) 8481 0338 www.sgsgroup.com.cn
中国・四川・成都市龙泉驿区经济技术开发区成龙大道二段1666号 D1栋05层1号 邮编：610100 t (86–28) 8481 0338 sgs.china@sgs.com

Member of the SGS Group (SGS SA)

编号	检测项目	单位	CAS No.	检测结果<br>1	定量限
69	嘧菌环胺	mg/kg	121552-61-2	ND	0.01
70	#杀草隆	mg/kg	42609-52-9	ND	0.01
71	#内吸磷	mg/kg	8065-48-3	ND	0.01
72	甲基砜内吸磷	mg/kg	17040-19-6	ND	0.01
73	#亚砜磷	mg/kg	301-12-2	ND	0.01
74	#甜菜胺（甜菜安）	mg/kg	13684-56-5	ND	0.01
75	#丁醚脲（丁嘧脲）	mg/kg	80060-09-9	ND	0.01
76	#燕麦敌（丁变敌）	mg/kg	2303-16-4	ND	0.01
77	#2,4-滴丙酸	mg/kg	120-36-5	ND	0.01
78	#双氯氰菌胺	mg/kg	139920-32-4	ND	0.01
79	#双氯磺草胺	mg/kg	145701-21-9	ND	0.01
80	百治磷	mg/kg	141-66-2	ND	0.01
81	乙霉威	mg/kg	87130-20-9	ND	0.01
82	苯醚甲环唑	mg/kg	119446-68-3	ND	0.01
83	除虫脲	mg/kg	35367-38-5	ND	0.01
84	#吡氟酰草胺	mg/kg	83164-33-4	ND	0.01
85	#哌草丹	mg/kg	61432-55-1	ND	0.01
86	#异戊乙净（阔草净）	mg/kg	22936-75-0	ND	0.01
87	#二甲嘧酚	mg/kg	5221-53-4	ND	0.01
88	乐果	mg/kg	60-51-5	ND	0.01
89	烯酰吗啉	mg/kg	110488-70-5	ND	0.01
90	烯唑醇	mg/kg	83657-24-3	ND	0.01
91	#敌螨普	mg/kg	39300-45-3	ND	0.01
92	#丁诺特呋喃（呋虫胺）	mg/kg	165252-70-0	ND	0.01
93	#特乐酚	mg/kg	1420-07-1	ND	0.01
94	敌草隆	mg/kg	330-54-1	ND	0.01
95	#甲氨基阿维菌素苯甲酸盐	mg/kg	155569-91-8	ND	0.01
96	#苯氧菌胺	mg/kg	133408-50-1	ND	0.01
97	氟环唑	mg/kg	106325-08-0<br>/133855-98-8	ND	0.01
98	#戊草丹	mg/kg	85785-20-2	ND	0.01
99	#乙硫苯威	mg/kg	29973-13-5	ND	0.01
100	#乙硫苯威砜	mg/kg	53380-23-7	ND	0.01
101	#乙硫苯威亚砜	mg/kg	53380-22-6	ND	0.01
102	#乙虫清	mg/kg	181587-01-9	ND	0.01
103	#乙嘧酚	mg/kg	23947-60-6	ND	0.01
104	#乙氧嘧磺隆	mg/kg	126801-58-9	ND	0.01
105	#乙氧苯草胺	mg/kg	79540-50-4	ND	0.01
106	#乙螨唑	mg/kg	153233-91-1	ND	0.01
107	咪唑菌酮	mg/kg	161326-34-7	ND	0.01

除非另有书面协议，本报告由本公司依据《 服务通用条款 》出具。
《 服务通用条款 》印刷在正本报告纸背面，或通过 https://www.sgs.com/en/Terms-and-Conditions 查询。请特别关注其中涉及责任限定，赔偿以及司法管辖的相关条款。任何报告的持有方需知悉，此报告内容仅反映SGS受限于客户指示下，且在当时所得结论。SGS仅对其客户负责，并且此报告不能免除交易各方根据交易文件所享有的权利和应履行的义务。未获得本公司书面批准，本文件不得进行复制，全文除外。任何未经授权对报告内容及形式进行的修改、伪造或扭曲都是违法行为，违法者将会被追究法律责任。除非另有声明，本测试报告所示结果仅涉及受测试样品。
注意：检测/检验报告或证书的真实性，请通过电话 (86－755) 83071443 或邮箱 CN.Doccheck@sgs.com 查询。

No.1, 5/F., Building D1, No. 1666, Section 2 of Chenglong Road, Economic and Technological Development Zone, Longquanyi District, Chengdu, Sichuan, China 610100　t (86–28) 8481 0338　www.sgsgroup.com.cn
中国・四川・成都市龙泉驿区经济技术开发区成龙大道二段1666号 D1栋05层1号　邮编：610100　t (86–28) 8481 0338　sgs.china@sgs.com

Member of the SGS Group (SGS SA)

编号	检测项目	单位	CAS No.	检测结果 1	定量限
108	喹螨醚	mg/kg	120928-09-8	ND	0.01
109	腈苯唑	mg/kg	114369-43-6	ND	0.01
110	#甲呋酰胺	mg/kg	24691-80-3	ND	0.01
111	环酰菌胺	mg/kg	126833-17-8	ND	0.01
112	#仲丁威	mg/kg	3766-81-2	ND	0.01
113	#稻瘟酰胺（氰菌胺）	mg/kg	115852-48-7	ND	0.01
114	#恶唑禾草灵	mg/kg	66441-23-4	ND	0.01
115	双氧威（苯醚威）	mg/kg	72490-01-8 /79127-80-3	ND	0.01
116	苯锈啶	mg/kg	67306-00-7	ND	0.01
117	丁苯吗啉	mg/kg	67564-91-4	ND	0.01
118	唑螨酯	mg/kg	111812-58-9	ND	0.01
119	倍硫磷	mg/kg	55-38-9	ND	0.01
120	#倍硫磷砜	mg/kg	3761-42-0	ND	0.01
121	#倍硫磷亚砜	mg/kg	3761-41-9	ND	0.01
122	#四唑酰草胺（四唑啉酮）	mg/kg	158237-07-1	ND	0.01
123	#嘧菌腙	mg/kg	89269-64-7	ND	0.01
124	氟虫腈	mg/kg	120068-37-3	ND	0.01
125	#氟虫腈亚砜	mg/kg	120067-83-6	ND	0.01
126	#氟虫腈砜	mg/kg	120068-36-2	ND	0.01
127	#甲基麦草氟异丙酯（麦草氟甲酯）	mg/kg	52756-25-9	ND	0.01
128	#啶嘧磺隆	mg/kg	104040-78-0	ND	0.01
129	氟啶虫酰胺	mg/kg	158062-67-0	ND	0.01
130	#双氟磺草胺	mg/kg	145701-23-1	ND	0.01
131	#吡氟禾草灵和精吡氟禾草灵	mg/kg	69806-50-4 /79241-46-6	ND	0.01
132	#氟啶胺	mg/kg	79622-59-6	ND	0.01
133	#氟虫双酰胺（氟苯虫酰胺）	mg/kg	272451-65-7	ND	0.01
134	#氟唑磺隆	mg/kg	181274-17-9	ND	0.01
135	咯菌腈	mg/kg	131341-86-1	ND	0.01
136	#氟噻草胺	mg/kg	142459-58-3	ND	0.01
137	氟虫脲	mg/kg	101463-69-8	ND	0.01
138	#丁虫腈	mg/kg	704886-18-0	ND	0.01
139	#唑嘧磺草胺	mg/kg	98967-40-9	ND	0.01
140	#丙炔氟草胺	mg/kg	103361-09-7	ND	0.02
141	#氟吗啉	mg/kg	211867-47-9	ND	0.01
142	#伏草隆（氟草隆）	mg/kg	2164-17-2	ND	0.01
143	氟吡菌胺	mg/kg	239110-15-7	ND	0.01
144	#氯氟吡氧乙酸异辛酯	mg/kg	81406-37-3	ND	0.01
145	#磺菌胺	mg/kg	106917-52-6	ND	0.01
146	#氟噻乙草酯（氟赛乙草酯）	mg/kg	117337-19-6	ND	0.05

除非另有书面协议，本报告由本公司依据《 服务通用条款 》出具。
《 服务通用条款 》印刷在正本报告纸背面，或通过 https://www.sgs.com/en/Terms-and-Conditions 查询。请特别关注其中涉及责任限定，赔偿以及司法管辖的相关条款。任何报告的持有方需知悉，此报告内容仅反映SGS受限于客户指示下，且在当时所得结论。SGS仅对其客户负责，并且此报告不能免除交易各方根据交易文件所享有的权利和应履行的义务。未获得本公司书面批准，本文件不得进行复制，全文除外。任何未经授权对报告内容及形式进行的修改、伪造或扭曲都是违法行为，违法者将会被追究法律责任。除非另有声明，本测试报告所示结果仅涉及受测试样品。
注意：检测/检验报告或证书的真实性，请通过电话 (86－755) 83071443 或邮箱 CN.Doccheck@sgs.com 查询。

No.1, 5/F., Building D1, No. 1666, Section 2 of Chenglong Road, Economic and Technological Development Zone, Longquanyi District, Chengdu, Sichuan, China 610100 t (86–28) 8481 0338 www.sgsgroup.com.cn
中国・四川・成都市龙泉驿区经济技术开发区成龙大道二段1666号 D1栋05层1号 邮编：610100 t (86–28) 8481 0338 sgs.china@sgs.com

Member of the SGS Group (SGS SA)

通标标准技术服务有限公司四川分公司 检验检测专用章 Inspection & Testing Services 农产食品实验室

检测报告

CDF25-0001880-01 发布日期：2025-05-06

编号	检测项目	单位	CAS No.	检测结果 1	定量限
147	粉唑醇	mg/kg	76674-21-0	ND	0.01
148	#氟磺胺草醚	mg/kg	72178-02-0	ND	0.01
149	#氯吡脲	mg/kg	68157-60-8	ND	0.01
150	#福拉比（呋比菌胺）	mg/kg	123572-88-3	ND	0.01
151	#呋线威	mg/kg	65907-30-4	ND	0.01
152	#氯吡嘧磺隆	mg/kg	100784-20-1	ND	0.01
153	#氟吡禾灵	mg/kg	69806-34-4	ND	0.01
154	#氟吡乙禾灵	mg/kg	87237-48-7	ND	0.01
155	#氟吡甲禾灵	mg/kg	69806-40-2	ND	0.01
156	己唑醇	mg/kg	79983-71-4	ND	0.01
157	#氟铃脲	mg/kg	86479-06-3	ND	0.01
158	噻螨酮	mg/kg	78587-05-0	ND	0.01
159	抑霉唑（烯菌灵）	mg/kg	35554-44-0	ND	0.01
160	#醚草酸甲酯	mg/kg	81405-85-8	ND	0.01
161	#甲氧咪草烟	mg/kg	114311-32-9	ND	0.01
162	#甲咪唑烟酸	mg/kg	104098-48-8	ND	0.01
163	#咪唑喹啉酸（灭草喹）	mg/kg	81335-37-7	ND	0.01
164	#亚胺唑	mg/kg	86598-92-7	ND	0.01
165	吡虫啉	mg/kg	138261-41-3	ND	0.01
166	#氯噻啉	mg/kg	105843-36-5	ND	0.01
167	#茚草酮	mg/kg	133220-30-1	ND	0.01
168	茚虫威（安打）	mg/kg	173584-44-6	ND	0.01
169	#甲基碘磺隆（钠盐）	mg/kg	144550-36-7	ND	0.01
170	#碘苯腈	mg/kg	1689-83-4	ND	0.01
171	#异稻瘟净	mg/kg	26087-47-8	ND	0.01
172	异菌脲	mg/kg	36734-19-7	ND	0.01
173	缬霉威	mg/kg	140923-17-7	ND	0.01
174	#异丙威	mg/kg	2631-40-5	ND	0.01
175	#稻瘟灵	mg/kg	50512-35-1	ND	0.01
176	#异丙隆	mg/kg	34123-59-6	ND	0.01
177	#异唑隆（异恶隆）	mg/kg	55861-78-4	ND	0.01
178	#双苯恶唑酸	mg/kg	163520-33-0	ND	0.01
179	#异恶氟草（异恶唑草酮）	mg/kg	141112-29-0	ND	0.01
180	醚菌酯（苯氧菊酯）	mg/kg	143390-89-0	ND	0.01
181	#乳氟禾草灵	mg/kg	77501-63-4	ND	0.01
182	#环草定（环草啶）	mg/kg	2164-08-1	ND	0.01
183	利谷隆	mg/kg	330-55-2	ND	0.01
184	虱螨脲（氟丙氧脲）	mg/kg	103055-07-8	ND	0.01
185	马拉硫磷	mg/kg	121-75-5	ND	0.01
186	双炔酰菌胺	mg/kg	374726-62-2	ND	0.01

检验检测专用章
Inspection & Testing Services

除非另有书面协议，本报告由本公司依据《 服务通用条款 》出具。
《 服务通用条款 》印刷在正本报告纸背面，或通过 https://www.sgs.com/en/Terms-and-Conditions 查询。请特别关注其中涉及责任限定，赔偿以及司法管辖的相关条款。任何报告的持有方需知悉，此报告内容仅反映SGS受限于客户指示下，且在当时所得结论。SGS仅对其客户负责，并且此报告不能免除交易各方根据交易文件所享有的权利和应履行的义务。未获得本公司书面批准，本文件不得进行复制，全文除外。任何未经授权对报告内容及形式进行的修改、伪造或扭曲都是违法行为，违法者将会被追究法律责任。除非另有声明，本测试报告所示结果仅涉及受测试样品。
注意：检测/检验报告或证书的真实性，请通过电话(86－755) 83071443或邮箱 CN.Doccheck@sgs.com查询。

No.1, 5/F., Building D1, No. 1666, Section 2 of Chenglong Road, Economic and Technological Development Zone, Longquanyi District, Chengdu, Sichuan, China 610100 t (86–28) 8481 0338 www.sgsgroup.com.cn
中国・四川・成都市龙泉驿区经济技术开发区成龙大道二段1666号 D1栋05层1号 邮编：610100 t (86–28) 8481 0338 sgs.china@sgs.com

Member of the SGS Group (SGS SA)

编号	检测项目	单位	CAS No.	检测结果 1	定量限
187	#2 甲 4 氯	mg/kg	94-74-6	ND	0.01
188	#苯噻酰草胺	mg/kg	73250-68-7	ND	0.01
189	#吡咯解草酯（吡咯二酸二乙酯）	mg/kg	135590-91-9	ND	0.01
190	嘧菌胺	mg/kg	110235-47-7	ND	0.01
191	#甲基二磺隆	mg/kg	208465-21-8	ND	0.01
192	#硝磺草酮	mg/kg	104206-82-8	ND	0.01
193	#甲霜灵和精甲霜灵	mg/kg	57837-19-1 /70630-17-0	ND	0.01
194	甲霜灵（阿普隆）（瑞毒霜）	mg/kg	57837-19-1	ND	0.01
195	苯嗪草酮（灭它通）	mg/kg	41394-05-2	ND	0.01
196	#叶菌唑	mg/kg	125116-23-6	ND	0.01
197	#甲草苯噻隆（甲草苯隆）	mg/kg	18691-97-9	ND	0.01
198	甲胺磷	mg/kg	10265-92-6	ND	0.01
199	杀扑磷	mg/kg	950-37-8	ND	0.01
200	甲硫威（灭虫威）	mg/kg	2032-65-7	ND	0.01
201	灭多威	mg/kg	16752-77-5	ND	0.01
202	甲氧虫酰肼	mg/kg	161050-58-4	ND	0.05
203	异丙甲草胺	mg/kg	51218-45-2	ND	0.01
204	#速灭威	mg/kg	1129-41-5	ND	0.01
205	苯菌酮	mg/kg	220899-03-6	ND	0.01
206	#甲磺隆	mg/kg	74223-64-6	ND	0.05
207	#禾草敌	mg/kg	2212-67-1	ND	0.01
208	久效磷	mg/kg	6923-22-4	ND	0.01
209	腈菌唑（灭克落）	mg/kg	88671-89-0	ND	0.01
210	#萘草胺	mg/kg	132-66-1	ND	0.01
211	#烟嘧磺隆	mg/kg	111991-09-4	ND	0.01
212	#氟草敏（哒草伏）	mg/kg	27314-13-2	ND	0.01
213	#氟酰脲	mg/kg	116714-46-6	ND	0.01
214	#呋酰胺	mg/kg	58810-48-3	ND	0.01
215	氧乐果	mg/kg	1113-02-6	ND	0.01
216	#嘧苯胺磺隆	mg/kg	213464-77-8	ND	0.01
217	#丙炔噁草酮	mg/kg	39807-15-3	ND	0.01
218	恶草酮	mg/kg	19666-30-9	ND	0.01
219	恶霜灵	mg/kg	77732-09-3	ND	0.01
220	杀线威	mg/kg	23135-22-0	ND	0.01
221	#氯恶嗪草	mg/kg	153197-14-9	ND	0.01
222	#氧化萎锈灵	mg/kg	5259-88-1	ND	0.01
223	纹枯脲（戊菌隆）	mg/kg	66063-05-6	ND	0.01
224	#五氟磺草胺	mg/kg	219714-96-2	ND	0.01
225	甜菜宁	mg/kg	13684-63-4	ND	0.01

除非另有书面协议，本报告由本公司依据《 服务通用条款 》出具。
《 服务通用条款 》印刷在正本报告纸背面，或通过 https://www.sgs.com/en/Terms-and-Conditions 查询。请特别关注其中涉及责任限定，赔偿以及司法管辖的相关条款。任何报告的持有方需知悉，此报告内容仅反映SGS受限于客户指示下，且在当时所得结论。SGS仅对其客户负责，并且此报告不能免除交易各方根据交易文件所享有的权利和应履行的义务。未获得本公司书面批准，本文件不得进行复制，全文除外。任何未经授权对报告内容及形式进行的修改、伪造或扭曲都是违法行为，违法者将会被追究法律责任。除非另有声明，本测试报告所示结果仅涉及受测试样品。
注意：检测/检验报告或证书的真实性，请通过电话 (86 –755) 83071443 或邮箱 CN.Doccheck@sgs.com 查询。

No.1, 5/F., Building D1, No. 1666, Section 2 of Chenglong Road, Economic and Technological Development Zone, Longquanyi District, Chengdu, Sichuan, China 610100 t (86–28) 8481 0338 www.sgsgroup.com.cn
中国・四川・成都市龙泉驿区经济技术开发区成龙大道二段1666号 D1栋05层1号 邮编：610100 t (86–28) 8481 0338 sgs.china@sgs.com

Member of the SGS Group (SGS SA)

编号	检测项目	单位	CAS No.	检测结果 1	定量限
226	伏杀硫磷	mg/kg	2310-17-0	ND	0.01
227	#硫环磷	mg/kg	947-02-4	ND	0.01
228	#甲基硫环磷	mg/kg	5120-23-0	ND	0.01
229	亚胺硫磷	mg/kg	732-11-6	ND	0.01
230	辛硫磷	mg/kg	14816-18-3	ND	0.01
231	#氟吡草胺	mg/kg	137641-05-5	ND	0.01
232	#啶氧菌酯	mg/kg	117428-22-5	ND	0.01
233	抗蚜威	mg/kg	23103-98-2	ND	0.01
234	#烯丙苯噻唑	mg/kg	27605-76-1	ND	0.01
235	#咪鲜胺	mg/kg	67747-09-5	ND	0.01
236	#茉莉酮（茉莉酸导体）	mg/kg	158474-72-7	ND	0.01
237	#猛杀威	mg/kg	2631-37-0	ND	0.01
238	#毒草胺	mg/kg	1918-16-7	ND	0.01
239	霜霉威	mg/kg	24579-73-5	ND	0.01
240	#恶草酸	mg/kg	111479-05-1	ND	0.01
241	炔螨特	mg/kg	2312-35-8	ND	0.01
242	#异丙草胺	mg/kg	86763-47-5	ND	0.01
243	残杀威	mg/kg	114-26-1	ND	0.01
244	苄草丹	mg/kg	52888-80-9	ND	0.01
245	#氟磺隆	mg/kg	94125-34-5	ND	0.01
246	吡蚜酮（吡嗪酮）	mg/kg	123312-89-0	ND	0.01
247	吡唑醚菌酯（唑菌胺酯）（百克敏）	mg/kg	175013-18-0	ND	0.01
248	#唑菌酯	mg/kg	862588-11-2	ND	0.01
249	#吡唑特	mg/kg	58011-68-0	ND	0.01
250	#吡菌磷	mg/kg	13457-18-6	ND	0.01
251	#乙基吡嘧磺隆（百速隆）	mg/kg	93697-74-6	ND	0.01
252	#苄草唑	mg/kg	71561-11-0	ND	0.01
253	#稗草丹	mg/kg	88678-67-5	ND	0.01
254	哒螨灵	mg/kg	96489-71-3	ND	0.01
255	#啶虫丙醚	mg/kg	179101-81-6	ND	0.01
256	#哒嗪硫磷	mg/kg	119-12-0	ND	0.01
257	啶斑肟（比芬诺）	mg/kg	88283-41-4	ND	0.01
258	#环酯草醚	mg/kg	135186-78-6	ND	0.01
259	嘧霉胺	mg/kg	53112-28-0	ND	0.01
260	#嘧螨醚	mg/kg	105779-78-0	ND	0.01
261	#吡丙醚（蚊蝇醚）	mg/kg	95737-68-1	ND	0.01
262	#咯哇酮（百快隆）	mg/kg	57369-32-1	ND	0.01
263	#灭藻醌	mg/kg	2797-51-5	ND	0.01
264	喹氧灵	mg/kg	124495-18-7	ND	0.01
265	#喹禾灵和精喹禾灵	mg/kg	100646-51-3	ND	0.01

除非另有书面协议，本报告由本公司依据《服务通用条款》出具。
《服务通用条款》印刷在正本报告纸背面，或通过 https://www.sgs.com/en/Terms-and-Conditions 查询。请特别关注其中涉及责任限定，赔偿以及司法管辖的相关条款。任何报告的持有方需知悉，此报告内容仅反映SGS受限于客户指示下，且在当时所得结论。SGS仅对其客户负责，并且此报告不能免除交易各方根据交易文件所享有的权利和应履行的义务。未获得本公司书面批准，本文件不得进行复制，全文除外。任何未经授权对报告内容及形式进行的修改、伪造或扭曲都是违法行为，违法者将会被追究法律责任。除非另有声明，本测试报告所示结果仅涉及受测试样品。
注意：检测/检验报告或证书的真实性，请通过电话 (86－755) 83071443 或邮箱 CN.Doccheck@sgs.com 查询。

检验检测专用章 Inspection & Testing Services
通标标准技术服务有限公司四川分公司 农产食品实验室

No.1, 5F, Building D1, No. 1666, Section 2 of Chenglong Road, Economic and Technological Development Zone, Longquanyi District, Chengdu, Sichuan, China 610100 t (86–28) 8481 0338 www.sgsgroup.com.cn
中国・四川・成都市龙泉驿区经济技术开发区成龙大道二段1666号 D1栋05层1号 邮编：610100 t (86–28) 8481 0338 sgs.china@sgs.com

Member of the SGS Group (SGS SA)

编号	检测项目	单位	CAS No.	检测结果 1	定量限
			/76578-14-8		
266	#砜嘧磺隆	mg/kg	122931-48-0	ND	0.01
267	#鱼藤酮	mg/kg	83-79-4	ND	0.01
268	#单甲脒	mg/kg	33089-74-6	ND	0.01
269	#稀禾定	mg/kg	74051-80-2	ND	0.01
270	#西玛津	mg/kg	122-34-9	ND	0.01
271	#硅氟唑	mg/kg	149508-90-7	ND	0.01
272	#西草净	mg/kg	1014-70-6	ND	0.01
273	螺螨酯	mg/kg	148477-71-8	ND	0.01
274	#螺虫乙酯	mg/kg	203313-25-1	ND	0.01
275	螺恶茂胺（螺环菌胺）	mg/kg	118134-30-8	ND	0.01
276	#磺草酮	mg/kg	99105-77-8	ND	0.01
277	#乙黄隆（磺酰磺隆）	mg/kg	141776-32-1	ND	0.05
278	#氟啶虫胺腈	mg/kg	946578-00-3	ND	0.01
279	戊唑醇	mg/kg	107534-96-3	ND	0.01
280	虫酰肼	mg/kg	112410-23-8	ND	0.01
281	吡螨胺	mg/kg	119168-77-3	ND	0.02
282	#特丁隆	mg/kg	34014-18-1	ND	0.01
283	氟苯脲	mg/kg	83121-18-0	ND	0.01
284	#噻吩草胺（甲氧噻草胺）	mg/kg	96491-05-3	ND	0.01
285	噻菌灵	mg/kg	148-79-8	ND	0.01
286	噻虫啉	mg/kg	111988-49-9	ND	0.01
287	噻虫嗪	mg/kg	153719-23-4	ND	0.01
288	#噻唑烟酸（噻草啶）	mg/kg	117718-60-2	ND	0.01
289	#赛苯隆	mg/kg	51707-55-2	ND	0.01
290	#噻吩磺隆（阔叶散）	mg/kg	79277-27-3	ND	0.01
291	#噻呋酰胺	mg/kg	130000-40-7	ND	0.01
292	#禾草丹	mg/kg	28249-77-6	ND	0.01
293	#硫双威	mg/kg	59669-26-0	ND	0.01
294	#久效威砜	mg/kg	39184-59-3	ND	0.01
295	#久效威亚砜	mg/kg	39184-27-5	ND	0.01
296	#甲基硫菌灵	mg/kg	23564-05-8	ND	0.01
297	#噻酰菌胺	mg/kg	223580-51-6	ND	0.01
298	#唑虫酰胺	mg/kg	129558-76-5	ND	0.01
299	#苯草酮（甲氧基草酮）	mg/kg	87820-88-0	ND	0.01
300	#三唑酮	mg/kg	43121-43-3	ND	0.01
301	三唑醇	mg/kg	55219-65-3	ND	0.01
302	#醚苯磺隆	mg/kg	82097-50-5	ND	0.01
303	三唑磷	mg/kg	24017-47-8	ND	0.01
304	#苯磺隆	mg/kg	101200-48-0	ND	0.01

通标标准技术服务有限公司四川分公司 检验检测专用章 Inspection & Testing Services
通标标准技术服务有限公司四川分公司 农产食品实验室

除非另有书面协议，本报告由本公司依据《服务通用条款》出具。
《服务通用条款》印刷在正本报告纸背面，或通过 https://www.sgs.com/en/Terms-and-Conditions 查询。请特别关注其中涉及责任限定，赔偿以及司法管辖的相关条款。任何报告的持有方需知悉，此报告内容仅反映SGS受限于客户指示下，且在当时所得结论。SGS仅对其客户负责，并且此报告不能免除交易各方根据交易文件所享有的权利和应履行的义务。未获得本公司书面批准，本文件不得进行复制，全文除外。任何未经授权对报告内容及形式进行的修改、伪造或扭曲都是违法行为，违法者将会被追究法律责任。除非另有声明，本测试报告所示结果仅涉及受测试样品。
注意：检测/检验报告或证书的真实性，请通过电话(86-755) 83071443或邮箱 CN.Doccheck@sgs.com查询。
No.1, 5/F, Building D1, No. 1666, Section 2 of Chengdong Road, Economic and Technological Development Zone, Longquanyi District, Chengdu, Sichuan, China 610100　t (86–28) 8481 0338　www.sgsgroup.com.cn
中国・四川・成都市龙泉驿区经济技术开发区成龙大道二段1666号 D1栋05层1号　邮编：610100　t (86–28) 8481 0338　sgs.china@sgs.com
Member of the SGS Group (SGS SA)

编号	检测项目	单位	CAS No.	检测结果 1	定量限
305	#敌百虫	mg/kg	52-68-6	ND	0.01
306	#氯啶菌酯	mg/kg	902760-40-1	ND	0.01
307	三环唑	mg/kg	41814-78-2	ND	0.01
308	#十三吗啉	mg/kg	24602-86-6	ND	0.01
309	肟菌酯	mg/kg	141517-21-7	ND	0.01
310	氟菌唑	mg/kg	68694-11-1	ND	0.01
311	杀铃脲	mg/kg	64628-44-0	ND	0.01
312	#氟胺磺隆	mg/kg	126535-15-7	ND	0.01
313	#抗倒酯	mg/kg	95266-40-3	ND	0.01
314	#灭菌唑	mg/kg	131983-72-7	ND	0.01
315	#烯效唑和高效烯效唑	mg/kg	83657-17-4 /83657-22-1	ND	0.01
316	#蚜灭磷（完灭硫磷）	mg/kg	2275-23-2	ND	0.01
317	#灭除威	mg/kg	2655-14-3	ND	0.01
318	苯酰菌胺（苯酰草胺）	mg/kg	156052-68-5	ND	0.01
319	#三唑酮和三唑醇之和	mg/kg	-	ND	-
320	#灭多威（灭多威和硫双威之和）	mg/kg	-	ND	-
321	克百威(克百威和 3-羟基克百威之和，以克百威表示)	mg/kg	-	ND	-
322	#氟虫腈(氟虫腈、氟虫腈砜及氟虫腈亚砜之和，以氟虫腈表示)	mg/kg	-	ND	-
323	#涕灭威(涕灭威、涕灭威亚砜及涕灭砜威之和，以涕灭威表示)	mg/kg	-	ND	-
324	#倍硫磷(倍硫磷、倍硫磷亚砜及倍硫磷砜之和，以倍硫磷表示)	mg/kg	-	ND	-
325	#乙硫甲威（乙硫甲威,乙硫甲威-砜,乙硫甲威-亚砜之和，以乙硫甲威表示）	mg/kg	-	ND	-
326	#环丙氨嗪(灭蝇胺)	mg/kg	66215-27-8	ND	0.01
327	#二硫酮砜	mg/kg	2497-06-5	ND	0.01
328	#二硫代亚砜	mg/kg	2497-07-6	ND	0.01
329	#氟甲腈	mg/kg	205650-65-3	ND	0.01
330	#噁唑菌酮	mg/kg	131807-57-3	ND	0.01
331	#咪鲜胺-脱氨基咪唑	mg/kg	139520-94-8	ND	0.05
332	#咪鲜胺-脱咪唑甲酰胺基	mg/kg	139542-32-8	ND	0.05
333	#丁氟螨酯	mg/kg	400882-07-7	ND	0.01
334	#苯嘧磺草胺	mg/kg	372137-35-4	ND	0.01
335	#氟唑环菌胺	mg/kg	874967-67-6	ND	0.01
336	#胺苯吡菌酮	mg/kg	473798-59-3	ND	0.01
337	#消螨酚	mg/kg	131-89-5	ND	0.01
338	#戊硝酚	mg/kg	4097-36-3	ND	0.01
339	#烯啶虫胺	mg/kg	120738-89-8	ND	0.01

除非另有书面协议，本报告由本公司依据《 服务通用条款 》出具。
《 服务通用条款 》印刷在正本报告纸背面，或通过 https://www.sgs.com/en/Terms-and-Conditions 查询。请特别关注其中涉及责任限定，赔偿以及司法管辖的相关条款。任何报告的持有方需知悉，此报告内容仅反映SGS受限于客户指示下，且在当时所得结论。SGS仅对其客户负责，并且此报告不能免除交易各方根据交易文件所享有的权利和应履行的义务。未获得本公司书面批准，本文件不得进行复制，全文除外。任何未经授权对报告内容及形式进行的修改、伪造或扭曲都是违法行为，违法者将会被追究法律责任。除非另有声明，本测试报告所示结果仅涉及受测试样品。
注意：检测/检验报告或证书的真实性，请通过电话 (86-755) 83071443 或邮箱 CN.Doccheck@sgs.com 查询。
No.1, 5F, Building D1, No. 1666, Section 2 of Chenglong Road, Economic and Technological Development Zone, Longquanyi District, Chengdu, Sichuan, China 610100 t (86–28) 8481 0338 www.sgsgroup.com.cn
中国・四川・成都市龙泉驿区经济技术开发区成龙大道二段1666号 D1栋05层1号 邮编：610100 t (86–28) 8481 0338 sgs.china@sgs.com
Member of the SGS Group (SGS SA)

编号	检测项目	单位	CAS No.	检测结果 1	定量限
			/150824-47-8		
340	#联苯肼酯	mg/kg	149877-41-8	ND	0.01
341	#吡噻菌胺	mg/kg	183675-82-3	ND	0.01
342	2,4-滴 & 2,4-滴钠盐	mg/kg	2702-72-9 /94-75-7	ND	0.01
343	#伊维菌素	mg/kg	70288-86-7	ND	0.05
344	#除虫菊素 I	mg/kg	121-21-1	ND	0.01
345	#除虫菊素 II	mg/kg	121-29-9	ND	0.01
346	#氟咯草酮	mg/kg	61213-25-0	ND	0.01
347	#扑草净	mg/kg	7287-19-6	ND	0.01
348	多杀霉素	mg/kg	168316-95-8	ND	0.01
349	#胺苯磺隆	mg/kg	97780-06-8	ND	0.01
350	#唑嘧菌胺	mg/kg	865318-97-4	ND	0.01
351	#噻草酮	mg/kg	101205-02-1	ND	0.01
352	#唑胺菌酯	mg/kg	915410-70-7	ND	0.01
353	#丁吡吗啉	mg/kg	868390-90-3	ND	0.01
354	#啶菌噁唑	mg/kg	847749-37-5	ND	0.01
355	#乙基多杀菌素 J	mg/kg	187166-40-1	ND	0.01
356	#咪鲜胺(咪鲜胺、咪鲜胺-脱氨基咪唑及咪鲜胺-脱咪唑甲酰胺基之和，以咪鲜胺表示)	mg/kg	-	ND	-
357	#胺鲜酯	mg/kg	10369-83-2	ND	0.05
358	#苯菌灵和多菌灵	mg/kg	-	ND	0.01
359	#苯并烯氟菌唑	mg/kg	1072957-71-1	ND	0.05
360	#烯草酮亚砜	mg/kg	111031-14-2	ND	0.01
361	#烯草酮砜	mg/kg	111031-17-5	ND	0.01
362	#螺虫乙酯-酮基-羟基	mg/kg	1172134-11-0	ND	0.01
363	#螺虫乙酯-烯醇-葡萄糖苷	mg/kg	1172614-86-6	ND	0.01
364	#螺虫乙酯-单-羟基	mg/kg	1172134-12-1	ND	0.01
365	#甲磺草胺	mg/kg	122836-35-5	ND	0.05
366	#丰索磷砜	mg/kg	14255-72-2	ND	0.01
367	#螺虫乙酯-烯醇	mg/kg	203312-38-3	ND	0.01
368	#甲硫威砜	mg/kg	2179-25-1	ND	0.01
369	#甲硫威亚砜	mg/kg	2635-10-1	ND	0.01
370	#烯肟菌酯	mg/kg	238410-11-2	ND	0.01
371	#N-二甲基甲酰基甲硫基甲醛肟	mg/kg	30558-43-1	ND	0.01
372	#烯肟菌胺	mg/kg	366815-39-6	ND	0.01
373	#阿苯达唑	mg/kg	54965-21-8	ND	0.01

除非另有书面协议，本报告由本公司依据《服务通用条款》出具。
《服务通用条款》印刷在正本报告纸背面，或通过 https://www.sgs.com/en/Terms-and-Conditions 查询。请特别关注其中涉及责任限定，赔偿以及司法管辖的相关条款。任何报告的持有方需知悉，此报告内容仅反映SGS受限于客户指示下，且在当时所得结论。SGS仅对其客户负责，并且此报告不能免除交易各方根据交易文件所享有的权利和应履行的义务。未获得本公司书面批准，本文件不得进行复制，全文除外。任何未经授权对报告内容及形式进行的修改、伪造或扭曲都是违法行为，违法者将会被追究法律责任。除非另有声明，本测试报告所示结果仅涉及受测试样品。
注意：检测/检验报告或证书的真实性，请通过电话 (86-755) 83071443 或邮箱 CN.Doccheck@sgs.com 查询。
No.1, 5F, Building D1, No. 1666, Section 2 of Chenglong Road, Economic and Technological Development Zone, Longquanyi District, Chengdu, Sichuan, China 610100 t (86–28) 8481 0338 www.sgsgroup.com.cn
中国・四川・成都市龙泉驿区经济技术开发区成龙大道二段1666号 D1栋05层1号 邮编：610100 t (86–28) 8481 0338 sgs.china@sgs.com
Member of the SGS Group (SGS SA)

编号	检测项目	单位	CAS No.	检测结果 1	定量限
374	#丙嗪嘧磺隆	mg/kg	570415-88-2	ND	0.01
375	#丰索磷-氧化砜	mg/kg	6132-17-8	ND	0.01
376	#氟节胺	mg/kg	62924-70-3	ND	0.05
377	#丰索磷-氧	mg/kg	6552-21-2	ND	0.01
378	#丁香菌酯	mg/kg	850881-70-8	ND	0.01
379	#嗪吡嘧磺隆	mg/kg	868680-84-6	ND	0.05
380	#吡唑萘菌胺	mg/kg	881685-58-1	ND	0.01
381	#螺虫乙酯(螺虫乙酯、螺虫乙酯-烯醇、螺虫乙酯-烯醇-葡萄糖苷、螺虫乙酯-酮基-羟基及螺虫乙酯-单-羟基之和，以螺虫乙酯表示)	mg/kg	-	ND	0.01
382	#甲硫威(甲硫威、甲硫威亚砜及甲硫威砜之和，以甲硫威表示)	mg/kg	-	ND	-
383	#亚砜磷(亚砜磷、甲基内吸磷及砜吸磷之和，以亚砜磷表示)	mg/kg	-	ND	-
384	#烯草酮(烯草酮、烯草酮亚砜及烯草酮砜之和，以烯草酮表示)	mg/kg	-	ND	-
385	#丰索磷(丰索磷、氧丰索磷、氧丰索磷砜及丰索磷砜，以丰索磷表示)	mg/kg	-	ND	-
386	#除虫菊素(除虫菊素 I 和除虫菊素 II 之和)	mg/kg	-	ND	-
387	#乙草胺	mg/kg	34256-82-1	ND	0.01
388	#氟丙菊酯	mg/kg	101007-06-1	ND	0.1
389	甲草胺	mg/kg	15972-60-8	ND	0.01
390	艾氏剂	mg/kg	309-00-2	ND	0.01
391	#丙烯菊酯	mg/kg	584-79-2	ND	0.01
392	#二丙烯草胺	mg/kg	93-71-0	ND	0.01
393	莠灭净	mg/kg	834-12-8	ND	0.02
394	#蒽醌	mg/kg	84-65-1	ND	0.01
395	益棉磷（乙基保棉磷）（乙基谷硫磷）	mg/kg	2642-71-9	ND	0.02
396	#苯草醚	mg/kg	74070-46-5	ND	0.01
397	α-六六六	mg/kg	319-84-6	ND	0.01
398	#燕麦灵	mg/kg	101-27-9	ND	0.05
399	噁虫威	mg/kg	22781-23-3	ND	0.01
400	乙丁氟灵	mg/kg	1861-40-1	ND	0.01
401	#呋草黄（呋草磺）	mg/kg	68505-69-1	ND	0.01
402	β-六六六	mg/kg	319-85-7	ND	0.01
403	#甲羧除草醚	mg/kg	42576-02-3	ND	0.01
404	联苯菊酯	mg/kg	82657-04-3	ND	0.01
405	乙基溴硫磷	mg/kg	4824-78-6	ND	0.01
406	溴硫磷	mg/kg	2104-96-3	ND	0.01

除非另有书面协议，本报告由本公司依据《 服务通用条款 》出具。
《 服务通用条款 》印刷在正本报告纸背面，或通过 https://www.sgs.com/en/Terms-and-Conditions 查询。请特别关注其中涉及责任限定，赔偿以及司法管辖的相关条款。任何报告的持有方需知悉，此报告内容仅反映SGS受限于客户指示下，且在当时所得结论。SGS仅对其客户负责，并且此报告不能免除交易各方根据交易文件所享有的权利和应履行的义务。未获得本公司书面批准，本文件不得进行复制，全文除外。任何未经授权对报告内容及形式进行的修改、伪造或扭曲都是违法行为，违法者将会被追究法律责任。除非另有声明，本测试报告所示结果仅涉及受测试样品。
注意：检测/检验报告或证书的真实性，请通过电话 (86－755) 83071443 或邮箱 CN.Doccheck@sgs.com 查询。

通标标准技术服务有限公司四川分公司 检验检测专用章 Inspection & Testing Services 农产食品实验室

No.1, 5F, Building D1, No. 1666, Section 2 of Chenglong Road, Economic and Technological Development Zone, Longquanyi District, Chengdu, Sichuan, China 610100 t (86–28) 8481 0338 www.sgsgroup.com.cn
中国・四川・成都市龙泉驿区经济技术开发区成龙大道二段1666号 D1栋05层1号 邮编：610100 t (86–28) 8481 0338 sgs.china@sgs.com

Member of the SGS Group (SGS SA)

编号	检测项目	单位	CAS No.	检测结果<br>1	定量限
407	溴螨酯	mg/kg	18181-80-1	ND	0.01
408	#丁草胺	mg/kg	23184-66-9	ND	0.01
409	#抑草磷	mg/kg	36335-67-8	ND	0.01
410	仲丁灵	mg/kg	33629-47-9	ND	0.01
411	#丁草敌（丁草特）	mg/kg	2008-41-5	ND	0.01
412	δ-六六六	mg/kg	319-86-8	ND	0.01
413	γ-六六六（林丹）	mg/kg	58-89-9	ND	0.01
414	硫线磷	mg/kg	95465-99-9	ND	0.02
415	三硫磷	mg/kg	786-19-6	ND	0.01
416	杀螨醚（氯杀螨）	mg/kg	103-17-3	ND	0.01
417	虫螨腈（溴虫腈）	mg/kg	122453-73-0	ND	0.01
418	杀螨酯	mg/kg	80-33-1	ND	0.01
419	毒虫畏（杀螟威）	mg/kg	470-90-6	ND	0.01
420	乙酯杀螨醇（克氯苯）	mg/kg	510-15-6	ND	0.01
421	氯苯甲醚	mg/kg	2675-77-6	ND	0.02
422	#百菌清	mg/kg	1897-45-6	ND	0.01
423	氯苯胺灵	mg/kg	101-21-3	ND	0.01
424	毒死蜱	mg/kg	2921-88-2	ND	0.01
425	甲基毒死蜱	mg/kg	5598-13-0	ND	0.01
426	氯酞酸甲酯	mg/kg	1861-32-1	ND	0.01
427	乙菌利（克氯得）	mg/kg	84332-86-5	ND	0.01
428	顺式-氯丹（α-氯丹）	mg/kg	5103-71-9	ND	0.01
429	炔草酯（炔草酸）	mg/kg	105512-06-9	ND	0.01
430	杀螟腈（杀虫腈）	mg/kg	2636-26-2	ND	0.01
431	异噁草松（异噁草酮）	mg/kg	81777-89-1	ND	0.01
432	#解草酯（解毒喹）	mg/kg	99607-70-2	ND	0.01
433	#蝇毒磷	mg/kg	56-72-4	ND	0.01
434	#氟氯氰菊酯和高效氟氯氰菊酯	mg/kg	1820573-27-0<br>/68359-37-5	ND	0.01
435	#氰氟草酯	mg/kg	122008-85-9	ND	0.01
436	#氯氟氰菊酯和高效氯氟氰菊酯	mg/kg	68085-85-8<br>/91465-08-6	ND	0.01
437	氯氰菊酯	mg/kg	52315-07-8	ND	0.01
438	环丙唑醇	mg/kg	94361-06-5	ND	0.01
439	#草枯醚	mg/kg	1836-77-7	ND	0.01
440	#丙酯杀螨醇	mg/kg	5836-10-2	ND	0.01
441	#巴毒磷	mg/kg	7700-17-6	ND	0.01
442	#2,4-滴丁酯	mg/kg	94-80-4	ND	0.01
443	溴氰菊酯	mg/kg	52918-63-5	ND	0.01
444	二嗪磷（二嗪农）	mg/kg	333-41-5	ND	0.01

除非另有书面协议，本报告由本公司依据《 服务通用条款 》出具。
《 服务通用条款 》印刷在正本报告纸背面，或通过 https://www.sgs.com/en/Terms-and-Conditions 查询。请特别关注其中涉及责任限定，赔偿以及司法管辖的相关条款。任何报告的持有方需知悉，此报告内容仅反映SGS受限于客户指示下，且在当时所得结论。SGS仅对其客户负责，并且此报告不能免除交易各方根据交易文件所享有的权利和应履行的义务。未获得本公司书面批准，本文件不得进行复制，全文除外。任何未经授权对报告内容及形式进行的修改、伪造或扭曲都是违法行为，违法者将会被追究法律责任。除非另有声明，本测试报告所示结果仅涉及受测试样品。
注意：检测/检验报告或证书的真实性，请通过电话(86－755) 83071443或邮箱 CN.Doccheck@sgs.com查询。

No.1, 5/F., Building D1, No. 1666, Section 2 of Chenglong Road, Economic and Technological Development Zone, Longquanyi District, Chengdu, Sichuan, China 610100 t (86–28) 8481 0338 www.sgsgroup.com.cn
中国・四川・成都市龙泉驿区经济技术开发区成龙大道二段1666号 D1栋05层1号 邮编：610100 t (86–28) 8481 0338 sgs.china@sgs.com

Member of the SGS Group (SGS SA)

编号	检测项目	单位	CAS No.	检测结果 1	定量限
445	#敌草腈	mg/kg	1194-65-6	ND	0.01
446	除线磷	mg/kg	97-17-6	ND	0.01
447	#苯氟磺胺（抑菌灵）	mg/kg	1085-98-9	ND	0.01
448	#烯丙酰草胺（二氯丙烯胺）	mg/kg	37764-25-3	ND	0.01
449	敌敌畏	mg/kg	62-73-7	ND	0.01
450	#苄氯三唑醇	mg/kg	75736-33-3	ND	0.01
451	禾草灵	mg/kg	51338-27-3	ND	0.01
452	氯硝胺	mg/kg	99-30-9	ND	0.01
453	p,p'-三氯杀螨醇	mg/kg	115-32-2	ND	0.01
454	狄氏剂	mg/kg	60-57-1	ND	0.01
455	#二甲吩草胺	mg/kg	87674-68-8	ND	0.01
456	#噻节因	mg/kg	55290-64-7	ND	0.01
457	#甲基毒虫畏	mg/kg	2274-67-1	ND	0.01
458	#敌噁磷（敌杀磷）	mg/kg	78-34-2	ND	0.01
459	#双苯酰草胺	mg/kg	957-51-7	ND	0.01
460	二苯胺	mg/kg	122-39-4	ND	0.01
461	#乙拌磷	mg/kg	298-04-4	ND	0.01
462	#氟硫草定	mg/kg	97886-45-8	ND	0.01
463	#甲基异内吸磷	mg/kg	919-86-8	ND	0.01
464	#灭菌磷	mg/kg	5131-24-8	ND	0.01
465	α-硫丹	mg/kg	959-98-8	ND	0.01
466	β-硫丹	mg/kg	33213-65-9	ND	0.01
467	#敌瘟磷（敌瘟散）	mg/kg	17109-49-8	ND	0.01
468	硫丹硫酸酯	mg/kg	1031-07-8	ND	0.02
469	异狄氏剂	mg/kg	72-20-8	ND	0.01
470	苯硫磷	mg/kg	2104-64-5	ND	0.01
471	#乙丁烯氟灵	mg/kg	55283-68-6	ND	0.01
472	乙硫磷	mg/kg	563-12-2	ND	0.01
473	乙氧呋草黄	mg/kg	26225-79-6	ND	0.01
474	灭线磷	mg/kg	13194-48-4	ND	0.01
475	#吲熟酯	mg/kg	27512-72-7	ND	0.01
476	醚菊酯	mg/kg	80844-07-1	ND	0.01
477	#土菌灵	mg/kg	2593-15-9	ND	0.01
478	乙嘧硫磷	mg/kg	38260-54-7	ND	0.01
479	苯线磷	mg/kg	22224-92-6	ND	0.05
480	#氯苯嘧啶醇	mg/kg	60168-88-9	ND	0.01
481	皮蝇磷	mg/kg	299-84-3	ND	0.01
482	杀螟硫磷（杀螟松）	mg/kg	122-14-5	ND	0.01
483	#苯硫威	mg/kg	62850-32-2	ND	0.01
484	甲氰菊酯	mg/kg	39515-41-8	ND	0.01

除非另有书面协议，本报告由本公司依据《服务通用条款》出具。
《服务通用条款》印刷在正本报告纸背面，或通过 https://www.sgs.com/en/Terms-and-Conditions 查询。请特别关注其中涉及责任限定，赔偿以及司法管辖的相关条款。任何报告的持有方需知悉，此报告内容仅反映SGS受限于客户指示下，且在当时所得结论。SGS仅对其客户负责，并且此报告不能免除交易各方根据交易文件所享有的权利和应履行的义务。未获得本公司书面批准，本文件不得进行复制，全文除外。任何未经授权对报告内容及形式进行的修改、伪造或扭曲都是违法行为，违法者将会被追究法律责任。除非另有声明，本测试报告所示结果仅涉及受测试样品。
注意：检测/检验报告或证书的真实性，请通过电话(86-755) 83071443或邮箱 CN.Doccheck@sgs.com查询。
No.1, 5F, Building D1, No. 1666, Section 2 of Chenglong Road, Economic and Technological Development Zone, Longquanyi District, Chengdu, Sichuan, China 610100 t (86–28) 8481 0338 www.sgsgroup.com.cn
中国・四川・成都市龙泉驿区经济技术开发区成龙大道二段1666号 D1栋05层1号 邮编：610100 t (86–28) 8481 0338 sgs.china@sgs.com
Member of the SGS Group (SGS SA)

编号	检测项目	单位	CAS No.	检测结果 1	定量限
			/64257-84-7		
485	丰索磷	mg/kg	115-90-2	ND	0.01
486	氰戊菊酯和 S-氰戊菊酯	mg/kg	51630-58-1 /66230-04-4	ND	0.01
487	嘧螨酯	mg/kg	229977-93-9	ND	0.01
488	氟氰戊菊酯	mg/kg	70124-77-5	ND	0.01
489	#胺氟草酯（氟烯草酸）	mg/kg	87546-18-7	ND	0.01
490	#氟喹唑	mg/kg	136426-54-5	ND	0.01
491	#氟啶草酮	mg/kg	59756-60-4	ND	0.01
492	氟哇唑（氟硅唑）	mg/kg	85509-19-9	ND	0.01
493	#四氯苯酞	mg/kg	27355-22-2	ND	0.01
494	#氟酰胺	mg/kg	66332-96-5	ND	0.01
495	地虫硫磷	mg/kg	944-22-9	ND	0.01
496	#安硫磷（安果磷）	mg/kg	2540-82-1	ND	0.01
497	#噻唑硫磷（噻唑磷）	mg/kg	98886-44-3	ND	0.01
498	#乙羧氟草醚	mg/kg	77501-90-7	ND	0.01
499	呋霜灵	mg/kg	57646-30-7	ND	0.01
500	#三氟硝草醚	mg/kg	15457-05-3	ND	0.01
501	#氟除草醚	mg/kg	13738-63-1	ND	0.01
502	#格螨酯	mg/kg	97-16-5	ND	0.01
503	#苄螨醚（苄满醚）	mg/kg	111872-58-3	ND	0.01
504	七氯	mg/kg	76-44-8	ND	0.01
505	环氧七氯（B 型，顺式）	mg/kg	1024-57-3 /28044-83-9	ND	0.01
506	庚烯磷	mg/kg	23560-59-0	ND	0.01
507	六氯苯	mg/kg	118-74-1	ND	0.01
508	#环嗪酮	mg/kg	51235-04-2	ND	0.01
509	#氯唑磷	mg/kg	42509-80-8	ND	0.01
510	#水胺硫磷	mg/kg	24353-61-5	ND	0.01
511	异艾氏剂	mg/kg	465-73-6	ND	0.01
512	异柳磷	mg/kg	25311-71-1	ND	0.01
513	甲基异柳磷	mg/kg	99675-03-3	ND	0.01
514	#恶唑磷	mg/kg	18854-01-8	ND	0.01
515	#灭蚜磷	mg/kg	2595-54-2	ND	0.01
516	#灭锈胺	mg/kg	55814-41-0	ND	0.01
517	虫螨畏	mg/kg	62610-77-9	ND	0.01
518	#烯虫酯	mg/kg	40596-69-8	ND	0.01
519	甲氧氯（甲氧滴滴涕）	mg/kg	72-43-5	ND	0.01
520	#五氯苯甲硫醚（甲基五氯苯硫醚）	mg/kg	1825-19-0	ND	0.01
521	嗪草酮	mg/kg	21087-64-9	ND	0.01
522	速灭磷	mg/kg	7786-34-7	ND	0.01

除非另有书面协议，本报告由本公司依据《服务通用条款》出具。《服务通用条款》印刷在正本报告纸背面，或通过 https://www.sgs.com/en/Terms-and-Conditions 查询。请特别关注其中涉及责任限定，赔偿以及司法管辖的相关条款。任何报告的持有方需知悉，此报告内容仅反映SGS受限于客户指示下，且在当时所得结论。SGS仅对其客户负责，并且此报告不能免除交易各方根据交易文件所享有的权利和应履行的义务。未获得本公司书面批准，本文件不得进行复制，全文除外。任何未经授权对报告内容及形式进行的修改、伪造或扭曲都是违法行为，违法者将会被追究法律责任。除非另有声明，本测试报告所示结果仅涉及受测试样品。
注意：检测/检验报告或证书的真实性，请通过电话 (86－755) 83071443 或邮箱 CN.Doccheck@sgs.com 查询。

No.1, 5F, Building D1, No. 1666, Section 2 of Chenglong Road, Economic and Technological Development Zone, Longquanyi District, Chengdu, Sichuan, China 610100 t (86–28) 8481 0338 www.sgsgroup.com.cn
中国·四川·成都市龙泉驿区经济技术开发区成龙大道二段1666号 D1栋05层1号 邮编：610100 t (86–28) 8481 0338 sgs.china@sgs.com

Member of the SGS Group (SGS SA)

编号	检测项目	单位	CAS No.	检测结果 1	定量限
523	灭蚁灵	mg/kg	2385-85-5	ND	0.01
524	#绿谷隆	mg/kg	1746-81-2	ND	0.01
525	敌草胺	mg/kg	15299-99-7	ND	0.01
526	除草醚	mg/kg	1836-75-5	ND	0.01
527	酞菌酯	mg/kg	10552-74-6	ND	0.01
528	o,p'-滴滴涕	mg/kg	789-02-6	ND	0.01
529	邻苯基苯酚（2-苯基苯酚）（OPP）	mg/kg	90-43-7	ND	0.01
530	八氯二丙醚	mg/kg	127-90-2	ND	0.01
531	p,p'-滴滴滴	mg/kg	72-54-8	ND	0.01
532	p,p'-滴滴伊	mg/kg	72-55-9	ND	0.01
533	p,p'-滴滴涕	mg/kg	50-29-3	ND	0.01
534	多效唑	mg/kg	76738-62-0	ND	0.01
535	乙基对硫磷（对硫磷）	mg/kg	56-38-2	ND	0.01
536	甲基对硫磷	mg/kg	298-00-0	ND	0.01
537	戊菌唑	mg/kg	66246-88-6	ND	0.01
538	二甲戊灵（二甲戊乐灵）	mg/kg	40487-42-1	ND	0.01
539	五氯苯胺	mg/kg	527-20-8	ND	0.01
540	五氯甲氧基苯（五氯茴香醚）	mg/kg	1825-21-4	ND	0.01
541	#戊基恶唑酮	mg/kg	110956-75-7	ND	0.01
542	氯菊酯	mg/kg	52645-53-1	ND	0.01
543	#苯醚菊酯	mg/kg	26002-80-2	ND	0.01
544	稻丰散	mg/kg	2597-03-7	ND	0.01
545	甲拌磷	mg/kg	298-02-2	ND	0.01
546	#甲拌磷砜	mg/kg	2588-04-7	ND	0.01
547	#甲拌磷亚砜	mg/kg	2588-03-6	ND	0.01
548	磷胺	mg/kg	13171-21-6	ND	0.02
549	增效醚	mg/kg	51-03-6	ND	0.01
550	#哌草磷	mg/kg	24151-93-7	ND	0.01
551	嘧啶磷（乙基嘧啶磷）	mg/kg	23505-41-1	ND	0.01
552	甲基虫螨磷（甲基嘧啶磷）	mg/kg	29232-93-7	ND	0.01
553	#丙草胺	mg/kg	51218-49-6	ND	0.01
554	腐霉利	mg/kg	32809-16-8	ND	0.01
555	丙溴磷（溴氯磷）	mg/kg	41198-08-7	ND	0.01
556	#敌稗	mg/kg	709-98-8	ND	0.01
557	#丙虫磷	mg/kg	7292-16-2	ND	0.01
558	#扑灭津	mg/kg	139-40-2	ND	0.01
559	苯胺灵	mg/kg	122-42-9	ND	0.01
560	丙环唑	mg/kg	60207-90-1	ND	0.01
561	炔苯酰草胺（拿草特）	mg/kg	23950-58-5	ND	0.01
562	丙硫磷（普硫松）	mg/kg	34643-46-4	ND	0.01

除非另有书面协议，本报告由本公司依据《服务通用条款》出具。
《服务通用条款》印刷在正本报告纸背面，或通过 https://www.sgs.com/en/Terms-and-Conditions 查询。请特别关注其中涉及责任限定，赔偿以及司法管辖的相关条款。任何报告的持有方需知悉，此报告内容仅反映SGS受限于客户指示下，且在当时所得结论。SGS仅对其客户负责，并且此报告不能免除交易各方根据交易文件所享有的权利和应履行的义务。未获得本公司书面批准，本文件不得进行复制，全文除外。任何未经授权对报告内容及形式进行的修改、伪造或扭曲都是违法行为，违法者将会被追究法律责任。除非另有声明，本测试报告所示结果仅涉及受测试样品。
注意：检测/检验报告或证书的真实性，请通过电话（86－755）83071443或邮箱 CN.Doccheck@sgs.com 查询。

No.1, 5F, Building D1, No. 1666, Section 2 of Chenglong Road, Economic and Technological Development Zone, Longquanyi District, Chengdu, Sichuan, China 610100 t (86–28) 8481 0338 www.sgsgroup.com.cn
中国・四川・成都市龙泉驿区经济技术开发区成龙大道二段1666号 D1栋05层1号 邮编：610100 t (86–28) 8481 0338 sgs.china@sgs.com

Member of the SGS Group (SGS SA)

通标标准技术服务有限公司四川分公司 检验检测专用章 Inspection & Testing Services

编号	检测项目	单位	CAS No.	检测结果 1	定量限
563	#吡唑硫磷	mg/kg	77458-01-6	ND	0.01
564	#吡草醚（霸草灵）	mg/kg	129630-19-9	ND	0.01
565	#嘧草醚	mg/kg	136191-64-5	ND	0.01
566	喹硫磷	mg/kg	13593-03-8	ND	0.01
567	五氯硝基苯	mg/kg	82-68-8	ND	0.01
568	胺丙畏（巴胺磷）（烯虫磷）	mg/kg	31218-83-4	ND	0.01
569	#蔬果磷	mg/kg	3811-49-2	ND	0.01
570	#硅醚菊酯	mg/kg	105024-66-6	ND	0.01
571	治螟磷	mg/kg	3689-24-5	ND	0.01
572	硫丙磷	mg/kg	35400-43-2	ND	0.01
573	#苯腈磷（苯腈硫磷）	mg/kg	13067-93-1	ND	0.01
574	#螺甲螨酯	mg/kg	283594-90-1	ND	0.01
575	氟胺氰菊酯	mg/kg	102851-06-9	ND	0.01
576	#丁基嘧啶磷	mg/kg	96182-53-5	ND	0.01
577	四氯硝基苯	mg/kg	117-18-0	ND	0.01
578	七氟菊酯	mg/kg	79538-32-2	ND	0.01
579	#特草定	mg/kg	5902-51-2	ND	0.01
580	特丁硫磷	mg/kg	13071-79-9	ND	0.01
581	#特丁净（特丁草净）	mg/kg	886-50-0	ND	0.01
582	#特丁津	mg/kg	5915-41-3	ND	0.01
583	杀虫畏	mg/kg	22248-79-9	ND	0.02
584	四氟醚唑（氟醚唑）	mg/kg	112281-77-3	ND	0.01
585	三氯杀螨砜	mg/kg	116-29-0	ND	0.01
586	胺菊酯	mg/kg	7696-12-0	ND	0.01
587	#甲基乙拌磷（二甲基硫吸磷）	mg/kg	640-15-3	ND	0.01
588	甲基立枯磷	mg/kg	57018-04-9	ND	0.01
589	甲苯氟磺胺	mg/kg	731-27-1	ND	0.02
590	反式氯丹	mg/kg	5103-74-2	ND	0.01
591	野麦畏	mg/kg	2303-17-5	ND	0.01
592	1,2,4-三丁基三硫磷酸酯（脱叶磷）	mg/kg	78-48-8	ND	0.01
593	氟乐灵	mg/kg	1582-09-8	ND	0.01
594	乙烯菌核利	mg/kg	50471-44-8	ND	0.01
595	（顺式-氯丹,反式-氯丹）总量	mg/kg	-	ND	-
596	七氯和环氧七氯总量	mg/kg	-	ND	-
597	滴滴涕总量（o,p'-滴滴涕,p,p'-滴滴滴,p,p'-滴滴伊,p,p'-滴滴涕）	mg/kg	-	ND	-
598	#（甲拌磷,甲拌磷砜,甲拌磷亚砜）总量	mg/kg	-	ND	-
599	硫丹总量 （α-硫丹,β-硫丹和硫丹硫酸酯之和）	mg/kg	1031-07-8 /33213-65-9 /959-98-8	ND	-

通标标准技术服务有限公司四川分公司 检验检测专用章 Inspection & Testing Services SGS-CSTC Standards Technical Services Co., Ltd. Sichuan Branch
通标标准技术服务有限公司四川分公司 农产食品实验室

除非另有书面协议，本报告由本公司依据《 服务通用条款 》出具。
《 服务通用条款 》印刷在正本报告纸背面，或通过 https://www.sgs.com/en/Terms-and-Conditions 查询。请特别关注其中涉及责任限定，赔偿以及司法管辖的相关条款。任何报告的持有方需知悉，此报告内容仅反映SGS受限于客户指示下，且在当时所得结论。SGS仅对其客户负责，并且此报告不能免除交易各方根据交易文件所享有的权利和应履行的义务。未获得本公司书面批准，本文件不得进行复制，全文除外。任何未经授权对报告内容及形式进行的修改、伪造或扭曲都是违法行为，违法者将会被追究法律责任。除非另有声明，本测试报告所示结果仅涉及受测试样品。
注意：检测/检验报告或证书的真实性，请通过电话 (86－755) 83071443 或邮箱 CN.Doccheck@sgs.com 查询。

No.1, 5/F., Building D1, No. 1666, Section 2 of Chenglong Road, Economic and Technological Development Zone, Longquanyi District, Chengdu, Sichuan, China 610100 t (86–28) 8481 0338 www.sgsgroup.com.cn
中国・四川・成都市龙泉驿区经济技术开发区成龙大道二段1666号 D1栋05层1号 邮编：610100 t (86–28) 8481 0338 sgs.china@sgs.com

Member of the SGS Group (SGS SA)

检测报告

CDF25-0001880-01 发布日期：2025-05-06

编号	检测项目	单位	CAS No.	检测结果 1	定量限
600	六六六总量（α-六六六,β-六六六,γ-六六六,δ-六六六）	mg/kg	319-84-6 /319-85-7 /319-86-8 /58-89-9	ND	-
601	#氯氰菊酯&Zeta-氯氰菊酯	mg/kg	-	ND	0.01
602	#野燕枯（硫酸二甲酯）	mg/kg	43222-48-6	ND	0.01

备注:
1.ND = 未检出
2.#检测项目不在本实验室的 CNAS 认可范围内。

注意事项：
除非另有说明，本检测结果仅与被检测物品有关。未经检验机构书面同意，委托人不得擅自使用检测结果进行不当宣传。本检测报告仅作为科研、教学或内部质量控制之用。未经检验机构批准，不得复制（全文复制除外）报告。

结束

除非另有书面协议，本报告由本公司依据《服务通用条款》出具。
《服务通用条款》印刷在正本报告纸背面，或通过 https://www.sgs.com/en/Terms-and-Conditions 查询。请特别关注其中涉及责任限定，赔偿以及司法管辖的相关条款。任何报告的持有方需知悉，此报告内容仅反映SGS受限于客户指示下，且在当时所得结论。SGS仅对其客户负责，并且此报告不能免除交易各方根据交易文件所享有的权利和应履行的义务。未获得本公司书面批准，本文件不得进行复制，全文除外。任何未经授权对报告内容及形式进行的修改、伪造或扭曲都是违法行为，违法者将会被追究法律责任。除非另有声明，本测试报告所示结果仅涉及受测试样品。
注意：检测/检验报告或证书的真实性，请通过电话 (86－755) 83071443 或邮箱 CN.Doccheck@sgs.com 查询。

No.1, 5/F., Building D1, No. 1666, Section 2 of Chenglong Road, Economic and Technological Development Zone, Longquanyi District, Chengdu, Sichuan, China 610100 t (86–28) 8481 0338 www.sgsgroup.com.cn
中国・四川・成都市龙泉驿区经济技术开发区成龙大道二段1666号 D1栋05层1号 邮编：610100 t (86–28) 8481 0338 sgs.china@sgs.com

Member of the SGS Group (SGS SA)
