# Supplementary material for: Monoglyceride-structured rubber seed oil oleogels as a partial cocoa butter replacement in chocolate: physical properties, oxidative stability and applications
Source: Food Chem X. 2026 Oct 11;39:104594. doi: 10.1016/j.fochx.2026.104594 (PMC13683454; doi:10.1016/j.fochx.2026.104594)
Supplement: Supplementary file 6 [file mmc6.pdf]

ICS 67.200.10
X 14

LS

中华人民共和国粮食行业标准

LS/T 3262—2019

食用橡胶籽油

Edible rubber seed oil

2019-06-06 发布　　　　2019-12-06 实施

国家粮食和物资储备局　发布

中 华 人 民 共 和 国 粮 食
行 业 标 准
食用橡胶籽油
LS/T 3262—2019
*
中 国 标 准 出 版 社 出 版 发 行
北京市朝阳区和平里西街甲 2 号(100029)
北京市西城区三里河北街 16 号(100045)
网址 www.spc.net.cn
总编室:(010)68533533 发行中心:(010)51780238
读者服务部:(010)68523946
中国标准出版社秦皇岛印刷厂印刷
各地新华书店经销
*
开本 880×1230 1/16 印张 0.5 字数 10 千字
2019 年 7 月第一版 2019 年 7 月第一次印刷
*
书号: 155066·2-34435 定价 14.00 元

如有印装差错 由本社发行中心调换
版权专有 侵权必究
举报电话:(010)68510107

前　言

本标准按照 GB/T 1.1—2009 给出的规则起草。

本标准由国家粮食和物资储备局提出。

本标准由全国粮油标准化技术委员会(SAC/TC 270)归口。

本标准起草单位:海南省粮油科学研究所、武汉轻工大学、国家粮食和物资储备局标准质量中心、海南侯臣生物科技有限公司、西双版纳华坤生物科技有限责任公司、云南摩尔农庄生物科技开发有限公司。

本标准主要起草人:郑联合、何东平、杨卫民、谭家安、王涛、张世宏、陈艳、赵阔、张红建、王云、朱义鑫、张跃进。

食用橡胶籽油

1 范围

本标准规定了食用橡胶籽油的术语和定义、分类、质量要求、检验方法、检验规则、标签标识以及包装、储存、运输和销售的要求。

本标准适用于食用橡胶籽原油和成品食用橡胶籽油。

食用橡胶籽原油的质量指标仅适用于原油的贸易。

2 规范性引用文件

下列文件对于本文件的应用是必不可少的。凡是注日期的引用文件，仅注日期的版本适用于本文件。凡是不注日期的引用文件，其最新版本(包括所有的修改单)适用于本文件。

GB/T 191 包装储运图示标志

GB/T 5009.37 食用植物油卫生标准的分析方法

GB/T 5009.168 食品安全国家标准 食品中脂肪酸的测定

GB 5009.229 食品安全国家标准 食品中酸价的测定

GB 5009.236 食品安全国家标准 动植物油脂水分及挥发物的测定

GB/T 5490 粮油检验 一般规则

GB/T 5524 动植物油脂 扦样

GB/T 5525 植物油脂 透明度、气味、滋味鉴定法

GB/T 5526 植物油脂检验 比重测定法

GB/T 5527 动植物油脂 折光指数的测定

GB/T 5532 动植物油脂 碘值的测定

GB/T 5534 动植物油脂 皂化值的测定

GB 7718 食品安全国家标准 预包装食品标签通则

GB/T 15688 动植物油脂 不溶性杂质含量的测定

GB/T 17374 食用植物油销售包装

GB 28050 食品安全国家标准 预包装食品营养标签通则

GB/T 30354 食用植物油散装运输规范

3 术语和定义

下列术语和定义适用于本文件。

3.1

食用橡胶籽油 edible rubber seed oil

又称食用橡胶树种子油，采用橡胶籽为原料加工制成的油脂。

3.2

食用橡胶籽原油 crude edible rubber seed oil

橡胶籽经压榨、浸出法制取的未经精炼处理，不能直接食用的橡胶籽油。

3.3

成品食用橡胶籽油　finished product of edible rubber seed oil

由食用橡胶籽原油精炼制成，可供人类食用的油脂。

4　分类

食用橡胶籽油分为食用橡胶籽原油和成品食用橡胶籽油两类。

5　质量要求

5.1　基本组成和主要物理参数

食用橡胶籽油基本组成和主要物理参数见表1。

表1　食用橡胶籽油基本组成和主要物理参数

项目		指标
折光指数(n^{40})		1.467～1.472
相对密度(d_{20}^{20})		0.919～0.926
碘值(I_2)/(g/100 g)		120～145
皂化值(KOH)/(mg/g)		175～197
主要脂肪酸组成/%	棕榈酸(C16:0)	7.8～10.6
	硬脂酸(C18:0)	6.5～9.3
	油酸(C18:1)	16.2～28.7
	亚油酸(C18:2)	33.6～42.9
	亚麻酸(C18:3)	14.0～25.0

5.2　质量指标

5.2.1　食用橡胶籽原油质量指标

食用橡胶籽原油质量指标见表2。

表2　食用橡胶籽原油质量指标

项目	质量指标
气味、滋味	具有食用橡胶籽原油固有的气味和滋味，无异味
水分及挥发物含量/%　　≤	0.50
不溶性杂质含量/%　　≤	0.50

5.2.2　成品食用橡胶籽油质量指标

成品食用橡胶籽油质量指标见表3。

表 3 成品食用橡胶籽油质量指标

项目		质量指标	
		一级	二级
气味、滋味		具有食用橡胶籽油固有的气味和滋味，无异味	
色泽		淡黄色至棕黄色	
透明度(20 ℃)		透明	允许微浊
水分及挥发物含量/%	≤	0.20	
不溶性杂质含量/%	≤	0.05	
酸价(KOH)/(mg/g)	≤	1.0	3.0

5.3 食品安全要求

按食品安全国家标准和法律法规有关规定执行。

6 检验方法

6.1 透明度、气味、滋味检验：按照 GB/T 5525 执行。
6.2 色泽检验：按照 GB/T 5009.37 执行。
6.3 相对密度检验：按照 GB/T 5526 执行。
6.4 折光指数检验：按照 GB/T 5527 执行。
6.5 水分及挥发物含量检验：按照 GB 5009.236 执行。
6.6 不溶性杂质含量检验：按照 GB/T 15688 执行。
6.7 酸价检验：按照 GB 5009.229 执行。
6.8 碘值检验：按照 GB/T 5532 执行。
6.9 皂化值检验：按照 GB/T 5534 执行。
6.10 脂肪酸组成检验：按照 GB 5009.168 执行。

7 检验规则

7.1 检验一般规则

按照 GB/T 5490 的要求执行。

7.2 扦样

按照 GB/T 5524 的要求执行。

7.3 出厂检验

7.3.1 应逐批检验，并出具检验报告。
7.3.2 按 5.2 规定的项目检验。

7.4 型式检验

7.4.1 当原料、设备、工艺有较大变化或监督部门提出要求时，均应进行型式检验。

7.4.2 按本标准第5章的规定检验。当检测结果与表1的规定不符合时，可用生产该批产品的橡胶籽原料进行检验，并佐证。

7.5 判定规则

7.5.1 产品未标明质量等级时，按不合格判定。

7.5.2 产品经检验，有一项不符合表2、表3规定值时，判定为不符合该等级的产品。

8 标签标识

8.1 预包装产品应符合 GB 7718、GB 28050 要求。

8.2 非预包装产品根据分类标注产品名称。

9 包装、储存、运输和销售

9.1 包装

9.1.1 应符合 GB/T 17374 及国家的有关规定和要求。

9.1.2 包装储运图示标志应符合 GB/T 191 的规定。

9.2 储存

应储存于卫生、阴凉、干燥及避光处，不得与有害、有毒物品一同存放，尤其要避开有异常气味的物品。

9.3 运输

运输中应注意安全，防止日晒、雨淋、渗漏、污染和标签脱落。散装运输符合 GB/T 30354 的要求。

9.4 销售

预包装的成品食用橡胶籽油在零售终端不得脱离原包装散装销售。

LS/T 3262-2019

版权专有 侵权必究

*

书号：155066 · 2-34435

定价： 14.00 元
