# Supplementary material for: Monoglyceride-structured rubber seed oil oleogels as a partial cocoa butter replacement in chocolate: physical properties, oxidative stability and applications
Source: Food Chem X. 2026 Oct 11;39:104594. doi: 10.1016/j.fochx.2026.104594 (PMC13683454; doi:10.1016/j.fochx.2026.104594)
Supplement: Supplementary file 7 [file mmc7.pdf]

Table 1. QC (Quality Control) Data-result

Sample	No 4. Oil Samplie_1	No 4. Oil Samplie_2	No 4. Oil Samplie_3	No 4. Oil Samplie_4
**C4:0 ug/ml**	0.0000	0.0000	0.0000	0.0000
**C6:0 ug/ml**	0.0000	0.0000	0.0000	0.0000
**C8:0 ug/ml**	0.0000	0.0000	0.0000	0.0000
**C10:0 ug/ml**	0.0000	0.0000	0.0000	0.0000
**C11:0 ug/ml**	0.0000	0.0000	0.0000	0.0000
**C12:0 ug/ml**	0.0382	0.1102	0.0511	0.0856
**C13:0 ug/ml**	0.0000	0.0000	0.0000	0.0000
**C14:0 ug/ml**	0.7543	1.3085	1.1756	1.1299
**C14:1 ug/ml**	0.0000	0.0000	0.0000	0.0000
**C15:0 ug/ml**	0.0571	0.0906	0.0649	0.0286
**C15:1 ug/ml**	0.0000	0.0000	0.0000	0.0000
**C16:0 ug/ml**	80.4841	144.4967	129.1287	130.0010
**C16:1 ug/ml**	2.0247	3.4542	2.8344	2.6385
**C17:0 ug/ml**	0.2805	0.5724	0.5256	0.3512
**C17:1 ug/ml**	0.0000	0.0000	0.0000	0.0000
**C18:0 ug/ml**	57.1049	104.3571	104.7075	91.0578
**C18:1n9t ug/ml**	0.0000	0.0000	0.0000	0.0000
**C18:1n9c ug/ml**	269.5299	367.2588	349.0073	359.4810
**C18:2n6t ug/ml**	0.0000	0.0000	0.0000	0.0000
**C18:2n6c ug/ml**	425.9187	716.5888	605.5238	628.4496
**C20:0 ug/ml**	1.6464	3.2220	2.9614	2.3497
**C18:3n6 ug/ml**	1.1241	1.3532	1.3794	1.2246
**C20:1 ug/ml**	1.1030	2.0308	1.0418	1.9456
**C18:3n3 ug/ml**	232.3224	376.3816	313.2492	356.2991
**C21:0 ug/ml**	0.0000	0.0000	0.0000	0.0000
**C20:2 ug/ml**	0.0000	0.0000	0.0000	0.0000
**C22:0 ug/ml**	0.2995	0.6382	0.6679	0.5049

Sample	No 4. Oil Samplie_1	No 4. Oil Samplie_2	No 4. Oil Samplie_3	No 4. Oil Samplie_4
**C20:3n6 ug/ml**	0.0000	0.0000	0.0000	0.0000
**C22:1n9 ug/ml**	0.0000	0.0000	0.0000	0.0000
**C20:3n3 ug/ml**	0.0000	0.0000	0.0000	0.0000
**C23:0 ug/ml**	0.0784	0.2143	0.1715	0.1885
**C20:4n6 ug/ml**	0.0000	0.0000	0.0000	0.0000
**C22:2 ug/ml**	0.0000	0.0000	0.0000	0.0000
**C24:0 ug/ml**	0.1180	0.3914	0.3388	0.2576
**C20:5n3 ug/ml**	0.0000	0.0000	0.0000	0.0000
**C24:1 ug/ml**	0.0000	0.0000	0.0000	0.0000
**C22:6n3 ug/ml**	0.0000	0.0000	0.0000	0.0000

Table 2. QC RSD Results

Code Name	QC_01	QC_02	QC_03	Mean	SD	RSD
C4:0	31.1932	32.8596	35.4658	33.1729	2.1535	6.4916%
C6:0	30.4528	32.8231	34.9566	32.7442	2.2529	6.8804%
C8:0	28.5460	31.4605	33.1529	31.0531	2.3303	7.5043%
C10:0	29.8238	32.6425	34.8635	32.4433	2.5258	7.7851%
C11:0	30.1032	33.7423	35.9176	33.2544	2.9377	8.8342%
C12:0	31.0199	33.2380	36.1910	33.4830	2.5942	7.7479%
C13:0	30.8234	33.6745	36.0750	33.5243	2.6290	7.8421%
C14:0	31.8268	34.0015	37.1998	34.3427	2.7027	7.8698%
C14:1	31.7425	33.4269	36.1676	33.7790	2.2335	6.6120%
C15:0	32.4515	34.2758	38.0093	34.9122	2.8330	8.1147%
C15:1	30.4369	32.2964	35.6567	32.7967	2.6456	8.0667%
C16:0	65.8427	70.3362	77.5170	71.2320	5.8885	8.2666%
C16:1	31.0544	33.2167	36.3084	33.5265	2.6407	7.8764%
C17:0	30.1531	32.2517	36.5373	32.9807	3.2539	9.8662%
C17:1	30.3375	31.3192	36.8417	32.8328	3.5063	10.6794%
C18:0	29.2214	31.9233	35.6806	32.2751	3.2439	10.0509%
C18:1n9t	28.0530	29.9346	33.7663	30.5846	2.9116	9.5198%
C18:1n9c	28.7367	30.2719	33.2443	30.7510	2.2917	7.4523%
C18:2n6t	26.9695	29.1560	31.8423	29.3226	2.4407	8.3235%
C18:2n6c	27.7304	29.8418	32.9192	30.1638	2.6093	8.6506%
C20:0	26.4200	28.8071	32.1867	29.1379	2.8975	9.9443%
C18:3n6	27.1904	28.9142	32.9106	29.6717	2.9344	9.8895%
C20:1	26.1770	28.3847	31.8741	28.8119	2.8725	9.9698%
C18:3n3	28.1849	28.0498	32.4173	29.5507	2.4835	8.4042%
C21:0	26.9483	29.4130	33.0062	29.7892	3.0464	10.2266%
C20:2	26.9070	28.5397	31.2887	28.9118	2.2144	7.6592%
C22:0	27.7634	30.3452	33.9072	30.6719	3.0849	10.0577%
C20:3n6	26.4342	28.6191	31.0015	28.6849	2.2844	7.9636%

Code Name	QC_01	QC_02	QC_03	Mean	SD	RSD
C22:1n9	25.3839	29.1962	30.9482	28.5094	2.8450	9.9792%
C20:3n3	26.2123	28.5433	31.7134	28.8230	2.7612	9.5798%
C23:0	28.2853	31.6676	35.3555	31.7695	3.5362	11.1308%
C20:4n6	27.3605	30.3757	32.7225	30.1529	2.6879	8.9143%
C22:2	26.3144	28.4143	31.8138	28.8475	2.7752	9.6202%
C24:0	28.5624	31.6110	35.1178	31.7637	3.2804	10.3274%
C20:5n3	25.7786	28.2126	32.0351	28.6754	3.1538	10.9983%
C24:1	25.6073	28.6283	31.0824	28.4393	2.7424	9.6431%
C22:6n3	23.9350	25.7230	29.0477	26.2352	2.5946	9.8896%

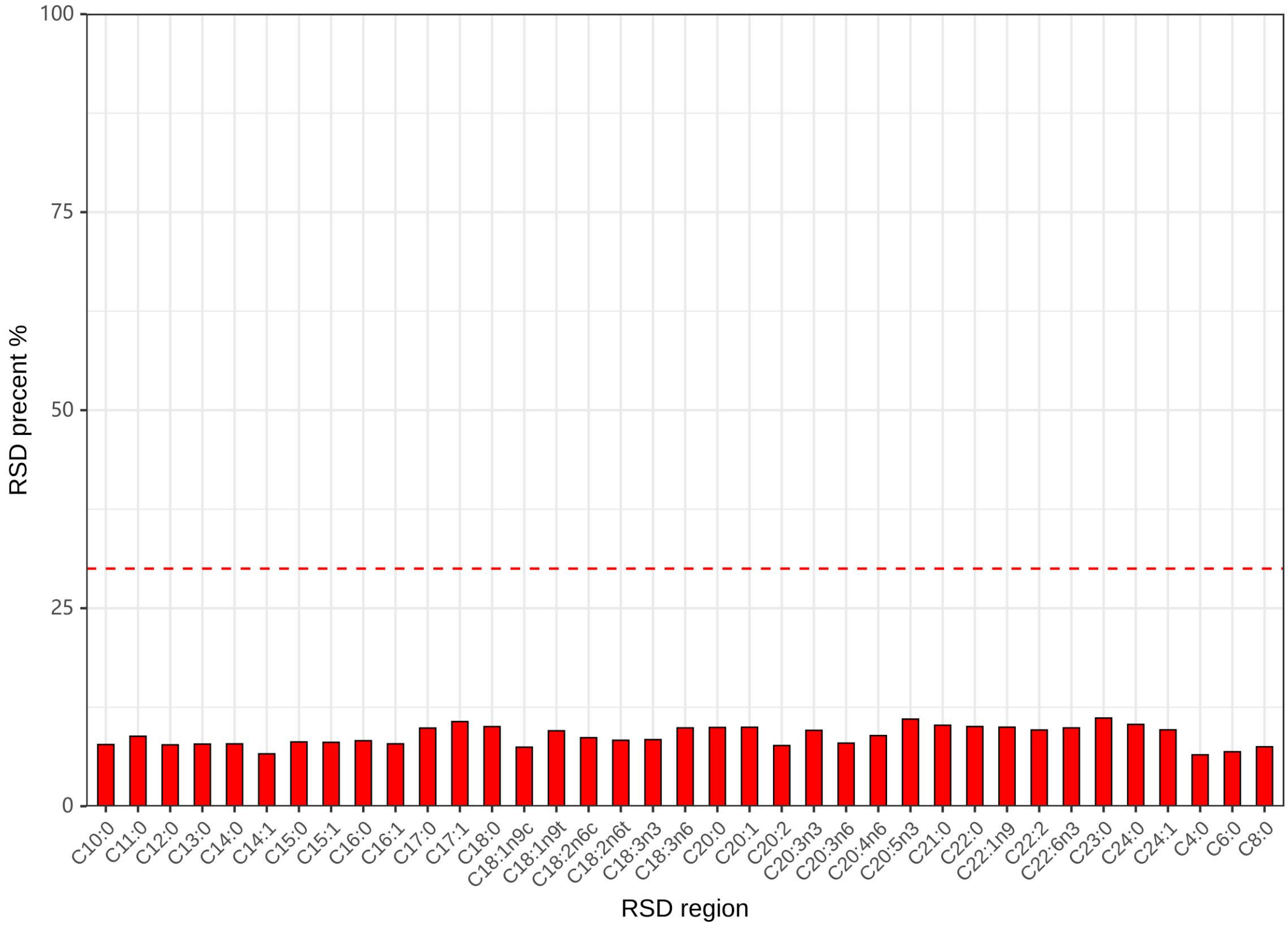

Figure 1. QC RSD graph

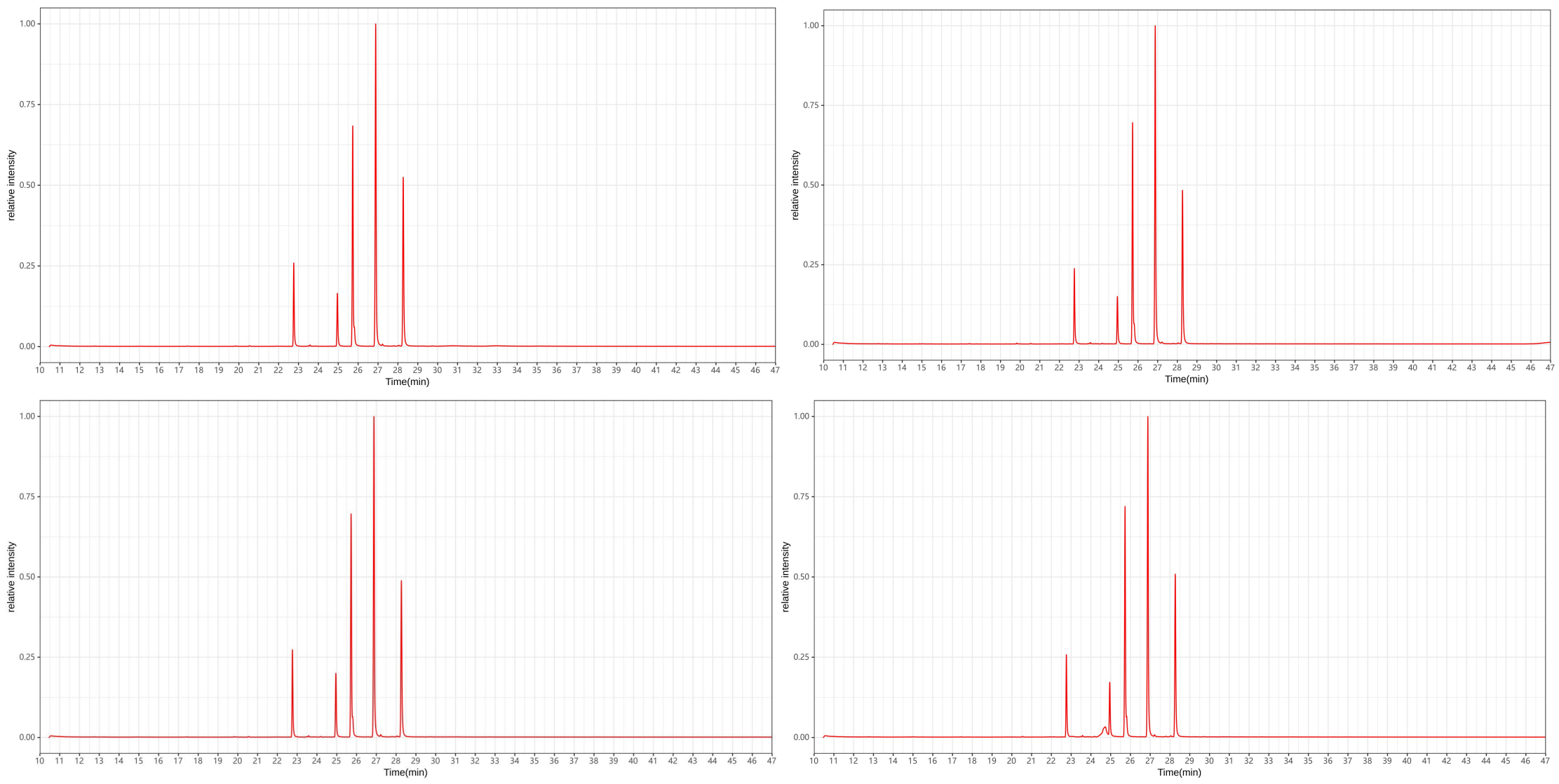

Figure 2. TIC (Total Ion Chromatogram) Result of Oil Samples (No 4. Oil Samplie_1: upper left. No 4. Oil Samplie_2: upper right. No 4. Oil Samplie_3: lower left. No 4. Oil Samplie_4: lower right)

Table 3. Samples Results STD

Retention time (minutes)	Indensity	Percentage of maximum peak area
10.41948333	125.7189789	4.21549E-07
10.42338333	985.2672119	3.3037E-06
10.42728333	787.4244385	2.64032E-06
10.43118333	402.2883301	1.34891E-06
10.43508333	131.5483856	4.41095E-07
10.4429	142.0788879	4.76405E-07
10.4468	557.9350204	1.87081E-06
10.4507	2867.940704	9.6165E-06
10.4546	8539.484528	2.86338E-05
10.4585	22804.793	7.64668E-05
10.4624	54376.58675	0.00018233
10.4663	113363.4677	0.000380119
10.47021667	220186.9909	0.00073831
10.47411667	375563.5301	0.001259303
10.47801667	569508.9713	0.001909623
10.48191667	783177.8079	0.002626076
10.48581667	964155.6768	0.003232914
10.48971667	1158724.662	0.003885324
10.4937	1411089.268	0.004731529
10.49765	1775677.038	0.005954029
10.50155	2220018.458	0.007443952
10.50545	2778178.15	0.009315519
10.50936667	3464931.296	0.011618274
10.51326667	4318772.836	0.014481293
10.51716667	5302824.636	0.017780921
10.52108333	6376911.728	0.021382446
10.52498333	7553245.492	0.025326816
10.5289	8740590.633	0.029308107
10.53281667	9900690.861	0.033198043
10.53671667	10953688.65	0.036728854
10.54061667	11859161.57	0.039764999
10.54453333	12585808.47	0.042201521
10.54843333	13164191.54	0.0441409
10.55235	13495835.39	0.045252936
10.55625	13612795.7	0.045645116
10.56016667	13504245.53	0.045281136
10.56406667	13237980.09	0.04438832
10.56798333	12800104.53	0.042920078
10.5719	12267259.19	0.041133392
10.5758	11690306.48	0.03919881
10.57971667	10985028.16	0.036833939
10.58361667	10294675.26	0.034519114
10.58753333	9608166.531	0.032217179
10.59143333	8862117.534	0.029715599
10.59533333	8123586.014	0.027239226
10.59925	7448655.715	0.024976115
10.60316667	6849103.948	0.022965756
10.60706667	6321826.219	0.021197739
10.61098333	5830424.181	0.019550017
10.61488333	5389272.094	0.018070788
10.6188	4926273.632	0.016518306
10.6227	4563480.953	0.015301825
10.62661667	4231499.396	0.014188656
10.63051667	3931414.142	0.013182439
10.63443333	3698896.877	0.012402785
10.63835	3496429.386	0.01172389
10.64225	3321756.299	0.011138193
10.64616667	3179114.511	0.010659901
10.65006667	3050563.972	0.010228857
10.65398333	2942837.77	0.00986764
10.65788333	2845268.894	0.009540482
10.6618	2745482.764	0.009205888
10.6657	2673289.63	0.008963817
10.6696	2642469.793	0.008860475
10.67351667	2514261.234	0.008430579
10.67741667	2463786.993	0.008261333
10.68133333	2448177.627	0.008208994
10.68525	2422488.079	0.008122854
10.68916667	2379667.75	0.007979273
10.69306667	2328772.423	0.007808615
10.69698333	2285037.948	0.007661969
10.70088333	2271515.925	0.007616628
10.7048	2272849.033	0.007621099
10.7087	2250841.813	0.007547306

Table 3. Samples Results STD

Retention time (minutes)	Indensity	Percentage of maximum peak area
10.7126	2252460.022	0.007552732
10.71651667	2258136.622	0.007571766
10.72041667	2261304.703	0.007582389
10.72433333	2267076.668	0.007601743
10.72825	2312988.154	0.007755689
10.73215	2325446.902	0.007797465
10.73606667	2313107.719	0.00775609
10.73996667	2296735.786	0.007701193
10.74388333	2258062.535	0.007571518
10.74778333	2228746.941	0.00747322
10.7517	2248857.847	0.007540654
10.7556	2244233.613	0.007525148
10.75951667	2253388.47	0.007555845
10.76341667	2234905.83	0.007493871
10.76733333	2211858.907	0.007416592
10.77123333	2136030.568	0.007162332
10.77513333	2087622.417	0.007000014
10.77905	2043608.893	0.006852433
10.78295	2036650.212	0.006829099
10.78686667	2016870.449	0.006762776
10.79076667	2012534.511	0.006748237
10.79468333	2023444.957	0.006784821
10.79858333	2016692.035	0.006762178
10.8025	2008725.939	0.006735466
10.80641667	1993498.79	0.006684408
10.81031667	1993240.584	0.006683542
10.81421667	1982616.239	0.006647918
10.81813333	1971521.385	0.006610716
10.82203333	1942636.091	0.00651386
10.82595	1925960.935	0.006457947
10.82985	1926262.891	0.006458959
10.83376667	1930707.84	0.006473864
10.83766667	1907212.817	0.006395082
10.84158333	1892667.058	0.006346309
10.84548333	1880566.963	0.006305736
10.8494	1880940.818	0.00630699
10.8533	1861780.783	0.006242744
10.85721667	1859519.179	0.006235161
10.86111667	1843115.74	0.006180158
10.86503333	1780578.117	0.005970463
10.86893333	1759447.073	0.005899609
10.87285	1754052.048	0.005881518
10.87675	1759240.521	0.005898916
10.88066667	1763662.111	0.005913742
10.88456667	1802361.106	0.006043504
10.88846667	1812105.483	0.006076178
10.89238333	1806570.815	0.006057619
10.89628333	1789885.508	0.006001672
10.9002	1774489.419	0.005950047
10.9041	1741990.899	0.005841076
10.90801667	1735147.288	0.005818129
10.91191667	1728935.6	0.0057973
10.91583333	1726541.509	0.005789273
10.91973333	1709335.504	0.005731579
10.92365	1690528.895	0.005668519
10.92755	1710057.147	0.005733999
10.93146667	1737392.364	0.005825657
10.93536667	1756494.577	0.005889708
10.93928333	1737134.891	0.005824793
10.94318333	1724164.292	0.005781302
10.9471	1718304.559	0.005761653
10.951	1726142.443	0.005787935
10.95491667	1727971.773	0.005794069
10.95881667	1691159.817	0.005670634
10.96273333	1676286.193	0.005620761
10.96663333	1653843.074	0.005545507
10.97055	1640012.462	0.005499132
10.97445	1607090.237	0.00538874
10.97836667	1627276.583	0.005456427
10.98226667	1671857.643	0.005605912
10.98618333	1717426.696	0.00575871
10.99008333	1775491.254	0.005953406
10.99398333	1848006.499	0.006196557
10.9979	1942609.428	0.006513771

Table 3. Samples Results STD

Retention time (minutes)	Indensity	Percentage of maximum peak area
11.0018	2050824.15	0.006876626
11.00571667	2140780.563	0.007178259
11.00961667	2243681.866	0.007523298
11.01353333	2311059.896	0.007749224
11.01743333	2396420.053	0.008035445
11.02135	2451450.843	0.008219969
11.02525	2395824.462	0.008033448
11.02916667	2392750.336	0.00802314
11.03306667	2362192.519	0.007920677
11.03698333	2267251.435	0.007602329
11.04088333	2184790.876	0.00732583
11.0448	2086043.502	0.00699472
11.0487	1999455.747	0.006704382
11.05261667	1890079.902	0.006337634
11.05651667	1831069.524	0.006139766
11.06041667	1764542.205	0.005916693
11.06433333	1702317.653	0.005708048
11.06823333	1664874.668	0.005582497
11.07215	1629264.668	0.005463093
11.07605	1591257.606	0.005335652
11.07996667	1569452.14	0.005262536
11.08386667	1554953.262	0.00521392
11.08778333	1548250.913	0.005191446
11.09168333	1566828.971	0.00525374
11.0956	1557609.245	0.005222825
11.0995	1533409.71	0.005141682
11.10341667	1469352.393	0.004926891
11.10731667	1439062.467	0.004825326
11.11123333	1421642.075	0.004766913
11.11513333	1419260.5	0.004758928
11.11905	1432307.274	0.004802675
11.12295	1409264.522	0.00472541
11.12686667	1381395.203	0.004631961
11.13076667	1378736.04	0.004623045
11.13468333	1367571.957	0.004585611
11.13858333	1358904.33	0.004556547
11.1425	1333093.781	0.004470002
11.1464	1338932.297	0.004489579
11.1503	1360180.595	0.004560827
11.15421667	1393638.88	0.004673016
11.15811667	1448890.117	0.004858279
11.16203333	1508151.221	0.005056987
11.16593333	1596571.207	0.005353469
11.16985	1629606.735	0.00546424
11.17375	1653057.663	0.005542874
11.17766667	1652379.208	0.005540599
11.18156667	1642377.893	0.005507063
11.18548333	1626136.553	0.005452605
11.18938333	1624993.322	0.005448771
11.1933	1601754.665	0.00537085
11.1972	1569084.384	0.005261303
11.20111667	1539035.176	0.005160545
11.20501667	1506103.703	0.005050122
11.20893333	1492634.23	0.005004957
11.21283333	1453519.367	0.004873801
11.21675	1417316.611	0.00475241
11.22065	1382129.111	0.004634422
11.22456667	1345502.732	0.00451161
11.22846667	1323656.798	0.004438359
11.23238333	1299223.196	0.00435643
11.23628333	1267382.701	0.004249666
11.2402	1234608.043	0.004139769
11.2441	1231930.351	0.00413079
11.24801667	1206015.86	0.004043896
11.25191667	1184987.637	0.003973386
11.25583333	1165309.821	0.003907405
11.25973333	1152473.574	0.003864363
11.26365	1174795.421	0.003939211
11.26755	1187256.615	0.003980995
11.27146667	1172409.676	0.003931211
11.27536667	1143295.112	0.003833587
11.27928333	1119959.013	0.003755339
11.28318333	1116637.95	0.003744203
11.2871	1130065.048	0.003789225

Table 3. Samples Results STD

Retention time (minutes)	Indensity	Percentage of maximum peak area
11.29101667	1141465.601	0.003827453
11.29491667	1137099.503	0.003812813
11.29883333	1135075.571	0.003806026
11.30273333	1147191.702	0.003846653
11.30665	1146840.189	0.003845474
11.31055	1147046.448	0.003846166
11.31446667	1130521.52	0.003790756
11.31836667	1107846.301	0.003714724
11.32228333	1084372.77	0.003636014
11.32618333	1069639.882	0.003586613
11.3301	1086873.961	0.003644401
11.334	1116323.972	0.00374315
11.33791667	1161173.185	0.003893534
11.34181667	1196153.635	0.004010827
11.34573333	1223432.791	0.004102297
11.34963333	1271269.878	0.0042627
11.35355	1321718.774	0.00443186
11.35745	1353721.662	0.004539169
11.36136667	1414377.298	0.004742554
11.36526667	1471026.259	0.004932504
11.36918333	1542015.378	0.005170537
11.37308333	1570466.409	0.005265937
11.377	1602799.436	0.005374353
11.3809	1622729.126	0.005441179
11.38481667	1619168.813	0.005429241
11.38871667	1588682.288	0.005327016
11.39261667	1553451.488	0.005208884
11.39653333	1531651.223	0.005135785
11.40043333	1496903.094	0.005019271
11.40435	1449964.528	0.004861881
11.40826667	1383008.64	0.004637371
11.41216667	1315465.03	0.004410891
11.41606667	1278568.775	0.004287174
11.41998333	1276293.015	0.004279543
11.42388333	1259762.622	0.004224115
11.4278	1203124.476	0.004034201
11.4317	1170364.122	0.003924352
11.43561667	1154831.761	0.003872271
11.43951667	1144704.205	0.003838312
11.44343333	1142588.861	0.003831219
11.44733333	1143182.543	0.00383321
11.45125	1154292.677	0.003870463
11.45515	1128767.367	0.003784874
11.45906667	1111269.756	0.003726203
11.46296667	1115034.814	0.003738827
11.46686667	1136578.908	0.003811067
11.47078333	1128867.328	0.003785209
11.47468333	1126309.062	0.003776631
11.4786	1099144.027	0.003685544
11.4825	1073568.422	0.003599786
11.48641667	1068281.771	0.00358206
11.49031667	1078190.922	0.003615286
11.49423333	1046418.732	0.003508751
11.49813333	1032650.93	0.003462586
11.50205	1034852.563	0.003469968
11.50595	1062814.585	0.003563728
11.50986667	1063414.831	0.00356574
11.51376667	1067688.714	0.003580071
11.51768333	1054507.624	0.003535873
11.52158333	1043114.502	0.003497671
11.52548333	1039038.219	0.003484003
11.5294	1064121.376	0.003568109
11.5333	1052481.091	0.003529078
11.53721667	1065667.555	0.003573294
11.54111667	1091288.474	0.003659203
11.54503333	1092344.342	0.003662744
11.54893333	1119000.203	0.003752124
11.55285	1134502.621	0.003804105
11.55675	1099010.167	0.003685095
11.56066667	1097145.01	0.003678841
11.56456667	1079765.494	0.003620566
11.56848333	1062504.758	0.003562689
11.57238333	1056515.089	0.003542605
11.5763	1045172.786	0.003504573

Table 3. Samples Results STD

Retention time (minutes)	Indensity	Percentage of maximum peak area
11.5802	1008115.992	0.003380317
11.58411667	1002604.764	0.003361838
11.58801667	980757.2958	0.003288581
11.59193333	962340.6944	0.003226828
11.59583333	956092.2941	0.003205877
11.59975	981599.6169	0.003291405
11.60366667	1009413.126	0.003384667
11.60756667	1007612.037	0.003378628
11.61148333	1008816.341	0.003382666
11.61538333	1011617.086	0.003392057
11.6193	1000398.887	0.003354441
11.6232	1008579.188	0.003381871
11.62711667	1004177.958	0.003367113
11.63101667	984999.2729	0.003302805
11.63493333	993892.9887	0.003332626
11.63883333	991886.0758	0.003325897
11.64275	1003076.99	0.003363421
11.64665	1013050.366	0.003396863
11.65056667	1001198.157	0.003357121
11.65446667	966081.1954	0.00323937
11.65838333	945788.0629	0.003171325
11.66228333	934698.9694	0.003134143
11.6663	939453.4751	0.003150085
11.6702	951913.9697	0.003191866
11.67411667	950294.6407	0.003186436
11.67801667	958178.3717	0.003212871
11.68193333	949173.1044	0.003182676
11.68583333	939012.7088	0.003148607
11.68975	927562.05	0.003110212
11.69365	951984.4557	0.003192103
11.69756667	948241.2693	0.003179551
11.70146667	941501.6143	0.003156953
11.70536667	952435.1181	0.003193614
11.70928333	946859.9513	0.00317492
11.71318333	949860.3043	0.00318498
11.7171	947971.7087	0.003178647
11.721	937580.3966	0.003143804
11.72491667	918749.3925	0.003080662
11.72883333	913069.0705	0.003061615
11.73273333	881447.8505	0.002955586
11.73665	875614.1212	0.002936025
11.74055	886965.9971	0.002974089
11.74445	894820.9383	0.003000427
11.74836667	897224.801	0.003008488
11.75226667	918553.4259	0.003080005
11.75618333	911644.5829	0.003056839
11.76008333	893651.0931	0.002996505
11.764	903952.413	0.003031046
11.7679	950292.4691	0.003186429
11.77181667	966961.9572	0.003242324
11.77571667	972131.1841	0.003259657
11.77961667	962324.8646	0.003226775
11.78353333	966634.2408	0.003241225
11.78743333	956904.6561	0.003208601
11.79135	966238.3736	0.003239897
11.79525	963746.8301	0.003231543
11.79923333	952097.7295	0.003192482
11.80313333	930530.6172	0.003120166
11.80705	931009.4434	0.003121771
11.81095	922979.5271	0.003094846
11.81485	912521.9279	0.003059781
11.81876667	898942.4194	0.003014247
11.82268333	934595.219	0.003133795
11.82658333	939540.4966	0.003150377
11.83048333	953748.4078	0.003198017
11.8344	958148.2041	0.00321277
11.8383	950889.9823	0.003188433
11.84221667	1010276.582	0.003387562
11.84613333	1028582.615	0.003448944
11.85003333	1065553.411	0.003572911
11.85395	1118439.163	0.003750243
11.85785	1248746.674	0.004187177
11.86176667	1426307.736	0.004782558
11.86566667	1754053.907	0.005881525

Table 3. Samples Results STD

Retention time (minutes)	Indensity	Percentage of maximum peak area
11.86956667	2288913.796	0.007674965
11.87348333	3164285.364	0.010610177
11.87738333	4491829.548	0.01506157
11.8813	6343950.374	0.021271924
11.8852	8795564.915	0.029492441
11.88911667	11895810.63	0.039887887
11.89301667	15634398.71	0.05242376
11.89693333	19865486.64	0.066611037
11.90083333	24297320.65	0.081471436
11.90475	28568561.91	0.095793351
11.90865	32472788.37	0.108884627
11.91256667	35744304.5	0.11985436
11.91646667	38147003.73	0.12791086
11.92038333	39449130.78	0.132277027
11.92428333	39537933.63	0.132574792
11.9282	38481402.97	0.129032135
11.9321	36503343.97	0.122399498
11.93601667	33899103.05	0.113667208
11.93991667	30823134.8	0.103353168
11.94383333	27518786.96	0.092273347
11.94773333	24138162.25	0.080937762
11.95165	20890021.36	0.070046408
11.95555	17868191.42	0.059913899
11.95946667	15211477.39	0.051005661
11.96336667	12927639.66	0.043347717
11.96726667	10891153.07	0.036519166
11.97118333	9169958.112	0.030747821
11.97508333	7769671.912	0.026052516
11.979	6666688.964	0.0223541
11.9829	5759137.308	0.019310985
11.98681667	5009027.743	0.01679579
11.99071667	4327597.397	0.014510883
11.99463333	3765320.851	0.012625511
11.99853333	3327777.871	0.011158384
12.00245	3009487.395	0.010091123
12.00635	2789812.08	0.009354529
12.01026667	2569543.104	0.008615945
12.01416667	2398613.636	0.0080428
12.01806667	2250371.653	0.00754573
12.02198333	2126122.53	0.007129109
12.0259	2028428.718	0.006801532
12.0298	1939465.393	0.006503229
12.03371667	1856273.462	0.006224277
12.03761667	1769287.478	0.005932604
12.04153333	1705356.467	0.005718237
12.04543333	1658211.428	0.005560155
12.04935	1610094.219	0.005398813
12.05325	1571460.92	0.005269271
12.05716667	1551734.695	0.005203127
12.06106667	1496469.708	0.005017818
12.06496667	1446727.143	0.004851026
12.06888333	1455445.459	0.00488026
12.07278333	1412736.176	0.004737051
12.0767	1400190.132	0.004694983
12.0806	1411426.774	0.00473266
12.08451667	1435043.399	0.004811849
12.08841667	1469973.221	0.004928973
12.09233333	1463601.812	0.004907609
12.09623333	1489873.796	0.004995701
12.10015	1451350.385	0.004866528
12.10405	1412416.16	0.004735978
12.10796667	1388233.001	0.004654889
12.11186667	1369993.982	0.004593732
12.11578333	1356114.939	0.004547194
12.11968333	1320338.288	0.004427231
12.1236	1263487.007	0.004236603
12.12751667	1226533.326	0.004112693
12.13141667	1199599.783	0.004022382
12.13531667	1180851.133	0.003959516
12.13923333	1124810.813	0.003771607
12.14315	1118260.85	0.003749645
12.14705	1127297.781	0.003779946
12.15096667	1135275.07	0.003806695
12.15486667	1107502.841	0.003713572

Table 3. Samples Results STD

Retention time (minutes)	Indensity	Percentage of maximum peak area
12.15878333	1082507.606	0.00362976
12.16268333	1055273.24	0.003538441
12.1666	1039776.791	0.003486479
12.1705	1025846.622	0.00343977
12.17441667	1031407.458	0.003458416
12.17831667	1001361.877	0.00335767
12.18221667	1009596.036	0.00338528
12.18613333	1005205.245	0.003370557
12.19003333	995306.8376	0.003337367
12.19395	1000902.203	0.003356129
12.19785	1028251.283	0.003447833
12.20176667	1024533.34	0.003435367
12.20568333	1014747.877	0.003402555
12.20958333	1022125.193	0.003427292
12.2135	1007000.969	0.003376579
12.2174	1004878.169	0.003369461
12.22131667	1007488.74	0.003378214
12.22521667	1016043.938	0.003406901
12.22911667	1020720.701	0.003422582
12.23303333	1012052.946	0.003393518
12.23693333	1003353.185	0.003364347
12.24085	968147.2076	0.003246298
12.24475	939125.4172	0.003148985
12.24866667	923776.5851	0.003097519
12.25256667	913104.1581	0.003061733
12.25648333	917565.0908	0.003076691
12.26038333	917124.7336	0.003075214
12.2643	919922.3944	0.003084595
12.2682	929468.1124	0.003116603
12.27211667	919312.4234	0.00308255
12.27601667	922497.4327	0.00309323
12.27993333	914398.0386	0.003066071
12.28383333	933813.8826	0.003131175
12.28775	934541.3	0.003133614
12.29165	925374.5822	0.003102877
12.29556667	929585.2491	0.003116996
12.29946667	943062.0008	0.003162185
12.30338333	949023.7934	0.003182175
12.30728333	932711.9694	0.00312748
12.3112	928883.319	0.003114642
12.3151	945779.1032	0.003171295
12.31901667	945686.1945	0.003170984
12.32291667	943821.7488	0.003164732
12.32683333	953073.396	0.003195754
12.33073333	959038.3443	0.003215755
12.33465	948564.3717	0.003180635
12.33855	945108.5719	0.003169047
12.34246667	919103.5323	0.003081849
12.34636667	884752.8823	0.002966668
12.35026667	878961.5472	0.002947249
12.35418333	923145.8526	0.003095404
12.35808333	933265.0975	0.003129335
12.362	900731.8154	0.003020247
12.3659	895770.0197	0.00300361
12.36981667	874513.4144	0.002932334
12.37371667	892158.0941	0.002991499
12.37763333	922562.5371	0.003093448
12.38153333	975984.9621	0.003272579
12.38545	977152.0555	0.003276492
12.38935	972680.5398	0.003261499
12.39326667	1006151.909	0.003373732
12.39716667	1044775.34	0.00350324
12.40106667	1073443.962	0.003599369
12.40498333	1045457.784	0.003505528
12.40888333	1055516.129	0.003539255
12.4128	1021167.804	0.003424082
12.4167	1004876.672	0.003369456
12.42061667	985870.6966	0.003305727
12.42451667	965758.5816	0.003238289
12.42843333	937594.9219	0.003143853
12.43233333	899919.3363	0.003017523
12.43625	868156.364	0.002911018
12.44015	867634.6121	0.002909269
12.44406667	870650.5193	0.002919381

Table 3. Samples Results STD

Retention time (minutes)	Indensity	Percentage of maximum peak area
12.44796667	868709.477	0.002912873
12.45188333	866778.3799	0.002906398
12.45578333	864889.986	0.002900066
12.4597	866305.693	0.002904813
12.4636	875588.9421	0.002935941
12.46751667	895739.8244	0.003003509
12.47141667	893427.8618	0.002995756
12.47533333	900061.6395	0.003018
12.47923333	914032.2442	0.003064845
12.48315	907070.634	0.003041502
12.48705	872481.5951	0.002925521
12.49096667	858247.9973	0.002877795
12.49486667	847505.3396	0.002841773
12.49878333	837511.4026	0.002808263
12.50268333	850650.5357	0.002852319
12.50658333	865523.1436	0.002902189
12.5105	873628.0215	0.002929365
12.5144	877854.6069	0.002943538
12.51831667	897137.6983	0.003008196
12.52221667	927171.6127	0.003108903
12.52613333	949749.0674	0.003184607
12.53003333	979533.5282	0.003284478
12.53395	987762.2834	0.003312069
12.53785	1001134.481	0.003356908
12.54176667	1011956.722	0.003393196
12.54566667	1000731.809	0.003355558
12.54958333	998766.1001	0.003348966
12.55348333	982064.668	0.003292965
12.5574	963744.1566	0.003231534
12.5613	954900.9808	0.003201882
12.56521667	924713.1804	0.003100659
12.56911667	906694.586	0.003040241
12.57301667	905735.6764	0.003037026
12.57693333	909623.9553	0.003050063
12.58083333	913778.1306	0.003063993
12.58475	931724.5668	0.003124169
12.58865	925654.1483	0.003103814
12.59256667	950073.9681	0.003185697
12.59646667	911290.3401	0.003055651
12.60038333	924179.8616	0.003098871
12.60428333	915081.1865	0.003068362
12.6082	920143.6478	0.003085337
12.6121	925552.49	0.003103473
12.61601667	952628.5288	0.003194262
12.61991667	967875.3007	0.003245386
12.62381667	990387.2091	0.003320871
12.62773333	1005874.198	0.003372801
12.63163333	1029788.705	0.003452988
12.63555	1041014.116	0.003490628
12.63945	1046631.508	0.003509464
12.64336667	1048505.213	0.003515747
12.64726667	1062614.027	0.003563055
12.65118333	1029432.524	0.003451794
12.65508333	998422.403	0.003347814
12.659	1002974.122	0.003363076
12.6629	1015685.82	0.0034057
12.66681667	977036.6598	0.003276105
12.67071667	926800.2028	0.003107657
12.67463333	927084.4945	0.00310861
12.67853333	949122.7342	0.003182507
12.68243333	923879.9138	0.003097865
12.68635	910376.4436	0.003052587
12.69025	880607.4096	0.002952768
12.69416667	878047.217	0.002944183
12.69806667	870953.2807	0.002920397
12.70198333	874882.5109	0.002933572
12.70588333	872082.3362	0.002924183
12.7098	853219.118	0.002860932
12.7137	848726.7145	0.002845869
12.71761667	840096.6202	0.002816931
12.72151667	845365.9302	0.0028346
12.72543333	839750.0144	0.002815769
12.72933333	844568.5761	0.002831926
12.73325	859615.9349	0.002882381

Table 3. Samples Results STD

Retention time (minutes)	Indensity	Percentage of maximum peak area
12.73715	936878.1161	0.003141449
12.74106667	981283.4386	0.003290345
12.74496667	1067944.582	0.003580929
12.74888333	1155624.955	0.00387493
12.75278333	1238486.133	0.004152772
12.7567	1342526.993	0.004501632
12.7606	1432526.131	0.004803409
12.76451667	1493420.45	0.005007594
12.76841667	1579287.318	0.005295514
12.77233333	1586434.941	0.005319481
12.77623333	1614213.113	0.005412624
12.78015	1604480.667	0.00537999
12.78405	1553133.708	0.005207818
12.78796667	1475479.571	0.004947436
12.79186667	1455057.013	0.004878957
12.79576667	1368111.684	0.00458742
12.79968333	1259604.944	0.004223586
12.80358333	1192331.849	0.003998012
12.8075	1200388.675	0.004025028
12.81141667	1185208.819	0.003974128
12.81531667	1158405.912	0.003884255
12.81923333	1170978.515	0.003926412
12.82313333	1166078.677	0.003909983
12.82705	1166595.656	0.003911716
12.83095	1174191.267	0.003937185
12.83486667	1136045.631	0.003809279
12.83876667	1107350.311	0.00371306
12.84266667	1107794.83	0.003714551
12.84658333	1097122.321	0.003678765
12.8505	1095314.901	0.003672704
12.8544	1056124.12	0.003541294
12.85831667	1030684.43	0.003455992
12.86221667	1004843.274	0.003369344
12.86613333	1001525.428	0.003358219
12.87003333	1019810.802	0.003419531
12.87395	1004409.193	0.003367888
12.87785	972964.8647	0.003262452
12.88176667	946857.0206	0.00317491
12.88566667	927002.2789	0.003108335
12.88958333	931418.216	0.003123142
12.89348333	930781.4859	0.003121007
12.89738333	934769.0164	0.003134377
12.9013	955446.6071	0.003203712
12.9052	1004797.841	0.003369191
12.90911667	1060481.32	0.003555904
12.91301667	1139995.606	0.003822524
12.91693333	1207068.316	0.004047425
12.92083333	1224503.738	0.004105888
12.92475	1209099.452	0.004054236
12.92865	1198215.906	0.004017742
12.93256667	1208912.997	0.004053611
12.93648333	1199652.162	0.004022558
12.94038333	1187907.149	0.003983176
12.94428333	1176578.498	0.00394519
12.9482	1163027.903	0.003899753
12.9521	1134382.839	0.003803703
12.95601667	1105227.776	0.003705943
12.95991667	1036855.956	0.003476686
12.96383333	986670.9451	0.00330841
12.96773333	961329.6551	0.003223438
12.97165	926854.8475	0.00310784
12.97555	896692.459	0.003006703
12.97946667	867090.3764	0.002907444
12.98336667	873752.1869	0.002929782
12.98728333	922287.4265	0.003092525
12.99118333	961984.5101	0.003225634
12.9951	973918.5533	0.00326565
12.999	1000508.611	0.003354809
13.0029	1042823.328	0.003496695
13.00681667	1149397.984	0.003854051
13.01071667	1255038.824	0.004208275
13.01463333	1345617.034	0.004511993
13.01853333	1397711.828	0.004686673
13.02245	1488464.434	0.004990976

Table 3. Samples Results STD

Retention time (minutes)	Indensity	Percentage of maximum peak area
13.02635	1535202.402	0.005147693
13.03026667	1547482.687	0.00518887
13.03416667	1580994.488	0.005301238
13.03808333	1577281.935	0.00528879
13.04198333	1541264.62	0.00516802
13.0459	1492140.67	0.005003302
13.0498	1448632.622	0.004857415
13.05371667	1404956.866	0.004710966
13.05761667	1347968.472	0.004519878
13.06153333	1255974.168	0.004211412
13.06543333	1158020.697	0.003882964
13.06935	1063274.88	0.003565271
13.07325	1015256.521	0.00340426
13.07715	975145.0114	0.003269762
13.08106667	947207.2559	0.003176084
13.08496667	925533.8495	0.003103411
13.08888333	901344.2954	0.003022301
13.09278333	907682.2984	0.003043553
13.0967	895043.7333	0.003001174
13.1006	905899.6855	0.003037576
13.10451667	900273.6139	0.003018711
13.10841667	892407.0664	0.002992333
13.11233333	884088.8496	0.002964442
13.11623333	900631.9997	0.003019913
13.12015	914574.1608	0.003066662
13.12405	930456.006	0.003119915
13.12796667	951084.955	0.003189086
13.13186667	1004978.434	0.003369797
13.13578333	1007588.321	0.003378548
13.13968333	1010590.008	0.003388613
13.14358333	1052249.313	0.003528301
13.1475	1102897.96	0.003698131
13.1514	1123499.526	0.00376721
13.15531667	1098475.325	0.003683302
13.15921667	1079193.271	0.003618647
13.16313333	1046280.263	0.003508286
13.16705	1018941.902	0.003416618
13.17095	989971.2265	0.003319476
13.17486667	952098.6116	0.003192485
13.17876667	929060.9747	0.003115238
13.18266667	893511.0224	0.002996035
13.18658333	891592.3013	0.002989601
13.19048333	887037.7105	0.002974329
13.1944	877468.2286	0.002942242
13.1983	912944.644	0.003061198
13.20221667	974229.0531	0.003266691
13.20613333	1043485.254	0.003498914
13.21003333	1108190.559	0.003715878
13.21395	1152246.333	0.003863601
13.21785	1197308.551	0.0040147
13.22175	1233466.964	0.004135943
13.22566667	1247035.025	0.004181438
13.22956667	1250321.3	0.004192457
13.23348333	1252879.861	0.004201036
13.23738333	1200959.765	0.004026943
13.2413	1170807.491	0.003925839
13.2452	1129701.343	0.003788006
13.24911667	1077018.536	0.003611355
13.25301667	1043662.431	0.003499508
13.25693333	1009018.852	0.003383345
13.26083333	984374.3463	0.003300709
13.26475	974993.6737	0.003269255
13.2687	974996.3865	0.003269264
13.27261667	938110.9676	0.003145583
13.27651667	933750.726	0.003130963
13.28043333	931611.94	0.003123791
13.28433333	923877.5747	0.003097857
13.28823333	908766.4552	0.003047188
13.29215	929231.7796	0.003115811
13.29605	936407.6525	0.003139872
13.29996667	965270.1288	0.003236651
13.30386667	967698.643	0.003244794
13.30778333	993471.2045	0.003331212
13.31168333	1005543.915	0.003371693

Table 3. Samples Results STD

Retention time (minutes)	Indensity	Percentage of maximum peak area
13.3156	999255.7314	0.003350608
13.3195	973880.1983	0.003265521
13.32341667	964222.0225	0.003233136
13.32731667	946500.1905	0.003173713
13.33123333	914078.2235	0.003064999
13.33513333	904591.3353	0.003033189
13.33905	909820.0533	0.003050721
13.34295	910064.1146	0.003051539
13.34686667	944743.4185	0.003167823
13.35076667	897308.1011	0.003008767
13.35468333	848445.0286	0.002844924
13.35858333	834022.9769	0.002796566
13.3625	818461.4259	0.002744386
13.3664	801003.4481	0.002685848
13.37031667	780034.9026	0.002615538
13.37421667	761368.2796	0.002552947
13.37813333	769467.8352	0.002580105
13.38203333	797552.7489	0.002674277
13.38595	844366.3099	0.002831248
13.38985	883090.3489	0.002961094
13.39375	873744.8968	0.002929757
13.39766667	872436.4712	0.00292537
13.40156667	883314.6607	0.002961846
13.40548333	876589.1886	0.002939294
13.40938333	870950.4295	0.002920387
13.4133	897670.6492	0.003009983
13.41721667	871704.8209	0.002922917
13.42111667	889725.4406	0.002983342
13.42503333	906356.1245	0.003039106
13.42893333	907451.3371	0.003042778
13.43285	888536.7149	0.002979356
13.43675	866874.7833	0.002906721
13.44066667	863469.5111	0.002895303
13.44456667	859178.8162	0.002880916
13.44848333	843398.8846	0.002828004
13.45238333	834759.7043	0.002799036
13.4563	843007.9583	0.002826693
13.4602	869917.1124	0.002916922
13.46411667	885869.394	0.002970412
13.46801667	921737.0884	0.00309068
13.47193333	936293.0427	0.003139488
13.47583333	890034.2704	0.002984377
13.47975	852064.1958	0.00285706
13.48365	813945.6165	0.002729244
13.48756667	788846.1032	0.002645083
13.49146667	779305.2823	0.002613091
13.49538333	774231.3859	0.002596078
13.49928333	772084.5385	0.00258888
13.5032	780217.7123	0.002616151
13.5071	804062.1717	0.002696104
13.51101667	825884.3856	0.002769276
13.51491667	835764.5928	0.002802405
13.51881667	859403.0806	0.002881668
13.52273333	890498.5351	0.002985934
13.52665	911778.2813	0.003057287
13.53055	950192.1557	0.003186093
13.53446667	976759.3696	0.003275175
13.53836667	976540.6903	0.003274442
13.54228333	959452.102	0.003217142
13.54618333	941004.9006	0.003155287
13.5501	951228.1356	0.003189567
13.554	981026.3495	0.003289483
13.55791667	1005511.853	0.003371586
13.56181667	1041274.968	0.003491503
13.56573333	1038306.746	0.00348155
13.56963333	1024560.513	0.003435458
13.57353333	1010271.835	0.003387546
13.57745	998938.1148	0.003349543
13.58136667	998041.7452	0.003346537
13.58526667	1002242.295	0.003360622
13.58918333	967563.9688	0.003244342
13.59308333	970091.6463	0.003252818
13.59698333	948546.4143	0.003180575
13.6009	932067.9562	0.003125321

Table 3. Samples Results STD

Retention time (minutes)	Indensity	Percentage of maximum peak area
13.6048	954147.7042	0.003199356
13.60871667	938767.5809	0.003147785
13.61261667	938961.1431	0.003148434
13.61653333	938642.2349	0.003147365
13.62043333	930978.694	0.003121668
13.62435	923972.9408	0.003098177
13.62825	930557.0561	0.003120254
13.63216667	917029.8702	0.003074896
13.63606667	938973.7205	0.003148476
13.63998333	931381.2493	0.003123018
13.64388333	913231.8961	0.003062161
13.6478	864765.793	0.002899649
13.6517	857112.3288	0.002873987
13.65561667	846825.6307	0.002839494
13.65951667	849725.9147	0.002849219
13.66343333	868157.5411	0.002911022
13.66733333	887796.7953	0.002976875
13.67125	913114.3553	0.003061767
13.67515	906326.9336	0.003039008
13.67906667	905376.2036	0.00303582
13.68296667	948782.9655	0.003181368
13.68688333	972930.3013	0.003262336
13.69078333	959072.5197	0.00321587
13.6947	974576.0936	0.003267855
13.6986	1009507.926	0.003384985
13.70251667	1022531.613	0.003428655
13.70641667	1003414.749	0.003364554
13.71033333	963101.066	0.003229378
13.71423333	953322.155	0.003196588
13.71815	946104.35	0.003172386
13.72205	945460.8699	0.003170228
13.72596667	961631.5489	0.00322445
13.72986667	950462.7656	0.003187
13.73378333	946627.0514	0.003174139
13.73768333	961079.7911	0.0032226
13.7416	986299.898	0.003307166
13.7455	1006091.827	0.00337353
13.74941667	1048254.315	0.003514905
13.75331667	1090051.309	0.003655055
13.75723333	1140834.569	0.003825337
13.76113333	1216090.253	0.004077677
13.76505	1413236.883	0.00473873
13.76895	1831433.906	0.006140988
13.77286667	2609915.698	0.008751318
13.77676667	3982230.157	0.013352831
13.78066667	6132869.158	0.020564146
13.78458333	9359700.116	0.031384045
13.78848333	13961610.5	0.046814728
13.7924	20133982.5	0.067511332
13.7963	27925866.88	0.093638328
13.80021667	36955866.37	0.123916853
13.80411667	46605666.02	0.156273632
13.80803333	55918899.96	0.187501871
13.81193333	64005464.9	0.214616962
13.81585	70127967.65	0.235146349
13.81975	73498668.03	0.246448657
13.82366667	73767549.04	0.247350243
13.82756667	71013758.3	0.238116497
13.83146667	65752469.8	0.220474851
13.83538333	58899011.19	0.197494493
13.83928333	51269334.75	0.171911397
13.8432	43615107.63	0.14624598
13.8471	36336305.3	0.1218394
13.85101667	29802537.17	0.099930998
13.85491667	24309880.62	0.081513551
13.85883333	19897795.94	0.066719374
13.86273333	16448787.81	0.055154492
13.86665	13812037.86	0.046313195
13.87055	11725510.84	0.039316854
13.87445	10100320.05	0.033867421
13.87836667	8828701.084	0.02960355
13.88226667	7785370.474	0.026105155
13.88618333	6899826.846	0.023135835
13.89008333	6183854.341	0.020735105

Table 3. Samples Results STD

Retention time (minutes)	Indensity	Percentage of maximum peak area
13.894	5605720.569	0.018796562
13.8979	5122674.355	0.017176858
13.90181667	4775480.652	0.016012682
13.90571667	4433474.546	0.0148659
13.90961667	4142363.225	0.013889774
13.91353333	3877210.762	0.01300069
13.91745	3655423.615	0.012257015
13.92135	3428895.176	0.011497441
13.92526667	3242825.692	0.010873531
13.92916667	3094385.051	0.010375794
13.93306667	2953530.76	0.009903495
13.93698333	2824735.495	0.009471631
13.94088333	2708896.481	0.009083211
13.9448	2591816.902	0.008690631
13.9487	2444112.708	0.008195363
13.95261667	2330899.038	0.007815746
13.95651667	2199205.071	0.007374163
13.96043333	2127762.304	0.007134608
13.96433333	2062695.824	0.006916433
13.96825	2017230.801	0.006763984
13.97215	1986552.22	0.006661116
13.97606667	1912039.853	0.006411268
13.97996667	1864419.136	0.006251591
13.98388333	1798895.414	0.006031883
13.98778333	1717294.144	0.005758265
13.9917	1682769.607	0.005642501
13.9956	1653512.719	0.0055444
13.99951667	1614224.562	0.005412662
14.00341667	1584418.125	0.005312718
14.00733333	1564072.678	0.005244498
14.01123333	1520335.218	0.005097842
14.01515	1494204.434	0.005010222
14.01905	1459751.771	0.004894699
14.02296667	1469262.249	0.004926589
14.02686667	1448462.716	0.004856846
14.03078333	1455979.383	0.00488205
14.03468333	1479741.179	0.004961726
14.0386	1453829.522	0.004874841
14.0425	1441463.485	0.004833377
14.04641667	1402269.114	0.004701954
14.05031667	1365810.643	0.004579705
14.05423333	1376708.11	0.004616245
14.05813333	1344682.995	0.004508862
14.06205	1316608.127	0.004414724
14.06595	1331536.517	0.00446478
14.06986667	1324233.831	0.004440293
14.07376667	1277230.558	0.004282687
14.07768333	1236745.049	0.004146934
14.08158333	1190504.41	0.003991885
14.0855	1175264.192	0.003940783
14.0894	1190931.029	0.003993315
14.09331667	1178803.403	0.00395265
14.09721667	1214595.101	0.004072663
14.10111667	1202816.406	0.004033168
14.10503333	1215187.445	0.00407465
14.10893333	1193133.217	0.004000699
14.11285	1158366.687	0.003884124
14.11676667	1153447.628	0.00386763
14.12066667	1167756.987	0.00391561
14.12456667	1200167.13	0.004024285
14.12848333	1180886.489	0.003959635
14.13238333	1178360.083	0.003951164
14.1363	1177899.607	0.00394962
14.1402	1186536.047	0.003978578
14.14411667	1215313.665	0.004075073
14.14801667	1236073.267	0.004144682
14.15193333	1277025.311	0.004281998
14.15583333	1297724.35	0.004351404
14.15975	1328063.824	0.004453136
14.16365	1392437.903	0.004668989
14.16756667	1464198.397	0.004909609
14.17146667	1503524.884	0.005041475
14.17538333	1500932.337	0.005032782
14.17928333	1460085.817	0.004895819

Table 3. Samples Results STD

Retention time (minutes)	Indensity	Percentage of maximum peak area
14.1832	1456133.987	0.004882568
14.1871	1431669.785	0.004800537
14.19101667	1383274.786	0.004638264
14.19491667	1342150.341	0.004500369
14.19881667	1286766.36	0.004314661
14.20273333	1257453.848	0.004216373
14.20665	1226525.4	0.004112667
14.21055	1229755.916	0.004123499
14.21445	1220074.414	0.004091036
14.21836667	1215762.927	0.004076579
14.22226667	1215063.954	0.004074235
14.22618333	1210189.382	0.004057891
14.23008333	1193346.561	0.004001415
14.234	1181583.108	0.003961971
14.2379	1167686.852	0.003915375
14.24181667	1143842.01	0.003835421
14.24571667	1135864.749	0.003808672
14.24963333	1100375.204	0.003689672
14.25353333	1140112.532	0.003822916
14.25745	1086102.671	0.003641815
14.26135	1080697.07	0.003623689
14.26526667	1061161.317	0.003558184
14.26916667	1018883.676	0.003416423
14.27308333	999484.187	0.003351374
14.27698333	1015773.47	0.003405994
14.2809	1022466.47	0.003428436
14.2848	1042606.878	0.003495969
14.28871667	1031490.204	0.003458694
14.29261667	1054054.528	0.003534354
14.29653333	1043969.876	0.003500539
14.30043333	1015948.293	0.00340658
14.30435	1020974.253	0.003423433
14.30825	1016692.317	0.003409075
14.31215	1021335.927	0.003424645
14.31606667	1031365.359	0.003458275
14.31996667	1059012.647	0.003550979
14.32388333	1037467.313	0.003478736
14.3278	1005680.275	0.00337215
14.3317	987881.4552	0.003312469
14.33561667	968671.5557	0.003248056
14.33951667	957240.8974	0.003209728
14.34343333	975913.566	0.003272339
14.34733333	988675.3084	0.003315131
14.35125	979302.083	0.003283701
14.35515	981090.5561	0.003289698
14.35906667	975786.6801	0.003271914
14.36296667	983147.6288	0.003296596
14.36688333	978280.0655	0.003280275
14.37078333	991479.1365	0.003324532
14.3747	977870.7008	0.003278902
14.3786	946087.2202	0.003172329
14.38251667	926280.2491	0.003105914
14.38641667	974803.9658	0.003268619
14.39031667	952110.9861	0.003192527
14.39423333	975199.0437	0.003269944
14.39815	988914.7641	0.003315934
14.40205	970803.2618	0.003255204
14.40596667	977265.2076	0.003276872
14.40986667	975213.1811	0.003269991
14.41378333	924867.1097	0.003101175
14.41768333	909285.0411	0.003048927
14.4216	874574.2304	0.002932538
14.4255	898717.6532	0.003013494
14.42941667	914901.6067	0.00306776
14.43331667	946826.4997	0.003174807
14.43721667	962918.4679	0.003228765
14.44113333	950219.2495	0.003186184
14.44503333	951308.6307	0.003189837
14.44895	965603.4405	0.003237768
14.45285	974839.5603	0.003268738
14.45676667	972494.6478	0.003260875
14.46066667	977240.4165	0.003276788
14.46458333	978468.9501	0.003280908
14.46848333	967900.736	0.003245472

Table 3. Samples Results STD

Retention time (minutes)	Indensity	Percentage of maximum peak area
14.4724	968498.4333	0.003247476
14.4763	963754.01	0.003231567
14.48021667	939803.3307	0.003151258
14.48411667	959372.9013	0.003216877
14.48803333	932655.7171	0.003127291
14.49193333	940606.0867	0.00315395
14.49583333	972088.8546	0.003259515
14.49975	975541.7059	0.003271093
14.50365	988261.5551	0.003313743
14.50756667	996220.5451	0.003340431
14.51146667	983194.025	0.003296752
14.51538333	992371.9187	0.003327526
14.51928333	1022122.86	0.003427284
14.5232	999571.7701	0.003351668
14.5271	988953.7261	0.003316064
14.53101667	1006877.302	0.003376164
14.53491667	995046.6764	0.003336495
14.53883333	1012440.675	0.003394819
14.54273333	1022817.544	0.003429613
14.54663333	1009060.39	0.003383484
14.55055	1014051.374	0.003400219
14.55445	991979.8318	0.003326211
14.55836667	1003327.327	0.003364261
14.56226667	1026292.66	0.003441266
14.56618333	1043454.775	0.003498812
14.57008333	1050604.338	0.003522785
14.574	1025732.621	0.003439388
14.5779	1029021.999	0.003450417
14.58181667	1050648.274	0.003522933
14.58571667	1103073.235	0.003698719
14.58963333	1160539.243	0.003891408
14.59353333	1163421.51	0.003901073
14.59745	1131391.387	0.003793673
14.60135	1111786.345	0.003727935
14.60526667	1112219.494	0.003729387
14.60916667	1131326.39	0.003793455
14.61308333	1140607.579	0.003824576
14.61698333	1138690.696	0.003818148
14.6209	1163678.884	0.003901936
14.6248	1124695.573	0.003771221
14.62871667	1092362.74	0.003662806
14.63261667	1080061.436	0.003621558
14.63653333	1118051.626	0.003748943
14.64043333	1142938.602	0.003832392
14.64433333	1150734.283	0.003858531
14.64825	1133314.737	0.003800122
14.65215	1100663.307	0.003690638
14.65606667	1083964.342	0.003634645
14.65996667	1111067.525	0.003725525
14.66388333	1107153.557	0.003712401
14.66778333	1124945.775	0.00377206
14.6717	1154535.431	0.003871277
14.6756	1160269.378	0.003890504
14.67951667	1179302.384	0.003954323
14.68341667	1182878.532	0.003966314
14.68733333	1202651.781	0.004032616
14.69123333	1213344.241	0.004068469
14.69515	1200449.703	0.004025232
14.69905	1173498.626	0.003934863
14.70296667	1121377.188	0.003760094
14.70686667	1095220.641	0.003672388
14.71078333	1052654.311	0.003529659
14.71468333	1045715.732	0.003506393
14.7186	1024082.301	0.003433854
14.7225	1025907.725	0.003439975
14.72641667	1030560.754	0.003455577
14.73031667	1022023.116	0.00342695
14.73421667	1024469.591	0.003435153
14.7382	1030238.098	0.003454495
14.74211667	1063808.719	0.003567061
14.74601667	1088271.476	0.003649087
14.74993333	1126297.888	0.003776594
14.75383333	1105119.332	0.00370558
14.75775	1102562.64	0.003697007

Table 3. Samples Results STD

Retention time (minutes)	Indensity	Percentage of maximum peak area
14.76165	1114586.45	0.003737324
14.76556667	1105374.356	0.003706435
14.76946667	1092749.84	0.003664104
14.77338333	1083274.487	0.003632332
14.77728333	1067731.437	0.003580214
14.7812	1109356.298	0.003719787
14.7851	1138154.603	0.00381635
14.78901667	1164588.175	0.003904985
14.79291667	1187648.08	0.003982307
14.79683333	1261977.028	0.00423154
14.80075	1289037.935	0.004322278
14.80465	1288475.083	0.004320391
14.80856667	1286850.939	0.004314945
14.81246667	1241093.909	0.004161517
14.81638333	1193118.177	0.004000649
14.82028333	1164035.304	0.003903131
14.82418333	1121729.568	0.003761276
14.8281	1092800.552	0.003664274
14.832	1079017.975	0.003618059
14.83591667	1057874.166	0.003547162
14.83981667	1033071.5	0.003463996
14.84373333	1024589.93	0.003435556
14.84763333	1017922.778	0.003413201
14.85155	1002850.039	0.00336266
14.85545	1024468.156	0.003435148
14.85936667	1029569.07	0.003452252
14.86326667	1103309.432	0.003699511
14.86718333	1176903.928	0.003946281
14.87108333	1261226.734	0.004229024
14.875	1336631.509	0.004481864
14.8789	1376580.629	0.004615818
14.88281667	1336685.954	0.004482047
14.88671667	1300201.242	0.00435971
14.89063333	1210469.774	0.004058831
14.89453333	1154131.751	0.003869923
14.89845	1158254.97	0.003883749
14.90235	1177428.163	0.003948039
14.90626667	1148659.076	0.003851573
14.91016667	1100365.545	0.00368964
14.91408333	1107626.643	0.003713987
14.91798333	1114516.736	0.00373709
14.9219	1114600.066	0.00373737
14.9258	1086412.036	0.003642852
14.92971667	1089866.606	0.003654436
14.93361667	1073233.846	0.003598664
14.93753333	1034598.304	0.003469115
14.94143333	1015938.028	0.003406546
14.94535	1009815.935	0.003386018
14.94925	1034827.811	0.003469885
14.95316667	1038605.944	0.003482553
14.95706667	1069582.676	0.003586422
14.96098333	1068371.46	0.00358236
14.96488333	1075617.578	0.003606657
14.9688	1056746.543	0.003543381
14.9727	1088649.653	0.003650355
14.97661667	1091717.149	0.003660641
14.98051667	1095839.123	0.003674462
14.98443333	1116598.107	0.003744069
14.98833333	1159986.348	0.003889555
14.99225	1219491.486	0.004089081
14.99615	1291941.955	0.004332015
15.00006667	1374541.279	0.004608979
15.00396667	1415853.871	0.004747505
15.00788333	1450651.231	0.004864184
15.01178333	1467281.715	0.004919948
15.0157	1461151.646	0.004899393
15.0196	1422140.391	0.004768584
15.02351667	1369854.391	0.004593264
15.02741667	1310847.295	0.004395407
15.03131667	1253793.362	0.004204099
15.03523333	1212495.462	0.004065623
15.03913333	1194543.065	0.004005427
15.04305	1195498.144	0.004008629
15.04695	1192828.207	0.003999677

Table 3. Samples Results STD

Retention time (minutes)	Indensity	Percentage of maximum peak area
15.05086667	1189862.207	0.003989731
15.05476667	1165835.744	0.003909168
15.05868333	1129315.962	0.003786714
15.06258333	1093886.757	0.003667916
15.0665	1107203.34	0.003712568
15.0704	1098278.452	0.003682642
15.0743	1099595.357	0.003687057
15.07821667	1124891.989	0.00377188
15.08211667	1172339.123	0.003930975
15.08603333	1210666.576	0.004059491
15.08993333	1262781.766	0.004234238
15.09385	1229932.777	0.004124092
15.09775	1228231.535	0.004118388
15.10166667	1223117.471	0.00410124
15.10556667	1196374.249	0.004011567
15.10948333	1173691.817	0.00393551
15.11338333	1153015.134	0.003866179
15.1173	1098896.904	0.003684715
15.1212	1043091.076	0.003497593
15.12511667	992555.5925	0.003328142
15.12901667	1000879.423	0.003356053
15.13293333	1011579.703	0.003391932
15.13683333	995535.338	0.003338133
15.14075	964008.6088	0.003232421
15.14465	989400.672	0.003317563
15.14856667	1028453.816	0.003448512
15.15246667	1033697.999	0.003466097
15.15638333	1080727.088	0.00362379
15.16028333	1061051.76	0.003557817
15.1642	1055501.802	0.003539207
15.1681	1022585.644	0.003428836
15.17201667	1037559.562	0.003479045
15.17591667	1068222.522	0.003581861
15.17983333	1072005.726	0.003594546
15.18373333	1047451.357	0.003512213
15.18765	1006986.279	0.003376529
15.19155	991458.8413	0.003324464
15.19546667	986871.1653	0.003309081
15.19936667	1027432.099	0.003445086
15.20328333	1049770.134	0.003519988
15.20718333	1083785.126	0.003634044
15.2111	1101663.799	0.003693993
15.215	1132868.31	0.003798625
15.21891667	1145122.307	0.003839714
15.22281667	1179829.163	0.00395609
15.22673333	1226497.85	0.004112575
15.23063333	1316821.723	0.00441544
15.23455	1369576.181	0.004592331
15.23845	1429844.691	0.004794418
15.24236667	1426165.1	0.004782079
15.24626667	1407092.475	0.004718127
15.25018333	1373593.995	0.004605803
15.25408333	1316032.715	0.004412794
15.258	1274287.235	0.004272817
15.2619	1219123.311	0.004087847
15.26581667	1183513.303	0.003968443
15.26971667	1143937.29	0.00383574
15.27363333	1138041.244	0.00381597
15.27753333	1088625.505	0.003650274
15.28145	1077514.88	0.003613019
15.28535	1052622.092	0.003529551
15.28926667	1052195.093	0.003528119
15.29316667	1012741.889	0.003395829
15.29706667	985644.6084	0.003304969
15.30098333	989700.8657	0.00331857
15.30488333	991187.3659	0.003323554
15.3088	1045100.706	0.003504331
15.3127	1036557.224	0.003475684
15.31661667	1025748.2	0.00343944
15.32051667	1013371.848	0.003397941
15.32443333	1013652.745	0.003398883
15.32833333	1057568.479	0.003546137
15.33225	1080265.533	0.003622242
15.33615	1078315.451	0.003615704

Table 3. Samples Results STD

Retention time (minutes)	Indensity	Percentage of maximum peak area
15.34006667	1059214.206	0.003551655
15.34396667	992705.9216	0.003328646
15.34788333	1035993.621	0.003473794
15.35178333	1061467.875	0.003559212
15.3557	1094356.232	0.00366949
15.3596	1079216.942	0.003618726
15.3635	1091209.823	0.00365894
15.36741667	1077482.84	0.003612912
15.37131667	1054109.057	0.003534537
15.37523333	1027081.621	0.003443911
15.37913333	1031346.362	0.003458211
15.38305	1035911.713	0.003473519
15.38695	1048819.879	0.003516802
15.39086667	1036864.839	0.003476715
15.39476667	1019974.14	0.003420079
15.39868333	1003989.654	0.003366481
15.40258333	1013683.259	0.003398985
15.4065	1002790.968	0.003362462
15.4104	1028753.113	0.003449516
15.4143	1020838.745	0.003422978
15.41821667	1044815.603	0.003503375
15.42213333	1045890.85	0.00350698
15.42603333	1020126.768	0.003420591
15.42995	1025278.169	0.003437864
15.43385	1011967.094	0.003393231
15.43776667	1042155.309	0.003494455
15.44166667	1053016.268	0.003530873
15.44558333	1121929.697	0.003761947
15.44948333	1154784.07	0.003872111
15.4534	1205048.271	0.004040652
15.4573	1247927.279	0.00418443
15.4612	1267676.054	0.004250649
15.46511667	1283683.026	0.004304322
15.46901667	1285126.587	0.004309163
15.47293333	1248241.115	0.004185482
15.47683333	1223081.241	0.004101118
15.48075	1185724.72	0.003975858
15.48466667	1113564.478	0.003733897
15.48856667	1068304.761	0.003582137
15.49248333	982498.6483	0.00329442
15.49638333	935436.0061	0.003136614
15.50028333	939284.2965	0.003149518
15.5042	941166.3827	0.003155828
15.50811667	943426.4165	0.003163407
15.51201667	932143.2099	0.003125573
15.51591667	920845.5656	0.003087691
15.51983333	967675.7787	0.003244717
15.52373333	978660.0019	0.003281548
15.52765	997821.6857	0.0033458
15.53155	1015577.061	0.003405335
15.53546667	1008076.636	0.003380186
15.53936667	1003132.13	0.003363606
15.54328333	990125.5492	0.003319994
15.54718333	989174.8788	0.003316806
15.5511	1013815.64	0.003399429
15.555	1042956.56	0.003497142
15.55891667	1071162.892	0.00359172
15.56281667	1089353.559	0.003652715
15.56673333	1096377.436	0.003676267
15.57063333	1104552.667	0.00370368
15.57455	1075122.309	0.003604997
15.57845	1071291.875	0.003592153
15.58236667	1045966.175	0.003507233
15.58626667	1034881.693	0.003470066
15.59018333	1015855.977	0.00340627
15.59408333	991166.4497	0.003323484
15.598	960021.787	0.003219053
15.6019	930890.3141	0.003121372
15.60581667	942048.1058	0.003158785
15.60971667	967924.5965	0.003245552
15.61363333	966540.4481	0.00324091
15.61753333	962064.4597	0.003225902
15.62145	966226.2997	0.003239857
15.62535	954946.2857	0.003202034

Table 3. Samples Results STD

Retention time (minutes)	Indensity	Percentage of maximum peak area
15.62926667	939863.0633	0.003151458
15.63316667	961600.5333	0.003224346
15.63708333	1000758.625	0.003355647
15.64098333	1024774.006	0.003436174
15.6449	1029091.17	0.003450649
15.6488	1071382.266	0.003592456
15.65271667	1107595.349	0.003713882
15.65661667	1101540.219	0.003693579
15.66053333	1100635.185	0.003690544
15.66443333	1049610.793	0.003519454
15.66835	1015671.569	0.003405652
15.67225	1015885.568	0.00340637
15.67616667	1054459.126	0.003535711
15.68006667	1069773.287	0.003587061
15.68396667	1048605.078	0.003516082
15.68788333	1059421.815	0.003552351
15.69178333	1062551.194	0.003562844
15.6957	1049542.204	0.003519224
15.6996	1053673.922	0.003533078
15.70351667	1022272.466	0.003427786
15.70741667	997961.0548	0.003346267
15.71133333	967609.8344	0.003244496
15.71523333	963295.7364	0.003230031
15.71915	990395.3856	0.003320898
15.72305	984272.1047	0.003300366
15.72696667	987831.1416	0.0033123
15.73086667	991618.296	0.003324999
15.73478333	1027531.268	0.003445419
15.73868333	1011190.453	0.003390626
15.7426	1021157.081	0.003424046
15.7465	1010544.93	0.003388462
15.75041667	999760.8214	0.003352302
15.75431667	988043.4881	0.003313012
15.75823333	933311.8275	0.003129491
15.76213333	893977.5299	0.002997599
15.76605	896447.2156	0.00300588
15.76995	885536.4622	0.002969296
15.77386667	889932.7055	0.002984037
15.77778333	887414.9014	0.002975594
15.78168333	886828.2916	0.002973627
15.7856	881255.7579	0.002954942
15.7895	879346.4946	0.00294854
15.79341667	872808.4772	0.002926617
15.79731667	859590.3376	0.002882296
15.80123333	871014.1779	0.002920601
15.80513333	887591.1568	0.002976185
15.80905	903361.8315	0.003029066
15.81295	959147.974	0.003216123
15.81686667	975793.2825	0.003271936
15.82076667	957703.0486	0.003211278
15.82468333	947496.0326	0.003177052
15.82858333	954787.3372	0.003201501
15.8325	947144.8626	0.003175875
15.8364	952116.0536	0.003192544
15.84031667	975141.0685	0.003269749
15.84421667	965647.5498	0.003237916
15.84813333	974857.8824	0.0032688
15.85205	951456.4919	0.003190332
15.85595	949648.8567	0.003184271
15.85986667	898619.9805	0.003013166
15.86376667	897514.6162	0.00300946
15.86766667	907497.4196	0.003042933
15.87158333	936106.1858	0.003138861
15.87548333	978290.7399	0.00328031
15.8794	1018211.308	0.003414168
15.8833	998540.0888	0.003348208
15.88721667	1017707.3	0.003412478
15.89111667	1000284.896	0.003354059
15.89503333	964293.5866	0.003233376
15.89893333	972680.2533	0.003261498
15.90285	963785.696	0.003231673
15.90675	963128.6467	0.00322947
15.91066667	945720.2134	0.003171098
15.91456667	920350.9569	0.003086032

Table 3. Samples Results STD

Retention time (minutes)	Indensity	Percentage of maximum peak area
15.91848333	912267.2142	0.003058927
15.92238333	904037.211	0.003031331
15.9263	875382.1862	0.002935247
15.9302	874202.0825	0.00293129
15.93411667	884749.4052	0.002966657
15.93801667	895599.332	0.003003037
15.94193333	919506.172	0.0030832
15.94583333	951621.6602	0.003190886
15.94973333	1054836.654	0.003536977
15.95365	1189037.67	0.003986967
15.95755	1392275.281	0.004668443
15.96146667	1897384.545	0.006362127
15.96536667	2845849.707	0.009542429
15.96928333	4569351.185	0.015321508
15.97318333	7417539.738	0.02487178
15.9771	11809145.11	0.039597288
15.981	18195209.66	0.061010425
15.98491667	26975727	0.09045241
15.98881667	38096177.99	0.127740436
15.99273333	51073730.81	0.171255517
15.99663333	65061485.45	0.218157909
16.00055	78678636.69	0.263817629
16.00445	90243509.05	0.302595846
16.00836667	98344778.48	0.329760242
16.01226667	101760428.2	0.34121327
16.01616667	100375234.7	0.336568573
16.02008333	94676533.57	0.317460237
16.02398333	85813299.57	0.287740894
16.0279	75059435.74	0.251682073
16.0318	63842026.51	0.214068936
16.03571667	53140726.95	0.178186369
16.03961667	43657455.26	0.146387976
16.04353333	35642220.49	0.119512062
16.04743333	29205057.31	0.097927586
16.05133333	24126929.38	0.080900097
16.05525	20290087.95	0.068034769
16.05915	17306812.86	0.058031539
16.06306667	14946111.3	0.050115861
16.06696667	13056818.69	0.043780867
16.07088333	11530196.57	0.038661945
16.07478333	10272108.52	0.034443445
16.0787	9250307.26	0.03101724
16.0826	8382215.049	0.028106436
16.08651667	7585646.963	0.025435461
16.09041667	6874495.804	0.023050897
16.09431667	6258453.603	0.020985244
16.09823333	5783194.099	0.01939165
16.10213333	5357395.697	0.017963903
16.10605	4997690.545	0.016757775
16.10996667	4655064.41	0.015608914
16.11386667	4379369.437	0.01468448
16.11776667	4104308.049	0.013762171
16.12168333	3871998.676	0.012983213
16.12558333	3664797.685	0.012288447
16.1295	3492867.207	0.011711946
16.1334	3306005.464	0.011085379
16.13731667	3138442.343	0.010523523
16.14121667	2965108.064	0.009942315
16.14513333	2862126.49	0.009597007
16.14903333	2750443.328	0.009222522
16.15295	2644058.407	0.008865802
16.15685	2584296.114	0.008665413
16.16076667	2496513.376	0.008371068
16.16466667	2391444.865	0.008018763
16.16858333	2251161.933	0.007548379
16.17248333	2146234.856	0.007196548
16.1764	2084754.781	0.006990399
16.18046667	2030590.741	0.006808781
16.18436667	2008105.823	0.006733387
16.18828333	1970348.244	0.006606782
16.19218333	1885298.926	0.006321603
16.1961	1821432.681	0.006107453
16.2	1764972.311	0.005918135
16.20391667	1699210.826	0.00569763

Table 3. Samples Results STD

Retention time (minutes)	Indensity	Percentage of maximum peak area
16.20781667	1667386.551	0.00559092
16.21173333	1608993.856	0.005395123
16.21563333	1613100.456	0.005408893
16.21955	1554221.711	0.005211467
16.22345	1505512.315	0.005048139
16.22736667	1465790.005	0.004914946
16.23126667	1446633.21	0.004850711
16.23518333	1408082.925	0.004721448
16.23908333	1392010.369	0.004667555
16.243	1385805.19	0.004646749
16.2469	1391241.378	0.004664977
16.2508	1378655.614	0.004622775
16.25471667	1385675.95	0.004646315
16.25861667	1375104.397	0.004610868
16.26253333	1369066.28	0.004590621
16.26643333	1355696.078	0.00454579
16.27035	1335079.918	0.004476661
16.27425	1328887.491	0.004455898
16.27816667	1313546.076	0.004404456
16.28208333	1326855.35	0.004449084
16.28598333	1381350.16	0.00463181
16.28988333	1447618.137	0.004854014
16.2938	1468583.894	0.004924314
16.2977	1498914.002	0.005026014
16.30161667	1559948.012	0.005230667
16.30551667	1610767.005	0.005401069
16.30943333	1717916.077	0.005760351
16.31333333	1693918.651	0.005679885
16.31725	1671973.851	0.005606302
16.32115	1630316.194	0.005466619
16.32506667	1564101.857	0.005244596
16.32896667	1499308.78	0.005027338
16.33288333	1412285.351	0.004735539
16.33678333	1364284.533	0.004574588
16.34068333	1323761.133	0.004438708
16.3446	1269510.396	0.0042568
16.3485	1230329.685	0.004125423
16.35241667	1222227.133	0.004098254
16.35631667	1186339.443	0.003977919
16.36023333	1149145.687	0.003853205
16.36413333	1143246.041	0.003833423
16.36805	1138615.455	0.003817896
16.37195	1136227.105	0.003809887
16.37586667	1154604.483	0.003871509
16.37976667	1160927.052	0.003892709
16.38368333	1158162.079	0.003883438
16.38758333	1141626.858	0.003827993
16.3915	1130995.131	0.003792344
16.3954	1148404.416	0.003850719
16.39931667	1127400.842	0.003780292
16.40321667	1143783.772	0.003835226
16.40713333	1137510.171	0.00381419
16.41103333	1098775.145	0.003684307
16.41495	1068231.254	0.00358189
16.41885	1037111.835	0.003477544
16.42276667	1036853.398	0.003476677
16.42666667	1038073.696	0.003480769
16.43058333	1031546.261	0.003458882
16.4346	1008069.558	0.003380162
16.4385	1030865.598	0.003456599
16.44241667	997106.9416	0.003343403
16.44631667	1024669.458	0.003435823
16.45023333	1059882.765	0.003553897
16.45413333	1076271.021	0.003608848
16.45805	1060714.154	0.003556685
16.46195	1015594.03	0.003405392
16.46586667	1005817.265	0.00337261
16.46976667	1007422.917	0.003377994
16.47366667	995808.9375	0.003339051
16.47758333	1017517.699	0.003411842
16.48148333	1015095.462	0.00340372
16.4854	983558.2228	0.003297973
16.48931667	969757.0989	0.003251696
16.49321667	975761.6921	0.00327183

Table 3. Samples Results STD

Retention time (minutes)	Indensity	Percentage of maximum peak area
16.49713333	965167.3461	0.003236306
16.50103333	1015418.617	0.003404804
16.50495	979868.7453	0.003285602
16.50885	984684.2912	0.003301749
16.51275	1001388.521	0.00335776
16.51666667	1014663.344	0.003402271
16.52056667	982878.0967	0.003295692
16.52448333	963523.6893	0.003230795
16.5284	989474.0231	0.003317809
16.53231667	976716.0992	0.00327503
16.53621667	961577.2386	0.003224268
16.54011667	997704.1908	0.003345406
16.54403333	997782.0049	0.003345667
16.54795	1001794.946	0.003359122
16.55185	999825.9855	0.00335252
16.55575	1006685.647	0.003375521
16.55966667	1013263.634	0.003397578
16.56356667	1009049.348	0.003383447
16.56748333	990899.8398	0.00332259
16.57138333	981267.133	0.00329029
16.5753	957112.569	0.003209298
16.5792	938484.3746	0.003146835
16.58311667	930071.1681	0.003118625
16.58701667	910349.8718	0.003052498
16.59093333	932339.5652	0.003126231
16.59483333	938393.9472	0.003146532
16.59873333	959917.1728	0.003218702
16.60265	949414.5001	0.003183485
16.60655	937548.8267	0.003143698
16.61046667	937144.2657	0.003142342
16.61436667	962599.7477	0.003227697
16.61828333	923252.1676	0.00309576
16.62218333	899291.4646	0.003015418
16.6261	893948.4894	0.002997502
16.63	882394.5702	0.002958761
16.63391667	895491.1652	0.003002675
16.63781667	899961.3337	0.003017664
16.64173333	908384.4017	0.003045907
16.64563333	913907.4101	0.003064426
16.64955	956277.8612	0.003206499
16.65345	937428.7355	0.003143296
16.65735	918141.3475	0.003078623
16.66126667	915725.5029	0.003070523
16.66516667	934540.2784	0.00313361
16.669	918768.862	0.003080727
16.673	907032.252	0.003041373
16.67683333	914289.6706	0.003065708
16.68083333	938254.5387	0.003146065
16.68466667	945712.9073	0.003171073
16.68866667	969777.952	0.003251766
16.6925	990893.7639	0.00332257
16.6965	999921.5665	0.003352841
16.70033333	1029437.39	0.00345181
16.70416667	1034432.747	0.00346856
16.70816667	1040875.046	0.003490162
16.712	1055660.975	0.003539741
16.716	1055930.957	0.003540646
16.71983333	1054897.009	0.003537179
16.72383333	1053312.933	0.003531868
16.72766667	1039837.199	0.003486682
16.73166667	994362.0884	0.003334199
16.7355	981266.6863	0.003290289
16.7395	1011060.359	0.00339019
16.74333333	1048292.383	0.003515033
16.74733333	1071132.604	0.003591619
16.75116667	1040390.44	0.003488537
16.755	1042368.481	0.00349517
16.759	1038270.094	0.003481427
16.76283333	1038749.618	0.003483035
16.76683333	1020845.919	0.003423002
16.77066667	1000182.822	0.003353717
16.77466667	1008065.789	0.003380149
16.7785	1028688.599	0.0034493
16.7825	1037949.88	0.003480354

Table 3. Samples Results STD

Retention time (minutes)	Indensity	Percentage of maximum peak area
16.78633333	1025753.816	0.003439459
16.79016667	1038913.215	0.003483584
16.79416667	1020419.571	0.003421573
16.798	1017636.107	0.003412239
16.802	1021645.271	0.003425683
16.80583333	1020339.553	0.003421304
16.80983333	1013647.814	0.003398866
16.81366667	1019094.198	0.003417129
16.81766667	1010422.917	0.003388053
16.8215	994579.0028	0.003334927
16.8255	972791.6629	0.003261871
16.82933333	978605.8497	0.003281367
16.83316667	988794.5102	0.003315531
16.83716667	994157.3598	0.003333513
16.841	984044.5228	0.003299603
16.845	991740.4942	0.003325409
16.84883333	946496.8986	0.003173702
16.85283333	911551.3936	0.003056526
16.85666667	896847.2995	0.003007222
16.8605	903118.5559	0.00302825
16.8645	886391.059	0.002972161
16.86833333	896378.9165	0.003005651
16.87233333	885822.3773	0.002970254
16.87616667	878897.5278	0.002947035
16.88016667	888169.0222	0.002978123
16.884	921484.4614	0.003089833
16.888	944223.6741	0.00316608
16.89183333	961893.5971	0.003225329
16.89583333	982130.2676	0.003293185
16.89966667	974855.7431	0.003268792
16.9035	946905.759	0.003175073
16.9075	964966.5483	0.003235633
16.91133333	995254.2539	0.003337191
16.91533333	988584.1488	0.003314825
16.91916667	989579.6228	0.003318163
16.92316667	987174.2673	0.003310098
16.927	986353.4242	0.003307345
16.931	996474.396	0.003341282
16.93483333	997557.8541	0.003344915
16.93866667	994355.3127	0.003334176
16.94266667	1017672.483	0.003412361
16.9465	1003624.8	0.003365258
16.9505	1016442.203	0.003408236
16.95433333	1001605.362	0.003358487
16.95833333	1039920.091	0.00348696
16.96216667	1039790.373	0.003486525
16.96616667	1060453.808	0.003555812
16.97	1078539.5	0.003616455
16.974	1071709.058	0.003593552
16.97783333	1117464.482	0.003746974
16.98166667	1107829.334	0.003714667
16.98566667	1099668.473	0.003687302
16.9895	1086805.978	0.003644173
16.9935	1084023.283	0.003634842
16.99733333	1058826.345	0.003550355
17.00133333	1038239.335	0.003481324
17.00516667	1026554.406	0.003442143
17.009	1008138.246	0.003380392
17.013	1004569.711	0.003368426
17.01683333	988825.0924	0.003315633
17.02083333	988322.5201	0.003313948
17.02466667	979912.0005	0.003285747
17.02866667	997250.3865	0.003343884
17.0325	1004668.325	0.003368757
17.0365	991971.1241	0.003326182
17.04033333	958667.3622	0.003214511
17.04433333	958395.8265	0.003213601
17.04816667	975520.3123	0.003271021
17.052	978654.124	0.003281529
17.056	988916.2485	0.003315939
17.05983333	998296.1009	0.00334739
17.06383333	1018276.496	0.003414387
17.06766667	1041213.858	0.003491298
17.07166667	1068797.406	0.003583789

Table 3. Samples Results STD

Retention time (minutes)	Indensity	Percentage of maximum peak area
17.0755	1102514.309	0.003696845
17.0795	1198510.755	0.004018731
17.08333333	1354626.183	0.004542202
17.08733333	1681624.518	0.005638661
17.09116667	2328372.276	0.007807274
17.095	3501104.862	0.011739568
17.099	5573075.783	0.018687101
17.10283333	9019627.823	0.030243748
17.10683333	14270108.89	0.047849155
17.11066667	21881844.51	0.073372094
17.11466667	31958422.83	0.107159906
17.1185	44581614.97	0.149486779
17.1225	59262168.53	0.198712198
17.12633333	74688522.76	0.250438363
17.13033333	89331319.78	0.29953718
17.13416667	101147259.9	0.339157253
17.138	108843881.3	0.364964823
17.142	111205928.7	0.372885013
17.14583333	108281039.8	0.363077557
17.14983333	100901126	0.33833194
17.15366667	90456351.12	0.303309527
17.15766667	78303774.28	0.262560677
17.1615	66034271.93	0.221419763
17.1655	54687847.34	0.183374024
17.16933333	44928437.79	0.150649712
17.17316667	36969628.58	0.123962999
17.17716667	30604629.2	0.102620496
17.181	25555627.25	0.085690669
17.185	21558042.87	0.072286354
17.18883333	18482139.24	0.06197253
17.19283333	16005913.82	0.053669489
17.19666667	13987689.6	0.046902174
17.20066667	12298394.52	0.041237792
17.2045	10893533.81	0.036527149
17.20833333	9704433.91	0.032539973
17.21233333	8733357.277	0.029283853
17.21616667	7897144.336	0.026479944
17.22016667	7229736.694	0.024242057
17.224	6678069.76	0.022392261
17.228	6166525.729	0.020677
17.23183333	5719530.63	0.019178179
17.23583333	5282216.877	0.017711821
17.23966667	4919181.872	0.016494527
17.2435	4605716.878	0.015443446
17.2475	4398783.395	0.014749577
17.25133333	4205261.868	0.014100679
17.25533333	3957346.986	0.013269395
17.25916667	3755757.525	0.012593445
17.26316667	3570129.357	0.011971014
17.267	3431032.767	0.011504609
17.271	3267397.631	0.010955923
17.27483333	3124825.547	0.010477864
17.27866667	2982491.21	0.010000602
17.28266667	2845403.168	0.009540932
17.2865	2702978.216	0.009063366
17.2905	2578910.202	0.008647353
17.29433333	2474469.98	0.008297154
17.29833333	2405279.594	0.008065152
17.30216667	2264888.194	0.007594405
17.30616667	2188601.052	0.007338606
17.31	2120925.057	0.007111682
17.314	2095624.318	0.007026846
17.31783333	2079923.817	0.0069742
17.32183333	2049826.492	0.006873281
17.32566667	2012606.512	0.006748478
17.3295	1965585.169	0.006590811
17.3335	1950246.398	0.006539378
17.33733333	1891628.192	0.006342826
17.34133333	1874293.927	0.006284702
17.34516667	1864356.262	0.00625138
17.34916667	1867001.232	0.006260249
17.353	1874169.138	0.006284284
17.357	1891262.918	0.006341601
17.36083333	1943905.569	0.006518117

Table 3. Samples Results STD

Retention time (minutes)	Indensity	Percentage of maximum peak area
17.36466667	1961916.951	0.006578511
17.36866667	1942000.536	0.006511729
17.3725	1912494.541	0.006412793
17.3765	1914646.244	0.006420007
17.38033333	1897286.09	0.006361797
17.38433333	1918349.89	0.006432426
17.38816667	1912988.489	0.006414449
17.392	1943065.63	0.006515301
17.396	1940287.758	0.006505986
17.39983333	1885951.214	0.00632379
17.40383333	1841998.893	0.006176413
17.40766667	1825421.767	0.006120829
17.41166667	1793563.355	0.006014004
17.4155	1773115.784	0.005945441
17.4195	1745594.056	0.005853158
17.42333333	1776891.84	0.005958103
17.42733333	1794343.354	0.006016619
17.43116667	1854673.826	0.006218914
17.435	1868975.512	0.006266869
17.439	1873406.378	0.006281726
17.44283333	1816828.341	0.006092014
17.44683333	1742979.916	0.005844392
17.45066667	1660743.259	0.005568644
17.45466667	1595286.027	0.00534916
17.4585	1482711.608	0.004971686
17.4625	1442955.22	0.004838379
17.46633333	1384585.176	0.004642658
17.47033333	1331395.983	0.004464309
17.47416667	1286139.365	0.004312559
17.478	1216171.333	0.004077949
17.482	1195538.595	0.004008765
17.48583333	1175909.43	0.003942946
17.48983333	1178521.115	0.003951704
17.49366667	1202062.045	0.004030639
17.49766667	1192031.644	0.003997006
17.5015	1211237.301	0.004061404
17.5055	1200191.275	0.004024366
17.50933333	1199123.274	0.004020785
17.51333333	1122785.952	0.003764818
17.51716667	1095677.355	0.00367392
17.521	1095241.57	0.003672459
17.525	1110006.31	0.003721966
17.52883333	1119958.95	0.003755339
17.53283333	1115656.966	0.003740914
17.53666667	1089724.501	0.003653959
17.54066667	1099627.882	0.003687166
17.5445	1114741.463	0.003737844
17.5485	1113593.972	0.003733996
17.55233333	1088873.203	0.003651105
17.55616667	1076418.228	0.003609342
17.56016667	1077422.021	0.003612708
17.564	1075893.721	0.003607583
17.568	1074631.35	0.00360335
17.57183333	1087857.391	0.003647699
17.57583333	1107457.486	0.00371342
17.57966667	1102274.024	0.003696039
17.58366667	1117194.215	0.003746068
17.5875	1048905.004	0.003517087
17.5915	1030928.137	0.003456809
17.59533333	1016165.429	0.003407308
17.59916667	1012308.887	0.003394377
17.60316667	1046761.981	0.003509901
17.607	1062373.465	0.003562248
17.611	1053001.846	0.003530824
17.61483333	1017228.729	0.003410873
17.61883333	1007411.56	0.003377955
17.62266667	1018639.481	0.003415604
17.62666667	1019153.541	0.003417328
17.6305	1038037.764	0.003480648
17.6345	1048387.258	0.003515351
17.63833333	1021837.865	0.003426328
17.64216667	976076.164	0.003272885
17.64616667	957235.7486	0.003209711
17.65	974971.0585	0.003269179

Table 3. Samples Results STD

Retention time (minutes)	Indensity	Percentage of maximum peak area
17.654	980953.2476	0.003289238
17.65783333	998711.3735	0.003348783
17.66183333	1003354.417	0.003364351
17.66566667	987672.4213	0.003311768
17.6695	978371.1772	0.00328058
17.6735	985185.155	0.003303428
17.67733333	963431.9593	0.003230487
17.68133333	988516.4438	0.003314598
17.68516667	1030841.714	0.003456519
17.68916667	1029938.199	0.00345349
17.693	1021605.56	0.003425549
17.697	1015934.316	0.003406533
17.70083333	1027766.923	0.003446209
17.70483333	1021739.51	0.003425999
17.70866667	1008379.726	0.003381202
17.7125	1007843.201	0.003379403
17.7165	1031500.761	0.003458729
17.72033333	1008876.137	0.003382866
17.72433333	996982.3067	0.003342985
17.72816667	980828.1253	0.003288818
17.73216667	969697.8584	0.003251497
17.73616667	955180.0852	0.003202818
17.74	980263.1236	0.003286924
17.744	1006006.041	0.003373243
17.74783333	1060705.668	0.003556656
17.75183333	1082166.728	0.003628617
17.75566667	1091976.311	0.00366151
17.7595	1096852.801	0.003677861
17.7635	1117779.857	0.003748032
17.76733333	1171470.203	0.003928061
17.77133333	1188278.96	0.003984423
17.77516667	1241549.001	0.004163043
17.77916667	1250218.057	0.004192111
17.783	1280510.441	0.004293684
17.787	1281651.377	0.00429751
17.79083333	1254799.641	0.004207473
17.79483333	1223549.312	0.004102688
17.79866667	1197027.76	0.004013758
17.8025	1140405.461	0.003823898
17.8065	1123093.639	0.003765849
17.81033333	1094868.922	0.003671209
17.81433333	1104478.54	0.003703431
17.81816667	1090888.299	0.003657862
17.82216667	1104668.624	0.003704068
17.826	1129500.186	0.003787331
17.83	1129298.51	0.003786655
17.83383333	1141854.977	0.003828758
17.83766667	1145591.897	0.003841288
17.84166667	1134267.455	0.003803316
17.8455	1141515.189	0.003827619
17.8495	1154943.322	0.003872645
17.85333333	1176484.091	0.003944873
17.85733333	1179217.753	0.003954039
17.86116667	1159155.868	0.00388677
17.86516667	1155488.884	0.003874474
17.869	1150821.519	0.003858824
17.873	1154043.539	0.003869628
17.87683333	1144178.354	0.003836549
17.88066667	1132906.459	0.003798753
17.88466667	1092167.032	0.003662149
17.8885	1082635.669	0.00363019
17.8925	1062253.156	0.003561845
17.89633333	1085170.953	0.003638691
17.90033333	1107600.302	0.003713899
17.90416667	1092136.538	0.003662047
17.908	1120602.481	0.003757496
17.912	1119983.206	0.00375542
17.91583333	1094864.907	0.003671196
17.91983333	1090133.752	0.003655332
17.92366667	1071008.881	0.003591204
17.92766667	1054157.833	0.003534701
17.9315	1017286.819	0.003411068
17.9355	1000158.783	0.003353636
17.93933333	985211.3627	0.003303516

Table 3. Samples Results STD

Retention time (minutes)	Indensity	Percentage of maximum peak area
17.94333333	959945.5025	0.003218797
17.94716667	987038.7289	0.003309643
17.951	995003.3558	0.003336349
17.955	987679.3888	0.003311791
17.95883333	1002307.044	0.003360839
17.96283333	977415.7598	0.003277376
17.96666667	942447.5197	0.003160124
17.97066667	935466.6285	0.003136717
17.9745	959336.6579	0.003216755
17.9785	986091.3952	0.003306467
17.98233333	990418.1502	0.003320975
17.98616667	998335.5739	0.003347523
17.99016667	998732.2158	0.003348853
17.994	978521.8971	0.003281085
17.998	976138.0978	0.003273092
18.00183333	940858.9755	0.003154798
18.00583333	930509.174	0.003120094
18.00966667	968842.1856	0.003248628
18.01366667	945259.9365	0.003169555
18.0175	935033.0389	0.003135263
18.0215	928237.6156	0.003112477
18.02533333	941643.7249	0.003157429
18.02933333	971951.8829	0.003259055
18.03316667	1021511.464	0.003425234
18.037	1019159.255	0.003417347
18.041	1025757.04	0.00343947
18.04483333	1057157.023	0.003544757
18.04883333	1049987.476	0.003520717
18.05266667	1023043.299	0.00343037
18.05666667	1030014.985	0.003453747
18.0605	1032836.998	0.00346321
18.0645	1011340.387	0.003391129
18.06833333	992140.0647	0.003326749
18.07216667	1009135.181	0.003383735
18.07616667	1050435.617	0.00352222
18.08	1039059.264	0.003484073
18.084	1007903.019	0.003379603
18.08783333	1008979.568	0.003383213
18.09183333	984363.776	0.003300674
18.09566667	971910.7811	0.003258918
18.09966667	978636.9084	0.003281471
18.1035	948182.63	0.003179355
18.1075	941231.3042	0.003156046
18.11133333	968239.2848	0.003246607
18.11516667	953833.3121	0.003198302
18.11916667	946334.7067	0.003173158
18.123	951729.6756	0.003191248
18.127	979478.552	0.003284293
18.13083333	997037.7912	0.003343171
18.13483333	1014170.416	0.003400619
18.13866667	999693.909	0.003352077
18.1425	968778.4376	0.003248415
18.1465	954406.1308	0.003200223
18.15033333	941494.1423	0.003156928
18.15433333	940737.1093	0.003154389
18.15816667	977518.3786	0.003277721
18.16216667	1015138.121	0.003403863
18.166	1033757.698	0.003466297
18.17	1035097.082	0.003470788
18.17383333	1022524.832	0.003428632
18.17766667	1048301.524	0.003515064
18.18166667	1046390.709	0.003508657
18.1855	1022862.701	0.003429765
18.1895	1027534.606	0.00344543
18.19333333	1017799.751	0.003412788
18.19733333	1005948.416	0.003373049
18.20116667	1036977.834	0.003477094
18.20516667	1056706.377	0.003543246
18.209	1126130.598	0.003776033
18.213	1311970.618	0.004399174
18.21683333	1641439.239	0.005503916
18.22066667	2309387.664	0.007743616
18.22466667	3479490.223	0.011667092
18.2285	5569586.286	0.0186754

Table 3. Samples Results STD

Retention time (minutes)	Indensity	Percentage of maximum peak area
18.2325	8984462.819	0.030125836
18.23633333	14268611.02	0.047844133
18.24033333	21900015.22	0.073433022
18.24416667	32317694.1	0.10836458
18.24816667	45391210.54	0.152201437
18.252	60624891.8	0.20328155
18.256	76935110.96	0.257971407
18.25983333	92657484.07	0.310690154
18.26366667	105859713.5	0.354958599
18.26766667	114875987.5	0.385191101
18.2715	118431658.7	0.397113635
18.2755	116145949.7	0.389449416
18.27933333	109013752.4	0.365534419
18.28333333	98455956.82	0.330133035
18.28716667	85899188.22	0.288028887
18.291	72918706.81	0.244503987
18.295	60625419	0.203283318
18.29883333	49938884.1	0.167450258
18.30283333	41034398.01	0.137592592
18.30666667	33842784.06	0.113478365
18.31066667	28141199.98	0.094360362
18.3145	23707046.57	0.079492186
18.3185	20251641.93	0.067905856
18.32233333	17409267.69	0.05837508
18.32633333	15159259.16	0.050830568
18.33016667	13289076.26	0.044559651
18.334	11790246.92	0.039533921
18.338	10574153.93	0.035456235
18.34183333	9561848.573	0.03206187
18.34583333	8656969.613	0.029027717
18.34966667	7748997.04	0.025983191
18.35366667	7045699.32	0.023624961
18.3575	6491236.686	0.02176579
18.3615	6004216.079	0.020132759
18.36533333	5589577.796	0.018742434
18.36916667	5209824.611	0.017469082
18.37316667	4861516.418	0.016301169
18.377	4544799.958	0.015239186
18.381	4225571.417	0.014168779
18.38483333	3929481.828	0.01317596
18.38883333	3689786.702	0.012372238
18.39266667	3475449.841	0.011653544
18.39666667	3286230.349	0.011019071
18.4005	3088506.455	0.010356082
18.4045	2943231.074	0.009868959
18.40833333	2801991.475	0.009395368
18.41216667	2688396.349	0.009014472
18.41616667	2591073.698	0.008688139
18.42	2516688.914	0.008438719
18.424	2399038.637	0.008044225
18.42783333	2303140.833	0.00772267
18.43183333	2213669.579	0.007422664
18.43566667	2176398.319	0.007297689
18.43966667	2136837.133	0.007165037
18.4435	2087530.574	0.006999707
18.4475	2056804.615	0.006896679
18.45133333	2039366.82	0.006838208
18.45516667	2019410.36	0.006771292
18.45916667	2065096.941	0.006924484
18.463	2084053.694	0.006988048
18.467	2115129.366	0.007092248
18.47083333	2094517.636	0.007023135
18.47483333	2082422.818	0.00698258
18.47866667	2052834.484	0.006883367
18.48266667	1987833.561	0.006665412
18.4865	1896334.471	0.006358606
18.4905	1829285.098	0.006133783
18.49433333	1728910.725	0.005797217
18.49816667	1655244.658	0.005550207
18.50216667	1593803.124	0.005344187
18.506	1543822.539	0.005176597
18.51	1488858.965	0.004992299
18.51383333	1449539.522	0.004860456
18.51783333	1449156.098	0.004859171

Table 3. Samples Results STD

Retention time (minutes)	Indensity	Percentage of maximum peak area
18.52166667	1417103.324	0.004751694
18.5255	1398240.533	0.004688446
18.5295	1395628.309	0.004679686
18.53333333	1378400.9	0.004621921
18.53733333	1379382.218	0.004625212
18.54116667	1356393.402	0.004548128
18.54516667	1338080.519	0.004486723
18.549	1335079.293	0.004476659
18.553	1289389.423	0.004323456
18.55683333	1268578.443	0.004253675
18.56066667	1237729.355	0.004150235
18.56466667	1220700.234	0.004093134
18.5685	1207307.112	0.004048226
18.5725	1222098.152	0.004097822
18.57633333	1207240.598	0.004048003
18.58033333	1224185.131	0.00410482
18.58416667	1198826.938	0.004019791
18.58816667	1180345.325	0.00395782
18.592	1183775.718	0.003969323
18.596	1142900.596	0.003832264
18.59983333	1148481.46	0.003850977
18.60383333	1152096.341	0.003863099
18.60766667	1136208.301	0.003809824
18.6115	1097689.794	0.003680668
18.6155	1138260.628	0.003816706
18.61933333	1108694.173	0.003717567
18.62333333	1131601.514	0.003794377
18.62716667	1128204.516	0.003782987
18.63116667	1135591.072	0.003807755
18.635	1129401.254	0.003787
18.639	1116745.728	0.003744564
18.64283333	1097367.602	0.003679587
18.64666667	1076706.977	0.00361031
18.65066667	1090939.203	0.003658032
18.6545	1088952.345	0.00365137
18.6585	1077617.027	0.003613362
18.66233333	1085547.051	0.003639952
18.66633333	1080004.319	0.003621366
18.67016667	1121538.529	0.003760635
18.67416667	1108966.371	0.003718479
18.678	1099435.147	0.00368652
18.682	1070295.742	0.003588813
18.68583333	1017521.381	0.003411855
18.68966667	1000385.169	0.003354395
18.69366667	997862.9494	0.003345938
18.6975	1039258.964	0.003484743
18.7015	1040514.146	0.003488952
18.70533333	1019102.491	0.003417156
18.70933333	1000381.442	0.003354383
18.71316667	1006606.717	0.003375257
18.717	990885.3789	0.003322541
18.721	967578.4633	0.003244391
18.72483333	941076.5621	0.003155527
18.72883333	935968.4064	0.003138399
18.73266667	929695.8011	0.003117366
18.73666667	911739.1945	0.003057156
18.7405	932349.937	0.003126266
18.7445	959259.1251	0.003216495
18.74833333	968778.7531	0.003248416
18.75216667	971928.031	0.003258975
18.75616667	959421.6341	0.00321704
18.76	965031.5978	0.003235851
18.764	977315.0956	0.003277039
18.76783333	982042.4433	0.00329289
18.77183333	1011334.248	0.003391109
18.77566667	979895.6308	0.003285692
18.77966667	954449.4247	0.003200368
18.7835	939152.1345	0.003149075
18.7875	969215.4236	0.00324988
18.79133333	978937.2792	0.003282478
18.79516667	976531.9746	0.003274413
18.79916667	971592.6511	0.003257851
18.803	961131.9111	0.003222775
18.807	970793.1025	0.00325517

Table 3. Samples Results STD

Retention time (minutes)	Indensity	Percentage of maximum peak area
18.81083333	955943.8265	0.003205379
18.81483333	969013.7353	0.003249204
18.81866667	969112.7373	0.003249536
18.82266667	1006846.759	0.003376062
18.8265	1003799.733	0.003365845
18.8305	959233.1941	0.003216408
18.83433333	962567.6729	0.003227589
18.83816667	985182.3584	0.003303419
18.84216667	1010490.651	0.00338828
18.846	1032159.896	0.003460939
18.85	1047989.581	0.003514018
18.85383333	1043911.311	0.003500343
18.85783333	1039660.391	0.003486089
18.86166667	1033178.173	0.003464354
18.86566667	1033351.142	0.003464934
18.8695	1039146.835	0.003484367
18.8735	1029833.381	0.003453138
18.87733333	1037050.338	0.003477337
18.88116667	1018976.494	0.003416734
18.88516667	1013917.042	0.003399769
18.889	1012304.337	0.003394361
18.893	1002226.704	0.00336057
18.89683333	1020943.066	0.003423328
18.90083333	1034268.197	0.003468009
18.90466667	1062168.648	0.003561562
18.9085	1042569.798	0.003495845
18.9125	1046569.265	0.003509255
18.91633333	1056421.962	0.003542292
18.92033333	1067704.554	0.003580124
18.92416667	1047021.29	0.003510771
18.92816667	1023413.128	0.00343161
18.932	1025213.282	0.003437646
18.936	1040280.548	0.003488169
18.93983333	1051594.426	0.003526105
18.94383333	1079929.248	0.003621115
18.94766667	1048955.604	0.003517257
18.9515	1048746.625	0.003516556
18.9555	1038620.094	0.003482601
18.95933333	999937.8171	0.003352895
18.96333333	1007430.376	0.003378019
18.96716667	986969.5618	0.003309411
18.97116667	1002709.664	0.00336219
18.975	1013573.512	0.003398617
18.979	980392.0518	0.003287356
18.98283333	968372.9147	0.003247055
18.98666667	995956.4622	0.003339545
18.99066667	1013707.946	0.003399068
18.9945	1007819.499	0.003379323
18.9985	992744.4257	0.003328775
19.00233333	1018934.571	0.003416593
19.00633333	1018668.527	0.003415701
19.01016667	991338.5307	0.003324061
19.01416667	977839.3457	0.003278797
19.018	970982.4465	0.003255805
19.022	962089.5817	0.003225986
19.02583333	949840.6809	0.003184914
19.02966667	923798.4457	0.003097592
19.03366667	948499.7088	0.003180418
19.0375	972174.6757	0.003259803
19.0415	997696.2014	0.003345379
19.04533333	1017634.412	0.003412234
19.04933333	991344.3135	0.00332408
19.05316667	980220.8269	0.003286782
19.05716667	965999.795	0.003239098
19.061	955174.7373	0.0032028
19.065	944245.8453	0.003166154
19.06883333	945487.6149	0.003170318
19.07266667	951264.6973	0.003189689
19.07666667	964788.4802	0.003235036
19.0805	973857.9773	0.003265447
19.0845	978049.6551	0.003279502
19.08833333	985149.7632	0.003303309
19.09233333	972265.0229	0.003260105
19.09616667	976112.7253	0.003273007

Table 3. Samples Results STD

Retention time (minutes)	Indensity	Percentage of maximum peak area
19.1	953185.5691	0.00319613
19.104	949473.402	0.003183683
19.10783333	958174.9374	0.00321286
19.11183333	941954.6651	0.003158472
19.11566667	936270.8016	0.003139413
19.11966667	936645.9811	0.003140671
19.1235	961725.3803	0.003224765
19.1275	990839.5459	0.003322388
19.13133333	985109.4207	0.003303174
19.13533333	997461.0389	0.00334459
19.13916667	1010369.632	0.003387874
19.143	1002955.784	0.003363015
19.147	971874.0742	0.003258795
19.15083333	950060.1384	0.00318565
19.15483333	945499.3404	0.003170357
19.15866667	950339.3404	0.003186586
19.16266667	947194.2425	0.003176041
19.1665	971358.5656	0.003257066
19.1705	972064.9359	0.003259435
19.17433333	975123.3011	0.00326969
19.17816667	967632.5111	0.003244572
19.18216667	987800.8597	0.003312199
19.186	994221.1033	0.003333726
19.19	1038097.043	0.003480847
19.19383333	1053448.731	0.003532323
19.19783333	1025269.255	0.003437834
19.20166667	1005074.081	0.003370118
19.20566667	975254.2155	0.003270129
19.2095	965535.614	0.003237541
19.2135	961204.2147	0.003223017
19.21733333	953005.8789	0.003195528
19.22133333	944580.1792	0.003167275
19.22516667	920414.784	0.003086246
19.229	942053.1887	0.003158802
19.233	949259.5189	0.003182966
19.23683333	967443.9399	0.00324394
19.24083333	971644.7447	0.003258026
19.24466667	983323.0077	0.003297184
19.24866667	988524.6562	0.003314626
19.2525	983149.01	0.003296601
19.2565	999356.3045	0.003350945
19.26033333	1003835.882	0.003365966
19.26433333	999441.8614	0.003351232
19.26816667	1007879.507	0.003379525
19.272	1000038.946	0.003353234
19.276	1011483.178	0.003391608
19.27983333	999897.0652	0.003352759
19.28383333	993604.2946	0.003331658
19.28766667	1019237.514	0.003417609
19.29166667	1013689.42	0.003399006
19.2955	1027332.65	0.003444753
19.2995	1062744.835	0.003563494
19.30333333	1061332.783	0.003558759
19.30716667	1065435.15	0.003572515
19.31116667	1064380.977	0.00356898
19.315	1097813.729	0.003681083
19.319	1099893.167	0.003688056
19.32283333	1103079.951	0.003698741
19.32683333	1106616.566	0.0037106
19.33066667	1125859.784	0.003775125
19.33466667	1106370.869	0.003709776
19.3385	1127573.774	0.003780872
19.3425	1233324.82	0.004135466
19.34633333	1463951.519	0.004908781
19.35016667	1910500.764	0.006406107
19.35416667	2772862.921	0.009297697
19.358	4324280.441	0.014499761
19.362	6953396.734	0.02331546
19.36583333	11224886.77	0.03763821
19.36983333	17593159.77	0.058991689
19.37383333	26698504.44	0.089522854
19.37766667	38653247.25	0.129608347
19.38166667	53290596.11	0.178688896
19.3855	69681806.07	0.233650323

Table 3. Samples Results STD

Retention time (minutes)	Indensity	Percentage of maximum peak area
19.3895	86598669.45	0.29037432
19.39333333	102264545.4	0.342903627
19.39733333	114978501.6	0.385534841
19.40116667	122960474.7	0.412299225
19.405	125331623.3	0.420249932
19.409	121995910.2	0.40906494
19.41283333	113537913.9	0.380704401
19.41683333	101492746.6	0.340315706
19.42066667	87654591.67	0.293914937
19.42466667	73879864.77	0.247726849
19.4285	61284767.3	0.205494181
19.4325	50451505.75	0.169169131
19.43633333	41464263.04	0.139033975
19.44016667	34211574.67	0.114714958
19.44416667	28344585.48	0.095042335
19.448	23762293.67	0.079677435
19.452	20271756.3	0.067973301
19.45583333	17329830.19	0.058108718
19.45983333	14963209.83	0.050173194
19.46366667	13057620.48	0.043783556
19.46766667	11505829.87	0.038580241
19.4715	10284775.79	0.03448592
19.4755	9355593.537	0.031370275
19.47933333	8484764.746	0.028450296
19.48316667	7674404.249	0.025733073
19.48716667	6866657.789	0.023024616
19.491	6194623.434	0.020771215
19.495	5620326.93	0.018845539
19.49883333	5242562.099	0.017578854
19.50283333	4900887.61	0.016433184
19.50666667	4552250.255	0.015264167
19.5105	4308595.286	0.014447167
19.5145	4044532.428	0.013561737
19.51833333	3769056.291	0.012638037
19.52233333	3575475.18	0.011988939
19.52616667	3336129.082	0.011186387
19.53016667	3159497.323	0.010594122
19.534	3017791.259	0.010118967
19.538	2918228.576	0.009785123
19.54183333	2814371.422	0.009436879
19.54583333	2775671.245	0.009307114
19.54966667	2726316.398	0.009141622
19.5535	2680560.303	0.008988197
19.5575	2590186.374	0.008685164
19.56133333	2534876.668	0.008499704
19.56533333	2478792.768	0.008311649
19.56916667	2441623.185	0.008187016
19.57316667	2378198.126	0.007974345
19.577	2288244.167	0.00767272
19.581	2195191.455	0.007360705
19.58483333	2073259.655	0.006951855
19.58866667	1978528.571	0.006634212
19.59266667	1903427	0.006382388
19.5965	1869815.37	0.006269685
19.6005	1808035.551	0.006062531
19.60433333	1729099.914	0.005797851
19.60833333	1636625.845	0.005487776
19.61216667	1620492.492	0.005433679
19.61616667	1641776.63	0.005505047
19.62	1677181.275	0.005623763
19.624	1656264.328	0.005553626
19.62783333	1615685.154	0.00541756
19.63166667	1575163.594	0.005281687
19.63566667	1545940.374	0.005183698
19.6395	1529769.325	0.005129475
19.6435	1521337.418	0.005101202
19.64733333	1510452.918	0.005064705
19.65133333	1492823.217	0.005005591
19.65516667	1532058.147	0.00513715
19.659	1506442.191	0.005051257
19.663	1448560.352	0.004857173
19.66683333	1444843.443	0.00484471
19.67083333	1439724.323	0.004827545
19.67466667	1449552.893	0.004860501

Table 3. Samples Results STD

Retention time (minutes)	Indensity	Percentage of maximum peak area
19.67866667	1410592.314	0.004729862
19.6825	1350196.609	0.004527349
19.6865	1334419.377	0.004474447
19.69033333	1327890.04	0.004452553
19.69416667	1350895.541	0.004529693
19.69816667	1328819.335	0.004455669
19.702	1305021.332	0.004375872
19.706	1307656.678	0.004384708
19.70983333	1332432.537	0.004467784
19.71383333	1331529.453	0.004464756
19.71766667	1289695.27	0.004324482
19.72166667	1282089.689	0.00429898
19.7255	1261293.525	0.004229248
19.7295	1287850.803	0.004318297
19.73333333	1301129.676	0.004362823
19.73716667	1324296.975	0.004440505
19.74116667	1353423.214	0.004538168
19.745	1355327.882	0.004544555
19.749	1365468.113	0.004578556
19.75283333	1413303.911	0.004738955
19.75683333	1405178.444	0.004711709
19.76066667	1376472.517	0.004615455
19.76466667	1374789.188	0.004609811
19.7685	1324508.062	0.004441213
19.7725	1289624.695	0.004324245
19.77633333	1262621.015	0.004233699
19.78016667	1218262.525	0.004084961
19.78416667	1214138.59	0.004071133
19.788	1181167.313	0.003960577
19.792	1167526.039	0.003914836
19.79583333	1131743.693	0.003794854
19.79983333	1115773.589	0.003741305
19.80366667	1121936.93	0.003761971
19.8075	1137194.612	0.003813131
19.8115	1129120.167	0.003786057
19.81533333	1123307.133	0.003766565
19.81933333	1129844.785	0.003788487
19.82316667	1118020.469	0.003748839
19.82716667	1143987.648	0.003835909
19.831	1176023.553	0.003943329
19.835	1166662.942	0.003911942
19.83883333	1161909.113	0.003896002
19.84283333	1173871.032	0.003936111
19.84666667	1135858.74	0.003808652
19.8505	1087305.252	0.003645847
19.8545	1073997.406	0.003601225
19.85833333	1094145.027	0.003668782
19.86233333	1086618.406	0.003643544
19.86616667	1106882.982	0.003711493
19.87016667	1102994.502	0.003698455
19.874	1091146.055	0.003658726
19.878	1069893.726	0.003587465
19.88183333	1057758.432	0.003546774
19.88566667	1032852.04	0.00346326
19.88966667	1059953.922	0.003554135
19.8935	1072144.326	0.003595011
19.8975	1015274.636	0.003404321
19.90133333	1024243.426	0.003434394
19.90533333	1021330.05	0.003424626
19.90916667	1040817.421	0.003489969
19.91316667	1077540.463	0.003613105
19.917	1144405.339	0.00383731
19.921	1179322.217	0.00395439
19.92483333	1247444.022	0.004182809
19.92883333	1317126.488	0.004416462
19.93266667	1406121.252	0.00471487
19.9365	1460884.16	0.004898496
19.9405	1537418.37	0.005155123
19.94433333	1603318.264	0.005376092
19.94833333	1668292.271	0.005593957
19.95216667	1666664.158	0.005588498
19.95616667	1674153.906	0.005613612
19.96	1682056.374	0.005640109
19.964	1715829.261	0.005753353

Table 3. Samples Results STD

Retention time (minutes)	Indensity	Percentage of maximum peak area
19.96783333	1703783.342	0.005712962
19.97166667	1704598.776	0.005715696
19.97566667	1700283.998	0.005701229
19.9795	1659324.622	0.005563888
19.9835	1614714.463	0.005414305
19.98733333	1540776.061	0.005166382
19.99133333	1498305.574	0.005023974
19.99516667	1440555.553	0.004830332
19.99916667	1377868.273	0.004620135
20.003	1299284.81	0.004356637
20.007	1311457.17	0.004397452
20.01083333	1276141.217	0.004279034
20.01466667	1211313.147	0.004061659
20.01866667	1114159.039	0.003735891
20.0225	1101205.846	0.003692457
20.0265	1096740.286	0.003677484
20.03033333	1056942.337	0.003544037
20.03433333	1049336.875	0.003518535
20.03816667	1021634.98	0.003425648
20.042	1032276.137	0.003461329
20.046	1060255.49	0.003555147
20.04983333	1066246.532	0.003575235
20.05383333	1071506.588	0.003592873
20.05766667	1077353.81	0.003612479
20.06166667	1062412.036	0.003562378
20.0655	1041770.541	0.003493165
20.0695	1040829.529	0.003490009
20.07333333	1075693.108	0.003606911
20.07733333	1061989.896	0.003560962
20.08116667	1099325.953	0.003686154
20.085	1099584.585	0.003687021
20.089	1097776.607	0.003680959
20.09283333	1096714.04	0.003677396
20.09683333	1061130.208	0.00355808
20.10066667	1040837.423	0.003490036
20.10466667	1031082.553	0.003457327
20.1085	1050089.998	0.003521061
20.1125	1059634.187	0.003553063
20.11633333	1071563.208	0.003593063
20.12016667	1103777.205	0.003701079
20.12416667	1115209.764	0.003739414
20.128	1109247.983	0.003719424
20.132	1075788.404	0.00360723
20.13583333	1058856.963	0.003550457
20.13983333	1093904.615	0.003667976
20.14366667	1122161.638	0.003762724
20.14766667	1141158.086	0.003826421
20.1515	1175462.497	0.003941448
20.1555	1183676.588	0.00396899
20.15933333	1160425.974	0.003891029
20.16333333	1098605.326	0.003683738
20.16716667	1100397.16	0.003689746
20.171	1093804.421	0.00366764
20.175	1072120.552	0.003594931
20.17883333	1076207.11	0.003608634
20.18283333	1079516.742	0.003619732
20.18666667	1048601.318	0.003516069
20.19066667	1069597.451	0.003586471
20.1945	1061708.429	0.003560018
20.1985	1073367.743	0.003599113
20.20233333	1053745.848	0.003533319
20.20616667	1036170.434	0.003474387
20.21016667	1060524.117	0.003556047
20.214	1067715.27	0.00358016
20.218	1067506.179	0.003579459
20.22183333	1065709.852	0.003573436
20.22583333	1100985.83	0.00369172
20.22966667	1072329.145	0.003595631
20.2335	1054502.495	0.003535856
20.2375	1050617.859	0.003522831
20.24133333	1042785.735	0.003496569
20.24533333	1023965.831	0.003433464
20.24916667	998876.0694	0.003349335
20.25316667	999192.6816	0.003350397

Table 3. Samples Results STD

Retention time (minutes)	Indensity	Percentage of maximum peak area
20.257	995821.218	0.003339092
20.261	1001598.093	0.003358462
20.26483333	1025810.605	0.003439649
20.26883333	1011473.266	0.003391575
20.27266667	984676.3366	0.003301722
20.2765	1003839.756	0.003365979
20.2805	1008945.158	0.003383098
20.28433333	1010913.346	0.003389697
20.28833333	1018584.596	0.00341542
20.29216667	1033403.486	0.003465109
20.29616667	1033752.924	0.003466281
20.3	1014867.914	0.003402957
20.304	1028458.069	0.003448527
20.30783333	1006705.393	0.003375588
20.31166667	1025138.049	0.003437394
20.31566667	1049956.149	0.003520612
20.3195	1032874.139	0.003463334
20.3235	1014701.859	0.003402401
20.32733333	995554.3586	0.003338197
20.33133333	987549.1331	0.003311355
20.33516667	990908.0792	0.003322618
20.33916667	984996.5732	0.003302796
20.343	973599.3639	0.00326458
20.347	1004861.202	0.003369404
20.35083333	998688.1409	0.003348705
20.35466667	1010860.009	0.003389518
20.35866667	1005590.405	0.003371849
20.3625	1019452.432	0.00341833
20.3665	1030116.47	0.003454087
20.37033333	1012515.828	0.003395071
20.37433333	1041778.032	0.00349319
20.37816667	1035084.941	0.003470747
20.38216667	1012396.751	0.003394671
20.386	972843.935	0.003262047
20.39	959756.8154	0.003218164
20.39383333	965709.4142	0.003238124
20.39766667	923453.5026	0.003096435
20.40166667	929630.7928	0.003117148
20.4055	915344.5481	0.003069245
20.4095	926892.5793	0.003107967
20.41333333	916567.3096	0.003073345
20.41733333	926402.1155	0.003106322
20.42116667	950592.3427	0.003187435
20.42516667	942616.5197	0.003160691
20.429	916299.9819	0.003072449
20.433	901718.3512	0.003023555
20.43683333	929858.472	0.003117912
20.44066667	937300.6668	0.003142866
20.44466667	955033.7434	0.003202327
20.4485	953201.7239	0.003196184
20.4525	939177.3499	0.003149159
20.45633333	956220.3889	0.003206306
20.46033333	961007.0956	0.003222356
20.46416667	998377.4431	0.003347663
20.468	985847.0273	0.003305647
20.472	993955.0945	0.003332835
20.47583333	1062192.265	0.003561641
20.47983333	1151982.374	0.003862716
20.48366667	1423775.617	0.004774067
20.48766667	1965391.092	0.00659016
20.4915	2977360.203	0.009983398
20.4955	4785707.61	0.016046974
20.49933333	7816710.177	0.02621024
20.50333333	12662726.59	0.042459436
20.50733333	19804525.71	0.066406629
20.51116667	29783259.67	0.099866359
20.51516667	42788994.46	0.143475936
20.519	58291759.04	0.195458314
20.523	75379221.32	0.252754347
20.52683333	92702779.19	0.310842033
20.53083333	108658450.4	0.364343054
20.53466667	121134207.8	0.406175563
20.5385	128553126.6	0.431051966
20.5425	130002771.5	0.435912776

Table 3. Samples Results STD

Retention time (minutes)	Indensity	Percentage of maximum peak area
20.54633333	125515548.7	0.420866653
20.55033333	116075467.7	0.389213082
20.55416667	103291156.7	0.346345961
20.55816667	88822479.5	0.297830986
20.562	74530864.29	0.249909718
20.566	61387612.76	0.205839033
20.56983333	50075971.9	0.167909928
20.57366667	40809009.96	0.136836843
20.57766667	33344705.38	0.111808256
20.5815	27557333.68	0.092402598
20.5855	23048493.74	0.07728399
20.58933333	19687471.43	0.066014134
20.59333333	16894655.58	0.056649532
20.59716667	14672432.1	0.049198187
20.601	12813526.41	0.042965083
20.605	11295576.09	0.037875238
20.60883333	10063478.04	0.033743886
20.61283333	9058684.461	0.030374709
20.61666667	8139612.886	0.027292966
20.62066667	7347950.508	0.02463844
20.6245	6707829.044	0.022492046
20.6285	6183314.299	0.020733294
20.63233333	5753109.773	0.019290774
20.63616667	5382348.691	0.018047573
20.64016667	5048976.636	0.016929742
20.644	4776333.837	0.016015543
20.648	4523894.797	0.015169088
20.65183333	4320219.159	0.014486143
20.65583333	4084882.557	0.013697035
20.65966667	3850373.928	0.012910703
20.66366667	3622888.563	0.012147921
20.6675	3421471.272	0.011472548
20.6715	3169687.416	0.010628291
20.67533333	2999216.07	0.010056683
20.67933333	2847061.108	0.009546491
20.68316667	2691176.335	0.009023793
20.68716667	2576565.813	0.008639492
20.691	2408593.564	0.008076264
20.695	2310561.281	0.007747552
20.69883333	2206371.36	0.007398192
20.70266667	2139442.318	0.007173772
20.70666667	2091043.532	0.007011486
20.7105	2014373.574	0.006754404
20.7145	1983663.73	0.00665143
20.71833333	1872688.81	0.00627932
20.72233333	1820229.294	0.006103418
20.72616667	1781572.906	0.005973799
20.73	1730759.792	0.005803417
20.734	1685606.403	0.005652013
20.73783333	1638847.869	0.005495227
20.74183333	1605097.282	0.005382058
20.74566667	1572713.631	0.005273472
20.74966667	1611873.588	0.005404779
20.7535	1573252.039	0.005275277
20.7575	1541845.534	0.005169968
20.76133333	1515068.736	0.005080183
20.76533333	1476765.08	0.004951746
20.76916667	1416465.488	0.004749556
20.773	1418411.36	0.00475608
20.777	1401925.854	0.004700803
20.78083333	1380953.192	0.004630479
20.78483333	1350167.033	0.00452725
20.78866667	1319140.97	0.004423216
20.79266667	1273390.207	0.004269809
20.7965	1261618.876	0.004230339
20.8005	1266012.606	0.004245072
20.80433333	1275639.483	0.004277351
20.80833333	1283521.51	0.004303781
20.81216667	1279484.086	0.004290243
20.816	1252372.056	0.004199333
20.82	1248081.429	0.004184946
20.82383333	1260289.149	0.00422588
20.82783333	1220935.754	0.004093924
20.83166667	1183296.819	0.003967717

Table 3. Samples Results STD

Retention time (minutes)	Indensity	Percentage of maximum peak area
20.83566667	1116365.429	0.003743289
20.8395	1118110.804	0.003749141
20.8435	1114116.091	0.003735747
20.84733333	1121915.04	0.003761897
20.85133333	1121844.445	0.003761661
20.85516667	1110305.709	0.00372297
20.859	1116778.568	0.003744674
20.863	1174468.343	0.003938114
20.86683333	1179496.494	0.003954974
20.87083333	1187819.737	0.003982883
20.87466667	1189672.307	0.003989095
20.87866667	1178259.041	0.003950825
20.8825	1165238.011	0.003907164
20.8865	1176359.227	0.003944454
20.89033333	1172491.795	0.003931487
20.89416667	1172631.646	0.003931956
20.89816667	1167875	0.003916006
20.902	1151947.409	0.003862599
20.906	1135977.646	0.003809051
20.90983333	1115228.892	0.003739478
20.91383333	1095313.633	0.0036727
20.91766667	1103751.713	0.003700994
20.92166667	1110773.768	0.00372454
20.9255	1095801.377	0.003674336
20.9295	1116540.284	0.003743875
20.93333333	1106430.436	0.003709976
20.93716667	1135130.822	0.003806211
20.94116667	1118792.83	0.003751428
20.945	1097221.499	0.003679097
20.949	1054045.698	0.003534325
20.95283333	1031909.524	0.0034601
20.95683333	1044821.775	0.003503396
20.96066667	1047850.853	0.003513553
20.96466667	1062014.399	0.003561044
20.9685	1063125.23	0.003564769
20.9725	1067661.512	0.00357998
20.97633333	1079576.195	0.003619931
20.98016667	1087455.627	0.003646352
20.98416667	1079786.498	0.003620636
20.988	1098737.366	0.00368418
20.992	1094474.755	0.003669887
20.99583333	1104949.99	0.003705012
20.99983333	1103285.932	0.003699432
21.00366667	1109722.951	0.003721016
21.0075	1109584.029	0.00372055
21.0115	1107685.343	0.003714184
21.01533333	1117103.279	0.003745763
21.01933333	1113699.693	0.003734351
21.02316667	1098630.256	0.003683821
21.02716667	1078298.778	0.003615648
21.031	1052795.057	0.003530131
21.035	1070655.137	0.003590018
21.03883333	1088211.838	0.003648887
21.04283333	1085163.135	0.003638665
21.04666667	1114321.99	0.003736437
21.0505	1140656.722	0.00382474
21.0545	1124240.6	0.003769695
21.05833333	1147563.467	0.003847899
21.06233333	1180695.568	0.003958995
21.06616667	1223868.814	0.004103759
21.07016667	1239274.437	0.004155416
21.074	1271750.797	0.004264312
21.078	1288262.153	0.004319677
21.08183333	1319824.252	0.004425508
21.08566667	1356488.801	0.004548448
21.08966667	1351289.873	0.004531015
21.0935	1332235.085	0.004467122
21.0975	1312513.703	0.004400995
21.10133333	1283716.707	0.004304435
21.10533333	1273897.957	0.004271512
21.10916667	1234660.022	0.004139943
21.11316667	1169560.967	0.003921659
21.117	1125587.291	0.003774211
21.121	1119028.675	0.003752219

Table 3. Samples Results STD

Retention time (minutes)	Indensity	Percentage of maximum peak area
21.12483333	1110747.744	0.003724452
21.12866667	1076368.473	0.003609175
21.13266667	1067399.366	0.003579101
21.1365	1039931.497	0.003486998
21.1405	1027881.489	0.003446593
21.14433333	999351.7176	0.00335093
21.14833333	1034696.531	0.003469445
21.15216667	1047028.607	0.003510796
21.15616667	1066229.247	0.003575177
21.16	1037862.36	0.00348006
21.164	1050102.715	0.003521103
21.16783333	1004994.877	0.003369852
21.17183333	984195.4369	0.003300109
21.17566667	988536.6937	0.003314666
21.1795	980879.2992	0.00328899
21.1835	1012363.286	0.003394559
21.18733333	1050089.551	0.003521059
21.19133333	1058723.173	0.003550009
21.19516667	1055443.21	0.003539011
21.19916667	1074272.282	0.003602146
21.203	1071203.006	0.003591855
21.207	1058812.082	0.003550307
21.21083333	1063012.76	0.003564392
21.21466667	1064470.907	0.003569281
21.21866667	1085264.14	0.003639003
21.2225	1057297.809	0.003545229
21.2265	1051977.418	0.003527389
21.23033333	1028234.642	0.003447777
21.23433333	1036567.053	0.003475717
21.23816667	1017080.304	0.003410376
21.242	1033793.591	0.003466417
21.246	1025569.152	0.00343884
21.24983333	1025532.282	0.003438716
21.25383333	1041077.742	0.003490842
21.25766667	1003259.889	0.003364034
21.26166667	1003302.036	0.003364176
21.2655	987964.8681	0.003312749
21.2695	987521.8181	0.003311263
21.27333333	995019.5166	0.003336404
21.27733333	1026144.286	0.003440768
21.28116667	1036936.974	0.003476957
21.285	1022742.308	0.003429361
21.289	1027416.808	0.003445035
21.29283333	1055072.445	0.003537767
21.29683333	1063250.755	0.00356519
21.30066667	1071043.167	0.003591319
21.30466667	1076505.553	0.003609635
21.3085	1104998.118	0.003705173
21.3125	1115984.718	0.003742013
21.31633333	1101464.148	0.003693324
21.32033333	1142860.787	0.003832131
21.32416667	1154029.437	0.00386958
21.328	1132662.885	0.003797936
21.332	1094532.548	0.003670081
21.33583333	1064885.753	0.003570672
21.33983333	1066587.087	0.003576377
21.34366667	1071336.973	0.003592304
21.34766667	1038500.743	0.003482201
21.3515	1034536.692	0.003468909
21.3555	1018089.467	0.00341376
21.35933333	1033779.287	0.003466369
21.36333333	1027196.494	0.003444296
21.36716667	1018511.403	0.003415174
21.371	990809.4875	0.003322287
21.375	993475.1116	0.003331225
21.37883333	988973.3219	0.00331613
21.38283333	993298.1656	0.003330632
21.38666667	983020.0074	0.003296168
21.39066667	974678.9214	0.0032682
21.3945	977537.8109	0.003277786
21.3985	992718.938	0.00332869
21.40233333	986637.6861	0.003308298
21.40633333	973543.6313	0.003264393
21.41016667	969495.5103	0.003250819

Table 3. Samples Results STD

Retention time (minutes)	Indensity	Percentage of maximum peak area
21.414	963178.0821	0.003229636
21.418	974690.3144	0.003268238
21.42183333	990692.0683	0.003321893
21.42583333	1003024.144	0.003363244
21.42966667	991555.173	0.003324787
21.43366667	976892.9441	0.003275623
21.4375	1012462.299	0.003394891
21.4415	1012805.889	0.003396043
21.44533333	994599.7792	0.003334996
21.44916667	985816.6197	0.003305545
21.45316667	1014160.33	0.003400585
21.457	1125258.018	0.003773107
21.461	1339456.61	0.004491337
21.46483333	1753364.244	0.005879212
21.46883333	2545960.346	0.008536869
21.47266667	3990493.243	0.013380538
21.47666667	6440348.923	0.021595158
21.4805	10424866.97	0.03495566
21.4845	16412451.31	0.055032651
21.48833333	24792975.93	0.08313342
21.49216667	35696085.99	0.119692679
21.49616667	48993353.07	0.164279794
21.5	64055003.04	0.214783069
21.504	79713176.74	0.267286549
21.50783333	94442169.26	0.316674389
21.51183333	106384413.3	0.356717972
21.51566667	113969862.8	0.382152771
21.5195	116190601.4	0.389599137
21.5235	112983102.6	0.378844061
21.52733333	105217800.3	0.352806197
21.53133333	94433180.98	0.31664425
21.53516667	82130482.35	0.275392026
21.53916667	69770273.58	0.233946964
21.543	58188770.33	0.195112982
21.547	48005880.03	0.160968695
21.55083333	39446810.8	0.132269248
21.55466667	32506332.6	0.108997105
21.55866667	27077285.45	0.090792946
21.5625	22832065.63	0.076558284
21.5665	19446456.62	0.065205986
21.57033333	16743056.74	0.056141206
21.57433333	14598045.18	0.04894876
21.57816667	12739283.5	0.042716139
21.58216667	11158888.09	0.037416909
21.586	9875239.275	0.033112702
21.59	8820305.736	0.0295754
21.59383333	8019119.592	0.02688894
21.59783333	7376641.423	0.024734644
21.60166667	6885207.118	0.023086814
21.6055	6569001.709	0.022026544
21.6095	6697600.06	0.022457748
21.61333333	7406726.629	0.024835523
21.61733333	8943331.134	0.029987917
21.62116667	11767793.47	0.039458632
21.62516667	16487042.3	0.055282763
21.629	23627873.09	0.079226709
21.633	33554159.57	0.112510577
21.63683333	46409225.39	0.155614946
21.64066667	61962735.87	0.20776748
21.64466667	79474633.47	0.266486689
21.6485	97464164.68	0.326807453
21.6525	113978036.9	0.382180179
21.65633333	127139048.5	0.426310416
21.66033333	135062230.8	0.452877668
21.66416667	136698860.7	0.458365458
21.66816667	132070361.8	0.442845621
21.672	122233649.6	0.409862105
21.676	108796000.5	0.364804274
21.67983333	93730755.61	0.314288945
21.68366667	78767524.37	0.264115679
21.68766667	64984058.74	0.217898289
21.6915	53093764.64	0.1780289
21.6955	43371920.7	0.145430549
21.69933333	35603864.66	0.119383451

Table 3. Samples Results STD

Retention time (minutes)	Indensity	Percentage of maximum peak area
21.70333333	29426672.23	0.098670684
21.70716667	24608212.31	0.082513888
21.711	20771969.05	0.069650567
21.715	17759310.21	0.059548809
21.71883333	15434154.49	0.051752321
21.72283333	13578403.25	0.045529794
21.72666667	12053306.03	0.040415985
21.73066667	10875031.58	0.036465109
21.7345	9748654.53	0.03268825
21.7385	8821470.305	0.029579305
21.74233333	8005731.107	0.026844047
21.74633333	7335091.553	0.024595323
21.75016667	6760310.419	0.022668022
21.754	6311359.899	0.021162644
21.758	5899271.815	0.01978087
21.76183333	5498257.149	0.018436226
21.76583333	5051824.091	0.01693929
21.76966667	4627966.703	0.015518052
21.77366667	4315594.835	0.014470637
21.7775	4083302.489	0.013691737
21.7815	3814357.076	0.012789935
21.78533333	3573837.089	0.011983446
21.78933333	3339126.205	0.011196436
21.79316667	3130765.196	0.01049778
21.797	2971184.212	0.009962689
21.801	2839958.095	0.009522674
21.80483333	2725624.132	0.0091393
21.80883333	2653006.34	0.008895805
21.81283333	2533713.668	0.008495805
21.81666667	2440041.591	0.008181713
21.82066667	2366378.678	0.007934713
21.8245	2293026.851	0.007688757
21.8285	2204772.149	0.00739283
21.83233333	2119785.226	0.00710786
21.83616667	2070141.923	0.006941401
21.84016667	2050384.096	0.006875151
21.844	2019209.91	0.00677062
21.848	1966216.968	0.006592929
21.85183333	1937906.406	0.006498001
21.85583333	1908360.921	0.006398932
21.85966667	1895985.995	0.006357438
21.86366667	1829815.518	0.006135561
21.8675	1818717.344	0.006098348
21.8715	1785462.44	0.005986841
21.87533333	1738785.858	0.005830329
21.87916667	1706626.128	0.005722494
21.88316667	1675334.711	0.005617571
21.887	1674565.035	0.00561499
21.891	1683482.351	0.005644891
21.89483333	1657286.151	0.005557052
21.89883333	1685400.885	0.005651324
21.90266667	1638271.373	0.005493294
21.90666667	1689704.406	0.005665754
21.9105	1725155.518	0.005784625
21.9145	1726634.973	0.005789586
21.91833333	1697862.789	0.00569311
21.92216667	1753578.969	0.005879932
21.92616667	1804264.611	0.006049886
21.93	1794971.999	0.006018727
21.934	1783517.615	0.00598032
21.93783333	1749064.509	0.005864795
21.94183333	1720649.23	0.005769515
21.94566667	1672169.02	0.005606956
21.9495	1636234.196	0.005486463
21.9535	1602831.88	0.005374462
21.95733333	1547135.654	0.005187706
21.96133333	1506790.971	0.005052426
21.96516667	1457377.668	0.004886738
21.96916667	1426540.385	0.004783338
21.973	1382703.915	0.00463635
21.977	1375457.139	0.00461205
21.98083333	1400587.908	0.004696317
21.98483333	1370011.312	0.00459379
21.98866667	1392919.718	0.004670604

Table 3. Samples Results STD

Retention time (minutes)	Indensity	Percentage of maximum peak area
21.9925	1370811.962	0.004596475
21.9965	1331367.755	0.004464214
22.00033333	1312850.989	0.004402126
22.00433333	1291094.287	0.004329173
22.00816667	1270446.596	0.004259939
22.01216667	1249462.861	0.004189579
22.016	1223959.438	0.004104063
22.02	1222017.104	0.00409755
22.02383333	1167607.672	0.00391511
22.02766667	1203731.874	0.004036238
22.03166667	1196557.378	0.004012181
22.0355	1150565.544	0.003857966
22.0395	1175392.166	0.003941212
22.04333333	1158470.826	0.003884473
22.04733333	1192069.004	0.003997131
22.05116667	1189219.342	0.003987576
22.05516667	1174347.586	0.003937709
22.059	1204040.706	0.004037273
22.063	1213425.554	0.004068742
22.06683333	1245849.427	0.004177462
22.07083333	1227946.749	0.004117433
22.07466667	1202086.413	0.00403072
22.0785	1202111.532	0.004030805
22.0825	1171952.84	0.003929679
22.08633333	1158395.184	0.003884219
22.09033333	1169452.274	0.003921295
22.09416667	1181694.628	0.003962345
22.09816667	1199621.163	0.004022454
22.102	1205727.728	0.00404293
22.106	1212880.389	0.004066914
22.10983333	1205786.948	0.004043129
22.11383333	1220658.785	0.004092996
22.11766667	1242596.275	0.004166554
22.1215	1216782.164	0.004079997
22.1255	1221834.641	0.004096938
22.12933333	1215487.759	0.004075657
22.13333333	1198833.709	0.004019814
22.13716667	1156975.657	0.003879459
22.14116667	1174431.742	0.003937991
22.145	1217387.485	0.004082027
22.149	1162972.583	0.003899568
22.15283333	1133216.047	0.003799791
22.15666667	1126359.811	0.003776801
22.16066667	1120199.095	0.003756144
22.1645	1136533.803	0.003810916
22.1685	1153969.298	0.003869379
22.17233333	1144260.053	0.003836823
22.17633333	1160037.394	0.003889726
22.18016667	1136009.522	0.003809158
22.184	1119289.602	0.003753094
22.188	1106118.151	0.003708929
22.19183333	1102118.58	0.003695518
22.19583333	1139917.706	0.003822262
22.19966667	1149140.758	0.003853188
22.20366667	1166465.732	0.003911281
22.2075	1150352.302	0.003857251
22.2115	1160845.022	0.003892434
22.21533333	1153148.532	0.003866627
22.21933333	1163300.067	0.003900666
22.22316667	1159083.158	0.003886526
22.227	1178258.593	0.003950823
22.231	1167458.847	0.003914611
22.23483333	1143925.797	0.003835702
22.23883333	1106234.229	0.003709318
22.24266667	1115777.608	0.003741318
22.24666667	1082423.093	0.003629477
22.2505	1051007.087	0.003524136
22.2545	1065753.193	0.003573581
22.25833333	1069254.527	0.003585321
22.26233333	1066443.173	0.003575895
22.26616667	1041926.681	0.003493688
22.27	1044579.322	0.003502583
22.274	1043438.203	0.003498757
22.27783333	1033767.083	0.003466328

Table 3. Samples Results STD

Retention time (minutes)	Indensity	Percentage of maximum peak area
22.28183333	1012040.767	0.003393478
22.28566667	1044011.227	0.003500678
22.28966667	1045048.654	0.003504157
22.2935	1083939.018	0.00363456
22.2975	1106726.351	0.003710968
22.30133333	1109186.859	0.003719219
22.30533333	1067922.285	0.003580854
22.30916667	1051042.671	0.003524255
22.313	1075345.385	0.003605745
22.317	1084659.136	0.003636975
22.32083333	1085507.11	0.003639818
22.32483333	1077626.53	0.003613394
22.32866667	1073048.673	0.003598043
22.33266667	1097014.711	0.003678404
22.3365	1099173.863	0.003685644
22.3405	1083190.27	0.003632049
22.34433333	1075564.785	0.00360648
22.34816667	1075177.143	0.00360518
22.35216667	1082793.352	0.003630718
22.356	1079370.14	0.00361924
22.36	1089287.445	0.003652494
22.36383333	1087710.726	0.003647207
22.36783333	1088220.528	0.003648916
22.37166667	1074350.117	0.003602407
22.37566667	1063659.133	0.003566559
22.3795	1104499.053	0.0037035
22.3835	1076841.133	0.00361076
22.38733333	1090727.915	0.003657324
22.39116667	1119529.045	0.003753897
22.39516667	1102901.375	0.003698143
22.399	1117587.579	0.003747387
22.403	1147537.016	0.003847811
22.40683333	1147195.534	0.003846666
22.41083333	1164292.549	0.003903994
22.41466667	1137775.462	0.003815079
22.4185	1118674.741	0.003751032
22.4225	1162513.341	0.003898028
22.42633333	1176594.186	0.003945242
22.43033333	1158795.25	0.003885561
22.43416667	1163687.601	0.003901965
22.43816667	1152437.076	0.003864241
22.442	1151045.715	0.003859576
22.446	1146126.098	0.00384308
22.44983333	1103554.201	0.003700332
22.45383333	1067920.54	0.003580848
22.45766667	1071554.164	0.003593032
22.4615	1067719.212	0.003580173
22.4655	1091353.809	0.003659423
22.46933333	1110327.744	0.003723044
22.47333333	1095096.362	0.003671972
22.47716667	1041229.406	0.00349135
22.48116667	1006998.459	0.00337657
22.485	1006300.197	0.003374229
22.489	1037534.769	0.003478962
22.49283333	1069731.503	0.003586921
22.49683333	1075500.302	0.003606264
22.50066667	1084151.586	0.003635273
22.5045	1082446.725	0.003629556
22.5085	1057589.741	0.003546208
22.51233333	1059571.641	0.003552854
22.51633333	1094764.442	0.003670859
22.52016667	1125260.368	0.003773115
22.52416667	1183614.396	0.003968782
22.528	1236613.774	0.004146494
22.532	1307129.583	0.004382941
22.53583333	1359994.494	0.004560203
22.53983333	1372681.274	0.004602743
22.54366667	1404603.218	0.00470978
22.5475	1420111.572	0.004761781
22.5515	1388850.728	0.004656961
22.55533333	1351442.73	0.004531528
22.55933333	1308938.278	0.004389006
22.56316667	1283968.043	0.004305278
22.56716667	1302031.309	0.004365846

Table 3. Samples Results STD

Retention time (minutes)	Indensity	Percentage of maximum peak area
22.571	1399997.111	0.004694336
22.575	1545060.357	0.005180748
22.57883333	1931169.295	0.006475411
22.58283333	2657619.511	0.008911274
22.58666667	4009039.866	0.013442726
22.5905	6283552.194	0.021069402
22.5945	9986530.216	0.033485872
22.59833333	15666033.06	0.052529834
22.60233333	23847579.02	0.079963406
22.60616667	34792643.33	0.116663342
22.61016667	48462933.24	0.162501242
22.614	64462866.53	0.216150677
22.618	81343051.87	0.27275169
22.62183333	97418350.57	0.326653833
22.62583333	110764860.2	0.371406065
22.62966667	119799488.9	0.401700111
22.6335	123550042.2	0.414276106
22.6375	121336329.1	0.406853296
22.64133333	113779426.4	0.381514218
22.64533333	102287725.2	0.342981352
22.64916667	88959523.84	0.29829051
22.65316667	75408115.02	0.252851231
22.657	62818947.56	0.210638447
22.661	51672849.18	0.173264423
22.66483333	42339865.96	0.141969962
22.66883333	34809491.82	0.116719836
22.67266667	28807037.88	0.096592986
22.6765	24060483.69	0.080677297
22.6805	20333198.41	0.068179323
22.68433333	17278260.31	0.057935799
22.68833333	14827369.72	0.049717709
22.69216667	12858405.89	0.043115569
22.69616667	11285819.12	0.037842522
22.7	10119520.15	0.033931801
22.704	9455076.202	0.031703851
22.70783333	9082144.43	0.030453372
22.71166667	9342935.995	0.031327833
22.71566667	10556701.6	0.035397715
22.7195	13145994.56	0.044079883
22.7235	17788243.91	0.059645827
22.72733333	25187953.11	0.084457819
22.73133333	35905317.77	0.120394254
22.73516667	50840681.72	0.170474079
22.739	70652410.39	0.23690486
22.743	95481608.13	0.320159735
22.74683333	125314787.7	0.42019348
22.75083333	159521020.5	0.534890527
22.75466667	196149557.3	0.65770981
22.75866667	232161850.9	0.778462765
22.7625	263732097.8	0.884321077
22.7665	286883273.9	0.961949372
22.77033333	298231156.7	1
22.77433333	295944039.5	0.992331059
22.77816667	280121370.7	0.939276009
22.782	252927812.9	0.84809319
22.786	217952117.1	0.730816054
22.78983333	180120687.7	0.603963348
22.79383333	143961520.2	0.482717909
22.79766667	112296097.3	0.376540461
22.80166667	86744141.32	0.290862103
22.8055	67313029.9	0.225707571
22.8095	53089846.47	0.178015762
22.81333333	42790507.27	0.143481009
22.81733333	35271094.24	0.118267637
22.82116667	29529573.93	0.099015724
22.825	24988630.63	0.08378947
22.829	21580390.79	0.072361289
22.83283333	18961483.69	0.063579821
22.83683333	16702837.93	0.056006348
22.84066667	14795473.45	0.049610757
22.84466667	13229995.2	0.044361546
22.8485	11878873.96	0.039831096
22.8525	10783507.18	0.036158218
22.85633333	9877089.341	0.033118905

Table 3. Samples Results STD

Retention time (minutes)	Indensity	Percentage of maximum peak area
22.86033333	9078773.75	0.03044207
22.86416667	8424143.986	0.028247029
22.868	7813450.947	0.026199311
22.872	7289575.233	0.024442702
22.87583333	6736852.981	0.022589367
22.87983333	6238601.7	0.020918679
22.88366667	5820262.444	0.019515944
22.88766667	5468739.703	0.018337251
22.8915	5210039.374	0.017469802
22.8955	4937177.463	0.016554868
22.89933333	4719849.552	0.015826145
22.90316667	4519269.639	0.01515358
22.90716667	4329927.901	0.014518697
22.911	4080416.99	0.013682061
22.915	3845165.323	0.012893238
22.91883333	3651283.47	0.012243132
22.92283333	3549035.534	0.011900284
22.92666667	3446945.019	0.011557964
22.93066667	3370629.967	0.011302072
22.9345	3284715.185	0.011013991
22.9385	3204810.11	0.010746061
22.94233333	3063539.448	0.010272366
22.94616667	2948184.566	0.009885569
22.95016667	2836932.476	0.009512529
22.954	2775560.637	0.009306743
22.958	2728067.456	0.009147493
22.96183333	2675360.102	0.00897076
22.96583333	2611915.803	0.008758025
22.96966667	2575591.577	0.008636226
22.97366667	2470470.819	0.008283745
22.9775	2408388.575	0.008075577
22.9815	2335530.194	0.007831275
22.98533333	2268688.318	0.007607147
22.98916667	2169331.521	0.007273994
22.99316667	2078044.24	0.006967898
22.997	2012283.997	0.006747397
23.001	1979153.289	0.006636306
23.00483333	1949943.255	0.006538362
23.00883333	1927564.013	0.006463322
23.01266667	1844213.062	0.006183838
23.01666667	1803960.916	0.006048868
23.0205	1798899.269	0.006031896
23.02433333	1766488.42	0.005923219
23.02833333	1740444.76	0.005835892
23.03216667	1706588.025	0.005722367
23.03616667	1680759.668	0.005635761
23.04	1676803.242	0.005622495
23.044	1674332.968	0.005614212
23.04783333	1649871.575	0.005532191
23.05183333	1631212.119	0.005469623
23.05566667	1580043.036	0.005298048
23.0595	1531329.067	0.005134705
23.0635	1480236.974	0.004963388
23.06733333	1494695.4	0.005011869
23.07133333	1516851.592	0.005086161
23.07516667	1506822.285	0.005052531
23.07916667	1501362.393	0.005034224
23.083	1471035.714	0.004932535
23.087	1444419.148	0.004843287
23.09083333	1414480.769	0.004742901
23.09483333	1417311.917	0.004752394
23.09866667	1402602.465	0.004703072
23.1025	1420299.315	0.004762411
23.1065	1425576.755	0.004780107
23.11033333	1408952.008	0.004724362
23.11433333	1409132.829	0.004724969
23.11816667	1389002.349	0.004657469
23.12216667	1366710.624	0.004582722
23.126	1340522.456	0.004494911
23.13	1355930.829	0.004546577
23.13383333	1351710.694	0.004532426
23.13783333	1340136.979	0.004493618
23.14166667	1326992.256	0.004449543
23.1455	1305338.647	0.004376936

Table 3. Samples Results STD

Retention time (minutes)	Indensity	Percentage of maximum peak area
23.1495	1292797.645	0.004334885
23.15333333	1266471.329	0.00424661
23.15733333	1277781.172	0.004284533
23.16116667	1310431.303	0.004394012
23.16516667	1311514.005	0.004397642
23.169	1294188.304	0.004339548
23.173	1257532.72	0.004216638
23.17683333	1238773.552	0.004153736
23.18066667	1231818.117	0.004130414
23.18466667	1239858.101	0.004157373
23.1885	1255590.95	0.004210127
23.1925	1235038.602	0.004141213
23.19633333	1254902.007	0.004207817
23.20033333	1238283.982	0.004152095
23.20416667	1202938.329	0.004033577
23.20816667	1224270.05	0.004105104
23.21216667	1239321.553	0.004155574
23.216	1248682.083	0.004186961
23.22	1272128.995	0.00426558
23.22383333	1243653.475	0.004170099
23.22783333	1195620.222	0.004009039
23.23166667	1202667.275	0.004032668
23.2355	1230762.785	0.004126875
23.2395	1239468.997	0.004156068
23.24333333	1207102.47	0.00404754
23.24733333	1222888.717	0.004100473
23.25116667	1216009.209	0.004077405
23.25516667	1204697.514	0.004039476
23.259	1189533.548	0.003988629
23.263	1189900.773	0.003989861
23.26683333	1197247.106	0.004014494
23.27083333	1184576.579	0.003972008
23.27466667	1207360.961	0.004048407
23.2785	1250448.193	0.004192882
23.2825	1271165.244	0.004262349
23.28633333	1274177.195	0.004272448
23.29033333	1273555.203	0.004270363
23.29416667	1264546.378	0.004240155
23.29816667	1261175.33	0.004228852
23.302	1283030.918	0.004302136
23.306	1260381.518	0.00422619
23.30983333	1225477.11	0.004109152
23.31366667	1211323.09	0.004061692
23.31766667	1222975.575	0.004100764
23.3215	1178279.045	0.003950892
23.3255	1153763.62	0.003868689
23.32933333	1154329.881	0.003870588
23.33333333	1150596.503	0.003858069
23.33716667	1148549.896	0.003851207
23.34116667	1120273.041	0.003756392
23.345	1099342.262	0.003686209
23.349	1101453.435	0.003693288
23.35283333	1077055.154	0.003611478
23.35666667	1090600.309	0.003656896
23.36066667	1082489.025	0.003629698
23.3645	1093854.294	0.003667807
23.3685	1101509.409	0.003693475
23.37233333	1097131.663	0.003678796
23.37633333	1071281.401	0.003592118
23.38016667	1100821.008	0.003691167
23.38416667	1107665.095	0.003714116
23.388	1120068.907	0.003755707
23.392	1101959.057	0.003694983
23.39583333	1095860.711	0.003674535
23.39966667	1102795.69	0.003697788
23.40366667	1097159.105	0.003678888
23.4075	1099746.861	0.003687565
23.4115	1099616.59	0.003687128
23.41533333	1118812.377	0.003751494
23.41933333	1102356.548	0.003696316
23.42316667	1097044.536	0.003678504
23.427	1103596.159	0.003700472
23.431	1078672.551	0.003616901
23.43483333	1049989.295	0.003520723

Table 3. Samples Results STD

Retention time (minutes)	Indensity	Percentage of maximum peak area
23.43883333	1058660.483	0.003549798
23.44266667	1089431.416	0.003652977
23.44683333	1137042.653	0.003812622
23.45066667	1160920.967	0.003892688
23.4545	1141828.148	0.003828668
23.4585	1137793.719	0.00381514
23.46233333	1170903.935	0.003926162
23.46633333	1180661.185	0.003958879
23.47016667	1218535.032	0.004085874
23.47416667	1203455.079	0.00403531
23.478	1218131.049	0.00408452
23.482	1231885.305	0.004130639
23.48583333	1206077.438	0.004044103
23.48966667	1201059.998	0.004027279
23.49366667	1151391.754	0.003860736
23.4975	1144837.035	0.003838757
23.5015	1168919.87	0.00391951
23.50533333	1155229.836	0.003873605
23.50933333	1121505.567	0.003760524
23.51316667	1111432.206	0.003726747
23.51716667	1144744.076	0.003838446
23.521	1173793.646	0.003935852
23.525	1343126.994	0.004503644
23.52883333	1724855.888	0.005783621
23.53266667	2456469.133	0.008236796
23.53666667	3816792.876	0.012798102
23.5405	6099764.319	0.020453142
23.5445	9822829.474	0.032936966
23.54833333	15581212.15	0.05224542
23.55233333	23856095.91	0.079991964
23.55616667	34862648.64	0.116898077
23.56	48630235.6	0.163062224
23.564	64775263.04	0.217198175
23.56783333	82285371.15	0.275911384
23.57183333	99693544.72	0.334282795
23.57566667	114810340.2	0.384970979
23.57966667	125668350.8	0.421379014
23.5835	130974339.9	0.439170546
23.5875	129963004.3	0.435779433
23.59133333	123264422.6	0.413318393
23.59516667	111976611.6	0.375469193
23.59916667	97949543.11	0.328434977
23.603	83047260.54	0.278466078
23.607	68937651.58	0.231155096
23.61083333	56473630.01	0.189361939
23.61483333	45915656.43	0.153959958
23.61866667	37533981.89	0.125855334
23.62266667	30945062.37	0.103762004
23.6265	25722125.09	0.086248953
23.6305	21597023.87	0.072417061
23.63433333	18290743.68	0.061330761
23.63816667	15662308.47	0.052517345
23.64216667	13559746.61	0.045467237
23.646	11848635.04	0.039729702
23.65	10502365.85	0.035215522
23.65383333	9346489.385	0.031339748
23.65783333	8379687.979	0.028097963
23.66166667	7565412.892	0.025367614
23.66566667	6869653.956	0.023034662
23.6695	6229930.343	0.020889603
23.6735	5678048.189	0.019039084
23.67733333	5224291.496	0.017517591
23.68116667	4834698.121	0.016211244
23.68516667	4523195.377	0.015166743
23.689	4222707.373	0.014159176
23.693	3970761.811	0.013314376
23.69683333	3746510.469	0.012562438
23.70083333	3630233.743	0.01217255
23.70466667	3551297.902	0.01190787
23.7085	3426086.326	0.011488023
23.7125	3329328.651	0.011163584
23.71633333	3275771.332	0.010984001
23.72033333	3170020.58	0.010629408
23.72416667	3016892.107	0.010115952

Table 3. Samples Results STD

Retention time (minutes)	Indensity	Percentage of maximum peak area
23.72816667	2891636.574	0.009695957
23.732	2776723.632	0.009310642
23.736	2711525.327	0.009092026
23.73983333	2638532.013	0.008847272
23.74383333	2570670.817	0.008619726
23.74766667	2385462.14	0.007998702
23.7515	2280057.66	0.00764527
23.7555	2192939.349	0.007353153
23.75933333	2113770.76	0.007087693
23.76333333	2001799.365	0.006712241
23.76716667	1977977.583	0.006632364
23.77116667	1910653.005	0.006406618
23.775	1867528.849	0.006262018
23.779	1856855.561	0.006226229
23.78283333	1876043.429	0.006290568
23.78683333	1961662.723	0.006577659
23.79066667	2191507.149	0.007348351
23.7945	2664782.364	0.008935292
23.7985	3568069.527	0.011964107
23.80233333	5215411.866	0.017487817
23.80633333	8010261.006	0.026859236
23.81016667	12428501.15	0.041674053
23.81416667	18915167.96	0.06342452
23.818	27905070.89	0.093568597
23.822	39619220.72	0.132847356
23.82583333	54399742.4	0.182407978
23.82983333	71343117.81	0.239220874
23.83366667	89729339.71	0.300871783
23.8375	107749720.7	0.361295989
23.8415	123385270.3	0.413723608
23.84533333	134534546.7	0.451108289
23.84933333	139939779.9	0.469232596
23.85316667	138644080.4	0.464887981
23.85716667	131318082.4	0.44032315
23.861	118664099.5	0.397893033
23.865	103115418.9	0.345756694
23.86883333	86409479.91	0.289739948
23.87266667	70614606.77	0.2367781
23.87666667	56779844.42	0.190388707
23.8805	45310776.3	0.151931732
23.8845	36348890.79	0.121881601
23.88833333	29485374.22	0.098867518
23.89233333	24230336.46	0.081246831
23.89616667	20168513.74	0.067627118
23.90016667	17041801.32	0.057142927
23.904	14647060.85	0.049113114
23.908	12756865.86	0.042775094
23.91183333	11204606.45	0.037570208
23.91566667	9924480.782	0.033277813
23.91966667	8924702.786	0.029925454
23.9235	7972670.648	0.026733192
23.9275	7209912.716	0.024175585
23.93133333	6509049.517	0.021825518
23.93533333	5867371.912	0.019673907
23.93916667	5380263.028	0.01804058
23.943	4966058.825	0.01665171
23.947	4586918.582	0.015380414
23.95083333	4320479.617	0.014487016
23.95483333	4118546.372	0.013809913
23.95866667	3839769.996	0.012875147
23.96266667	3637409.494	0.012196611
23.9665	3439334.538	0.011532445
23.9705	3202020.101	0.010736706
23.97433333	3035688.864	0.01017898
23.97816667	2882626.899	0.009665747
23.98216667	2797592.546	0.009380618
23.986	2725911.351	0.009140263
23.99	2655772.19	0.00890508
23.99383333	2547416.061	0.00854175
23.99783333	2440570.061	0.008183485
24.00166667	2378390.712	0.007974991
24.00566667	2319535.085	0.007777642
24.0095	2218265.004	0.007438073
24.0135	2130486.135	0.007143741

Table 3. Samples Results STD

Retention time (minutes)	Indensity	Percentage of maximum peak area
24.01733333	2051820.582	0.006879967
24.02116667	2007467.272	0.006731246
24.02516667	1946343.534	0.006526292
24.029	1925919.627	0.006457808
24.033	1875766.193	0.006289639
24.03683333	1818580.288	0.006097888
24.04083333	1821266.618	0.006106896
24.04466667	1781784.811	0.005974509
24.04866667	1764369.266	0.005916113
24.0525	1748948.238	0.005864405
24.0565	1725582.968	0.005786059
24.06033333	1703630.777	0.005712451
24.06433333	1640017.891	0.00549915
24.06816667	1619124.453	0.005429092
24.072	1624931.203	0.005448563
24.076	1584802.491	0.005314007
24.07983333	1580173.752	0.005298486
24.08383333	1546335.285	0.005185023
24.08766667	1504345.369	0.005044226
24.09166667	1498380.628	0.005024226
24.0955	1493008.839	0.005006213
24.0995	1480301.467	0.004963604
24.10333333	1449618.863	0.004860722
24.10716667	1458216.688	0.004889552
24.11116667	1442149.501	0.004835677
24.115	1448191.3	0.004855936
24.119	1433683.752	0.00480729
24.12283333	1427559.168	0.004786754
24.12683333	1388883.311	0.00465707
24.13066667	1393865.821	0.004673777
24.13466667	1379791.126	0.004626583
24.1385	1407743.224	0.004720309
24.1425	1344901.27	0.004509593
24.14633333	1301998.327	0.004365735
24.15016667	1303254.677	0.004369948
24.15416667	1302074.146	0.00436599
24.158	1297683.168	0.004351266
24.162	1329241.98	0.004457086
24.16583333	1320314.896	0.004427153
24.16983333	1283042.578	0.004302175
24.17366667	1314338.086	0.004407112
24.17766667	1296506.713	0.004347321
24.1815	1308998.321	0.004389207
24.1855	1315190.159	0.004409969
24.18933333	1353498.265	0.00453842
24.19316667	1341369.539	0.004497751
24.19716667	1307589.956	0.004384485
24.201	1275559.123	0.004277082
24.205	1270043.04	0.004258586
24.20883333	1287300.934	0.004316454
24.21283333	1264652.887	0.004240512
24.21666667	1246878.839	0.004180914
24.22066667	1250914.912	0.004194447
24.2245	1272629.681	0.004267259
24.2285	1264247.236	0.004239152
24.23233333	1241941.246	0.004164358
24.23616667	1230129.784	0.004124753
24.24016667	1219289.408	0.004088404
24.244	1228970.886	0.004120867
24.248	1249922.283	0.004191119
24.25183333	1247280.025	0.004182259
24.25583333	1280342.287	0.00429312
24.25966667	1302401.473	0.004367087
24.26366667	1293342.593	0.004336712
24.2675	1274302.328	0.004272868
24.2715	1238804.352	0.004153839
24.27533333	1242674.807	0.004166818
24.27916667	1222727.206	0.004099931
24.28316667	1220795.606	0.004093454
24.287	1211703.581	0.004062968
24.291	1223070.781	0.004101083
24.29483333	1245148.211	0.004175111
24.29883333	1253022.562	0.004201515
24.30266667	1248938.548	0.00418782

Table 3. Samples Results STD

Retention time (minutes)	Indensity	Percentage of maximum peak area
24.30666667	1262937.889	0.004234762
24.3105	1258996.594	0.004221546
24.3145	1299027.293	0.004355773
24.31833333	1316855.575	0.004415553
24.32216667	1370701.214	0.004596103
24.32616667	1387471.427	0.004652336
24.33	1405568.776	0.004713018
24.334	1405338.425	0.004712245
24.33783333	1419098.378	0.004758384
24.34183333	1441439.616	0.004833297
24.34566667	1439550.46	0.004826962
24.3495	1447362.716	0.004853157
24.3535	1450178.433	0.004862599
24.35733333	1432493.485	0.004803299
24.36133333	1431504.47	0.004799983
24.36516667	1455658.315	0.004880973
24.36916667	1454150.482	0.004875917
24.373	1463780.066	0.004908206
24.377	1488549.122	0.00499126
24.38083333	1513996.601	0.005076588
24.38483333	1517481.619	0.005088273
24.38866667	1519980.297	0.005096652
24.3925	1546633.334	0.005186022
24.3965	1518934.558	0.005093145
24.40033333	1469499.328	0.004927384
24.40433333	1457535.521	0.004887268
24.40816667	1429975.702	0.004794857
24.41216667	1439422.009	0.004826531
24.416	1443018.44	0.00483859
24.42	1439924.009	0.004828215
24.42383333	1442466.541	0.00483674
24.42783333	1435239.127	0.004812506
24.43166667	1430556.767	0.004796805
24.4355	1450088.207	0.004862296
24.4395	1438889.573	0.004824746
24.44333333	1388939.291	0.004657257
24.44733333	1346319.785	0.00451435
24.45116667	1353137.576	0.004537211
24.45516667	1347917.646	0.004519708
24.459	1346870.443	0.004516196
24.463	1341904.627	0.004499545
24.46683333	1353645.405	0.004538913
24.47066667	1346649.692	0.004515456
24.47466667	1346074.325	0.004513527
24.4785	1313685.424	0.004404923
24.4825	1293227.036	0.004336324
24.48633333	1274038.878	0.004271984
24.49033333	1267489.415	0.004250023
24.49416667	1244857.962	0.004174138
24.49816667	1279023.45	0.004288698
24.502	1304052.882	0.004372625
24.506	1272470.837	0.004266727
24.50983333	1264142.945	0.004238802
24.51383333	1235597.099	0.004143085
24.51766667	1215691.092	0.004076338
24.5215	1239349.898	0.004155669
24.5255	1264930.457	0.004241443
24.52933333	1304792.985	0.004375106
24.53333333	1296357.741	0.004346822
24.53716667	1243798.045	0.004170584
24.54116667	1241194.142	0.004161853
24.545	1256078.018	0.00421176
24.549	1240238.901	0.00415865
24.55283333	1224328.726	0.004105301
24.55666667	1188986.485	0.003986795
24.56066667	1204227.127	0.004037898
24.5645	1174273.861	0.003937462
24.5685	1181791.137	0.003962668
24.57233333	1192083.401	0.003997179
24.57633333	1190221.806	0.003990937
24.58016667	1206199.557	0.004044512
24.58416667	1199932.705	0.004023499
24.588	1195957.907	0.004010171
24.592	1258833.645	0.004221

Table 3. Samples Results STD

Retention time (minutes)	Indensity	Percentage of maximum peak area
24.59583333	1315769.222	0.004411911
24.59966667	1324516.193	0.00444124
24.60366667	1297383.182	0.00435026
24.6075	1281514.364	0.004297051
24.6115	1276066.459	0.004278783
24.61533333	1306150.763	0.004379659
24.61933333	1357572.583	0.004552082
24.62316667	1488650.105	0.004991598
24.62716667	1752989.629	0.005877956
24.631	2170959.069	0.007279451
24.635	3004211.951	0.010073434
24.63883333	4443994.678	0.014901175
24.64266667	6876999.912	0.023059294
24.64666667	10738087.49	0.036005921
24.6505	16501942.21	0.055332724
24.6545	24591089.95	0.082456475
24.65833333	35291023.73	0.118334463
24.66233333	48649044.34	0.163125291
24.66616667	64208174.38	0.215296668
24.67	80971775.62	0.271506762
24.674	97453597.21	0.326772019
24.67783333	111806364.3	0.374898336
24.68183333	122321908.9	0.410158047
24.68566667	127726356.7	0.428279721
24.68966667	127173448.8	0.426425764
24.6935	121139614.8	0.406193693
24.6975	110730948.2	0.371292354
24.70133333	97532530.61	0.327036691
24.70533333	83331374.03	0.27941874
24.70916667	69586958.68	0.23333229
24.713	57164631.17	0.191678937
24.717	46631231.36	0.156359355
24.72083333	38016744.9	0.127474089
24.72483333	31298523.03	0.104947194
24.72866667	26030579.42	0.087283233
24.73266667	21805698.24	0.073116768
24.7365	18371770.34	0.061602451
24.7405	15785459.84	0.052930284
24.74433333	13637623.94	0.045728368
24.74816667	11832940.03	0.039677075
24.75216667	10408692.17	0.034901424
24.756	9272869.341	0.031092893
24.76	8381465.541	0.028103923
24.76383333	7647291.084	0.02564216
24.76783333	6977197.809	0.023395268
24.77166667	6363972.707	0.021339061
24.77566667	5800240.844	0.019448809
24.7795	5338296.009	0.01789986
24.7835	4865738.634	0.016315326
24.78733333	4505782.164	0.015108355
24.79133333	4178114.341	0.014009651
24.79516667	3910678.228	0.01311291
24.799	3713428.347	0.01245151
24.803	3590521.773	0.012039392
24.80683333	3377426.24	0.01132486
24.81083333	3191413.788	0.010701141
24.81466667	3081930.103	0.010334031
24.81866667	2947397.572	0.00988293
24.8225	2834297.57	0.009503694
24.8265	2756834.117	0.009243951
24.83033333	2606045.17	0.00873834
24.83416667	2503303.681	0.008393837
24.83816667	2391909.112	0.008020319
24.842	2314029.291	0.00775918
24.846	2304869.819	0.007728468
24.84983333	2318164.657	0.007773047
24.85383333	2282128.997	0.007652215
24.85766667	2178140.774	0.007303532
24.86166667	2132380.017	0.007150091
24.8655	2081138.445	0.006978273
24.86933333	2055101.507	0.006890969
24.87333333	2029692.375	0.006805769
24.87716667	1994903.395	0.006689118
24.88116667	1972700.641	0.00661467

Table 3. Samples Results STD

Retention time (minutes)	Indensity	Percentage of maximum peak area
24.885	2005852.371	0.006725831
24.889	2107237.99	0.007065788
24.89283333	2270779.292	0.007614158
24.89683333	2669042.202	0.008949575
24.90066667	3437806.059	0.01152732
24.9045	4725915.129	0.015846484
24.9085	6921538.092	0.023208635
24.91233333	10293945.04	0.034516665
24.91633333	15143721.43	0.050778469
24.92016667	21857768.11	0.073291364
24.92416667	30930507.64	0.1037132
24.928	42093563.38	0.141144084
24.932	55203315.42	0.185102442
24.93583333	70014129.58	0.234764638
24.93966667	85748380.63	0.287523214
24.94366667	101385301.2	0.33995543
24.9475	115637614.1	0.387744914
24.9515	127032408.3	0.425952841
24.95533333	133949396.9	0.449146221
24.95933333	135304736.8	0.453690816
24.96316667	130801218.9	0.438590053
24.96716667	121267199.4	0.406621497
24.971	108112850	0.362513599
24.975	93230582.72	0.312611813
24.97883333	77947676.75	0.261366645
24.98283333	63751298.78	0.213764717
24.98666667	51442212.52	0.172491074
24.99066667	41433568.76	0.138931053
24.9945	33554366.34	0.112511271
24.9985	27454243.92	0.092056927
25.00233333	22692391.98	0.076089944
25.00616667	19071595.25	0.063949037
25.01016667	16178324.56	0.0542476
25.014	13887087.09	0.046564843
25.018	12044589.05	0.040386756
25.02183333	10587638.67	0.035501451
25.02583333	9427677.262	0.03161198
25.02966667	8498760.495	0.028497225
25.0335	7634361.04	0.025598804
25.0375	6943766.499	0.023283169
25.04133333	6367599.85	0.021351223
25.04533333	5827578.992	0.019540477
25.04916667	5389485.053	0.018071502
25.05316667	4966393.63	0.016652833
25.057	4561919.521	0.015296589
25.061	4266434.404	0.014305797
25.06483333	4028205.06	0.013506989
25.06883333	3876109.774	0.012996998
25.07266667	3675266.449	0.01232355
25.0765	3507262.57	0.011760215
25.0805	3370281.542	0.011300904
25.08433333	3168786.07	0.010625268
25.08833333	2988425.045	0.010020499
25.09216667	2870912.036	0.009626466
25.09616667	2761142.349	0.009258397
25.1	2692288.662	0.009027523
25.104	2627211.376	0.008809312
25.10783333	2550778.653	0.008553025
25.11166667	2432087.289	0.008155041
25.11566667	2343124.306	0.007856739
25.1195	2266915.11	0.007601201
25.1235	2180967.194	0.007313009
25.12733333	2134102.314	0.007155866
25.13133333	2088577.637	0.007003217
25.13516667	2061834.772	0.006913546
25.13916667	2019969.093	0.006773166
25.143	2002479.439	0.006714521
25.147	1970795.166	0.006608281
25.15083333	1947100.077	0.006528829
25.15483333	1836503.404	0.006157986
25.15866667	1827977.547	0.006129398
25.1625	1750801.379	0.005870619
25.1665	1703874.939	0.005713269
25.17033333	1714454.639	0.005748744

Table 3. Samples Results STD

Retention time (minutes)	Indensity	Percentage of maximum peak area
25.17433333	1653469.915	0.005544256
25.17816667	1655591.068	0.005551369
25.18216667	1647400.571	0.005523905
25.186	1640429.862	0.005500531
25.19	1627510.372	0.005457211
25.19383333	1681489.925	0.00563821
25.19783333	1710325.11	0.005734897
25.20166667	1728396.903	0.005795494
25.2055	1802941.81	0.006045451
25.2095	1843026.706	0.00617986
25.21333333	1883963.92	0.006317126
25.21733333	1891740.152	0.006343201
25.22116667	1865460.962	0.006255084
25.22516667	1819049.652	0.006099462
25.229	1753462.818	0.005879543
25.233	1704117.953	0.005714084
25.23683333	1686604.251	0.005655359
25.24066667	1665526.977	0.005584685
25.24466667	1663936.801	0.005579353
25.2485	1667439.187	0.005591097
25.2525	1655352.324	0.005550568
25.25633333	1608505.386	0.005393485
25.26033333	1583880.751	0.005310916
25.26416667	1578892.282	0.00529419
25.26816667	1544983.364	0.005180489
25.272	1536919.056	0.005153449
25.276	1501373.683	0.005034262
25.27983333	1468073.9	0.004922604
25.28366667	1465886.764	0.00491527
25.28766667	1417709.936	0.004753728
25.2915	1380655.819	0.004629482
25.2955	1346327.123	0.004514374
25.29933333	1357222.512	0.004550908
25.30333333	1338962.776	0.004489681
25.30716667	1339435.836	0.004491267
25.31116667	1280213.685	0.004292689
25.315	1296636.134	0.004347755
25.319	1294941.475	0.004342073
25.32283333	1271704.191	0.004264156
25.32666667	1260721.761	0.004227331
25.33066667	1253074.01	0.004201687
25.3345	1269275.467	0.004256012
25.3385	1256881.876	0.004214455
25.34233333	1238920.559	0.004154229
25.34633333	1263977.132	0.004238246
25.35016667	1277570.412	0.004283826
25.35416667	1275220.401	0.004275946
25.358	1265604.859	0.004243704
25.36183333	1247099.575	0.004181654
25.36583333	1225614.6	0.004109613
25.36966667	1225465.448	0.004109113
25.37366667	1209251.83	0.004054747
25.3775	1185582.881	0.003975382
25.3815	1179319.787	0.003954382
25.38533333	1186581.423	0.003978731
25.38933333	1182730.989	0.00396582
25.39316667	1182716.431	0.003965771
25.397	1208998.903	0.004053899
25.401	1228904.107	0.004120643
25.40483333	1293688.44	0.004337872
25.40883333	1412229.964	0.004735354
25.41266667	1647254.733	0.005523416
25.41666667	2165909.736	0.00726252
25.4205	3079068.729	0.010324437
25.4245	4606065.03	0.015444614
25.42833333	7073117.293	0.023716896
25.43233333	10758879.35	0.036075638
25.43616667	16119626.7	0.05405078
25.44	23487889.99	0.078757331
25.444	33120609.3	0.111056838
25.44783333	44993089.58	0.150866496
25.45183333	59002026.16	0.197839913
25.45566667	74333500.09	0.249247935
25.45966667	89996073.57	0.301766169

Table 3. Samples Results STD

Retention time (minutes)	Indensity	Percentage of maximum peak area
25.4635	104636007.1	0.350855384
25.4675	116978326.4	0.392240461
25.47133333	125419692.2	0.420545236
25.47516667	128947733.3	0.432375124
25.47916667	127169836	0.42641365
25.483	120535612.3	0.404168409
25.487	110089229.5	0.369140605
25.49083333	97200887.88	0.325924658
25.49483333	83208022.66	0.27900513
25.49866667	69661393.8	0.233581878
25.50266667	57434227.46	0.192582922
25.5065	46968614.02	0.157490634
25.5105	38318392.31	0.128485544
25.51433333	31472713.39	0.105531272
25.51816667	26037392.17	0.087306076
25.52216667	21817362.14	0.073155878
25.526	18399240.26	0.061694561
25.53	15775907.93	0.052898256
25.53383333	13652669.07	0.045778815
25.53783333	11907413.1	0.039926791
25.54166667	10472911.7	0.035116759
25.54566667	9285852.825	0.031136428
25.5495	8302094.939	0.027837785
25.5535	7486377.833	0.025102601
25.55733333	6824931.784	0.022884704
25.56116667	6237934.916	0.020916443
25.56516667	5707877.315	0.019139105
25.569	5217100.575	0.017493479
25.573	4795538.169	0.016079937
25.57683333	4437351.775	0.014878901
25.58083333	4169830.3	0.013981873
25.58466667	3906633.262	0.013099347
25.5885	3661177.692	0.012276309
25.5925	3459110.006	0.011598755
25.59633333	3316017.768	0.011118952
25.60033333	3149305.442	0.010559948
25.60416667	3027029.434	0.010149944
25.60816667	2888407.162	0.009685129
25.612	2726256.311	0.00914142
25.616	2592247.039	0.008692073
25.61983333	2549214.374	0.00854778
25.62383333	2531015.005	0.008486756
25.62766667	2436607.391	0.008170197
25.6315	2372639.7	0.007955707
25.6355	2234976.284	0.007494107
25.63933333	2142827.197	0.007185122
25.64333333	2085893.985	0.006994219
25.64716667	2099530.014	0.007039942
25.65116667	2200832.743	0.00737962
25.655	2407913.084	0.008073982
25.659	2758667.841	0.009250099
25.66283333	3419974.797	0.01146753
25.66683333	4538560.046	0.015218263
25.67066667	6499331.809	0.021792934
25.6745	9697383.524	0.032516333
25.6785	14353747.98	0.048129606
25.68233333	21065104.13	0.070633479
25.68633333	30174873.02	0.101179479
25.69016667	41707591	0.139849878
25.69416667	55644823.45	0.186582864
25.698	71312432.41	0.239117982
25.702	87890530.01	0.294706063
25.70583333	103962977.3	0.348598645
25.70966667	117715036.9	0.394710728
25.71366667	127524041.9	0.427601339
25.7175	132065462.7	0.442829194
25.7215	130762125.2	0.438458968
25.72533333	123930904.6	0.415553177
25.72933333	112836323.6	0.378351896
25.73316667	99159972.59	0.332493673
25.73716667	84507873.63	0.283363665
25.741	70364605.09	0.235939819
25.745	57553001.3	0.192981183
25.74883333	46827300.39	0.157016795

Table 3. Samples Results STD

Retention time (minutes)	Indensity	Percentage of maximum peak area
25.75266667	38210213.25	0.128122808
25.75666667	31315646.33	0.10500461
25.7605	25960973.86	0.087049838
25.7645	21782658	0.073039512
25.76833333	18535432.6	0.062151228
25.77233333	15925675.44	0.053400442
25.77616667	13825764.42	0.046359222
25.78	12120160.23	0.040640154
25.784	10711729.15	0.035917539
25.78783333	9547932.242	0.032015207
25.79183333	8536433.849	0.028623548
25.79566667	7697109.348	0.025809206
25.79966667	6969940.941	0.023370935
25.8035	6300727.578	0.021126993
25.8075	5765091.723	0.01933095
25.81133333	5349502.235	0.017937436
25.81516667	5002254.071	0.016773077
25.81916667	4707016.358	0.015783114
25.823	4379445.906	0.014684736
25.827	4167015.056	0.013972434
25.83083333	3956364.885	0.013266102
25.83483333	3871559.728	0.012981741
25.83866667	3674189.545	0.012319939
25.84266667	3509866.999	0.011768948
25.8465	3347499.823	0.011224514
25.8505	3169553.662	0.010627842
25.85433333	3047497.127	0.010218574
25.85833333	2913076.625	0.009767848
25.86216667	2788129.606	0.009348888
25.866	2668398.383	0.008947417
25.87	2557854.647	0.008576752
25.87383333	2495492.736	0.008367646
25.87783333	2426327.219	0.008135727
25.88166667	2344291.479	0.007860652
25.88566667	2237693.217	0.007503217
25.8895	2161033.038	0.007246168
25.8935	2143337.832	0.007186834
25.89733333	2088840.209	0.007004098
25.90116667	2039387.634	0.006838278
25.90516667	2024817.38	0.006789423
25.909	2004771.075	0.006722205
25.913	1977340.164	0.006630227
25.91683333	1939085.906	0.006501956
25.92083333	1890826.99	0.006340139
25.92466667	1868351.676	0.006264777
25.92866667	1880073.369	0.006304081
25.9325	1791812.06	0.006008132
25.9365	1753013.924	0.005878038
25.94033333	1715571.691	0.00575249
25.94416667	1670220.297	0.005600422
25.94816667	1698964.712	0.005696805
25.952	1649152.562	0.00552978
25.956	1617310.197	0.005423009
25.95983333	1617889.761	0.005424952
25.96383333	1572929.868	0.005274197
25.96766667	1552388.909	0.005205321
25.9715	1523845.811	0.005109613
25.9755	1498093.44	0.005023263
25.97933333	1486155.16	0.004983232
25.98333333	1496427.304	0.005017676
25.98716667	1488077.438	0.004989678
25.99116667	1500006.418	0.005029677
25.995	1501360.98	0.005034219
25.999	1490178.996	0.004996725
26.00283333	1442774.82	0.004837774
26.00683333	1419946.791	0.004761229
26.01066667	1388189.951	0.004654745
26.0145	1418457.814	0.004756236
26.0185	1391823.244	0.004666928
26.02233333	1397674.088	0.004686546
26.02633333	1406453.386	0.004715984
26.03016667	1458681.037	0.004891109
26.03416667	1469308.247	0.004926743
26.038	1509032.627	0.005059943

Table 3. Samples Results STD

Retention time (minutes)	Indensity	Percentage of maximum peak area
26.042	1535012.054	0.005147055
26.04583333	1605897.363	0.00538474
26.04966667	1681419.62	0.005637974
26.05366667	1758443.758	0.005896244
26.0575	1799968.796	0.006035482
26.0615	1813332.772	0.006080293
26.06533333	1804304.577	0.00605002
26.06933333	1830652.643	0.006138368
26.07316667	1816353.361	0.006090421
26.07716667	1811378.555	0.00607374
26.081	1768269.87	0.005929192
26.085	1721217.195	0.00577142
26.08883333	1655930.823	0.005552508
26.09266667	1613825.904	0.005411326
26.09666667	1595591.446	0.005350184
26.1005	1517998.442	0.005090006
26.1045	1460726.6	0.004897968
26.10833333	1432516.838	0.004803378
26.11233333	1420974.974	0.004764676
26.11616667	1400288.933	0.004695314
26.12016667	1375659.661	0.00461273
26.124	1370544.956	0.004595579
26.128	1358752.514	0.004556038
26.13183333	1380886.493	0.004630256
26.13566667	1373647.226	0.004605982
26.13966667	1344936.438	0.004509711
26.1435	1357836.834	0.004552968
26.1475	1365061.577	0.004577193
26.15133333	1346482.667	0.004514896
26.15533333	1354494.457	0.00454176
26.15916667	1360385.942	0.004561515
26.16316667	1339411.505	0.004491186
26.167	1350234.819	0.004527477
26.171	1367473.466	0.00458528
26.17483333	1344749.754	0.004509085
26.17866667	1334750.877	0.004475558
26.18266667	1320315	0.004427153
26.1865	1303093.254	0.004369407
26.1905	1303855.642	0.004371963
26.19433333	1293751.472	0.004338083
26.19833333	1282341.111	0.004299823
26.20216667	1268175.588	0.004252324
26.20616667	1270197.598	0.004259104
26.21	1264688.725	0.004240632
26.214	1241474.494	0.004162793
26.21783333	1264085.846	0.004238611
26.22166667	1317570.308	0.00441795
26.22566667	1406597.65	0.004716468
26.2295	1579927.662	0.005297661
26.2335	1916387.149	0.006425845
26.23733333	2475099.994	0.008299267
26.24133333	3566707.301	0.01195954
26.24516667	5401610.24	0.018112159
26.24916667	8273948.823	0.027743409
26.253	12454800.45	0.041762238
26.257	18382933	0.061639881
26.26083333	26298353.66	0.088181107
26.26466667	36437332.99	0.122178157
26.26866667	48563033.03	0.162836887
26.2725	62144596.73	0.208377278
26.2765	76402191.02	0.256184471
26.28033333	90217330.63	0.302508067
26.28433333	102248982.6	0.342851443
26.28816667	111128949.7	0.372626894
26.292	115636758.8	0.387742046
26.296	115275494.7	0.38653069
26.29983333	110387770.6	0.370141644
26.30383333	101922420.2	0.341756446
26.30766667	91136671.76	0.305590713
26.31166667	79408067.21	0.266263485
26.3155	67543665.47	0.226480916
26.3195	56489029.36	0.189413574
26.32333333	46799310.53	0.156922942
26.32733333	38695684.33	0.129750643

Table 3. Samples Results STD

Retention time (minutes)	Indensity	Percentage of maximum peak area
26.33116667	31964027.07	0.107178698
26.335	26580103.84	0.089125845
26.339	22281080.95	0.074710775
26.34283333	18857106.45	0.063229834
26.34683333	16176227.35	0.054240568
26.35066667	14049562.47	0.04710964
26.35466667	12354250.38	0.041425083
26.3585	10921247.75	0.036620076
26.3625	9727972.961	0.032618902
26.36633333	8736217.071	0.029293442
26.37016667	7810567.86	0.026189644
26.37416667	7035960.753	0.023592306
26.378	6364351.456	0.021340331
26.382	5784884.639	0.019397318
26.38583333	5384831.767	0.018055899
26.38983333	5047768.415	0.016925691
26.39366667	4748387.511	0.015921836
26.39766667	4481994.144	0.015028591
26.4015	4266152.373	0.014304851
26.4055	4024961.187	0.013496112
26.40933333	3782442.437	0.012682922
26.41316667	3479664.04	0.011667674
26.41716667	3285785.759	0.01101758
26.421	3208661.303	0.010758974
26.425	3104915.117	0.010411102
26.42883333	2986472.398	0.010013952
26.43283333	2835722.192	0.009508471
26.43666667	2710843.128	0.009089738
26.44066667	2643224.877	0.008863007
26.4445	2508935.513	0.008412721
26.4485	2409102.778	0.008077971
26.45233333	2360746.532	0.007915828
26.45616667	2263550.132	0.007589918
26.46016667	2170121.22	0.007276642
26.464	2083424.403	0.006985938
26.468	2053901.995	0.006886946
26.47183333	2032716.14	0.006815908
26.47583333	2046172.401	0.006861028
26.47966667	2006599.775	0.006728337
26.4835	1981736.525	0.006644968
26.4875	1969326.705	0.006603357
26.49133333	1975176.013	0.00662297
26.49533333	1989991.713	0.006672649
26.49916667	1947221.828	0.006529237
26.50316667	1890293.017	0.006338349
26.507	1873272.566	0.006281277
26.511	1794862.536	0.00601836
26.51483333	1756890.92	0.005891037
26.51883333	1715660.818	0.005752789
26.52266667	1675590.426	0.005618428
26.5265	1654854.673	0.005548899
26.5305	1607290.176	0.005389411
26.53433333	1601064.2	0.005368534
26.53833333	1599061.854	0.00536182
26.54216667	1600099.013	0.005365298
26.54616667	1604882.827	0.005381339
26.55	1614522.455	0.005413661
26.554	1609581.676	0.005397094
26.55783333	1642542.503	0.005507615
26.56166667	1679408.471	0.005631231
26.56566667	1754131.395	0.005881785
26.5695	1720065.667	0.005767559
26.5735	1744005.763	0.005847832
26.57733333	1710536.325	0.005735606
26.58133333	1704780.507	0.005716306
26.58516667	1719622.997	0.005766074
26.58916667	1749569.781	0.005866489
26.593	1746170.317	0.00585509
26.597	1722009.492	0.005774076
26.60083333	1679314.975	0.005630917
26.60466667	1651120.421	0.005536378
26.60866667	1608118.401	0.005392188
26.6125	1561205.104	0.005234883
26.6165	1489852.142	0.004995629

Table 3. Samples Results STD

Retention time (minutes)	Indensity	Percentage of maximum peak area
26.62033333	1461833.534	0.004901679
26.62433333	1467818.807	0.004921749
26.62816667	1455528.516	0.004880538
26.63216667	1465572.011	0.004914215
26.636	1452074.782	0.004868957
26.64	1468271.62	0.004923267
26.64383333	1481782.848	0.004968572
26.64766667	1498658.372	0.005025157
26.65166667	1508962.635	0.005059708
26.6555	1504003.653	0.00504308
26.6595	1532866.273	0.00513986
26.66333333	1620161.115	0.005432568
26.66733333	1649648.263	0.005531442
26.67116667	1701277.569	0.00570456
26.67516667	1708227.741	0.005727865
26.679	1717668.763	0.005759522
26.683	1719047.58	0.005764145
26.68683333	1696597.685	0.005688868
26.69066667	1688746.151	0.005662541
26.69466667	1649148.776	0.005529767
26.6985	1557127.757	0.005221211
26.7025	1467001.549	0.004919008
26.70633333	1467025.468	0.004919089
26.71033333	1419952.832	0.004761249
26.71416667	1405709.415	0.004713489
26.718	1391459.557	0.004665708
26.722	1361858.237	0.004566452
26.72583333	1360282.118	0.004561167
26.72983333	1367408.195	0.004585062
26.73366667	1356264.236	0.004547695
26.73766667	1362779.21	0.00456954
26.7415	1350578.085	0.004528628
26.7455	1300098.388	0.004359365
26.74933333	1293982.627	0.004338858
26.75333333	1283183.46	0.004302647
26.75716667	1252575.517	0.004200016
26.761	1263531.175	0.004236751
26.765	1264516.318	0.004240054
26.76883333	1226685.583	0.004113204
26.77283333	1218955.569	0.004087284
26.77666667	1237799.799	0.004150471
26.78066667	1224297.975	0.004105198
26.78466667	1218115.47	0.004084468
26.7885	1242520.866	0.004166301
26.7925	1281128.44	0.004295757
26.79633333	1396210.26	0.004681638
26.80033333	1658897.13	0.005562454
26.80416667	2115936.468	0.007094954
26.808	2859201.22	0.009587198
26.812	4214767.11	0.014132551
26.81583333	6356359.167	0.021313532
26.81983333	9612644.021	0.032232192
26.82366667	14400612.84	0.048286748
26.82766667	21011980.68	0.070455351
26.8315	29620005.55	0.099318951
26.8355	40180594.03	0.134729699
26.83933333	52426510.41	0.175791527
26.84333333	65669867.35	0.220197876
26.84716667	79182916.78	0.265508533
26.851	91700187.84	0.307480241
26.855	101962810.8	0.34189188
26.85883333	108896081	0.365139854
26.86283333	112014965.7	0.375597798
26.86666667	111049256.5	0.372359675
26.87066667	106338478.5	0.356563947
26.8745	98420579.86	0.330014412
26.8785	88272019.68	0.295985237
26.88233333	77070646.04	0.25842587
26.8865	65900448.11	0.220971038
26.89033333	55481542.77	0.186035367
26.89416667	46198655.64	0.154908884
26.89816667	38377471.49	0.128683642
26.902	31975617.32	0.107217561
26.906	26841052.87	0.090000834

Table 3. Samples Results STD

Retention time (minutes)	Indensity	Percentage of maximum peak area
26.90983333	22695858.74	0.076101568
26.91383333	19362650.22	0.064924974
26.91766667	16666286.44	0.055883787
26.92166667	14384756.78	0.048233581
26.9255	12531104.88	0.042018094
26.9295	10995670.25	0.036869623
26.93333333	9774805.626	0.032775937
26.93716667	8812417.665	0.02954895
26.94116667	7954166.939	0.026671147
26.945	7250756.939	0.02431254
26.949	6581885.356	0.022069744
26.95283333	6058300.226	0.020314109
26.95683333	5576528.627	0.018698679
26.96066667	5205376.042	0.017454166
26.9645	4851437.974	0.016267375
26.9685	4502919.974	0.015098758
26.97233333	4243573.928	0.014229144
26.97633333	4015682.308	0.013464999
26.98016667	3782628.001	0.012683544
26.98416667	3567870.201	0.011963439
26.988	3368078.749	0.011293517
26.992	3162760.866	0.010605065
26.99583333	2975581.971	0.009977435
26.99966667	2833487.605	0.009500978
27.00366667	2748191.214	0.00921497
27.0075	2677283.848	0.00897721
27.0115	2630240.682	0.00881947
27.01533333	2545519.738	0.008535392
27.01933333	2460205.553	0.008249324
27.02316667	2394939.274	0.00803048
27.02716667	2314382.573	0.007760365
27.031	2187859.546	0.00733612
27.035	2130867.691	0.00714502
27.03883333	2127067.16	0.007132277
27.04266667	2119890.335	0.007108212
27.04666667	2067330.373	0.006931973
27.0505	2034941.7	0.006823371
27.0545	1990417.169	0.006674075
27.05833333	1942644.287	0.006513888
27.06233333	1898782.217	0.006366814
27.06616667	1863245.306	0.006247655
27.07016667	1803627.064	0.006047749
27.074	1759830.662	0.005900895
27.078	1751089.382	0.005871584
27.08183333	1725861.891	0.005786994
27.08566667	1687609.113	0.005658728
27.08966667	1676067.045	0.005620027
27.0935	1631180.522	0.005469517
27.0975	1578813.201	0.005293924
27.10133333	1580137.659	0.005298365
27.10533333	1557979.666	0.005224067
27.10916667	1571700.316	0.005270074
27.11316667	1571900.031	0.005270744
27.117	1566169.619	0.005251529
27.121	1569919.35	0.005264102
27.12483333	1536546.799	0.005152201
27.12866667	1490179.526	0.004996727
27.13266667	1431969.814	0.004801543
27.1365	1407726.654	0.004720253
27.1405	1418981.7	0.004757993
27.14433333	1439228.011	0.004825881
27.14833333	1465678.353	0.004914572
27.15216667	1468435.922	0.004923818
27.156	1458852.146	0.004891683
27.16	1471632.529	0.004934537
27.16383333	1509239.313	0.005060636
27.16783333	1563139.727	0.00524137
27.17166667	1621321.601	0.005436459
27.17566667	1790375.068	0.006003313
27.1795	2116133.671	0.007095616
27.1835	2721140.841	0.009124267
27.18733333	3719621.813	0.012472278
27.19133333	5344547.613	0.017920822
27.19516667	7750967.161	0.025989797

Table 3. Samples Results STD

Retention time (minutes)	Indensity	Percentage of maximum peak area
27.199	11183473.43	0.037499346
27.203	15719554.73	0.052709297
27.20683333	21774285.06	0.073011436
27.21083333	29388832.37	0.098543803
27.21466667	38665528.9	0.129649529
27.21866667	49567907.74	0.166206336
27.2225	61949658.39	0.20772363
27.2265	75315603.66	0.252541031
27.23033333	89602726.66	0.300447236
27.23433333	103700862.6	0.347719748
27.23816667	116784520.1	0.391590608
27.242	126940893.8	0.425645983
27.246	134000690.9	0.449318215
27.24983333	136435956.1	0.457483912
27.25383333	133896589.8	0.448969153
27.25766667	126097366.1	0.422817547
27.26166667	114430971	0.383698914
27.2655	99911802.66	0.335014637
27.2695	84004143.05	0.281674604
27.27333333	68728314.16	0.230453166
27.27716667	55338402.18	0.185555402
27.28116667	44212877.42	0.148250364
27.285	35421184.92	0.118770907
27.289	28688006.85	0.096193862
27.29283333	23504273.96	0.078812268
27.29683333	19515475.03	0.065437412
27.30066667	16504645.13	0.055341787
27.30466667	14194574.99	0.047595882
27.3085	12324020.39	0.041323719
27.3125	10834304.22	0.036328546
27.31633333	9666185.606	0.032411723
27.32016667	8678180.517	0.029098839
27.32416667	7851394.674	0.026326541
27.328	7147806.412	0.023967336
27.332	6522065.01	0.02186916
27.33583333	6057401.118	0.020311094
27.33983333	5581768.38	0.018716248
27.34366667	5162369.213	0.017309959
27.3475	4832050.117	0.016202365
27.3515	4503241.627	0.015099836
27.35533333	4207025.347	0.014106592
27.35933333	3965046.102	0.013295211
27.36316667	3787726.237	0.012700639
27.36716667	3643318.2	0.012216424
27.371	3465114.079	0.011618887
27.375	3263849.058	0.010944024
27.37883333	3152558.243	0.010570855
27.38283333	3032598.712	0.010168618
27.38666667	2895504.777	0.009708928
27.3905	2798921.112	0.009385073
27.3945	2721533.274	0.009125583
27.39833333	2655601.681	0.008904508
27.40233333	2639404.414	0.008850197
27.40616667	2490919.142	0.00835231
27.41016667	2386200.994	0.008001179
27.414	2296194.237	0.007699377
27.418	2223657.459	0.007456154
27.42183333	2202598.051	0.00738554
27.42583333	2148772.723	0.007205058
27.42966667	2071817.296	0.006947018
27.4335	2007346.513	0.006730841
27.4375	1961388.87	0.00657674
27.44133333	1952485.251	0.006546886
27.44533333	1900572.521	0.006372817
27.44916667	1868473.635	0.006265186
27.45316667	1841568.187	0.006174969
27.457	1772019.372	0.005941765
27.461	1728092.756	0.005794474
27.46483333	1724028.846	0.005780848
27.46883333	1701597.763	0.005705634
27.47266667	1713916.112	0.005746938
27.4765	1702672.996	0.005709239
27.4805	1672298.035	0.005607389
27.48433333	1631582.287	0.005470865

Table 3. Samples Results STD

Retention time (minutes)	Indensity	Percentage of maximum peak area
27.48833333	1617457.328	0.005423502
27.49216667	1570152.306	0.005264884
27.49616667	1573798.088	0.005277108
27.5	1567261.077	0.005255189
27.504	1533758.57	0.005142852
27.50783333	1527786.07	0.005122825
27.51166667	1525910.731	0.005116537
27.51566667	1526382.573	0.005118119
27.5195	1505614.637	0.005048482
27.5235	1452485.26	0.004870334
27.52733333	1445073.083	0.00484548
27.53133333	1485980.426	0.004982646
27.53516667	1499013.53	0.005026348
27.53916667	1518718.898	0.005092422
27.543	1480067.897	0.004962821
27.547	1507277.406	0.005054057
27.55083333	1473799.711	0.004941803
27.55466667	1468296.996	0.004923352
27.55866667	1462577.676	0.004904175
27.5625	1492911.538	0.005005887
27.5665	1497431.483	0.005021043
27.57033333	1483828.493	0.004975431
27.57433333	1432248.87	0.004802479
27.57816667	1408254.162	0.004722022
27.58216667	1426593.446	0.004783516
27.586	1416419.025	0.0047494
27.59	1405079.185	0.004711376
27.59383333	1380521.879	0.004629033
27.59766667	1338429.512	0.004487893
27.60166667	1312946.46	0.004402446
27.6055	1343189.055	0.004503852
27.6095	1344021.94	0.004506645
27.61333333	1331065.696	0.004463201
27.61733333	1312912.056	0.00440233
27.62116667	1283772.61	0.004304623
27.625	1258160.42	0.004218742
27.629	1244819.001	0.004174007
27.63283333	1225294.863	0.004108541
27.63683333	1249022.401	0.004188102
27.64066667	1275076.577	0.004275464
27.64466667	1266799.56	0.00424771
27.6485	1261844.352	0.004231095
27.6525	1238396.251	0.004152471
27.65633333	1213466.719	0.00406888
27.66033333	1175732.37	0.003942353
27.66416667	1190656.996	0.003992396
27.668	1211235.611	0.004061399
27.672	1227543.122	0.004116079
27.67583333	1194228.746	0.004004373
27.67983333	1200847.376	0.004026566
27.68366667	1209219.105	0.004054637
27.68766667	1270192.255	0.004259086
27.6915	1334383.732	0.004474327
27.6955	1418904.617	0.004757734
27.69933333	1602422.8	0.00537309
27.70316667	1947038.016	0.00652862
27.70716667	2566824.604	0.008606829
27.711	3584129.224	0.012017957
27.715	5165973.633	0.017322045
27.71883333	7679900.322	0.025751502
27.72283333	11478688.98	0.038489235
27.72666667	16816363.58	0.056387011
27.73066667	23785014.91	0.079753622
27.7345	32619808.73	0.109377602
27.7385	43176874.22	0.144776537
27.74233333	55222164.21	0.185165644
27.74633333	68035669.79	0.228130657
27.75016667	80782683.01	0.270872715
27.754	92425125.35	0.309911031
27.758	101738430.3	0.341139509
27.76183333	107756599.9	0.361319056
27.76583333	110444077.3	0.370330446
27.76966667	109562239.9	0.367373554
27.77366667	105109707.8	0.352443752

Table 3. Samples Results STD

Retention time (minutes)	Indensity	Percentage of maximum peak area
27.7775	97820336.48	0.328001734
27.7815	88663979.21	0.297299518
27.78533333	78598229.94	0.263548017
27.78916667	68396299.72	0.229339887
27.79316667	58702188	0.196834525
27.797	49939016.88	0.167450703
27.801	42159564.76	0.141365393
27.80483333	35519753.14	0.119101416
27.80883333	30075558.03	0.100846465
27.81266667	25604350.43	0.085854043
27.8165	21924988.64	0.073516761
27.8205	18953056.05	0.063551563
27.82433333	16364837.67	0.054872998
27.82833333	14200015.11	0.047614123
27.83216667	12528991.75	0.042011009
27.83616667	11243767.27	0.037701518
27.84	10183555.08	0.034146516
27.844	9289212.691	0.031147694
27.84783333	8437439.47	0.02829161
27.85183333	7619777.364	0.025549904
27.85566667	6918467.472	0.023198339
27.8595	6310771.923	0.021160673
27.8635	5782697.754	0.019389985
27.86733333	5361543.848	0.017977813
27.87133333	4940085.056	0.016564618
27.87516667	4616087.76	0.015478221
27.87916667	4297424.978	0.014409712
27.883	4039655.799	0.013545385
27.887	3787168.602	0.012698769
27.89083333	3595622.14	0.012056494
27.89466667	3460535.281	0.011603534
27.89866667	3357805.231	0.011259069
27.9025	3172758.776	0.010638589
27.9065	3031483.202	0.010164878
27.91033333	2901206.518	0.009728046
27.91433333	2792944.406	0.009365032
27.91816667	2699331.964	0.00905114
27.92216667	2610389.557	0.008752907
27.926	2582695.881	0.008660047
27.93	2436824.196	0.008170924
27.93383333	2337458.958	0.007837742
27.93783333	2266749.789	0.007600647
27.94166667	2208337.332	0.007404784
27.9455	2162267.483	0.007250307
27.9495	2106546.754	0.00706347
27.95333333	2095626.729	0.007026854
27.95733333	2051337.074	0.006878346
27.96116667	2042085.101	0.006847323
27.96516667	1988083.29	0.006666249
27.969	1945581.875	0.006523738
27.973	1875161.776	0.006287612
27.97683333	1894857.652	0.006353654
27.98083333	1920691.478	0.006440278
27.98466667	2017857.943	0.006766087
27.9885	2147624.091	0.007201206
27.9925	2369697.411	0.007945841
27.99633333	2791278.689	0.009359447
28.00033333	3584741.24	0.012020009
28.00416667	4841576.658	0.016234309
28.00816667	6881783.896	0.023075335
28.012	9928937.946	0.033292759
28.016	14210541.85	0.047649421
28.01983333	19992483.57	0.067036871
28.02366667	27613896.44	0.092592259
28.02766667	37050672.4	0.124234747
28.0315	48387584.2	0.162248588
28.0355	61153401.75	0.205053699
28.03933333	75026934.61	0.251573093
28.04333333	89217484.29	0.299155478
28.04716667	102816757.4	0.344755252
28.051	114890669.6	0.385240331
28.055	123899764.2	0.41544876
28.05883333	129125145.8	0.432970007
28.06283333	129718351.1	0.434959085

Table 3. Samples Results STD

Retention time (minutes)	Indensity	Percentage of maximum peak area
28.06666667	125385640.3	0.420431057
28.07066667	116757320.8	0.391499406
28.0745	105004221.3	0.352090045
28.0785	91623287.17	0.307222385
28.0825	78057448.21	0.26173472
28.08633333	65356266.03	0.219146339
28.09033333	54072819.73	0.181311773
28.09416667	44356252.47	0.148731115
28.09816667	36325521.87	0.121803242
28.102	29921457.25	0.10032975
28.106	24914427.51	0.083540659
28.10983333	20875955.42	0.069999244
28.11366667	17774292.54	0.059599046
28.11766667	15330224.31	0.051403832
28.1215	13374719.02	0.04484682
28.1255	11816365.21	0.039621498
28.12933333	10634227.55	0.035657668
28.13333333	9593395.697	0.032167651
28.13716667	8712957.824	0.029215451
28.141	8002984.817	0.026834838
28.145	7307546.185	0.02450296
28.149	6695870.074	0.022451947
28.15283333	6145764.486	0.020607386
28.15666667	5739888.291	0.019246441
28.16066667	5411062.215	0.018143853
28.1645	5020455.285	0.016834107
28.1685	4723842.8	0.015839535
28.17233333	4460946.948	0.014958018
28.17633333	4237514.527	0.014208826
28.18016667	4022846.737	0.013489022
28.184	3880126.085	0.013010465
28.188	3796454.084	0.012729904
28.19183333	3841579.211	0.012881214
28.19583333	4030041.757	0.013513148
28.19966667	4465788.26	0.014974251
28.20366667	5245141.183	0.017587502
28.2075	6604218.221	0.022144629
28.2115	8712986.157	0.029215546
28.21533333	11948586.9	0.040064851
28.21933333	16533144.39	0.055437348
28.22316667	22716144.37	0.076169588
28.227	30579655.53	0.102536757
28.231	39952887.56	0.133966176
28.23483333	50772491.21	0.170245429
28.23883333	62521857.69	0.209642273
28.24266667	74540819.29	0.249943098
28.24666667	85838706.55	0.287826086
28.2505	95533354.89	0.320333247
28.2545	102760006.5	0.34456496
28.25833333	106907575	0.358472187
28.26233333	107501948.4	0.360465183
28.26616667	104596708.7	0.350723613
28.27016667	98732268.18	0.331059535
28.274	90626148.61	0.303878876
28.278	81144120.39	0.272084652
28.28183333	71133082.9	0.238516605
28.28566667	61432400.45	0.20598921
28.28966667	52458913.51	0.175900178
28.2935	44537311.11	0.149338223
28.2975	37858721.4	0.126944219
28.30133333	32243489.04	0.108115763
28.30533333	27524504.57	0.092292519
28.30916667	23504312.86	0.078812399
28.313	20205160.19	0.067749998
28.317	17687519.56	0.059308088
28.32083333	15607661.57	0.052334108
28.32483333	13861216.17	0.046478095
28.32866667	12331166.36	0.04134768
28.33266667	11056523.96	0.037073672
28.3365	9966125.742	0.033417453
28.3405	9079161.312	0.03044337
28.34433333	8275407.449	0.027748299
28.34833333	7582083.039	0.025423511
28.35216667	6940625.305	0.023272637

Table 3. Samples Results STD

Retention time (minutes)	Indensity	Percentage of maximum peak area
28.356	6375318.386	0.021377104
28.36	5863506.729	0.019660946
28.36383333	5421917.148	0.018180251
28.36783333	5071264.917	0.017004477
28.37166667	4744374.745	0.015908381
28.37566667	4485562.652	0.015040557
28.3795	4293285.307	0.014395831
28.3835	4150302.815	0.013916396
28.38733333	4153426.632	0.01392687
28.39116667	4273665.498	0.014330044
28.39516667	4638696.163	0.015554029
28.399	5308963.533	0.017801505
28.403	6453796.429	0.021640249
28.40683333	8189403.323	0.027459919
28.41083333	10733486.35	0.035990493
28.41466667	14165526.76	0.04749848
28.41866667	18679119.88	0.062633026
28.4225	24467266.84	0.082041283
28.4265	31554134.24	0.105804285
28.43033333	40086154.94	0.134413035
28.43416667	50019178.53	0.167719493
28.43816667	61353284.29	0.205723926
28.442	73661926.95	0.246996081
28.446	86621855.1	0.290452064
28.44983333	99531677.59	0.333740038
28.45383333	111502318	0.373878837
28.45766667	121627163.7	0.407828495
28.46166667	128845572.8	0.432032569
28.4655	132425821.4	0.444037514
28.4695	131769897.1	0.441838132
28.47333333	126202479.3	0.423170002
28.47716667	116389547.6	0.390266225
28.48116667	102989933.6	0.345335929
28.485	88255957.02	0.295931378
28.489	73193673.7	0.245425979
28.49283333	59706940.92	0.200203566
28.49683333	47993042.13	0.160925648
28.50066667	38568170.55	0.129323076
28.50466667	31134852.28	0.104398389
28.5085	25553588.98	0.085683834
28.5125	20961254.26	0.07028526
28.51633333	17787518.4	0.059643394
28.52016667	15260444.59	0.051169853
28.52416667	13193163.82	0.044238047
28.528	11530274.09	0.038662205
28.532	10411252.61	0.03491001
28.53583333	9418394.725	0.031580854
28.53983333	8574069.747	0.028749745
28.54366667	7822344.506	0.026229132
28.5475	7283149.112	0.024421154
28.5515	6689071.178	0.022429149
28.55533333	6185211.511	0.020739656
28.55933333	5725258.252	0.019197385
28.56333333	5432757.215	0.018216598
28.56716667	5061832.539	0.01697285
28.57116667	4834461.147	0.01621045
28.575	4617893.826	0.015484277
28.579	4396900.064	0.014743262
28.58283333	4219685.431	0.014149043
28.58666667	4045292.312	0.013564285
28.59066667	3824277.484	0.012823199
28.5945	3694226.158	0.012387123
28.5985	3560506.566	0.011938748
28.60233333	3376530.957	0.011321859
28.60633333	3225071.713	0.010814
28.61016667	3142361.281	0.010536663
28.61416667	3037635.818	0.010185508
28.618	2940459.306	0.009859665
28.622	2849517.527	0.009554728
28.62583333	2754810.774	0.009237166
28.62966667	2671580.544	0.008958087
28.63366667	2610731.369	0.008754053
28.6375	2578768.028	0.008646877
28.6415	2497244.246	0.008373519

Table 3. Samples Results STD

Retention time (minutes)	Indensity	Percentage of maximum peak area
28.64533333	2422648.17	0.008123391
28.64933333	2333371.21	0.007824036
28.65316667	2282587.301	0.007653752
28.65716667	2251921.216	0.007550925
28.661	2238922.811	0.00750734
28.665	2218743.26	0.007439676
28.66883333	2144789.714	0.007191702
28.67266667	2069185.176	0.006938193
28.67666667	2066927.125	0.006930621
28.6805	2069966.865	0.006940814
28.6845	2031897.035	0.006813161
28.68833333	1981648.502	0.006644673
28.69233333	1979262.052	0.006636671
28.69616667	1875860.325	0.006289954
28.7	1839031.065	0.006166462
28.704	1829146.588	0.006133318
28.70783333	1770708.726	0.00593737
28.71183333	1770556.34	0.005936859
28.71566667	1741231.97	0.005838531
28.71966667	1721096.909	0.005771016
28.7235	1706691.278	0.005722713
28.7275	1689541.06	0.005665206
28.73133333	1689373.973	0.005664646
28.73533333	1647020.988	0.005522632
28.73916667	1673848.226	0.005612587
28.743	1634300.379	0.005479979
28.747	1632657.638	0.00547447
28.75083333	1617318.267	0.005423036
28.75483333	1612868.467	0.005408115
28.75866667	1609693.203	0.005397468
28.76266667	1543003.348	0.00517385
28.7665	1516202.56	0.005083984
28.7705	1511492.206	0.00506819
28.77433333	1474476.095	0.004944071
28.77816667	1512383.636	0.005071179
28.78216667	1524749.839	0.005112644
28.786	1493934.878	0.005009319
28.79	1492593.719	0.005004822
28.79383333	1489340.733	0.004993914
28.79783333	1466250.361	0.00491649
28.80166667	1466837.35	0.004918458
28.80566667	1441062.831	0.004832033
28.8095	1449784.01	0.004861276
28.8135	1432872.189	0.004804569
28.81733333	1440966.366	0.00483171
28.82116667	1394740.454	0.004676709
28.82516667	1383517.947	0.004639079
28.829	1397986.127	0.004687592
28.833	1407731.728	0.00472027
28.83683333	1356844.027	0.004549639
28.84083333	1351077.269	0.004530302
28.84466667	1349361.788	0.00452455
28.84866667	1357999.736	0.004553514
28.8525	1374243.883	0.004607982
28.8565	1363771.944	0.004572869
28.86033333	1358239.413	0.004554318
28.86416667	1362178.68	0.004567526
28.86816667	1359885.603	0.004559837
28.872	1349320.7	0.004524412
28.876	1299027.506	0.004355774
28.87983333	1267709.702	0.004250762
28.88383333	1260635.634	0.004227042
28.88766667	1295410.576	0.004343646
28.89166667	1324400.996	0.004440854
28.8955	1363487.111	0.004571914
28.8995	1343775.128	0.004505817
28.90333333	1311195.128	0.004396573
28.90716667	1261631.082	0.00423038
28.91116667	1249739.685	0.004190507
28.915	1263138.677	0.004235435
28.919	1266577.021	0.004246964
28.92283333	1255786.1	0.004210781
28.92683333	1274240.561	0.004272661
28.93066667	1274149.359	0.004272355

Table 3. Samples Results STD

Retention time (minutes)	Indensity	Percentage of maximum peak area
28.9345	1284579.384	0.004307328
28.9385	1272356.273	0.004266343
28.9425	1260760.846	0.004227462
28.94633333	1272663.542	0.004267373
28.95016667	1278390.062	0.004286574
28.95416667	1239236.341	0.004155288
28.958	1249041.22	0.004188165
28.962	1260382.907	0.004226195
28.96583333	1271679.104	0.004264072
28.96983333	1269262.621	0.004255969
28.97366667	1292411.158	0.004333589
28.9775	1310906.813	0.004395607
28.9815	1331436.916	0.004464446
28.98533333	1315630.451	0.004411445
28.98933333	1327075.623	0.004449822
28.99316667	1290510.865	0.004327217
28.99716667	1270098.497	0.004258772
29.001	1276810.011	0.004281276
29.005	1307489.881	0.004384149
29.00883333	1293393.16	0.004336881
29.01283333	1286206.067	0.004312782
29.01666667	1299718.126	0.00435809
29.0205	1344157.765	0.0045071
29.0245	1351113.023	0.004530422
29.02833333	1355669.398	0.0045457
29.03233333	1364435.636	0.004575094
29.03616667	1390153.707	0.00466133
29.04016667	1375982.949	0.004613814
29.044	1335349.977	0.004477567
29.048	1312643.576	0.00440143
29.05183333	1285583.253	0.004310694
29.05583333	1286242.162	0.004312903
29.05966667	1314462.667	0.00440753
29.0635	1281555.026	0.004297187
29.0675	1269926.534	0.004258195
29.07133333	1247303.698	0.004182339
29.07533333	1227409.536	0.004115631
29.07916667	1215325.507	0.004075112
29.08316667	1227488.204	0.004115895
29.087	1212493.039	0.004065615
29.091	1211676.016	0.004062875
29.09483333	1219922.883	0.004090528
29.09866667	1224773.269	0.004106792
29.10266667	1225382.385	0.004108834
29.1065	1226058.093	0.0041111
29.1105	1216489.535	0.004079016
29.11433333	1244685.834	0.004173561
29.11833333	1237440.833	0.004149267
29.12216667	1271496.492	0.00426346
29.12616667	1302713.705	0.004368134
29.13	1303285.426	0.004370051
29.134	1278766.31	0.004287836
29.13783333	1242890.778	0.004167542
29.14166667	1216863.718	0.00408027
29.14566667	1230742.041	0.004126806
29.1495	1217424.028	0.004082149
29.1535	1184732.305	0.00397253
29.15733333	1149044.036	0.003852864
29.16133333	1132787.911	0.003798355
29.16516667	1113664.03	0.003734231
29.16916667	1118978.707	0.003752052
29.173	1101274.573	0.003692688
29.177	1096872.071	0.003677926
29.18083333	1098813.401	0.003684435
29.18466667	1133248.129	0.003799899
29.18866667	1121354.47	0.003760018
29.1925	1144574.251	0.003837876
29.1965	1141059.278	0.00382609
29.20033333	1121976.751	0.003762104
29.20433333	1099349.516	0.003686233
29.20816667	1078436.943	0.003616111
29.21216667	1074202.609	0.003601913
29.216	1088974.424	0.003651444
29.22	1067120.646	0.003578166

Table 3. Samples Results STD

Retention time (minutes)	Indensity	Percentage of maximum peak area
29.22383333	1060440.901	0.003555768
29.22766667	1068447.455	0.003582615
29.23166667	1058445.712	0.003549078
29.2355	1076927.609	0.00361105
29.2395	1101592.197	0.003693753
29.24333333	1156014.252	0.003876236
29.24733333	1183468.198	0.003968292
29.25116667	1239183.635	0.004155111
29.25516667	1363916.441	0.004573353
29.259	1571677.599	0.005269998
29.263	1851421.458	0.006208008
29.26683333	2357508.34	0.00790497
29.27066667	3137719.162	0.010521098
29.27466667	4331808.242	0.014525002
29.2785	6265127.345	0.021007622
29.2825	9067225.377	0.030403347
29.28633333	13057385.17	0.043782767
29.29033333	18116244.18	0.060745646
29.29416667	24393637.49	0.081794397
29.29816667	32135791	0.10775464
29.302	41539192.67	0.139285221
29.306	52126833.17	0.174786678
29.30983333	63275230.07	0.212168409
29.31366667	74415106.88	0.249521571
29.31766667	84909726.32	0.284711119
29.3215	93786061.74	0.314474392
29.3255	100177152.4	0.335904382
29.32933333	103594956.9	0.347364635
29.33333333	103756268.4	0.347905529
29.33716667	100689496.3	0.337622324
29.341	94883223.16	0.318153288
29.345	87131015.65	0.292159332
29.34883333	78086211.85	0.261831167
29.35283333	68363113.6	0.22922861
29.35666667	58792372.44	0.197136923
29.36066667	49894763.85	0.167302318
29.3645	42048750.19	0.140993821
29.3685	35435022.05	0.118817304
29.37233333	29906529.35	0.100279695
29.37616667	25332184.78	0.084941443
29.38016667	21582580.85	0.072368632
29.38416667	18591950.81	0.062340739
29.38816667	16224618.51	0.054402829
29.392	14098095.23	0.047272376
29.396	12468485.36	0.041808125
29.39983333	11094586.22	0.037201298
29.40366667	9938601.057	0.03332516
29.40766667	8948950.928	0.030006761
29.4115	8099633.364	0.027158911
29.4155	7375253.13	0.024729989
29.41933333	6769948.759	0.02270034
29.42333333	6203898.338	0.020802315
29.42716667	5741013.512	0.019250214
29.431	5315329.502	0.017822851
29.435	4931880.967	0.016537108
29.43883333	4552852.909	0.015266188
29.44283333	4233400.29	0.01419503
29.44666667	4032049.581	0.01351988
29.45066667	3871128.921	0.012980297
29.4545	3658761.09	0.012268205
29.4585	3458622.211	0.011597119
29.46233333	3268879.732	0.010960893
29.46616667	3121472.337	0.01046662
29.47016667	3024608.804	0.010141827
29.474	2925595.804	0.009809826
29.478	2834669.889	0.009504942
29.48183333	2728234.038	0.009148052
29.48583333	2579314.793	0.00864871
29.48966667	2504889.386	0.008399154
29.49366667	2379525.826	0.007978797
29.4975	2346006.448	0.007866403
29.5015	2295580.155	0.007697318
29.50533333	2232152.128	0.007484638
29.50933333	2153362.939	0.007220449

Table 3. Samples Results STD

Retention time (minutes)	Indensity	Percentage of maximum peak area
29.51316667	2065581.974	0.006926111
29.517	2013577.301	0.006751734
29.521	1959054.859	0.006568914
29.52483333	1945039.646	0.00652192
29.52883333	1944258.305	0.0065193
29.53266667	1937377.507	0.006496228
29.53666667	1887221.75	0.00632805
29.5405	1831375.399	0.006140792
29.5445	1761815.974	0.005907552
29.54833333	1714833.11	0.005750013
29.55216667	1661888.081	0.005572483
29.55616667	1657524.302	0.005557851
29.56	1632215.244	0.005472987
29.564	1622452.061	0.00544025
29.56783333	1569399.062	0.005262358
29.57183333	1575664.628	0.005283367
29.57566667	1596016.285	0.005351608
29.5795	1589264.951	0.00532897
29.5835	1583761.364	0.005310516
29.58733333	1551498.5	0.005202335
29.59133333	1516061.18	0.00508351
29.59516667	1537361.908	0.005154934
29.59916667	1509774.106	0.005062429
29.603	1519030.014	0.005093465
29.607	1478431.817	0.004957335
29.61083333	1477134.922	0.004952987
29.61483333	1468553.036	0.004924211
29.61866667	1444381.78	0.004843162
29.6225	1454101.288	0.004875752
29.6265	1451987.113	0.004868663
29.63033333	1417308.823	0.004752383
29.63433333	1425605.813	0.004780204
29.63816667	1448519.954	0.004857038
29.64216667	1417707.626	0.004753721
29.646	1370015.586	0.004593804
29.65	1350188.325	0.004527321
29.65383333	1339150.806	0.004490312
29.65783333	1347966.339	0.004519871
29.66166667	1367852.955	0.004586553
29.6655	1411153.272	0.004731743
29.6695	1480950.871	0.004965782
29.67333333	1524481.872	0.005111746
29.67733333	1672311.478	0.005607434
29.68116667	1957064.277	0.00656224
29.68516667	2461163.166	0.008252535
29.689	3187540.886	0.010688155
29.693	4308659.49	0.014447382
29.69683333	5832289.732	0.019556272
29.70083333	7835457.307	0.026273101
29.70466667	10499581.89	0.035206187
29.7085	13970694.94	0.046845189
29.7125	18283505.22	0.061306489
29.71633333	23386847.63	0.078418526
29.72033333	29439084.26	0.098712303
29.72416667	36511752.75	0.122427694
29.72816667	44367201.46	0.148767828
29.73216667	52879883.27	0.177311733
29.736	62024772.56	0.207975495
29.74	71702997.58	0.240427588
29.74383333	81466365.35	0.273165172
29.74783333	91507968.31	0.306835709
29.75166667	101374527.6	0.339919305
29.7555	110069864.4	0.369075671
29.7595	116627885.1	0.391065395
29.76333333	120429841.1	0.403813748
29.76733333	120909617.2	0.405422487
29.77116667	117561934.1	0.394197358
29.77516667	110789741.2	0.371489493
29.779	100608307.3	0.337350089
29.783	88492137.32	0.296723315
29.78683333	75402165.31	0.252831281
29.79066667	62447739.1	0.209393746
29.79466667	50932374.72	0.170781535
29.7985	41197882.66	0.138140773

Table 3. Samples Results STD

Retention time (minutes)	Indensity	Percentage of maximum peak area
29.8025	33395278.44	0.111977832
29.80633333	27390043.96	0.091841658
29.81033333	22666201.64	0.076002125
29.81416667	19119485.06	0.064109616
29.81816667	16339251.69	0.054787206
29.822	14117340.39	0.047336907
29.826	12432981.64	0.041689077
29.82983333	11048654.54	0.037047285
29.83366667	10001343.53	0.033535542
29.83766667	9098273.683	0.030507455
29.8415	8300722.073	0.027833182
29.8455	7593142.857	0.025460596
29.84933333	7004723.509	0.023487564
29.85333333	6493403.701	0.021773056
29.85716667	6083583.117	0.020398885
29.861	5713451.164	0.019157794
29.865	5307866.443	0.017797827
29.86883333	5043883.218	0.016912664
29.87283333	4730844.737	0.015863013
29.87666667	4445443.223	0.014906032
29.88066667	4236443.364	0.014205234
29.8845	4037377.272	0.013537745
29.8885	3802659.659	0.012750712
29.89233333	3640342.888	0.012206447
29.89633333	3422330.713	0.01147543
29.90016667	3247411.463	0.010888907
29.904	3128445.49	0.010490002
29.908	2980317.204	0.009993313
29.91183333	2866873.219	0.009612923
29.91583333	2789512.257	0.009353524
29.91966667	2706381.514	0.009074778
29.92366667	2639467.035	0.008850407
29.9275	2588490.331	0.008679477
29.9315	2552277.86	0.008558052
29.93533333	2484986.061	0.008332416
29.93933333	2394320.063	0.008028404
29.94316667	2285659.086	0.007664052
29.947	2222974.123	0.007453863
29.951	2176212.035	0.007297065
29.95483333	2152621.578	0.007217963
29.95883333	2104732.053	0.007057385
29.96266667	2090028.197	0.007008081
29.96666667	2051232.924	0.006877997
29.9705	2059764.153	0.006906603
29.9745	1997761.616	0.006698702
29.97833333	1985721.913	0.006658332
29.98216667	1927123.564	0.006461845
29.98616667	1883051.427	0.006314067
29.99	1852051.365	0.00621012
29.994	1870662.909	0.006272527
29.99783333	1875853.142	0.00628993
30.00183333	1875586.678	0.006289037
30.00566667	1836549.062	0.006158139
30.00966667	1769172.548	0.005932219
30.0135	1704614.785	0.00571575
30.0175	1649563.535	0.005531158
30.02133333	1627106.18	0.005455856
30.02516667	1626969.332	0.005455397
30.02916667	1653999.113	0.005546031
30.033	1611287.914	0.005402815
30.037	1608260.596	0.005392665
30.04083333	1588794.501	0.005327393
30.04483333	1558106.101	0.005224491
30.04866667	1522568.565	0.00510533
30.05266667	1561316.926	0.005235258
30.0565	1511155.772	0.005067062
30.0605	1480127.228	0.00496302
30.06433333	1469959.632	0.004928927
30.06816667	1486683.74	0.004985005
30.07216667	1434071.724	0.004808591
30.076	1389983.288	0.004660758
30.08	1361489.168	0.004565214
30.08383333	1365847.723	0.004579829
30.08783333	1357010.462	0.004550197

Table 3. Samples Results STD

Retention time (minutes)	Indensity	Percentage of maximum peak area
30.09166667	1411358.861	0.004732433
30.09566667	1431635.694	0.004800423
30.0995	1386461.419	0.004648949
30.1035	1368089.078	0.004587345
30.10733333	1366628.353	0.004582447
30.11116667	1344266.212	0.004507464
30.11516667	1296354.207	0.00434681
30.119	1281720.461	0.004297742
30.123	1295333.024	0.004343386
30.12683333	1295540.002	0.00434408
30.13083333	1295643.173	0.004344426
30.13466667	1311947.975	0.004399098
30.1385	1292173.641	0.004332792
30.1425	1271691.21	0.004264113
30.14633333	1260515.652	0.00422664
30.15033333	1263035.223	0.004235088
30.15416667	1267470.423	0.00424996
30.15816667	1259045.446	0.00422171
30.162	1264161.864	0.004238866
30.166	1251955.261	0.004197936
30.16983333	1283826.594	0.004304804
30.17383333	1275352.14	0.004276388
30.17766667	1269128.032	0.004255518
30.1815	1249517.93	0.004189763
30.1855	1229006.925	0.004120988
30.18933333	1201721.431	0.004029497
30.19333333	1219473.99	0.004089023
30.19716667	1231914.645	0.004130738
30.20116667	1216531.904	0.004079158
30.205	1197297.758	0.004014664
30.209	1172811.191	0.003932558
30.21283333	1156925.673	0.003879292
30.21683333	1142306.955	0.003830274
30.22066667	1157631.189	0.003881657
30.2245	1161766.925	0.003895525
30.2285	1179679.433	0.003955587
30.23233333	1168024.446	0.003916507
30.23633333	1152427.392	0.003864209
30.24016667	1160457.734	0.003891135
30.24416667	1163949.232	0.003902842
30.248	1185481.846	0.003975044
30.252	1186944.482	0.003979948
30.25583333	1188491.321	0.003985135
30.25983333	1220704.065	0.004093147
30.26366667	1218767.214	0.004086653
30.2675	1243738.69	0.004170385
30.2715	1288467.61	0.004320366
30.27533333	1334527.68	0.00447481
30.27933333	1451311.181	0.004866397
30.28316667	1647677.222	0.005524833
30.28716667	1932204.013	0.00647888
30.291	2380100.083	0.007980722
30.295	3182328.678	0.010670678
30.29883333	4399864.676	0.014753203
30.30266667	6192114.822	0.020762803
30.30666667	8756092.492	0.029360086
30.3105	12218498.09	0.040969891
30.3145	16789311.44	0.056296303
30.31833333	22562337.83	0.075653859
30.32233333	29777412.44	0.099846752
30.32616667	38273466.12	0.128334902
30.33016667	47753820.21	0.160123512
30.334	57680668.87	0.193409265
30.338	67717451.49	0.227063638
30.34183333	77699701.77	0.260535159
30.34566667	86835268.99	0.291167663
30.34966667	94363470.97	0.316410505
30.3535	99597055.79	0.333959258
30.3575	102118784.5	0.342414876
30.36133333	101725428.1	0.341095911
30.36533333	98771580.84	0.331191355
30.36916667	93387923.12	0.313139392
30.373	86293901.62	0.289352402
30.377	78095373.33	0.261861887

Table 3. Samples Results STD

Retention time (minutes)	Indensity	Percentage of maximum peak area
30.38083333	69544766.81	0.233190816
30.38483333	61006229.78	0.204560216
30.38866667	52886129.14	0.177332676
30.39266667	45508741.48	0.15259553
30.3965	39123741.98	0.131185965
30.4005	33572006.29	0.112570419
30.40433333	28913305.21	0.096949311
30.40833333	24838515.51	0.083286119
30.41216667	21497775.57	0.072084271
30.416	18756745.21	0.062893312
30.42	16502642.58	0.055335072
30.42383333	14709866.9	0.049323709
30.42783333	13169689.57	0.044159335
30.43166667	11847328.76	0.039725322
30.43566667	10649503.71	0.03570889
30.4395	9616894.618	0.032246445
30.4435	8805953.49	0.029527275
30.44733333	8142348.369	0.027302139
30.45133333	7507553.185	0.025173604
30.45516667	6916787.389	0.023192706
30.459	6404373.259	0.021474528
30.463	5916265.222	0.019837851
30.46683333	5527828.009	0.018535381
30.47083333	5176956.392	0.017358872
30.47466667	4949621.881	0.016596596
30.47866667	4649475.925	0.015590175
30.4825	4378796.617	0.014682559
30.4865	4145088.147	0.01389891
30.49033333	3929356.767	0.013175541
30.49416667	3773458.983	0.012652799
30.49816667	3636537.865	0.012193689
30.502	3469051.219	0.011632089
30.506	3333040.605	0.011176031
30.50983333	3238607.895	0.010859388
30.51383333	3175596.557	0.010648105
30.51783333	3098615.372	0.010389979
30.52166667	2951459.974	0.009896551
30.52566667	2884823.679	0.009673113
30.5295	2785119.576	0.009338795
30.5335	2673740.722	0.00896533
30.53733333	2693650.494	0.009032089
30.54133333	2589251.553	0.008682029
30.54516667	2532594.183	0.008492051
30.549	2462259.708	0.008256212
30.553	2413259.774	0.00809191
30.55683333	2369695.636	0.007945835
30.56083333	2309897.343	0.007745325
30.56466667	2257145.581	0.007568443
30.56866667	2159528.055	0.007241122
30.5725	2128849.438	0.007138253
30.5765	2039070.856	0.006837216
30.58033333	2061060.201	0.006910949
30.58416667	1961290.986	0.006576412
30.58816667	1969524.172	0.006604019
30.592	1931939.837	0.006477995
30.596	2004245.465	0.006720443
30.59983333	2074964.946	0.006957573
30.60383333	2106838.627	0.007064448
30.60766667	2276060.143	0.007631866
30.61166667	2562289.647	0.008591623
30.6155	3027634.955	0.010151974
30.6195	3814248.079	0.012789569
30.62333333	4916226.923	0.016484619
30.62716667	6492759.404	0.021770896
30.63116667	8669044.15	0.029068204
30.635	11679521.14	0.039162646
30.639	15674017.43	0.052556606
30.64283333	20763626.65	0.069622594
30.64683333	26944285.57	0.090346984
30.65066667	34075498.85	0.114258682
30.6545	42419937.42	0.14223845
30.6585	51851726.33	0.173864216
30.66233333	62403194.32	0.209244383
30.66633333	73550349.27	0.24662195

Table 3. Samples Results STD

Retention time (minutes)	Indensity	Percentage of maximum peak area
30.67016667	84796287.78	0.284330748
30.67416667	95402010.1	0.319892835
30.678	104759729.9	0.35127024
30.682	112401782.4	0.376894834
30.68583333	117786335.6	0.3949498
30.68966667	120361924.8	0.403586018
30.69366667	119303120.5	0.400035737
30.6975	114599162.7	0.384262878
30.7015	106678695.2	0.35770473
30.70533333	96458274.47	0.323434599
30.70933333	84870991.17	0.284581236
30.71316667	73028575.28	0.244872387
30.71716667	61798192.99	0.207215751
30.721	51660963.72	0.173224569
30.725	43082737.01	0.144460886
30.72883333	35909639.23	0.120408745
30.73266667	29905351.1	0.100275744
30.73666667	25231030.19	0.084602261
30.7405	21622068.27	0.072501037
30.7445	18628217.56	0.062462346
30.74833333	16151140.12	0.054156448
30.75233333	14252340.07	0.047789574
30.75616667	12646859.81	0.042406233
30.76016667	11348701.53	0.038053373
30.764	10323429.14	0.034615529
30.768	9401964.948	0.031525764
30.77183333	8615112.065	0.028887364
30.77566667	7982607.602	0.026766511
30.77966667	7472542.033	0.025056208
30.7835	7009784.5	0.023504534
30.7875	6487793.536	0.021754245
30.79133333	6059311.174	0.020317499
30.79533333	5644307.25	0.018925948
30.79916667	5243547.762	0.017582159
30.80316667	4927074.359	0.016520991
30.807	4721094.366	0.015830319
30.811	4574832.168	0.015339887
30.81483333	4355692.381	0.014605088
30.81883333	4205765.394	0.014102368
30.82266667	4093031.329	0.013724359
30.8265	3987068.808	0.013369055
30.8305	3829396.347	0.012840363
30.83433333	3644594.679	0.012220704
30.83833333	3596328.912	0.012058864
30.84216667	3687646.157	0.01236506
30.84616667	3774875.698	0.01265755
30.85	4114298.055	0.013795668
30.854	4700321.5	0.015760665
30.85783333	5657834.757	0.018971307
30.86166667	7157316.126	0.023999223
30.86566667	9349857.21	0.031351041
30.8695	12251483.44	0.041080495
30.8735	16108484.93	0.054013421
30.87733333	21004193.99	0.070429241
30.88133333	27090921.48	0.09083867
30.88516667	34247781.37	0.114836363
30.88916667	42360993.72	0.142040805
30.893	51196811.46	0.171668219
30.897	60296991.66	0.202182067
30.90083333	69212629.33	0.232077125
30.90466667	77193445.53	0.258837629
30.90866667	84086832.89	0.281951872
30.9125	89394424.92	0.299748778
30.9165	93041162.36	0.311976667
30.92033333	94206297.5	0.315883486
30.92433333	92780434.39	0.311102419
30.92816667	89179376.13	0.299027698
30.932	83832053.3	0.28109757
30.936	77276822.03	0.259117199
30.93983333	69723861.01	0.233791338
30.94383333	62124109.64	0.208308583
30.94766667	54524253.41	0.182825477
30.95166667	47623623.52	0.159686949
30.9555	41247101.63	0.13830581

Table 3. Samples Results STD

Retention time (minutes)	Indensity	Percentage of maximum peak area
30.9595	35640527.32	0.119506385
30.96333333	30766076.16	0.103161844
30.96733333	26604711.19	0.089208356
30.97116667	23041242.1	0.077259675
30.975	20069859.45	0.06729632
30.979	17617284.84	0.059072583
30.98283333	15574240.76	0.052222045
30.98683333	13888501.47	0.046569586
30.99066667	12541490.11	0.042052917
30.99466667	11380441.41	0.0381598
30.9985	10403043.99	0.034882485
31.0025	9507781.529	0.031880578
31.00633333	8768146.613	0.029400505
31.01016667	8031249.059	0.026929611
31.01416667	7457344.303	0.025005249
31.018	6887374.047	0.02309408
31.022	6381952.977	0.02139935
31.02583333	6037984.701	0.020245989
31.02983333	5687354.598	0.01907029
31.03366667	5415580.784	0.018159004
31.03766667	5122123.917	0.017175013
31.0415	4908121.313	0.01645744
31.0455	4671720.454	0.015664763
31.04933333	4467088.593	0.014978611
31.05316667	4333497.778	0.014530668
31.05716667	4283593.027	0.014363332
31.061	4320929.797	0.014488526
31.065	4498227.394	0.015083023
31.06883333	4737771.422	0.015886239
31.07283333	5208975.373	0.017466235
31.07666667	5872377.972	0.019690692
31.08066667	6944268.742	0.023284853
31.0845	8399989.816	0.028166037
31.0885	10339382.28	0.034669021
31.09233333	12852279.93	0.043095028
31.09616667	16006631.66	0.053671896
31.10016667	19743031.64	0.066200433
31.104	24226986.16	0.081235597
31.108	29390442.32	0.098549201
31.11183333	35180057.8	0.117962383
31.11583333	41596943.41	0.139478866
31.11966667	48472904.27	0.162534675
31.1235	56280100.5	0.188713014
31.1275	64716124.38	0.216999877
31.13133333	73723522.44	0.247202617
31.13533333	82871388.58	0.277876361
31.13916667	92096971.9	0.308810699
31.14316667	101303492.4	0.339681117
31.147	110027061.6	0.368932149
31.151	118219951.1	0.396403757
31.15483333	125210146.9	0.419842609
31.15883333	130572237.7	0.437822256
31.16266667	133911161.9	0.449018015
31.1665	135249275.4	0.453504848
31.1705	134216191.8	0.450040811
31.17433333	131666227.5	0.441490517
31.17833333	128573865.7	0.431121507
31.18216667	126061320.4	0.422696682
31.18616667	124682849.9	0.418074528
31.19	124569120.6	0.417693181
31.194	125449864.7	0.420646408
31.19783333	126933093.7	0.425619828
31.20183333	128240585.4	0.430003984
31.20566667	128623538	0.431288064
31.2095	127466425.3	0.427408145
31.2135	123882542.5	0.415391014
31.21733333	118488807.3	0.39730526
31.22133333	111362443.4	0.373409823
31.22516667	103245755.8	0.346193728
31.22916667	94040467.58	0.315327441
31.233	84245814.66	0.282484954
31.237	74432762.73	0.249580773
31.24083333	65177030.65	0.218545344
31.24466667	56722800.85	0.190197434

Table 3. Samples Results STD

Retention time (minutes)	Indensity	Percentage of maximum peak area
31.24866667	49086155.09	0.164590969
31.2525	42602608.97	0.142850967
31.2565	37050454.02	0.124234015
31.26033333	32392144.9	0.108614221
31.26433333	28491006.91	0.095533301
31.26816667	25293426.26	0.084811482
31.272	22606809.97	0.075802979
31.276	20309647.09	0.068100353
31.27983333	18320188.83	0.061429493
31.28383333	16708466.35	0.056025221
31.28766667	15331642.69	0.051408588
31.29166667	14161421.61	0.047484715
31.2955	12989109.03	0.04355383
31.2995	11926248.61	0.039989949
31.30333333	10950441.93	0.036717968
31.30733333	10099077.56	0.033863255
31.31116667	9378083.084	0.031445685
31.315	8821385.581	0.029579021
31.319	8355156.03	0.028015705
31.32283333	7967814.414	0.026716908
31.32683333	7575151.393	0.025400268
31.33066667	7140394.542	0.023942483
31.33466667	6810279.507	0.022835574
31.3385	6460975.803	0.021664322
31.3425	6203196.742	0.020799962
31.34633333	5949460.168	0.019949157
31.35016667	5676643.248	0.019034374
31.35416667	5380553.401	0.018041554
31.358	5151964.622	0.017275072
31.362	4894309.966	0.016411129
31.36583333	4743347.381	0.015904936
31.36983333	4575467.377	0.015342017
31.37366667	4435177.578	0.01487161
31.37766667	4295001.183	0.014401584
31.3815	4193882.676	0.014062524
31.3855	4034173.789	0.013527003
31.38933333	3950041.753	0.0132449
31.39316667	3860636.684	0.012945115
31.39716667	3764620.153	0.012623162
31.401	3648522.091	0.012233873
31.405	3498417.283	0.011730556
31.40883333	3402831.647	0.011410047
31.41283333	3342562.534	0.011207959
31.41666667	3244405.804	0.010878829
31.42066667	3151766.582	0.0105682
31.4245	3027984.91	0.010153147
31.4285	2975757.195	0.009978023
31.43233333	2926302.349	0.009812195
31.43616667	2876238.55	0.009644326
31.44016667	2895507.807	0.009708938
31.444	2783414.616	0.009333078
31.448	2713460.424	0.009098514
31.45183333	2626026.746	0.00880534
31.45583333	2590130.544	0.008684976
31.45966667	2585641.127	0.008669923
31.4635	2535445.958	0.008501613
31.4675	2512342.474	0.008424145
31.47133333	2500279.5	0.008383696
31.47533333	2423944.701	0.008127738
31.47916667	2339709.141	0.007845287
31.48316667	2257519.12	0.007569696
31.487	2233094.478	0.007487797
31.491	2270570.288	0.007613458
31.49483333	2285642.952	0.007663998
31.49883333	2252334.514	0.007552311
31.50266667	2223818.394	0.007456694
31.5065	2191607.507	0.007348687
31.5105	2153837.418	0.00722204
31.51433333	2122812.752	0.007118011
31.51833333	2109164.356	0.007072247
31.52216667	2125035.964	0.007125466
31.52616667	2114028.304	0.007088556
31.53	2103678.158	0.007053851
31.534	2107543.783	0.007066813

Table 3. Samples Results STD

Retention time (minutes)	Indensity	Percentage of maximum peak area
31.53783333	2097553.172	0.007033313
31.54183333	2088422.218	0.007002696
31.54566667	2050445.394	0.006875356
31.5495	2026712.042	0.006795776
31.5535	1936106.459	0.006491966
31.55733333	1934027.662	0.006484995
31.56133333	1939621.774	0.006503753
31.56516667	1941424.862	0.006509799
31.56916667	1952211.323	0.006545967
31.573	1941830.758	0.00651116
31.577	1916169.089	0.006425114
31.58083333	1894320.194	0.006351852
31.58466667	1847960.506	0.006196403
31.58866667	1834308.517	0.006150627
31.5925	1815182.706	0.006086496
31.5965	1816139.611	0.006089704
31.60033333	1794620.266	0.006017548
31.60433333	1767558.357	0.005926806
31.60816667	1767845.48	0.005927769
31.61216667	1752736.307	0.005877107
31.616	1748966.667	0.005864467
31.62	1741734.507	0.005840216
31.62383333	1704111.872	0.005714064
31.62766667	1698243.434	0.005694386
31.63166667	1741582.526	0.005839707
31.6355	1674860.313	0.00561598
31.6395	1702005.162	0.005707
31.64333333	1690876.973	0.005669686
31.64733333	1686186.842	0.005653959
31.65116667	1711874.675	0.005740093
31.65516667	1732042.025	0.005807717
31.659	1750068.311	0.005868161
31.66283333	1761093.425	0.005905129
31.66683333	1771788.062	0.005940989
31.67066667	1776550.465	0.005956958
31.67466667	1801589.254	0.006040916
31.6785	1734862.175	0.005817173
31.6825	1770853.192	0.005937854
31.68633333	1756113.412	0.00588843
31.69033333	1717654.672	0.005759474
31.69416667	1724220.823	0.005781491
31.698	1683610.09	0.005645319
31.702	1666151.191	0.005586778
31.70583333	1654898.181	0.005549045
31.70983333	1641639.555	0.005504588
31.71366667	1631151.302	0.005469419
31.71766667	1659940.149	0.005565951
31.7215	1674367.949	0.005614329
31.7255	1695135.413	0.005683965
31.72933333	1656468.253	0.00555431
31.73333333	1627072.496	0.005455743
31.73716667	1635531.47	0.005484107
31.741	1629392.843	0.005463523
31.745	1650449.658	0.005534129
31.74883333	1677339.588	0.005624294
31.75283333	1661778.908	0.005572117
31.75666667	1658039.309	0.005559578
31.76066667	1662105.551	0.005573212
31.7645	1640784.74	0.005501721
31.7685	1586430.131	0.005319465
31.77233333	1617766.487	0.005424539
31.77633333	1657566.626	0.005557993
31.78016667	1635010.333	0.005482359
31.784	1602792.408	0.005374329
31.788	1559369.872	0.005228729
31.79183333	1531926.643	0.005136709
31.79583333	1522765.819	0.005105992
31.79966667	1517557.014	0.005088526
31.80366667	1513130.63	0.005073684
31.8075	1488766.349	0.004991988
31.8115	1540734.816	0.005166244
31.81533333	1469691.491	0.004928028
31.81916667	1444343.357	0.004843033
31.82316667	1427103.233	0.004785225

Table 3. Samples Results STD

Retention time (minutes)	Indensity	Percentage of maximum peak area
31.827	1483090.774	0.004972957
31.831	1496232.87	0.005017024
31.83483333	1500515.696	0.005031385
31.83883333	1481926.429	0.004969053
31.84266667	1430837.093	0.004797745
31.84666667	1353628.271	0.004538856
31.8505	1312139.996	0.004399741
31.8545	1275739.91	0.004277688
31.85833333	1270862.434	0.004261334
31.86216667	1309982.095	0.004392506
31.86616667	1348553.944	0.004521841
31.87	1331700.122	0.004465329
31.874	1350567.245	0.004528592
31.87783333	1378962.612	0.004623805
31.88183333	1373205.46	0.0046045
31.88566667	1405106.459	0.004711468
31.88966667	1375191.086	0.004611158
31.8935	1345473.994	0.004511514
31.8975	1331907.902	0.004466025
31.90133333	1335749.524	0.004478907
31.90516667	1339552.013	0.004491657
31.90916667	1318442.863	0.004420876
31.913	1325681.339	0.004445147
31.917	1319738.082	0.004425219
31.92083333	1301876.417	0.004365327
31.92483333	1323248.913	0.004436991
31.92866667	1326274.421	0.004447136
31.93266667	1338530.438	0.004488231
31.9365	1316353.345	0.004413869
31.9405	1320148.702	0.004426596
31.94433333	1288902.48	0.004321824
31.94816667	1314785.722	0.004408613
31.95216667	1328715.77	0.004455322
31.956	1337091.105	0.004483405
31.96	1358393.051	0.004554833
31.96383333	1334464.944	0.004474599
31.96783333	1300467.771	0.004360603
31.97166667	1325826.448	0.004445634
31.97566667	1304827.427	0.004375222
31.9795	1302016.109	0.004365795
31.9835	1276167.028	0.00427912
31.98733333	1253220.801	0.004202179
31.99116667	1213635.295	0.004069445
31.99516667	1238725.508	0.004153575
31.999	1263477.112	0.00423657
32.003	1284408.877	0.004306756
32.00683333	1276839.038	0.004281374
32.01083333	1269781.034	0.004257708
32.01466667	1271442.489	0.004263279
32.0185	1278801.907	0.004287955
32.0225	1281837.902	0.004298135
32.02633333	1254030.029	0.004204893
32.03033333	1227030.989	0.004114362
32.03416667	1199240.765	0.004021179
32.03816667	1211703.373	0.004062967
32.042	1209851.151	0.004056756
32.046	1231639.154	0.004129814
32.04983333	1227712.271	0.004116647
32.05383333	1229711.606	0.004123351
32.05766667	1251226.661	0.004195493
32.0615	1245833.842	0.00417741
32.0655	1231021.313	0.004127742
32.06933333	1241397.435	0.004162534
32.07333333	1261824.519	0.004231028
32.07716667	1332190.193	0.004466972
32.08116667	1373968.334	0.004607058
32.085	1460024.642	0.004895614
32.089	1580025.003	0.005297988
32.09283333	1767822.467	0.005927692
32.09683333	2089367.261	0.007005865
32.10066667	2616650.355	0.0087739
32.1045	3316253.88	0.011119743
32.1085	4337972.61	0.014545672
32.11233333	5814107.781	0.019495306

Table 3. Samples Results STD

Retention time (minutes)	Indensity	Percentage of maximum peak area
32.11633333	7807645.923	0.026179847
32.12016667	10497980.9	0.035200819
32.12416667	13870027.92	0.046507642
32.128	18070116.82	0.060590976
32.132	23179345.53	0.07772275
32.13583333	29083151.51	0.097518823
32.13966667	35795381.21	0.120025626
32.14366667	43297582.6	0.145181285
32.1475	51191225.76	0.171649489
32.1515	59396787.08	0.199163588
32.1555	67290594.71	0.225632343
32.15933333	75101659.75	0.251823654
32.16333333	82159618.74	0.275489723
32.16716667	87456349.1	0.293250209
32.17116667	90619848.96	0.303857752
32.175	92173604.33	0.309067655
32.179	91548850.8	0.306972792
32.18283333	88712475.78	0.297462132
32.18683333	83740853.87	0.280791768
32.19066667	76709994.22	0.257216567
32.1945	69061164.86	0.231569249
32.1985	61240375.05	0.205345329
32.20233333	53636449.34	0.179848578
32.20633333	46763193.06	0.156801836
32.21016667	40367787.65	0.135357379
32.21416667	34675781.2	0.116271491
32.218	29812456.24	0.099964258
32.222	25638771.54	0.08596946
32.22583333	22255192.67	0.074623969
32.22983333	19311141.67	0.064752261
32.23366667	17032494.57	0.057111721
32.2375	15093938.67	0.050611542
32.2415	13417560.49	0.044990472
32.24533333	12044519.43	0.040386523
32.24933333	10889772.24	0.036514536
32.25316667	9806560.074	0.032882413
32.25716667	8942000.536	0.029983455
32.261	8248558.151	0.027658271
32.265	7644572.96	0.025633046
32.26883333	7071450.665	0.023711308
32.27283333	6597138.067	0.022120888
32.27666667	6207242.319	0.020813527
32.2805	5818239.425	0.01950916
32.2845	5399339.539	0.018104545
32.28833333	5168686.655	0.017331142
32.29233333	4787607.602	0.016053345
32.29616667	4553558.796	0.015268555
32.30016667	4306246.91	0.014439293
32.304	4124336.724	0.013829329
32.308	3937000.183	0.01320117
32.31183333	3823210.826	0.012819622
32.31566667	3666333.639	0.012293597
32.31966667	3523899.117	0.011815999
32.3235	3350277.918	0.011233829
32.3275	3204435.628	0.010744805
32.33133333	3099653.034	0.010393458
32.33533333	2991918.173	0.010032212
32.33916667	2896365.465	0.009711814
32.34316667	2859137.628	0.009586985
32.347	2848117.732	0.009550034
32.351	2734312.671	0.009168434
32.35483333	2644390.072	0.008866914
32.35866667	2547194.357	0.008541007
32.36266667	2553093.764	0.008560788
32.3665	2509440.69	0.008414415
32.3705	2458964.735	0.008245164
32.37433333	2400517.332	0.008049184
32.37833333	2379557.434	0.007978903
32.38216667	2287546.187	0.00767038
32.386	2213128.088	0.007420848
32.39	2199050.493	0.007373644
32.39383333	2180531.203	0.007311547
32.39783333	2146480.014	0.00719737
32.40166667	2134022.851	0.0071556

Table 3. Samples Results STD

Retention time (minutes)	Indensity	Percentage of maximum peak area
32.40566667	2062110.172	0.006914469
32.4095	2041290.096	0.006844657
32.4135	2032274.262	0.006814426
32.41733333	2001073.762	0.006709808
32.42133333	1970173.746	0.006606197
32.42516667	1953293.053	0.006549594
32.429	1904853.285	0.006387171
32.433	1866803.206	0.006259585
32.43683333	1826252.197	0.006123613
32.44083333	1809449.104	0.006067271
32.44466667	1846277.797	0.006190761
32.44866667	1844349.675	0.006184296
32.4525	1867924.426	0.006263344
32.4565	1826103.387	0.006123114
32.46033333	1797446.843	0.006027026
32.46433333	1780852.615	0.005971384
32.46816667	1769177.877	0.005932237
32.472	1720869.502	0.005770254
32.476	1679910.585	0.005632914
32.47983333	1666055.319	0.005586456
32.48383333	1603205.921	0.005375716
32.48766667	1587705.412	0.005323741
32.49166667	1563705.035	0.005243265
32.4955	1550847.614	0.005200153
32.4995	1549368.687	0.005195194
32.50333333	1555569.177	0.005215985
32.50716667	1537915.501	0.00515679
32.51116667	1546242.353	0.005184711
32.515	1530039.186	0.00513038
32.519	1486793.071	0.004985371
32.52283333	1497779.148	0.005022209
32.52683333	1464530.629	0.004910723
32.53066667	1498929.067	0.005026065
32.53466667	1479595.58	0.004961237
32.5385	1441941.079	0.004834978
32.5425	1456448.562	0.004883623
32.54633333	1398117.879	0.004688034
32.55033333	1393353.406	0.004672058
32.55416667	1390778.276	0.004663424
32.558	1364743.19	0.004576125
32.562	1359549.041	0.004558709
32.56583333	1352001.616	0.004533402
32.56983333	1374753.184	0.00460969
32.57366667	1362380.523	0.004568203
32.57766667	1345133.615	0.004510373
32.5815	1376312.284	0.004614918
32.5855	1376390.512	0.00461518
32.58933333	1415665.098	0.004746872
32.59316667	1478364.11	0.004957108
32.59716667	1621070.624	0.005435618
32.601	1812579.985	0.006077769
32.605	2030570.938	0.006808715
32.60883333	2334713.679	0.007828537
32.61283333	2788970.357	0.009351707
32.61666667	3442273.496	0.0115423
32.62066667	4183651.217	0.014028216
32.6245	5171622.38	0.017340986
32.6285	6449158.798	0.021624698
32.63233333	8090254.513	0.027127462
32.63616667	9921351.712	0.033267321
32.64016667	12075379.81	0.040490001
32.644	14613494.35	0.049000562
32.648	17389418.92	0.058308525
32.65183333	20489569.56	0.068703652
32.65583333	23937507.47	0.080264945
32.65966667	27725623.26	0.09296689
32.66366667	31894947.74	0.106947068
32.6675	36555465.05	0.122574266
32.6715	41613645.7	0.13953487
32.67533333	46807039.37	0.156948858
32.67933333	52139079.57	0.174827742
32.68316667	57546554.88	0.192959567
32.687	63261801.39	0.212123381
32.691	68970770.06	0.231266145

Table 3. Samples Results STD

Retention time (minutes)	Indensity	Percentage of maximum peak area
32.69483333	74673810.71	0.250389032
32.69883333	79925954.2	0.268000014
32.70266667	84547552.01	0.283496711
32.70666667	88905371.4	0.298108931
32.7105	92290207.91	0.309458639
32.7145	94108626.35	0.315555985
32.71833333	93855111.58	0.314705924
32.72216667	91493798.15	0.306788195
32.72616667	87053295.35	0.291898728
32.73	80402340.5	0.269597387
32.734	72137595.98	0.241884841
32.73783333	63447552.98	0.212746226
32.74183333	54637999.23	0.183206878
32.74566667	46500900.82	0.155922343
32.7495	39191254.14	0.13141234
32.7535	32928066.51	0.110411222
32.75733333	27857586.19	0.093409376
32.76133333	23804894.74	0.079820281
32.76516667	20589447.74	0.069038554
32.76916667	18024923.44	0.060439438
32.773	15938057.71	0.053441961
32.777	14208148.64	0.047641396
32.78083333	12780331.81	0.042853778
32.78483333	11593062.64	0.038872741
32.78866667	10545426.88	0.03535991
32.7925	9643472.576	0.032335564
32.7965	8962774.155	0.030053111
32.80033333	8342595.501	0.027973588
32.80433333	7786948.211	0.026110445
32.80816667	7261020.744	0.024346956
32.81216667	6845184.251	0.022952613
32.816	6465851.178	0.02168067
32.82	6132873.106	0.02056416
32.82383333	5758890.749	0.019310158
32.82783333	5404969.84	0.018123424
32.83166667	5174686.166	0.017351259
32.8355	4955313.62	0.016615681
32.8395	4770527.67	0.015996074
32.84333333	4586484.784	0.015378959
32.84733333	4391897.824	0.014726489
32.85116667	4180375.517	0.014017233
32.85516667	3996377.153	0.013400267
32.859	3895240.464	0.013061145
32.863	3729973.525	0.012506988
32.86683333	3622691.818	0.012147261
32.87083333	3484037.933	0.011682341
32.87466667	3432615.522	0.011509916
32.8785	3370481.199	0.011301573
32.8825	3251012.442	0.010900982
32.88633333	3206255.455	0.010750907
32.89033333	3124700.702	0.010477446
32.89416667	3096666.653	0.010383444
32.89816667	3051479.758	0.010231928
32.902	2964555.382	0.009940462
32.906	2863155.286	0.009600457
32.90983333	2757189.116	0.009245141
32.91366667	2700716.916	0.009055784
32.91766667	2670725.946	0.008955221
32.9215	2589542.921	0.008683006
32.9255	2533133.71	0.00849386
32.92933333	2454494.899	0.008230176
32.93333333	2395307.799	0.008031715
32.93716667	2330454.279	0.007814255
32.94116667	2241451.741	0.00751582
32.945	2218324.894	0.007438273
32.949	2206638.461	0.007399088
32.95283333	2179818.835	0.007309159
32.95666667	2117784.386	0.007101151
32.96066667	2108282.128	0.007069289
32.9645	2021134.591	0.006777074
32.9685	1972614.647	0.006614382
32.97233333	2006835.05	0.006729126
32.97633333	2000422.635	0.006707625
32.98016667	1960651.794	0.006574269

Table 3. Samples Results STD

Retention time (minutes)	Indensity	Percentage of maximum peak area
32.984	1902631.021	0.006379719
32.988	1860010.767	0.006236809
32.99183333	1830602.974	0.006138202
32.99583333	1793251.061	0.006012957
32.99966667	1819701.81	0.006101649
33.00366667	1752674.403	0.005876899
33.0075	1733733.37	0.005813388
33.0115	1703006.877	0.005710359
33.01533333	1724807.141	0.005783457
33.01933333	1768526.407	0.005930052
33.02316667	1753237.834	0.005878788
33.027	1787869.752	0.005994913
33.031	1824252.838	0.006116909
33.03483333	1897130.238	0.006361274
33.03883333	1983560.549	0.006651084
33.04266667	2204950.936	0.007393429
33.04666667	2479084.821	0.008312629
33.0505	3053686.261	0.010239327
33.0545	3840088.839	0.012876216
33.05833333	4894915.188	0.016413158
33.06233333	6407877.011	0.021486276
33.06616667	8578574.479	0.02876485
33.07	11184318.44	0.03750218
33.074	14677921.69	0.049216594
33.07783333	18872280.55	0.063280714
33.08183333	23981746.99	0.080413285
33.08566667	29856517.21	0.100111999
33.08966667	36475516.16	0.122306189
33.0935	43617856.15	0.146255196
33.0975	51264716.63	0.171895912
33.10133333	58945013.17	0.197648743
33.10516667	66385849.5	0.222598639
33.10916667	73340307.43	0.245917657
33.113	79429235.11	0.266334463
33.117	84379559.5	0.282933415
33.12083333	87712880.98	0.294110387
33.12483333	89193010.64	0.299073416
33.12866667	88916121	0.298144976
33.13266667	87281333.91	0.292663365
33.1365	84306852.09	0.282689619
33.1405	80213651.76	0.268964694
33.14433333	75004403.28	0.251497543
33.14816667	68941675.02	0.231168587
33.15216667	62622223.54	0.209978811
33.156	56418204.92	0.189176093
33.16	50446182.33	0.169151281
33.16383333	45001809.77	0.150895736
33.16783333	40043793.19	0.134270992
33.17166667	35533881.61	0.119148791
33.1755	31516790.08	0.105679066
33.1795	27991241.53	0.093857536
33.1835	24852075.31	0.083331586
33.18733333	22110840.63	0.074139942
33.19116667	19701431.76	0.066060944
33.19516667	17568007.98	0.058907353
33.199	15801639.57	0.052984536
33.203	14234877.97	0.047731022
33.20683333	12810396.25	0.042954587
33.21083333	11619658.94	0.038961922
33.21466667	10632199.56	0.035650868
33.2185	9787590.341	0.032818806
33.2225	9098266.439	0.030507431
33.22633333	8453047.89	0.028343946
33.23033333	7889526.898	0.026454402
33.23416667	7419278.341	0.02487761
33.23816667	7007624.262	0.023497291
33.242	6583702.551	0.022075837
33.246	6205375.176	0.020807267
33.24983333	5851185.086	0.01961963
33.25366667	5520087.139	0.018509425
33.25766667	5223208.264	0.017513959
33.2615	4936816.198	0.016553657
33.2655	4690997.091	0.0157294
33.26933333	4506082.8	0.015109363

Table 3. Samples Results STD

Retention time (minutes)	Indensity	Percentage of maximum peak area
33.27333333	4354458.687	0.014600952
33.27716667	4217520.128	0.014141782
33.28116667	4093617.128	0.013726323
33.285	3974579.866	0.013327179
33.289	3836895.162	0.012865507
33.29283333	3728941.179	0.012503527
33.29683333	3567430.431	0.011961964
33.30066667	3416812.931	0.011456928
33.3045	3330751.033	0.011168354
33.3085	3257252.304	0.010921905
33.31233333	3157406.418	0.010587111
33.31633333	3088666.31	0.010356618
33.32016667	3029762.97	0.010159109
33.32416667	2933970.263	0.009837907
33.328	2871770.563	0.009629345
33.332	2833115.04	0.009499729
33.33583333	2813905.348	0.009435316
33.33983333	2780078.515	0.009321892
33.34366667	2685561.245	0.009004965
33.3475	2660478.929	0.008920862
33.3515	2615783.656	0.008770994
33.35533333	2535556.654	0.008501984
33.35933333	2561148.635	0.008587797
33.36316667	2399770.358	0.008046679
33.36716667	2364227.84	0.007927501
33.371	2319326.816	0.007776943
33.375	2322783.671	0.007788535
33.37883333	2294438.076	0.007693489
33.38266667	2275624.515	0.007630405
33.38666667	2272887.989	0.007621229
33.3905	2251664.788	0.007550066
33.3945	2166076.861	0.00726308
33.39833333	2142422.33	0.007183764
33.40233333	2085070.654	0.006991458
33.40616667	2112477.501	0.007083356
33.41	2136197.377	0.007162891
33.414	2092673.806	0.007016952
33.41783333	2030601.266	0.006808817
33.42183333	2017863.176	0.006766105
33.42566667	2018819.386	0.006769311
33.42966667	2008583.063	0.006734987
33.4335	2004452.318	0.006721137
33.4375	1995322.376	0.006690523
33.44133333	1981230.99	0.006643273
33.44533333	1979523.682	0.006637548
33.44916667	1957175.776	0.006562613
33.453	1926856.388	0.006460949
33.457	1883511.064	0.006315608
33.46083333	1872729.612	0.006279457
33.46483333	1880830.266	0.006306619
33.46866667	1855636.858	0.006222143
33.47266667	1873930.231	0.006283482
33.4765	1930268.798	0.006472391
33.4805	1894351.232	0.006351956
33.48433333	1858989.997	0.006233386
33.48833333	1846812.294	0.006192553
33.49216667	1842710.068	0.006178798
33.496	1868309.307	0.006264635
33.5	1855303.455	0.006221025
33.50383333	1836304.31	0.006157319
33.50783333	1809239.205	0.006066567
33.51166667	1830184.989	0.0061368
33.51566667	1799385.308	0.006033526
33.51966667	1782180.062	0.005975835
33.5235	1722976.375	0.005777318
33.5275	1737465.08	0.005825901
33.53133333	1725649.957	0.005786283
33.53533333	1683247.464	0.005644103
33.53916667	1708498.176	0.005728772
33.543	1660322.952	0.005567235
33.547	1700388.712	0.00570158
33.55083333	1695049.366	0.005683676
33.55483333	1677260.06	0.005624027
33.55866667	1611716.74	0.005404253

Table 3. Samples Results STD

Retention time (minutes)	Indensity	Percentage of maximum peak area
33.56266667	1604772.592	0.005380969
33.5665	1566025.906	0.005251047
33.5705	1542373.511	0.005171738
33.57433333	1555056.34	0.005214265
33.57833333	1516413.782	0.005084693
33.58216667	1532605.179	0.005138984
33.586	1542095.632	0.005170807
33.59	1513210.466	0.005073952
33.59383333	1511403.243	0.005067892
33.59783333	1495999.561	0.005016242
33.60166667	1497487.903	0.005021232
33.60566667	1541774.684	0.00516973
33.6095	1541748.044	0.005169641
33.6135	1501700.356	0.005035357
33.61733333	1477596.109	0.004954533
33.62133333	1477591.354	0.004954517
33.62516667	1493108.641	0.005006548
33.629	1489558.867	0.004994645
33.633	1468649.327	0.004924534
33.63683333	1457739.831	0.004887953
33.64083333	1479570.74	0.004961154
33.64466667	1457975.052	0.004888742
33.64866667	1448167.956	0.004855857
33.6525	1438887.593	0.004824739
33.6565	1412791.391	0.004737236
33.66033333	1412012.793	0.004734625
33.66433333	1424873.821	0.00477775
33.66816667	1390909.7	0.004663864
33.672	1393615.69	0.004672938
33.676	1388887.347	0.004657083
33.67983333	1350390.584	0.004528
33.68383333	1333051.392	0.00446986
33.68766667	1326353.424	0.004447401
33.69166667	1374023.098	0.004607242
33.6955	1422798.73	0.004770792
33.6995	1431167.061	0.004798852
33.70333333	1470450.861	0.004930574
33.70733333	1505362.012	0.005047635
33.71116667	1444519.339	0.004843623
33.715	1416031.366	0.0047481
33.719	1401737.077	0.00470017
33.72283333	1437826.035	0.00482118
33.72683333	1538791.751	0.005159728
33.73066667	1636509.584	0.005487386
33.73466667	1738567.06	0.005829596
33.7385	1890161.094	0.006337906
33.7425	2111098.928	0.007078734
33.74633333	2412693.748	0.008090012
33.75033333	2924619.75	0.009806553
33.75416667	3612428.207	0.012112846
33.758	4561443.143	0.015294992
33.762	5734507.892	0.0192284
33.76583333	7129082.891	0.023904554
33.76983333	8892406.284	0.029817161
33.77366667	11082881	0.037162049
33.77766667	13785259.85	0.046223406
33.7815	16826904.61	0.056422356
33.7855	20416527.5	0.068458734
33.78933333	24326163.31	0.081568149
33.79316667	28689145.02	0.096197679
33.79716667	33437973.47	0.112120993
33.801	38541214.7	0.12923269
33.805	44095628.11	0.147857215
33.80883333	50047096.27	0.167813105
33.81283333	56300789.5	0.188782387
33.81666667	62596709.69	0.20989326
33.82066667	68934311.45	0.231143896
33.8245	75179144.34	0.252083468
33.8285	80977481.67	0.271525895
33.83233333	86040548.12	0.288502882
33.83616667	90198604.16	0.302445275
33.84016667	92599779.72	0.310496666
33.844	93221663.16	0.312581905
33.848	91725060.23	0.30756364

Table 3. Samples Results STD

Retention time (minutes)	Indensity	Percentage of maximum peak area
33.85183333	88237689.78	0.295870126
33.85583333	83020557.06	0.278376538
33.85966667	76458432.69	0.256373055
33.8635	68916425.37	0.231083922
33.8675	60945193.9	0.204355556
33.87133333	53237674.21	0.178511443
33.87533333	46116639.09	0.154633874
33.87916667	39552695.63	0.132624291
33.88316667	34074815.52	0.114256391
33.887	29383180.77	0.098524853
33.891	25424816.95	0.085252048
33.89483333	22206257.6	0.074459885
33.89866667	19565463.12	0.065605027
33.90266667	17375837.12	0.058262984
33.9065	15565455.55	0.052192587
33.9105	13944182.56	0.04675629
33.91433333	12542538.09	0.042056431
33.91833333	11449593.26	0.038391674
33.92216667	10449932.76	0.035039708
33.92616667	9679404.491	0.032456047
33.93	9114896.203	0.030563192
33.934	8599235.49	0.028834128
33.93783333	8030182.607	0.026926035
33.94166667	7473468.126	0.025059314
33.94566667	6996491.756	0.023459963
33.9495	6569039.62	0.022026671
33.9535	6242857.001	0.020932947
33.95733333	5906629.039	0.01980554
33.96133333	5592771.635	0.018753143
33.96516667	5292012.153	0.017744666
33.96916667	5052400.29	0.016941222
33.973	4818421.166	0.016156666
33.977	4598511.337	0.015419285
33.98083333	4402218.329	0.014761095
33.98466667	4259676.216	0.014283136
33.98866667	4143226.533	0.013892668
33.9925	4029670.423	0.013511903
33.9965	3868159.679	0.012970341
34.00033333	3747239.98	0.012564884
34.00433333	3597409.3	0.012062486
34.00816667	3493503.246	0.011714079
34.01216667	3407205.364	0.011424713
34.016	3258584.141	0.010926371
34.02	3178031.205	0.010656268
34.02383333	3133354.303	0.010506462
34.02766667	3048220.23	0.010220999
34.03166667	2957363.291	0.009916346
34.0355	2864999.061	0.009606639
34.0395	2782241.629	0.009329145
34.04333333	2739761.647	0.009186705
34.04733333	2683804.113	0.008999074
34.05116667	2673693.974	0.008965173
34.055	2692549.019	0.009028396
34.059	2644357.625	0.008866805
34.06283333	2620804.017	0.008787828
34.06683333	2516369.417	0.008437648
34.07066667	2460220.45	0.008249374
34.07466667	2373813.585	0.007959643
34.0785	2338393.993	0.007840878
34.0825	2298005.852	0.007705452
34.08633333	2301341.676	0.007716637
34.09033333	2262394.648	0.007586044
34.09416667	2245083.69	0.007527998
34.098	2242993.887	0.007520991
34.102	2255203.003	0.00756193
34.10583333	2211963.004	0.007416941
34.10983333	2116832.695	0.00709796
34.11366667	2049618.319	0.006872583
34.11766667	2019791.886	0.006772572
34.1215	2026199.043	0.006794056
34.1255	2050338.877	0.006874999
34.12933333	2003305.481	0.006717291
34.13333333	1964119.753	0.006585897
34.13716667	1904464.69	0.006385868

Table 3. Samples Results STD

Retention time (minutes)	Indensity	Percentage of maximum peak area
34.141	1904077.079	0.006384568
34.145	1881204.053	0.006307872
34.14883333	1888852.524	0.006333518
34.15283333	1887853.828	0.00633017
34.15666667	1913567.506	0.00641639
34.16066667	1907844.826	0.006397202
34.1645	1878002.028	0.006297136
34.1685	1840940.853	0.006172866
34.17233333	1789157.204	0.00599923
34.17633333	1765875.801	0.005921165
34.18016667	1731200.566	0.005804895
34.184	1716511.069	0.00575564
34.188	1715038.806	0.005750703
34.19183333	1767132.486	0.005925379
34.19583333	1759670.851	0.005900359
34.19966667	1755249.02	0.005885532
34.20366667	1708051.352	0.005727273
34.2075	1716303.784	0.005754945
34.2115	1716725.438	0.005756358
34.21533333	1670346.094	0.005600844
34.21933333	1639384.875	0.005497028
34.22316667	1634592.464	0.005480958
34.227	1611982.337	0.005405144
34.231	1574282.653	0.005278733
34.23483333	1581764.574	0.005303821
34.23883333	1598727.78	0.0053607
34.24266667	1604178.792	0.005378978
34.24666667	1585328.363	0.00531577
34.2505	1546004.875	0.005183915
34.2545	1554030.005	0.005210824
34.25833333	1529983.539	0.005130193
34.26233333	1514673.247	0.005078856
34.26616667	1484147.035	0.004976499
34.27	1436073.434	0.004815303
34.274	1411844.188	0.00473406
34.27783333	1425494.125	0.00477983
34.28183333	1457828.765	0.004888251
34.28566667	1449430.629	0.004860091
34.28966667	1446276.265	0.004849514
34.2935	1415818.472	0.004747386
34.2975	1430848.73	0.004797784
34.30133333	1406966.853	0.004717706
34.30516667	1406379.734	0.004715737
34.30916667	1426421.32	0.004782939
34.313	1405573.738	0.004713035
34.317	1398536.224	0.004689437
34.32083333	1365826.503	0.004579758
34.32483333	1347216.222	0.004517356
34.32866667	1367147.597	0.004584188
34.33266667	1400433.439	0.004695799
34.3365	1390964.579	0.004664049
34.3405	1414629.986	0.004743401
34.34433333	1415520.26	0.004746386
34.34816667	1400238.442	0.004695145
34.35216667	1391787.963	0.004666809
34.356	1381058.251	0.004630832
34.36	1365189.671	0.004577623
34.36383333	1378791.139	0.00462323
34.36783333	1418110.671	0.004755072
34.37166667	1469509.421	0.004927418
34.3755	1499433.957	0.005027758
34.3795	1454898.742	0.004878426
34.38333333	1406421.608	0.004715878
34.38733333	1361368.189	0.004564809
34.39116667	1376027.506	0.004613963
34.39516667	1390292.902	0.004661796
34.399	1388146.403	0.004654599
34.403	1394008.633	0.004674256
34.40683333	1386774.672	0.004649999
34.41083333	1352948.179	0.004536576
34.41466667	1343041.066	0.004503356
34.4185	1326892.414	0.004449208
34.4225	1311188.642	0.004396552
34.42633333	1329663.976	0.004458501

Table 3. Samples Results STD

Retention time (minutes)	Indensity	Percentage of maximum peak area
34.43033333	1318785.209	0.004422024
34.43416667	1326028.976	0.004446313
34.43816667	1270443.075	0.004259927
34.442	1216707.627	0.004079747
34.446	1236413.571	0.004145823
34.44983333	1235066.933	0.004141308
34.45383333	1243608.294	0.004169948
34.45766667	1282750.445	0.004301195
34.4615	1280855.474	0.004294841
34.4655	1279206.72	0.004289313
34.46933333	1283491.5	0.00430368
34.47333333	1279371.482	0.004289865
34.47716667	1266219.975	0.004245767
34.48116667	1260181.413	0.004225519
34.485	1288142.466	0.004319275
34.489	1299058.799	0.004355879
34.49283333	1281776.51	0.00429793
34.49666667	1285838.013	0.004311548
34.50066667	1269019.943	0.004255155
34.5045	1256024.808	0.004211581
34.5085	1272558.179	0.00426702
34.51233333	1238907.527	0.004154185
34.51633333	1236779.769	0.004147051
34.52016667	1237081.453	0.004148062
34.52416667	1233148.932	0.004134876
34.528	1207667.235	0.004049433
34.532	1184045.934	0.003970229
34.53583333	1191715.373	0.003995945
34.53966667	1193755.792	0.004002787
34.54366667	1209672.861	0.004056159
34.5475	1178504.047	0.003951646
34.5515	1188058.053	0.003983682
34.55533333	1166838.283	0.00391253
34.55933333	1158965.065	0.00388613
34.56316667	1148552.159	0.003851215
34.56716667	1138115.607	0.00381622
34.571	1121258.7	0.003759697
34.575	1108047.398	0.003715398
34.57883333	1100932.996	0.003691543
34.58266667	1102783.266	0.003697747
34.58666667	1126793.316	0.003778255
34.5905	1151478.58	0.003861027
34.5945	1137508.875	0.003814185
34.59833333	1114632.321	0.003737478
34.60233333	1085123.014	0.00363853
34.60616667	1060249.748	0.003555127
34.61016667	1082590.894	0.00363004
34.614	1092316.469	0.00366265
34.618	1103280.966	0.003699416
34.62183333	1126198.262	0.00377626
34.62566667	1164497.591	0.003904681
34.62966667	1166004.767	0.003909735
34.6335	1129330.741	0.003786763
34.6375	1083133.763	0.00363186
34.64133333	1102069.493	0.003695353
34.64533333	1096423.269	0.003676421
34.64916667	1097474.266	0.003679945
34.65316667	1098267.886	0.003682606
34.657	1088923.181	0.003651272
34.661	1123540.888	0.003767349
34.66483333	1130808.61	0.003791719
34.66866667	1108547.839	0.003717076
34.67266667	1092076.984	0.003661847
34.6765	1098735.399	0.003684174
34.6805	1104728.474	0.003704269
34.68433333	1111740.75	0.003727782
34.68833333	1096308.8	0.003676037
34.69216667	1076960.746	0.003611161
34.696	1026298.098	0.003441284
34.7	1041978.284	0.003493861
34.70383333	1061181.104	0.00355825
34.70783333	1073665.186	0.003600111
34.71166667	1094105.051	0.003668648
34.71566667	1069615.051	0.00358653

Table 3. Samples Results STD

Retention time (minutes)	Indensity	Percentage of maximum peak area
34.7195	1058594.581	0.003549577
34.7235	1047767.53	0.003513273
34.72733333	1034734.486	0.003469572
34.73133333	1030263.139	0.003454579
34.73516667	1031436.66	0.003458514
34.739	1059956.856	0.003554145
34.743	1113002.179	0.003732012
34.74683333	1143412.218	0.00383398
34.75083333	1162692.469	0.003898628
34.75466667	1147583.518	0.003847967
34.75866667	1131594.07	0.003794352
34.7625	1116614.542	0.003744124
34.7665	1129206.389	0.003786346
34.77033333	1137898.785	0.003815493
34.77416667	1160335.415	0.003890725
34.77816667	1133484.345	0.003800691
34.782	1086244.147	0.003642289
34.786	1090412.409	0.003656266
34.78983333	1101417.74	0.003693168
34.79383333	1115222.499	0.003739457
34.79766667	1125065.784	0.003772462
34.80166667	1158687.065	0.003885198
34.8055	1141691.342	0.003828209
34.8095	1134456.962	0.003803952
34.81333333	1102908.981	0.003698168
34.81716667	1096320.476	0.003676076
34.82116667	1083130.603	0.003631849
34.825	1089304.631	0.003652551
34.829	1089786.471	0.003654167
34.83283333	1100064.61	0.003688631
34.83683333	1138406.449	0.003817195
34.84066667	1092801.004	0.003664275
34.84466667	1066659.383	0.00357662
34.8485	1038234.863	0.003481309
34.8525	1015658.31	0.003405608
34.85633333	1028800.44	0.003449675
34.86016667	1029638.599	0.003452485
34.86416667	1053827.436	0.003533593
34.868	1061708.497	0.003560019
34.872	1078872.75	0.003617572
34.87583333	1078168.95	0.003615212
34.87983333	1086441.714	0.003642952
34.88366667	1080054.714	0.003621535
34.88766667	1069781.21	0.003587087
34.8915	1085979.442	0.003641402
34.8955	1058132.038	0.003548026
34.89933333	1042835.841	0.003496737
34.90316667	1050555.405	0.003522621
34.90716667	1047167.708	0.003511262
34.911	1023691.315	0.003432543
34.915	1006237.814	0.00337402
34.91883333	1013658.381	0.003398902
34.92283333	1036060.671	0.003474019
34.92666667	1053330.091	0.003531925
34.93066667	1010210.555	0.003387341
34.9345	1006752.711	0.003375746
34.93833333	991268.4295	0.003323826
34.94233333	1001859.512	0.003359339
34.94616667	1022072.413	0.003427115
34.95016667	1046505.15	0.00350904
34.954	1040988.819	0.003490543
34.958	1029947.91	0.003453522
34.96183333	1040195.854	0.003487885
34.96583333	1026462.251	0.003441834
34.96966667	1016960.605	0.003409974
34.9735	992688.4375	0.003328587
34.9775	978188.7297	0.003279968
34.98133333	976847.3151	0.00327547
34.98533333	945359.2248	0.003169888
34.98916667	967039.5099	0.003242584
34.99316667	1002225.053	0.003360565
34.997	1048787.122	0.003516692
35.001	1064231.401	0.003568478
35.00483333	1054219.27	0.003534907

Table 3. Samples Results STD

Retention time (minutes)	Indensity	Percentage of maximum peak area
35.00883333	1060298.371	0.00355529
35.01266667	1055280.321	0.003538464
35.0165	1050302.775	0.003521774
35.0205	1042448.66	0.003495438
35.02433333	1002663.581	0.003362035
35.02833333	979698.0519	0.003285029
35.03216667	969126.374	0.003249581
35.03616667	967810.3513	0.003245168
35.04	953253.4924	0.003196358
35.044	936322.8736	0.003139588
35.04783333	951070.3842	0.003189038
35.05183333	988104.9077	0.003313218
35.05566667	1009824.791	0.003386047
35.0595	1020661.939	0.003422385
35.0635	1009159.545	0.003383817
35.06733333	1002336.413	0.003360938
35.07133333	986702.9559	0.003308517
35.07516667	970664.9729	0.00325474
35.07916667	965781.7534	0.003238366
35.083	962544.5716	0.003227512
35.087	942824.4313	0.003161388
35.09083333	959204.7072	0.003216313
35.09466667	978177.8524	0.003279932
35.09866667	987115.3789	0.0033099
35.1025	1004250.912	0.003367357
35.1065	1005185.815	0.003370492
35.11033333	1034112.849	0.003467488
35.11433333	1017164.883	0.003410659
35.11816667	1019431.414	0.003418259
35.12216667	1004010.411	0.003366551
35.126	980902.9745	0.003289069
35.13	954997.8596	0.003202207
35.13383333	963536.9371	0.003230839
35.13766667	955969.4568	0.003205465
35.14166667	950720.3052	0.003187864
35.1455	960404.6421	0.003220336
35.1495	969763.1389	0.003251716
35.1535	964914.146	0.003235457
35.15733333	962956.6041	0.003228893
35.16133333	953379.7894	0.003196781
35.16516667	949713.2265	0.003184487
35.16916667	938316.3806	0.003146272
35.173	937047.3879	0.003142017
35.177	951427.7382	0.003190236
35.18083333	952880.9094	0.003195109
35.18466667	947122.1803	0.003175799
35.18866667	925783.7315	0.003104249
35.1925	935478.2006	0.003136755
35.1965	949112.2312	0.003182472
35.20033333	946684.1396	0.00317433
35.20433333	949107.2454	0.003182455
35.20816667	969582.6604	0.003251111
35.21216667	981690.6148	0.00329171
35.216	969057.9315	0.003249352
35.22	939924.353	0.003151664
35.22383333	916032.83	0.003071553
35.22766667	929257.1255	0.003115896
35.23166667	928631.9414	0.003113799
35.2355	926636.0826	0.003107107
35.2395	940875.0803	0.003154852
35.24333333	938880.487	0.003148164
35.24733333	943321.1368	0.003163054
35.25116667	934539.2434	0.003133607
35.255	937088.609	0.003142155
35.259	939698.9518	0.003150908
35.26283333	970629.2953	0.003254621
35.26683333	985278.8526	0.003303742
35.27066667	961343.7939	0.003223485
35.27466667	947721.748	0.003177809
35.2785	938486.6187	0.003146843
35.2825	931884.6387	0.003124706
35.28633333	907683.7821	0.003043558
35.29016667	904414.1752	0.003032595
35.29416667	929023.4225	0.003115112

Table 3. Samples Results STD

Retention time (minutes)	Indensity	Percentage of maximum peak area
35.298	949745.1158	0.003184594
35.302	921069.7391	0.003088442
35.30583333	919333.6751	0.003082621
35.30983333	911666.7704	0.003056913
35.31366667	916069.7633	0.003071677
35.31766667	934291.7879	0.003132777
35.3215	920820.1344	0.003087605
35.3255	905679.5779	0.003036838
35.32933333	898700.2003	0.003013435
35.33316667	893010.7015	0.002994358
35.33716667	902936.1022	0.003027638
35.341	910940.6289	0.003054478
35.345	910002.0793	0.003051331
35.34883333	885135.4596	0.002967951
35.35283333	879924.0233	0.002950477
35.35666667	883755.4237	0.002963324
35.36066667	862227.9297	0.00289114
35.3645	859911.9358	0.002883374
35.3685	871216.2581	0.002921278
35.37233333	880806.8493	0.002953437
35.37616667	896861.545	0.00300727
35.38016667	922344.047	0.003092715
35.384	947941.2903	0.003178545
35.388	942317.5466	0.003159688
35.39183333	937067.7917	0.003142085
35.39583333	907172.7347	0.003041844
35.39966667	884658.9531	0.002966353
35.40366667	899872.0278	0.003017364
35.4075	921919.495	0.003091292
35.4115	918856.7764	0.003081022
35.41533333	925724.7465	0.003104051
35.41916667	922653.8562	0.003093754
35.42316667	930323.9258	0.003119473
35.427	901365.4879	0.003022372
35.431	880175.6434	0.00295132
35.43483333	888350.0325	0.00297873
35.43883333	900577.7383	0.003019731
35.44266667	895490.9116	0.003002674
35.4465	871355.9412	0.002921747
35.4505	862049.969	0.002890543
35.45433333	845597.0594	0.002835375
35.45833333	858202.68	0.002877643
35.46216667	856999.5896	0.002873609
35.46616667	841042.7521	0.002820104
35.47	832670.3155	0.00279203
35.474	849682.5695	0.002849074
35.47783333	874176.4658	0.002931204
35.48183333	893274.033	0.00299524
35.48566667	902966.6262	0.003027741
35.4895	918995.053	0.003081486
35.4935	918298.2765	0.003079149
35.49733333	909492.88	0.003049624
35.50133333	888708.4813	0.002979932
35.50516667	876639.065	0.002939462
35.50916667	864168.1192	0.002897645
35.513	881954.3269	0.002957284
35.517	875500.0811	0.002935643
35.52083333	877351.1952	0.00294185
35.52483333	893892.9346	0.002997316
35.52866667	893254.3739	0.002995175
35.5325	923116.644	0.003095306
35.5365	942854.3217	0.003161488
35.54033333	928093.5396	0.003111994
35.54433333	892315.8712	0.002992028
35.54816667	851452.6973	0.002855009
35.55216667	868331.3898	0.002911605
35.556	843370.1344	0.002827908
35.56	855322.8365	0.002867986
35.56383333	861559.1284	0.002888897
35.56766667	871985.3158	0.002923857
35.57166667	876902.5623	0.002940345
35.5755	883704.0171	0.002963151
35.5795	888917.0999	0.002980631
35.58333333	892435.6916	0.002992429

Table 3. Samples Results STD

Retention time (minutes)	Indensity	Percentage of maximum peak area
35.58733333	897060.2284	0.003007936
35.59116667	893797.4286	0.002996995
35.59516667	873455.0809	0.002928785
35.599	844342.9758	0.00283117
35.603	846988.5745	0.002840041
35.60683333	876183.7644	0.002937935
35.61066667	884883.6255	0.002967107
35.61466667	867471.3331	0.002908721
35.6185	865582.8029	0.002902389
35.6225	884678.5729	0.002966419
35.62633333	903145.1495	0.003028339
35.63033333	909731.3594	0.003050424
35.63416667	922420.0764	0.00309297
35.63816667	921367.1037	0.003089439
35.642	896445.4853	0.003005875
35.646	868286.3172	0.002911454
35.64983333	851238.0449	0.002854289
35.65383333	845494.8	0.002835032
35.65766667	871006.004	0.002920573
35.6615	907407.083	0.00304263
35.6655	927153.9648	0.003108843
35.66933333	943574.3048	0.003163903
35.67333333	920250.9726	0.003085697
35.67716667	906925.9356	0.003041017
35.68116667	872211.1451	0.002924614
35.685	874333.2286	0.00293173
35.689	875254.946	0.002934821
35.69283333	882782.9972	0.002960063
35.69666667	911789.3144	0.003057324
35.70066667	928482.9585	0.0031133
35.7045	919246.1864	0.003082328
35.7085	923714.874	0.003097312
35.71233333	897740.8835	0.003010218
35.71633333	887259.6428	0.002975074
35.72016667	862714.1559	0.00289277
35.72416667	859551.5809	0.002882166
35.728	859608.9541	0.002882358
35.732	863528.689	0.002895501
35.73583333	883388.6029	0.002962094
35.73966667	883666.3254	0.002963025
35.74366667	901204.8511	0.003021833
35.7475	883494.6049	0.002962449
35.7515	867289.984	0.002908113
35.75533333	855728.2706	0.002869346
35.75933333	844675.4558	0.002832284
35.76316667	854960.7107	0.002866772
35.767	882989.5231	0.002960755
35.771	886697.2309	0.002973188
35.77483333	868630.1379	0.002912607
35.77883333	835471.1859	0.002801422
35.78266667	814253.64	0.002730277
35.78666667	833386.8362	0.002794432
35.7905	857563.6717	0.0028755
35.7945	860014.2705	0.002883717
35.79833333	871618.6096	0.002922628
35.80233333	865053.0176	0.002900612
35.80616667	844550.1355	0.002831864
35.81	861824.0606	0.002889785
35.814	864352.5501	0.002898264
35.81783333	851957.1672	0.002856701
35.82183333	865416.0767	0.00290183
35.82566667	846531.9009	0.002838509
35.82966667	857997.2931	0.002876954
35.8335	863324.067	0.002894815
35.8375	846778.6587	0.002839337
35.84133333	830359.0845	0.00278428
35.84533333	831425.5441	0.002787856
35.84916667	845229.5824	0.002834142
35.853	867768.0107	0.002909716
35.857	861911.3186	0.002890078
35.86083333	886106.1183	0.002971206
35.86483333	886343.3994	0.002972001
35.86866667	884598.9542	0.002966152
35.87266667	897489.7176	0.003009376

Table 3. Samples Results STD

Retention time (minutes)	Indensity	Percentage of maximum peak area
35.8765	890147.8109	0.002984758
35.8805	914493.1388	0.00306639
35.88433333	930131.1154	0.003118826
35.88833333	929575.2414	0.003116962
35.89216667	924271.3158	0.003099178
35.896	927983.7053	0.003111626
35.9	900764.9768	0.003020358
35.90383333	890981.1401	0.002987552
35.90783333	895283.5206	0.003001979
35.91166667	889972.7465	0.002984171
35.91566667	881143.6034	0.002954566
35.9195	883465.295	0.002962351
35.9235	897893.4318	0.00301073
35.92733333	914512.3669	0.003066455
35.93116667	869514.4137	0.002915572
35.93516667	880543.7358	0.002952554
35.939	879448.7304	0.002948883
35.943	873217.3533	0.002927988
35.94683333	885770.9446	0.002970082
35.95083333	893231.5964	0.002995098
35.95466667	869199.6389	0.002914517
35.95866667	868606.3269	0.002912527
35.9625	863319.6607	0.0028948
35.9665	872153.8409	0.002924422
35.97033333	885220.9825	0.002968238
35.97416667	863266.1782	0.002894621
35.97816667	862005.7217	0.002890395
35.982	871289.7762	0.002921525
35.986	899123.6739	0.003014855
35.98983333	875213.8729	0.002934683
35.99383333	879156.408	0.002947903
35.99766667	878590.1881	0.002946004
36.0015	880351.6804	0.00295191
36.0055	872262.7076	0.002924787
36.00933333	880791.5201	0.002953385
36.01333333	865962.5918	0.002903662
36.01716667	851229.4247	0.002854261
36.02116667	842802.253	0.002826003
36.025	827697.83	0.002775357
36.029	845278.6384	0.002834307
36.03283333	845979.0361	0.002836655
36.03683333	838970.7562	0.002813156
36.04066667	850119.4224	0.002850539
36.0445	857302.3157	0.002874624
36.0485	865356.4995	0.00290163
36.05233333	870438.8856	0.002918672
36.05633333	872313.73	0.002924958
36.06016667	870636.1576	0.002919333
36.06416667	860685.4912	0.002885968
36.068	859386.5345	0.002881612
36.072	859176.1768	0.002880907
36.07583333	849877.6265	0.002849728
36.07966667	863863.6836	0.002896625
36.08366667	874548.4663	0.002932452
36.0875	875559.993	0.002935843
36.0915	861079.4292	0.002887289
36.09533333	834197.9263	0.002797152
36.09933333	831479.2196	0.002788036
36.10316667	845514.0427	0.002835096
36.10716667	844394.2177	0.002831341
36.111	877783.5604	0.002943299
36.115	885901.1194	0.002970518
36.11883333	854227.0214	0.002864312
36.12283333	835505.8652	0.002801538
36.12666667	833528.5572	0.002794908
36.1305	848193.6708	0.002844081
36.1345	855095.7029	0.002867225
36.13833333	848800.0154	0.002846114
36.14233333	874782.0059	0.002933235
36.14616667	877192.071	0.002941316
36.15016667	869732.5827	0.002916304
36.154	865057.9331	0.002900629
36.158	842470.4933	0.002824891
36.16183333	829783.5381	0.00278235

Table 3. Samples Results STD

Retention time (minutes)	Indensity	Percentage of maximum peak area
36.16566667	818058.2233	0.002743034
36.16966667	819877.3683	0.002749134
36.1735	839998.815	0.002816603
36.1775	831548.0684	0.002788267
36.18133333	820183.9529	0.002750162
36.18533333	827706.1018	0.002775384
36.18916667	837458.9506	0.002808087
36.19316667	854344.0503	0.002864704
36.197	886762.1497	0.002973405
36.20083333	865527.1034	0.002902202
36.20483333	873113.0816	0.002927639
36.20866667	875387.6923	0.002935266
36.21266667	857258.8966	0.002874478
36.2165	842493.1018	0.002824967
36.2205	833964.8592	0.002796371
36.22433333	840495.4548	0.002818268
36.22833333	870237.667	0.002917997
36.23216667	879053.4952	0.002947558
36.236	882318.7275	0.002958506
36.24	863277.2972	0.002894658
36.24383333	866629.3985	0.002905898
36.24783333	877002.4926	0.00294068
36.25166667	866393.619	0.002905108
36.25566667	864571.5245	0.002898998
36.2595	869002.8877	0.002913857
36.2635	852605.1238	0.002858873
36.26733333	843961.4381	0.00282989
36.27133333	820370.3062	0.002750787
36.27516667	827270.0974	0.002773922
36.279	840949.7508	0.002819792
36.283	854703.3788	0.002865909
36.28683333	855305.4385	0.002867928
36.29083333	848922.2116	0.002846524
36.29466667	849658.9405	0.002848995
36.29866667	859576.0107	0.002882248
36.3025	841739.2682	0.002822439
36.3065	832155.9102	0.002790305
36.31033333	847683.5043	0.002842371
36.31433333	877546.9564	0.002942506
36.31816667	883168.3743	0.002961355
36.322	873572.765	0.00292918
36.326	890607.6251	0.0029863
36.32983333	890900.5505	0.002987282
36.33383333	882171.9503	0.002958014
36.33766667	914114.1252	0.003065119
36.34166667	935143.4234	0.003135633
36.3455	951827.6024	0.003191577
36.3495	950785.564	0.003188083
36.35333333	926032.4208	0.003105083
36.35716667	933329.0116	0.003129549
36.36116667	925247.8325	0.003102452
36.365	920609.7603	0.0030869
36.369	886832.9524	0.002973643
36.37283333	890790.8104	0.002986914
36.37683333	890882.4877	0.002987221
36.38066667	902695.4243	0.003026831
36.3845	908327.631	0.003045717
36.3885	900802.6991	0.003020485
36.39233333	891956.4126	0.002990822
36.39633333	878785.4141	0.002946659
36.40016667	876734.6601	0.002939782
36.40416667	874832.3767	0.002933404
36.408	877824.2558	0.002943436
36.412	866382.0114	0.002905069
36.41583333	864440.4645	0.002898559
36.41983333	852391.4499	0.002858157
36.42366667	829778.591	0.002782334
36.4275	858674.7322	0.002879225
36.4315	871460.7189	0.002922098
36.43533333	883769.2984	0.00296337
36.43933333	883999.7436	0.002964143
36.44316667	867368.5763	0.002908377
36.44716667	842689.6394	0.002825626
36.451	835252.1593	0.002800687

Table 3. Samples Results STD

Retention time (minutes)	Indensity	Percentage of maximum peak area
36.455	840826.9434	0.00281938
36.45883333	836619.4333	0.002805272
36.46283333	852257.9939	0.002857709
36.46666667	856382.3403	0.002871539
36.4705	845265.5275	0.002834263
36.4745	832233.1834	0.002790564
36.47833333	819537.0661	0.002747993
36.48233333	806035.7562	0.002702721
36.48616667	803876.4022	0.002695481
36.49016667	817364.4267	0.002740708
36.494	834200.4731	0.002797161
36.498	825964.1831	0.002769544
36.50183333	812039.3993	0.002722852
36.50566667	826097.9567	0.002769992
36.50966667	820896.6478	0.002752552
36.5135	828073.5881	0.002776617
36.5175	824031.0223	0.002763061
36.52133333	830348.5638	0.002784245
36.52533333	843646.2579	0.002828833
36.52916667	813398.1333	0.002727408
36.53316667	829781.625	0.002782344
36.537	842331.4121	0.002824425
36.541	844287.2138	0.002830983
36.54483333	842200.2249	0.002823985
36.54866667	845287.2329	0.002834336
36.55266667	846540.7233	0.002838539
36.5565	852148.0563	0.002857341
36.5605	858585.1842	0.002878925
36.56433333	870591.7158	0.002919184
36.56833333	890538.7948	0.002986069
36.57216667	913401.0255	0.003062728
36.57616667	928096.7572	0.003112005
36.58	929533.7334	0.003116823
36.584	935465.5915	0.003136713
36.58783333	938254.8174	0.003146066
36.59166667	930150.7319	0.003118892
36.59566667	935969.7541	0.003138404
36.5995	944726.7251	0.003167767
36.6035	962074.0874	0.003225934
36.60733333	959071.2484	0.003215865
36.61133333	948886.3464	0.003181714
36.61516667	943010.5956	0.003162012
36.619	950249.4059	0.003186285
36.623	939400.5773	0.003149908
36.62683333	968152.9719	0.003246317
36.63083333	965632.4932	0.003237866
36.63466667	954169.9143	0.003199431
36.63866667	939759.6365	0.003151112
36.6425	942281.9363	0.003159569
36.6465	942926.2811	0.00316173
36.65033333	906023.1518	0.00303799
36.65433333	893574.5303	0.002996248
36.65816667	871441.0353	0.002922032
36.662	873137.1479	0.002927719
36.666	903266.873	0.003028748
36.66983333	885405.0164	0.002968855
36.67383333	880728.4471	0.002953174
36.67766667	888533.6513	0.002979345
36.68166667	913367.4535	0.003062616
36.6855	915113.6242	0.003068471
36.6895	897185.1553	0.003008355
36.69333333	867317.2601	0.002908205
36.69733333	854805.2943	0.002866251
36.70116667	840330.0241	0.002817714
36.705	851682.8061	0.002855781
36.709	834003.7369	0.002796501
36.71283333	842676.3646	0.002825581
36.71683333	831050.7373	0.002786599
36.72066667	838521.4919	0.00281165
36.72466667	844800.4954	0.002832704
36.7285	855485.5838	0.002868532
36.7325	861479.8326	0.002888631
36.73633333	869559.4114	0.002915723
36.74016667	855463.6571	0.002868458

Table 3. Samples Results STD

Retention time (minutes)	Indensity	Percentage of maximum peak area
36.74416667	847747.6288	0.002842586
36.748	826510.4231	0.002771375
36.752	818379.8953	0.002744113
36.75583333	820181.3205	0.002750153
36.75983333	820409.4134	0.002750918
36.76366667	835175.2962	0.002800429
36.76766667	816014.4339	0.002736181
36.7715	833197.4824	0.002793798
36.7755	847461.194	0.002841625
36.77933333	865984.8046	0.002903737
36.78333333	888267.1298	0.002978452
36.78716667	860150.4771	0.002884174
36.79116667	841387.8574	0.002821261
36.795	824244.6759	0.002763778
36.799	820391.8971	0.002750859
36.80283333	802202.258	0.002689867
36.80683333	791563.0552	0.002654193
36.81066667	794926.4466	0.002665471
36.8145	803353.7008	0.002693728
36.8185	809147.6495	0.002713156
36.82233333	821731.2656	0.00275535
36.82633333	826893.8448	0.002772661
36.83016667	826746.3813	0.002772166
36.83416667	852378.2736	0.002858113
36.838	860867.9717	0.00288658
36.842	847817.5561	0.00284282
36.84583333	841538.1743	0.002821765
36.84983333	855818.4179	0.002869648
36.85366667	884736.2781	0.002966613
36.8575	914883.855	0.0030677
36.8615	904059.91	0.003031407
36.86533333	915293.5186	0.003069074
36.86933333	911918.8673	0.003057759
36.87316667	866655.9981	0.002905987
36.87716667	863575.869	0.002895659
36.881	879403.1098	0.00294873
36.885	901344.1132	0.0030223
36.88883333	911346.8724	0.003055841
36.89266667	920089.5265	0.003085156
36.89666667	911765.665	0.003057245
36.9005	907422.4696	0.003042682
36.9045	908999.1805	0.003047969
36.90833333	914475.5414	0.003066331
36.91233333	918211.0775	0.003078857
36.91616667	909040.8029	0.003048108
36.92016667	894546.2979	0.002999507
36.924	868644.2932	0.002912654
36.928	864029.4334	0.00289718
36.93183333	863361.2139	0.00289494
36.93566667	887531.1157	0.002975984
36.93966667	885458.6045	0.002969035
36.9435	862259.4308	0.002891245
36.9475	869628.8604	0.002915956
36.95133333	901377.7333	0.003022413
36.95533333	909207.5872	0.003048667
36.95933333	905867.6011	0.003037468
36.96316667	893576.2962	0.002996254
36.96716667	888035.0339	0.002977674
36.971	901973.0212	0.003024409
36.975	892413.0722	0.002992354
36.97883333	878300.1448	0.002945031
36.98283333	874775.6617	0.002933214
36.98666667	876747.259	0.002939824
36.9905	861655.2402	0.002889219
36.9945	873180.0867	0.002927863
36.99833333	877368.0423	0.002941906
37.00233333	874680.0642	0.002932893
37.00616667	876172.1416	0.002937896
37.01016667	851627.5469	0.002855595
37.014	828256.8702	0.002777231
37.018	834394.1536	0.00279781
37.02183333	833762.4058	0.002795692
37.02583333	826205.4944	0.002770353
37.02966667	843623.3185	0.002828756

Table 3. Samples Results STD

Retention time (minutes)	Indensity	Percentage of maximum peak area
37.0335	864334.0175	0.002898202
37.0375	861332.09	0.002888136
37.04133333	858381.056	0.002878241
37.04533333	848351.9744	0.002844612
37.04916667	839082.1369	0.002813529
37.05316667	830019.4515	0.002783141
37.057	828747.3181	0.002778876
37.061	830780.494	0.002785693
37.06483333	844105.1249	0.002830372
37.06866667	857067.845	0.002873837
37.07266667	858527.6886	0.002878732
37.0765	861763.5997	0.002889583
37.0805	882943.1706	0.0029606
37.08433333	912064.0558	0.003058245
37.08833333	930075.8419	0.003118641
37.09216667	928497.9355	0.00311335
37.09616667	917114.3718	0.00307518
37.1	907721.8207	0.003043685
37.104	891108.3069	0.002987979
37.10783333	920105.5584	0.003085209
37.11166667	928605.877	0.003113712
37.11566667	946619.9072	0.003174115
37.1195	950076.4997	0.003185705
37.1235	945483.9355	0.003170306
37.12733333	932492.4215	0.003126744
37.13133333	931229.4992	0.003122509
37.13516667	913419.8324	0.003062791
37.139	906903.0936	0.00304094
37.143	902227.3754	0.003025262
37.14683333	898683.2549	0.003013378
37.15083333	899694.304	0.003016768
37.15466667	891922.3631	0.002990708
37.15866667	914608.6635	0.003066778
37.1625	907086.6331	0.003041556
37.1665	893284.3651	0.002995275
37.17033333	852145.9111	0.002857334
37.17433333	829085.518	0.00278001
37.17816667	819966.8042	0.002749434
37.182	830586.39	0.002785042
37.186	822213.5064	0.002756967
37.18983333	803556.9569	0.00269441
37.19383333	786022.8865	0.002635616
37.19766667	793376.5731	0.002660274
37.20166667	813033.4208	0.002726185
37.2055	810622.2404	0.0027181
37.2095	798050.3605	0.002675946
37.21333333	803284.5977	0.002693497
37.21716667	792886.9123	0.002658632
37.22116667	804967.2574	0.002699139
37.225	798898.8008	0.002678791
37.229	809487.4976	0.002714296
37.23283333	820235.6781	0.002750335
37.23683333	821734.5565	0.002755361
37.24066667	813333.0229	0.00272719
37.24466667	816399.8203	0.002737473
37.2485	811208.7598	0.002720067
37.2525	789213.2287	0.002646314
37.25633333	774086.9437	0.002595594
37.26016667	768518.0811	0.002576921
37.26416667	784013.7965	0.00262888
37.268	798553.7295	0.002677633
37.272	824810.0172	0.002765674
37.27583333	813984.4743	0.002729374
37.27983333	812264.635	0.002723608
37.28366667	812465.0908	0.00272428
37.28766667	815164.062	0.00273333
37.2915	807927.0325	0.002709063
37.2955	808450.8132	0.002710819
37.29933333	820333.544	0.002750663
37.30316667	822707.4389	0.002758623
37.30716667	827296.5944	0.002774011
37.311	837858.4708	0.002809426
37.315	840721.3186	0.002819026
37.31883333	846661.6444	0.002838944

Table 3. Samples Results STD

Retention time (minutes)	Indensity	Percentage of maximum peak area
37.32283333	874631.4307	0.00293273
37.32666667	907824.0992	0.003044028
37.3305	915734.2823	0.003070552
37.3345	887151.7094	0.002974712
37.3385	882573.6538	0.002959361
37.34233333	889490.9339	0.002982555
37.34616667	884046.6436	0.0029643
37.35016667	892694.478	0.002993297
37.354	888248.3668	0.002978389
37.358	882809.4894	0.002960152
37.36183333	885282.2768	0.002968443
37.36583333	882403.8809	0.002958792
37.36966667	884252.3772	0.00296499
37.3735	876040.003	0.002937453
37.3775	866075.7007	0.002904042
37.38133333	880552.0551	0.002952582
37.38533333	884514.725	0.00296587
37.38916667	890262.6836	0.002985143
37.39316667	868881.0366	0.002913448
37.397	839903.64	0.002816284
37.401	829753.323	0.002782249
37.40483333	858945.7174	0.002880134
37.40866667	850995.3927	0.002853476
37.41266667	840608.4137	0.002818647
37.4165	825351.9761	0.002767491
37.4205	822610.8781	0.0027583
37.42433333	804373.1778	0.002697147
37.42833333	816097.7358	0.00273646
37.43216667	834080.0126	0.002796757
37.43616667	806670.9171	0.002704851
37.44	815352.0956	0.00273396
37.444	786525.6415	0.002637302
37.44783333	790822.0352	0.002651708
37.45183333	814643.3724	0.002731584
37.45566667	825188.8432	0.002766944
37.4595	823941.5381	0.002762761
37.4635	807900.9265	0.002708976
37.46733333	768008.7111	0.002575213
37.47133333	778545.8861	0.002610545
37.47516667	780888.5206	0.0026184
37.47916667	790533.8876	0.002650742
37.483	798275.9556	0.002676702
37.487	800410.7519	0.00268386
37.49083333	809543.3227	0.002714483
37.49466667	797763.2043	0.002674983
37.49866667	791362.627	0.002653521
37.5025	809947.3691	0.002715838
37.5065	791175.878	0.002652895
37.51033333	785361.8147	0.0026334
37.51433333	779334.9577	0.002613191
37.51816667	798290.1745	0.00267675
37.52216667	786164.2416	0.00263609
37.526	802634.2276	0.002691316
37.53	801016.9869	0.002685893
37.53383333	806903.1357	0.00270563
37.53766667	774238.5565	0.002596102
37.54166667	797372.9129	0.002673674
37.5455	789185.7121	0.002646222
37.5495	793838.9076	0.002661824
37.55333333	808110.3703	0.002709678
37.55733333	808607.229	0.002711344
37.56116667	805046.6185	0.002699405
37.565	805635.6562	0.00270138
37.569	800972.6823	0.002685744
37.57283333	814983.4809	0.002732724
37.57683333	815807.5019	0.002735487
37.58066667	807027.1481	0.002706046
37.58466667	806156.2899	0.002703126
37.5885	811583.7588	0.002721325
37.5925	798894.0568	0.002678775
37.59633333	786747.1234	0.002638045
37.60033333	767094.8837	0.002572149
37.60416667	772798.3161	0.002591273
37.608	778613.2973	0.002610771

Table 3. Samples Results STD

Retention time (minutes)	Indensity	Percentage of maximum peak area
37.612	773896.5568	0.002594955
37.61583333	788448.5899	0.00264375
37.61983333	774190.5117	0.002595941
37.62366667	753590.5181	0.002526867
37.62766667	756146.7984	0.002535439
37.6315	782971.7546	0.002625386
37.6355	791809.0314	0.002655018
37.63933333	778026.3164	0.002608803
37.64333333	772547.5042	0.002590432
37.64716667	758645.5766	0.002543817
37.651	765239.288	0.002565927
37.655	793959.2723	0.002662228
37.65883333	796580.4822	0.002671017
37.66283333	772622.7075	0.002590684
37.66666667	772763.6165	0.002591157
37.67066667	759645.8405	0.002547171
37.6745	759797.2244	0.002547679
37.6785	787192.3068	0.002639537
37.68233333	798106.6353	0.002676134
37.68633333	776860.8656	0.002604895
37.69016667	761220.2116	0.00255245
37.694	787525.8798	0.002640656
37.698	800644.564	0.002684644
37.70183333	803548.607	0.002694382
37.70583333	809658.8637	0.00271487
37.70966667	789391.1838	0.002646911
37.71366667	760931.9796	0.002551484
37.7175	737754.9447	0.002473769
37.7215	746004.3866	0.00250143
37.72533333	752166.2466	0.002522091
37.72933333	740369.5007	0.002482536
37.73316667	727547.1964	0.002439541
37.737	745952.089	0.002501255
37.741	745889.601	0.002501045
37.74483333	754320.9955	0.002529317
37.74883333	772048.5243	0.002588759
37.75266667	761086.2702	0.002552001
37.75666667	763676.7022	0.002560687
37.7605	775742.0207	0.002601143
37.7645	777079.5208	0.002605628
37.76833333	766543.6741	0.0025703
37.77233333	739378.0993	0.002479211
37.77616667	736246.5984	0.002468711
37.78	729470.4989	0.00244599
37.784	741669.4527	0.002486895
37.78783333	760035.8455	0.002548479
37.79183333	771612.4929	0.002587297
37.79566667	771738.7034	0.00258772
37.79966667	756897.1771	0.002537955
37.8035	747559.9062	0.002506646
37.8075	761088.2444	0.002552008
37.81133333	750788.1012	0.00251747
37.81516667	763869.6011	0.002561334
37.81916667	758378.1095	0.00254292
37.823	759087.3249	0.002545299
37.827	762600.9971	0.00255708
37.83083333	781359.069	0.002619978
37.83483333	776918.4038	0.002605088
37.83866667	759449.5742	0.002546513
37.8425	757864.4521	0.002541198
37.8465	735721.1015	0.002466949
37.85033333	739402.3184	0.002479293
37.85433333	743548.9559	0.002493197
37.85816667	769076.6355	0.002578794
37.86216667	788895.119	0.002645247
37.866	781653.5724	0.002620965
37.87	759008.8874	0.002545036
37.87383333	750378.5023	0.002516097
37.87766667	762275.1913	0.002555988
37.88166667	767498.5615	0.002573502
37.8855	763738.9525	0.002560896
37.8895	752647.8093	0.002523706
37.89333333	761421.6605	0.002553126
37.89733333	774905.7255	0.002598339

Table 3. Samples Results STD

Retention time (minutes)	Indensity	Percentage of maximum peak area
37.90116667	771014.0729	0.00258529
37.90516667	754583.0931	0.002530195
37.909	762968.9564	0.002558314
37.913	765623.5456	0.002567215
37.91683333	766615.3688	0.002570541
37.92066667	765377.1921	0.002566389
37.92466667	785184.2827	0.002632804
37.9285	794989.1413	0.002665681
37.9325	790117.206	0.002649345
37.93633333	787703.6118	0.002641252
37.94033333	770536.8603	0.00258369
37.94416667	752077.4261	0.002521794
37.94816667	763980.0959	0.002561704
37.952	779788.0937	0.00261471
37.956	784950.0422	0.002632019
37.95983333	774414.5145	0.002596692
37.96366667	761982.4297	0.002555006
37.96766667	757380.4945	0.002539575
37.9715	758995.0888	0.002544989
37.9755	755011.175	0.002531631
37.97933333	762544.2981	0.00255689
37.98333333	763619.5249	0.002560495
37.98716667	757395.365	0.002539625
37.99116667	769922.0378	0.002581628
37.995	797490.9579	0.00267407
37.999	809533.2015	0.002714449
38.00283333	805691.9549	0.002701569
38.00666667	810197.0582	0.002716675
38.01066667	805342.8498	0.002700398
38.0145	809793.6689	0.002715322
38.0185	810892.4281	0.002719006
38.02233333	803720.5247	0.002694958
38.02633333	793730.9333	0.002661462
38.03016667	800064.8102	0.0026827
38.03416667	812244.5142	0.00272354
38.038	815018.9	0.002732843
38.042	824270.4182	0.002763864
38.04583333	825510.5612	0.002768023
38.04966667	826243.6978	0.002770481
38.05366667	830452.5704	0.002784594
38.0575	831706.119	0.002788797
38.0615	824550.7926	0.002764804
38.06533333	806148.9335	0.002703101
38.06933333	782513.2416	0.002623848
38.07316667	776886.925	0.002604982
38.077	752210.5971	0.00252224
38.081	738901.361	0.002477613
38.08483333	760604.2474	0.002550385
38.08883333	769357.4259	0.002579735
38.09266667	789232.9803	0.00264638
38.09666667	795860.938	0.002668604
38.1005	792092.2071	0.002655967
38.1045	812533.5124	0.002724509
38.10833333	837150.3327	0.002807052
38.11233333	833042.5576	0.002793278
38.11616667	845400.2258	0.002834715
38.12	818750.8675	0.002745357
38.124	797033.7127	0.002672537
38.12783333	783124.8015	0.002625899
38.13183333	779816.6561	0.002614806
38.13566667	794021.816	0.002662438
38.13966667	789257.2583	0.002646461
38.1435	808687.7169	0.002711614
38.1475	799236.8798	0.002679924
38.15133333	783222.0633	0.002626225
38.15533333	773814.8905	0.002594682
38.15916667	762585.6398	0.002557029
38.163	755828.8203	0.002534372
38.167	743843.5068	0.002494184
38.17083333	767961.1728	0.002575053
38.17483333	779831.8689	0.002614857
38.17866667	803095.0054	0.002692861
38.18266667	811322.5036	0.002720449
38.1865	825950.2281	0.002769497

Table 3. Samples Results STD

Retention time (minutes)	Indensity	Percentage of maximum peak area
38.1905	818327.7465	0.002743938
38.19433333	819967.919	0.002749437
38.19816667	831118.3479	0.002786826
38.20216667	818189.1757	0.002743473
38.206	819158.0867	0.002746722
38.21	813874.2547	0.002729005
38.21383333	815312.2349	0.002733826
38.21783333	820000.066	0.002749545
38.22166667	831300.3621	0.002787436
38.22566667	822260.6232	0.002757125
38.2295	803242.0624	0.002693354
38.2335	791411.0736	0.002653683
38.23733333	784809.8651	0.002631549
38.24116667	786807.9237	0.002638249
38.24516667	793818.2515	0.002661755
38.249	807514.7439	0.002707681
38.253	847733.5107	0.002842538
38.25683333	858327.7264	0.002878062
38.26083333	875078.1136	0.002934228
38.26466667	898561.4349	0.00301297
38.26866667	925561.5806	0.003103504
38.2725	984248.4946	0.003300287
38.2765	1072511.677	0.003596243
38.28033333	1173805.547	0.003935892
38.28416667	1351526.868	0.00453181
38.28816667	1616489.97	0.005420259
38.292	1935000.364	0.006488257
38.296	2366090.008	0.007933745
38.29983333	2886911.225	0.009680113
38.30383333	3612516.583	0.012113143
38.30766667	4525225.964	0.015173552
38.31166667	5680768.476	0.019048206
38.3155	7110208.846	0.023841268
38.3195	8885002.477	0.029792335
38.32333333	10947121.95	0.036706835
38.32716667	13374277.59	0.04484534
38.33116667	16056108.98	0.053837799
38.335	18998528.3	0.063704036
38.339	22259172.03	0.074637312
38.34283333	25674736.42	0.086090054
38.34683333	29363151.26	0.098457692
38.35066667	33194763.36	0.111305484
38.3545	37177529.92	0.124660114
38.3585	40980054.76	0.137410374
38.36233333	44681370.44	0.149821269
38.36633333	48035559.45	0.161068213
38.37016667	50971148.59	0.170911548
38.37416667	53502089.2	0.179398054
38.378	55577831.15	0.186358232
38.382	57080228.41	0.191395926
38.38583333	57920727.02	0.194214205
38.38983333	58336607.5	0.195608695
38.39366667	58008773.86	0.194509435
38.3975	57094129.76	0.191442539
38.4015	55614533.4	0.186481299
38.40533333	53608617.33	0.179755254
38.40933333	51190222.64	0.171646126
38.41316667	48470893.13	0.162527932
38.41716667	45484506.75	0.152514269
38.421	42431784.16	0.142278173
38.425	39269229.1	0.131673798
38.42883333	36101385.41	0.12105169
38.43283333	33092786.48	0.110963545
38.43666667	30272034.04	0.10150527
38.4405	27748211.9	0.093042632
38.4445	25455966.57	0.085356496
38.44833333	23322054.45	0.078201267
38.45233333	21352549.35	0.071597313
38.45616667	19546814.77	0.065542497
38.46016667	17897548.28	0.060012336
38.464	16431186.31	0.055095472
38.468	15117535.74	0.050690665
38.47183333	13961921.55	0.046815771
38.47566667	12933609.72	0.043367735

Table 3. Samples Results STD

Retention time (minutes)	Indensity	Percentage of maximum peak area
38.47966667	12034293.05	0.040352233
38.4835	11138766.43	0.037349439
38.4875	10327313.28	0.034628553
38.49133333	9619559.712	0.032255381
38.49533333	8953885.585	0.030023307
38.49916667	8329571.534	0.027929917
38.50316667	7816610.915	0.026209907
38.507	7386339.201	0.024767161
38.511	6978297.481	0.023398955
38.51483333	6560426.012	0.021997789
38.51866667	6192531.268	0.0207642
38.52266667	5862812.258	0.019658618
38.5265	5617097.494	0.01883471
38.5305	5329500.854	0.017870369
38.53433333	5088017.61	0.017060651
38.53833333	4885413.029	0.016381297
38.54216667	4710047.156	0.015793277
38.546	4538423.514	0.015217805
38.55	4385495.494	0.014705021
38.55383333	4239829.946	0.01421659
38.55783333	4019778.106	0.013478733
38.56166667	3849757.215	0.012908635
38.56566667	3728339.465	0.012501509
38.5695	3603684.704	0.012083529
38.5735	3467361.311	0.011626422
38.57733333	3363754.543	0.011279018
38.58133333	3261556.812	0.010936338
38.58516667	3185115.456	0.010680022
38.589	3134517.649	0.010510363
38.593	3048396.192	0.010221589
38.597	2954657.079	0.009907272
38.60083333	2896030.281	0.00971069
38.60483333	2803171.346	0.009399324
38.60866667	2693730.371	0.009032357
38.61266667	2601682.858	0.008723712
38.6165	2548995.84	0.008547047
38.6205	2468174.704	0.008276046
38.62433333	2420992.313	0.008117838
38.62833333	2396510.344	0.008035748
38.63216667	2343462.425	0.007857873
38.636	2326134.984	0.007799772
38.64	2258114.33	0.007571692
38.64383333	2254536.297	0.007559694
38.64783333	2220046.084	0.007444045
38.65166667	2201995.353	0.007383519
38.65566667	2171383.087	0.007280873
38.6595	2141569.289	0.007180904
38.6635	2101059.329	0.00704507
38.66733333	2084405.531	0.006989228
38.67133333	2051855.281	0.006880084
38.67516667	2004922.496	0.006722713
38.679	1953339.272	0.006549749
38.683	1933545.502	0.006483379
38.68683333	1905645.311	0.006389826
38.69083333	1858616.71	0.006232135
38.69466667	1831501.166	0.006141213
38.69866667	1850197.427	0.006203904
38.7025	1851807.564	0.006209303
38.7065	1821408.844	0.006107373
38.71033333	1809626.343	0.006067865
38.71416667	1774125.714	0.005948828
38.71816667	1713401.391	0.005745213
38.722	1680854.508	0.005636079
38.726	1697493.732	0.005691873
38.72983333	1675108.064	0.005616811
38.73383333	1653960.437	0.005545901
38.73766667	1607688.475	0.005390746
38.7415	1569469.367	0.005262594
38.7455	1596466.348	0.005353117
38.74933333	1611107.94	0.005402212
38.75333333	1616791.616	0.00542127
38.75716667	1595959.796	0.005351419
38.76116667	1569946.533	0.005264194
38.765	1547184.313	0.005187869

Table 3. Samples Results STD

Retention time (minutes)	Indensity	Percentage of maximum peak area
38.769	1555154.719	0.005214595
38.77283333	1524143.472	0.005110611
38.77683333	1548993.604	0.005193936
38.78066667	1560970.131	0.005234095
38.7845	1537685.776	0.00515602
38.7885	1504997.697	0.005046413
38.79233333	1459998.982	0.004895528
38.79633333	1437102.148	0.004818753
38.80016667	1442541.409	0.004836991
38.80416667	1427669.197	0.004787123
38.808	1399719.052	0.004693403
38.812	1404621.748	0.004709842
38.81583333	1419553.193	0.004759909
38.81966667	1405921.501	0.004714201
38.82366667	1364983.42	0.004576931
38.8275	1352560.671	0.004535276
38.8315	1377619.066	0.0046193
38.83533333	1335024.655	0.004476476
38.83933333	1323699.735	0.004438503
38.84316667	1315177.528	0.004409927
38.84716667	1331047.766	0.004463141
38.851	1311983.49	0.004399217
38.855	1294685.226	0.004341214
38.85883333	1284317.553	0.00430645
38.86266667	1270676.413	0.00426071
38.86666667	1294437.453	0.004340383
38.8705	1314921.042	0.004409067
38.8745	1306936.229	0.004382293
38.87833333	1298116.087	0.004352718
38.88233333	1273284.022	0.004269453
38.88616667	1265689.778	0.004243989
38.89016667	1245345.352	0.004175772
38.894	1269513.606	0.004256811
38.898	1264607.685	0.004240361
38.90183333	1248825.594	0.004187442
38.90566667	1246927.372	0.004181077
38.90966667	1292004.408	0.004332225
38.9135	1276979.865	0.004281846
38.9175	1253138.204	0.004201902
38.92133333	1248820.658	0.004187425
38.92533333	1227401.136	0.004115603
38.92916667	1225081.812	0.004107826
38.933	1194073.35	0.004003852
38.937	1197009.765	0.004013698
38.94083333	1171615.736	0.003928549
38.94483333	1149203.787	0.003853399
38.94866667	1137787.336	0.003815119
38.95266667	1140356.886	0.003823735
38.9565	1135260.703	0.003806647
38.9605	1131749.129	0.003794872
38.96433333	1147299.796	0.003847015
38.96833333	1149587.826	0.003854687
38.97216667	1182146.723	0.003963861
38.976	1158056.911	0.003883085
38.98	1155092.853	0.003873146
38.98383333	1154642.923	0.003871637
38.98783333	1170917.545	0.003926208
38.99166667	1196683.653	0.004012604
38.99566667	1199137.988	0.004020834
38.9995	1201610.522	0.004029125
39.0035	1181524.507	0.003961774
39.00733333	1136315.718	0.003810184
39.01116667	1150465.362	0.00385763
39.01516667	1130417.789	0.003790408
39.019	1124287.039	0.003769851
39.023	1116859.937	0.003744947
39.02683333	1130413.412	0.003790393
39.03083333	1165523.462	0.003908121
39.03466667	1178614.733	0.003952017
39.03866667	1138912.067	0.00381889
39.0425	1105055.139	0.003705364
39.0465	1085170.316	0.003638689
39.05033333	1074095.539	0.003601554
39.05416667	1057102.277	0.003544574

Table 3. Samples Results STD

Retention time (minutes)	Indensity	Percentage of maximum peak area
39.05816667	1082978.537	0.003631339
39.062	1132581.373	0.003797663
39.066	1115446.681	0.003740208
39.06983333	1105296.171	0.003706173
39.07383333	1093788.381	0.003667586
39.07766667	1089631.887	0.003653649
39.0815	1069101.378	0.003584808
39.0855	1082194.445	0.00362871
39.08933333	1090798.494	0.00365756
39.09333333	1103315.272	0.003699531
39.09716667	1088963.299	0.003651407
39.10116667	1077938.421	0.003614439
39.105	1085080.034	0.003638386
39.109	1097741.275	0.00368084
39.11283333	1098332.804	0.003682824
39.11683333	1089407.022	0.003652895
39.12066667	1069429.522	0.003585908
39.1245	1058753.359	0.00355011
39.1285	1039224.735	0.003484628
39.13233333	1048542.297	0.003515871
39.13633333	1043439.947	0.003498762
39.14016667	1056145.345	0.003541365
39.14416667	1060549.625	0.003556133
39.148	1069172.957	0.003585048
39.152	1063068.783	0.00356458
39.15583333	1068460.912	0.00358266
39.15983333	1036488.935	0.003475455
39.16366667	1014585.656	0.003402011
39.1675	1000260.747	0.003353978
39.1715	994081.3451	0.003333258
39.17533333	1022986.827	0.003430181
39.17933333	1051039.579	0.003524245
39.18316667	1032994.33	0.003463737
39.18716667	1048900.593	0.003517072
39.191	1045408.054	0.003505362
39.195	1060144.512	0.003554775
39.19883333	1050273.553	0.003521676
39.20266667	1058166.121	0.003548141
39.20666667	1031913.641	0.003460113
39.2105	1025025.311	0.003437016
39.2145	1019156.814	0.003417339
39.21833333	1008426.52	0.003381359
39.22233333	993629.9146	0.003331744
39.22616667	1002420.056	0.003361218
39.23016667	984912.7431	0.003302515
39.234	994670.9299	0.003335235
39.238	993012.5524	0.003329674
39.24183333	989846.2018	0.003319057
39.24566667	989666.623	0.003318455
39.24966667	998520.3298	0.003348142
39.2535	1006614.506	0.003375283
39.2575	1008311.111	0.003380972
39.26133333	1019044.123	0.003416961
39.26533333	1013823.111	0.003399454
39.26916667	994815.6841	0.00333572
39.27316667	1004528.573	0.003368288
39.277	990283.4418	0.003320523
39.281	1009801.032	0.003385968
39.28483333	1022562.969	0.00342876
39.28866667	1002232.952	0.003360591
39.29266667	975521.1955	0.003271024
39.2965	972604.576	0.003261244
39.3005	988335.8782	0.003313993
39.30433333	1008294.162	0.003380915
39.30833333	998726.03	0.003348832
39.31216667	995012.7949	0.003336381
39.316	998201.2841	0.003347072
39.32	982305.9621	0.003293774
39.32383333	993675.5651	0.003331897
39.32783333	993006.1899	0.003329653
39.33166667	982478.5829	0.003294353
39.33566667	981256.6468	0.003290255
39.3395	985882.5007	0.003305766
39.3435	1005479.19	0.003371476

Table 3. Samples Results STD

Retention time (minutes)	Indensity	Percentage of maximum peak area
39.34733333	996109.6711	0.003340059
39.35133333	973495.762	0.003264232
39.35516667	952710.6126	0.003194537
39.359	942427.352	0.003160057
39.363	954713.3762	0.003201253
39.36683333	992676.2154	0.003328546
39.37083333	1006924.912	0.003376324
39.37466667	1003586.308	0.003365129
39.37866667	976848.6668	0.003275475
39.3825	984857.6298	0.00330233
39.3865	978458.5835	0.003280873
39.39033333	957787.9562	0.003211562
39.39416667	945901.142	0.003171705
39.39816667	949406.4209	0.003183458
39.402	968281.2395	0.003246747
39.406	963183.7223	0.003229655
39.40983333	959860.5177	0.003218512
39.41383333	953484.5479	0.003197133
39.41766667	929028.9651	0.00311513
39.42166667	962200.4723	0.003226358
39.4255	956804.9841	0.003208266
39.4295	967537.1188	0.003244252
39.43333333	968877.8002	0.003248748
39.43716667	985375.3573	0.003304066
39.44116667	981186.4375	0.00329002
39.445	947048.3862	0.003175551
39.449	920433.1769	0.003086308
39.45283333	895013.1418	0.003001072
39.45683333	900471.101	0.003019373
39.46066667	916190.5209	0.003072082
39.46466667	933925.3897	0.003131549
39.4685	932029.3628	0.003125191
39.4725	939984.2907	0.003151865
39.47633333	967531.0405	0.003244232
39.48016667	958681.0012	0.003214557
39.48416667	951585.5417	0.003190765
39.488	924737.9001	0.003100742
39.492	926038.0375	0.003105102
39.49583333	922874.844	0.003094495
39.49983333	936116.6801	0.003138896
39.50366667	940514.5586	0.003153643
39.5075	916671.8344	0.003073696
39.5115	915012.7374	0.003068133
39.51533333	935306.466	0.00313618
39.51933333	961415.9416	0.003223727
39.52316667	975624.4116	0.00327137
39.52716667	982449.303	0.003294254
39.531	999439.2393	0.003351223
39.535	978545.1376	0.003281163
39.53883333	965675.1308	0.003238009
39.54283333	963799.2625	0.003231719
39.54666667	946436.9315	0.003173501
39.5505	949969.2097	0.003185345
39.5545	941864.9706	0.003158171
39.55833333	923808.4671	0.003097626
39.56233333	953535.0572	0.003197302
39.56616667	989599.74	0.003318231
39.57016667	939178.2924	0.003149162
39.574	924142.7471	0.003098746
39.578	901131.2676	0.003021587
39.58183333	882873.2848	0.002960366
39.58583333	874515.4367	0.002932341
39.58966667	874565.1005	0.002932507
39.5935	900448.8855	0.003019299
39.5975	901712.0337	0.003023534
39.60133333	887686.8521	0.002976506
39.60533333	874068.1359	0.002930841
39.60916667	873928.0839	0.002930372
39.61316667	880554.8677	0.002952592
39.617	857080.9002	0.002873881
39.621	856095.0625	0.002870576
39.62483333	866048.8812	0.002903952
39.62866667	882528.4314	0.002959209
39.63266667	888732.742	0.002980013

Table 3. Samples Results STD

Retention time (minutes)	Indensity	Percentage of maximum peak area
39.6365	886617.595	0.002972921
39.6405	886655.7539	0.002973049
39.64433333	884307.8133	0.002965176
39.64833333	905250.9158	0.0030354
39.65216667	914705.8052	0.003067103
39.65616667	929967.4954	0.003118277
39.66	932922.7624	0.003128187
39.664	932785.2668	0.003127726
39.66783333	905486.4551	0.00303619
39.67183333	900805.1121	0.003020493
39.67566667	893028.1139	0.002994416
39.6795	881913.2211	0.002957146
39.6835	882905.4162	0.002960473
39.68733333	885716.4634	0.002969899
39.69133333	877115.2491	0.002941058
39.69516667	874043.341	0.002930758
39.69916667	850153.6402	0.002850653
39.703	878755.6878	0.002946559
39.707	903124.4875	0.00302827
39.71083333	914267.8679	0.003065635
39.71466667	882943.1659	0.0029606
39.71866667	871232.4739	0.002921333
39.7225	902482.2164	0.003026116
39.7265	921832.439	0.003091
39.73033333	939514.8225	0.003150291
39.73433333	919435.1051	0.003082961
39.73816667	922876.832	0.003094502
39.74216667	933858.3038	0.003131324
39.746	914231.456	0.003065513
39.75	911422.1403	0.003056093
39.75383333	900469.6416	0.003019368
39.75766667	884685.9642	0.002966444
39.76166667	885387.8307	0.002968797
39.7655	889998.9532	0.002984259
39.7695	901133.5426	0.003021594
39.77333333	898011.226	0.003011125
39.77733333	899174.5174	0.003015025
39.78116667	906899.4169	0.003040928
39.785	930812.919	0.003121112
39.789	910498.2004	0.003052995
39.79283333	904618.5749	0.00303328
39.79683333	899872.9278	0.003017367
39.80066667	849225.9718	0.002847543
39.80466667	855905.8935	0.002869941
39.8085	882921.5596	0.002960528
39.8125	888128.8911	0.002977988
39.81633333	888447.0229	0.002979055
39.82033333	851614.2216	0.002855551
39.82416667	836512.5248	0.002804913
39.828	822760.5339	0.002758801
39.832	841488.1255	0.002821597
39.83583333	860792.7739	0.002886327
39.83983333	859592.9123	0.002882304
39.84366667	873979.8042	0.002930545
39.84766667	890705.3569	0.002986627
39.8515	897187.923	0.003008364
39.8555	896866.5426	0.003007287
39.85933333	873527.9568	0.00292903
39.86333333	865849.3094	0.002903283
39.86716667	884607.3802	0.00296618
39.871	902399.1829	0.003025838
39.875	926093.3927	0.003105287
39.87883333	931305.9849	0.003122766
39.88283333	906917.8349	0.00304099
39.88666667	854670.14	0.002865798
39.89066667	854356.2377	0.002864745
39.8945	845854.2645	0.002836237
39.8985	854270.0922	0.002864456
39.9025	841013.1806	0.002820004
39.90633333	840632.3196	0.002818727
39.91016667	863654.8543	0.002895924
39.91416667	877429.7301	0.002942113
39.918	870441.5653	0.002918681
39.922	866428.4908	0.002905225

Table 3. Samples Results STD

Retention time (minutes)	Indensity	Percentage of maximum peak area
39.92583333	849832.0652	0.002849575
39.92983333	849343.0144	0.002847935
39.93366667	848558.5694	0.002845305
39.9375	847580.8793	0.002842027
39.9415	844886.7617	0.002832993
39.94533333	849459.6964	0.002848326
39.94933333	846696.0396	0.00283906
39.95316667	856618.6674	0.002872331
39.95716667	872107.0659	0.002924265
39.961	890283.5679	0.002985213
39.965	862301.4876	0.002891386
39.96883333	866636.7794	0.002905923
39.97283333	868982.6454	0.002913789
39.97666667	862473.7965	0.002891964
39.9805	868520.1493	0.002912238
39.9845	874779.6983	0.002933227
39.98833333	852605.2621	0.002858874
39.99233333	840744.0676	0.002819102
39.99616667	862573.4978	0.002892298
40.00016667	873962.0655	0.002930485
40.004	903343.3812	0.003029004
40.008	888383.899	0.002978843
40.01183333	843766.3513	0.002829236
40.01583333	818744.2642	0.002745334
40.01966667	800758.1249	0.002685025
40.0235	775810.4064	0.002601373
40.0275	801544.9848	0.002687663
40.03133333	802382.1176	0.00269047
40.03533333	820425.0243	0.00275097
40.03916667	811927.1116	0.002722476
40.04316667	806703.1221	0.002704959
40.047	797803.9432	0.002675119
40.051	792633.1141	0.002657781
40.05483333	791168.2784	0.002652869
40.05883333	816553.2934	0.002737988
40.06266667	848685.2906	0.00284573
40.0665	872204.1631	0.002924591
40.0705	866854.9062	0.002906654
40.07433333	839419.3419	0.00281466
40.07833333	813316.9432	0.002727136
40.08216667	835922.0749	0.002802933
40.08616667	840176.2781	0.002817198
40.09	828224.2871	0.002777122
40.094	800983.1378	0.00268578
40.098	785327.502	0.002633285
40.102	798105.4126	0.00267613
40.10583333	825806.7309	0.002769016
40.10966667	829450.792	0.002781235
40.11366667	841411.9702	0.002821342
40.1175	840626.2391	0.002818707
40.1215	823143.9179	0.002760087
40.12533333	835481.8819	0.002801457
40.12933333	838298.6245	0.002810902
40.13316667	845378.9551	0.002834643
40.13716667	852378.1181	0.002858112
40.141	859421.5937	0.00288173
40.145	868223.0183	0.002911242
40.14883333	889455.9834	0.002982438
40.15266667	882522.3851	0.002959189
40.15666667	884967.9962	0.002967389
40.1605	862053.3536	0.002890554
40.1645	854981.1744	0.002866841
40.16833333	851236.0092	0.002854283
40.17233333	819222.6035	0.002746938
40.17616667	827366.5256	0.002774246
40.18	835830.6746	0.002802627
40.184	842193.2584	0.002823961
40.18783333	840023.6653	0.002816686
40.19183333	838527.9336	0.002811671
40.19566667	830571.4737	0.002784992
40.19966667	830497.8465	0.002784745
40.2035	815449.7303	0.002734288
40.2075	809001.0718	0.002712665
40.21133333	817016.4902	0.002739541

Table 3. Samples Results STD

Retention time (minutes)	Indensity	Percentage of maximum peak area
40.21533333	797249.1103	0.002673259
40.21916667	801350.3687	0.002687011
40.223	826498.4195	0.002771335
40.227	833178.0115	0.002793732
40.23083333	809490.627	0.002714306
40.23483333	805268.2037	0.002700148
40.23866667	807524.4394	0.002707713
40.24266667	805916.5965	0.002702322
40.2465	790949.9291	0.002652137
40.2505	778510.5025	0.002610426
40.25433333	788265.3009	0.002643135
40.25816667	793022.8819	0.002659088
40.26216667	793422.6033	0.002660428
40.266	824167.9034	0.00276352
40.27	834190.803	0.002797128
40.27383333	854313.6678	0.002864602
40.27783333	864720.2537	0.002899497
40.28166667	844496.9515	0.002831686
40.28566667	820730.1516	0.002751993
40.2895	827140.0423	0.002773486
40.2935	844469.2133	0.002831593
40.29733333	840832.576	0.002819399
40.30116667	844143.3168	0.0028305
40.30516667	838972.1762	0.002813161
40.309	825002.1489	0.002766318
40.313	815990.5539	0.002736101
40.31683333	817446.5816	0.002740983
40.32083333	821864.3141	0.002755796
40.32466667	815135.6798	0.002733234
40.32866667	789460.0906	0.002647142
40.3325	797003.7947	0.002672436
40.3365	791932.5347	0.002655432
40.34033333	802471.9243	0.002690772
40.34416667	832393.2012	0.002791101
40.34816667	839456.3561	0.002814784
40.352	850331.3927	0.002851249
40.356	834855.8973	0.002799358
40.35983333	824065.918	0.002763178
40.36383333	829617.6636	0.002781794
40.36766667	826954.8487	0.002772865
40.3715	801171.2533	0.00268641
40.3755	820995.6173	0.002752883
40.37933333	808387.0388	0.002710606
40.38333333	817319.0303	0.002740555
40.38716667	828501.5678	0.002778052
40.39116667	820481.1244	0.002751158
40.395	814547.0991	0.002731261
40.399	794382.7497	0.002663648
40.40283333	791244.5546	0.002653125
40.40683333	773843.6247	0.002594778
40.41066667	771580.6038	0.00258719
40.4145	802223.3472	0.002689938
40.4185	812567.725	0.002724624
40.42233333	816667.9704	0.002738372
40.42633333	810410.4968	0.00271739
40.43016667	812723.1175	0.002725145
40.43416667	800346.4697	0.002683645
40.438	790006.1983	0.002648973
40.442	793785.7825	0.002661646
40.44583333	755776.5985	0.002534197
40.44983333	750459.2021	0.002516368
40.45366667	773040.5877	0.002592085
40.4575	786334.1845	0.00263666
40.4615	791091.3643	0.002652611
40.46533333	778833.4537	0.002611509
40.46933333	781358.281	0.002619975
40.47316667	779608.5352	0.002614108
40.47716667	785154.1573	0.002632703
40.481	809778.1825	0.00271527
40.485	829933.4941	0.002782853
40.48883333	830793.3951	0.002785736
40.49266667	831568.3164	0.002788335
40.49666667	820547.3766	0.00275138
40.5005	809874.7166	0.002715594

Table 3. Samples Results STD

Retention time (minutes)	Indensity	Percentage of maximum peak area
40.5045	815116.676	0.002733171
40.50833333	795485.6063	0.002667346
40.51233333	773590.2544	0.002593928
40.51616667	759999.8616	0.002548358
40.52	771299.9451	0.002586249
40.524	778182.8058	0.002609328
40.52783333	775901.1911	0.002601677
40.53183333	772284.5392	0.00258955
40.53566667	766187.8911	0.002569107
40.53966667	766998.3118	0.002571825
40.5435	779922.9576	0.002615163
40.5475	761616.2152	0.002553778
40.55133333	775746.2597	0.002601158
40.55533333	796488.7347	0.002670709
40.55916667	793180.4285	0.002659616
40.563	815113.503	0.00273316
40.567	802530.7666	0.002690969
40.57083333	786791.6093	0.002638194
40.57483333	778026.8379	0.002608805
40.57866667	789757.2075	0.002648138
40.58266667	800362.2611	0.002683698
40.5865	797110.3675	0.002672794
40.5905	807032.2269	0.002706063
40.59433333	806835.1587	0.002705402
40.59816667	804860.7117	0.002698781
40.60216667	799275.3191	0.002680053
40.606	779594.7507	0.002614062
40.61	770193.9056	0.00258254
40.61383333	750690.442	0.002517143
40.61783333	746725.6545	0.002503849
40.62166667	742350.0086	0.002489177
40.62566667	768083.3445	0.002575463
40.6295	782795.3703	0.002624794
40.6335	799527.2615	0.002680898
40.63733333	804680.8015	0.002698178
40.64116667	814956.5595	0.002732634
40.64516667	820614.3325	0.002751605
40.649	794754.8031	0.002664895
40.653	802415.047	0.002690581
40.65683333	809529.0887	0.002714435
40.66083333	818774.2524	0.002745435
40.66466667	833951.5397	0.002796326
40.66866667	825955.4863	0.002769514
40.6725	817977.322	0.002742763
40.6765	825019.6743	0.002766377
40.68033333	823270.2109	0.00276051
40.68416667	811287.4422	0.002720331
40.68816667	790667.9865	0.002651192
40.692	767818.247	0.002574574
40.696	753769.8371	0.002527468
40.69983333	751378.0468	0.002519449
40.70383333	778780.8039	0.002611333
40.70766667	800778.373	0.002685093
40.71166667	799884.9429	0.002682097
40.7155	773036.2849	0.002592071
40.7195	779504.7757	0.00261376
40.72333333	767791.1695	0.002574483
40.72716667	765516.5852	0.002566857
40.73116667	782353.9442	0.002623314
40.735	782169.4684	0.002622695
40.739	776784.9541	0.002604641
40.74283333	773722.412	0.002594371
40.74683333	793458.8381	0.00266055
40.75066667	780761.3958	0.002617974
40.75466667	774357.4425	0.002596501
40.7585	786522.7549	0.002637292
40.7625	786768.4529	0.002638116
40.76633333	781813.9457	0.002621503
40.77016667	780386.9444	0.002616718
40.77416667	762887.798	0.002558042
40.778	753475.7056	0.002526482
40.782	768746.0716	0.002577685
40.78583333	760017.8116	0.002548419
40.78983333	775854.0717	0.002601519

Table 3. Samples Results STD

Retention time (minutes)	Indensity	Percentage of maximum peak area
40.79366667	778647.4067	0.002610886
40.7975	784796.2477	0.002631503
40.8015	778904.2029	0.002611747
40.80533333	768640.449	0.002577331
40.80933333	773481.6698	0.002593564
40.81316667	764464.2137	0.002563328
40.81716667	755136.075	0.00253205
40.821	797491.5904	0.002674072
40.825	821107.2371	0.002753258
40.82883333	785528.3004	0.002633958
40.83266667	782120.386	0.002622531
40.83666667	767383.7622	0.002573117
40.8405	750107.6573	0.002515189
40.8445	739757.8028	0.002480485
40.84833333	744205.0042	0.002495397
40.85233333	742507.7819	0.002489706
40.85616667	743249.1362	0.002492191
40.86016667	745460.1525	0.002499605
40.864	762136.2664	0.002555522
40.868	777608.5037	0.002607402
40.87183333	807533.9748	0.002707745
40.87566667	784071.8773	0.002629074
40.87966667	754466.4887	0.002529804
40.8835	746816.8917	0.002504154
40.8875	753439.0371	0.002526359
40.89133333	752581.1519	0.002523483
40.89533333	746433.514	0.002502869
40.89916667	768197.9092	0.002575847
40.90316667	759556.0249	0.00254687
40.907	763144.9902	0.002558904
40.911	762998.0099	0.002558411
40.91483333	776542.8128	0.002603829
40.91866667	758120.3335	0.002542056
40.92266667	754111.9132	0.002528615
40.9265	735674.7106	0.002466794
40.9305	737822.68	0.002473996
40.93433333	720323.8682	0.002415321
40.93833333	725183.2841	0.002431615
40.94216667	727694.9477	0.002440037
40.94616667	739782.7332	0.002480568
40.95	749706.5388	0.002513844
40.954	753788.1614	0.00252753
40.95783333	771790.9362	0.002587895
40.96166667	790274.4595	0.002649872
40.96566667	811922.2638	0.00272246
40.9695	812178.1144	0.002723317
40.9735	801301.2314	0.002686846
40.97733333	758832.6321	0.002544445
40.98133333	751896.4826	0.002521187
40.98516667	743769.5651	0.002493936
40.98916667	747769.4746	0.002507349
40.993	748444.845	0.002509613
40.997	768281.3652	0.002576127
41.00083333	769638.9885	0.002580679
41.00466667	765997.7564	0.00256847
41.00866667	786736.4686	0.002638009
41.0125	792511.478	0.002657373
41.0165	779602.3935	0.002614088
41.02033333	790296.3323	0.002649946
41.02433333	792073.7947	0.002655906
41.02816667	779079.731	0.002612335
41.032	764588.3602	0.002563744
41.036	753617.4721	0.002526958
41.03983333	770710.5476	0.002584272
41.04383333	753263.3867	0.00252577
41.04766667	769515.9547	0.002580267
41.05166667	766611.9928	0.00257053
41.0555	763692.4545	0.00256074
41.0595	768786.8849	0.002577822
41.06333333	786066.0373	0.002635761
41.06733333	796276.3753	0.002669997
41.07116667	783278.3025	0.002626413
41.075	780845.1716	0.002618255
41.079	765769.5505	0.002567705

Table 3. Samples Results STD

Retention time (minutes)	Indensity	Percentage of maximum peak area
41.08283333	761185.6535	0.002552334
41.08683333	760385.6692	0.002549652
41.09066667	764220.2362	0.00256251
41.09466667	761458.9035	0.002553251
41.0985	764539.9576	0.002563582
41.1025	754451.6712	0.002529755
41.10633333	740594.427	0.00248329
41.11033333	757629.3664	0.00254041
41.11416667	747146.4738	0.00250526
41.118	755720.3064	0.002534009
41.122	757211.0615	0.002539007
41.12583333	773817.4012	0.00259469
41.12983333	765934.4919	0.002568258
41.13366667	771172.405	0.002585821
41.13766667	807767.2943	0.002708528
41.1415	808909.2069	0.002712356
41.1455	801090.3845	0.002686139
41.14933333	786413.7534	0.002636927
41.15333333	782271.6304	0.002623038
41.15716667	760196.5572	0.002549018
41.161	745793.651	0.002500723
41.165	741996.3217	0.002487991
41.16883333	750249.2525	0.002515664
41.17283333	747146.2256	0.002505259
41.17666667	753837.8036	0.002527696
41.18066667	762651.0114	0.002557248
41.1845	755236.8522	0.002532387
41.1885	756090.2674	0.002535249
41.19233333	754501.0005	0.00252992
41.19616667	767627.1569	0.002573933
41.20016667	787298.6166	0.002639894
41.204	781321.8078	0.002619853
41.208	784901.5661	0.002631856
41.21183333	774282.4802	0.002596249
41.21583333	753547.5167	0.002526723
41.21966667	749568.5787	0.002513381
41.22366667	750568.1275	0.002516733
41.2275	748934.0495	0.002511254
41.2315	737230.3011	0.00247201
41.23533333	759824.8776	0.002547772
41.23916667	778799.9491	0.002611397
41.24316667	773155.4148	0.00259247
41.247	773348.5161	0.002593118
41.251	776345.0374	0.002603165
41.25483333	799983.1996	0.002682427
41.25883333	771444.4358	0.002586733
41.26266667	760338.613	0.002549494
41.26666667	743383.1052	0.002492641
41.2705	757150.298	0.002538803
41.2745	752037.2994	0.002521659
41.27833333	745316.6457	0.002499124
41.28216667	782392.4035	0.002623443
41.28616667	787705.6864	0.002641259
41.29	780790.3248	0.002618071
41.294	770775.217	0.002584489
41.29783333	770066.5811	0.002582113
41.30183333	758811.6368	0.002544374
41.30566667	754265.8047	0.002529131
41.3095	746440.1134	0.002502891
41.3135	738138.4655	0.002475055
41.31733333	760825.0215	0.002551125
41.32133333	783191.3924	0.002626122
41.32516667	772785.3393	0.002591229
41.32916667	746698.4473	0.002503757
41.333	719180.4796	0.002411487
41.337	717458.1117	0.002405711
41.34083333	719768.534	0.002413459
41.34483333	713713.2879	0.002393155
41.34866667	713070.5842	0.002391
41.3525	712779.5538	0.002390024
41.3565	710585.904	0.002382668
41.36033333	718377.7353	0.002408795
41.36433333	711456.5608	0.002385588
41.36816667	715351.0646	0.002398646

Table 3. Samples Results STD

Retention time (minutes)	Indensity	Percentage of maximum peak area
41.37216667	721999.8804	0.00242094
41.376	729201.5739	0.002445089
41.38	729726.3086	0.002446848
41.38383333	733462.8317	0.002459377
41.38766667	747863.7485	0.002507665
41.39166667	764531.1718	0.002563552
41.3955	762394.364	0.002556387
41.3995	757775.5407	0.0025409
41.40333333	730619.529	0.002449843
41.40733333	742053.6853	0.002488183
41.41116667	722502.9083	0.002422627
41.41516667	728325.0853	0.00244215
41.419	726247.3744	0.002435183
41.423	736499.2746	0.002469558
41.427	728035.6984	0.002441179
41.43083333	737527.5191	0.002473006
41.43483333	748906.567	0.002511161
41.43866667	765926.4639	0.002568231
41.4425	769046.4802	0.002578693
41.4465	778701.2882	0.002611066
41.45033333	770327.0246	0.002582986
41.45433333	743533.2992	0.002493144
41.45816667	743228.8305	0.002492123
41.46216667	724119.4921	0.002428048
41.466	732366.5414	0.002455701
41.47	730009.8829	0.002447799
41.47383333	737395.8834	0.002472565
41.47766667	730923.38	0.002450862
41.48166667	736957.6597	0.002471095
41.4855	741578.2996	0.002486589
41.4895	739469.2585	0.002479517
41.49333333	735080.6052	0.002464802
41.49733333	731145.1566	0.002451606
41.50116667	727521.2438	0.002439454
41.50516667	739320.7154	0.002479019
41.509	748256.3172	0.002508981
41.513	763342.0333	0.002559565
41.51683333	770779.8568	0.002584505
41.52066667	771654.8692	0.002587439
41.52466667	746816.2256	0.002504152
41.5285	750161.3859	0.002515369
41.5325	738017.8928	0.002474651
41.53633333	733519.7679	0.002459568
41.54033333	731355.9678	0.002452312
41.54416667	754472.2862	0.002529824
41.54816667	742503.1676	0.00248969
41.552	738090.7731	0.002474895
41.556	730134.7299	0.002448217
41.55983333	718392.5425	0.002408845
41.56366667	717919.6106	0.002407259
41.56766667	732973.7437	0.002457737
41.5715	721255.4935	0.002418444
41.5755	720707.5066	0.002416607
41.57933333	766455.2878	0.002570004
41.58333333	768174.7477	0.00257577
41.58716667	736937.493	0.002471028
41.591	763766.1213	0.002560987
41.595	751989.3385	0.002521498
41.59883333	741109.3158	0.002485016
41.60283333	712888.8515	0.00239039
41.60666667	705515.7922	0.002365668
41.61066667	711149.2565	0.002384557
41.6145	744881.6499	0.002497665
41.6185	750634.6248	0.002516956
41.62233333	747965.9987	0.002508008
41.62616667	735199.9988	0.002465202
41.63016667	754648.9526	0.002530416
41.634	761272.1432	0.002552624
41.638	757546.0382	0.00254013
41.64183333	752536.263	0.002523332
41.64583333	736795.0207	0.00247055
41.64966667	725004.3152	0.002431015
41.65366667	736354.0741	0.002469072
41.6575	735289.6116	0.002465502

Table 3. Samples Results STD

Retention time (minutes)	Indensity	Percentage of maximum peak area
41.6615	744821.4745	0.002497464
41.66533333	763109.857	0.002558786
41.66916667	755073.6604	0.00253184
41.67316667	740144.9339	0.002481783
41.677	737527.5896	0.002473007
41.681	731885.1005	0.002454087
41.68483333	746447.4362	0.002502916
41.68883333	744802.2262	0.002497399
41.69266667	758769.17	0.002544232
41.69666667	763637.3957	0.002560555
41.7005	754260.6132	0.002529114
41.7045	762627.3201	0.002557169
41.70833333	732428.0288	0.002455907
41.71216667	702058.7984	0.002354076
41.71616667	693486.6263	0.002325333
41.72	689612.9022	0.002312344
41.724	700029.6119	0.002347272
41.72783333	706090.413	0.002367594
41.73183333	713875.2536	0.002393698
41.73566667	705761.2002	0.002366491
41.73966667	708171.074	0.002374571
41.7435	703106.6411	0.002357589
41.7475	723013.7607	0.00242434
41.75133333	757367.7404	0.002539533
41.75516667	771958.9475	0.002588458
41.75916667	771007.1901	0.002585267
41.763	751516.3479	0.002519912
41.767	726113.1328	0.002434733
41.77083333	720506.1912	0.002415932
41.77483333	725387.8428	0.002432301
41.77866667	732795.0443	0.002457138
41.7825	740565.9278	0.002483194
41.7865	747133.2061	0.002505215
41.79033333	737992.6649	0.002474566
41.79433333	730720.502	0.002450182
41.79816667	720356.4345	0.00241543
41.80216667	722520.119	0.002422685
41.806	717788.164	0.002406818
41.81	717917.5459	0.002407252
41.81383333	743649.8099	0.002493535
41.81766667	770527.5653	0.002583659
41.82166667	784362.9767	0.00263005
41.8255	775849.7013	0.002601505
41.8295	753171.6466	0.002525463
41.83333333	744636.8662	0.002496845
41.83733333	743804.5119	0.002494054
41.84116667	728634.414	0.002443187
41.84516667	719286.5418	0.002411842
41.849	716214.0416	0.00240154
41.853	727566.285	0.002439605
41.85683333	739858.734	0.002480823
41.86066667	737665.8441	0.00247347
41.86466667	731861.1056	0.002454006
41.8685	718276.2704	0.002408455
41.8725	713323.8813	0.002391849
41.87633333	725656.3434	0.002433201
41.88033333	725437.8806	0.002432468
41.88416667	726878.2452	0.002437298
41.88816667	732596.5774	0.002456472
41.892	746942.593	0.002504576
41.896	758027.5903	0.002541745
41.89983333	764252.202	0.002562617
41.90366667	743686.1919	0.002493657
41.90766667	710319.3408	0.002381774
41.9115	697358.1509	0.002338314
41.9155	689981.8362	0.002313581
41.91933333	694183.5443	0.002327669
41.92333333	696599.9284	0.002335772
41.92716667	717319.6934	0.002405247
41.931	718116.9403	0.002407921
41.935	707568.0249	0.002372549
41.93883333	689714.8468	0.002312685
41.94283333	677320.7679	0.002271127
41.94666667	678577.8078	0.002275342

Table 3. Samples Results STD

Retention time (minutes)	Indensity	Percentage of maximum peak area
41.95066667	691046.505	0.002317151
41.9545	704951.2375	0.002363775
41.9585	718535.3349	0.002409324
41.96233333	714881.9923	0.002397073
41.96633333	696851.7173	0.002336616
41.97016667	702192.6547	0.002354525
41.974	697226.1094	0.002337871
41.978	707710.2523	0.002373026
41.98183333	703196.956	0.002357892
41.98583333	727612.8593	0.002439761
41.98966667	732769.3036	0.002457051
41.99366667	720798.1583	0.002416911
41.9975	706917.5036	0.002370368
42.0015	705491.9351	0.002365588
42.00533333	724437.2601	0.002429113
42.00933333	742885.8005	0.002490973
42.01316667	745005.8689	0.002498082
42.017	762412.4555	0.002556448
42.021	747428.4136	0.002506205
42.02483333	733968.8499	0.002461074
42.02883333	732636.5549	0.002456606
42.03266667	738981.032	0.00247788
42.03666667	758850.7843	0.002544505
42.0405	749341.4962	0.00251262
42.0445	738181.8909	0.0024752
42.04833333	718652.6182	0.002409717
42.05233333	723185.2605	0.002424915
42.05616667	715577.7189	0.002399406
42.06	706165.317	0.002367846
42.064	694231.2765	0.002327829
42.06783333	684896.1791	0.002296528
42.07183333	683297.7758	0.002291168
42.07566667	695782.5584	0.002333031
42.07966667	724980.4613	0.002430935
42.0835	746393.0123	0.002502733
42.0875	767973.595	0.002575095
42.09133333	767372.1082	0.002573078
42.09516667	757635.4083	0.00254043
42.09916667	776143.8551	0.002602491
42.103	781512.4921	0.002620492
42.107	768543.893	0.002577007
42.11083333	762067.1914	0.00255529
42.11483333	758006.4855	0.002541674
42.11866667	749426.8971	0.002512906
42.1225	734449.5294	0.002462685
42.1265	741546.6222	0.002486483
42.13033333	736305.6032	0.002468909
42.13433333	750945.9581	0.002518
42.13816667	750350.2439	0.002516002
42.14216667	747048.3433	0.002504931
42.146	774003.4509	0.002595314
42.15	786008.8003	0.002635569
42.15383333	789210.4474	0.002646304
42.15783333	783026.9538	0.002625571
42.16166667	786146.0343	0.002636029
42.1655	789365.7242	0.002646825
42.1695	785006.9984	0.00263221
42.17333333	766244.8363	0.002569298
42.17733333	752903.5662	0.002524564
42.18116667	758013.6977	0.002541699
42.18516667	761192.1231	0.002552356
42.189	791306.2539	0.002653332
42.193	797151.7159	0.002672932
42.19683333	824117.1494	0.00276335
42.20083333	812392.7993	0.002724037
42.20466667	789854.4537	0.002648464
42.2085	777594.6297	0.002607355
42.2125	777122.2441	0.002605771
42.21633333	820506.6388	0.002751244
42.22033333	841369.0252	0.002821198
42.22416667	841204.9724	0.002820648
42.22816667	845286.7972	0.002834334
42.232	820257.5004	0.002750408
42.236	818477.4888	0.00274444

Table 3. Samples Results STD

Retention time (minutes)	Indensity	Percentage of maximum peak area
42.23983333	807863.8666	0.002708851
42.24383333	796647.6416	0.002671242
42.24766667	779803.337	0.002614761
42.2515	791852.3181	0.002655163
42.2555	827579.2764	0.002774959
42.25933333	820182.9074	0.002750158
42.26333333	818609.207	0.002744882
42.26716667	813656.9936	0.002728276
42.27116667	830100.5993	0.002783413
42.275	807956.617	0.002709162
42.279	827229.3891	0.002773786
42.28283333	827656.9288	0.00277522
42.28683333	827871.7359	0.00277594
42.29066667	834734.8654	0.002798953
42.2945	852646.8946	0.002859013
42.2985	864442.9688	0.002898567
42.30233333	857680.4766	0.002875892
42.30633333	831873.2363	0.002789357
42.31016667	837691.5663	0.002808867
42.31416667	845212.1577	0.002834084
42.318	838887.5546	0.002812877
42.322	845766.7184	0.002835944
42.32583333	842709.3616	0.002825692
42.32966667	840002.5004	0.002816616
42.33366667	848407.8017	0.002844799
42.3375	827670.2813	0.002775264
42.3415	808582.8414	0.002711262
42.34533333	793307.8552	0.002660044
42.34933333	809363.0552	0.002713878
42.35316667	785902.7058	0.002635213
42.35716667	792229.6955	0.002656428
42.361	806452.6505	0.002704119
42.365	805530.2303	0.002701026
42.36883333	797695.2583	0.002674755
42.37266667	807697.4455	0.002708293
42.37666667	817214.4296	0.002740205
42.3805	804461.1714	0.002697442
42.3845	814617.504	0.002731497
42.38833333	822436.3977	0.002757715
42.39233333	834528.96	0.002798262
42.39616667	838925.1572	0.002813003
42.4	827142.1017	0.002773493
42.404	819754.8695	0.002748723
42.40783333	816719.9441	0.002738547
42.41183333	809107.7028	0.002713022
42.41566667	839450.3159	0.002814764
42.41966667	821562.2194	0.002754783
42.4235	810565.9856	0.002717912
42.4275	801575.4224	0.002687766
42.43133333	797812.8545	0.002675149
42.43533333	798220.9041	0.002676517
42.43916667	832900.8573	0.002792803
42.443	824309.5103	0.002763995
42.447	817579.0333	0.002741427
42.45083333	808113.9314	0.00270969
42.45483333	807381.7871	0.002707235
42.45866667	794233.97	0.002663149
42.46266667	774807.8949	0.002598011
42.4665	769070.6101	0.002578774
42.4705	783698.1671	0.002627821
42.47433333	781709.1289	0.002621152
42.47833333	791691.1769	0.002654623
42.48216667	813285.9636	0.002727032
42.486	805895.4534	0.002702251
42.49	806395.4142	0.002703927
42.49383333	779092.27	0.002612377
42.49783333	758445.9388	0.002543148
42.50166667	770142.2046	0.002582367
42.50566667	772661.4093	0.002590814
42.5095	776160.2527	0.002602546
42.5135	805624.5202	0.002701343
42.51733333	802891.1193	0.002692177
42.52133333	801905.832	0.002688873
42.52516667	781797.2307	0.002621447

Table 3. Samples Results STD

Retention time (minutes)	Indensity	Percentage of maximum peak area
42.529	768402.247	0.002576532
42.533	770816.8827	0.002584629
42.53683333	791251.4714	0.002653148
42.54083333	795697.082	0.002668055
42.54466667	779048.2546	0.00261223
42.54866667	780318.0611	0.002616487
42.5525	763348.7047	0.002559587
42.5565	739095.1899	0.002478263
42.56033333	720925.6666	0.002417339
42.56416667	712050.1592	0.002387578
42.56816667	710440.9455	0.002382182
42.572	698272.5722	0.00234138
42.576	723677.7871	0.002426567
42.57983333	762649.5251	0.002557243
42.58383333	795505.1746	0.002667411
42.58766667	823351.6339	0.002760783
42.5915	821249.2955	0.002753734
42.5955	816837.4397	0.002738941
42.59933333	773629.0425	0.002594058
42.60333333	740012.5265	0.002481339
42.60716667	723661.2403	0.002426511
42.61116667	735523.0751	0.002466285
42.615	765391.2792	0.002566436
42.619	760314.187	0.002549412
42.62283333	771876.2109	0.002588181
42.62683333	761181.997	0.002552322
42.63066667	767148.2108	0.002572328
42.6345	771186.4259	0.002585868
42.6385	772172.0338	0.002589173
42.64233333	774932.1377	0.002598428
42.64633333	784703.3502	0.002631192
42.65016667	805801.911	0.002701937
42.65416667	798565.7923	0.002677674
42.658	794057.671	0.002662558
42.662	760018.1736	0.00254842
42.66583333	752056.5196	0.002521724
42.66966667	739530.557	0.002479723
42.67366667	720380.7793	0.002415511
42.6775	708518.6857	0.002375737
42.6815	701892.6548	0.002353519
42.68533333	710699.7564	0.00238305
42.68933333	734710.2826	0.00246356
42.69316667	753041.0224	0.002525025
42.69716667	751583.7247	0.002520138
42.701	767614.3291	0.00257389
42.705	742021.9789	0.002488077
42.70883333	747474.7378	0.00250636
42.71266667	750220.5171	0.002515567
42.71666667	752778.4139	0.002524144
42.7205	736707.373	0.002470256
42.7245	713130.2387	0.0023912
42.7285	713454.0987	0.002392286
42.73233333	710992.053	0.00238403
42.73633333	697746.715	0.002339617
42.74016667	726317.4111	0.002435418
42.74416667	727056.0763	0.002437894
42.748	738792.615	0.002477248
42.752	743390.8835	0.002492667
42.75583333	757833.0608	0.002541093
42.75966667	762360.7848	0.002556275
42.76366667	744757.0928	0.002497248
42.7675	737146.8658	0.00247173
42.7715	747142.7024	0.002505247
42.77533333	734310.5701	0.00246222
42.77933333	729883.9919	0.002447377
42.78316667	748278.3317	0.002509055
42.78716667	737382.4012	0.00247252
42.791	746010.0821	0.002501449
42.795	746170.0193	0.002501985
42.79883333	762233.776	0.002555849
42.80266667	773768.3483	0.002594526
42.80666667	757467.8931	0.002539868
42.8105	723748.1645	0.002426803
42.8145	710934.8007	0.002383838

Table 3. Samples Results STD

Retention time (minutes)	Indensity	Percentage of maximum peak area
42.81833333	723018.3413	0.002424355
42.82233333	716978.6582	0.002404104
42.82616667	717446.7906	0.002405674
42.83	706347.8422	0.002368458
42.834	700955.8738	0.002350378
42.83783333	700294.8996	0.002348161
42.84183333	714060.9373	0.00239432
42.84566667	726691.8443	0.002436673
42.84966667	737015.8308	0.002471291
42.8535	730834.205	0.002450563
42.8575	726054.927	0.002434537
42.86133333	735126.8546	0.002464957
42.86533333	704644.4926	0.002362746
42.86916667	721905.8784	0.002420625
42.873	728186.8822	0.002441686
42.877	746385.0637	0.002502707
42.88083333	717024.5149	0.002404258
42.88483333	722815.8706	0.002423677
42.88866667	706256.8435	0.002368152
42.89266667	689085.9308	0.002310577
42.8965	670842.0879	0.002249403
42.9005	681843.2681	0.002286291
42.90433333	686416.5743	0.002301626
42.90833333	693912.5241	0.002326761
42.91216667	702205.236	0.002354567
42.91616667	711782.5431	0.002386681
42.92	710365.5945	0.00238193
42.924	719692.0068	0.002413202
42.92783333	725418.1817	0.002432402
42.93183333	733909.5252	0.002460875
42.93566667	742826.6347	0.002490775
42.9395	745245.2928	0.002498885
42.9435	720373.1851	0.002415486
42.94733333	726162.2859	0.002434897
42.95133333	712815.106	0.002390143
42.95516667	711997.0798	0.0023874
42.95916667	734266.7412	0.002462073
42.963	716016.1256	0.002400876
42.967	691565.4713	0.002318891
42.97083333	686037.5492	0.002300355
42.97466667	676625.7204	0.002268796
42.97866667	684068.6905	0.002293753
42.9825	697089.0872	0.002337412
42.9865	690208.9025	0.002314342
42.99033333	690954.2566	0.002316841
42.99433333	697016.9657	0.00233717
42.99816667	706130.3232	0.002367728
43.00216667	710886.4802	0.002383676
43.006	716719.8479	0.002403236
43.01	711601.0389	0.002386072
43.01383333	709435.8563	0.002378812
43.01766667	722233.4104	0.002421724
43.02166667	732123.9973	0.002454888
43.0255	722278.0503	0.002421873
43.0295	717598.455	0.002406182
43.03333333	724047.6384	0.002427807
43.03733333	737129.394	0.002471671
43.04116667	731270.548	0.002452026
43.04516667	716514.606	0.002402548
43.049	727235.9785	0.002438498
43.053	733731.5952	0.002460278
43.05683333	739968.638	0.002481192
43.06066667	726222.6122	0.0024351
43.06466667	728232.8356	0.00244184
43.0685	716124.4076	0.002401239
43.0725	718614.1124	0.002409588
43.07633333	725785.2192	0.002433633
43.08033333	747510.0512	0.002506479
43.08416667	747718.7248	0.002507178
43.08816667	729398.9449	0.00244575
43.092	721544.0233	0.002419412
43.096	710740.551	0.002383187
43.09983333	712715.0308	0.002389807
43.10366667	721387.0048	0.002418885

Table 3. Samples Results STD

Retention time (minutes)	Indensity	Percentage of maximum peak area
43.10766667	719710.9925	0.002413266
43.1115	722430.5252	0.002422384
43.1155	718392.8932	0.002408846
43.11933333	710906.8849	0.002383745
43.12333333	715640.108	0.002399616
43.12716667	714554.1151	0.002395974
43.13116667	709174.2619	0.002377935
43.135	706014.6566	0.00236734
43.139	713468.2363	0.002392333
43.14283333	705584.0922	0.002365897
43.14683333	703415.2192	0.002358624
43.15066667	698022.0863	0.00234054
43.1545	682021.8729	0.00228689
43.1585	699873.0932	0.002346747
43.16233333	716942.2499	0.002403982
43.16633333	737612.5962	0.002473292
43.17016667	711369.0655	0.002385294
43.17416667	717291.5315	0.002405153
43.178	721635.2268	0.002419718
43.182	703726.5968	0.002359668
43.18583333	705617.414	0.002366008
43.18983333	683327.65	0.002291268
43.19366667	678816.3909	0.002276142
43.1975	676718.288	0.002269107
43.2015	679023.563	0.002276836
43.20533333	706984.219	0.002370591
43.20933333	706907.0138	0.002370333
43.21316667	719222.3681	0.002411627
43.21716667	713346.636	0.002391925
43.221	701436.685	0.00235199
43.225	693680.2213	0.002325982
43.22883333	693946.0233	0.002326873
43.23283333	703787.4773	0.002359872
43.23666667	714864.3092	0.002397014
43.2405	708511.6307	0.002375713
43.2445	711444.6915	0.002385548
43.24833333	717130.6473	0.002404613
43.25233333	717319.1064	0.002405245
43.25616667	708594.315	0.00237599
43.26016667	695175.464	0.002330995
43.264	698572.3338	0.002342385
43.268	703514.8221	0.002358958
43.27183333	742186.7347	0.002488629
43.27566667	714310.2217	0.002395156
43.27966667	714184.2882	0.002394734
43.2835	704065.7934	0.002360806
43.2875	691032.6652	0.002317104
43.29133333	684689.1954	0.002295834
43.29533333	686016.947	0.002300286
43.29916667	678672.9373	0.002275661
43.303	683694.1358	0.002292497
43.307	701167.137	0.002351086
43.31083333	681907.4198	0.002286506
43.31483333	691247.3638	0.002317824
43.31866667	700960.6951	0.002350394
43.32266667	735652.0966	0.002466718
43.3265	733544.7049	0.002459651
43.3305	740920.3689	0.002484383
43.33433333	710636.8907	0.002382839
43.33833333	692557.5536	0.002322217
43.34216667	696612.8246	0.002335815
43.346	697960.1636	0.002340333
43.35	718819.7629	0.002410277
43.35383333	728812.1997	0.002443783
43.35783333	736225.962	0.002468642
43.36166667	710561.6413	0.002382587
43.36566667	707170.1585	0.002371215
43.3695	705202.1559	0.002364616
43.3735	682927.1608	0.002289926
43.37733333	686033.313	0.002300341
43.38133333	697054.052	0.002337295
43.38516667	685808.1652	0.002299586
43.389	671655.5836	0.002252131
43.393	671921.8137	0.002253024

Table 3. Samples Results STD

Retention time (minutes)	Indensity	Percentage of maximum peak area
43.39683333	677989.5361	0.002273369
43.40083333	703498.9612	0.002358905
43.40466667	718029.5455	0.002407628
43.40866667	710541.6629	0.00238252
43.4125	708170.7537	0.00237457
43.4165	702200.331	0.002354551
43.42033333	701756.4322	0.002353062
43.42416667	692436.74	0.002321812
43.42816667	704358.7093	0.002361788
43.432	707150.3863	0.002371149
43.436	699713.3669	0.002346211
43.43983333	709240.3697	0.002378157
43.44383333	700782.7339	0.002349797
43.44766667	695294.1486	0.002331393
43.45166667	694931.0308	0.002330176
43.4555	677940.1411	0.002273204
43.4595	677256.3815	0.002270911
43.46333333	675976.9285	0.002266621
43.46716667	675643.8817	0.002265504
43.47116667	702796.6388	0.00235655
43.475	704117.5553	0.002360979
43.479	708118.1595	0.002374394
43.48283333	712564.6799	0.002389303
43.48683333	722341.9556	0.002422087
43.49066667	711944.5972	0.002387224
43.49466667	698768.716	0.002343044
43.4985	693655.1046	0.002325898
43.5025	723847.1373	0.002427135
43.50633333	754071.0997	0.002528479
43.51016667	711824.0773	0.00238682
43.51416667	715088.4121	0.002397766
43.518	709178.3831	0.002377949
43.522	703263.904	0.002358117
43.52583333	718438.424	0.002408999
43.52983333	729814.1316	0.002447142
43.53366667	728788.3027	0.002443703
43.5375	724891.3384	0.002430636
43.5415	713744.8513	0.002393261
43.54533333	688039.5074	0.002307068
43.54933333	680654.6411	0.002282306
43.55316667	680409.2284	0.002281483
43.55716667	706465.8805	0.002368853
43.561	719643.7743	0.00241304
43.565	717848.0535	0.002407019
43.56883333	698323.7234	0.002341552
43.57266667	681663.1886	0.002285687
43.57666667	709433.43	0.002378804
43.5805	720524.3981	0.002415993
43.5845	702731.9215	0.002356333
43.58833333	713979.741	0.002394048
43.59233333	737259.5895	0.002472108
43.59616667	743878.9967	0.002494303
43.60016667	724708.5859	0.002430023
43.604	709772.7774	0.002379942
43.608	697648.4846	0.002339288
43.61183333	716257.2537	0.002401685
43.61566667	705494.3621	0.002365596
43.61966667	715017.3007	0.002397527
43.6235	691755.0756	0.002319527
43.6275	685750.6405	0.002299393
43.63133333	687125.7763	0.002304004
43.63533333	689920.8171	0.002313376
43.63916667	683248.4357	0.002291003
43.64316667	679524.9462	0.002278518
43.647	714762.0795	0.002396671
43.651	698952.7905	0.002343661
43.65483333	674079.5561	0.002260259
43.65866667	661174.1862	0.002216986
43.66266667	660430.5698	0.002214492
43.6665	656636.8696	0.002201772
43.6705	679169.4638	0.002277326
43.67433333	703798.0419	0.002359908
43.67833333	715136.4189	0.002397927
43.68216667	730548.7592	0.002449606

Table 3. Samples Results STD

Retention time (minutes)	Indensity	Percentage of maximum peak area
43.68616667	730650.5475	0.002449947
43.69	726585.2308	0.002436316
43.694	707183.2983	0.002371259
43.69783333	687039.7704	0.002303716
43.70166667	669240.3619	0.002244032
43.70566667	681754.178	0.002285992
43.7095	697891.2762	0.002340102
43.7135	708740.025	0.002376479
43.71733333	698211.3469	0.002341175
43.72133333	681596.9493	0.002285465
43.72516667	671074.2027	0.002250181
43.729	677799.1427	0.002272731
43.733	691621.3098	0.002319078
43.73683333	689025.1558	0.002310373
43.74083333	695912.9283	0.002333468
43.74466667	684382.8826	0.002294807
43.74866667	681276.791	0.002284392
43.7525	677865.7475	0.002272954
43.7565	668019.2397	0.002239938
43.76033333	682757.6486	0.002289357
43.76433333	704224.1758	0.002361337
43.76816667	711744.0317	0.002386552
43.772	716402.1573	0.002402171
43.77616667	717639.709	0.00240632
43.78	725727.8586	0.002433441
43.784	728680.1462	0.00244334
43.78783333	739812.8977	0.002480669
43.79183333	731777.8646	0.002453727
43.79566667	703232.3064	0.002358011
43.7995	704391.7964	0.002361899
43.8035	711745.91	0.002386558
43.80733333	703476.1511	0.002358828
43.81133333	707559.2892	0.00237252
43.81516667	697780.4453	0.00233973
43.81916667	685400.1096	0.002298218
43.823	670995.5322	0.002249918
43.827	654576.7146	0.002194864
43.83083333	650600.385	0.002181531
43.83466667	653219.4333	0.002190313
43.83866667	663822.4438	0.002225866
43.8425	669791.3526	0.00224588
43.8465	680888.9095	0.002283091
43.85033333	686193.5513	0.002300878
43.85433333	684190.15	0.002294161
43.85816667	700745.6889	0.002349673
43.86216667	681245.583	0.002284287
43.86616667	682345.5696	0.002287975
43.87	691365.5428	0.00231822
43.874	690153.0814	0.002314155
43.87783333	668203.2591	0.002240555
43.88166667	661559.9093	0.002218279
43.88566667	658202.9011	0.002207023
43.8895	665667.5787	0.002232052
43.8935	700803.6005	0.002349867
43.89733333	715552.8894	0.002399323
43.90133333	721847.4286	0.002420429
43.90516667	699391.3271	0.002345132
43.90916667	700737.4351	0.002349645
43.913	697227.7393	0.002337877
43.917	686388.8504	0.002301533
43.92083333	689073.585	0.002310535
43.92466667	671149.0888	0.002250433
43.92866667	661651.658	0.002218587
43.9325	671666.6109	0.002252168
43.9365	669745.8842	0.002245727
43.94033333	660548.3747	0.002214887
43.94433333	654925.0719	0.002196032
43.94816667	655060.0653	0.002196484
43.95216667	670058.9606	0.002246777
43.956	663288.1979	0.002224074
43.96	660662.9584	0.002215271
43.96383333	660011.5071	0.002213087
43.96766667	681335.8577	0.00228459
43.97166667	696224.6568	0.002334513

Table 3. Samples Results STD

Retention time (minutes)	Indensity	Percentage of maximum peak area
43.9755	691630.5979	0.002319109
43.9795	693330.6492	0.00232481
43.98333333	692175.0675	0.002320935
43.98733333	667984.9688	0.002239823
43.99116667	666309.8491	0.002234206
43.995	678945.5974	0.002276575
43.999	698137.7251	0.002340928
44.00283333	703033.4317	0.002357344
44.00683333	692284.0179	0.0023213
44.01066667	679202.3051	0.002277436
44.01466667	678768.94	0.002275983
44.0185	676192.7005	0.002267344
44.0225	666673.9741	0.002235427
44.02633333	650671.7372	0.00218177
44.03033333	671773.9337	0.002252528
44.03416667	656946.1118	0.002202808
44.03816667	664128.3306	0.002226891
44.042	671475.6341	0.002251527
44.046	662367.403	0.002220987
44.04983333	684874.8306	0.002296456
44.05366667	692980.4064	0.002323635
44.05766667	703020.5797	0.002357301
44.0615	703549.1565	0.002359073
44.0655	721655.8214	0.002419787
44.06933333	698917.3804	0.002343542
44.07333333	678540.6086	0.002275217
44.07716667	643284.312	0.002156999
44.08116667	627336.2599	0.002103524
44.085	636651.1987	0.002134758
44.089	646991.121	0.002169428
44.09283333	666528.9869	0.002234941
44.09666667	666038.4455	0.002233296
44.10066667	657840.6013	0.002205808
44.1045	666783.852	0.002235795
44.1085	676337.5084	0.00226783
44.11233333	677327.2852	0.002271149
44.11633333	678617.5134	0.002275475
44.12016667	668062.1517	0.002240082
44.124	664955.4067	0.002229664
44.128	676338.3851	0.002267833
44.13183333	693177.2359	0.002324295
44.13583333	689761.8573	0.002312843
44.13966667	681625.1018	0.00228556
44.14366667	674552.5176	0.002261845
44.1475	662601.8833	0.002221773
44.1515	653671.2779	0.002191828
44.15533333	644250.0324	0.002160237
44.15933333	655043.375	0.002196428
44.16316667	650189.3232	0.002180152
44.167	652400.6671	0.002187567
44.171	647383.6331	0.002170744
44.17483333	660665.5328	0.00221528
44.17883333	685707.2738	0.002299248
44.18266667	712237.8892	0.002388208
44.18666667	706367.3658	0.002368523
44.1905	698856.1199	0.002343337
44.1945	680572.8377	0.002282031
44.19833333	660844.2389	0.002215879
44.20216667	658046.6156	0.002206499
44.20616667	671566.2382	0.002251831
44.21	669687.7532	0.002245532
44.214	690686.9743	0.002315945
44.21783333	699724.2401	0.002346248
44.22183333	703610.6576	0.00235928
44.22566667	693181.3293	0.002324309
44.22966667	680307.5486	0.002281142
44.2335	671561.3794	0.002251815
44.2375	666193.0826	0.002233815
44.24133333	659043.6576	0.002209842
44.24533333	648066.9055	0.002173036
44.24916667	642292.4639	0.002153673
44.253	649660.725	0.00217838
44.257	658759.1112	0.002208888
44.26083333	667782.1063	0.002239143

Table 3. Samples Results STD

Retention time (minutes)	Indensity	Percentage of maximum peak area
44.26483333	669830.6289	0.002246012
44.26866667	669743.7522	0.00224572
44.27266667	681102.2117	0.002283806
44.2765	691289.8709	0.002317967
44.2805	675410.7597	0.002264722
44.28433333	672978.7614	0.002256568
44.28833333	662240.1125	0.00222056
44.29216667	674648.4399	0.002262166
44.296	675072.9519	0.00226359
44.3	697667.5881	0.002339352
44.30383333	693911.8717	0.002326758
44.30783333	697790.4648	0.002339764
44.31166667	676129.6146	0.002267133
44.31566667	659042.281	0.002209837
44.3195	660388.659	0.002214352
44.3235	654975.1886	0.0021962
44.32733333	653022.8758	0.002189653
44.33116667	654994.9197	0.002196266
44.33516667	658988.614	0.002209657
44.339	670557.0566	0.002248447
44.343	672439.9242	0.002254761
44.34683333	674028.2368	0.002260087
44.35083333	658553.3237	0.002208198
44.35466667	670187.062	0.002247207
44.3585	672899.0356	0.0022563
44.3625	670799.9741	0.002249262
44.3665	657149.0144	0.002203489
44.37033333	660464.0218	0.002214604
44.37416667	677342.088	0.002271198
44.37816667	690287.2748	0.002314605
44.382	677509.0052	0.002271758
44.386	689738.5398	0.002312765
44.38983333	695316.2857	0.002331468
44.39383333	695327.7004	0.002331506
44.39766667	692094.2426	0.002320664
44.4015	678436.8533	0.002274869
44.4055	668146.1423	0.002240363
44.40933333	666340.2738	0.002234308
44.41333333	668868.308	0.002242785
44.41716667	666093.3296	0.00223348
44.42116667	686147.028	0.002300722
44.425	690624.8387	0.002315737
44.429	674875.5005	0.002262928
44.43283333	661410.4248	0.002217778
44.43683333	659529.8258	0.002211472
44.44066667	671164.2423	0.002250483
44.4445	679354.5565	0.002277946
44.4485	671216.5762	0.002250659
44.45233333	667114.3501	0.002236904
44.45633333	646212.7289	0.002166818
44.46016667	654990.0761	0.00219625
44.46416667	645749.5171	0.002165265
44.468	640190.7797	0.002146626
44.472	623443.0637	0.002090469
44.47583333	624012.0864	0.002092377
44.47966667	648926.9867	0.002175919
44.48366667	666788.1803	0.00223581
44.4875	666488.8709	0.002234806
44.4915	671661.6922	0.002252151
44.49533333	665438.229	0.002231283
44.49933333	668654.3549	0.002242067
44.50316667	670334.3311	0.002247701
44.50716667	679471.7103	0.002278339
44.511	674510.1268	0.002261702
44.515	674497.0516	0.002261659
44.51883333	674224.4856	0.002260745
44.52266667	659985.3508	0.002212999
44.52666667	657360.1841	0.002204197
44.5305	642972.9921	0.002155955
44.5345	655018.6947	0.002196346
44.53833333	678154.1906	0.002273921
44.54233333	677484.7355	0.002271677
44.54616667	683841.8846	0.002292993
44.55016667	701355.6288	0.002351718

Table 3. Samples Results STD

Retention time (minutes)	Indensity	Percentage of maximum peak area
44.554	677368.2516	0.002271286
44.558	665042.5315	0.002229957
44.56183333	664931.9933	0.002229586
44.56566667	665506.3766	0.002231512
44.56966667	689035.4544	0.002310407
44.5735	698726.5188	0.002342902
44.5775	702302.6492	0.002354894
44.58133333	694867.8579	0.002329964
44.58533333	674993.5698	0.002263323
44.58916667	675690.4859	0.00226566
44.593	681100.9922	0.002283802
44.597	664980.1849	0.002229748
44.60083333	672966.846	0.002256528
44.60483333	660217.0418	0.002213776
44.60866667	643981.7573	0.002159338
44.61266667	620163.0595	0.002079471
44.6165	615534.0917	0.00206395
44.6205	637317.1192	0.00213699
44.62433333	646814.541	0.002168836
44.62833333	650572.9999	0.002181439
44.63216667	655261.4044	0.002197159
44.636	647024.7481	0.002169541
44.64	644192.7535	0.002160045
44.64383333	654477.5745	0.002194531
44.64783333	656279.8357	0.002200574
44.65166667	649030.9371	0.002176268
44.65566667	663606.0256	0.00222514
44.6595	661537.4157	0.002218204
44.6635	663429.0808	0.002224547
44.66733333	637521.7034	0.002137676
44.67133333	630994.4227	0.00211579
44.67516667	637281.2577	0.00213687
44.679	643172.7047	0.002156625
44.683	658834.1824	0.002209139
44.68683333	675546.5341	0.002265178
44.69083333	679744.5459	0.002279254
44.69466667	688095.8918	0.002307257
44.69866667	665340.2788	0.002230955
44.7025	646216.7473	0.002166832
44.7065	633169.0625	0.002123082
44.71033333	673940.3356	0.002259792
44.71416667	669211.7825	0.002243937
44.71816667	687424.8205	0.002305007
44.722	679921.828	0.002279848
44.726	686587.0389	0.002302198
44.72983333	692068.8991	0.002320579
44.73383333	671157.3902	0.00225046
44.73766667	674557.2341	0.00226186
44.7415	675647.1803	0.002265515
44.7455	665847.8731	0.002232657
44.74933333	659403.2102	0.002211047
44.75333333	658506.7218	0.002208041
44.75716667	679740.2358	0.00227924
44.76116667	686311.6228	0.002301274
44.765	673081.5664	0.002256912
44.769	672242.6266	0.002254099
44.77283333	675114.7508	0.00226373
44.77683333	680087.286	0.002280403
44.78066667	676516.9507	0.002268432
44.7845	670312.8186	0.002247628
44.7885	681403.4885	0.002284817
44.79233333	688210.46	0.002307641
44.79633333	675412.1162	0.002264727
44.80016667	672003.8416	0.002253299
44.80416667	671168.8409	0.002250499
44.808	674010.8367	0.002260028
44.812	666373.1232	0.002234418
44.81583333	647787.4183	0.002172098
44.81966667	639925.1766	0.002145735
44.82366667	639868.0901	0.002145544
44.8275	639891.6366	0.002145623
44.8315	638339.8067	0.00214042
44.83533333	630646.0173	0.002114622
44.83933333	605304.2524	0.002029648

Table 3. Samples Results STD

Retention time (minutes)	Indensity	Percentage of maximum peak area
44.84316667	612490.8098	0.002053745
44.84716667	638278.9592	0.002140216
44.851	644495.5621	0.00216106
44.855	645660.5033	0.002164967
44.85883333	654736.7171	0.0021954
44.86266667	650608.6142	0.002181558
44.86666667	651714.6078	0.002185267
44.8705	632852.0068	0.002122018
44.8745	635408.7136	0.002130591
44.87833333	645229.2079	0.00216352
44.88233333	645721.4473	0.002165171
44.88616667	633041.7391	0.002122655
44.89016667	616140.0775	0.002065982
44.894	636056.8297	0.002132765
44.898	658484.1432	0.002207966
44.90183333	679449.7894	0.002278266
44.90566667	696604.8521	0.002335788
44.90966667	673637.6916	0.002258777
44.9135	657568.1024	0.002204894
44.9175	667831.6793	0.002239309
44.92133333	676899.4949	0.002269714
44.92533333	711143.5263	0.002384538
44.92916667	727443.8978	0.002439195
44.933	721685.8287	0.002419887
44.937	703631.3717	0.002359349
44.941	700403.3536	0.002348525
44.94483333	678570.6241	0.002275318
44.94866667	656944.5244	0.002202803
44.95266667	657258.9093	0.002203857
44.9565	650101.7003	0.002179858
44.9605	636810.4924	0.002135292
44.96433333	636906.3155	0.002135613
44.96833333	630708.0466	0.002114829
44.97216667	643309.2804	0.002157083
44.976	657554.8695	0.00220485
44.98	661451.1552	0.002217914
44.98383333	655122.4352	0.002196693
44.98783333	652855.1009	0.002189091
44.99166667	652357.6387	0.002187423
44.99566667	658292.6127	0.002207323
44.9995	649061.9175	0.002176372
45.0035	654105.5616	0.002193284
45.00733333	656865.5258	0.002202538
45.01116667	648582.81	0.002174765
45.01516667	635437.6364	0.002130688
45.019	636129.3503	0.002133008
45.023	636042.3431	0.002132716
45.02683333	626791.3134	0.002101696
45.03083333	625443.0157	0.002097175
45.03466667	629254.6576	0.002109956
45.03866667	631726.4514	0.002118244
45.0425	640423.734	0.002147407
45.0465	660441.7485	0.00221453
45.05033333	671728.3199	0.002252375
45.05433333	667778.1933	0.00223913
45.05816667	648353.0381	0.002173995
45.062	650010.0315	0.002179551
45.066	631855.1762	0.002118676
45.06983333	624560.6751	0.002094217
45.07383333	626585.5113	0.002101006
45.07766667	648277.8679	0.002173743
45.08166667	669952.9727	0.002246422
45.0855	687841.3349	0.002306403
45.0895	684501.3982	0.002295204
45.09333333	662853.4249	0.002222616
45.09716667	652502.524	0.002187909
45.10116667	648469.651	0.002174386
45.105	621893.6435	0.002085274
45.109	613345.1781	0.00205661
45.11283333	617787.3909	0.002071505
45.11683333	635055.5027	0.002129407
45.12066667	643307.0724	0.002157075
45.12466667	647491.6358	0.002171107
45.1285	640364.204	0.002147208

Table 3. Samples Results STD

Retention time (minutes)	Indensity	Percentage of maximum peak area
45.1325	619825.1263	0.002078338
45.13633333	595152.8072	0.001995609
45.14016667	577727.0479	0.001937179
45.14416667	607821.7384	0.002038089
45.148	636115.3424	0.002132961
45.152	644742.6153	0.002161889
45.15583333	637728.8669	0.002138371
45.15983333	650971.787	0.002182776
45.16383333	646011.8349	0.002166145
45.16766667	652793.9771	0.002188886
45.17166667	655715.28	0.002198681
45.1755	643576.6981	0.002157979
45.1795	636342.6214	0.002133723
45.18333333	620309.3905	0.002079962
45.18716667	654284.941	0.002193885
45.19116667	657158.0694	0.002203519
45.195	635879.6071	0.00213217
45.199	645189.3188	0.002163387
45.20283333	641172.1173	0.002149917
45.20683333	642850.0634	0.002155543
45.21066667	638861.7213	0.00214217
45.21466667	621649.5833	0.002084456
45.2185	626976.7991	0.002102318
45.2225	639544.4713	0.002144459
45.22633333	645452.5672	0.002164269
45.23016667	646772.8551	0.002168696
45.23416667	645501.3039	0.002164433
45.238	644405.687	0.002160759
45.242	656587.2308	0.002201605
45.24583333	668400.1901	0.002241215
45.24983333	663667.1233	0.002225345
45.25366667	655413.5934	0.00219767
45.25766667	667035.8362	0.00223664
45.2615	665716.2641	0.002232216
45.26533333	655930.4733	0.002199403
45.26933333	631065.7063	0.002116029
45.27316667	628992.7102	0.002109078
45.27716667	627407.352	0.002103762
45.281	613870.7326	0.002058372
45.285	623161.3064	0.002089524
45.28883333	629919.1463	0.002112184
45.29283333	621196.4994	0.002082936
45.29666667	618370.9004	0.002073462
45.3005	620820.1943	0.002081675
45.3045	627043.7384	0.002102543
45.30833333	644810.9838	0.002162118
45.31233333	657436.0159	0.002204451
45.31616667	657004.1566	0.002203003
45.32016667	643563.5121	0.002157935
45.324	634901.7186	0.002128891
45.328	645797.3554	0.002165426
45.33183333	664572.3182	0.00222838
45.33566667	639102.6016	0.002142977
45.33966667	619832.0772	0.002078361
45.3435	619469.9338	0.002077147
45.3475	619453.8735	0.002077093
45.35133333	622017.8198	0.00208569
45.35533333	615771.7803	0.002064747
45.35916667	640331.3314	0.002147097
45.36316667	661432.6236	0.002217852
45.367	661097.5352	0.002216729
45.371	649835.1989	0.002178965
45.37483333	624502.6483	0.002094022
45.37866667	632795.2914	0.002121828
45.38266667	639506.6306	0.002144332
45.3865	642298.3695	0.002153693
45.3905	627328.7322	0.002103498
45.39433333	613501.4773	0.002057134
45.39833333	621881.2608	0.002085232
45.40216667	644381.8252	0.002160679
45.40616667	649399.6139	0.002177504
45.41	666820.6078	0.002235919
45.414	659926.4621	0.002212802
45.41783333	641866.4223	0.002152245

Table 3. Samples Results STD

Retention time (minutes)	Indensity	Percentage of maximum peak area
45.42166667	656983.3409	0.002202933
45.42566667	655416.9338	0.002197681
45.4295	655949.7073	0.002199467
45.4335	638101.6546	0.002139621
45.43733333	628851.6204	0.002108605
45.44133333	625800.0335	0.002098372
45.44516667	640019.6599	0.002146052
45.449	629328.0638	0.002110202
45.453	636456.8481	0.002134106
45.45683333	645601.7627	0.00216477
45.46083333	657716.316	0.002205391
45.46466667	641427.1357	0.002150772
45.46866667	638147.0239	0.002139773
45.4725	629047.3352	0.002109261
45.4765	622397.2248	0.002086962
45.48033333	605382.242	0.002029909
45.48433333	595900.5055	0.001998116
45.48816667	592047.3995	0.001985196
45.492	590434.6042	0.001979788
45.496	601084.2503	0.002015498
45.49983333	615746.2745	0.002064661
45.50383333	629697.9593	0.002111443
45.50766667	629239.0005	0.002109904
45.51166667	633174.9827	0.002123101
45.5155	644934.9728	0.002162534
45.5195	651335.3976	0.002183995
45.52333333	661709.5877	0.002218781
45.52733333	653466.8824	0.002191142
45.53116667	640034.8571	0.002146103
45.535	635885.0034	0.002132188
45.539	631902.1561	0.002118833
45.54283333	630780.5355	0.002115073
45.54683333	613093.3758	0.002055766
45.55066667	617462.6111	0.002070416
45.55466667	622994.8589	0.002088966
45.5585	614080.3931	0.002059075
45.5625	632868.9922	0.002122075
45.56633333	647468.9642	0.002171031
45.57016667	658784.9545	0.002208974
45.57416667	655842.0303	0.002199106
45.578	660940.9083	0.002216203
45.582	649706.1236	0.002178532
45.58583333	633970.6761	0.002125769
45.58983333	631156.3154	0.002116333
45.59366667	651023.9454	0.002182951
45.59766667	659019.2139	0.00220976
45.6015	661717.2226	0.002218806
45.6055	654748.4151	0.002195439
45.60933333	658528.1174	0.002208113
45.61316667	646727.2962	0.002168544
45.61716667	647956.1755	0.002172664
45.621	635173.2497	0.002129802
45.625	633806.7334	0.00212522
45.62883333	639012.3045	0.002142675
45.63283333	638319.7736	0.002140352
45.63666667	642325.265	0.002153783
45.6405	652436.3303	0.002187687
45.6445	654253.3923	0.002193779
45.64833333	651185.3752	0.002183492
45.65233333	656031.8241	0.002199743
45.65616667	666792.8841	0.002235826
45.66016667	657000.6168	0.002202991
45.664	646959.9261	0.002169324
45.668	647512.2861	0.002171176
45.67183333	634819.5037	0.002128616
45.67583333	637941.3959	0.002139084
45.67966667	631978.6376	0.00211909
45.6835	620951.2147	0.002082114
45.6875	625649.123	0.002097866
45.69133333	622826.6556	0.002088402
45.69533333	619172.1248	0.002076148
45.69916667	623810.4123	0.002091701
45.70316667	629979.2683	0.002112386
45.707	645748.2357	0.002165261

Table 3. Samples Results STD

Retention time (minutes)	Indensity	Percentage of maximum peak area
45.711	655480.1259	0.002197893
45.71483333	668582.9721	0.002241828
45.71883333	661076.4692	0.002216658
45.72266667	658528.5761	0.002208115
45.7265	645514.5853	0.002164477
45.7305	646472.1372	0.002167688
45.73433333	631064.0037	0.002116023
45.73833333	636928.6058	0.002135688
45.74216667	648864.3365	0.002175709
45.74616667	674331.3021	0.002261103
45.75	673106.683	0.002256997
45.754	641083.5656	0.00214962
45.75783333	618942.5946	0.002075379
45.76166667	616484.0236	0.002067135
45.76566667	625716.3244	0.002098092
45.7695	635145.6848	0.002129709
45.7735	640603.6893	0.002148011
45.77733333	647116.7538	0.00216985
45.78133333	644788.9277	0.002162044
45.78516667	639396.5923	0.002143963
45.789	625356.5435	0.002096885
45.793	620668.0448	0.002081164
45.79683333	617640.8749	0.002071014
45.80083333	621592.0303	0.002084263
45.80466667	629612.6182	0.002111156
45.80866667	639822.8507	0.002145392
45.8125	648683.3467	0.002175103
45.8165	635230.4764	0.002129994
45.82033333	629994.5114	0.002112437
45.82433333	631702.4431	0.002118164
45.82816667	634747.583	0.002128374
45.832	642785.9882	0.002155328
45.836	649857.2608	0.002179039
45.83983333	672610.7257	0.002255334
45.84383333	683742.538	0.00229266
45.84766667	679626.5619	0.002278858
45.85166667	675853.3004	0.002266206
45.8555	665021.2324	0.002229885
45.8595	674241.0233	0.0022608
45.86333333	692242.1095	0.00232116
45.86716667	681316.8662	0.002284526
45.87116667	688011.4856	0.002306974
45.875	679161.2108	0.002277298
45.879	674495.1641	0.002261652
45.88283333	676155.7347	0.00226722
45.88683333	688061.6506	0.002307142
45.89066667	688884.8317	0.002309902
45.89466667	683423.8206	0.002291591
45.8985	682252.2236	0.002287662
45.9025	661608.0852	0.002218441
45.90633333	654243.6691	0.002193747
45.91016667	660911.4222	0.002216105
45.91416667	690073.0749	0.002313887
45.918	703740.1798	0.002359714
45.922	707683.3655	0.002372936
45.92583333	711436.8036	0.002385521
45.92983333	688933.6459	0.002310066
45.93366667	671672.8234	0.002252189
45.93766667	652028.3928	0.002186319
45.9415	651340.676	0.002184013
45.9455	643908.8382	0.002159093
45.94933333	659890.4028	0.002212681
45.95316667	667758.2583	0.002239063
45.95716667	676432.9674	0.00226815
45.961	670265.1836	0.002247469
45.965	659375.9137	0.002210956
45.96883333	659932.8827	0.002212823
45.97283333	642852.4861	0.002155551
45.97666667	641482.6481	0.002150958
45.9805	642371.9746	0.00215394
45.9845	642251.5531	0.002153536
45.98833333	640384.6413	0.002147276
45.99233333	631271.3658	0.002116718
45.99616667	618880.0772	0.002075169

Table 3. Samples Results STD

Retention time (minutes)	Indensity	Percentage of maximum peak area
46.00016667	641201.0145	0.002150014
46.004	639357.6018	0.002143832
46.008	640115.098	0.002146372
46.01183333	633725.964	0.002124949
46.01583333	636571.712	0.002134491
46.01966667	635096.3357	0.002129544
46.0235	650496.158	0.002181181
46.0275	662605.6737	0.002221786
46.03133333	673550.3078	0.002258484
46.03533333	669284.2488	0.00224418
46.03916667	677743.3194	0.002272544
46.04316667	689140.9738	0.002310761
46.047	666901.2489	0.002236189
46.051	637587.8559	0.002137898
46.05483333	644558.293	0.002161271
46.05883333	662284.0812	0.002220707
46.06266667	676196.8091	0.002267358
46.0665	671495.632	0.002251594
46.0705	664947.7552	0.002229639
46.07433333	663877.8649	0.002226051
46.07833333	650237.2893	0.002180313
46.08216667	643190.5014	0.002156684
46.08616667	633656.2118	0.002124715
46.09	640204.1648	0.002146671
46.094	632198.546	0.002119827
46.09783333	630118.821	0.002112854
46.10183333	645205.2849	0.00216344
46.10566667	652614.2514	0.002188283
46.1095	660467.1788	0.002214615
46.1135	640301.3372	0.002146997
46.1175	637167.5701	0.002136489
46.12133333	645862.5273	0.002165644
46.12533333	640825.532	0.002148754
46.12916667	637335.1577	0.002137051
46.133	634090.002	0.00212617
46.137	641109.2387	0.002149706
46.14083333	635395.7217	0.002130548
46.14483333	620969.898	0.002082176
46.14866667	624684.028	0.00209463
46.15266667	636129.2657	0.002133007
46.1565	651831.6197	0.002185659
46.1605	647147.0708	0.002169951
46.16433333	643068.049	0.002156274
46.16833333	620325.743	0.002080017
46.17216667	602883.2341	0.00202153
46.176	598476.0604	0.002006752
46.18	621967.1462	0.00208552
46.18383333	629935.8094	0.00211224
46.18783333	634473.6601	0.002127456
46.19166667	645661.8298	0.002164971
46.19566667	650097.0391	0.002179843
46.1995	657405.3754	0.002204348
46.2035	661436.5967	0.002217866
46.20733333	645042.4775	0.002162894
46.21133333	638051.7459	0.002139454
46.21516667	623065.7884	0.002089204
46.219	624877.3943	0.002095279
46.223	620771.6271	0.002081512
46.22683333	618789.23	0.002074864
46.23083333	622989.0625	0.002088947
46.23466667	629781.3656	0.002111722
46.23866667	644744.1549	0.002161894
46.2425	654686.723	0.002195232
46.2465	657685.844	0.002205289
46.25033333	643130.5698	0.002156484
46.25416667	629665.3474	0.002111333
46.25816667	613159.7842	0.002055988
46.262	615258.7929	0.002063027
46.266	641645.902	0.002151505
46.26983333	639769.718	0.002145214
46.27383333	639751.6827	0.002145154
46.27766667	643744.315	0.002158541
46.2815	645646.8376	0.002164921
46.2855	651511.6346	0.002184586

Table 3. Samples Results STD

Retention time (minutes)	Indensity	Percentage of maximum peak area
46.28933333	655433.5589	0.002197737
46.29333333	643466.7291	0.002157611
46.29733333	641093.4011	0.002149653
46.30116667	646558.8867	0.002167979
46.30516667	663798.7745	0.002225786
46.309	668485.7801	0.002241502
46.313	687209.3937	0.002304284
46.31683333	669165.0071	0.00224378
46.32083333	656895.7122	0.002202639
46.32466667	659274.4488	0.002210616
46.3285	665894.2851	0.002232813
46.3325	649105.0431	0.002176517
46.33633333	633994.4457	0.002125849
46.34033333	640091.0548	0.002146292
46.34416667	647823.0211	0.002172218
46.34816667	641407.3434	0.002150705
46.352	666344.707	0.002234323
46.356	643893.8713	0.002159043
46.35983333	643588.6247	0.002158019
46.36383333	624898.1911	0.002095348
46.36766667	624689.0319	0.002094647
46.3715	633339.6295	0.002123653
46.3755	627063.798	0.00210261
46.37933333	626925.0309	0.002102145
46.38333333	631856.2796	0.00211868
46.38716667	635008.606	0.00212925
46.39116667	660012.1403	0.002213089
46.395	661912.8941	0.002219463
46.399	665452.958	0.002231333
46.40283333	676019.7669	0.002266764
46.40683333	678750.1255	0.00227592
46.41066667	675306.3434	0.002264372
46.4145	641631.1857	0.002151456
46.4185	623897.1865	0.002091992
46.42233333	623604.1802	0.002091009
46.42633333	641699.6853	0.002151686
46.43016667	640759.4077	0.002148533
46.43416667	643369.0837	0.002157283
46.438	643729.0384	0.00215849
46.442	640333.4165	0.002147104
46.44583333	644395.5941	0.002160725
46.44983333	667398.5434	0.002237857
46.45366667	673688.22	0.002258946
46.4575	667443.8612	0.002238008
46.4615	663345.0361	0.002224265
46.46533333	668613.6395	0.002241931
46.46933333	671960.4161	0.002253153
46.47316667	676807.3069	0.002269405
46.47716667	657863.8128	0.002205886
46.481	641676.9359	0.002151609
46.485	629172.4861	0.002109681
46.48883333	627326.0778	0.002103489
46.49266667	637668.8811	0.00213817
46.49666667	653989.3526	0.002192894
46.5005	644719.0542	0.00216181
46.5045	642962.7177	0.002155921
46.50833333	654532.7144	0.002194716
46.51233333	675918.1988	0.002266424
46.51616667	666321.4034	0.002234245
46.52016667	657647.1666	0.002205159
46.524	643136.065	0.002156502
46.528	648285.4262	0.002173768
46.53183333	650650.4147	0.002181698
46.53566667	650517.2543	0.002181252
46.53966667	616019.5407	0.002065577
46.5435	605586.7986	0.002030595
46.5475	621617.2225	0.002084347
46.55133333	629523.0761	0.002110856
46.55533333	621972.0243	0.002085537
46.55916667	635058.2766	0.002129416
46.563	644924.3088	0.002162498
46.567	660538.9852	0.002214856
46.571	665113.4304	0.002230194
46.57483333	664828.6289	0.002229239

Table 3. Samples Results STD

Retention time (minutes)	Indensity	Percentage of maximum peak area
46.57866667	659027.1336	0.002209786
46.58266667	654726.2905	0.002195365
46.5865	664168.6066	0.002227026
46.5905	677467.0856	0.002271617
46.59433333	672872.4761	0.002256211
46.59833333	669707.4543	0.002245599
46.60216667	662233.7323	0.002220538
46.606	661193.0558	0.002217049
46.61	664023.7834	0.002226541
46.61383333	669504.8819	0.002244919
46.61783333	671411.9391	0.002251314
46.62166667	646071.4514	0.002166345
46.62566667	636742.2816	0.002135063
46.6295	619439.174	0.002077044
46.6335	620012.1894	0.002078965
46.63733333	626048.341	0.002099205
46.64116667	634836.2916	0.002128672
46.64516667	645397.4045	0.002164084
46.649	657434.7561	0.002204447
46.653	647309.9062	0.002170497
46.65683333	659007.5478	0.002209721
46.66083333	639893.6212	0.00214563
46.66466667	629999.2288	0.002112453
46.66866667	629042.8778	0.002109246
46.6725	628907.5136	0.002108792
46.6765	625980.0161	0.002098976
46.68033333	630062.2851	0.002112664
46.68416667	641270.9874	0.002150248
46.68816667	666520.6975	0.002234913
46.692	659316.4613	0.002210756
46.696	648547.9149	0.002174648
46.69983333	631246.3862	0.002116635
46.70383333	626261.4338	0.00209992
46.70766667	624919.319	0.002095419
46.71166667	631608.428	0.002117849
46.7155	639105.004	0.002142985
46.7195	637411.9926	0.002137309
46.72333333	641983.0468	0.002152636
46.72716667	656549.567	0.002201479
46.73116667	675551.262	0.002265193
46.735	661691.9507	0.002218722
46.739	658417.0536	0.002207741
46.74283333	644862.4312	0.002162291
46.74683333	649428.1515	0.0021776
46.75066667	663621.9525	0.002225193
46.75466667	683509.0004	0.002291877
46.7585	682369.8405	0.002288057
46.7625	680684.056	0.002282404
46.76633333	675087.0957	0.002263637
46.77016667	652331.1522	0.002187334
46.77416667	652616.8354	0.002188292
46.778	667970.7805	0.002239775
46.782	643190.9387	0.002156686
46.78583333	659479.5921	0.002211303
46.78983333	684367.4542	0.002294755
46.79366667	696428.1818	0.002335196
46.7975	697164.0736	0.002337663
46.8015	684844.1509	0.002296353
46.80533333	646519.8817	0.002167848
46.80933333	638092.7417	0.002139591
46.81316667	644622.6415	0.002161487
46.81716667	651257.1676	0.002183733
46.821	642378.364	0.002153961
46.825	639361.5612	0.002143846
46.82883333	636121.6577	0.002132982
46.83283333	660554.2017	0.002214907
46.83666667	642092.4348	0.002153003
46.8405	645010.7819	0.002162788
46.8445	654333.8407	0.002194049
46.84833333	665583.9346	0.002231772
46.85233333	688559.8888	0.002308813
46.85616667	687357.8604	0.002304782
46.86016667	677866.0913	0.002272955
46.864	646012.8106	0.002166148

Table 3. Samples Results STD

Retention time (minutes)	Indensity	Percentage of maximum peak area
46.868	619225.9529	0.002076329
46.87183333	603246.5333	0.002022748
46.87566667	616391.8891	0.002066826
46.87966667	636205.6931	0.002133264
46.8835	642975.9863	0.002155965
46.8875	639993.2869	0.002145964
46.89133333	645960.4018	0.002165972
46.89533333	655277.2131	0.002197212
46.89916667	660397.1936	0.00221438
46.90316667	670708.8284	0.002248956
46.907	665934.1274	0.002232946
46.911	660948.1886	0.002216228
46.91483333	661220.3707	0.00221714
46.91866667	662087.6092	0.002220048
46.92266667	659958.3747	0.002212909
46.9265	652080.5678	0.002186494
46.9305	653044.813	0.002189727
46.93433333	639115.6158	0.002143021
46.93833333	649475.1822	0.002177758
46.94216667	645237.7138	0.002163549
46.946	644545.4961	0.002161228
46.95	644501.9383	0.002161082
46.95383333	654386.5899	0.002194226
46.95783333	659278.2645	0.002210628
46.96166667	657583.4102	0.002204945
46.96566667	664094.629	0.002226778
46.9695	660793.4588	0.002215709
46.9735	665753.1394	0.002232339
46.97733333	666425.0801	0.002234592
46.98133333	665464.4322	0.002231371
46.98516667	651316.9307	0.002183933

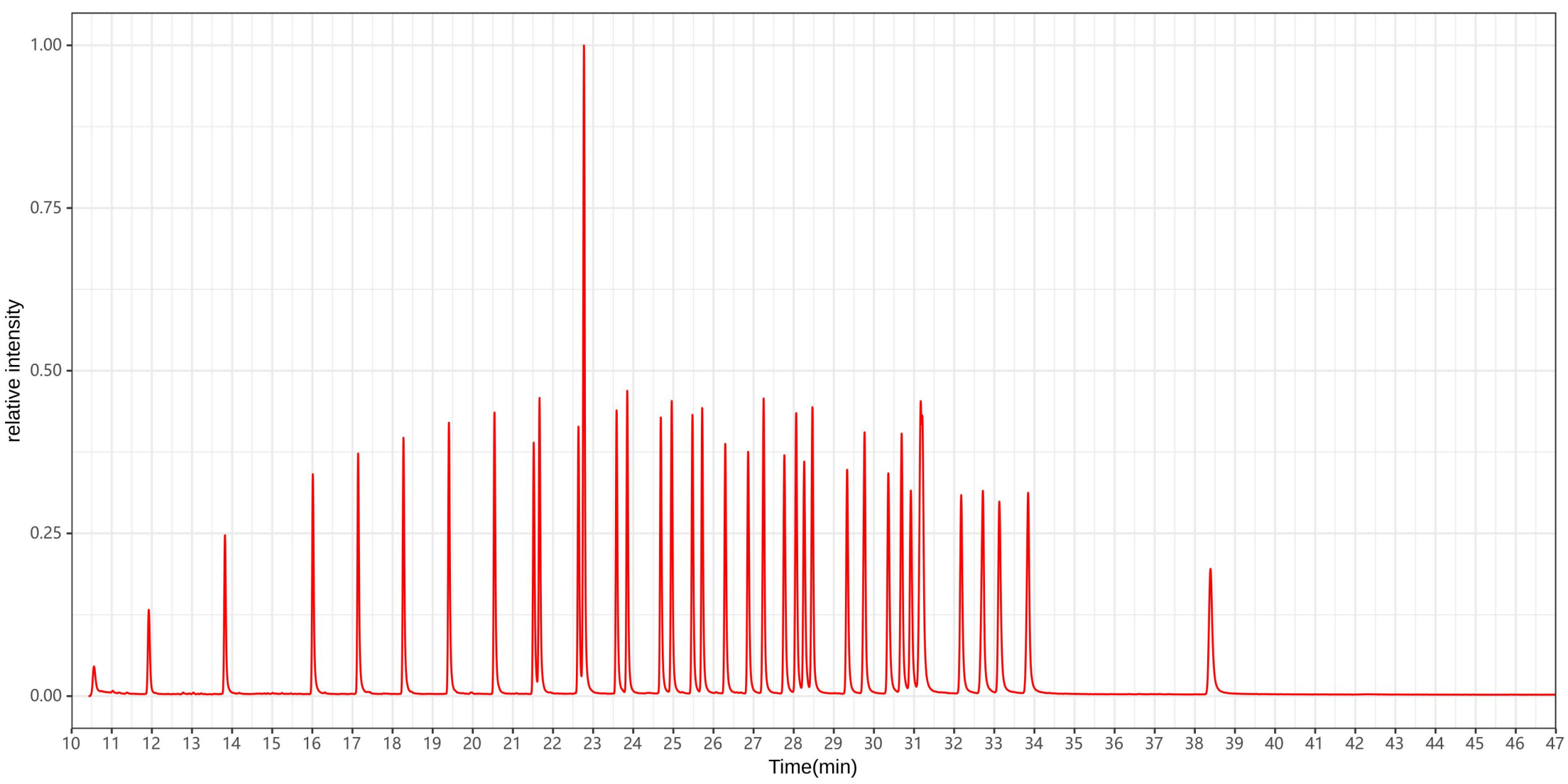

Figure 3. Result of Oil Samples (STD)

Table 4. Oil Sample Calculate Results

Sample Name	No 4. Oil Samplie_1	No 4. Oil Samplie_2	No 4. Oil Samplie_3	No 4. Oil Samplie_4	Average	SD	Proportion of fatty acids / %
C12:0 ug/ml	0.0382	0.1102	0.0511	0.0856	0.0713	0.0284	0.0048
C14:0 ug/ml	0.7543	1.3085	1.1756	1.1299	1.0921	0.2058	0.0742
C15:0 ug/ml	0.0571	0.0906	0.0649	0.0286	0.0603	0.0221	0.0041
C16:0 ug/ml	80.4841	144.4967	129.1287	130.0010	121.0276	24.1905	8.2273
C16:1 ug/ml	2.0247	3.4542	2.8344	2.6385	2.7380	0.5101	0.1861
C17:0 ug/ml	0.2805	0.5724	0.5256	0.3512	0.4324	0.1204	0.0294
C18:0 ug/ml	57.1049	104.3571	104.7075	91.0578	89.3068	19.3889	6.0710
C18:1n9c ug/ml	269.5299	367.2588	349.0073	359.4810	336.3193	39.1009	22.8626
C18:2n6c ug/ml	425.9187	716.5888	605.5238	628.4496	594.1202	105.5922	40.3877
C20:0 ug/ml	1.6464	3.2220	2.9614	2.3497	2.5449	0.6077	0.1730
C18:3n6 ug/ml	1.1241	1.3532	1.3794	1.2246	1.2703	0.1028	0.0864
C20:1 ug/ml	1.1030	2.0308	1.0418	1.9456	1.5303	0.4594	0.1040
C18:3n3 ug/ml	232.3224	376.3816	313.2492	356.2991	319.5631	55.2917	21.7236
C22:0 ug/ml	0.2995	0.6382	0.6679	0.5049	0.5276	0.1453	0.0359
C23:0 ug/ml	0.0784	0.2143	0.1715	0.1885	0.1632	0.0513	0.0111
C24:0 ug/ml	0.1180	0.3914	0.3388	0.2576	0.2765	0.1032	0.0188
**Total fatty acids**	**1072.8842**	**1722.4688**	**1512.8289**	**1575.9932**	**1471.0438**	**242.1291**	**100.0000**
ω-3 PUFA	232.3224	376.3816	313.2492	356.2991	319.5631	55.2917	21.7236
ω-6 PUFA	427.0428	717.9420	606.9032	629.6742	595.3906	105.6737	40.4740
ω-6 / ω-3	1.8381	1.9075	1.9374	1.7673	1.8631	1.9112	1.8631

定量分析报告（根据样品）

Agilent Technologies

**采集日期时间**	2025/5/13 9:07:10	**数据文件**	hz250417010.D
**类型**	样品	**名称**	hz250417010
**稀释**	1	**采集方法文件**	FAME-TEST-SCAN

样品色谱图

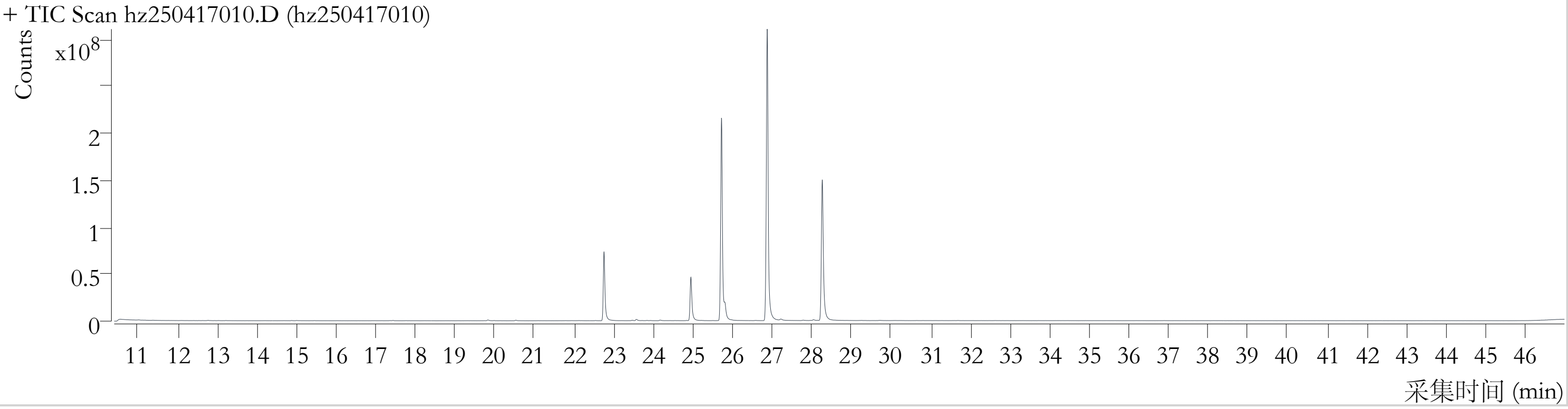

名称	RT	离子对	响应	峰高	最终浓度 单位	比值
C4:0	10.595	74.0	0	2101.27	ND ug/ml	
C6:0	11.834	74.0	0	2742.12	ND ug/ml	
C10:0	16.009	74.0	0	2630.15	ND ug/ml	
C11:0	17.111	74.0	0	3961.99	ND ug/ml	
C12:0	18.268	74.0	16319	9128.97	0.0382 ug/ml	92.5
C14:0	20.527	74.0	303784	94163.12	0.7543 ug/ml	52.9
C14:1	21.398	55.0	0	9507.49	ND ug/ml	
C15:0	21.660	74.0	22112	9631.98	0.0571 ug/ml	18.1
C15:1	22.758	55.0	0	1439340.13	ND ug/ml	
C16:0	22.758	87.0	28918238	9506980.91	80.4841 ug/ml	56.9
C16:1	23.567	55.0	149583	42587.81	2.0247 ug/ml	64.2
C17:0	23.841	74.0	96406	36565.90	0.2805 ug/ml	55.2
C17:1	24.951	55.0	0	907924.69	ND ug/ml	
C18:0	24.947	86.9	18665684	5549039.59	57.1049 ug/ml	59.5
C18:1n9t	25.760	55.0	0	1260797.13	ND ug/ml	
C18:1n9c	25.721	55.0	18734545	4797892.41	269.5299 ug/ml	93.4
C18:2n6t	25.721	81.0	0	4043898.75	ND ug/ml	
C18:2n6c	26.874	80.9	64878341	19413055.48	425.9187 ug/ml	36.2
C20:0	27.226	86.9	546041	142045.51	1.6464 ug/ml	52.4
C18:3n6	27.781	79.0	185393	55082.91	1.1241 ug/ml	-8.6
C20:1	28.046	55.0	82378	24695.54	1.1030 ug/ml	73.3
C18:3n3	28.265	78.9	49276752	13079233.08	232.3224 ug/ml	60.5
C21:0	28.261	86.9	0	1744460.63	ND ug/ml	
C20:2	29.426	81.0	0	9765.68	ND ug/ml	
C22:0	29.727	86.9	90495	19764.76	0.2995 ug/ml	57.1
C20:3n3	30.833	79.0	0	7831.44	ND ug/ml	
C23:0	31.095	86.9	24640	8590.15	0.0784 ug/ml	62.4
C22:2	32.146	81.0	0	10390.96	ND ug/ml	
C24:0	32.615	86.9	32108	9647.74	0.1180 ug/ml	-4.5
C20:5n3	33.108	79.0	0	7740.31	ND ug/ml	
C22:6n3	38.404	90.9	0	3854.28	ND ug/ml	

C4:0

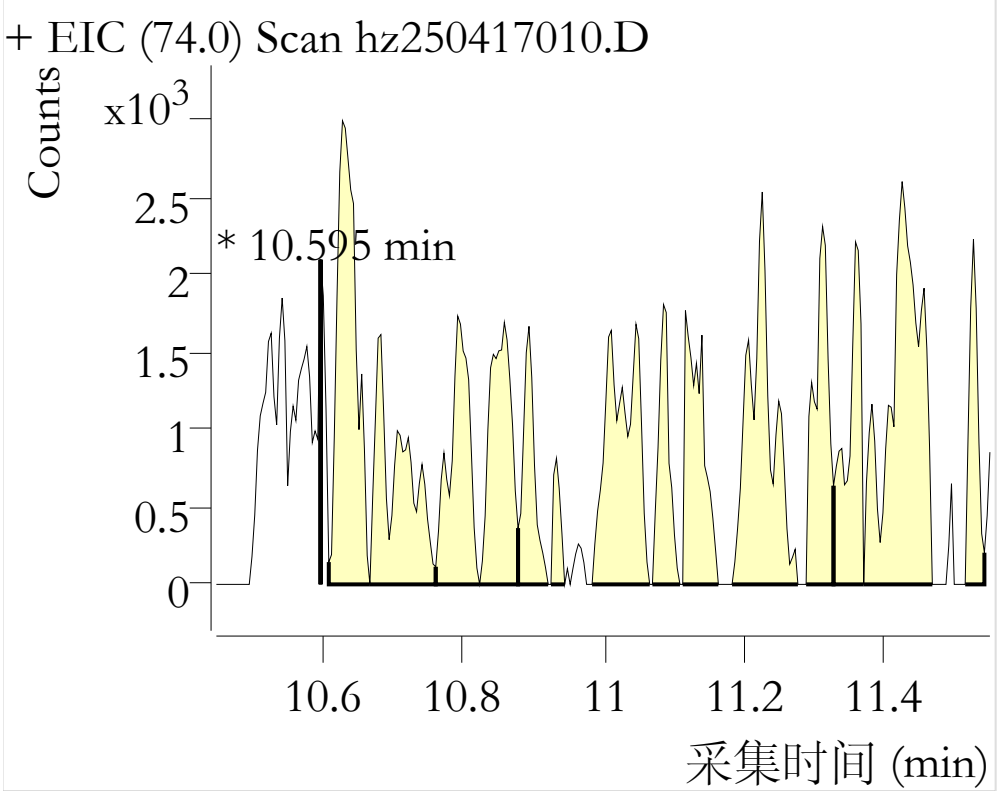

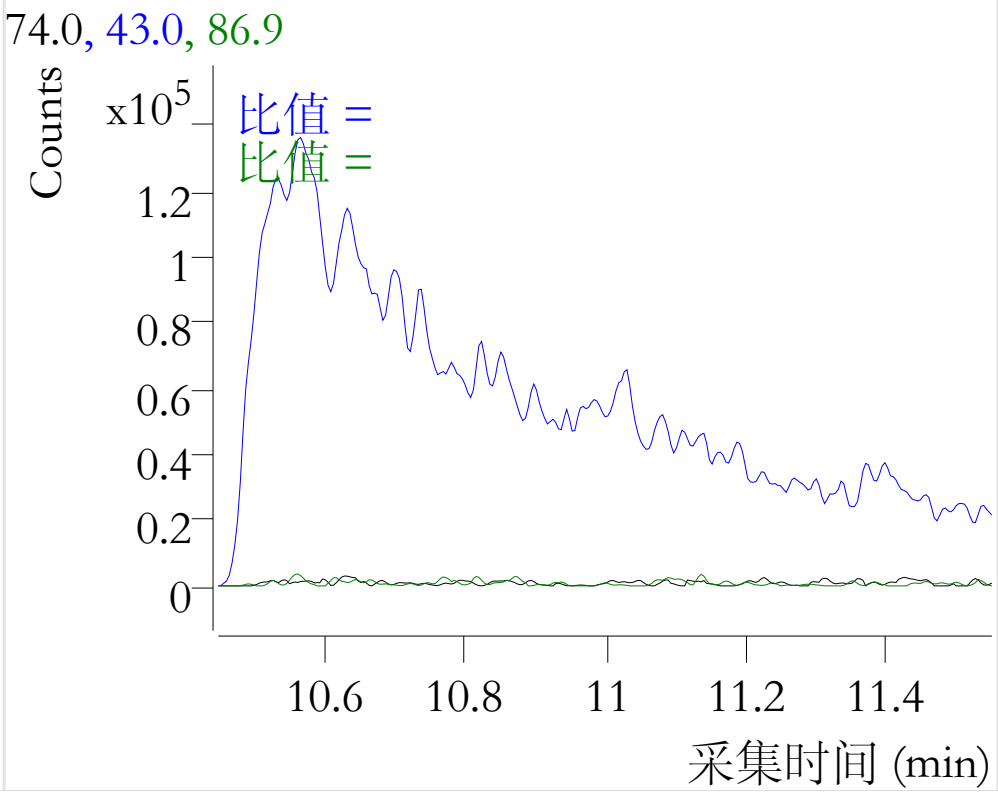

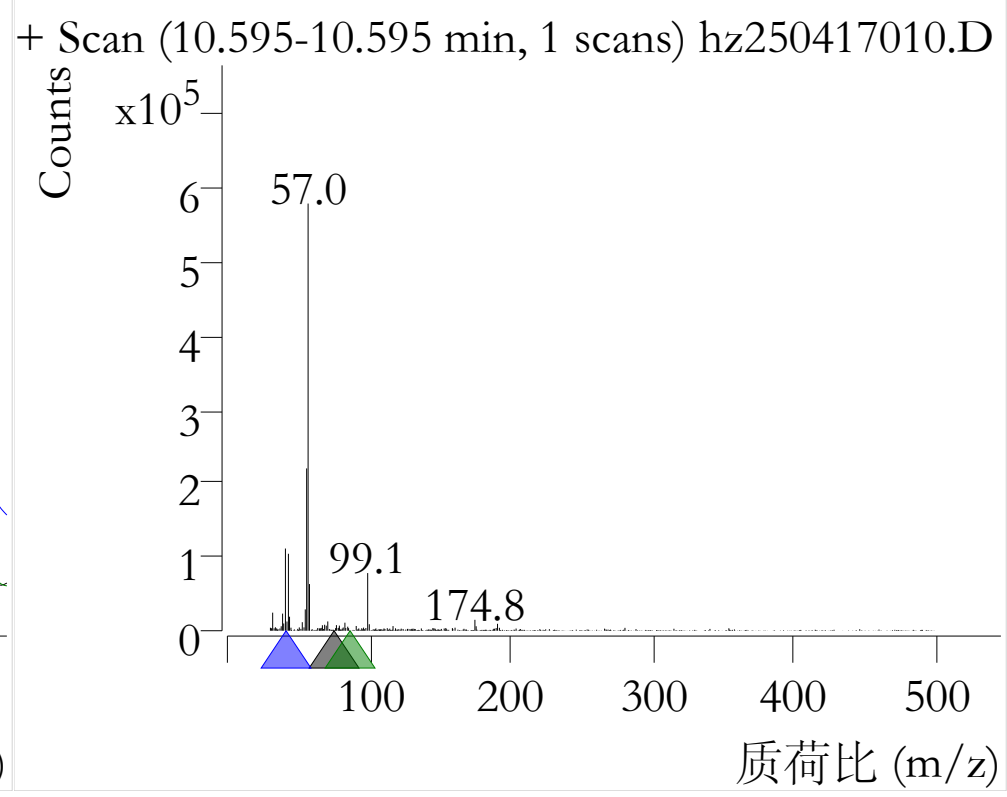

C6:0

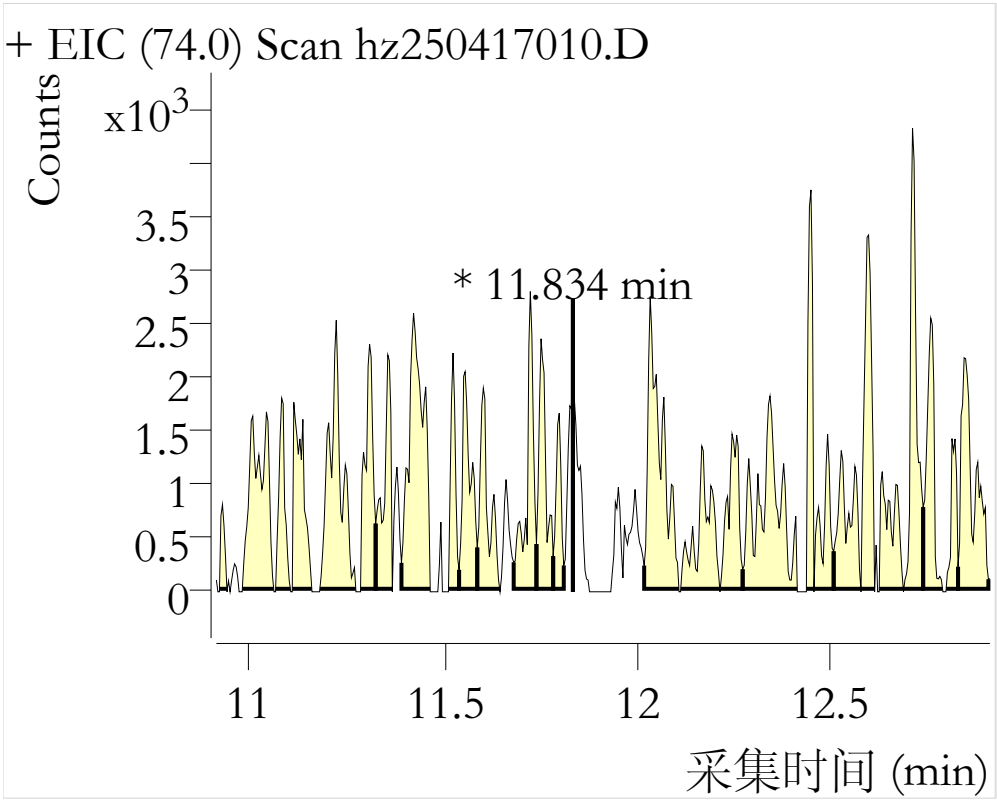

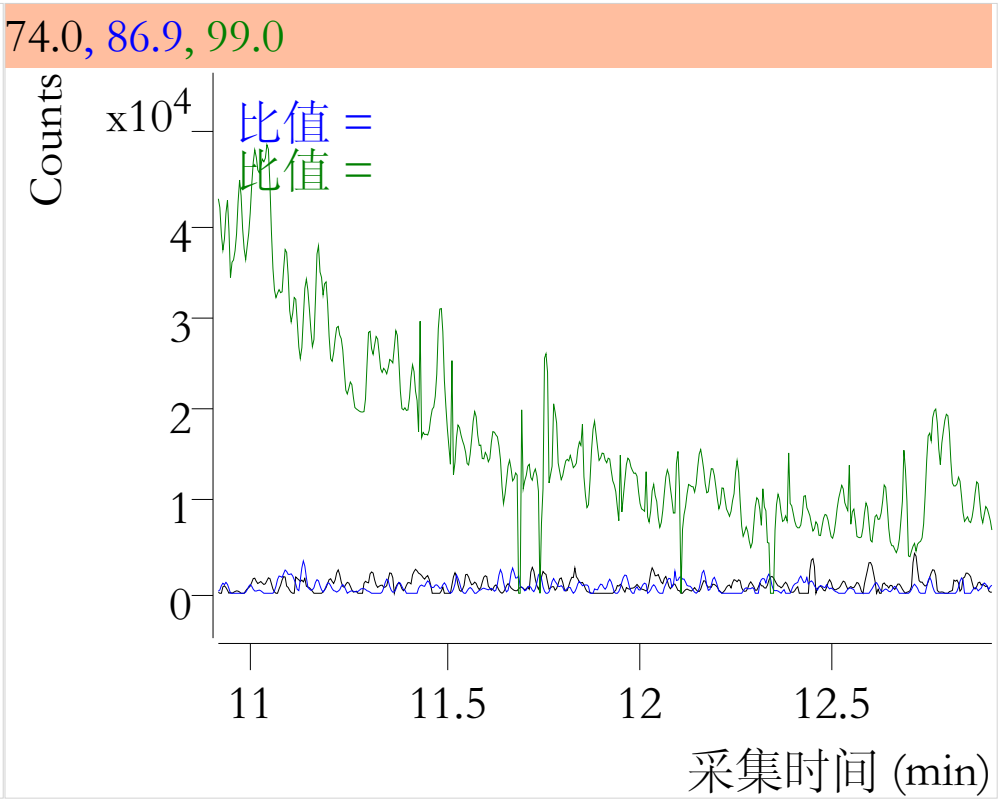

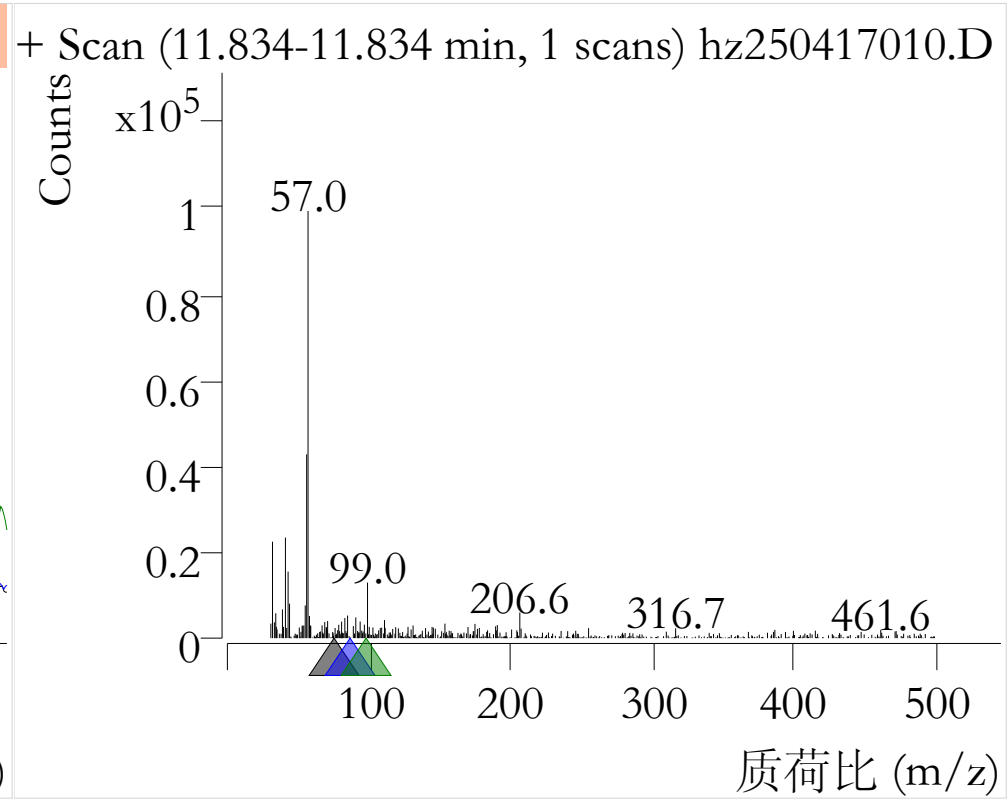

C8:0

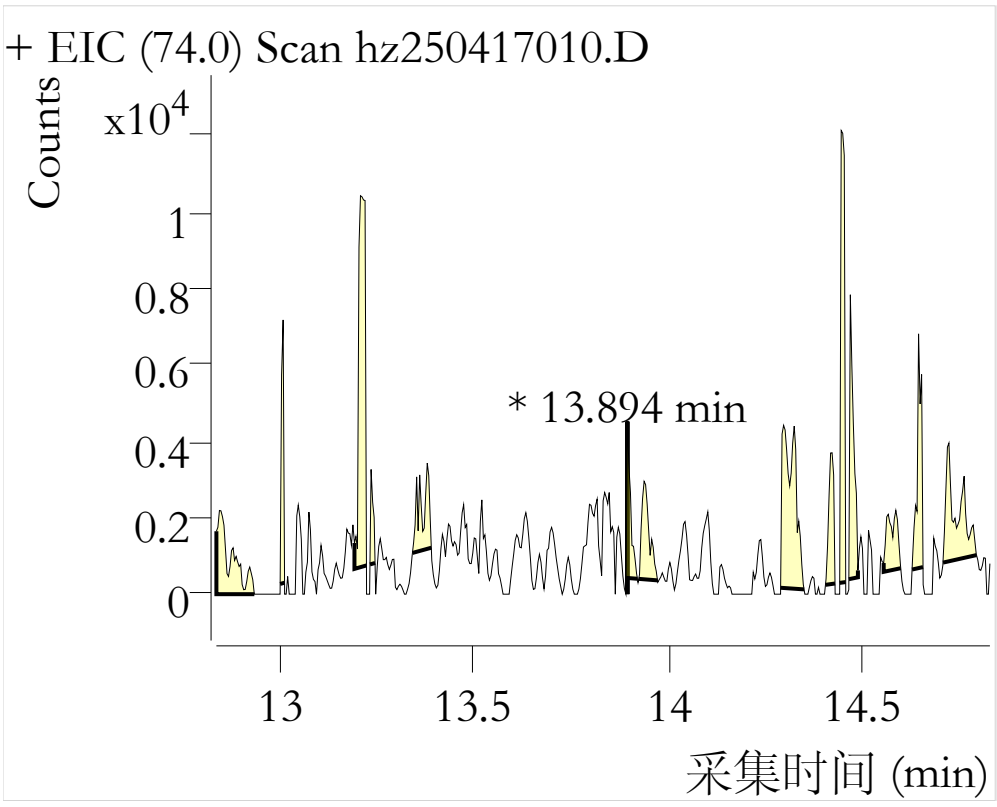

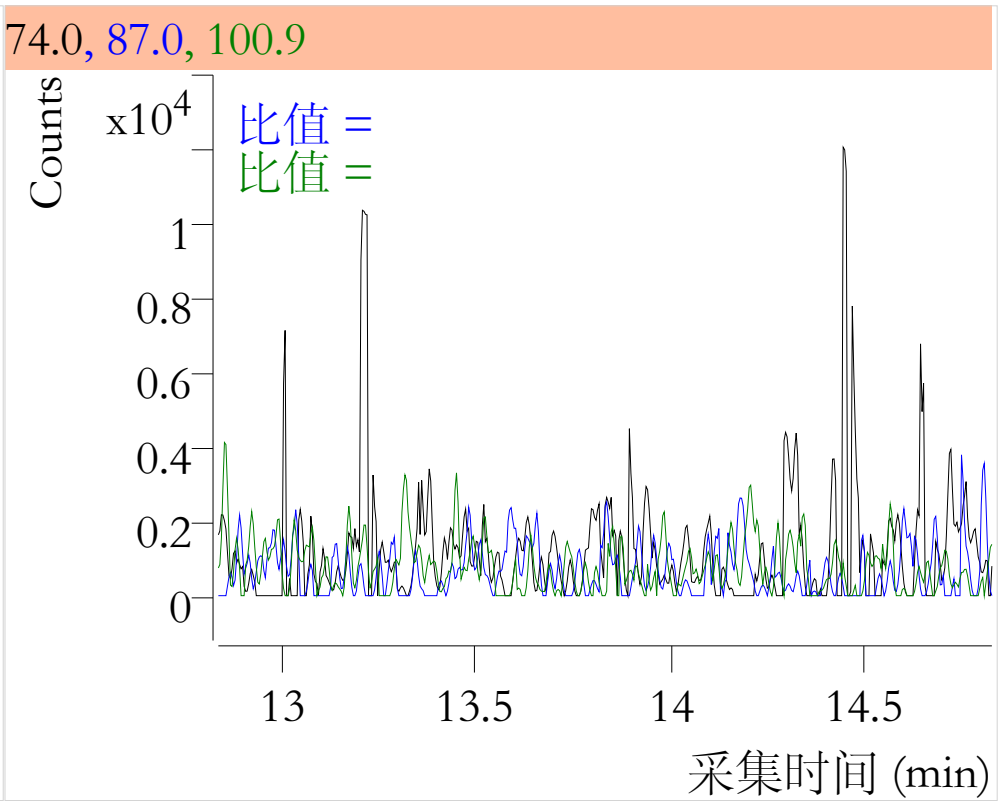

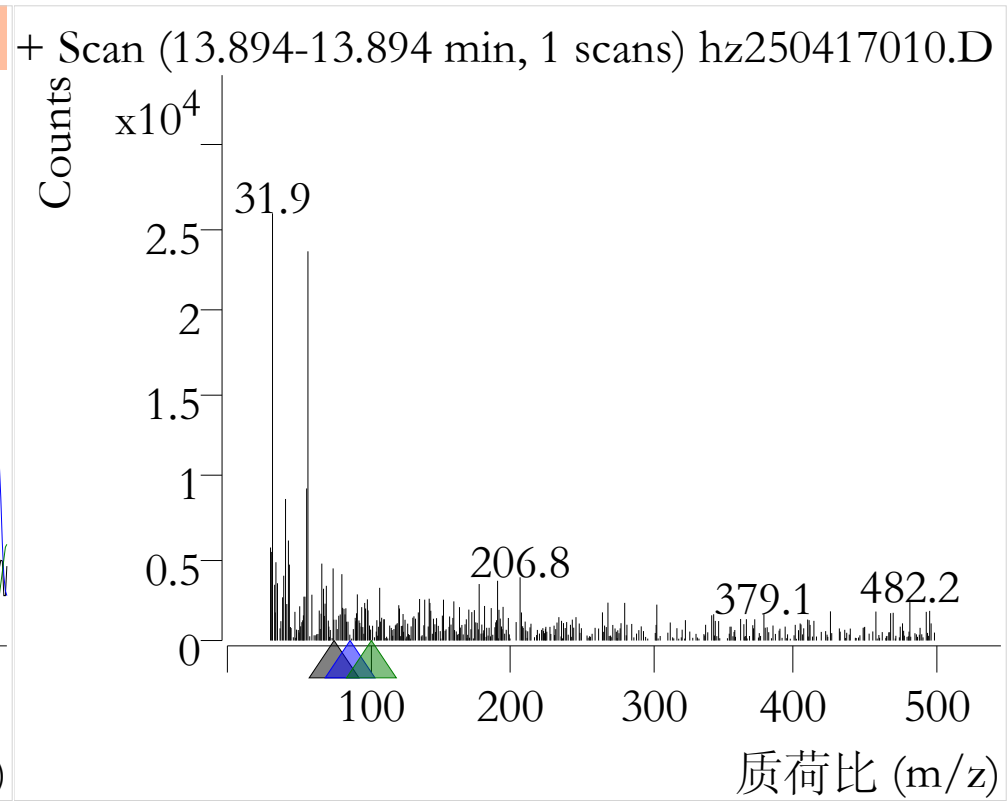

C10:0

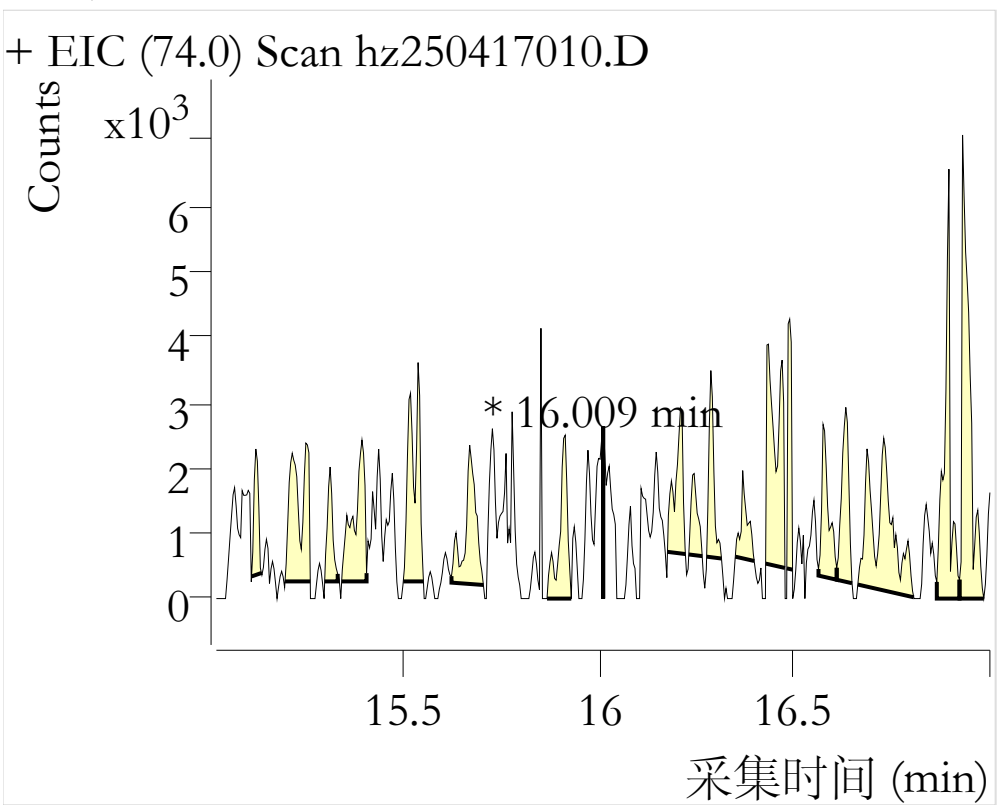

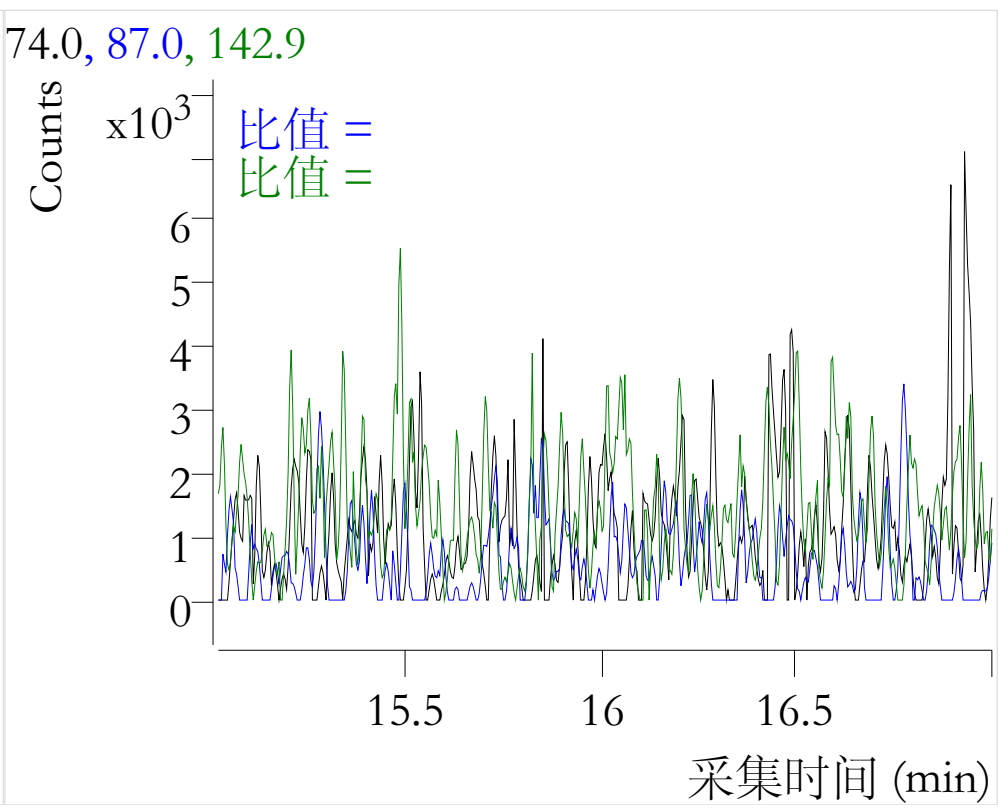

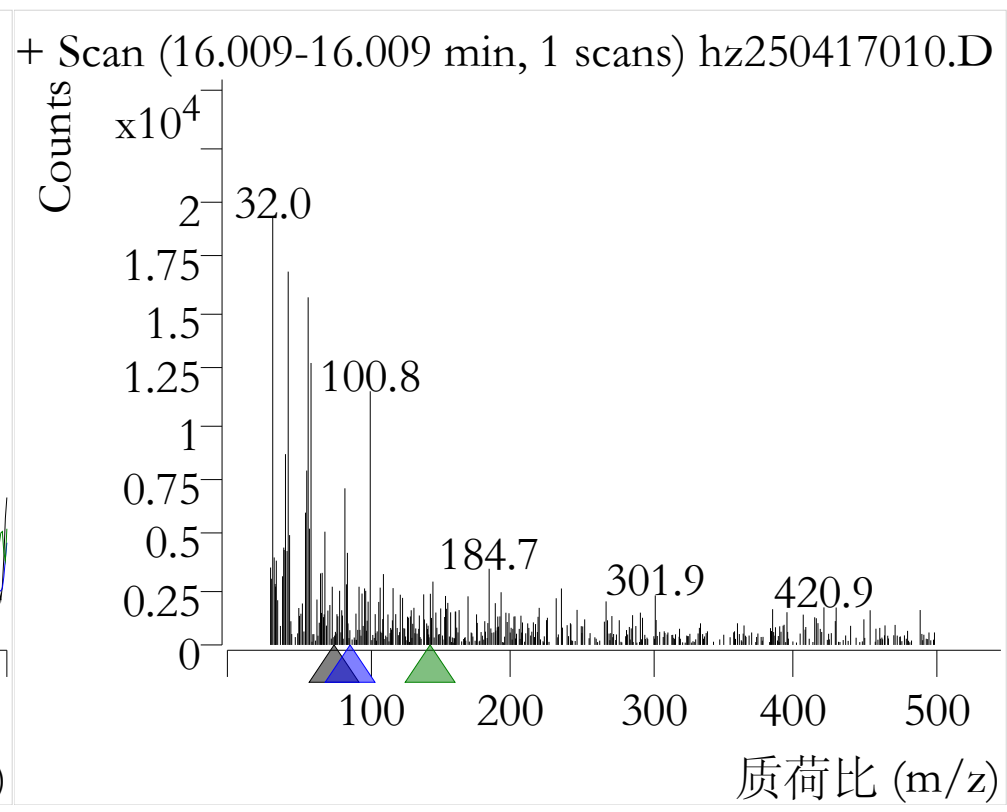

C11:0

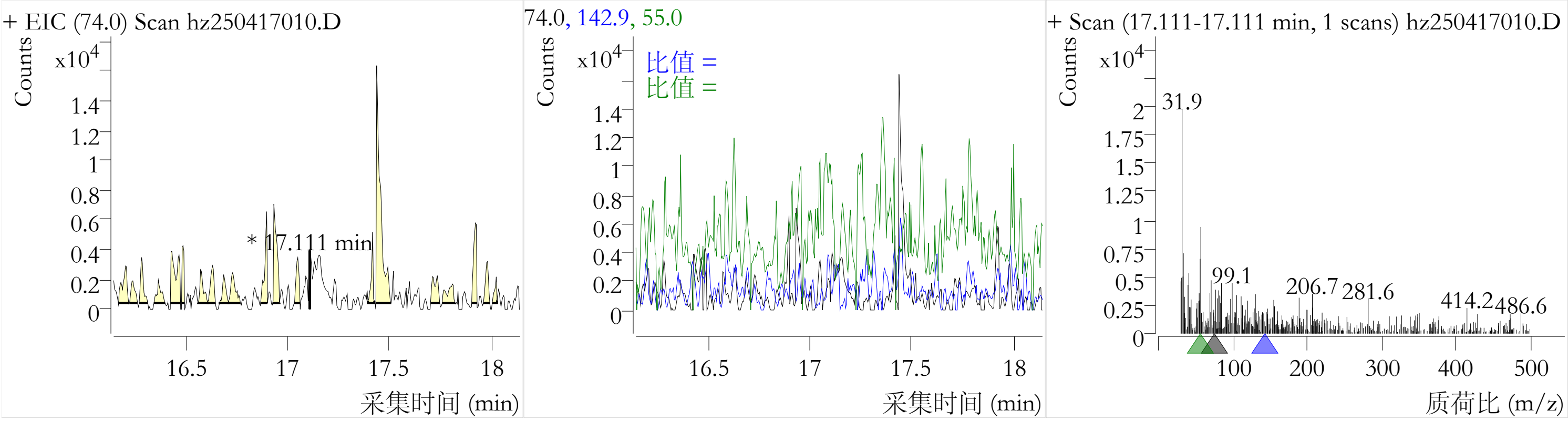

C12:0

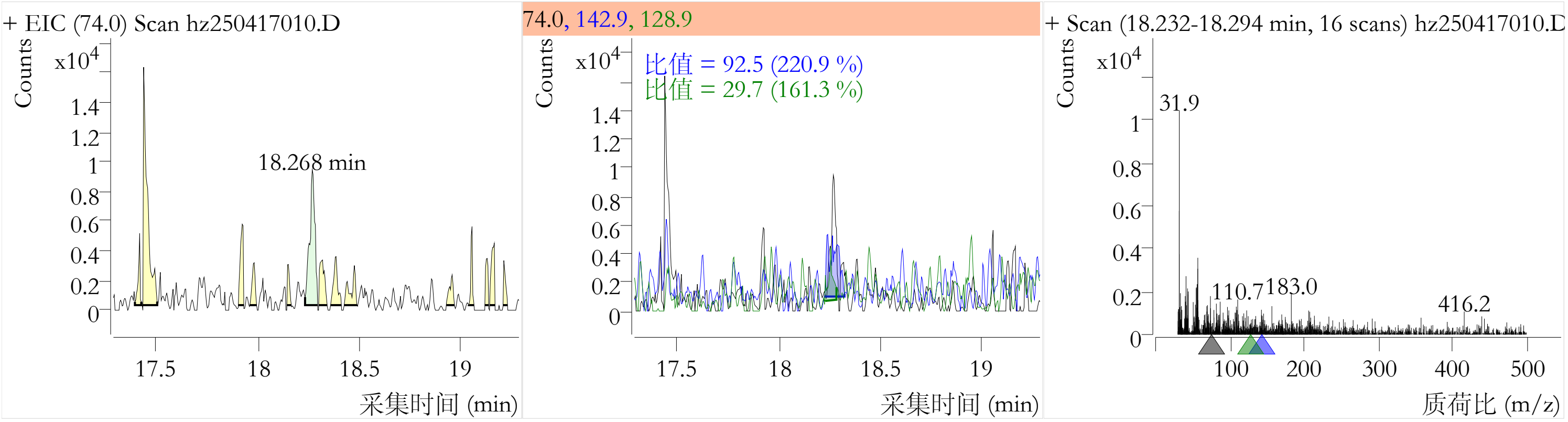

C13:0

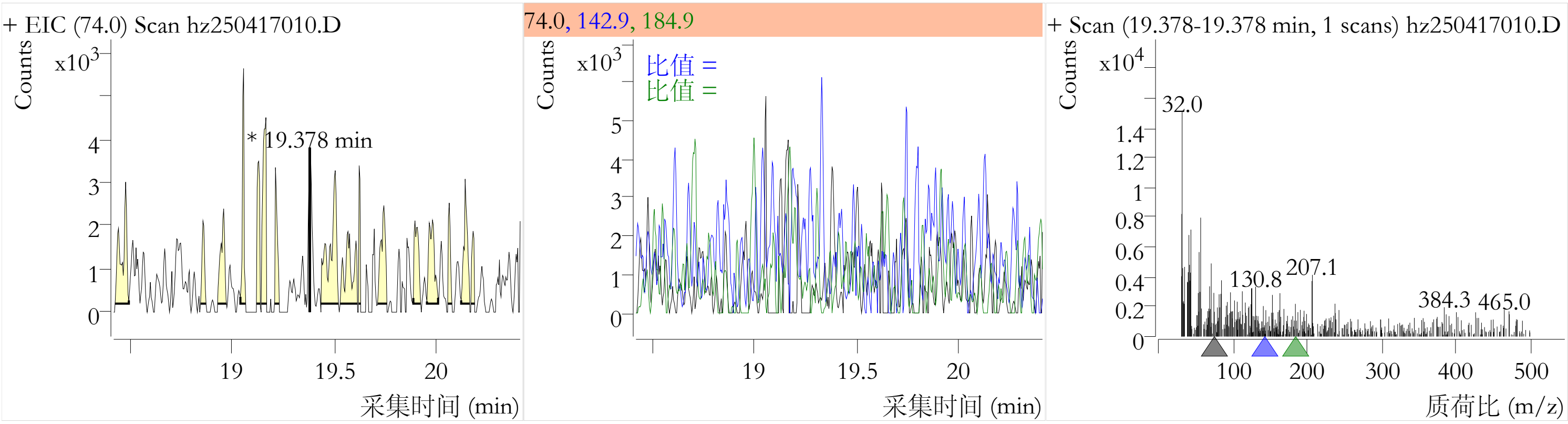

C14:0

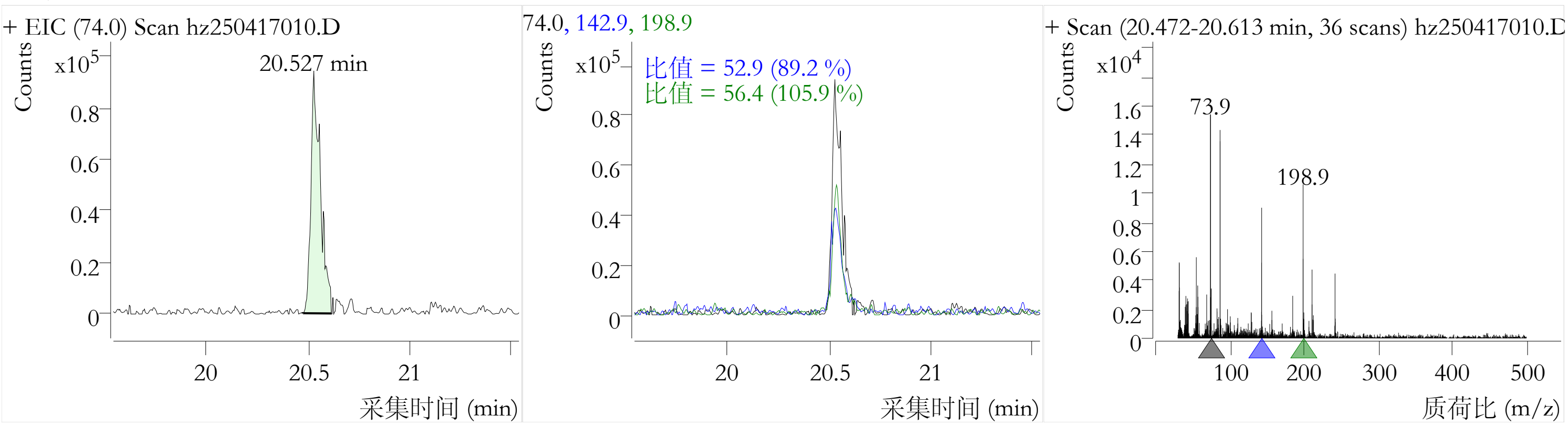

C14:1

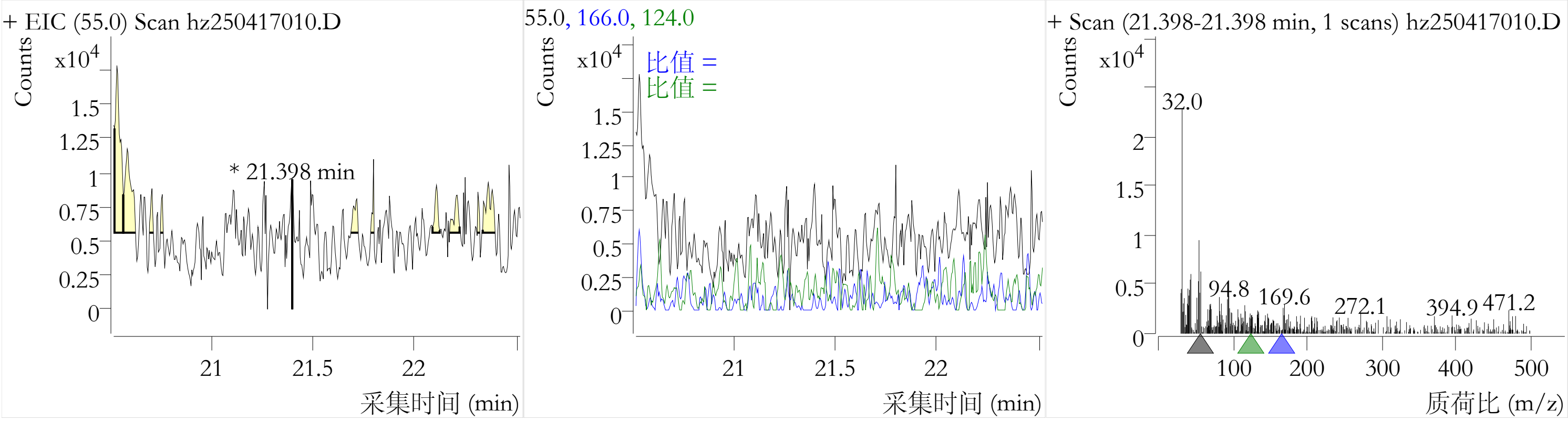

C15:0

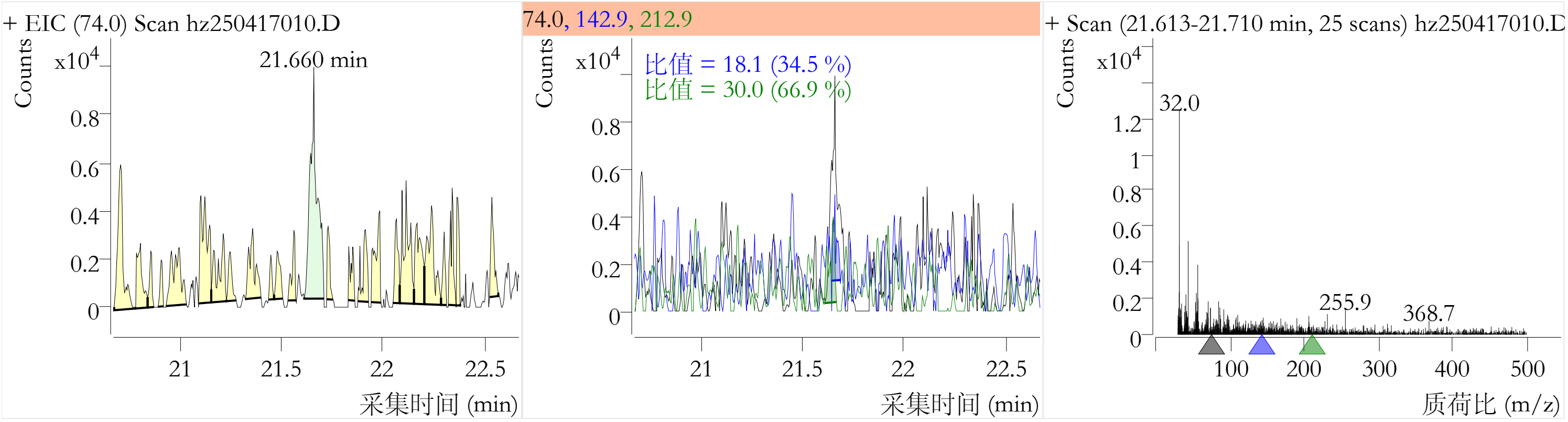

C15:1

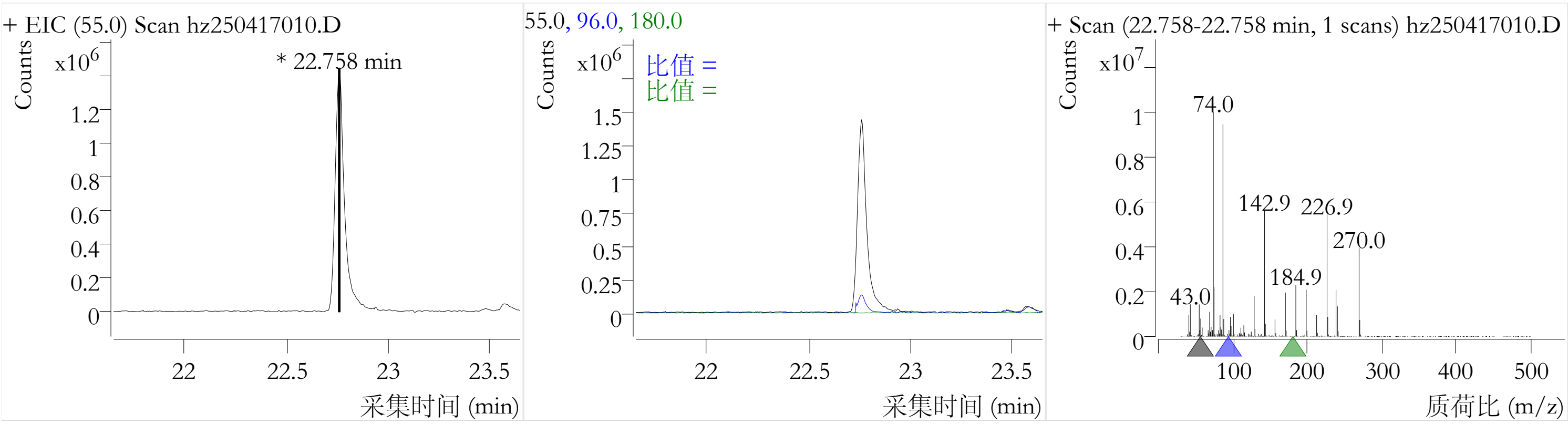

C16:0

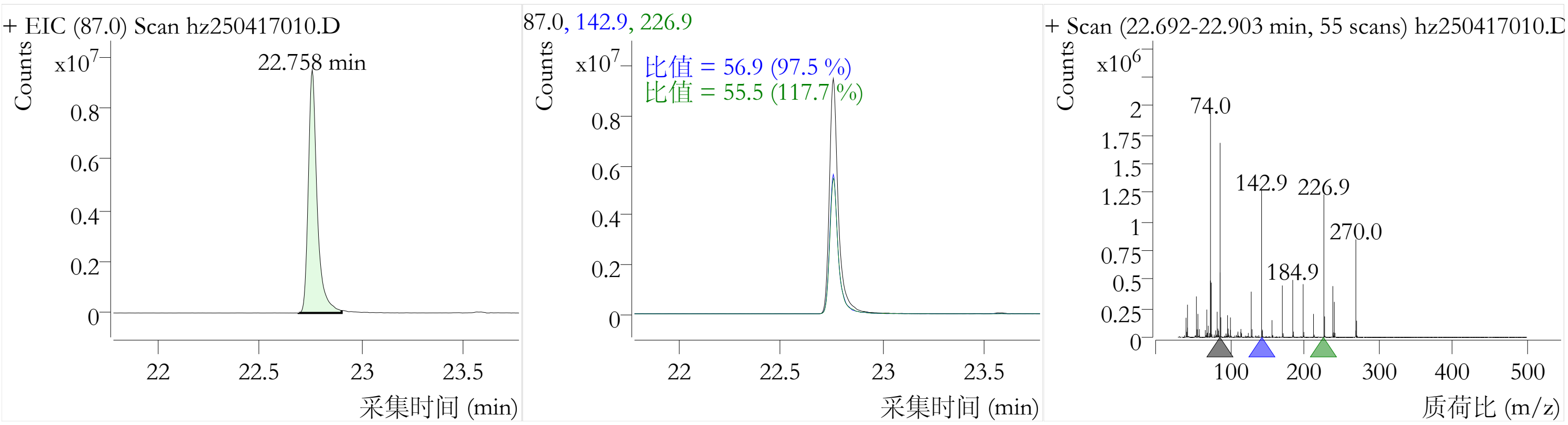

C16:1

+ EIC (55.0) Scan hz250417010.D

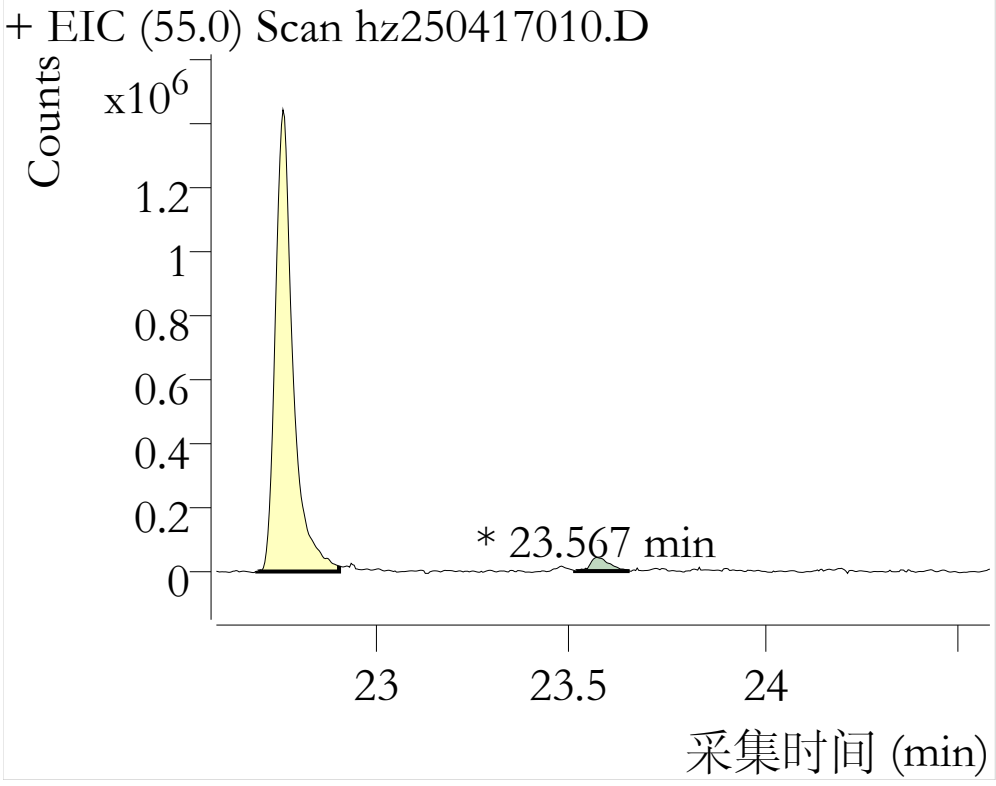

55.0, 152.0, 194.0

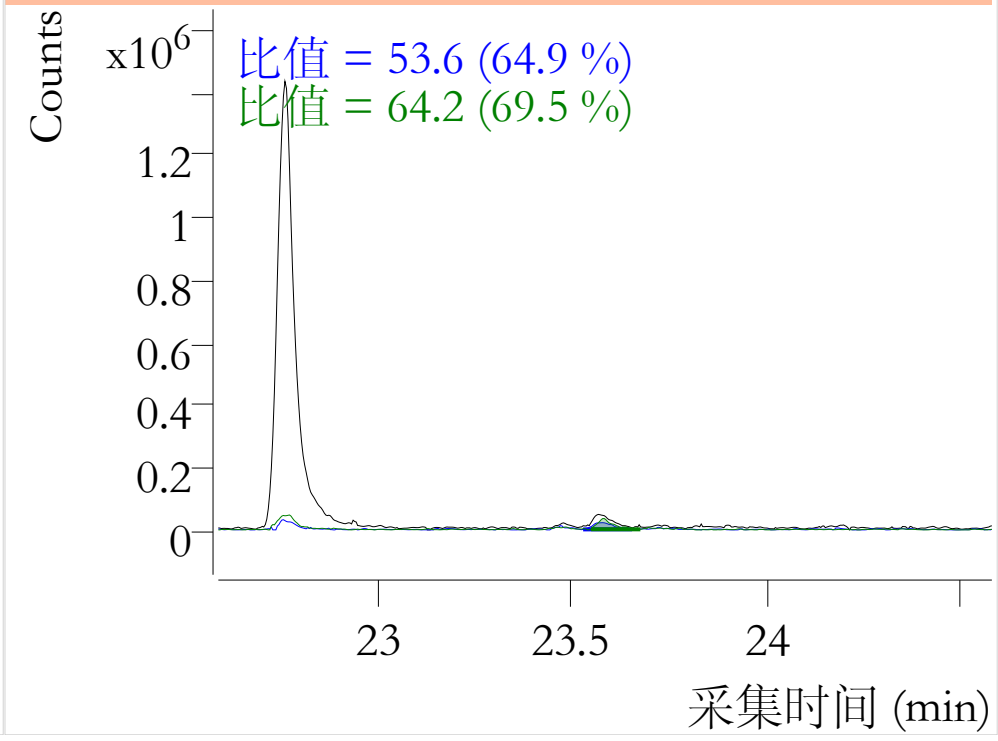

+ Scan (23.513-23.653 min, 37 scans) hz250417010.D

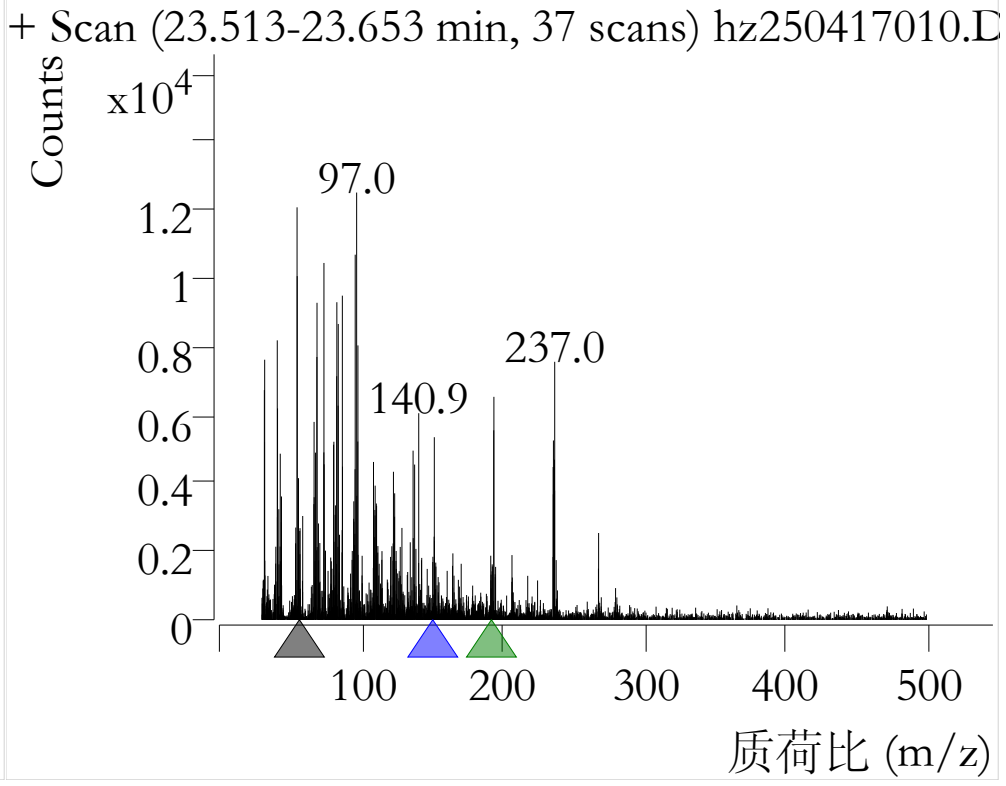

C17:0

+ EIC (74.0) Scan hz250417010.D

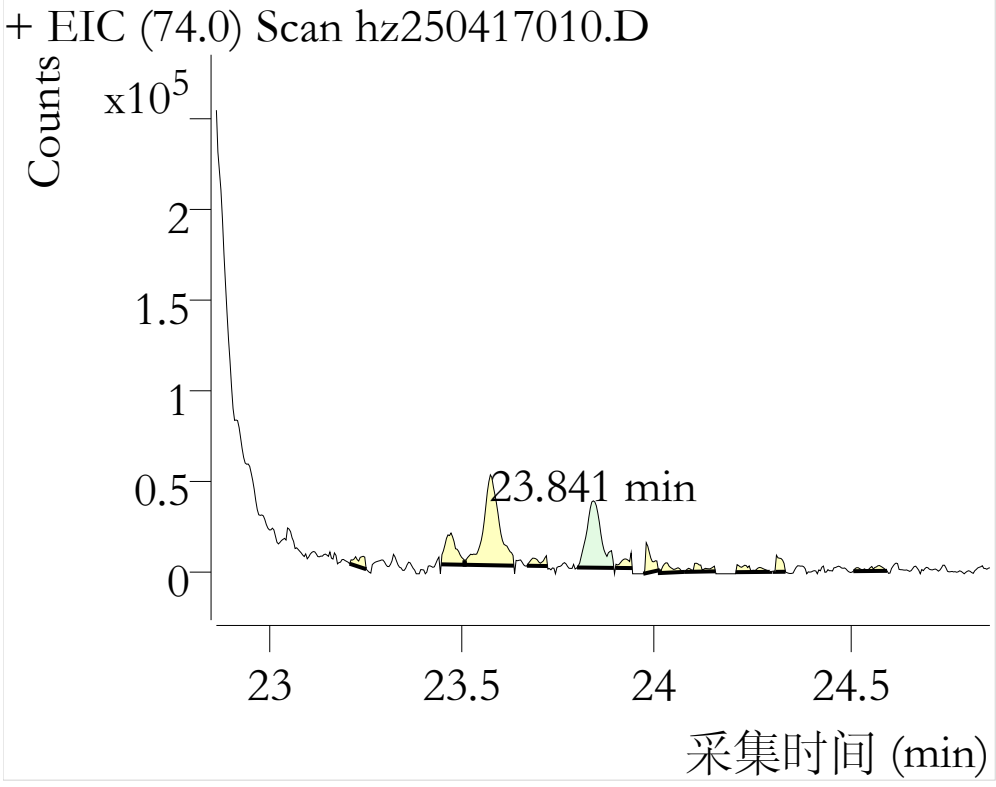

74.0, 142.9, 184.9

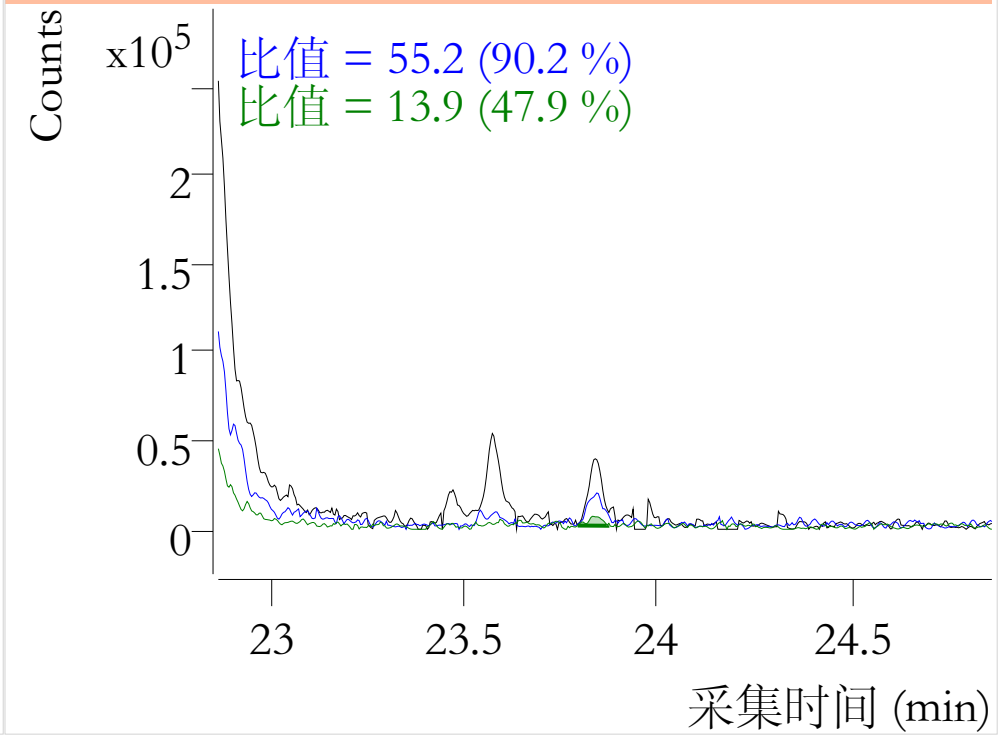

+ Scan (23.799-23.894 min, 24 scans) hz250417010.D

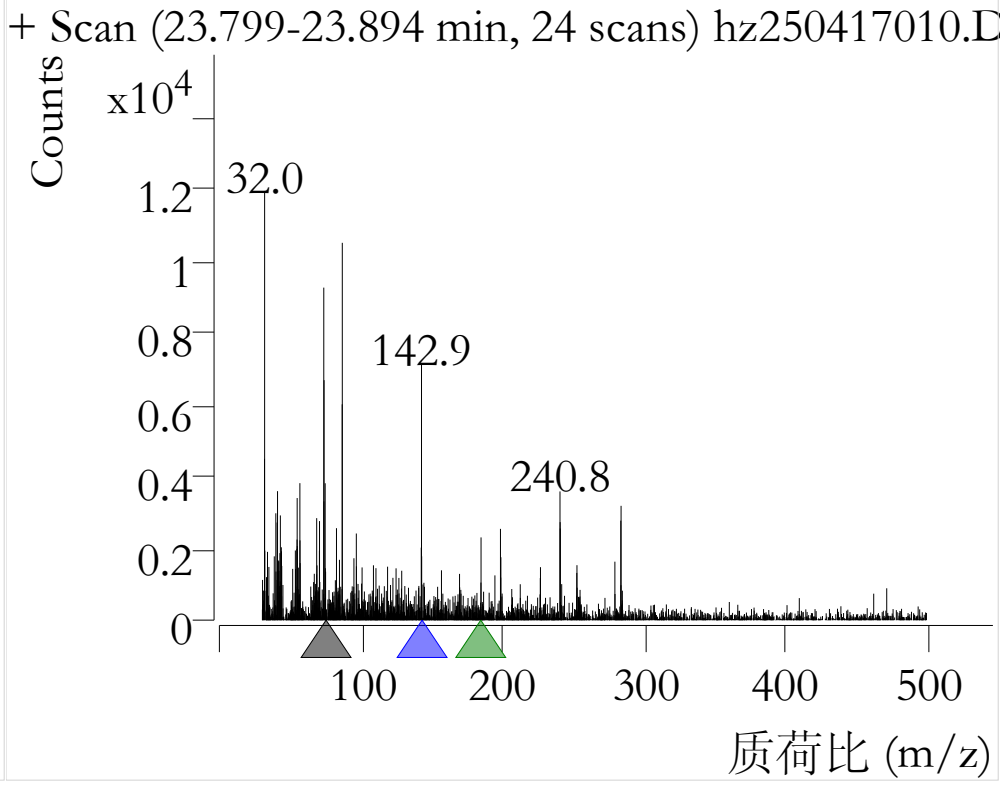

C17:1

+ EIC (55.0) Scan hz250417010.D

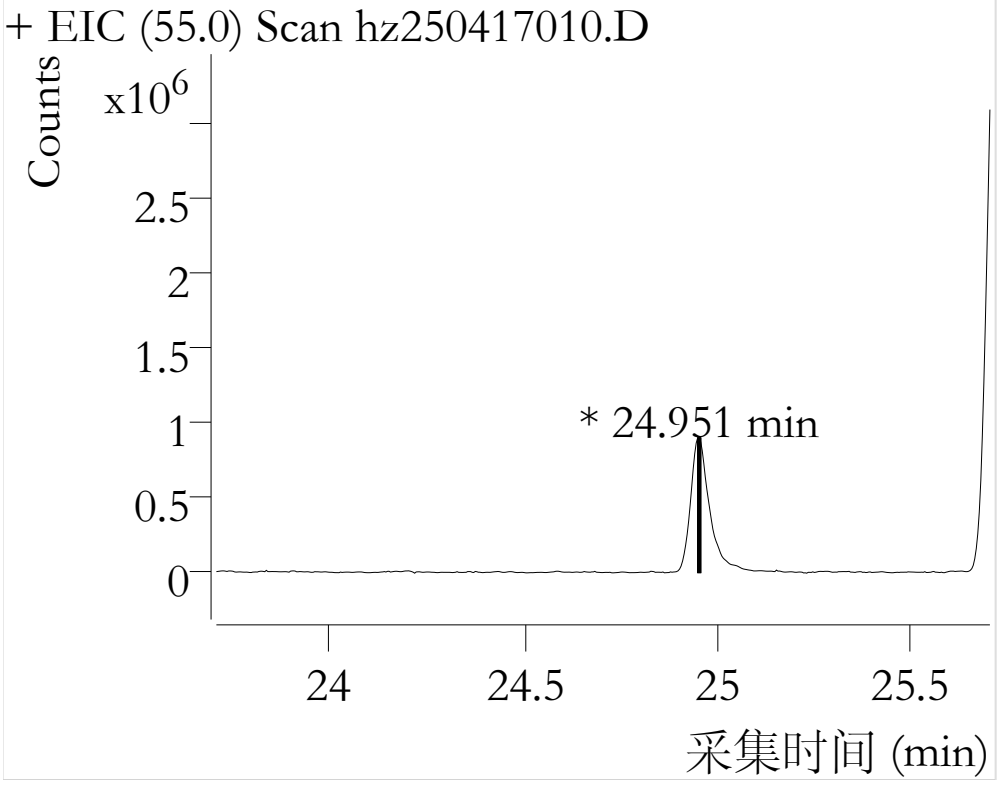

55.0, 208.0, 166.0

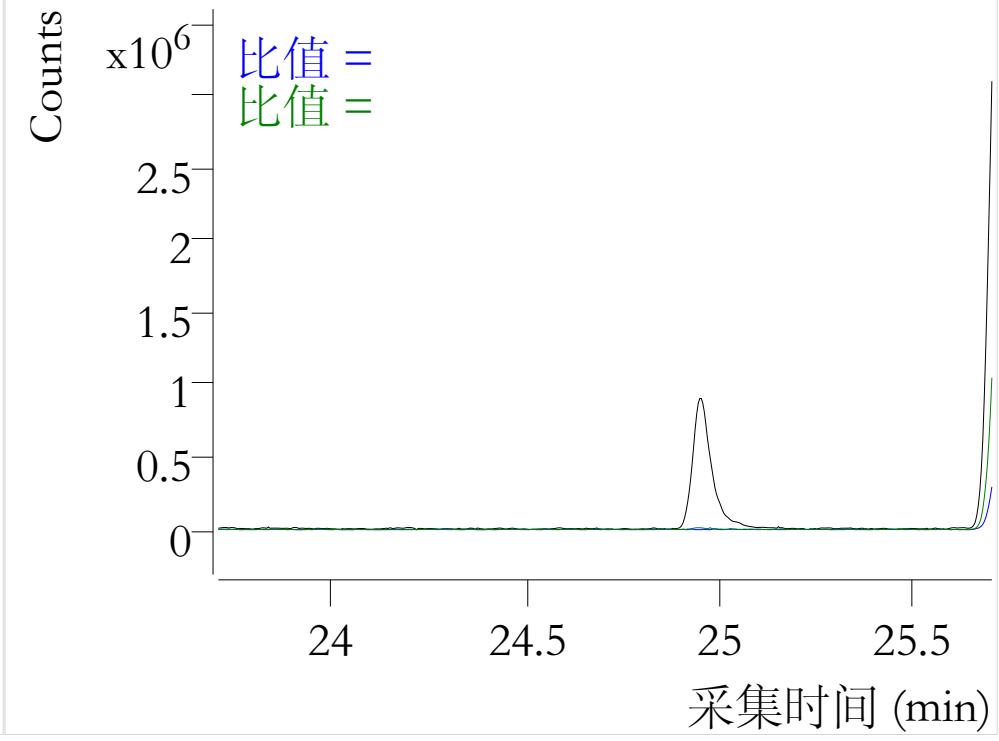

+ Scan (24.951-24.951 min, 1 scans) hz250417010.D

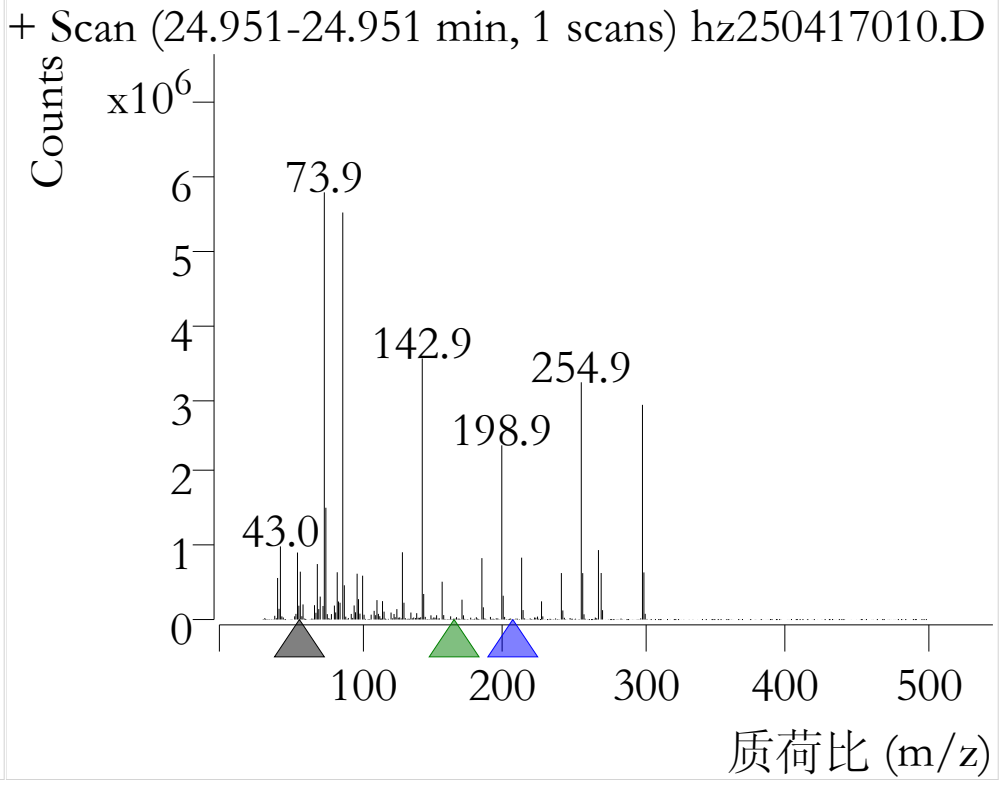

C18:0

+ EIC (86.9) Scan hz250417010.D

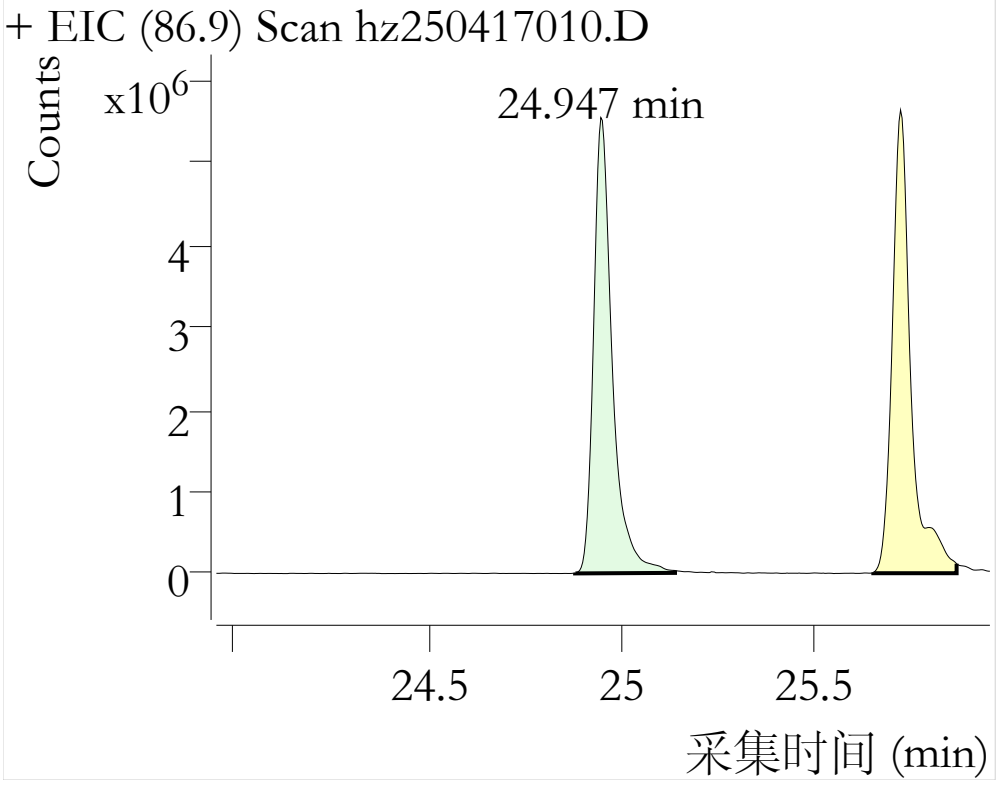

86.9, 142.9, 198.9

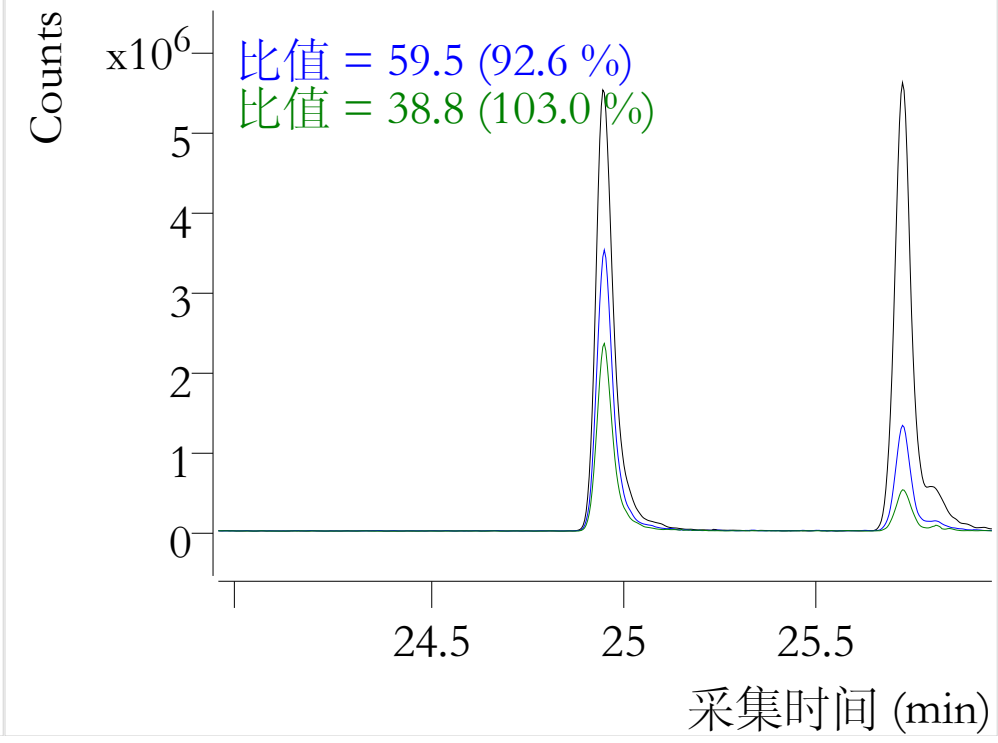

+ Scan (24.881-25.138 min, 67 scans) hz250417010.D

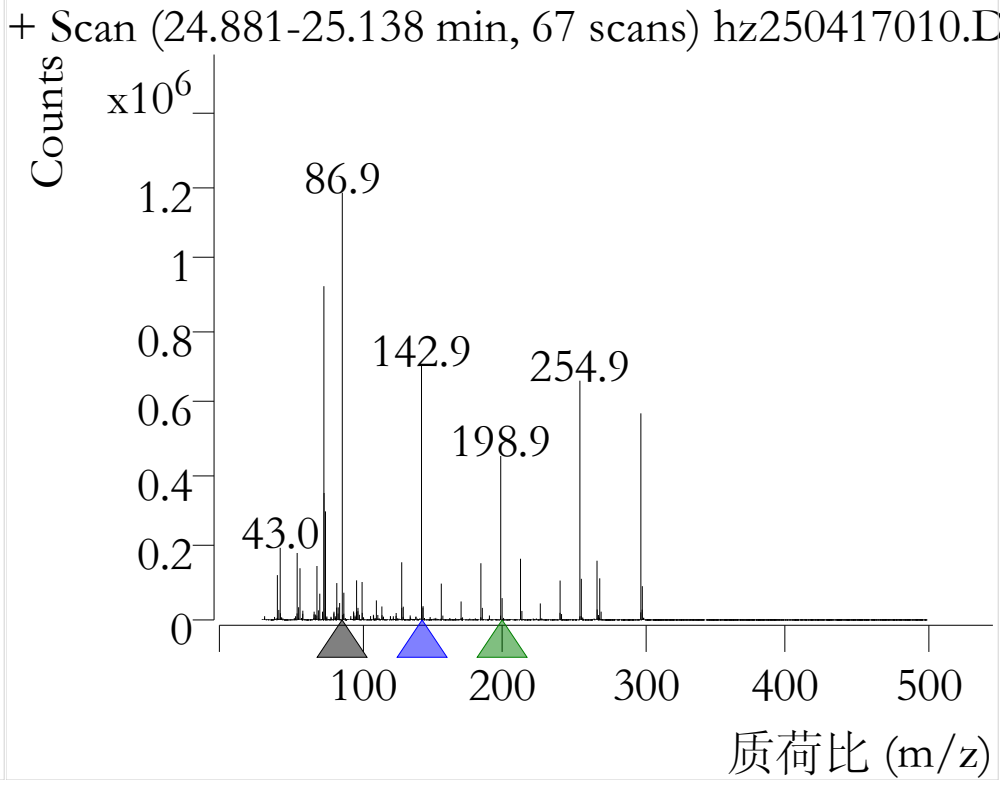

C18:1n9t

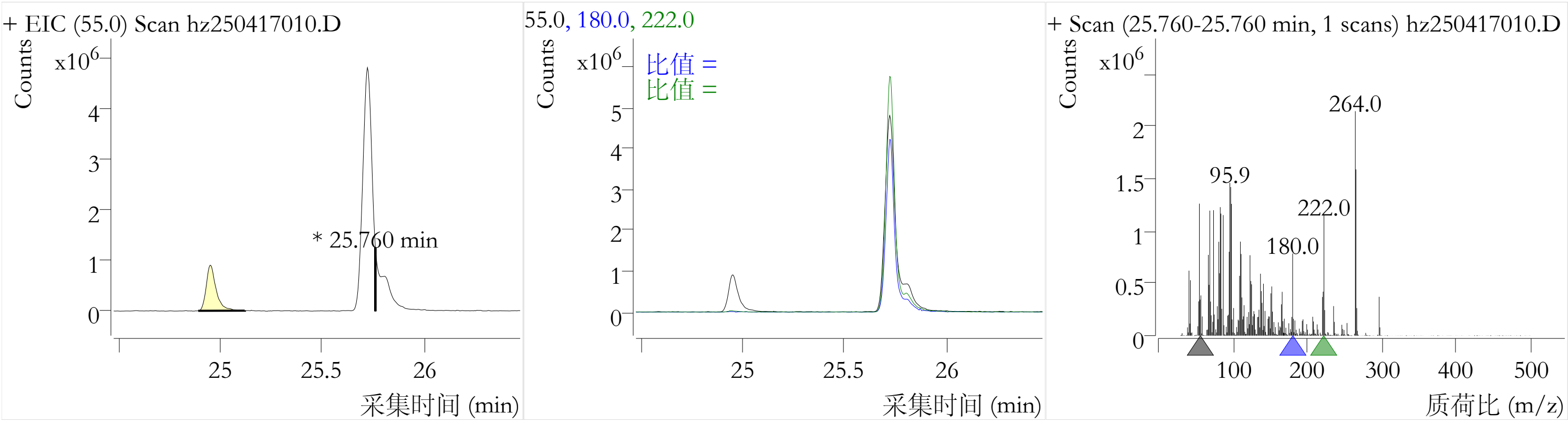

C18:1n9c

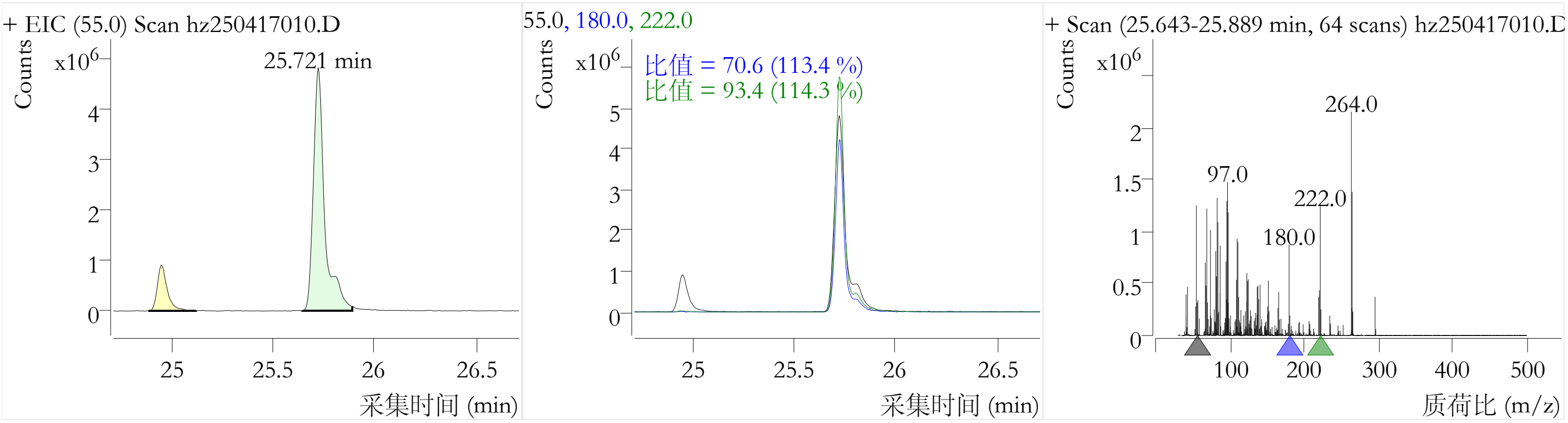

C18:2n6t

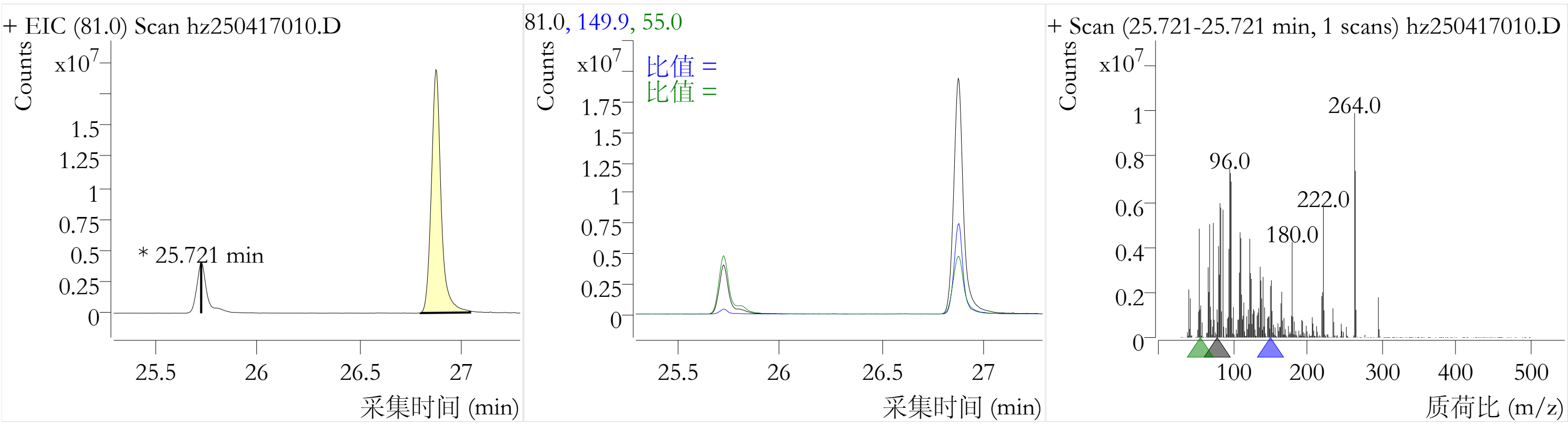

C18:2n6c

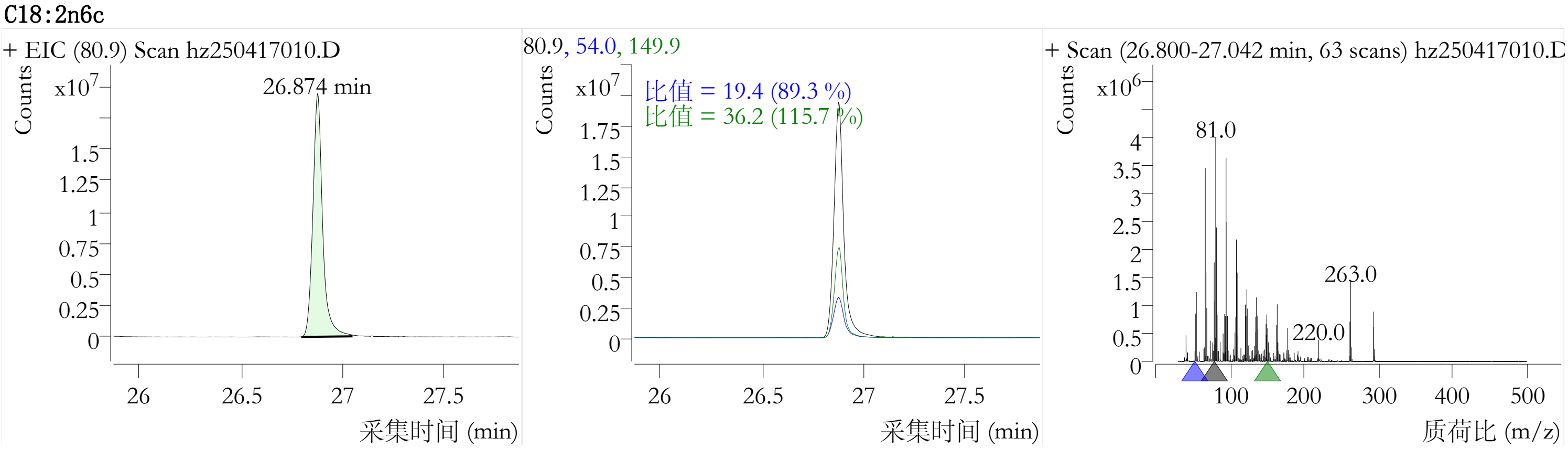

C20:0

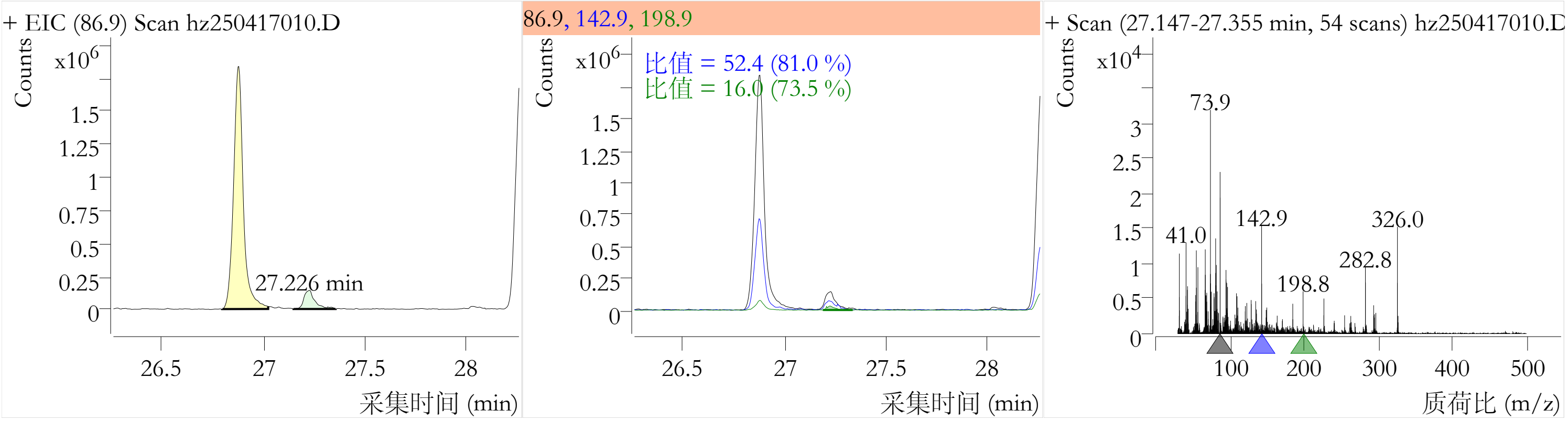

C18:3n6

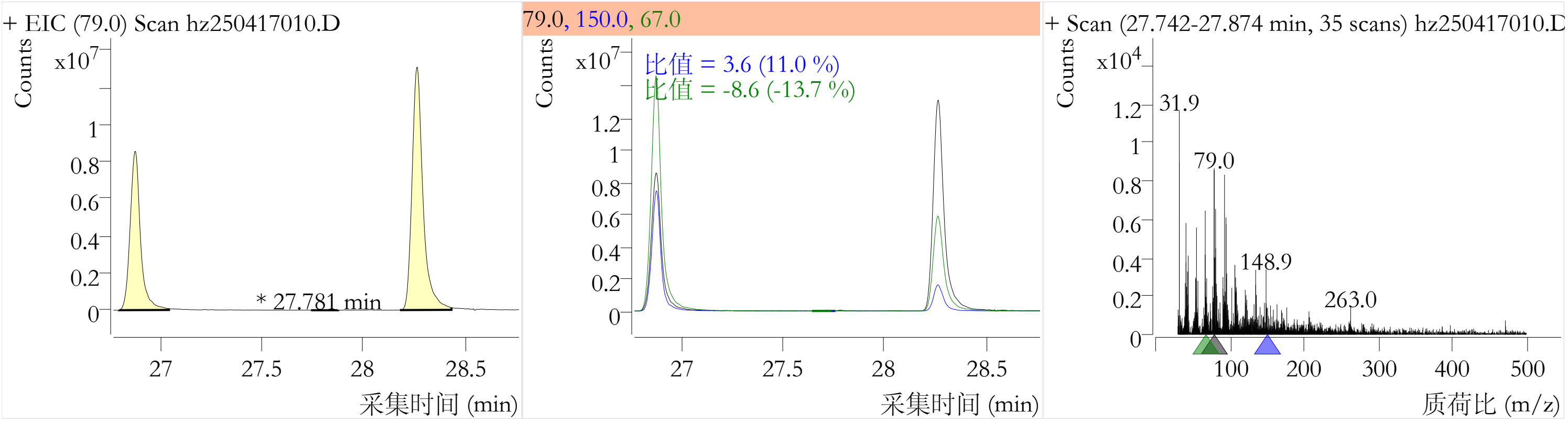

C20:1

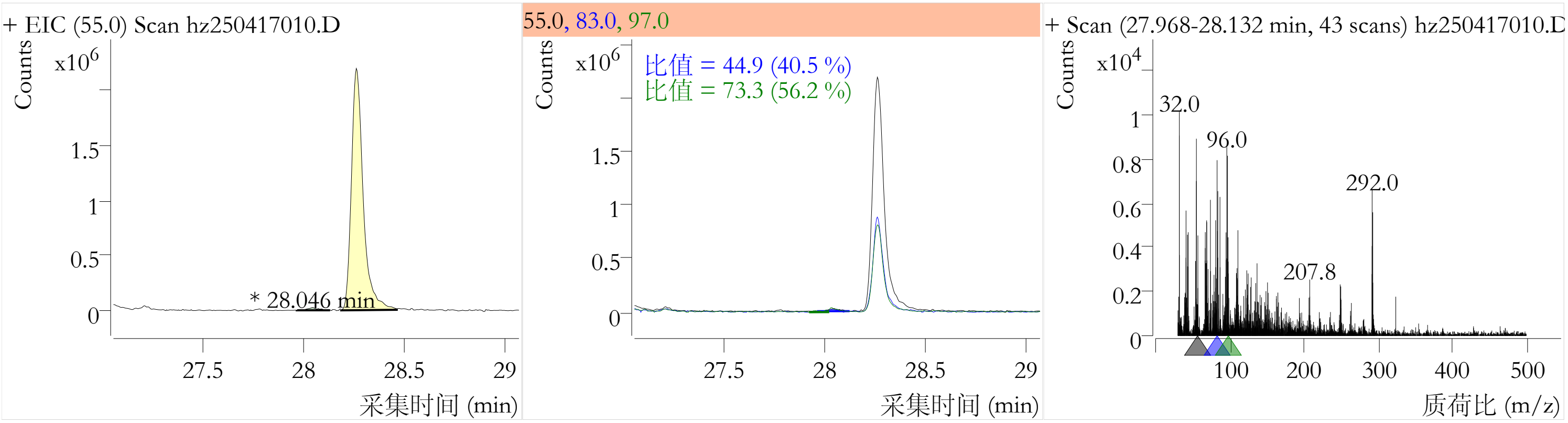

C18:3n3

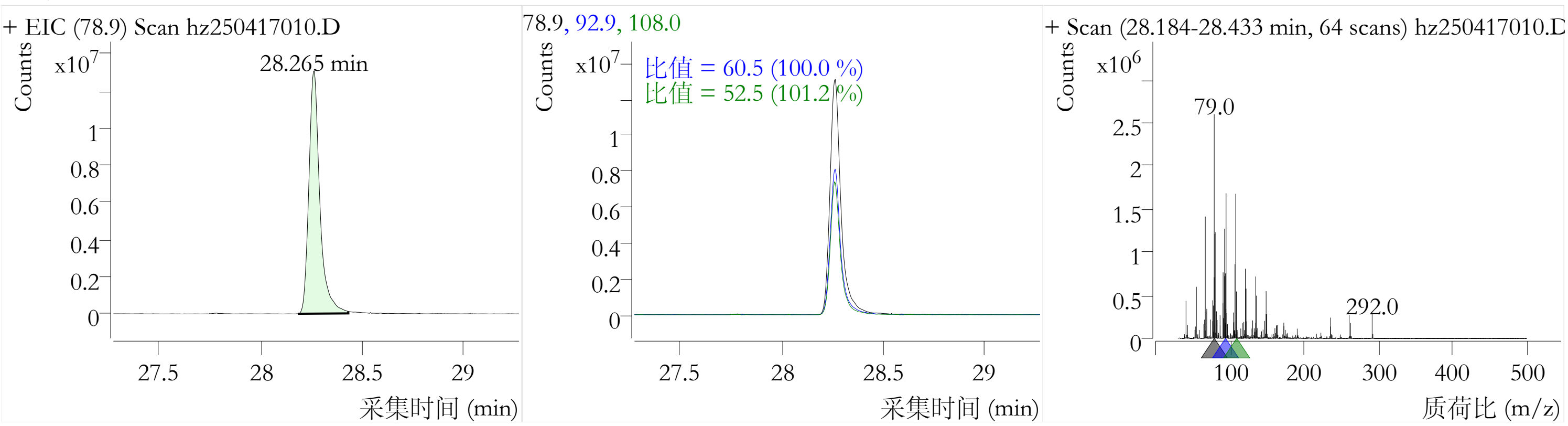

C21:0

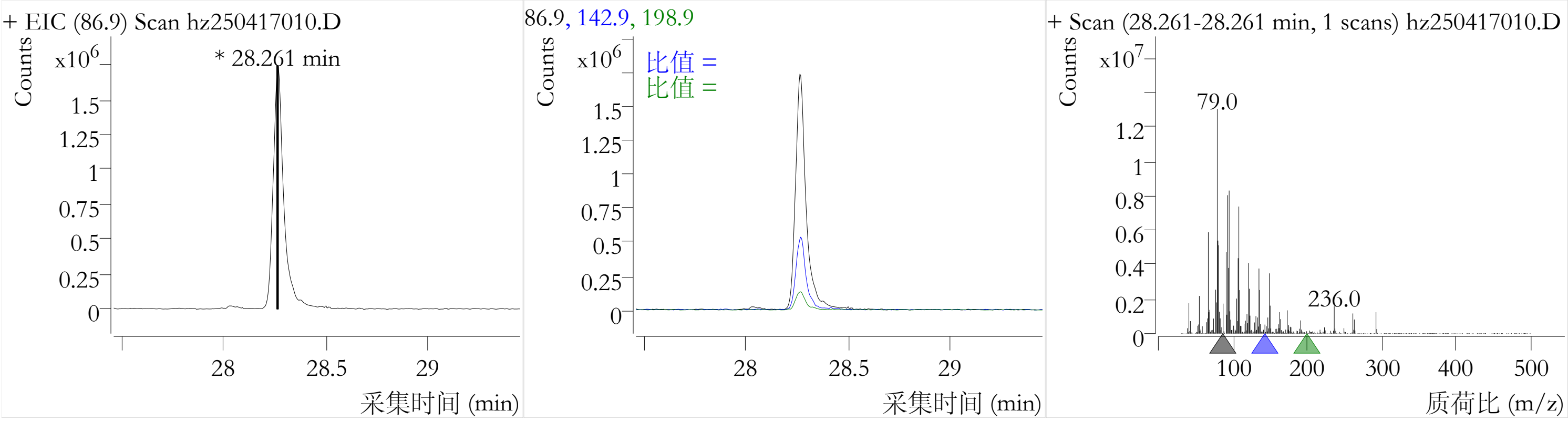

C20:2

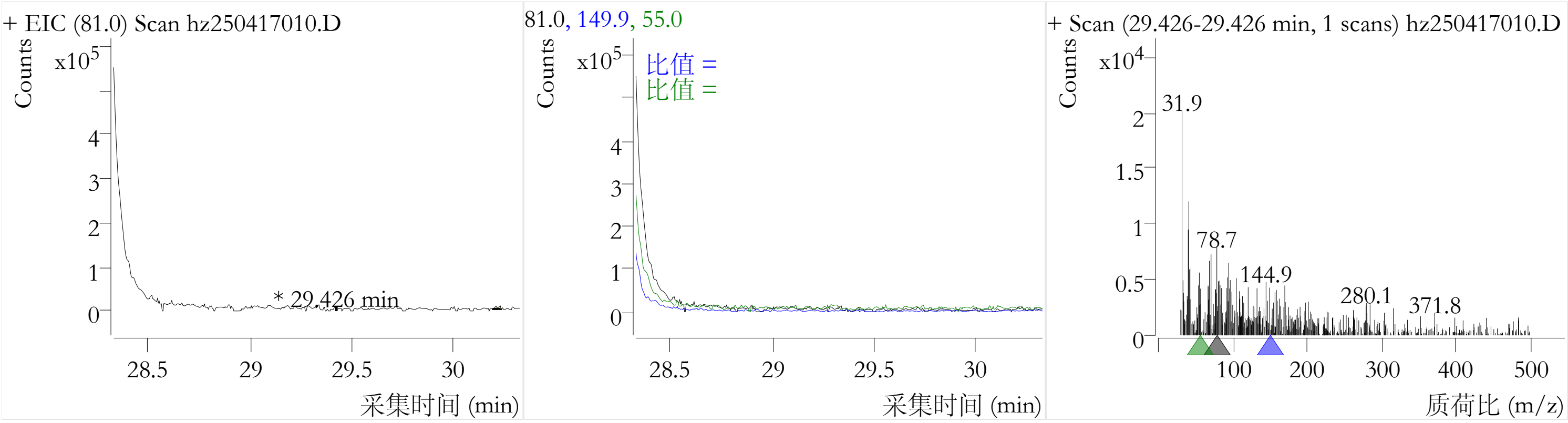

C22:0

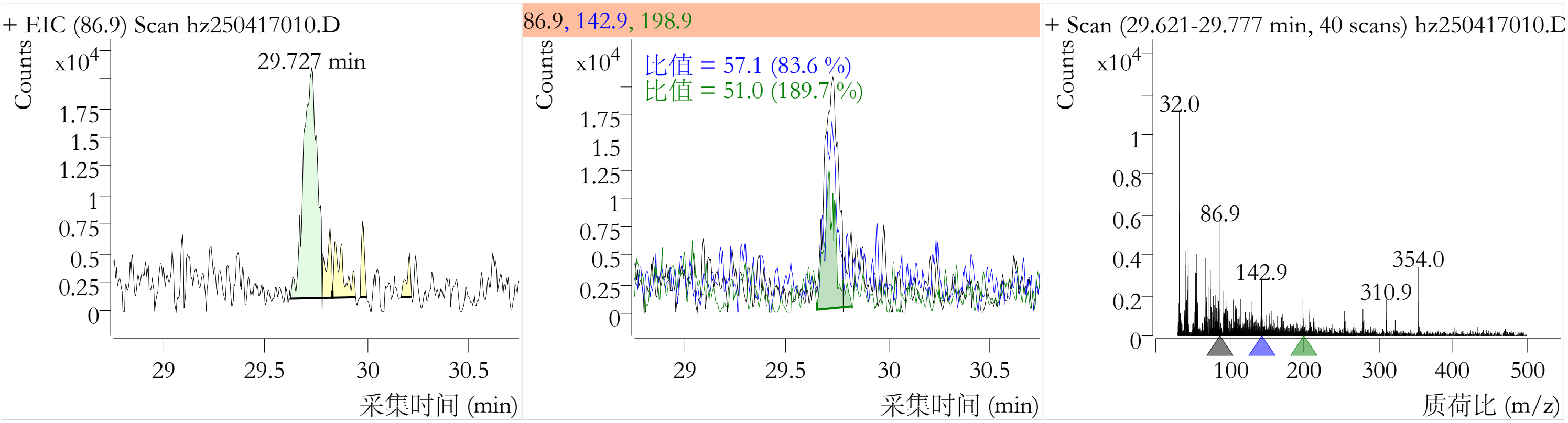

C20:3n6

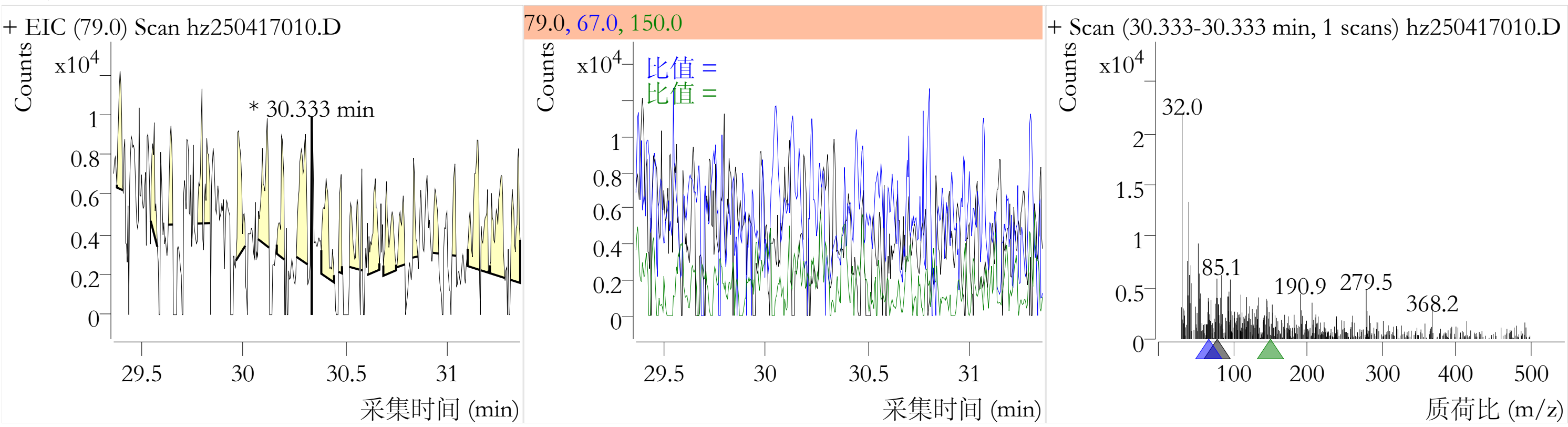

C22:1n9

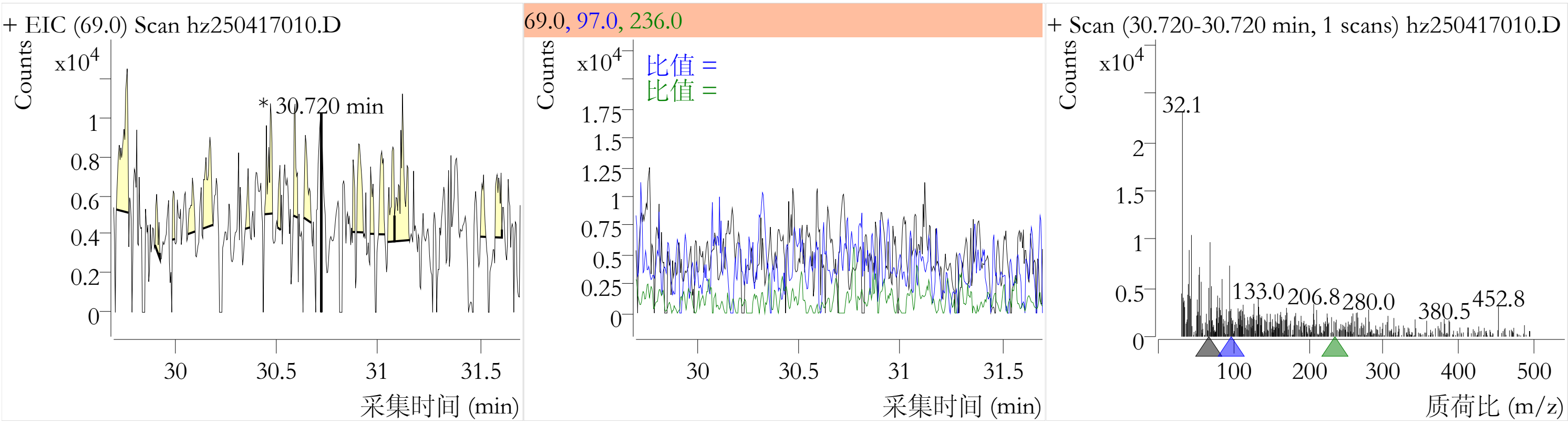

C20:3n3

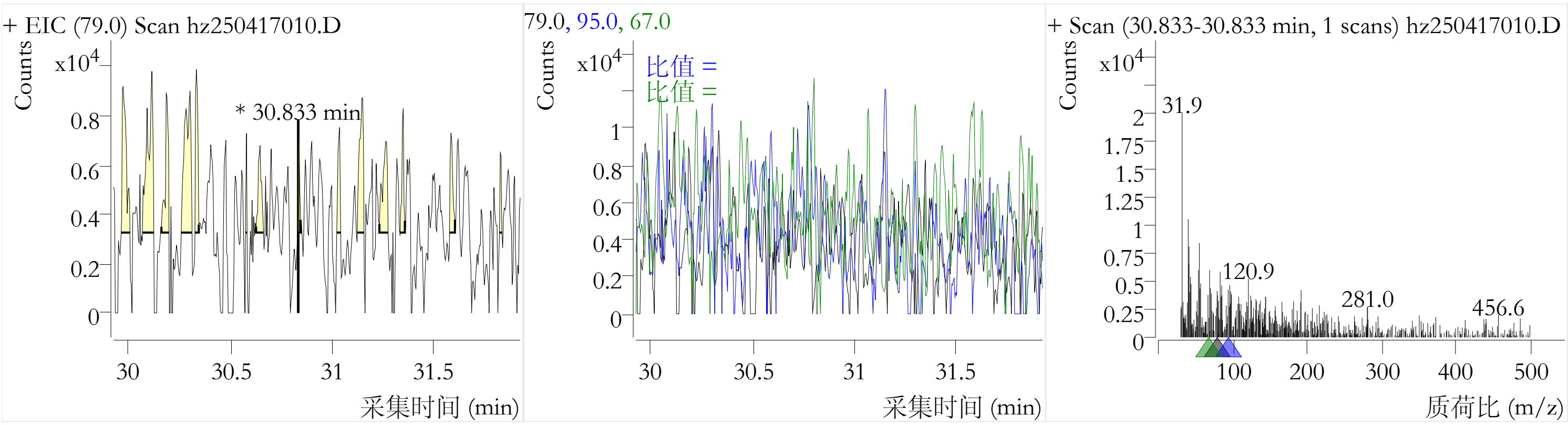

C23:0

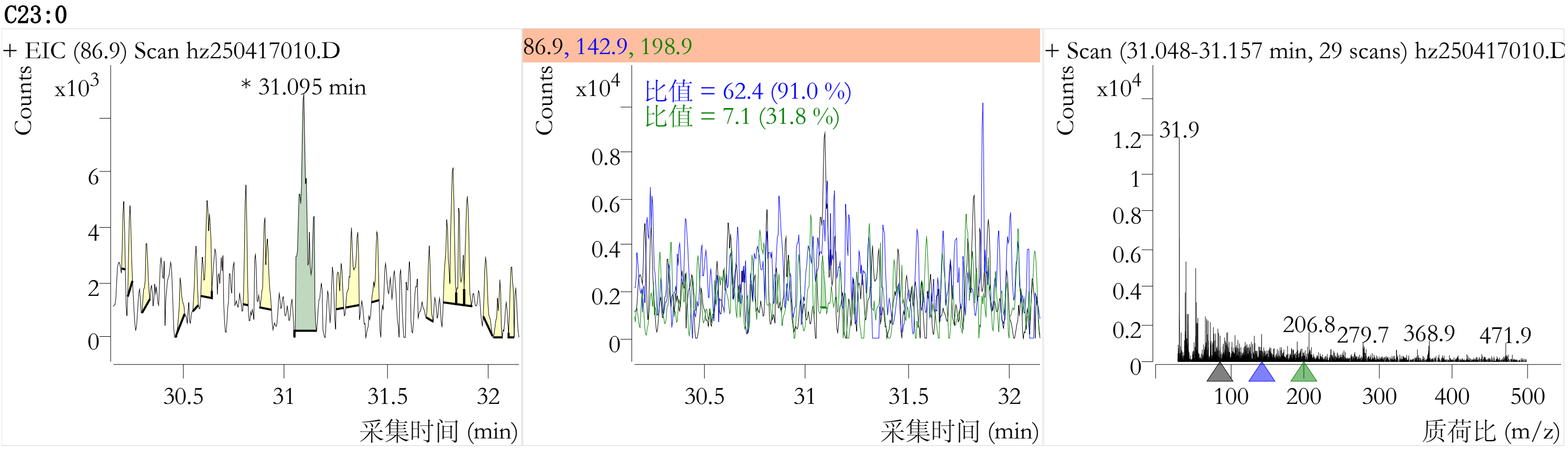

C20:4n6

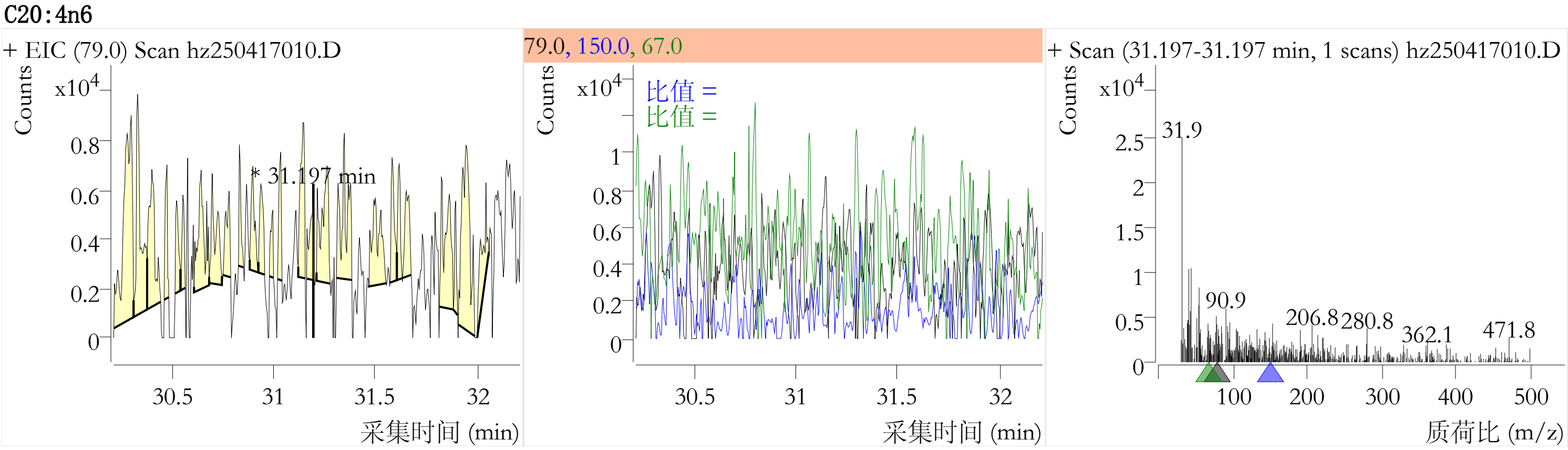

C22:2

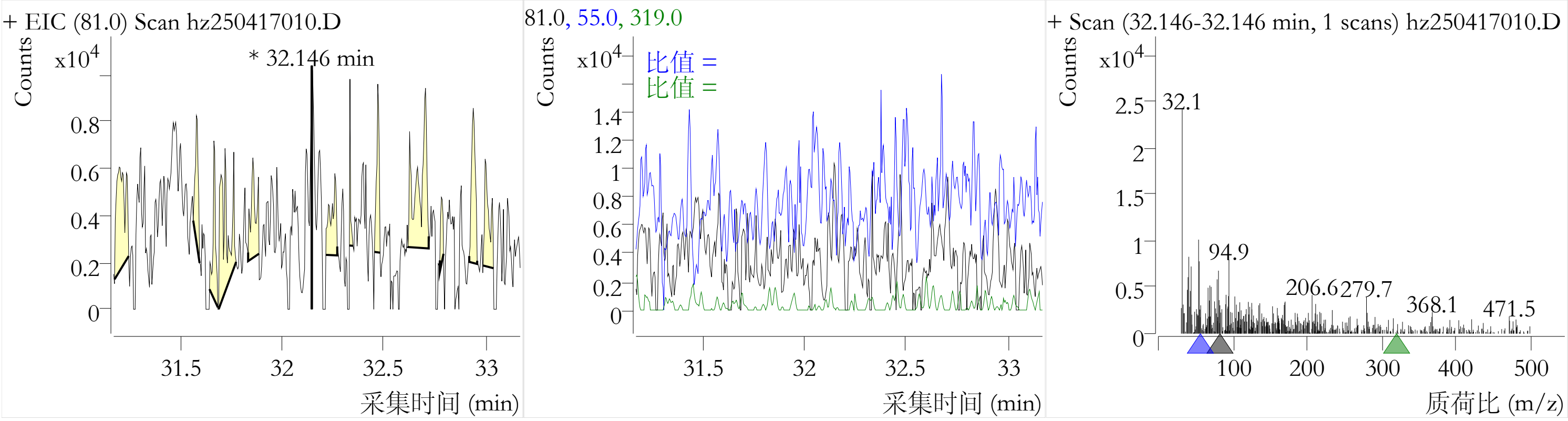

C24:0

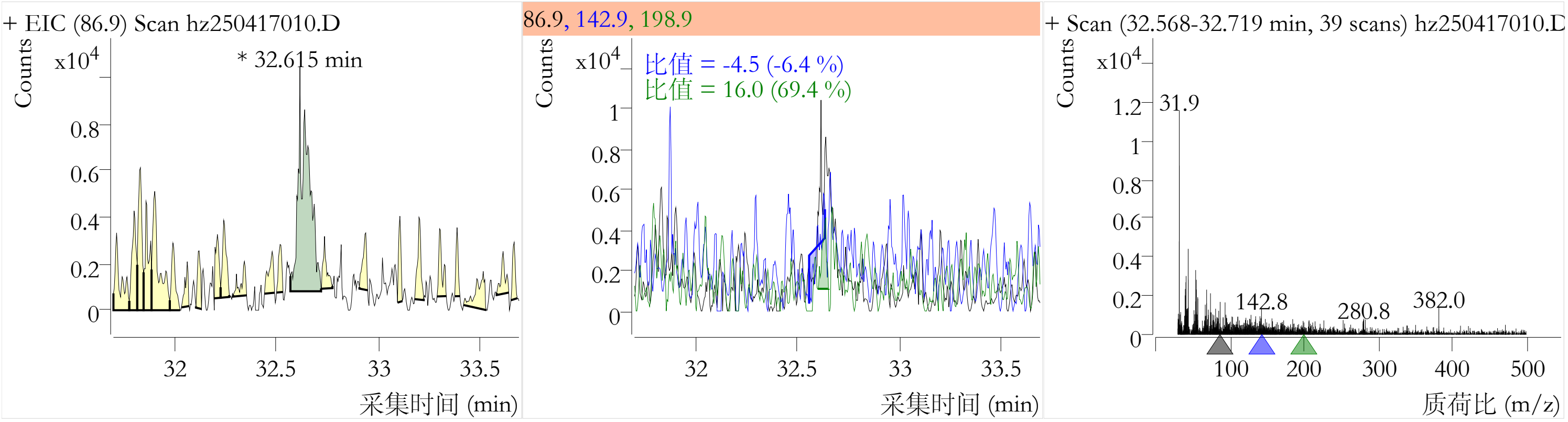

C20:5n3

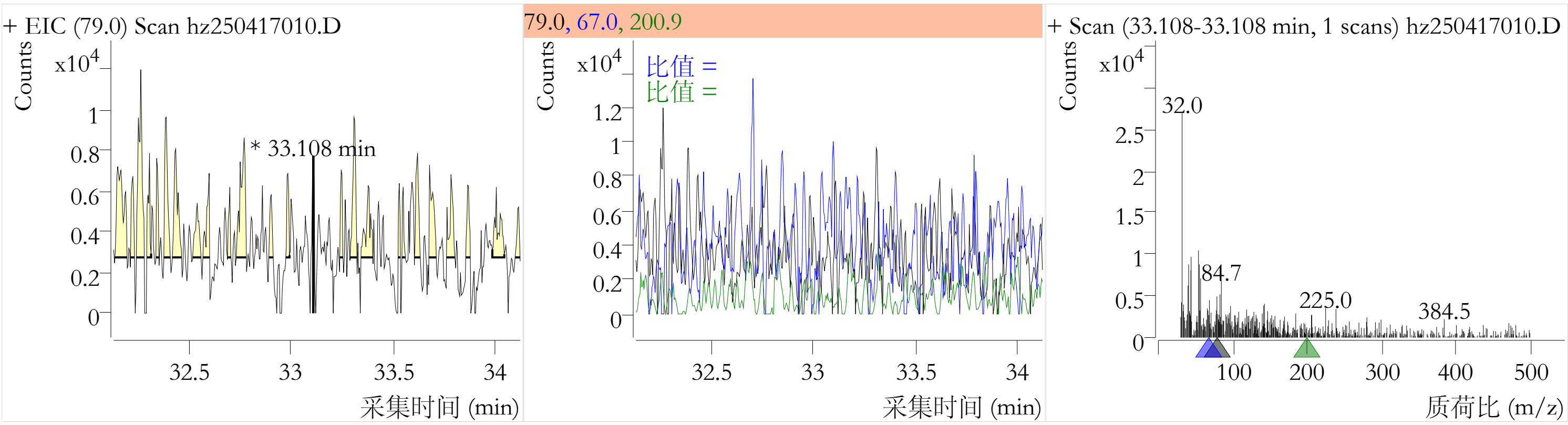

C24:1

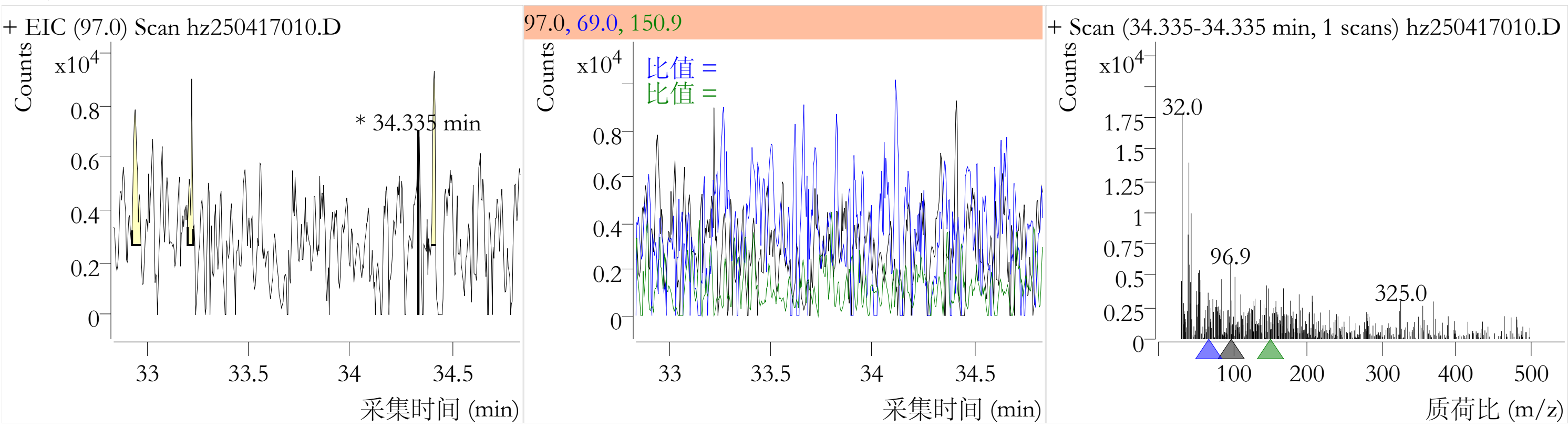

C22:6n3

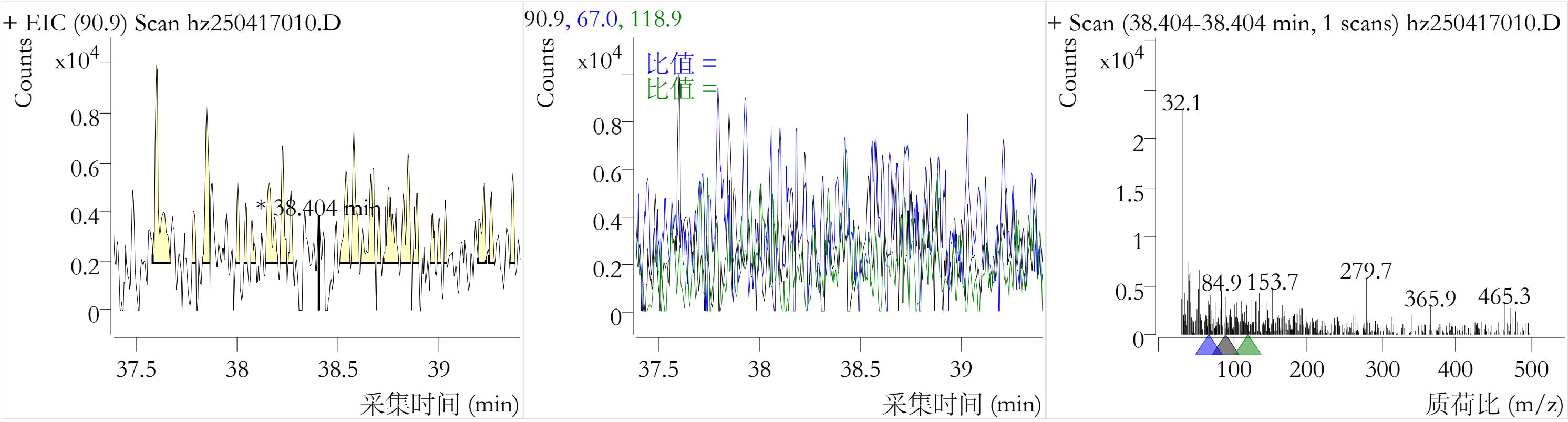
+ EIC (90.9) Scan hz250417010.D
Counts
x10^4
0.8
0.6
0.4
0.2
0
* 38.404 min
37.5
38
38.5
39
采集时间 (min)
90.9, 67.0, 118.9
比值 =
比值 =
采集时间 (min)
+ Scan (38.404-38.404 min, 1 scans) hz250417010.D
32.1
84.9
153.7
279.7
365.9
465.3
2
1.5
1
0.5
100
200
300
400
500
质荷比 (m/z)

定量分析报告（根据样品）

Agilent Technologies

**采集日期时间**	2025/5/13 14:25:27	**数据文件**	hz250417011.D
**类型**	样品	**名称**	hz250417011
**稀释**	1	**采集方法文件**	FAME-TEST-SCAN

样品色谱图

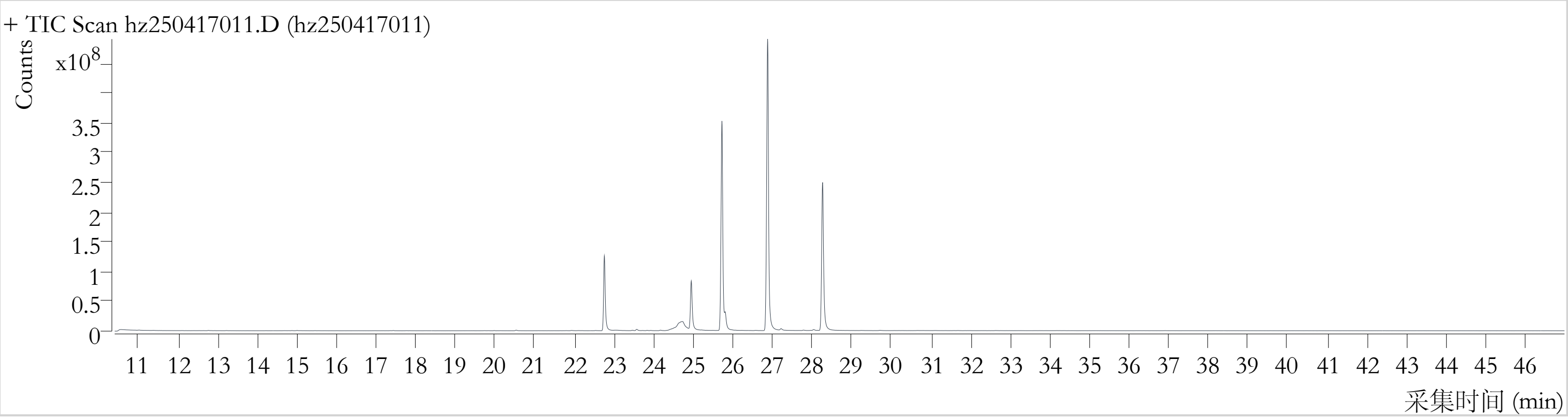

名称	RT	离子对	响应	峰高	最终浓度 单位	比值
C4:0	10.541	74.0	0	3387.86	ND ug/ml	
C8:0	13.824	74.0	0	4794.91	ND ug/ml	
C10:0	16.036	74.0	0	9022.85	ND ug/ml	
C11:0	17.142	74.0	0	9505.52	ND ug/ml	
C12:0	18.260	74.0	47102	18156.05	0.1102 ug/ml	58.1
C13:0	19.421	74.0	0	3582.92	ND ug/ml	
C14:0	20.535	74.0	526998	162109.89	1.3085 ug/ml	58.3
C14:1	21.524	55.0	0	11522.16	ND ug/ml	
C15:0	21.641	74.0	35073	18189.67	0.0906 ug/ml	26.1
C15:1	22.759	55.0	0	2189757.75	ND ug/ml	
C16:0	22.759	87.0	51918220	17368171.86	144.4967 ug/ml	57.1
C16:1	23.576	55.0	255193	81612.42	3.4542 ug/ml	63.1
C17:0	23.842	74.0	196690	60251.17	0.5724 ug/ml	49.9
C17:1	24.952	55.0	0	1448516.00	ND ug/ml	
C18:0	24.952	86.9	34110880	10376505.53	104.3571 ug/ml	62.4
C18:1n9t	25.726	55.0	0	7449509.50	ND ug/ml	
C18:1n9c	25.726	55.0	25527505	7435319.75	367.2588 ug/ml	110.8
C18:2n6t	25.726	81.0	0	6790719.50	ND ug/ml	
C18:2n6c	26.879	80.9	109154838	32027033.79	716.5888 ug/ml	34.1
C20:0	27.223	86.9	1068568	307650.20	3.2220 ug/ml	59.3
C18:3n6	27.786	79.0	223175	67305.93	1.3532 ug/ml	3158.6
C20:1	28.036	55.0	151664	39067.12	2.0308 ug/ml	-33.9
C18:3n3	28.266	78.9	79832430	21707473.55	376.3816 ug/ml	62.1
C21:0	28.266	86.9	0	3000578.00	ND ug/ml	
C20:2	29.239	81.0	0	18813.06	ND ug/ml	
C22:0	29.720	86.9	192838	46526.30	0.6382 ug/ml	55.4
C22:1n9	30.666	69.0	0	25225.65	ND ug/ml	
C23:0	31.100	86.9	67329	12317.96	0.2143 ug/ml	3.5
C20:4n6	31.190	79.0	0	9539.81	ND ug/ml	
C22:2		81.0			ND ug/ml	
C24:0	32.660	86.9	106515	22412.25	0.3914 ug/ml	35.1

C4:0

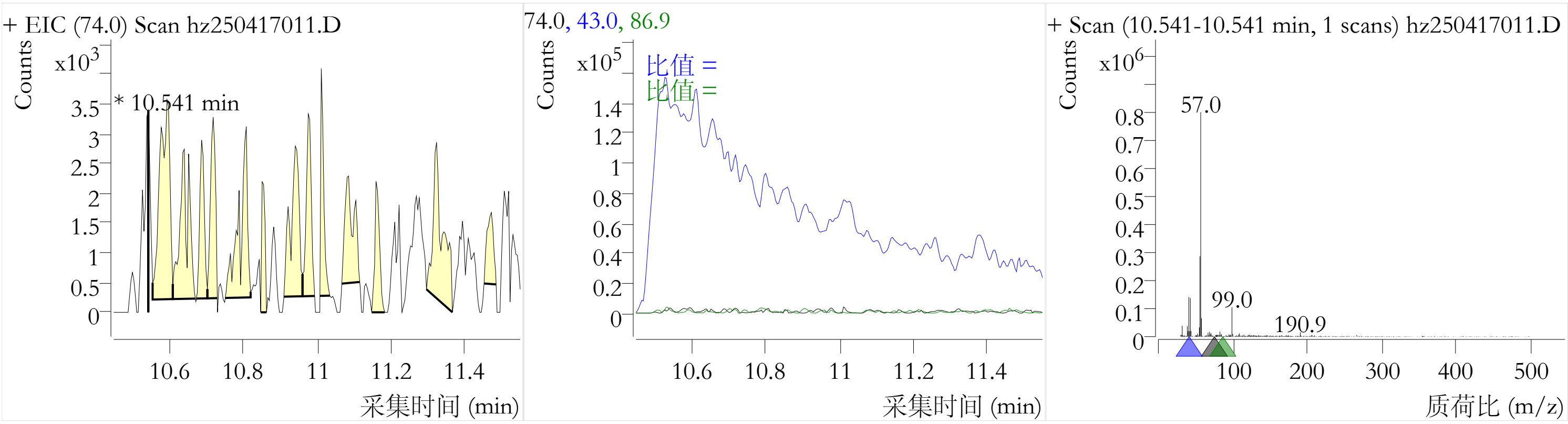

C6:0

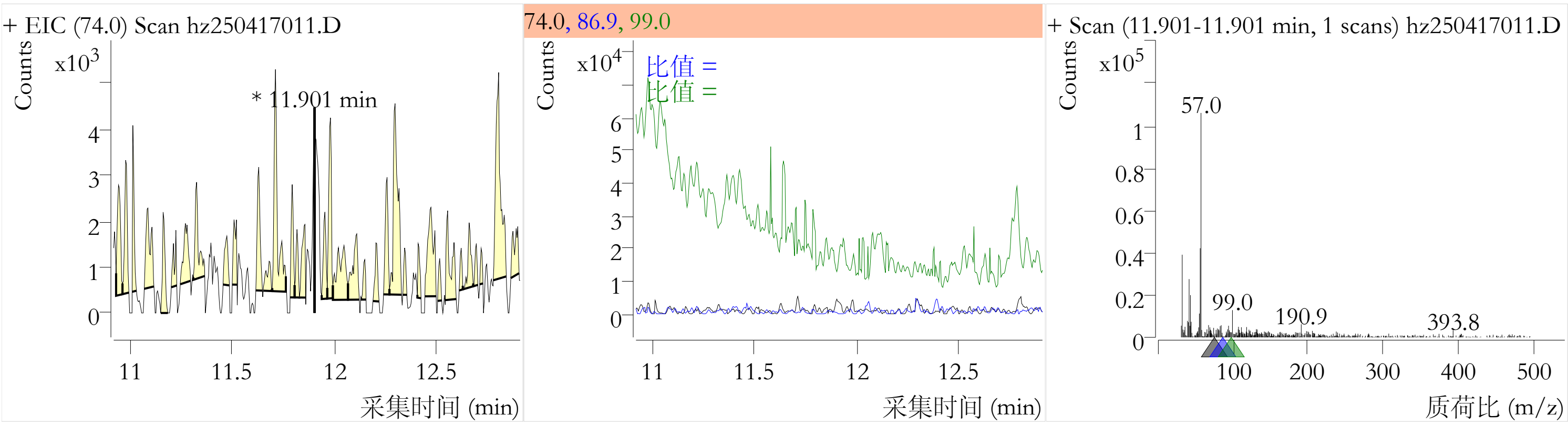

C8:0

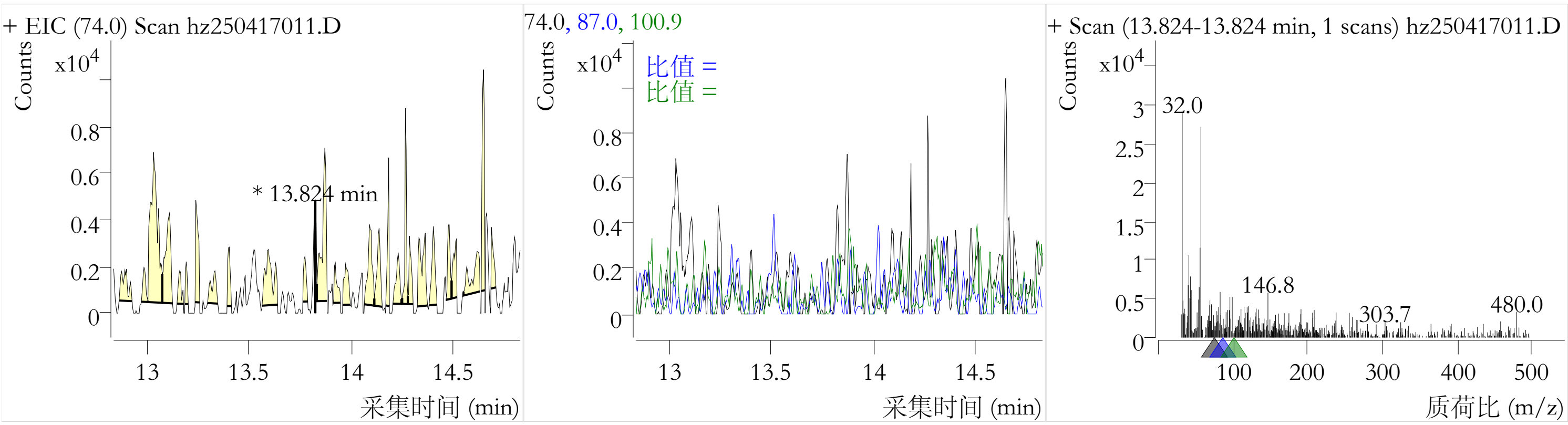

C10:0

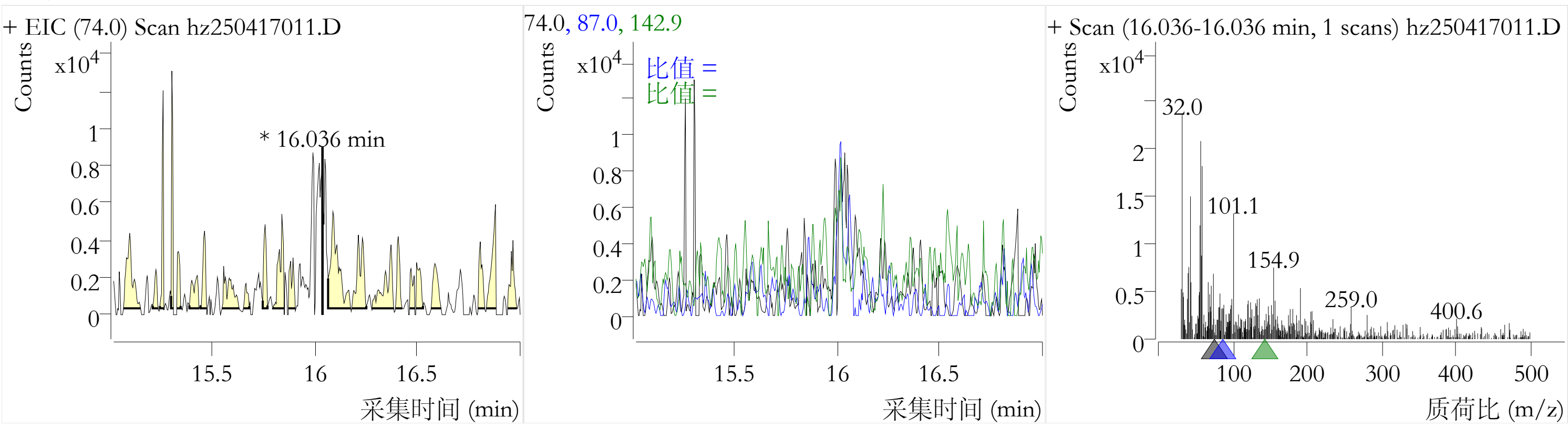

C11:0

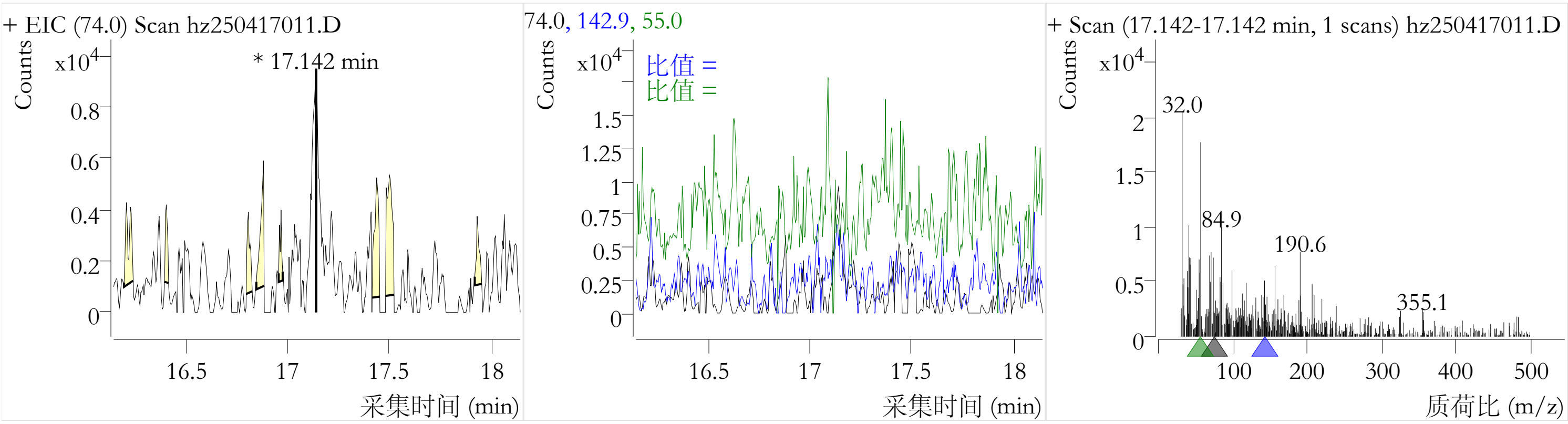

C12:0

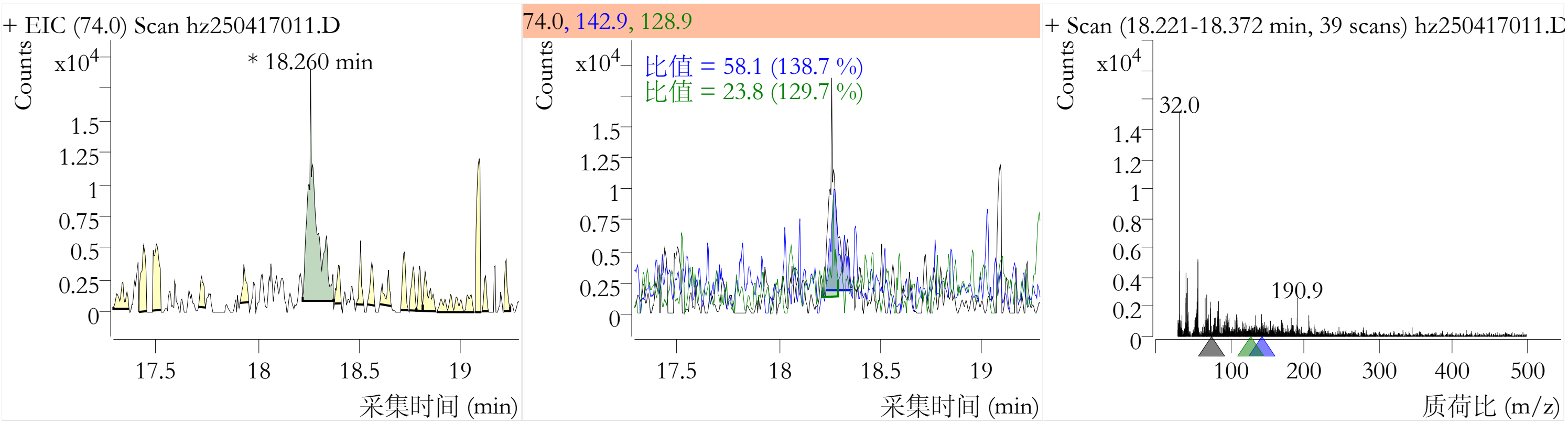

C13:0

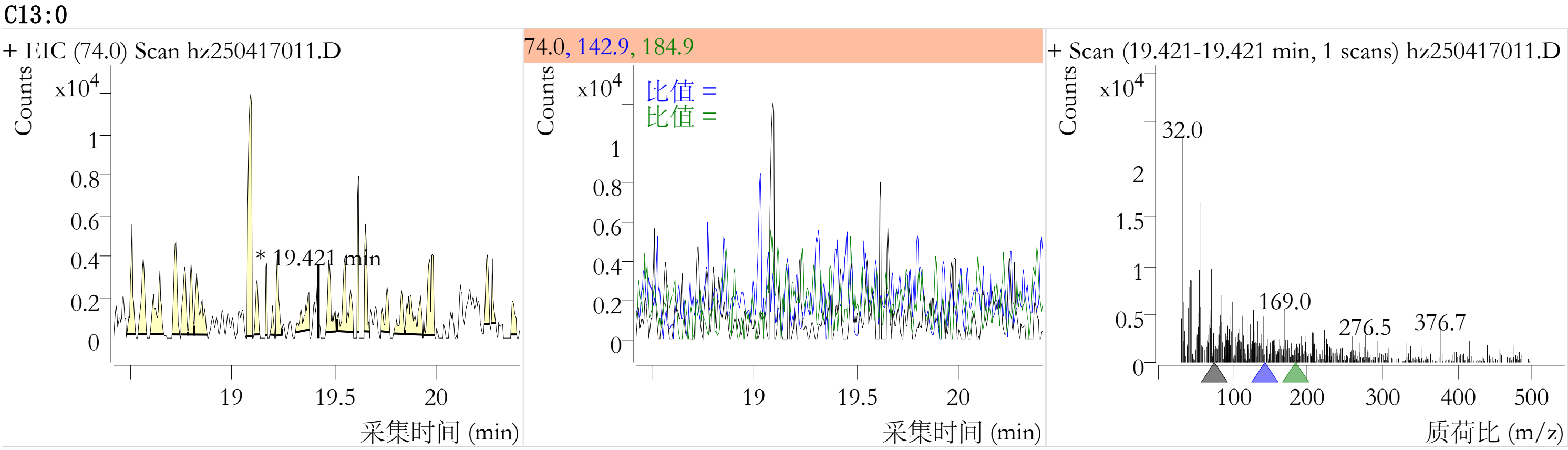

C14:0

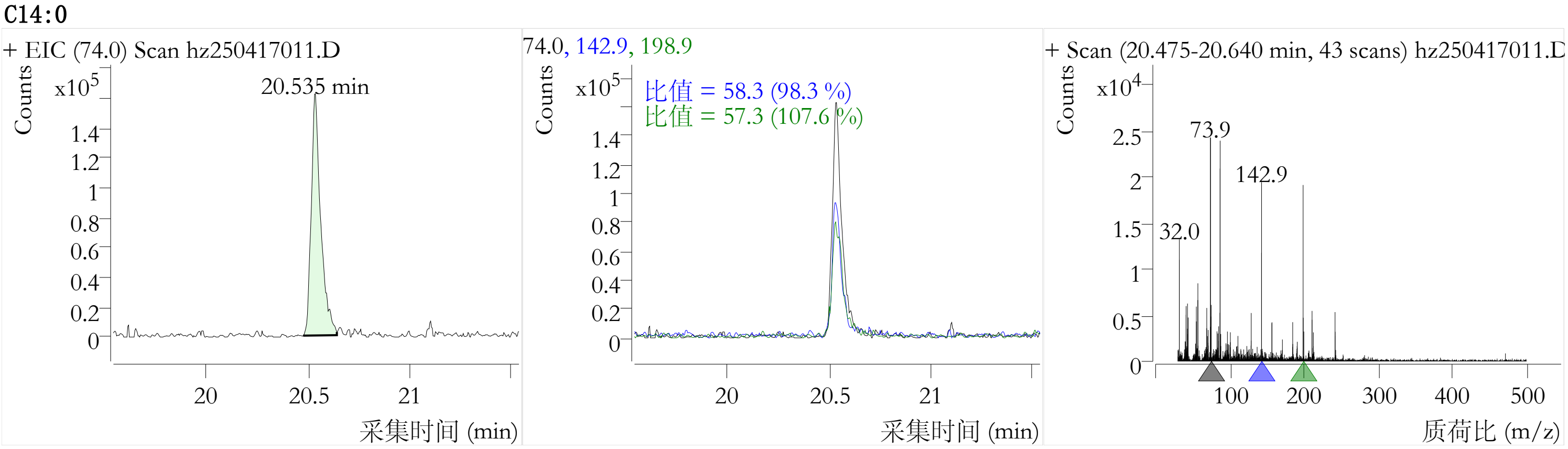

C14:1

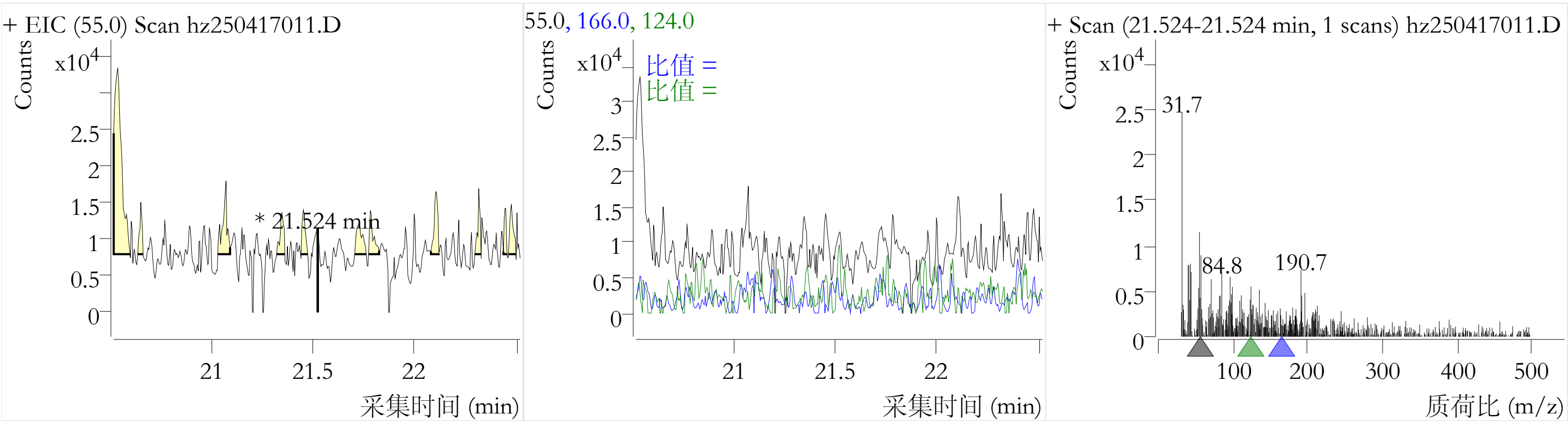

C15:0

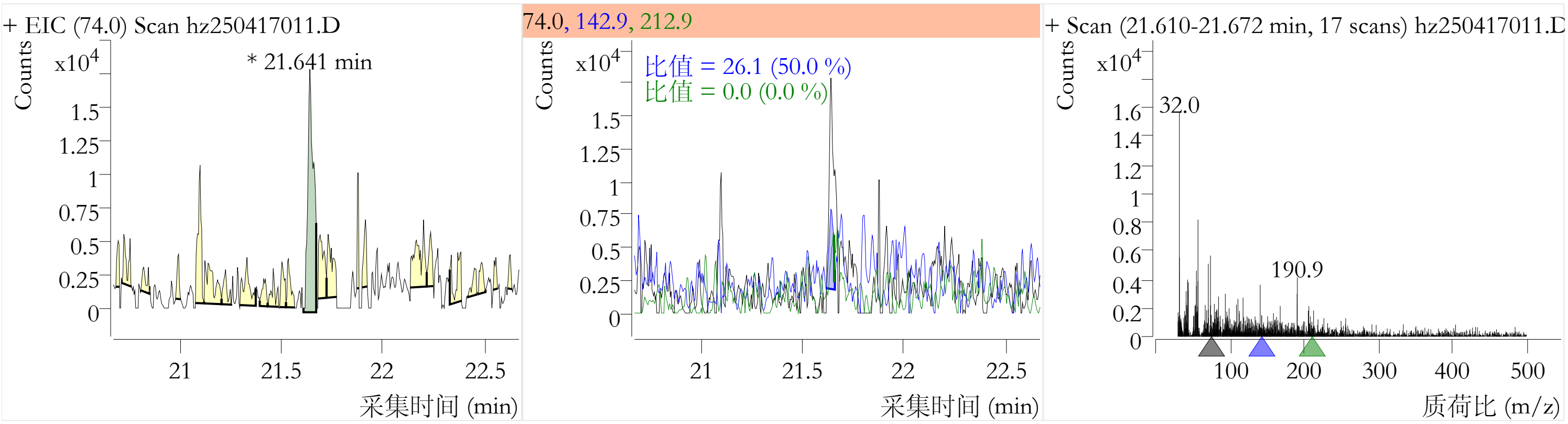

C15:1

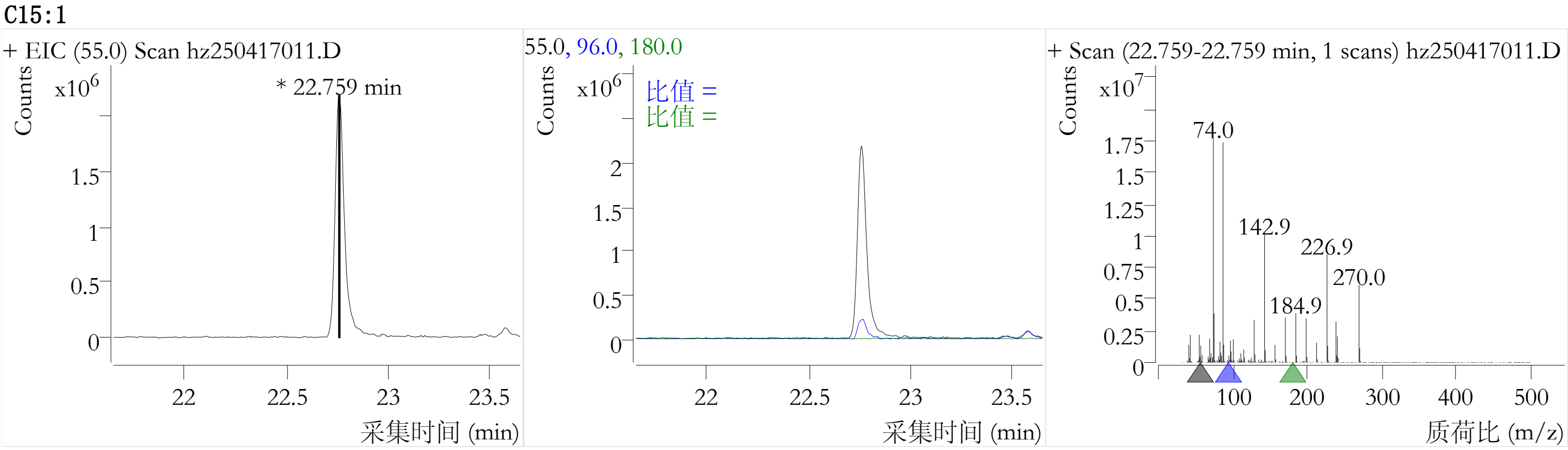

C16:0

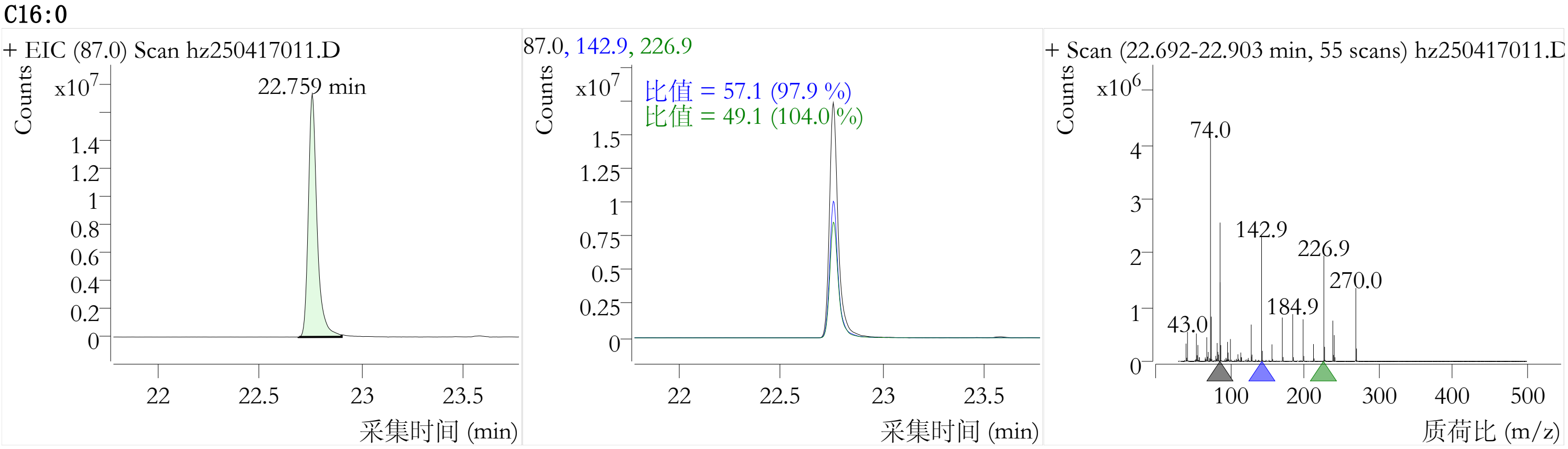

C16:1

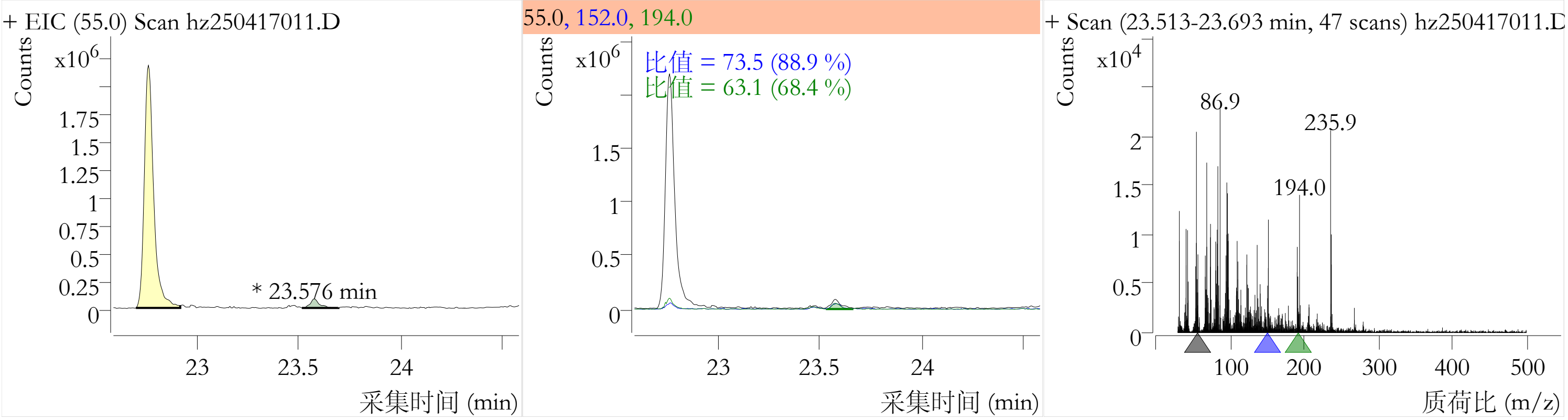

C17:0

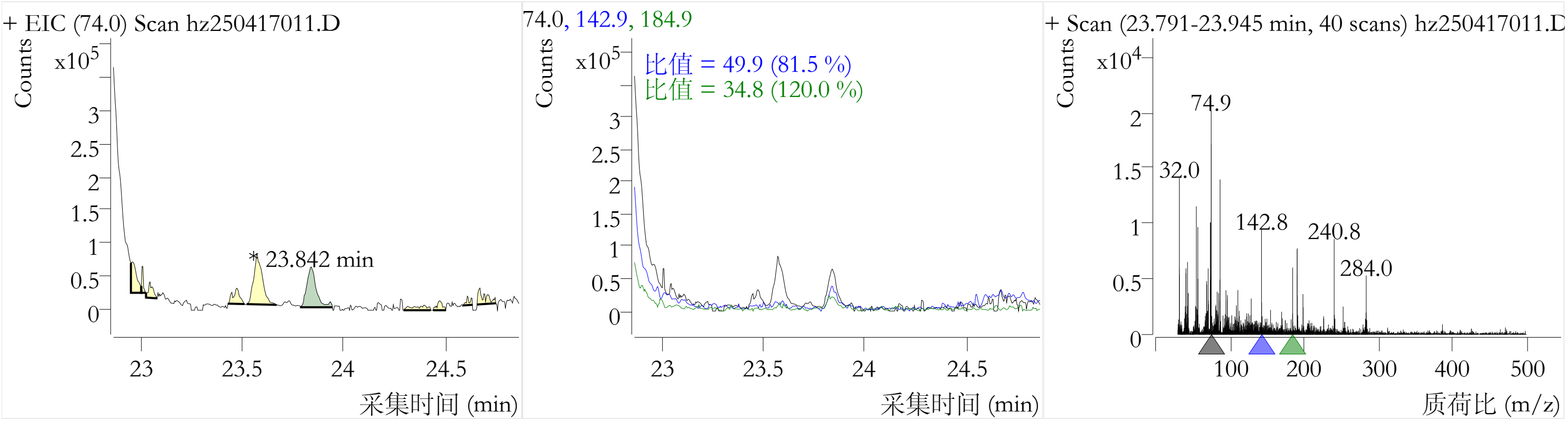

C17:1

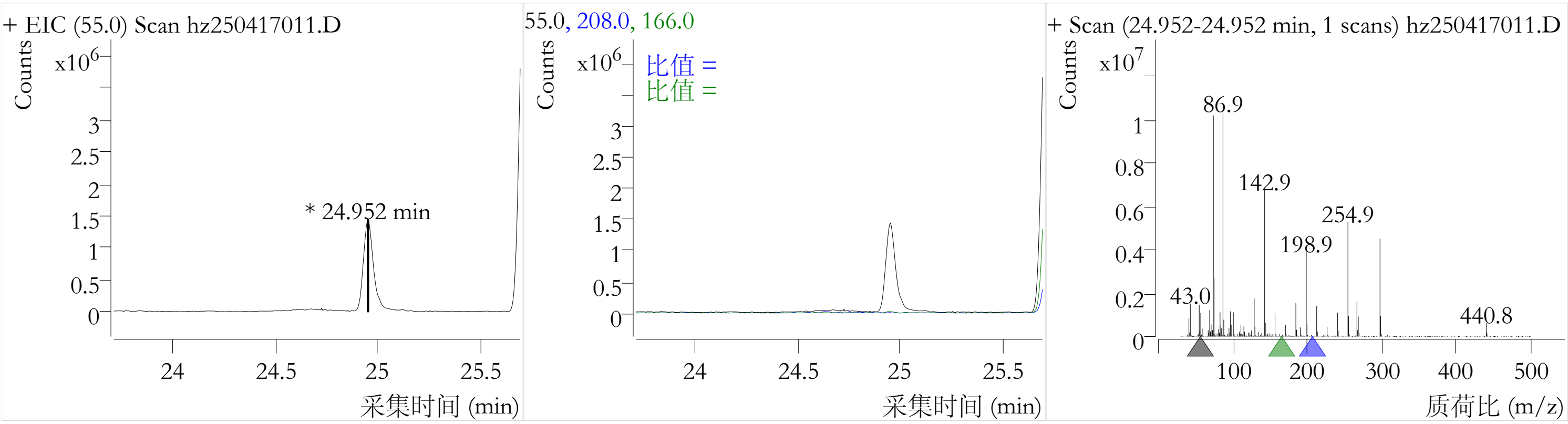

C18:0

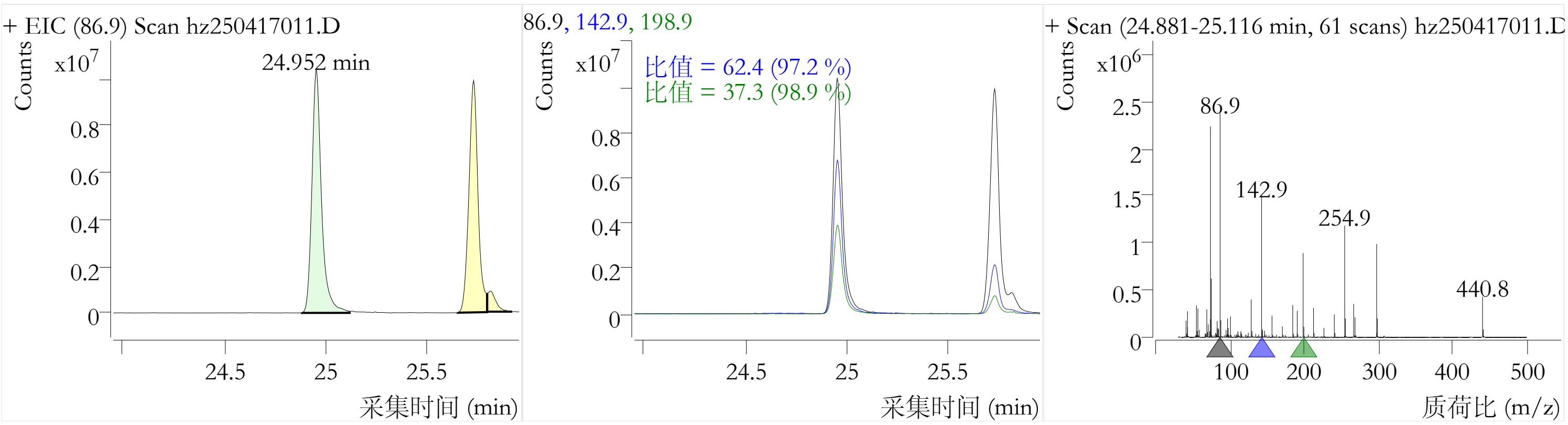

C18:1n9t

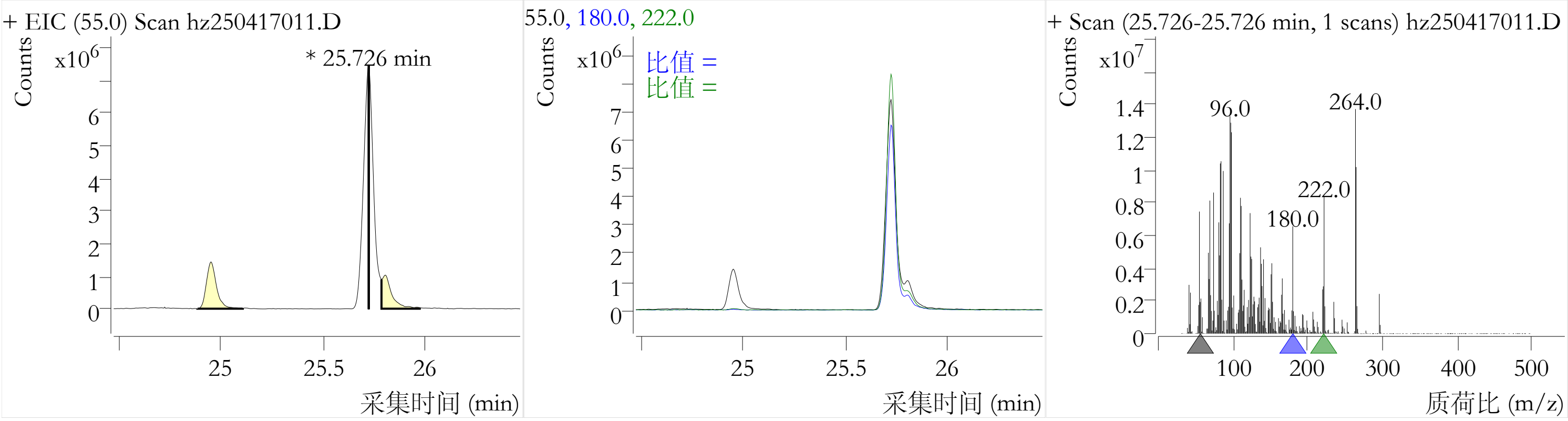

C18:1n9c

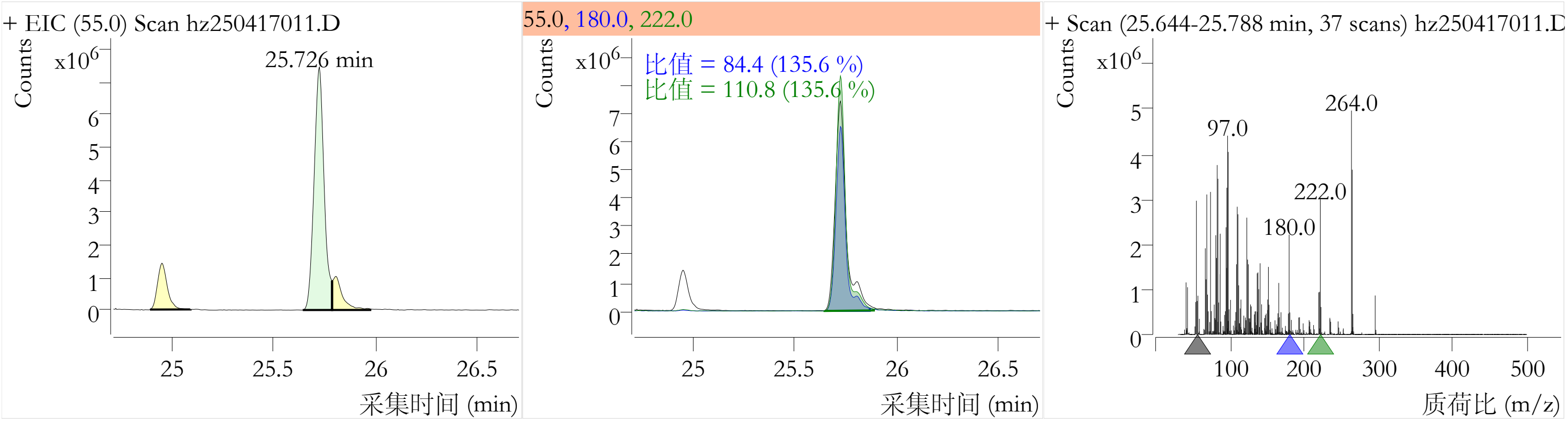

C18:2n6t

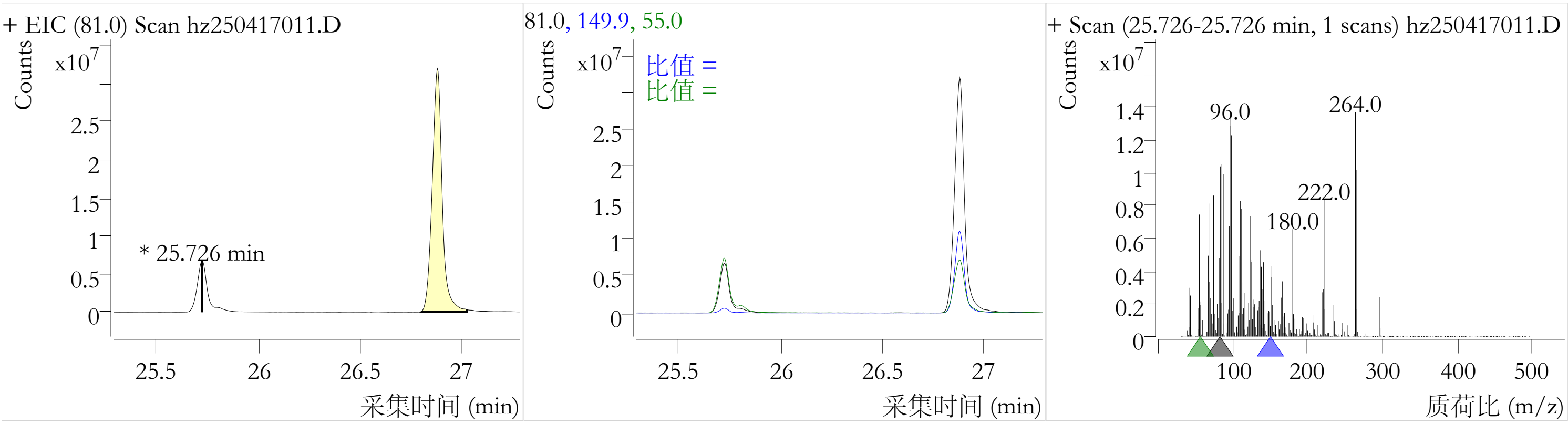

C18:2n6c

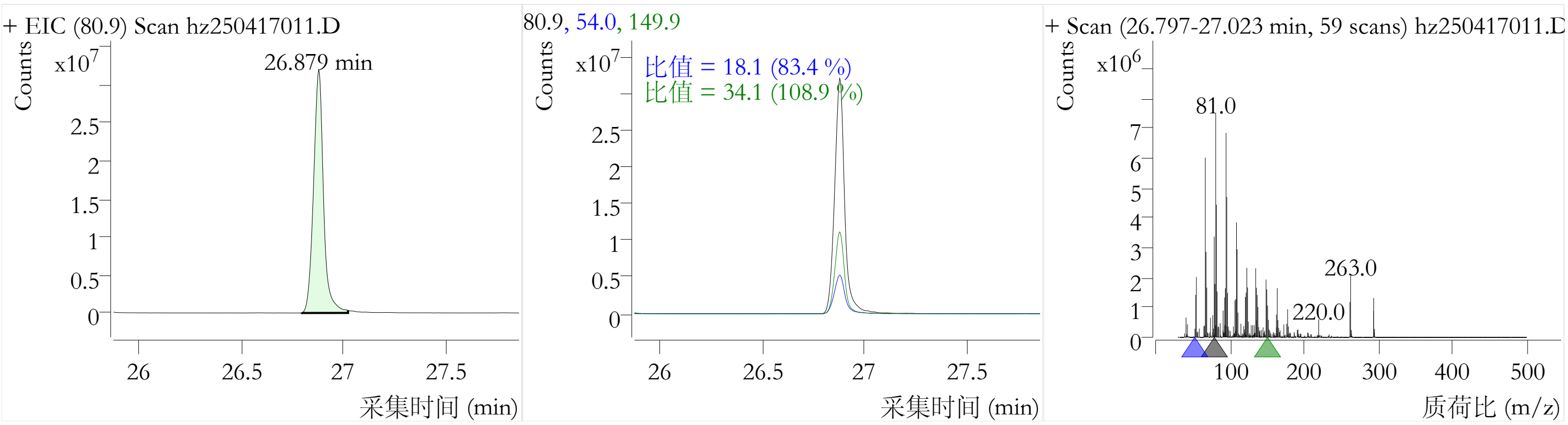

C20:0

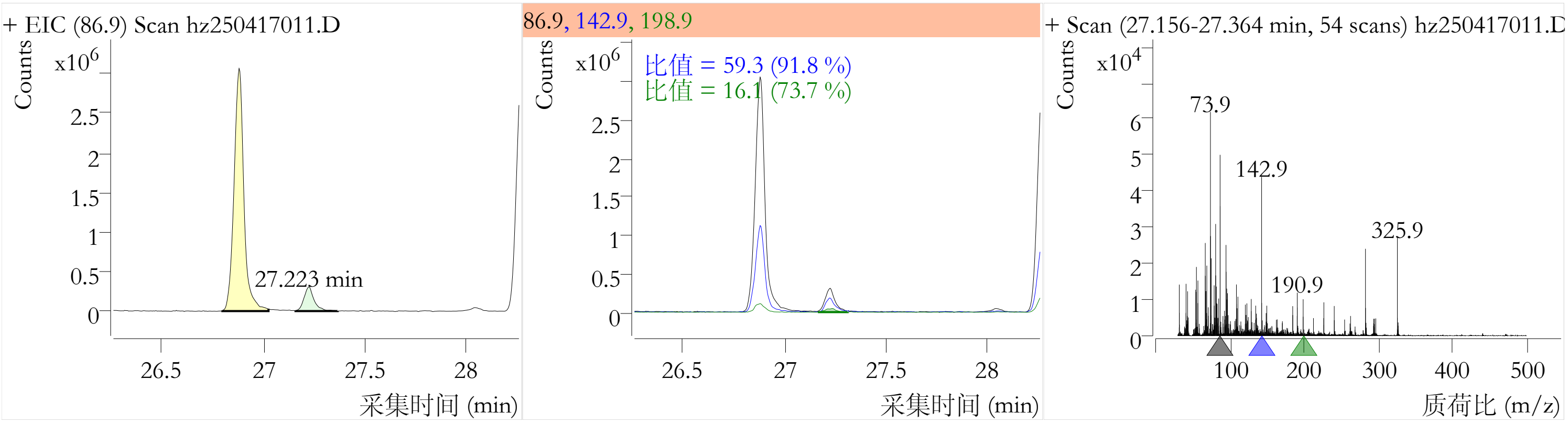

C18:3n6

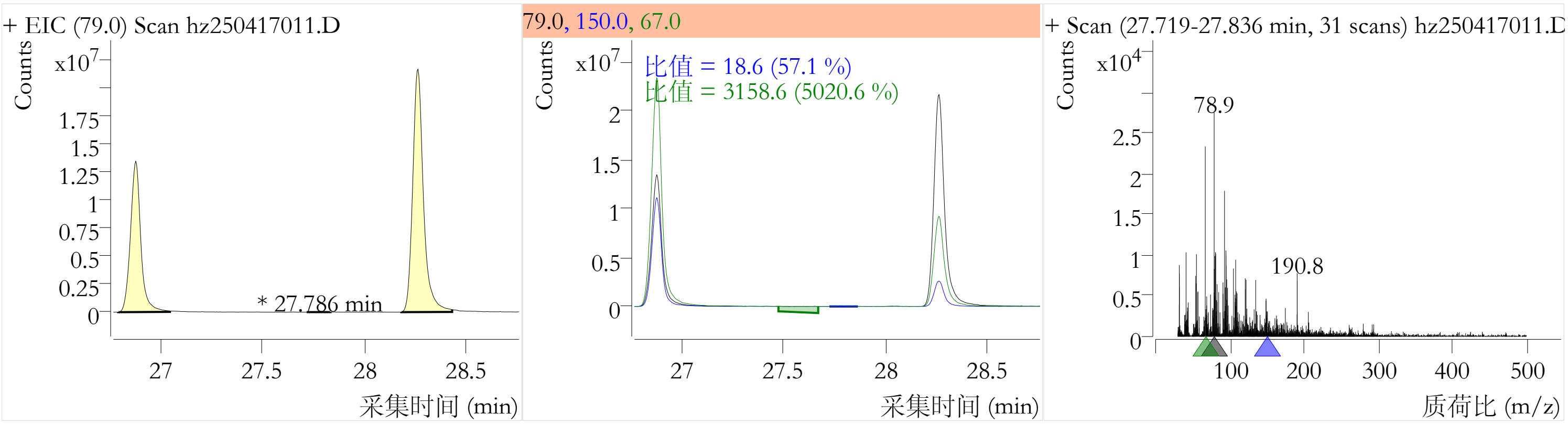

C20:1

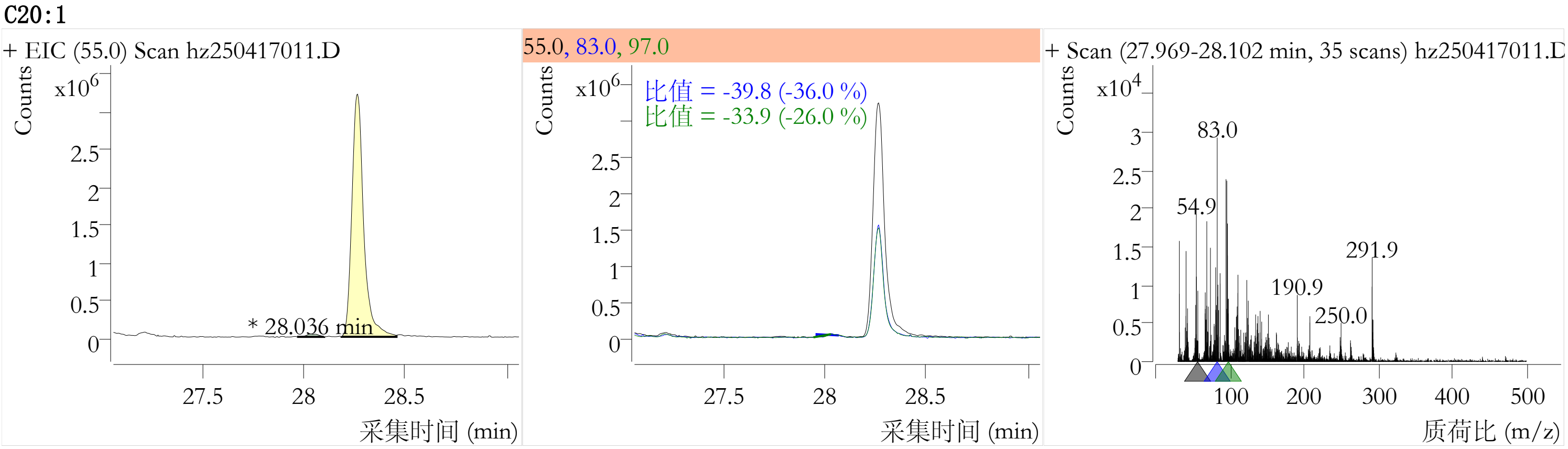

C18:3n3

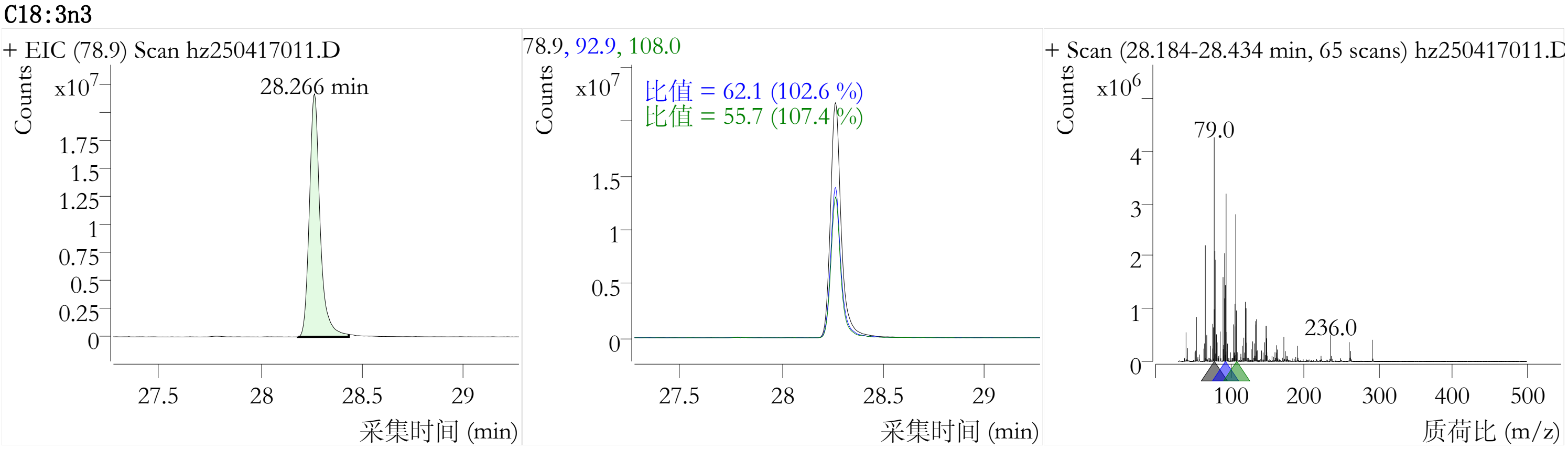

C21:0

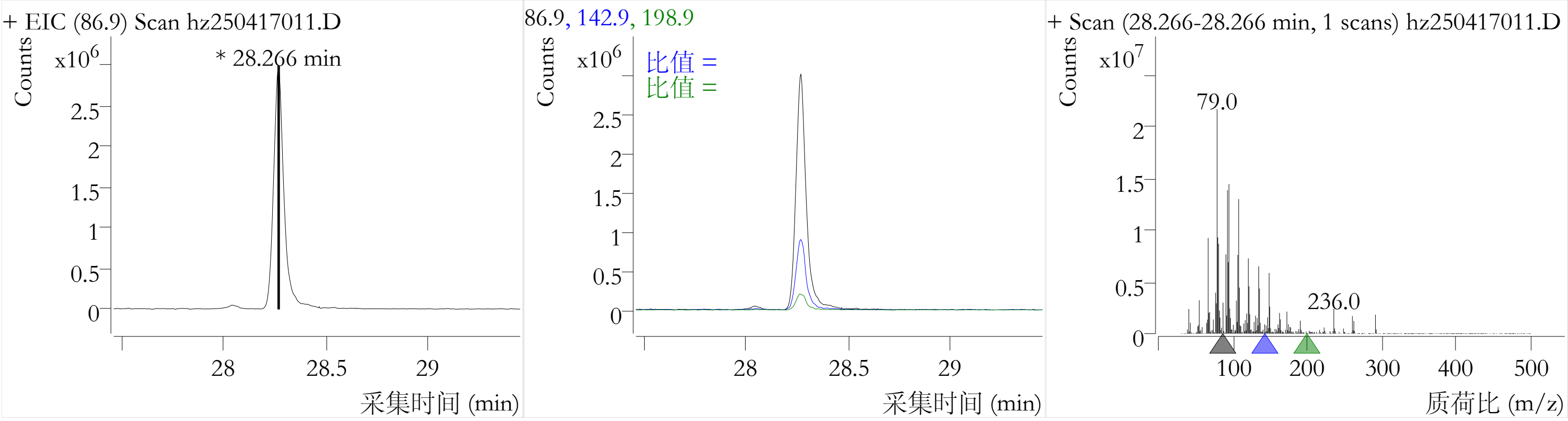

C20:2

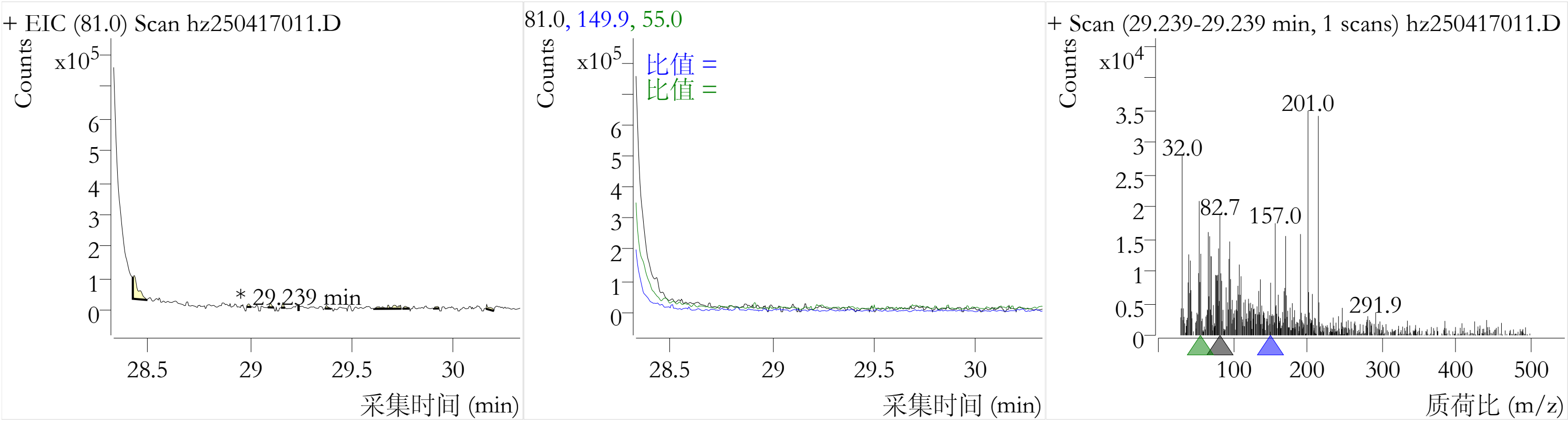

C22:0

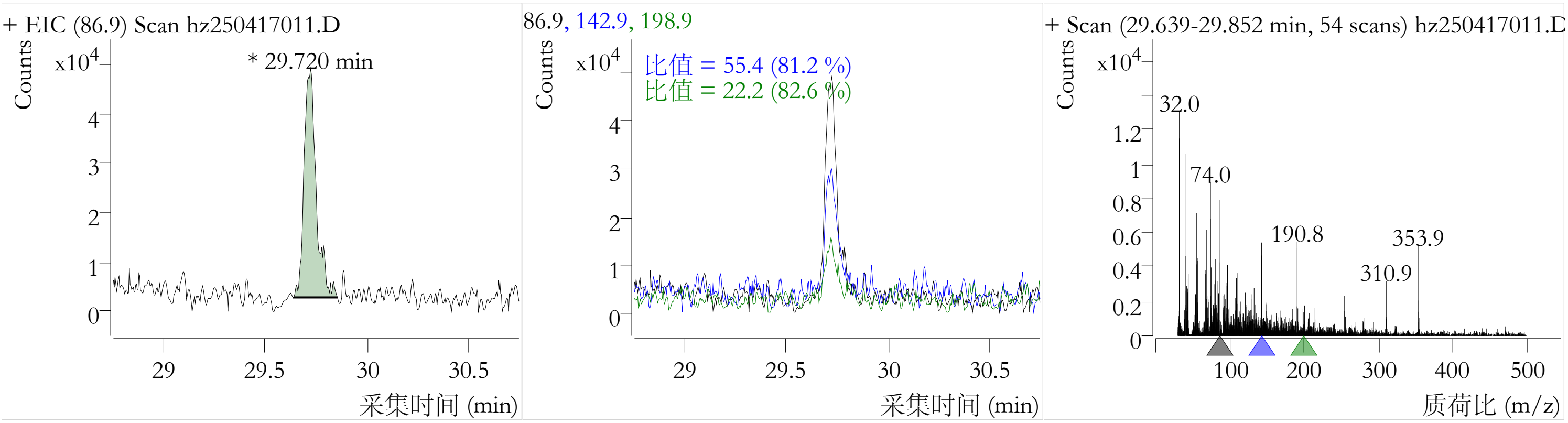

C20:3n6

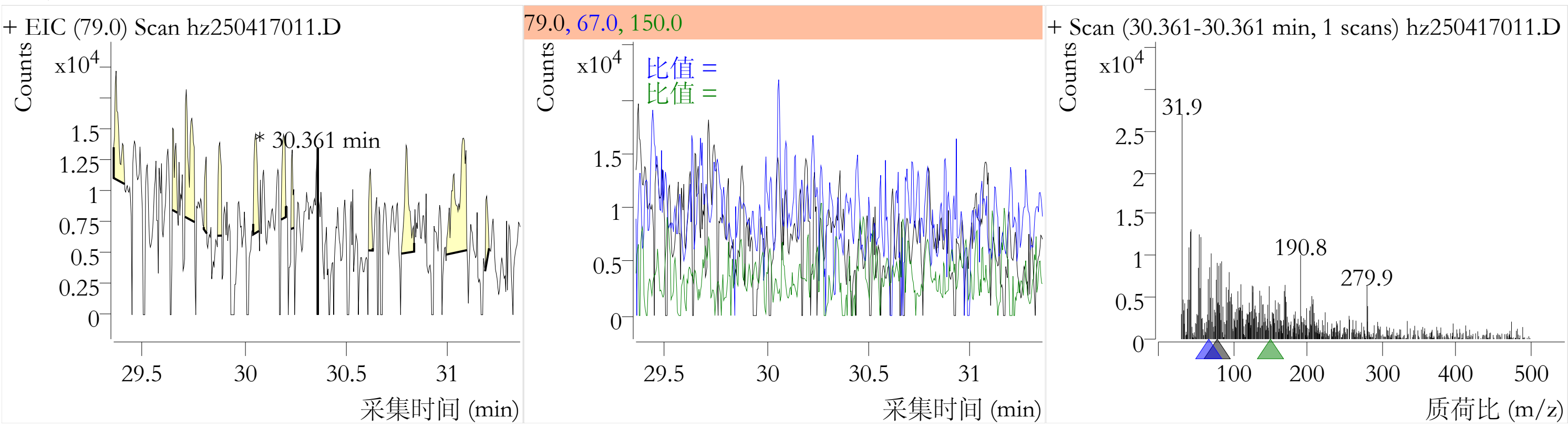

C22:1n9

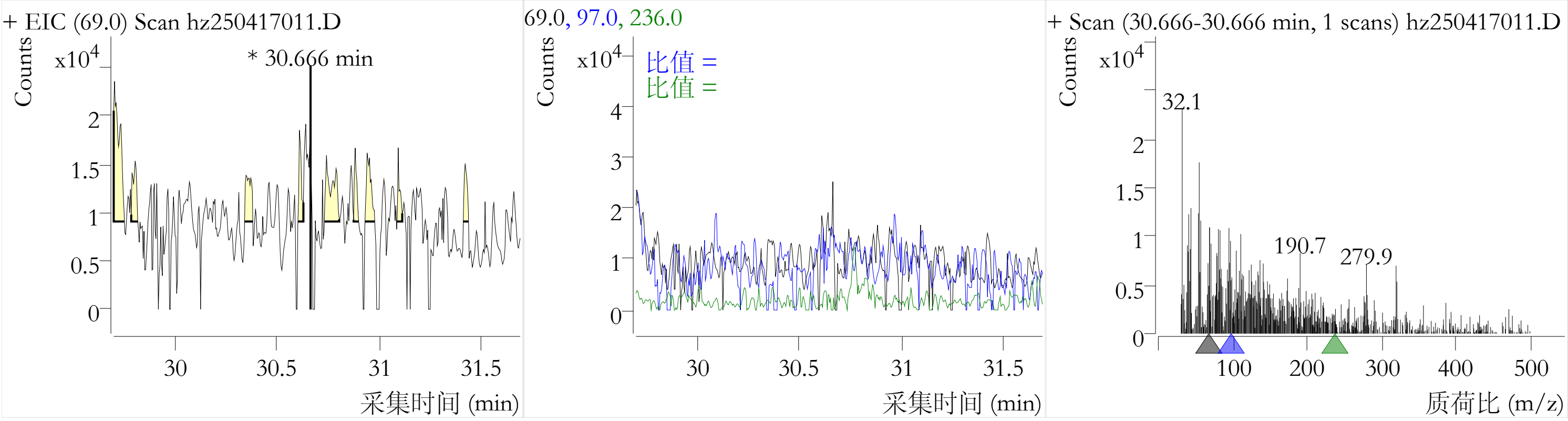

C20:3n3

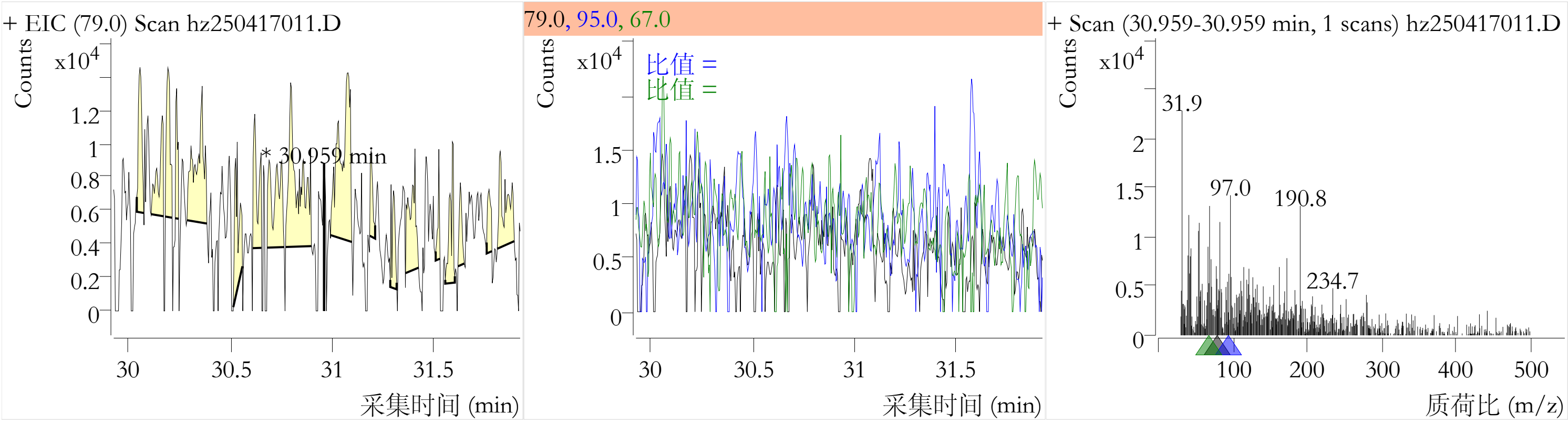

C23:0

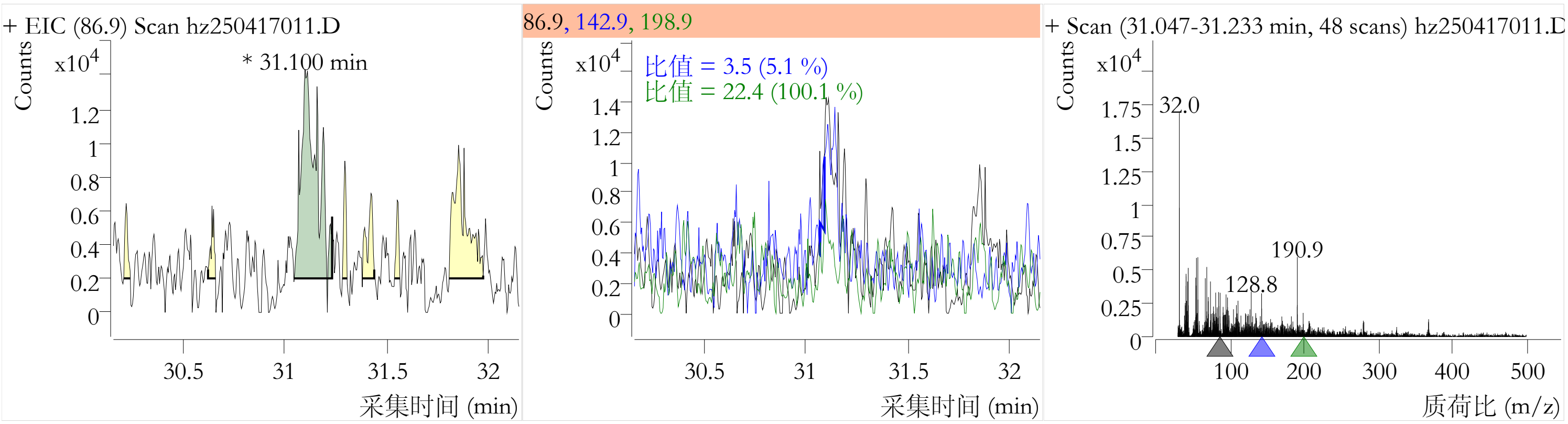

C20:4n6

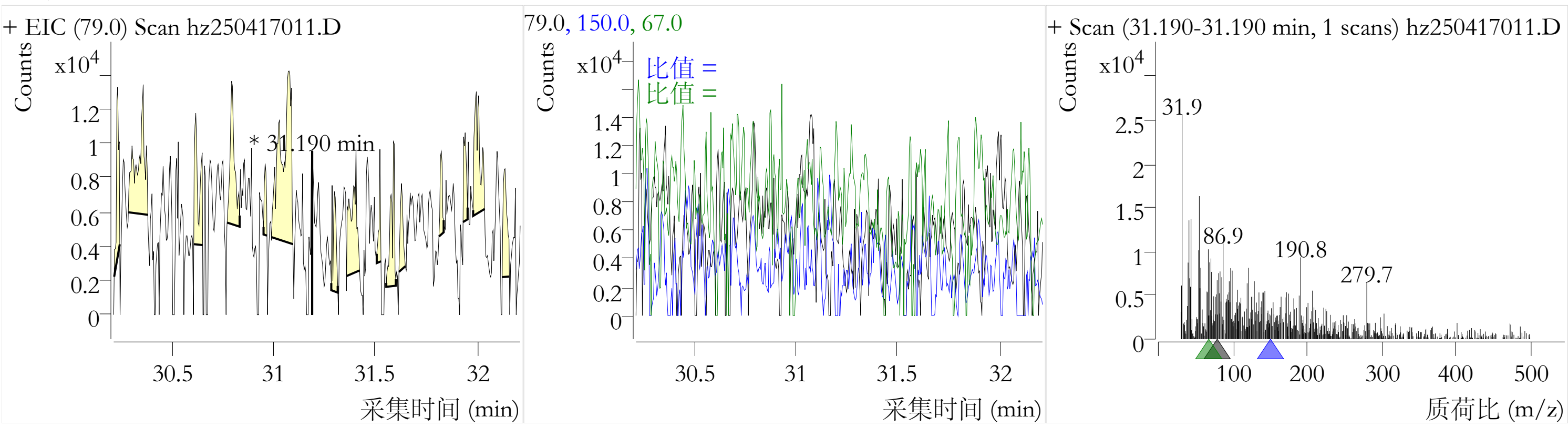

C22:2

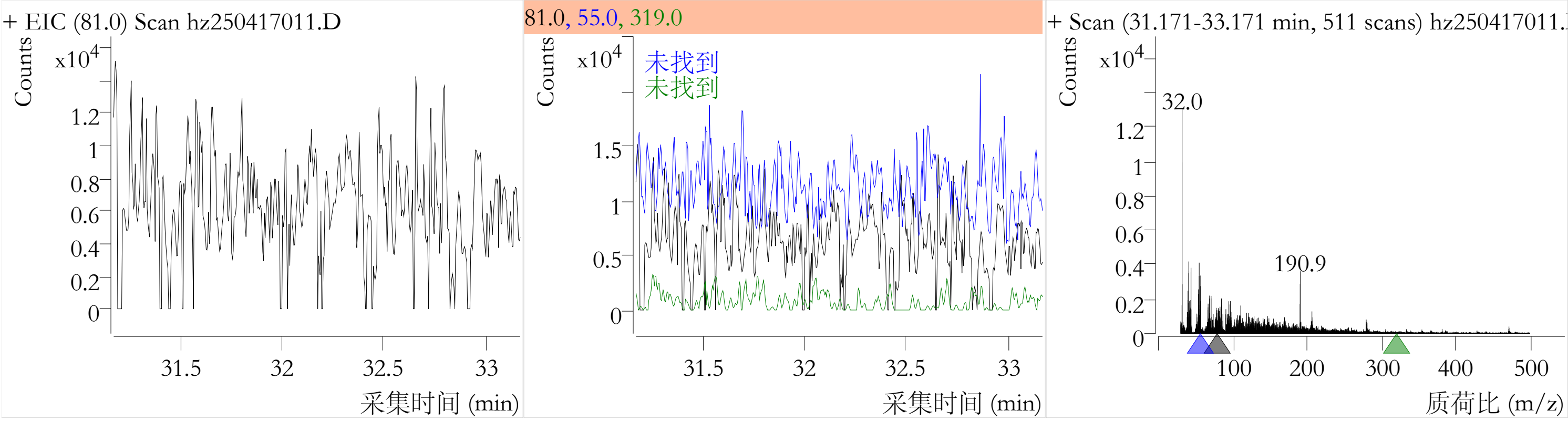

C24:0

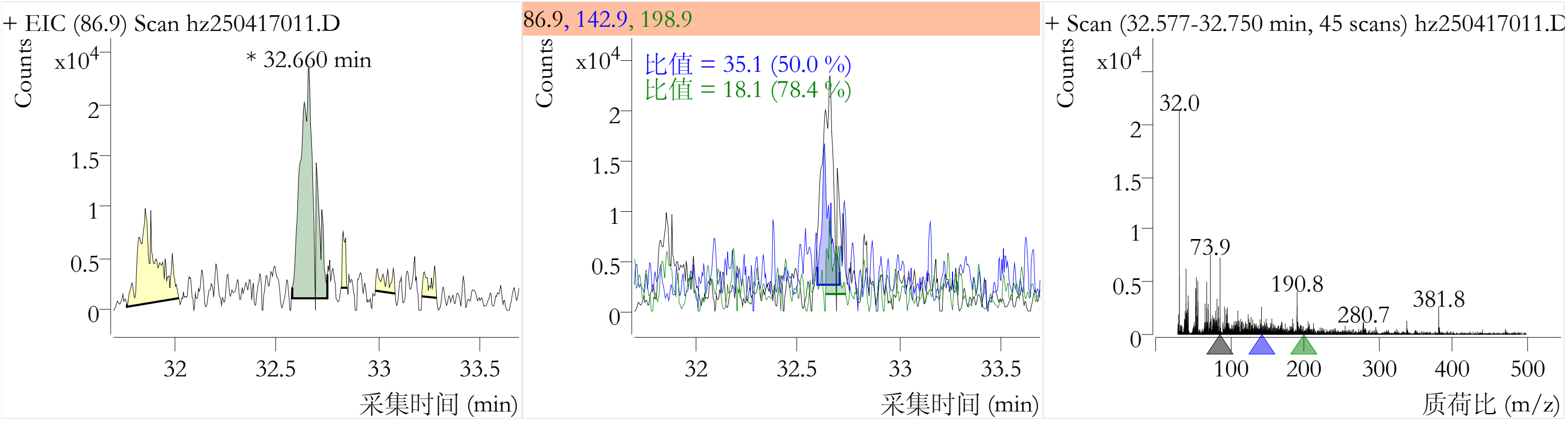

C20:5n3

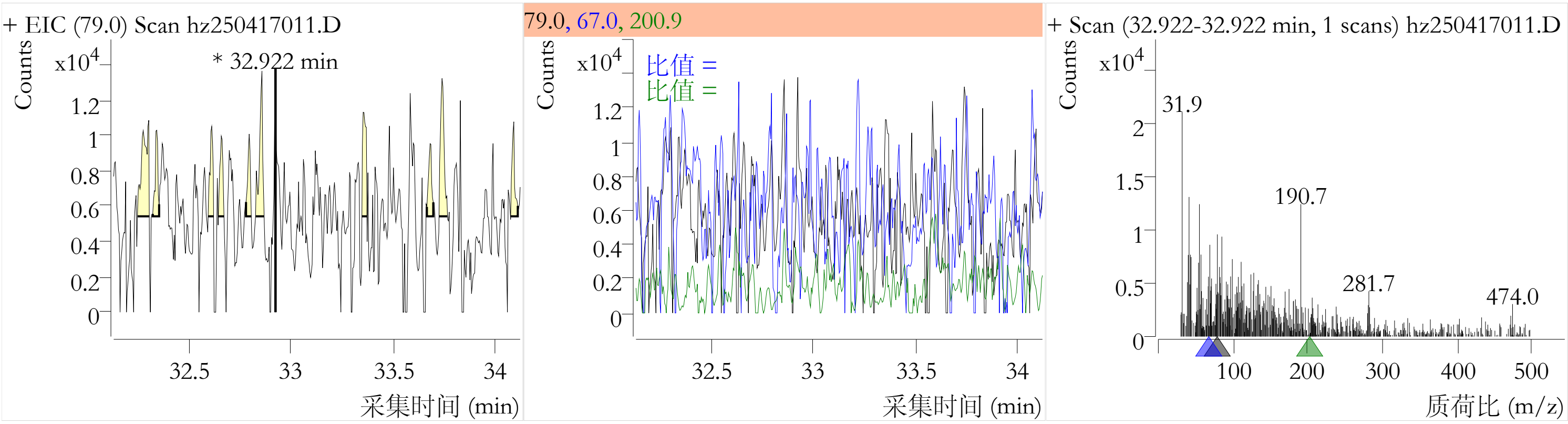

C24:1

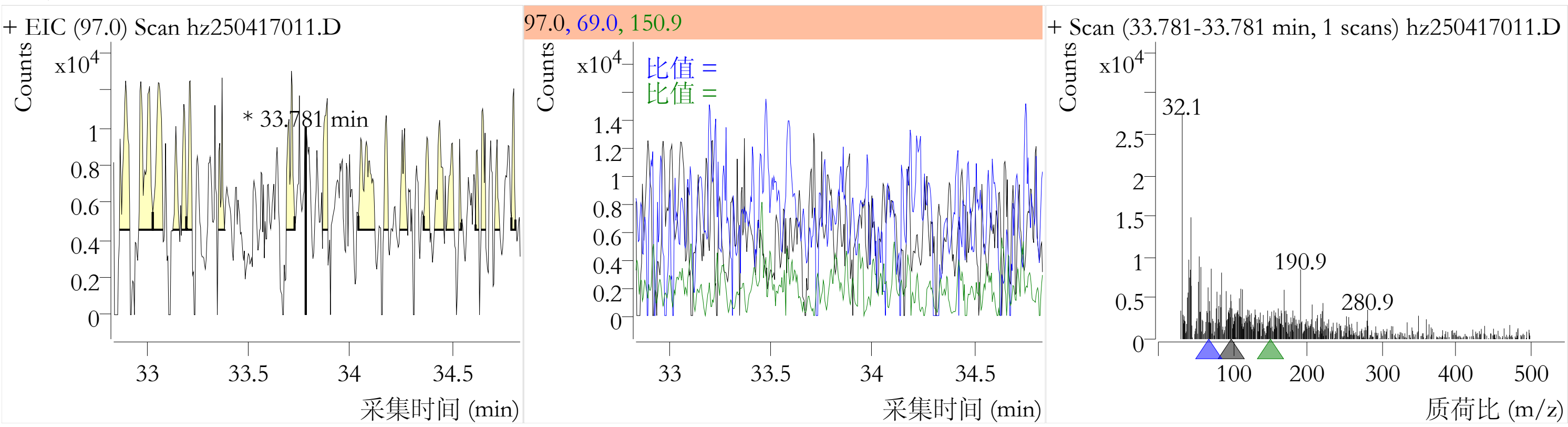

C22:6n3

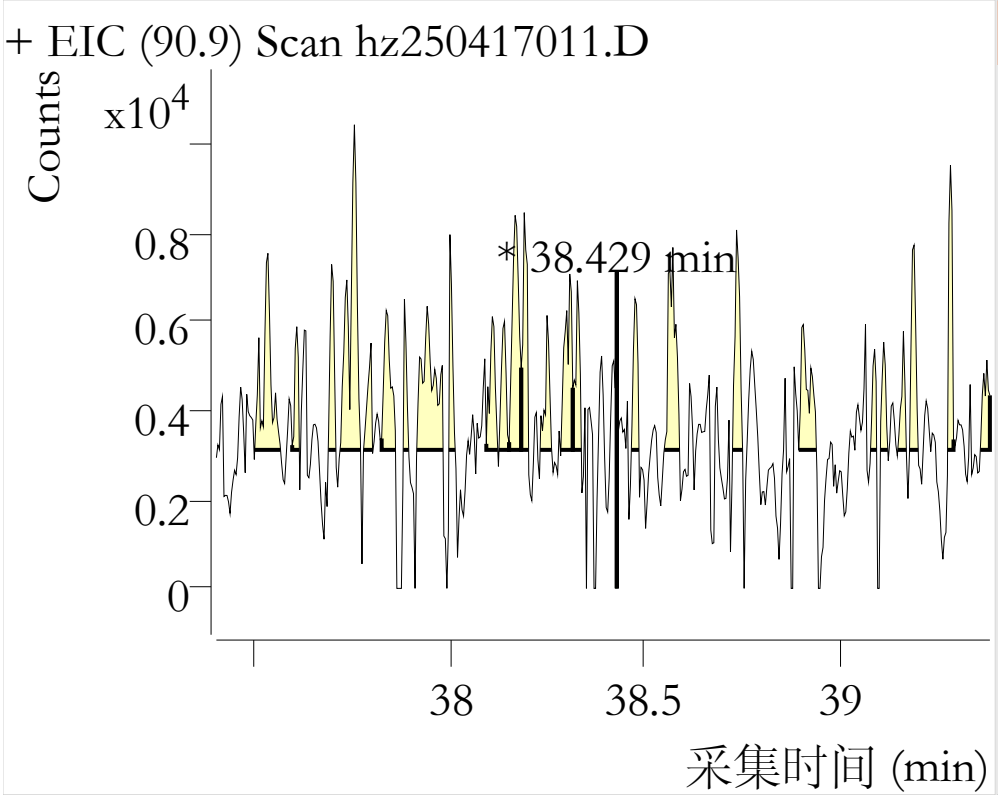
+ EIC (90.9) Scan hz250417011.D
Counts
x10^4
* 38.429 min
采集时间 (min)

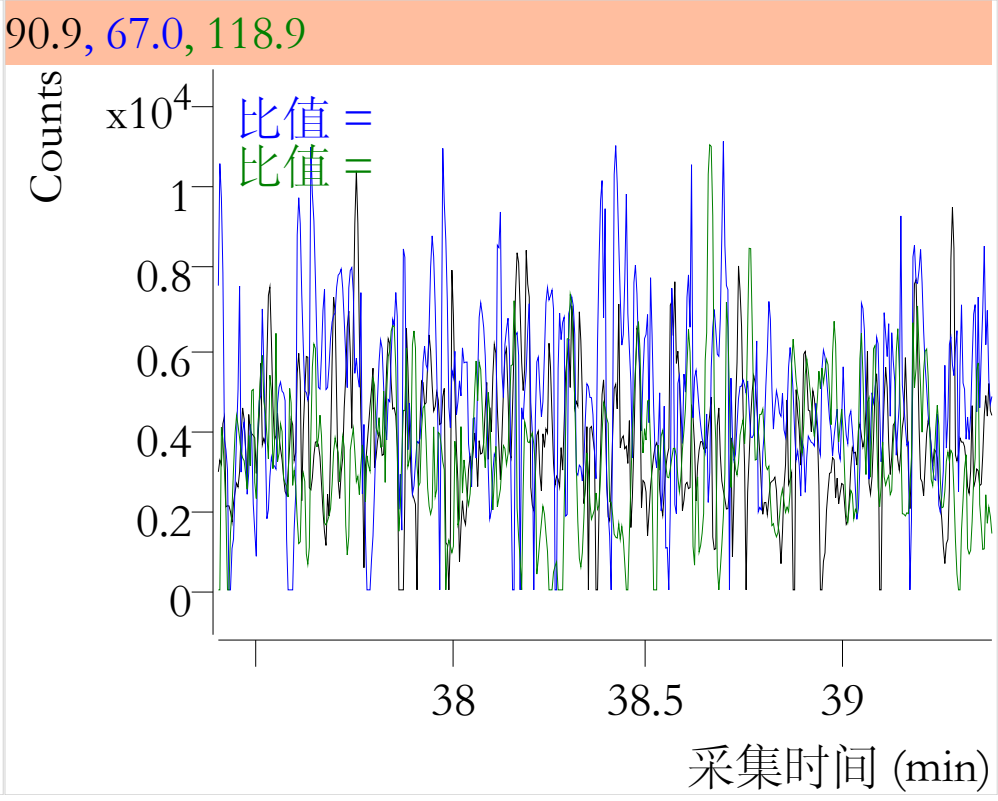
90.9, 67.0, 118.9
Counts
x10^4
比值 =
比值 =
采集时间 (min)

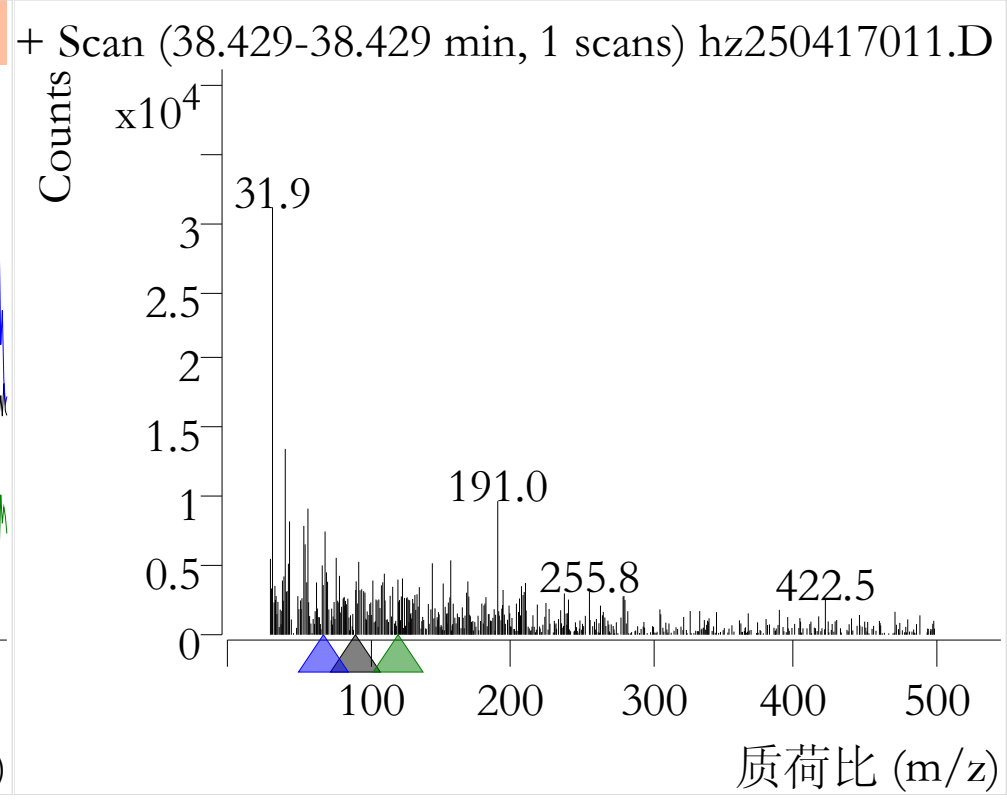
+ Scan (38.429-38.429 min, 1 scans) hz250417011.D
Counts
x10^4
31.9
191.0
255.8
422.5
质荷比 (m/z)

定量分析报告（根据样品）

Agilent Technologies

**采集日期时间**	2025/5/13 15:18:32	**数据文件**	hz250417012.D
**类型**	样品	**名称**	hz250417012
**稀释**	1	**采集方法文件**	FAME-TEST-SCAN

样品色谱图

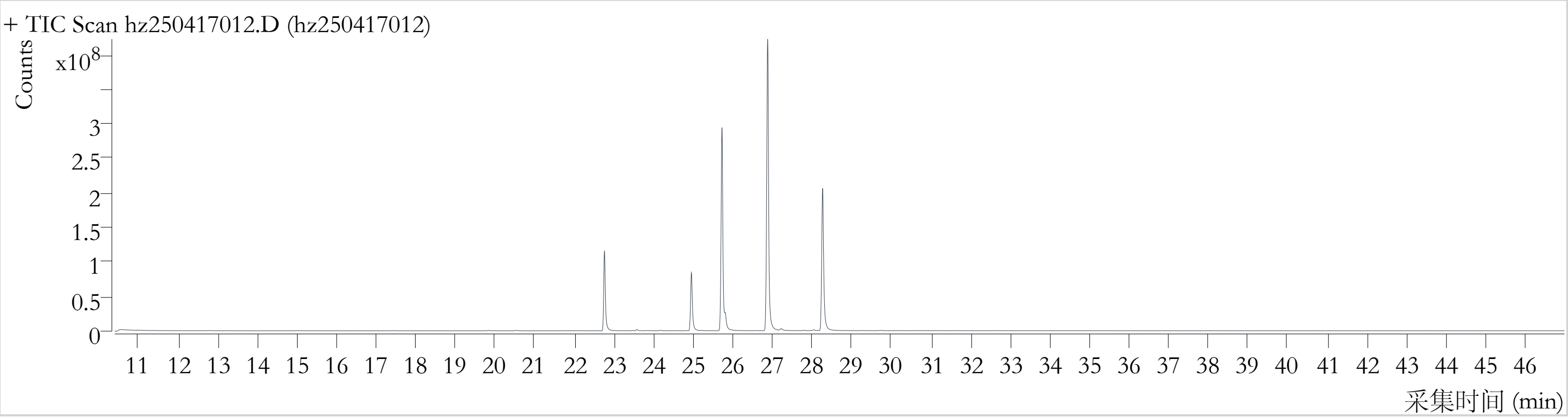

名称	RT	离子对	响应	峰高	最终浓度 单位	比值
C6:0	11.909	74.0	0	2868.56	ND ug/ml	
C8:0	13.847	74.0	0	5352.56	ND ug/ml	
C10:0	16.009	74.0	0	6201.94	ND ug/ml	
C11:0	17.146	74.0	0	6429.90	ND ug/ml	
C12:0	18.280	74.0	21843	14615.15	0.0511 ug/ml	73.0
C13:0	19.413	74.0	0	3629.14	ND ug/ml	
C14:0	20.531	74.0	473482	135077.88	1.1756 ug/ml	46.8
C15:0	21.649	74.0	25122	7362.73	0.0649 ug/ml	55.5
C15:1	22.763	55.0	0	2040069.38	ND ug/ml	
C16:0	22.763	87.0	46396449	15220923.53	129.1287 ug/ml	58.3
C16:1	23.575	55.0	209407	64967.91	2.8344 ug/ml	71.0
C17:0	23.841	74.0	180619	46978.08	0.5256 ug/ml	43.6
C17:1	24.955	55.0	0	1474276.25	ND ug/ml	
C18:0	24.955	86.9	34225396	10256584.71	104.7075 ug/ml	62.8
C18:1n9t	25.725	55.0	0	6106287.50	ND ug/ml	
C18:1n9c	25.725	55.0	24258878	6093660.44	349.0073 ug/ml	101.6
C18:2n6t	25.725	81.0	0	5490281.00	ND ug/ml	
C18:2n6c	26.878	80.9	92236801	26951304.49	605.5238 ug/ml	35.0
C20:0	27.218	86.9	982167	283363.62	2.9614 ug/ml	63.1
C18:3n6	27.788	79.0	227497	57516.76	1.3794 ug/ml	799.3
C20:1	28.031	55.0	77806	29062.27	1.0418 ug/ml	-381.2
C18:3n3	28.265	78.9	66441736	17961997.45	313.2492 ug/ml	61.2
C21:0	28.265	86.9	0	2346248.00	ND ug/ml	
C22:0	29.731	86.9	201820	49217.74	0.6679 ug/ml	44.0
C20:3n6	30.259	79.0	0	13005.62	ND ug/ml	
C22:1n9	30.684	69.0	0	16072.00	ND ug/ml	
C20:3n3	31.032	79.0	0	12261.83	ND ug/ml	
C23:0	31.091	86.9	53865	16761.46	0.1715 ug/ml	33.5
C20:4n6	31.267	79.0	0	11548.55	ND ug/ml	
C22:2	32.174	81.0	0	6413.19	ND ug/ml	
C24:0	32.643	86.9	92212	20629.97	0.3388 ug/ml	21.0
C20:5n3	33.194	79.0	0	8435.29	ND ug/ml	

C4:0

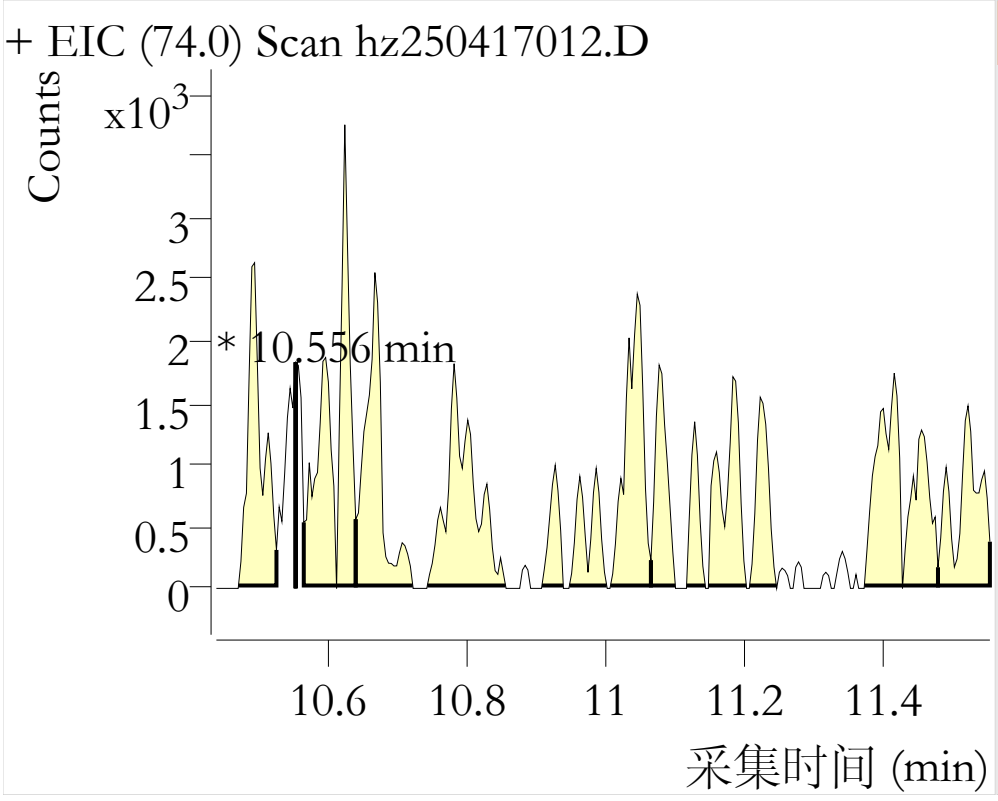

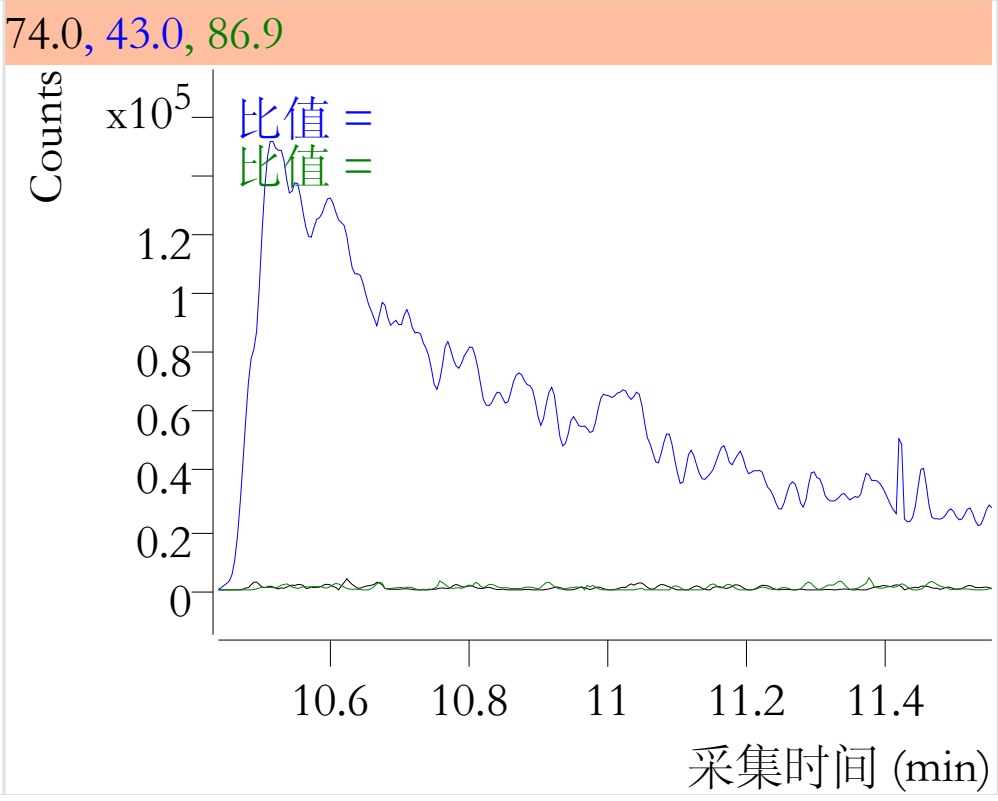

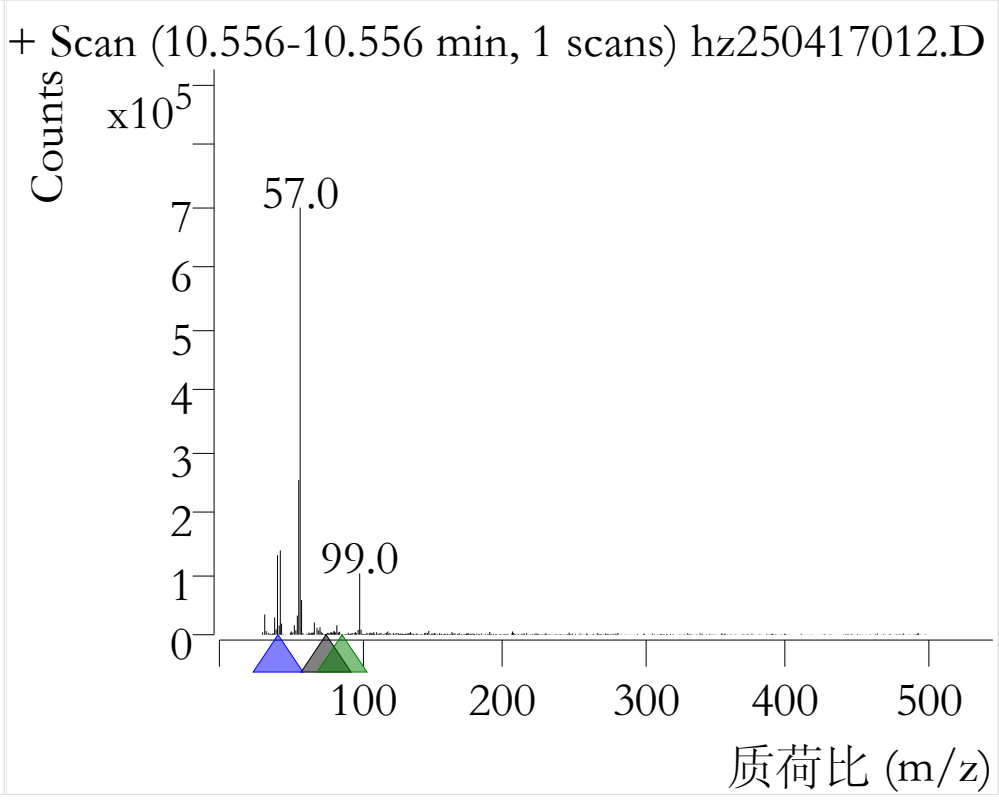

C6:0

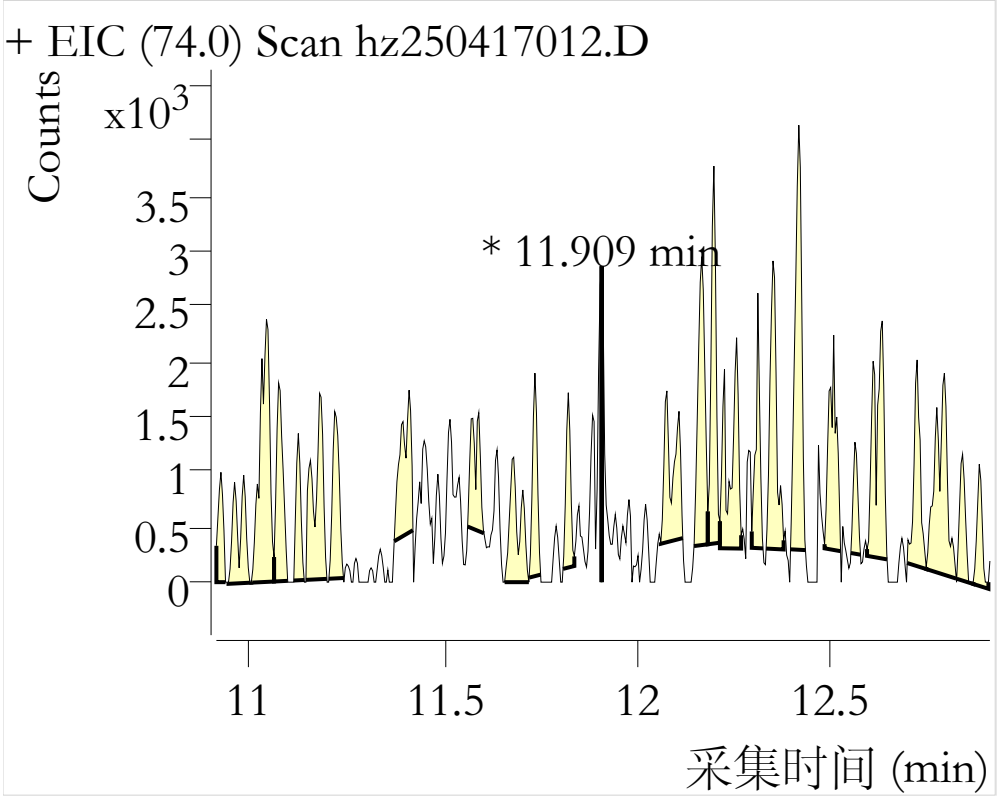

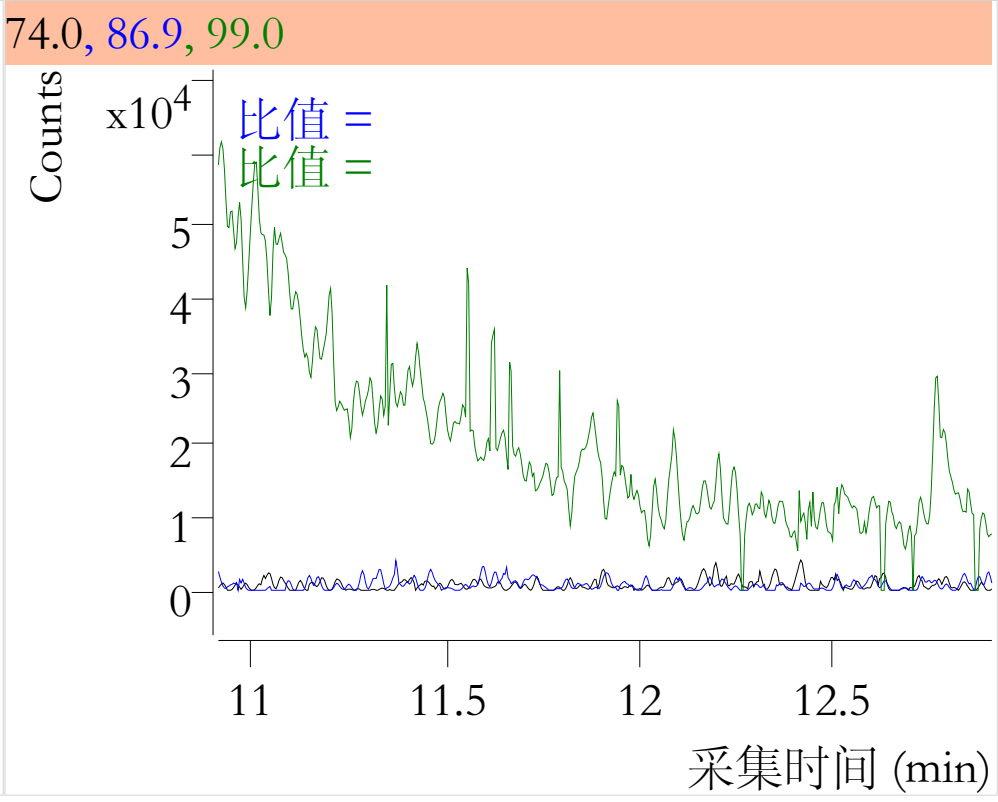

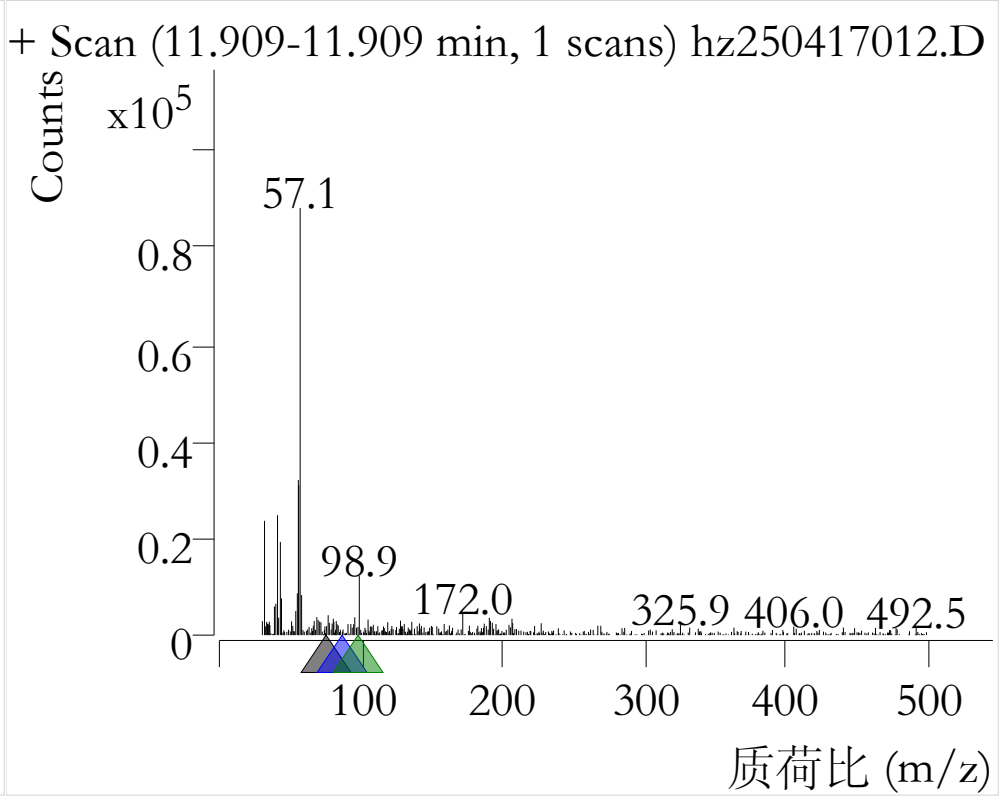

C8:0

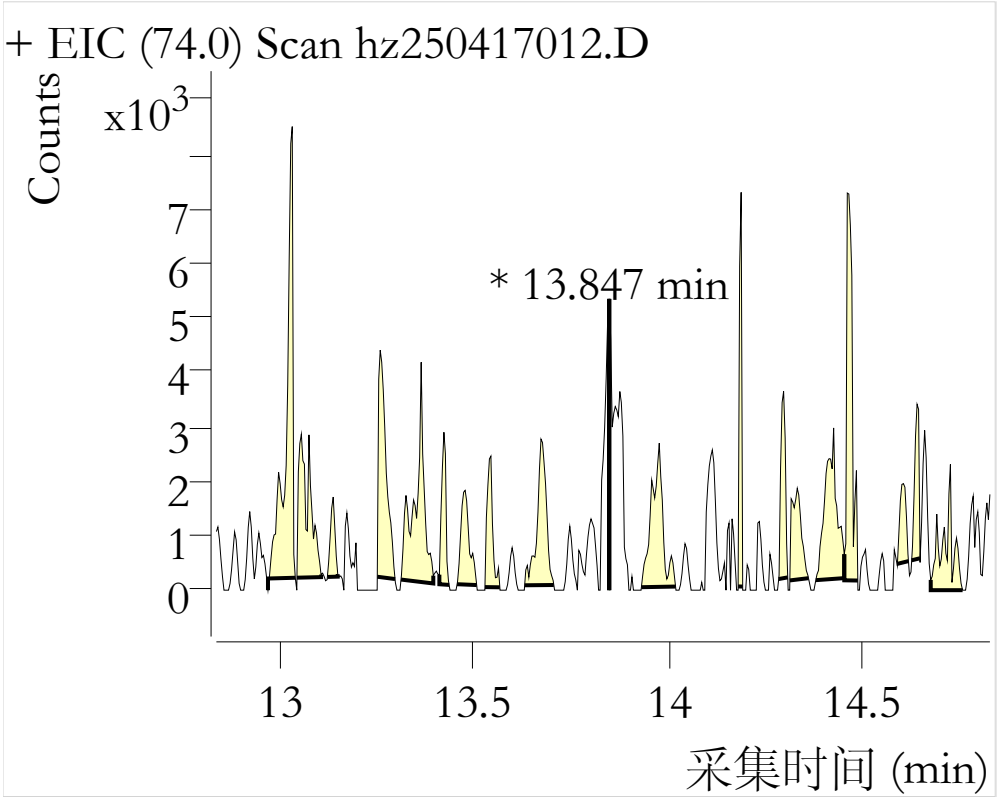

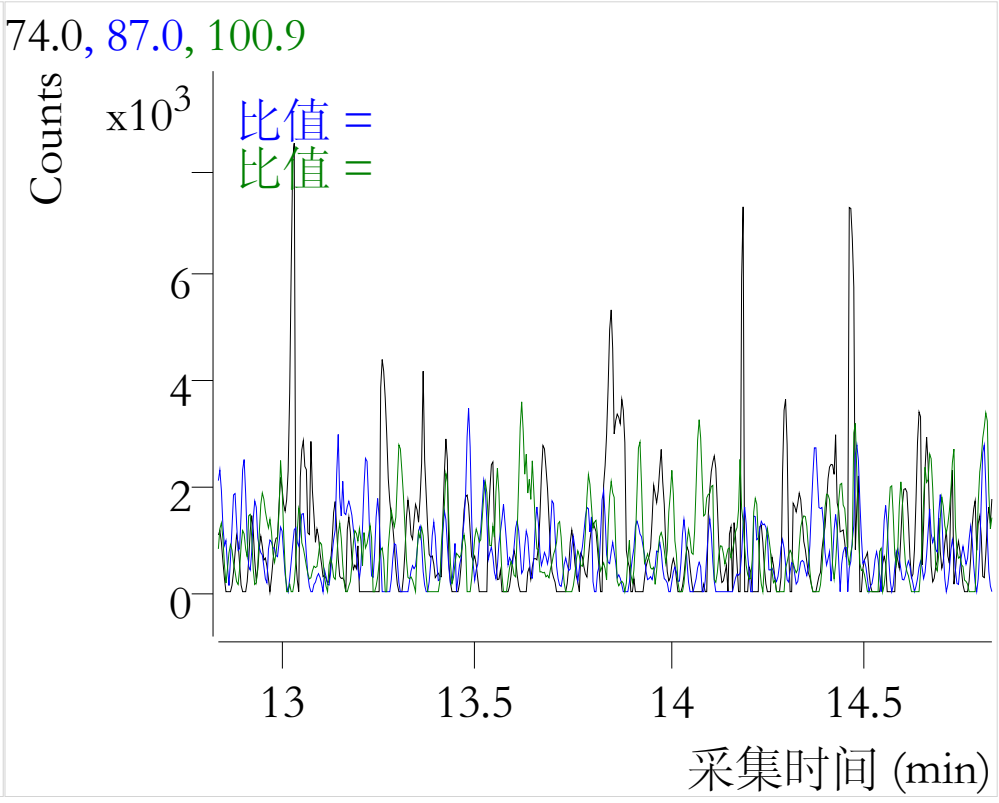

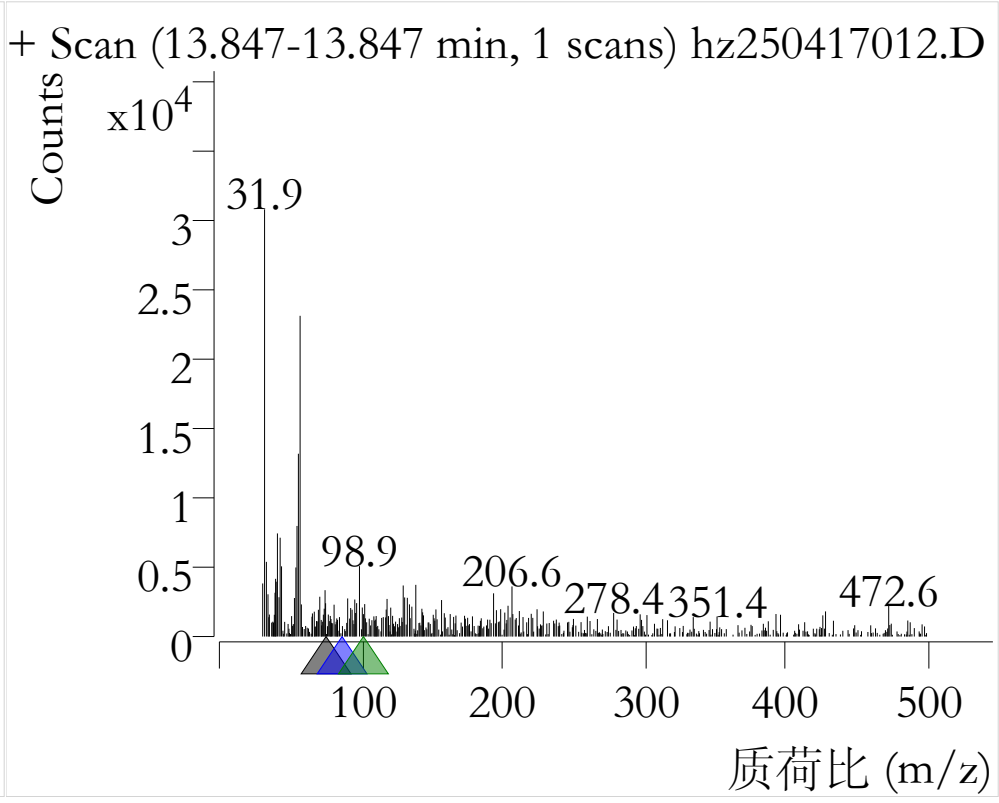

C10:0

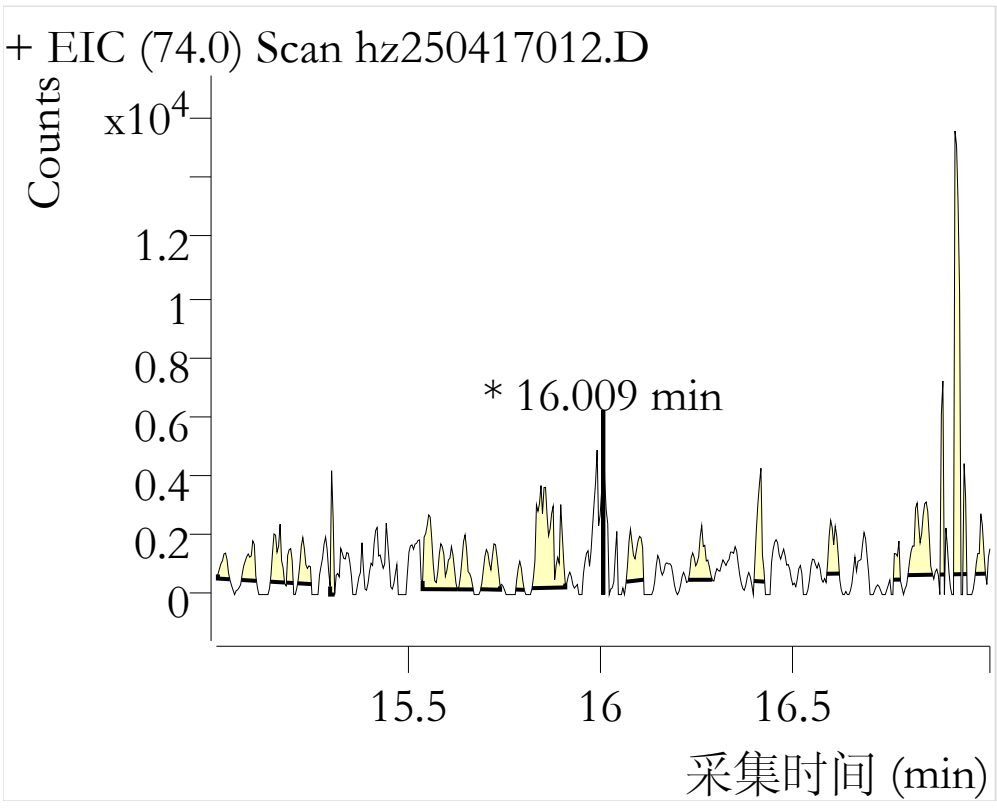

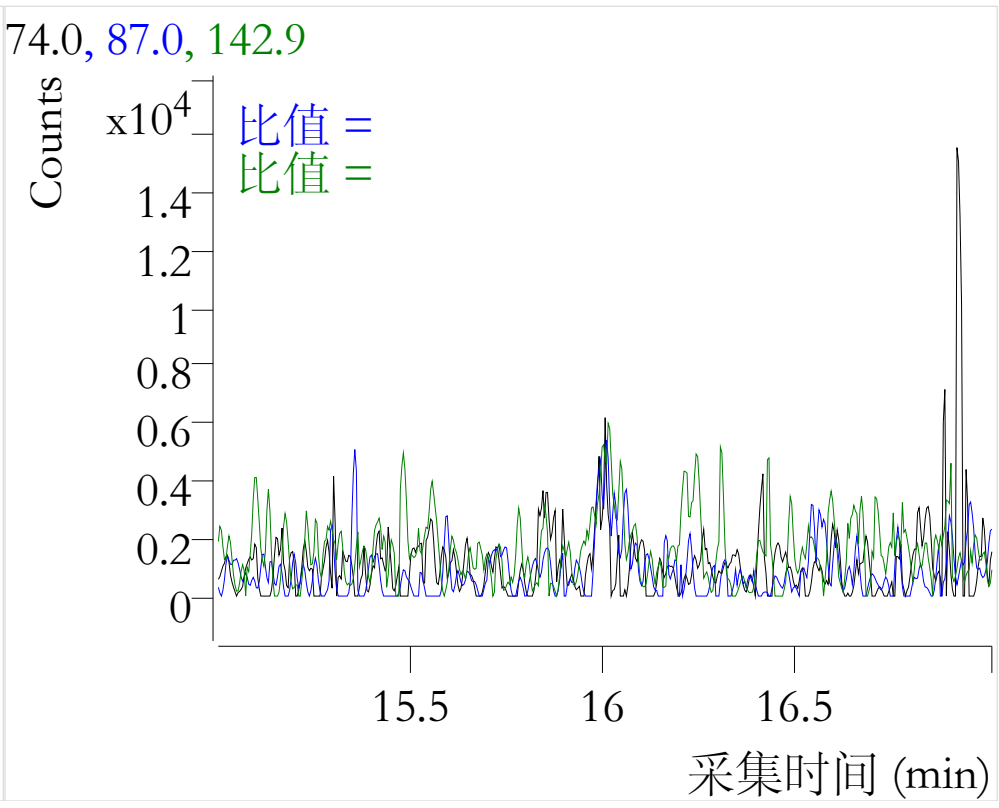

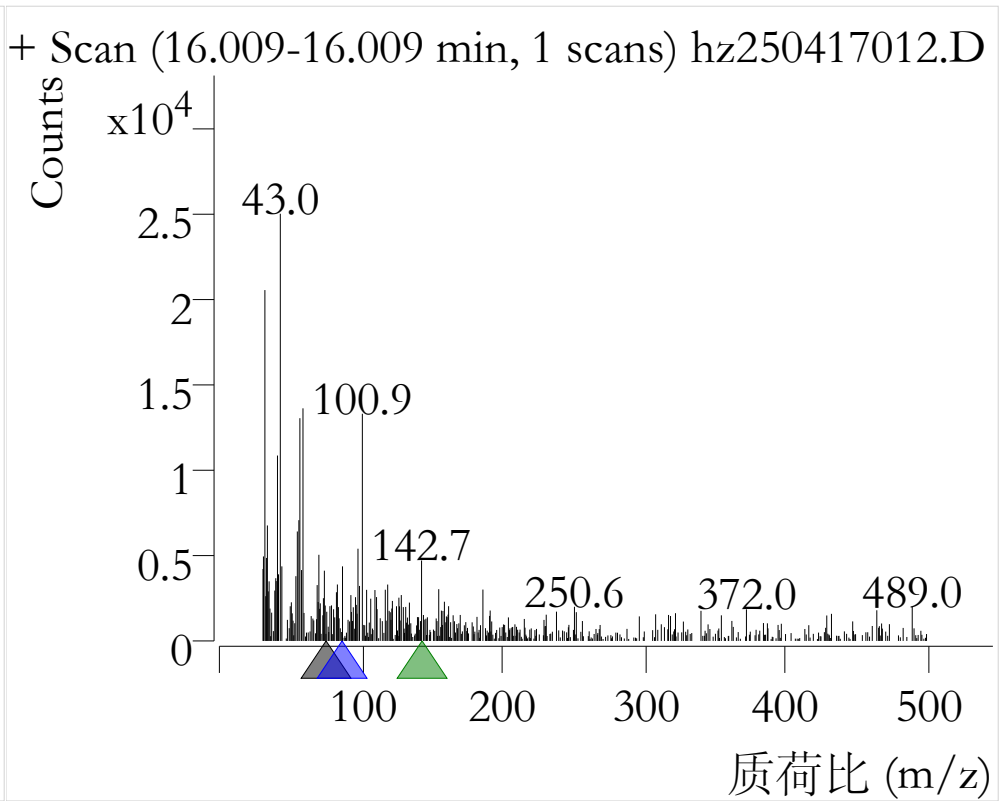

C11:0

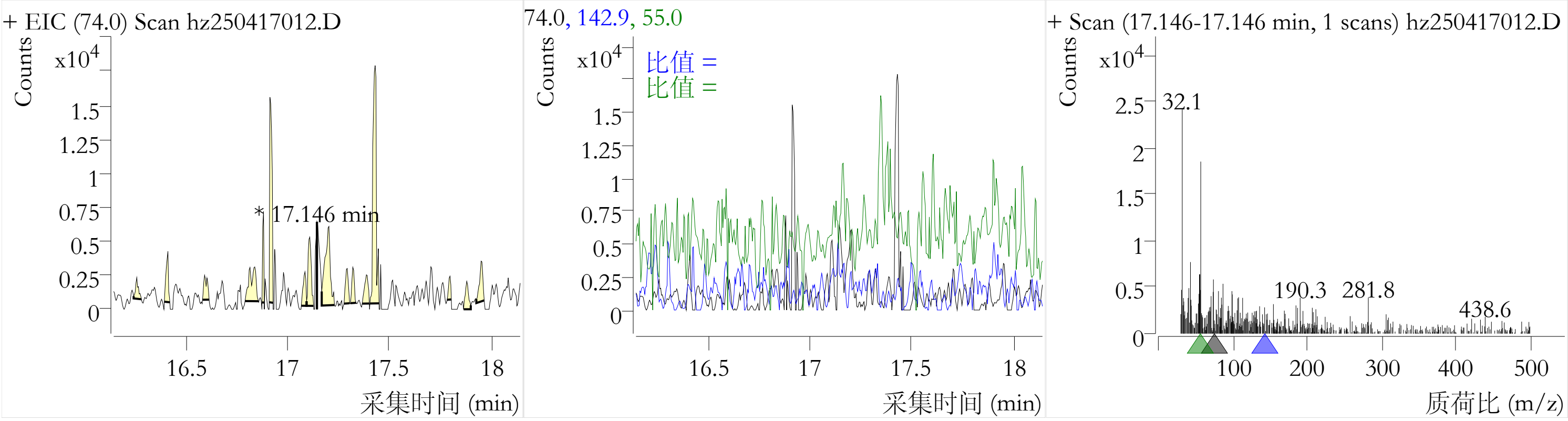

C12:0

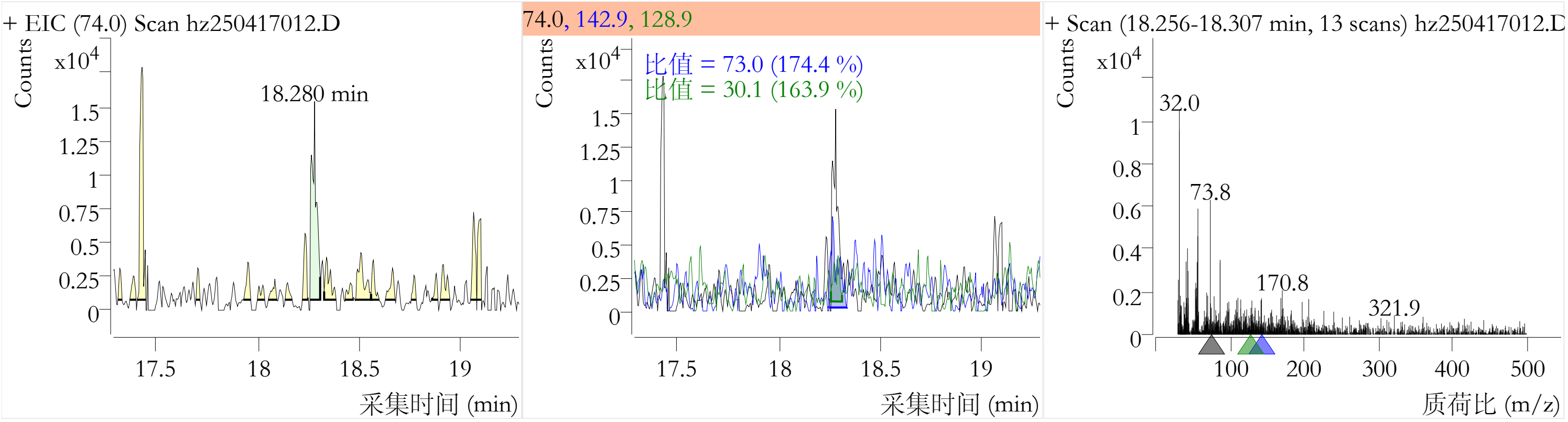

C13:0

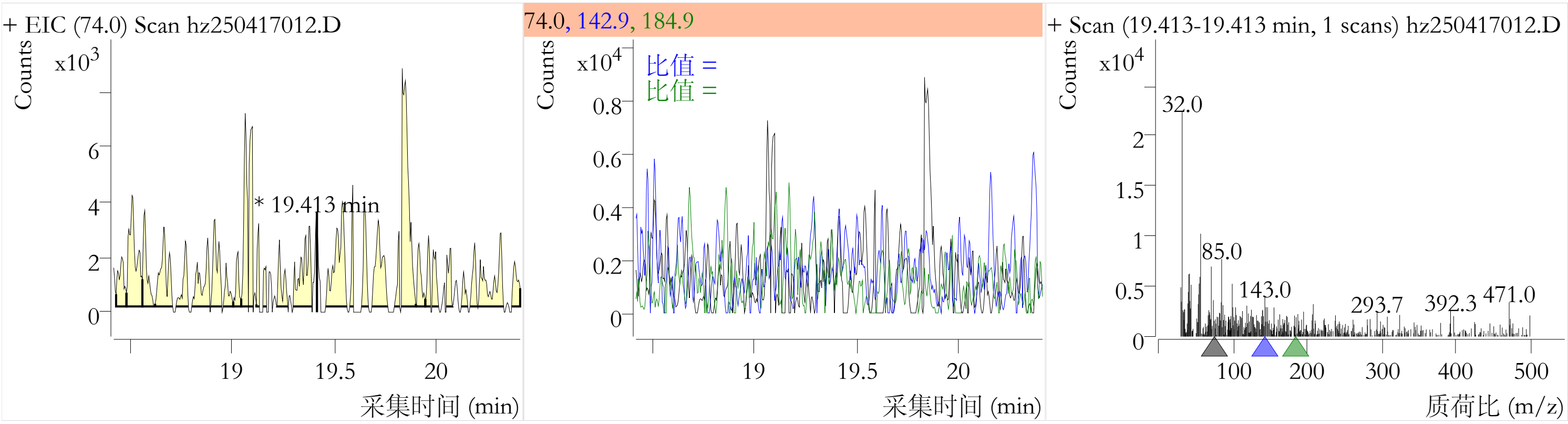

C14:0

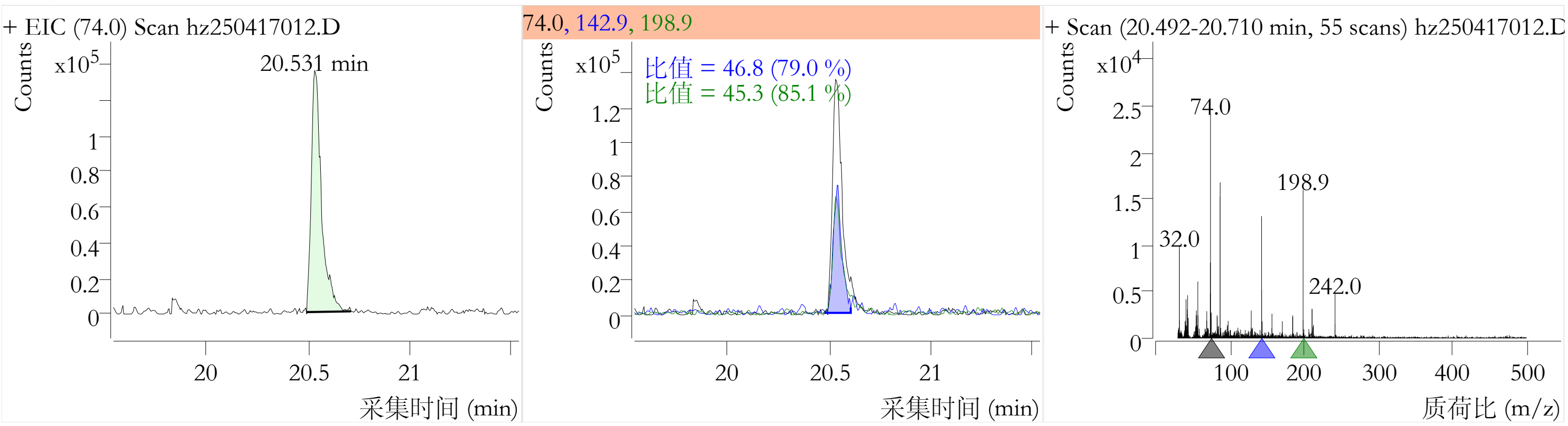

C14:1

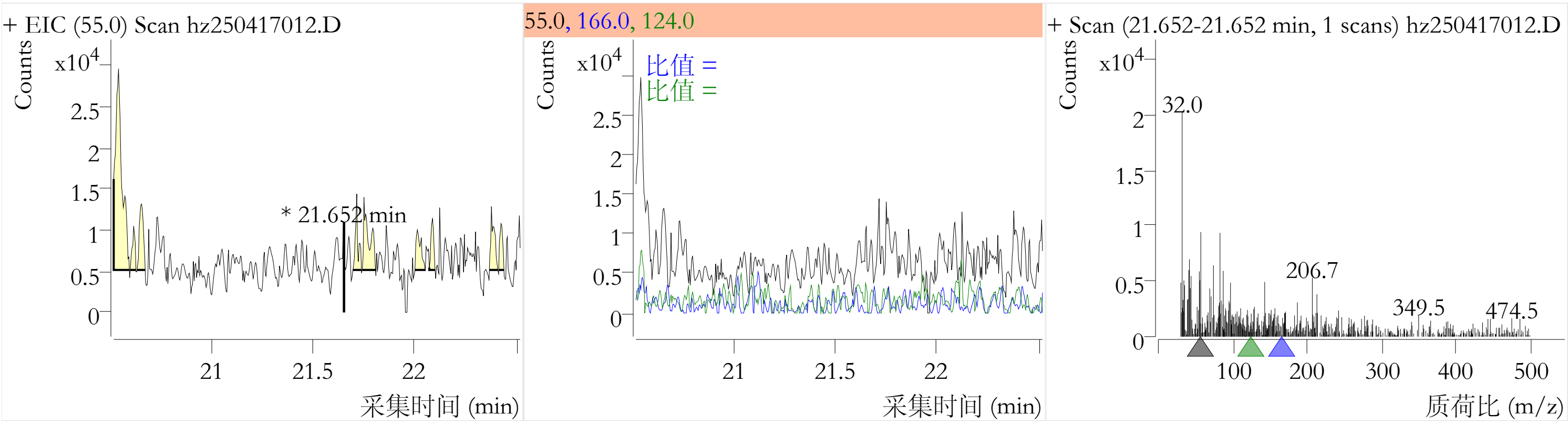

C15:0

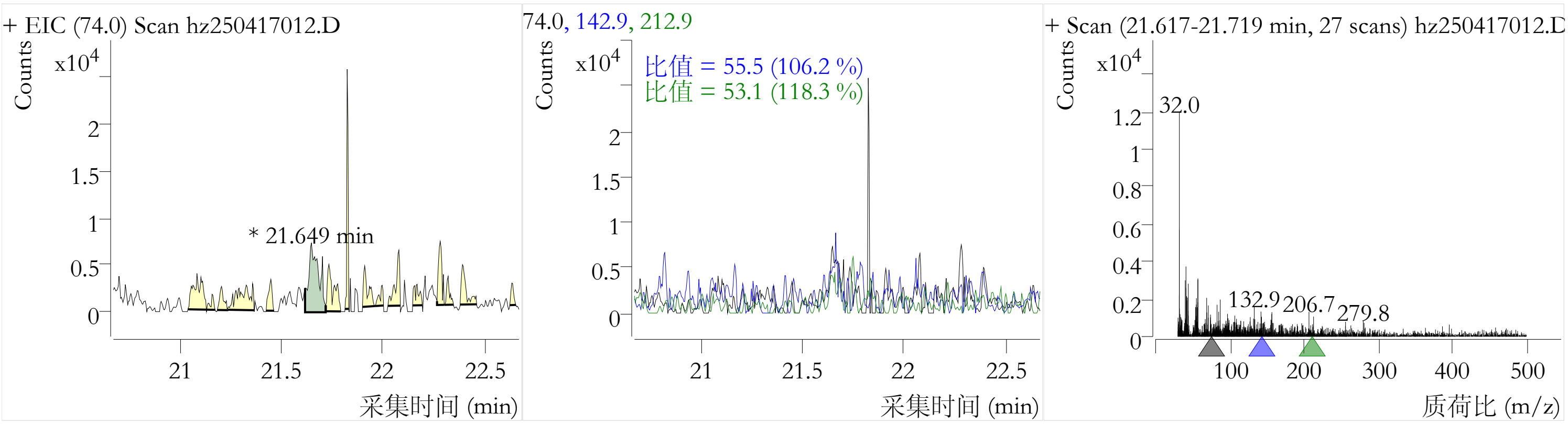

C15:1

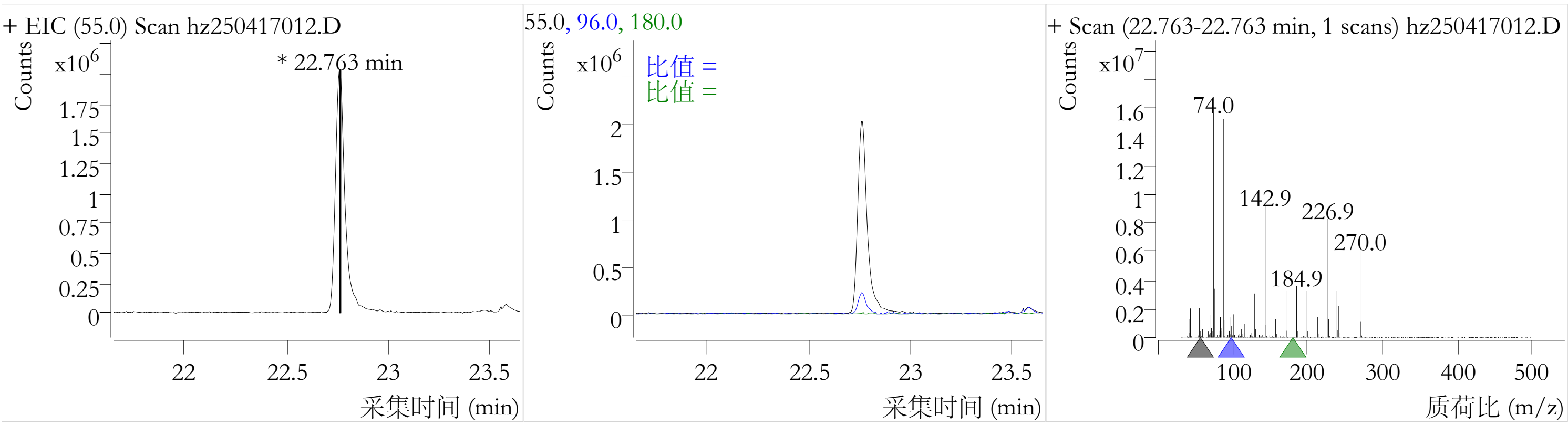

C16:0

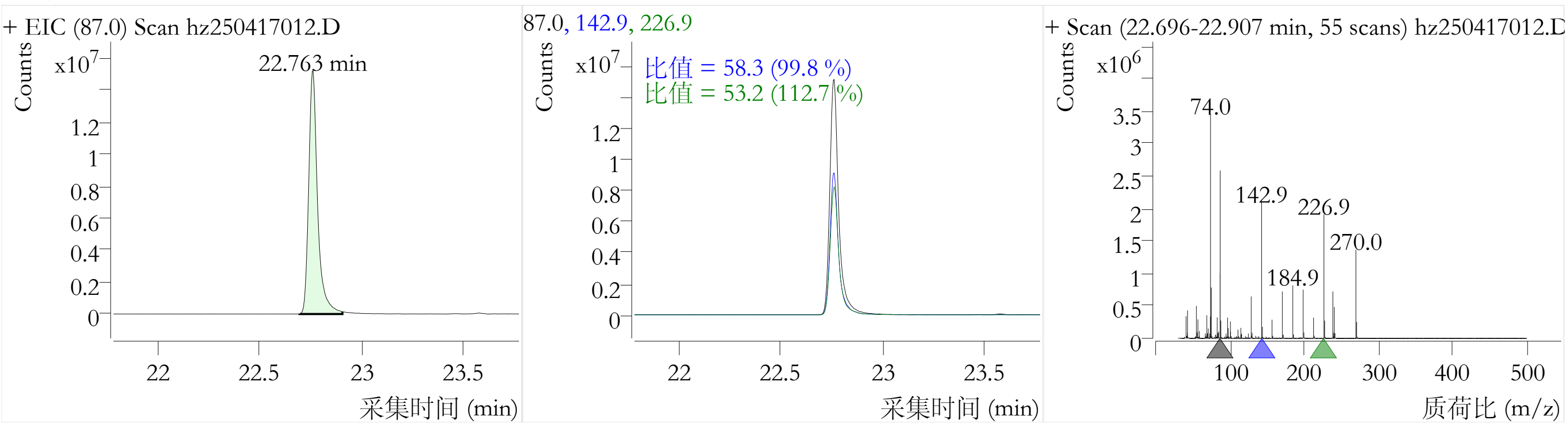

C16:1

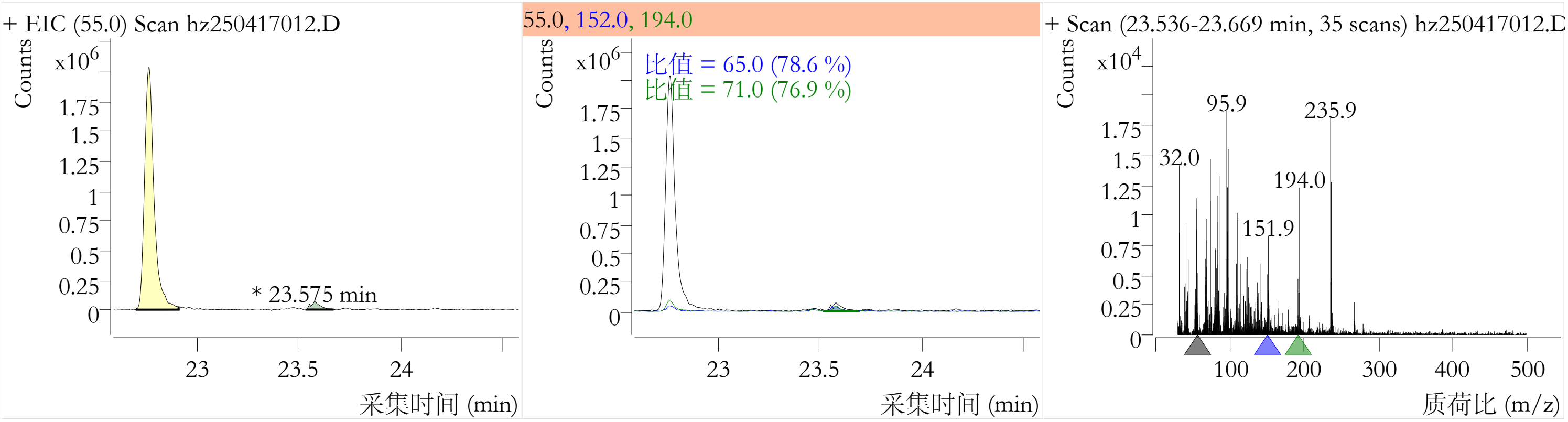

C17:0

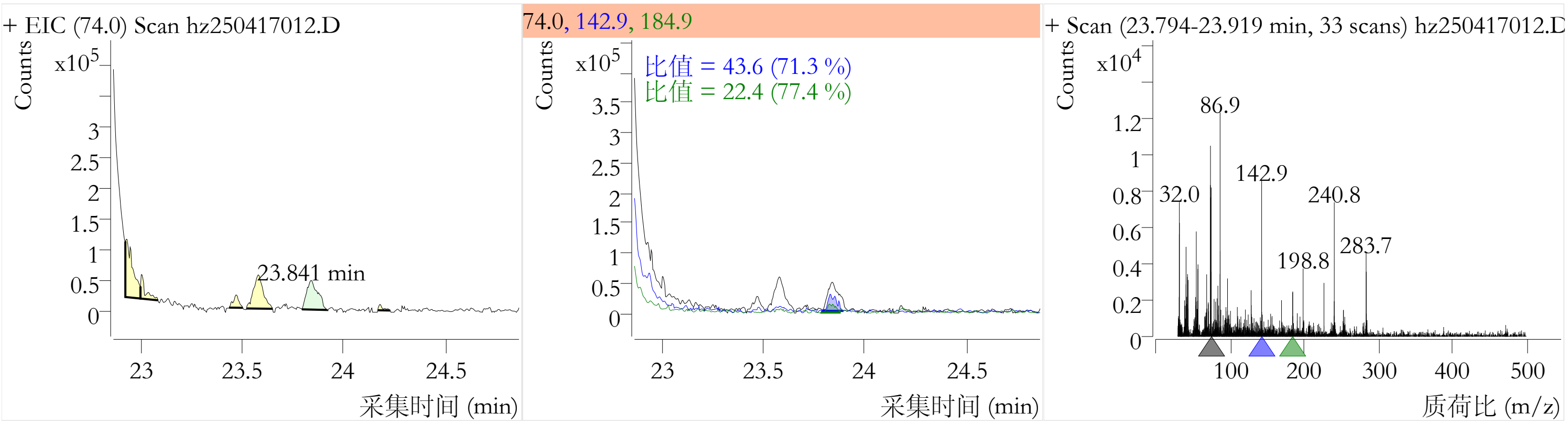

C17:1

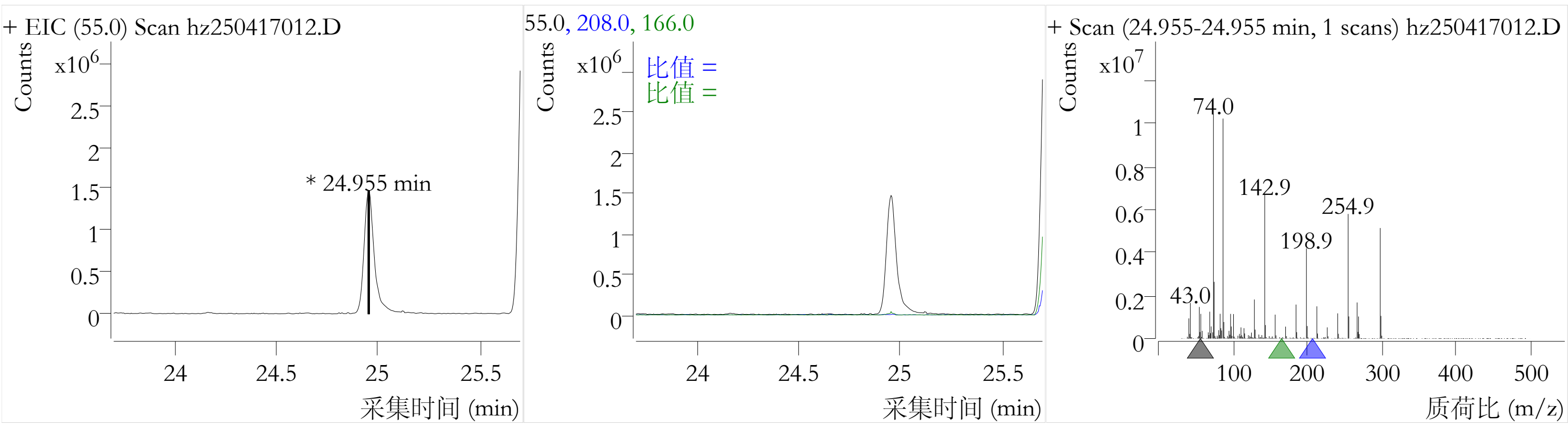

C18:0

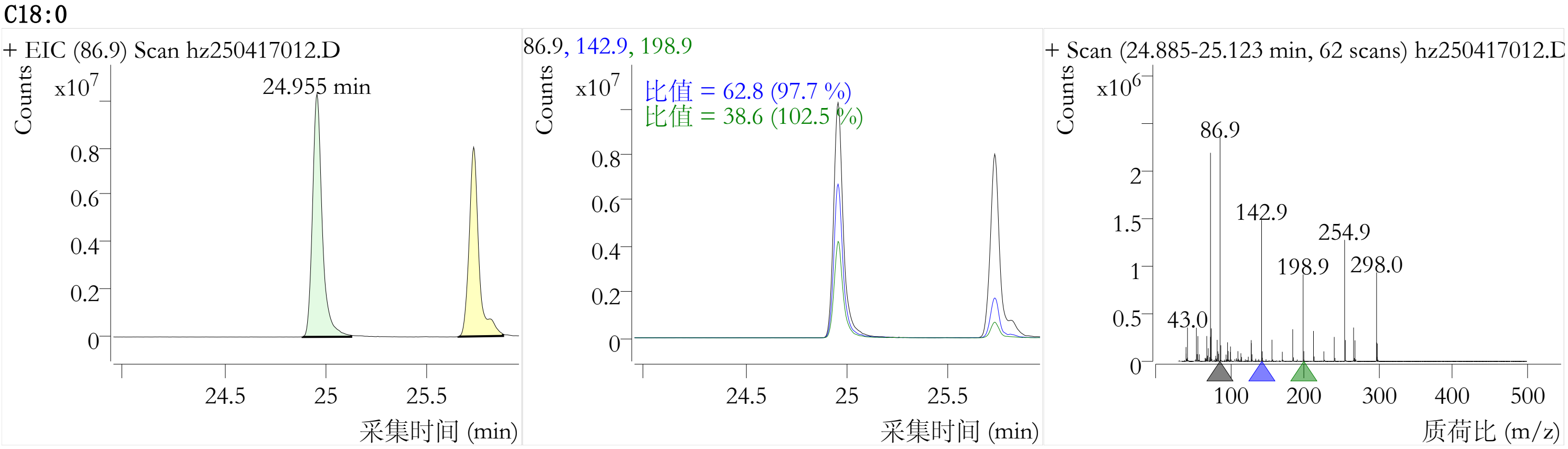

C18:1n9t

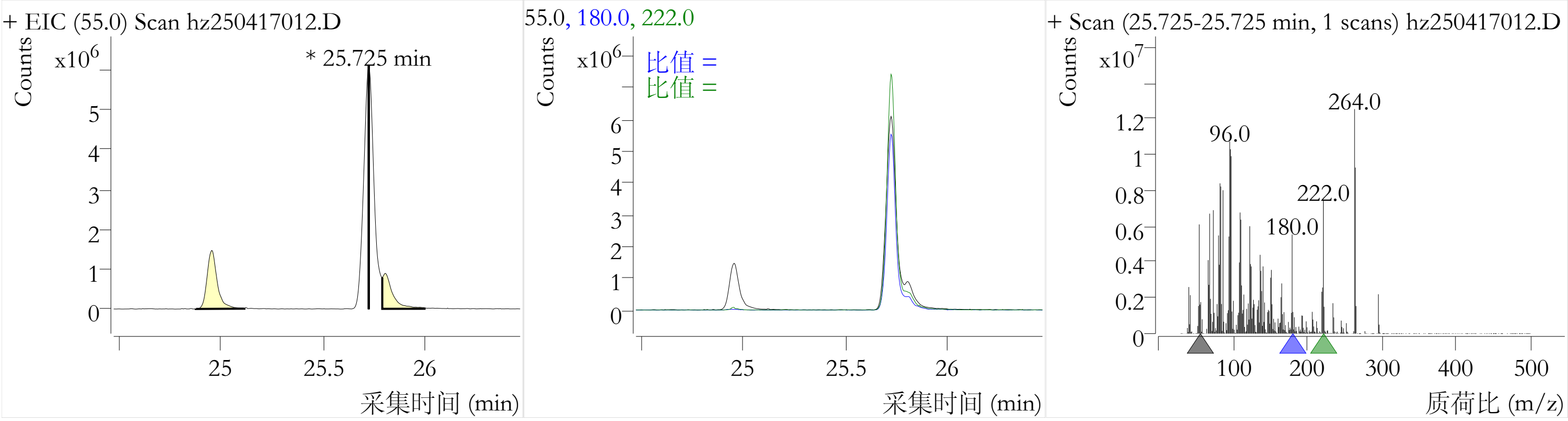

C18:1n9c

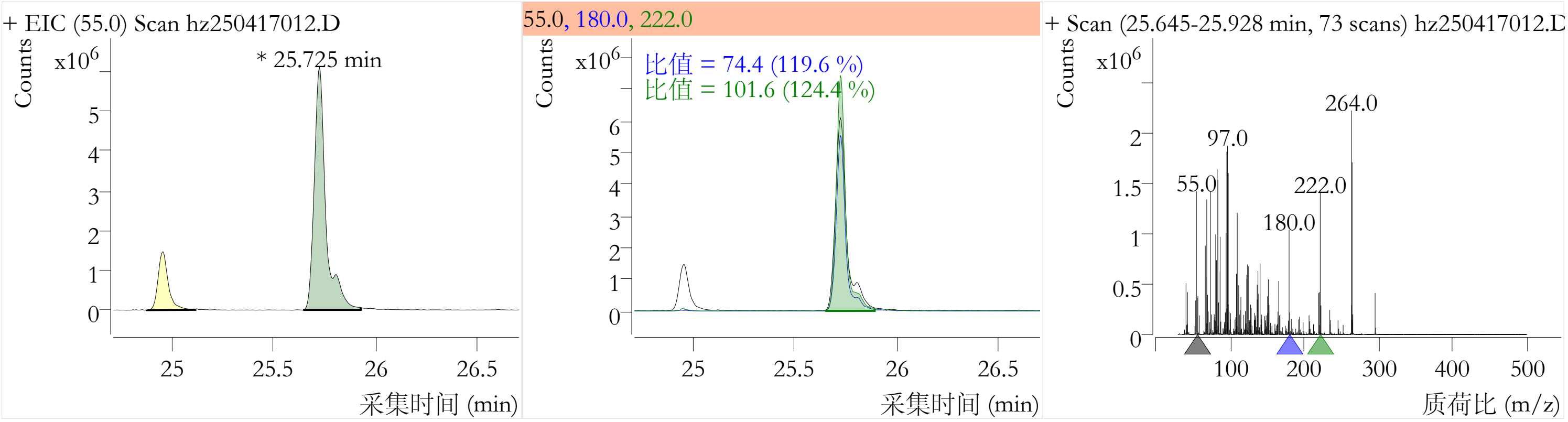

C18:2n6t

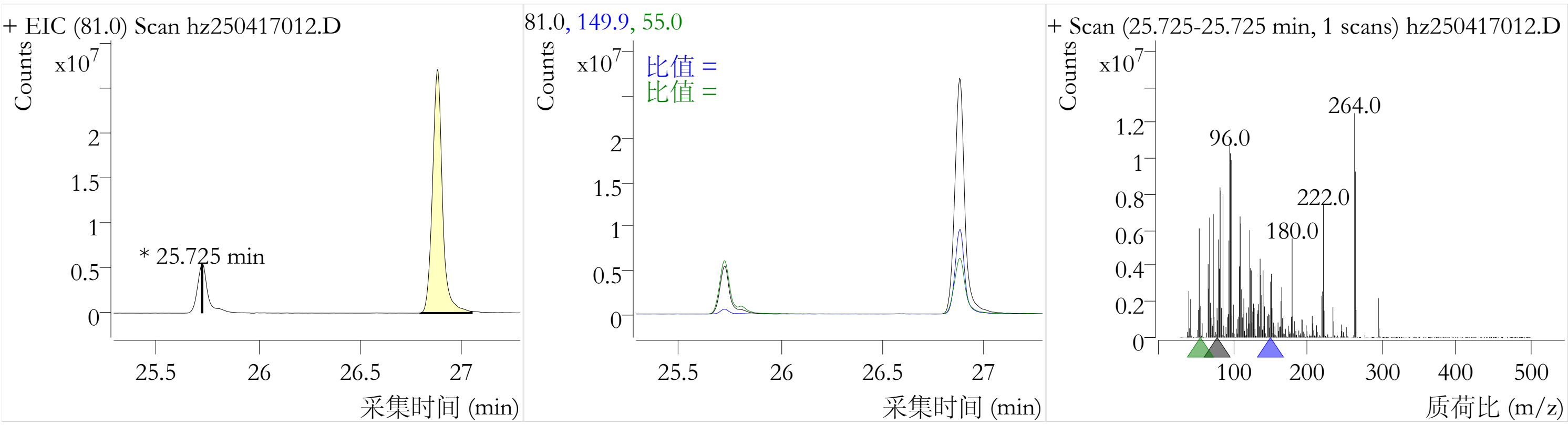

C18:2n6c

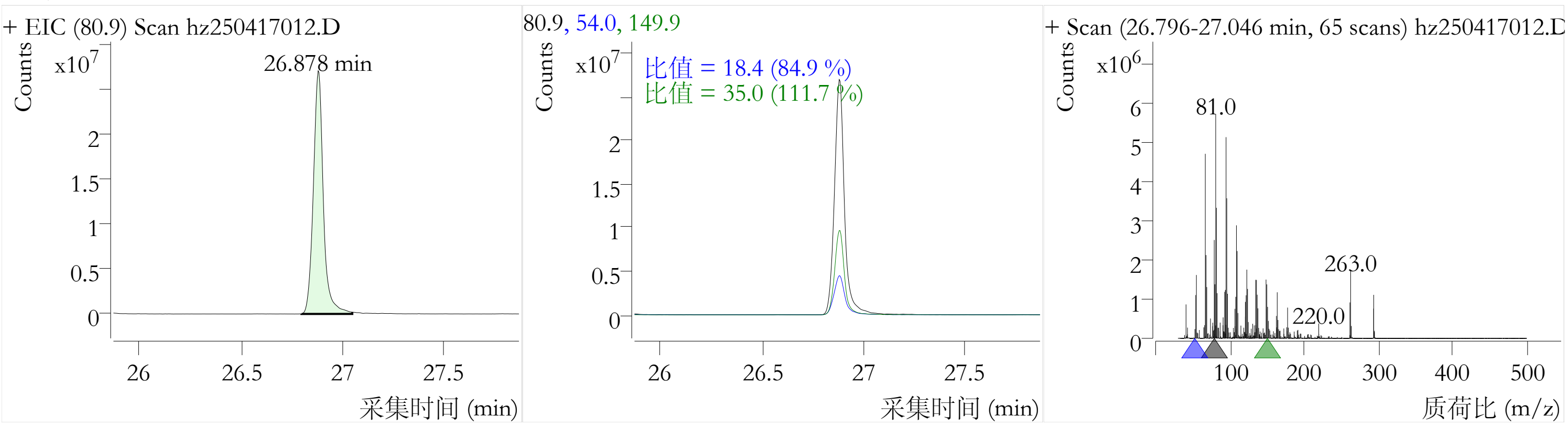

C20:0

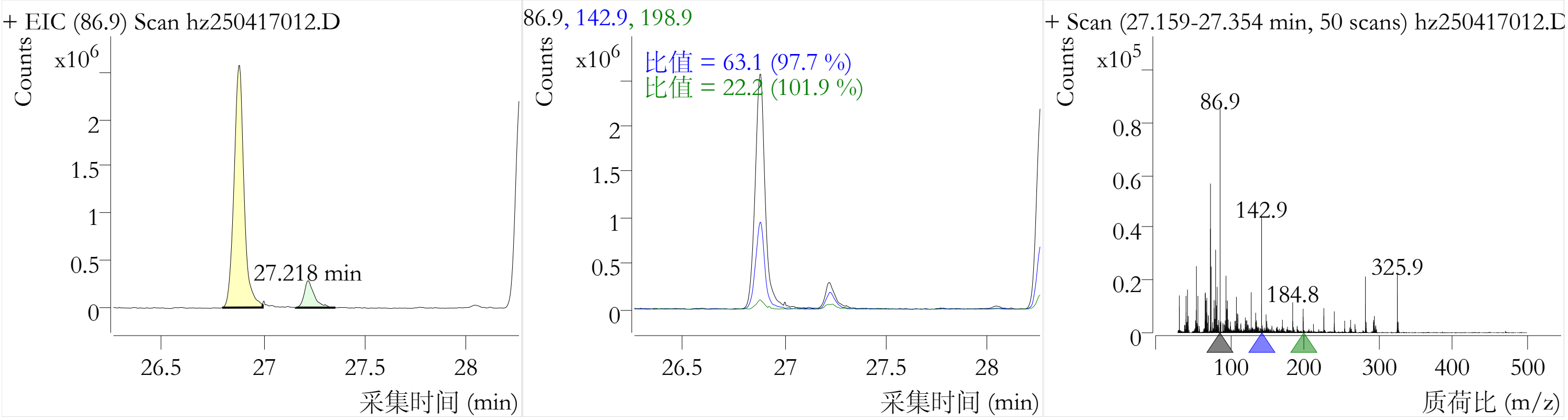

C18:3n6

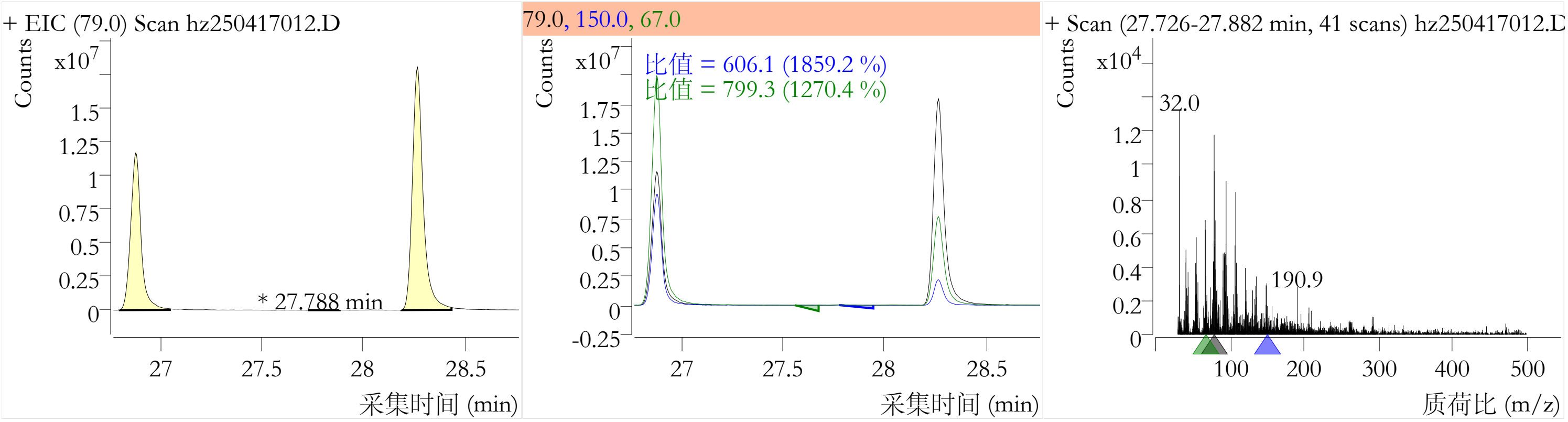

C20:1

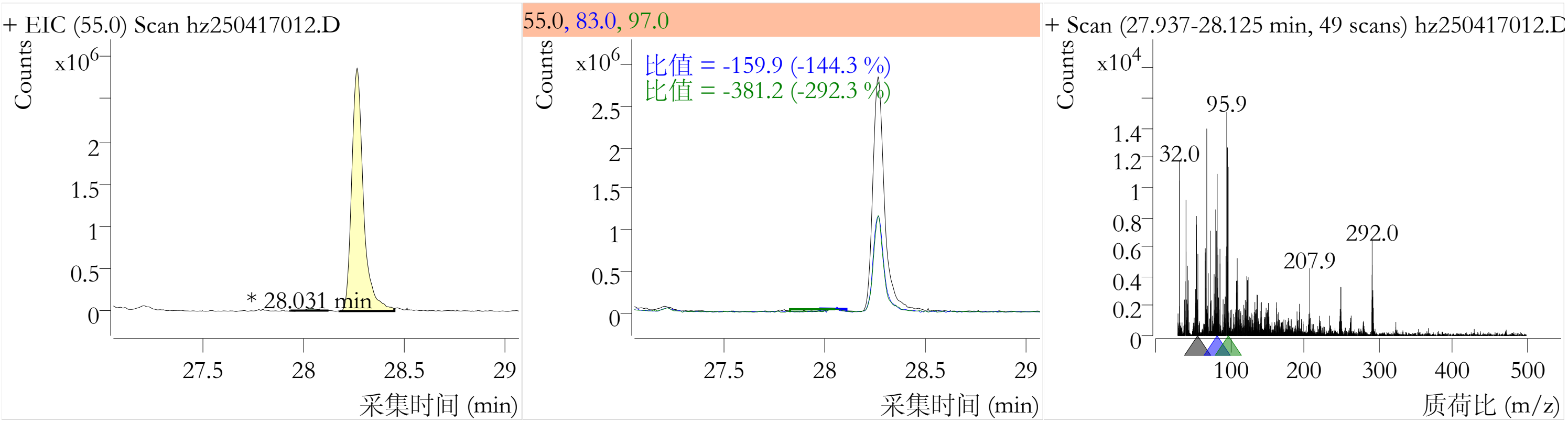

C18:3n3

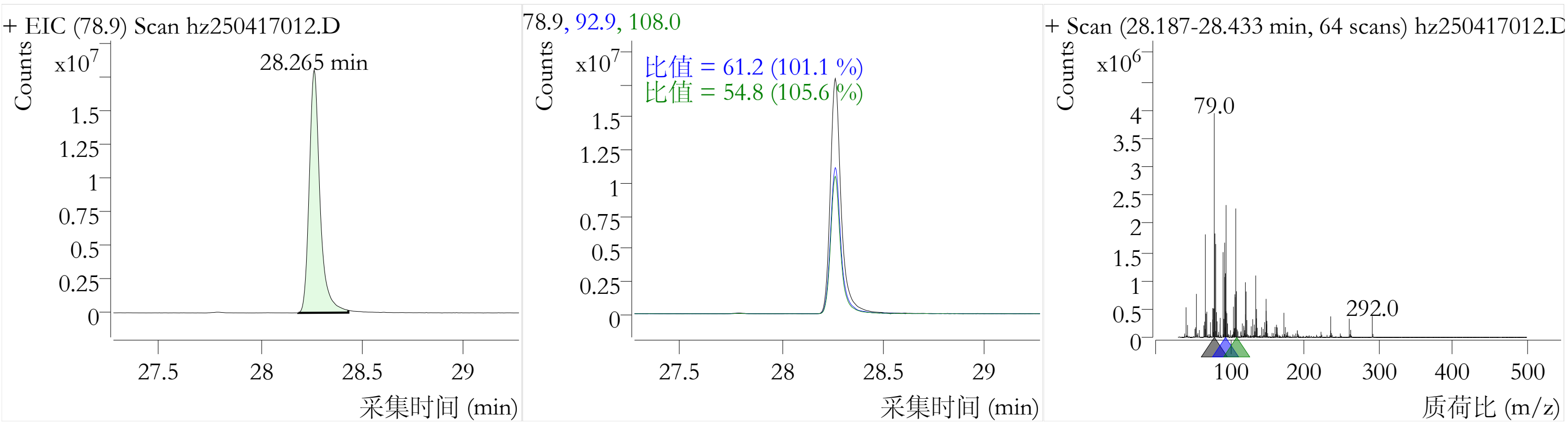

C21:0

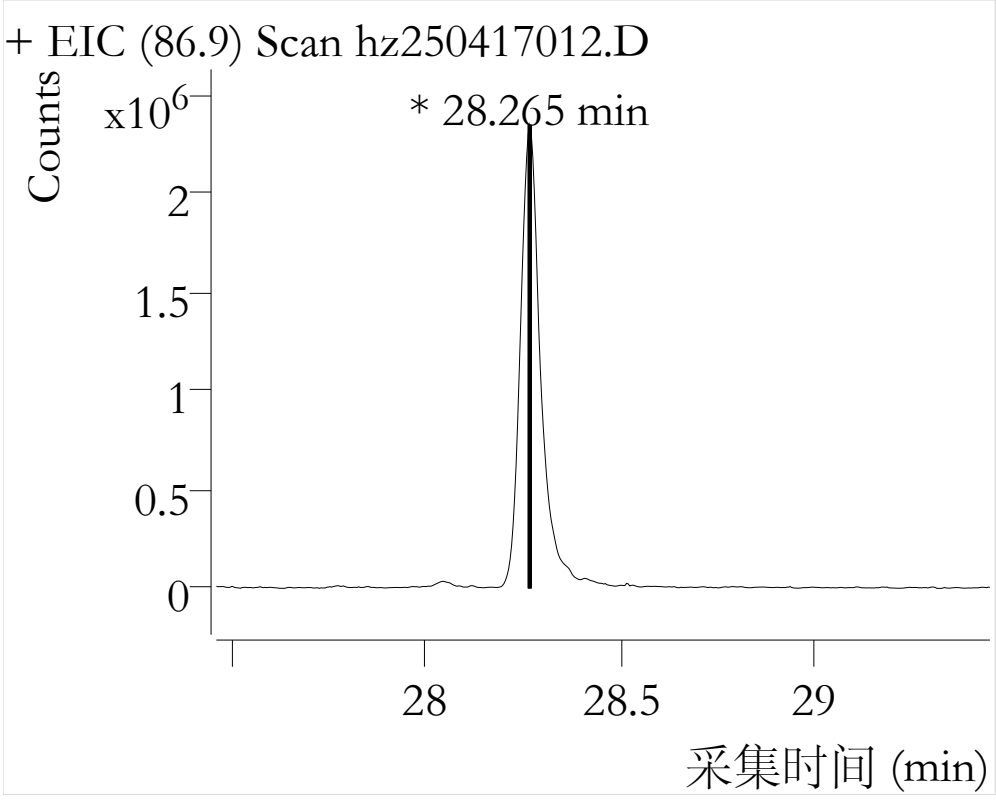

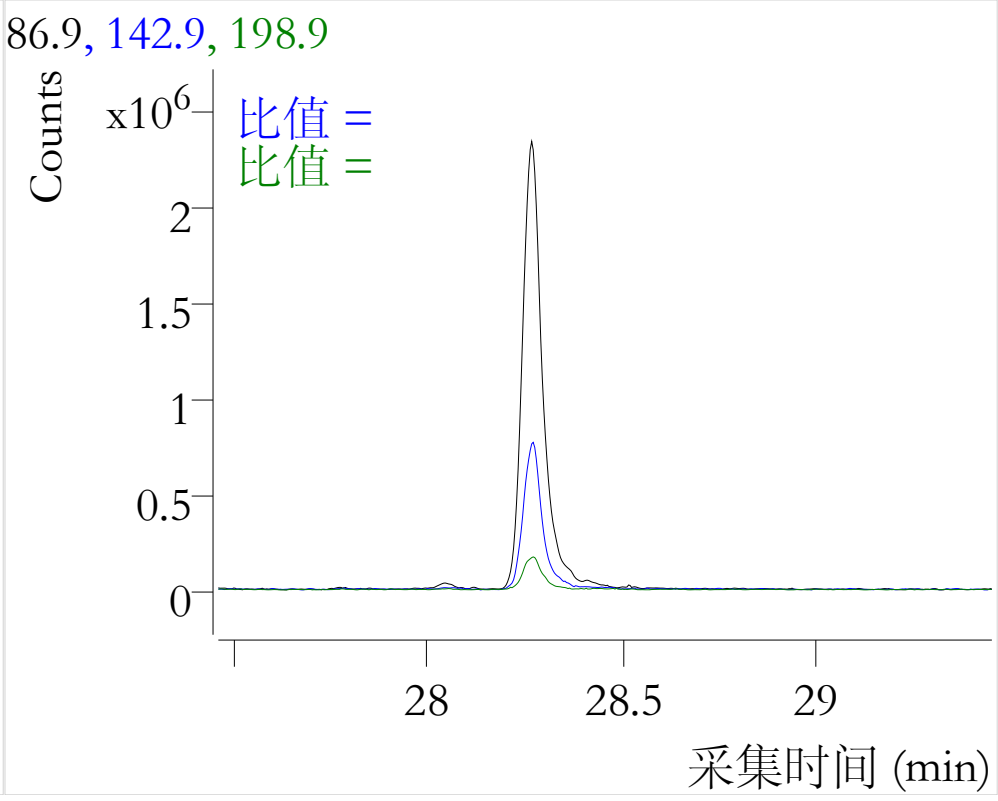

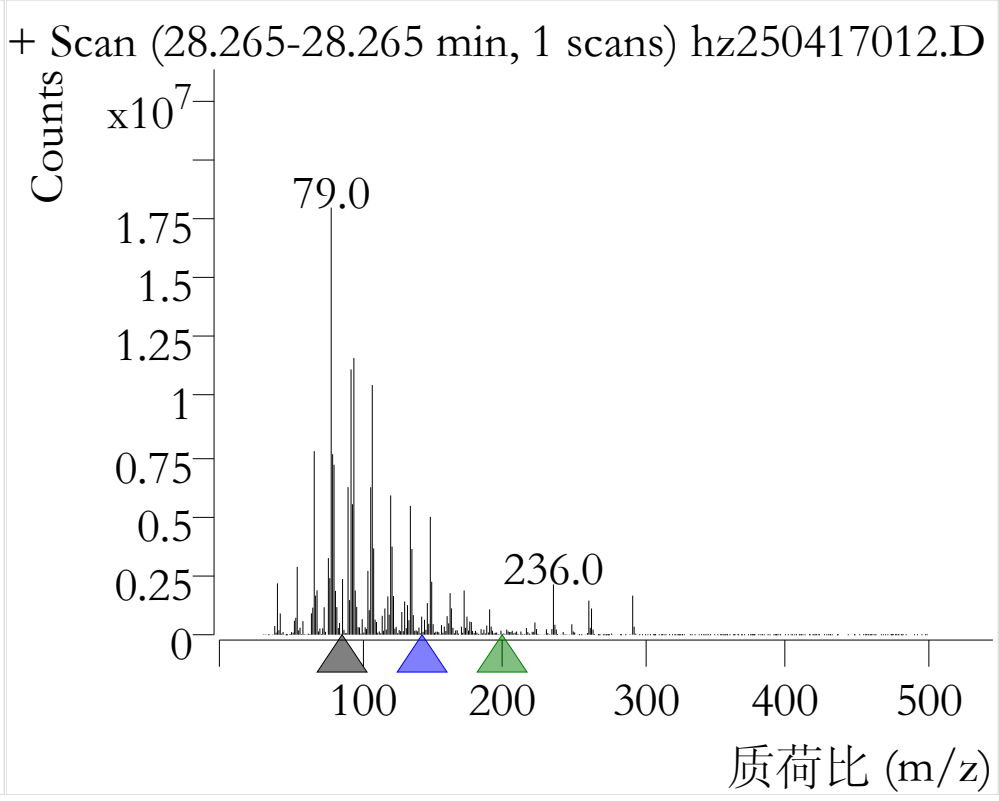

C20:2

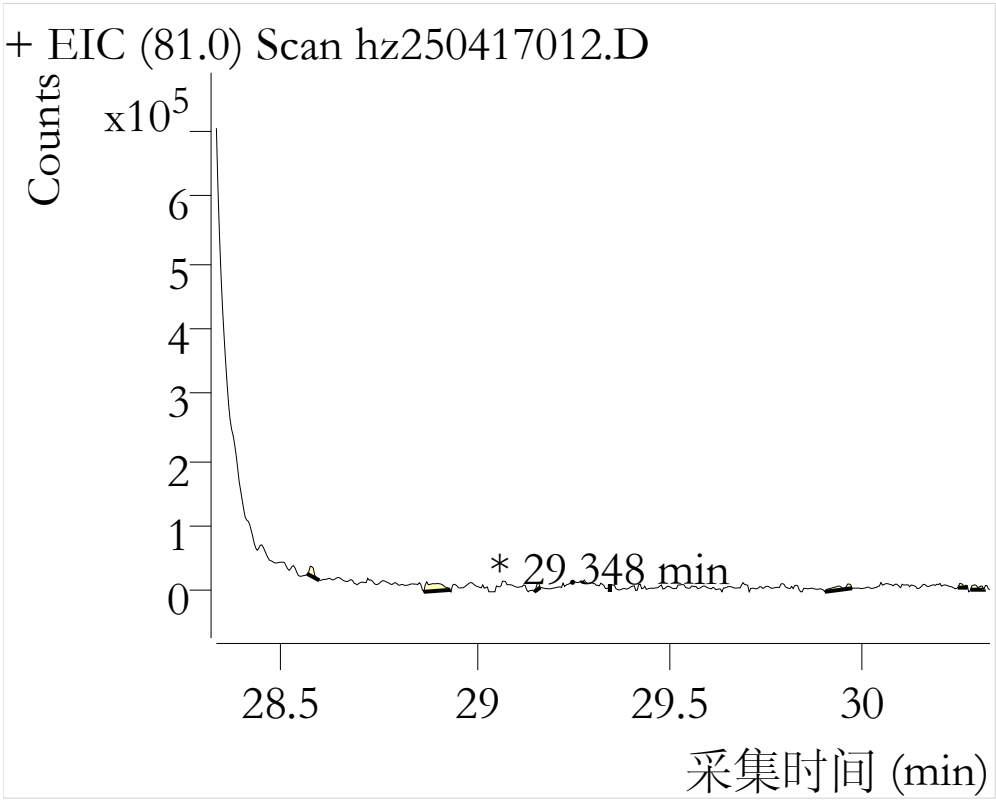

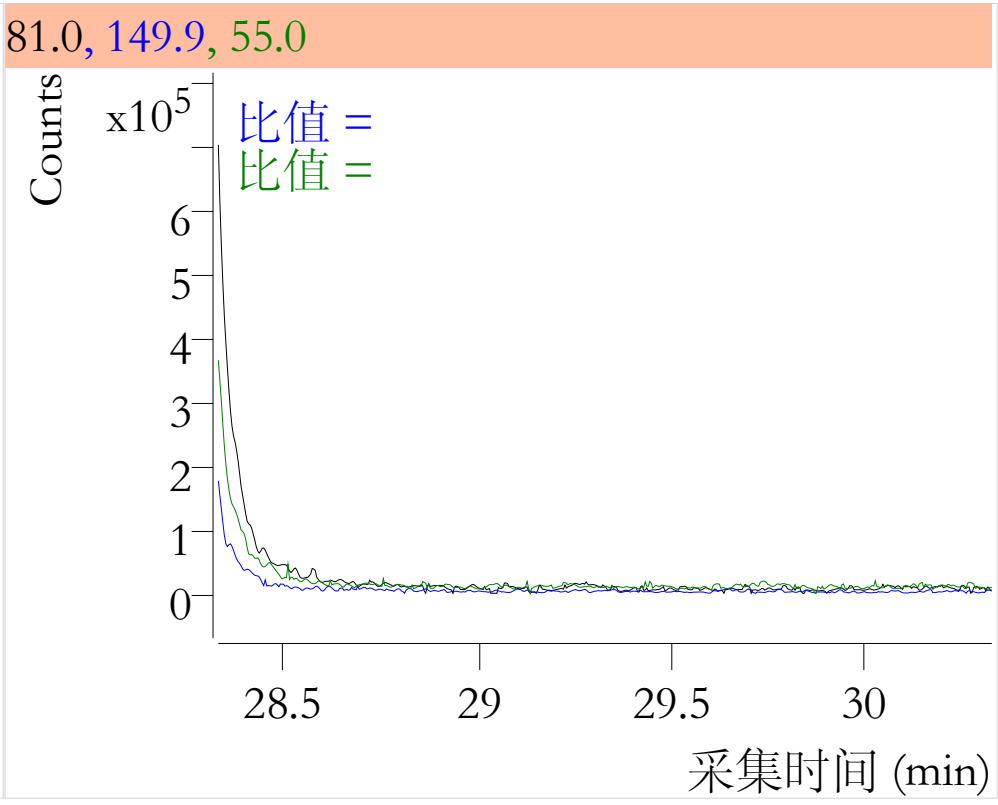

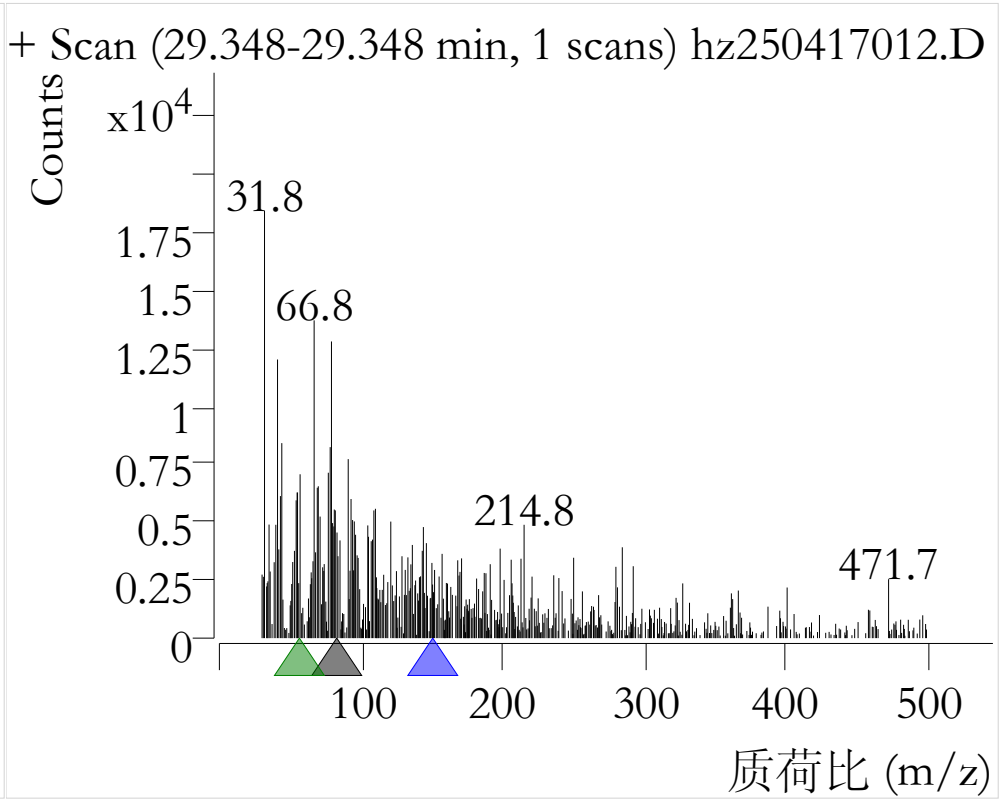

C22:0

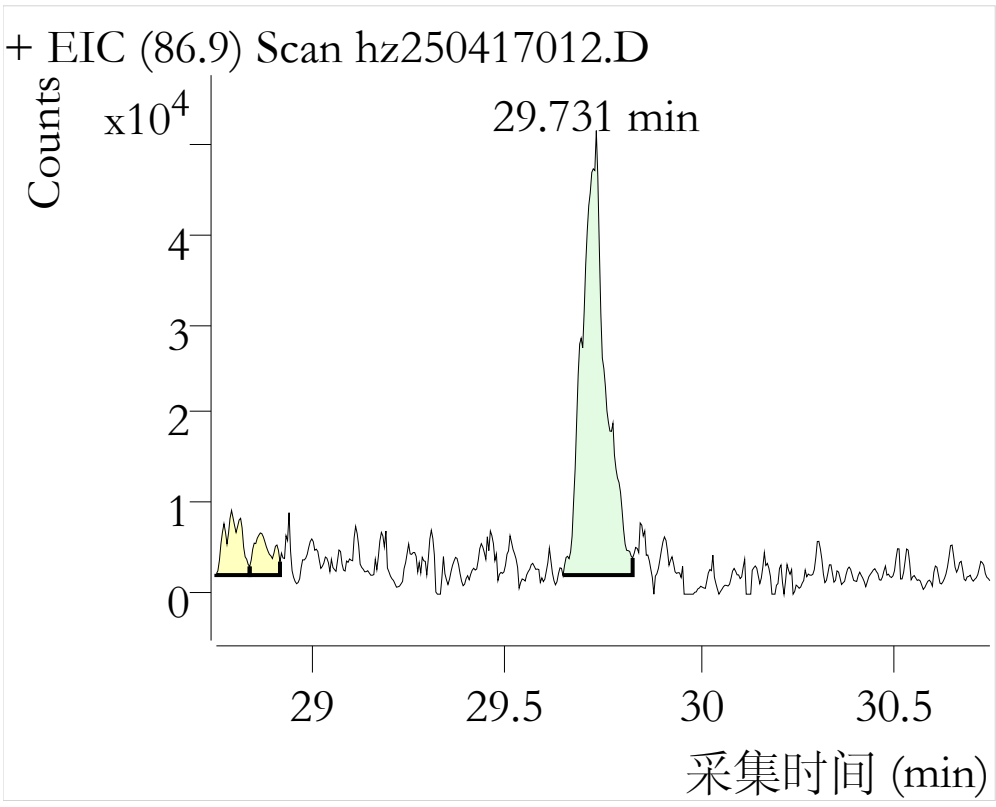

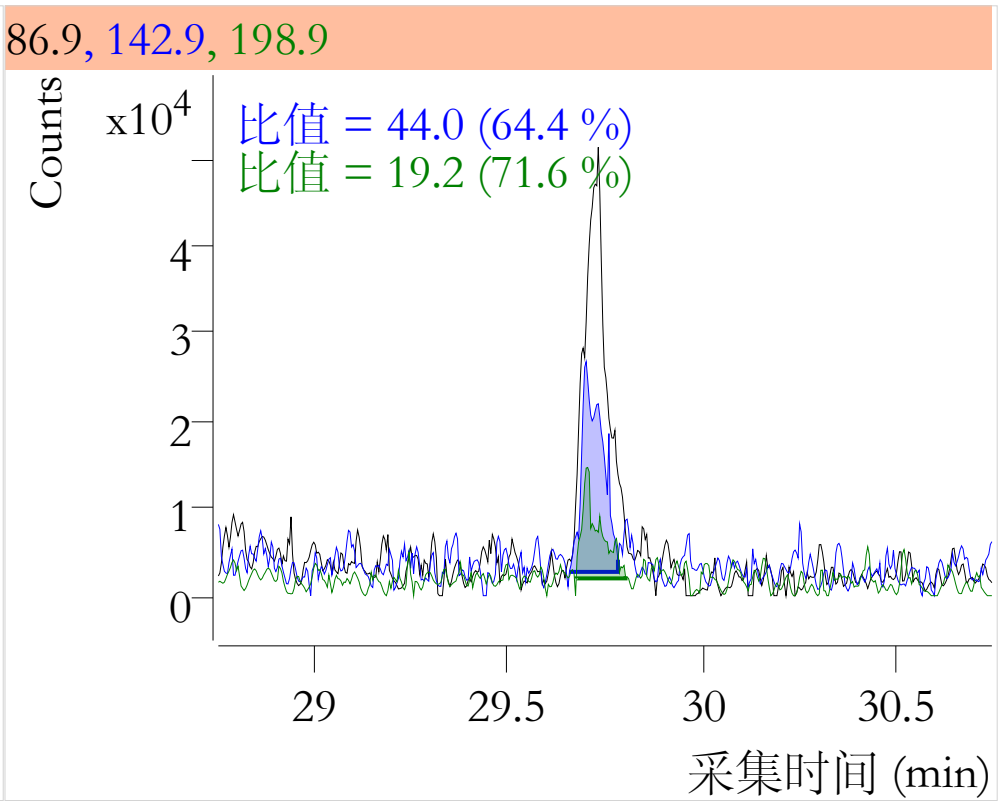

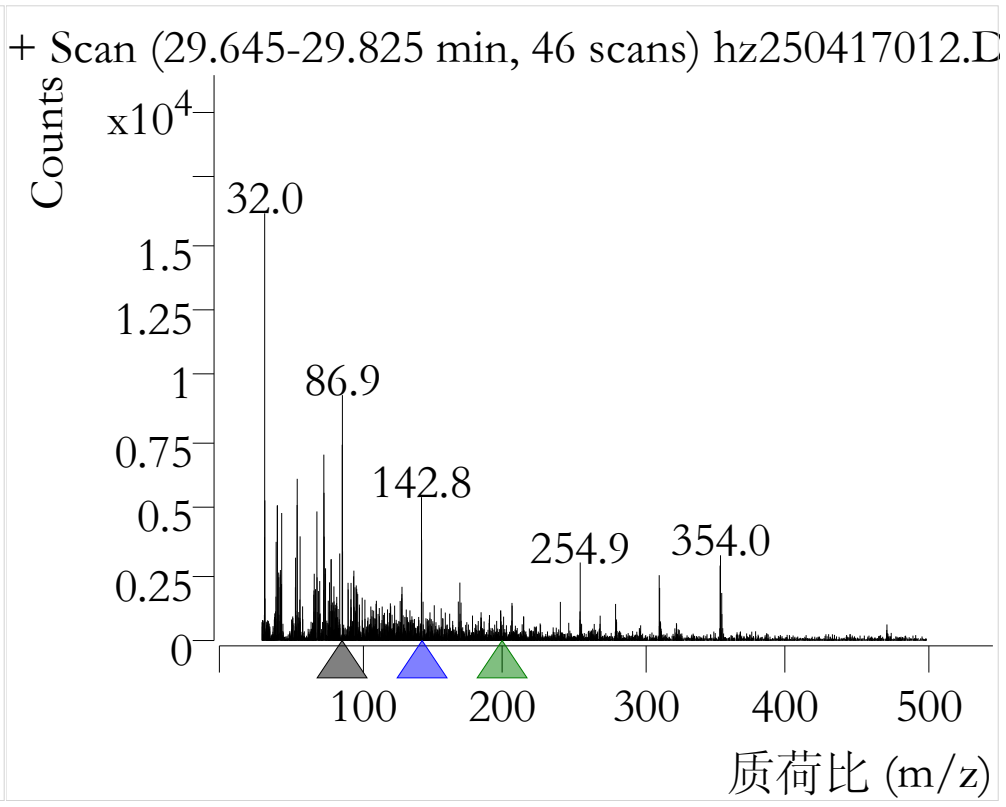

C20:3n6

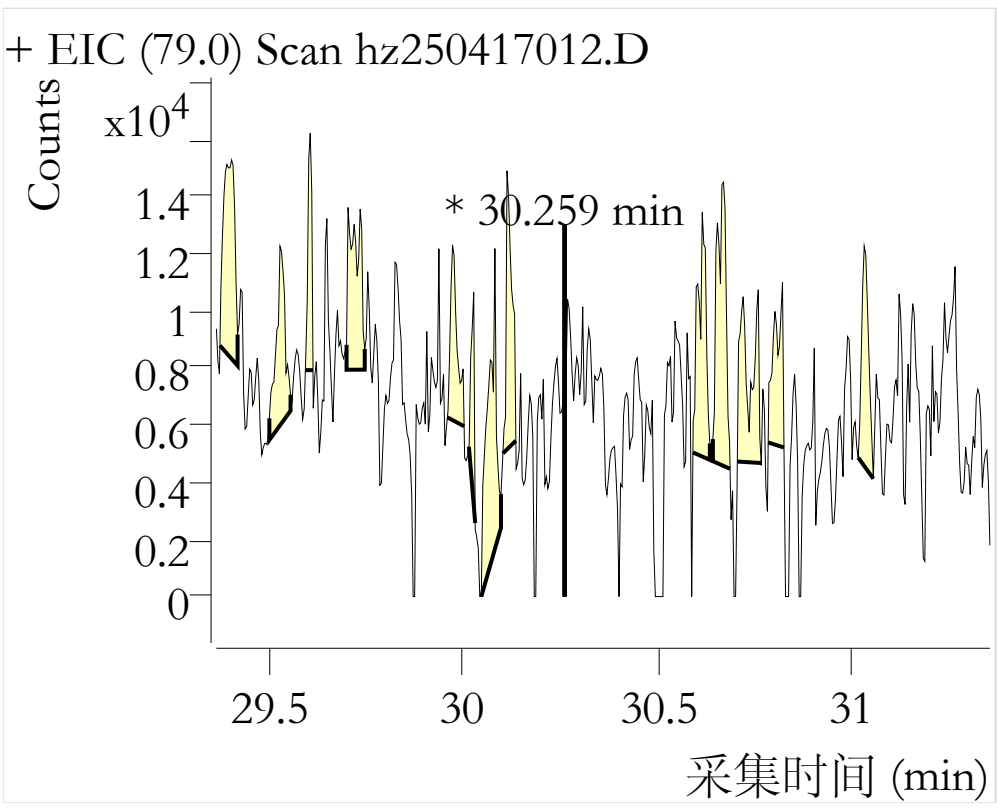

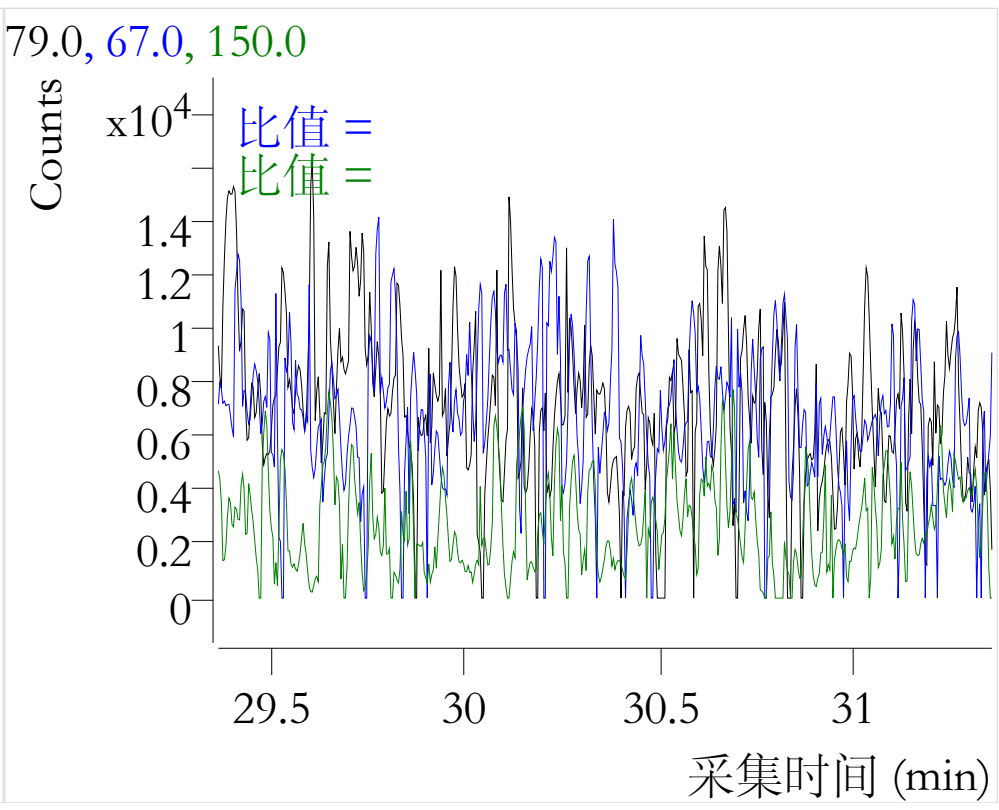

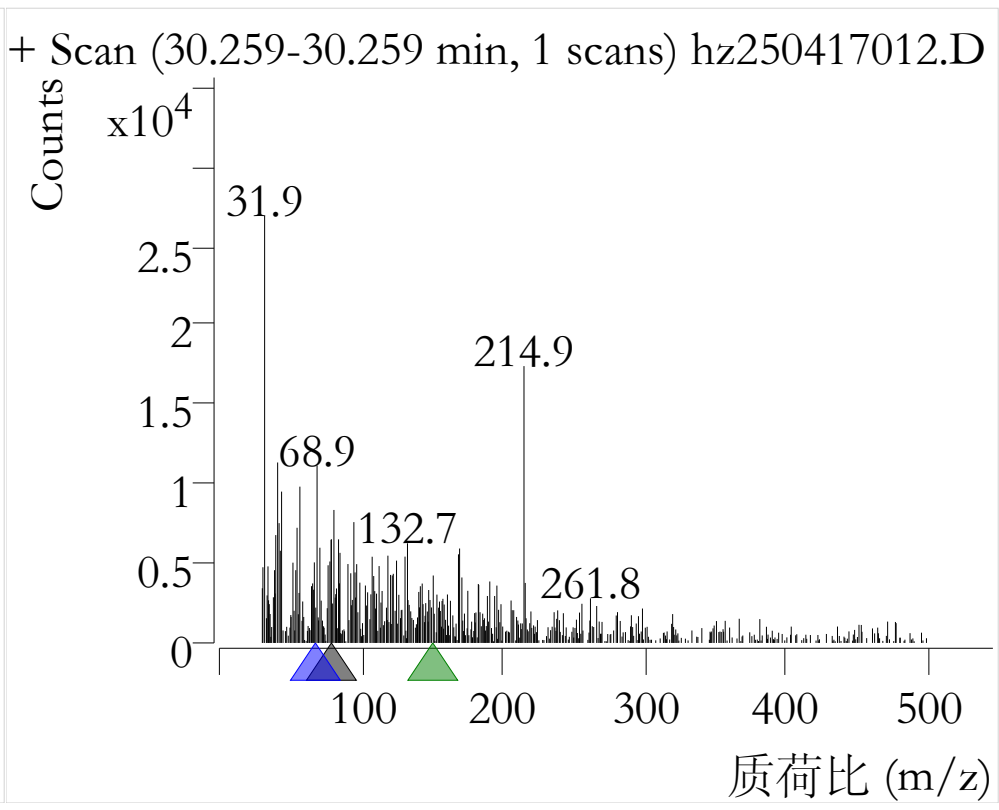

C22:1n9

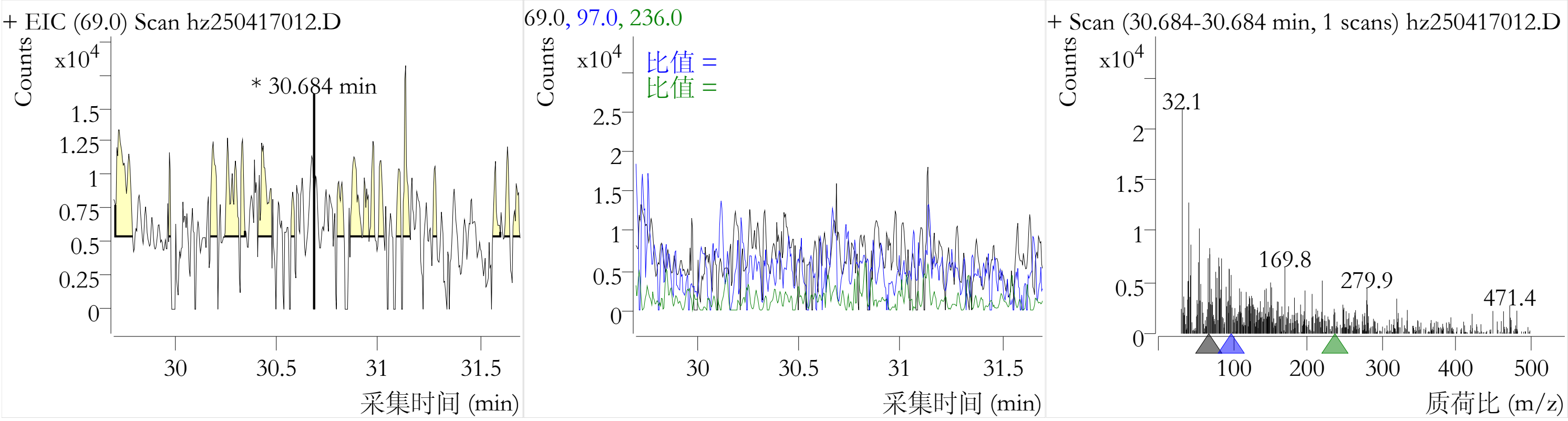

C20:3n3

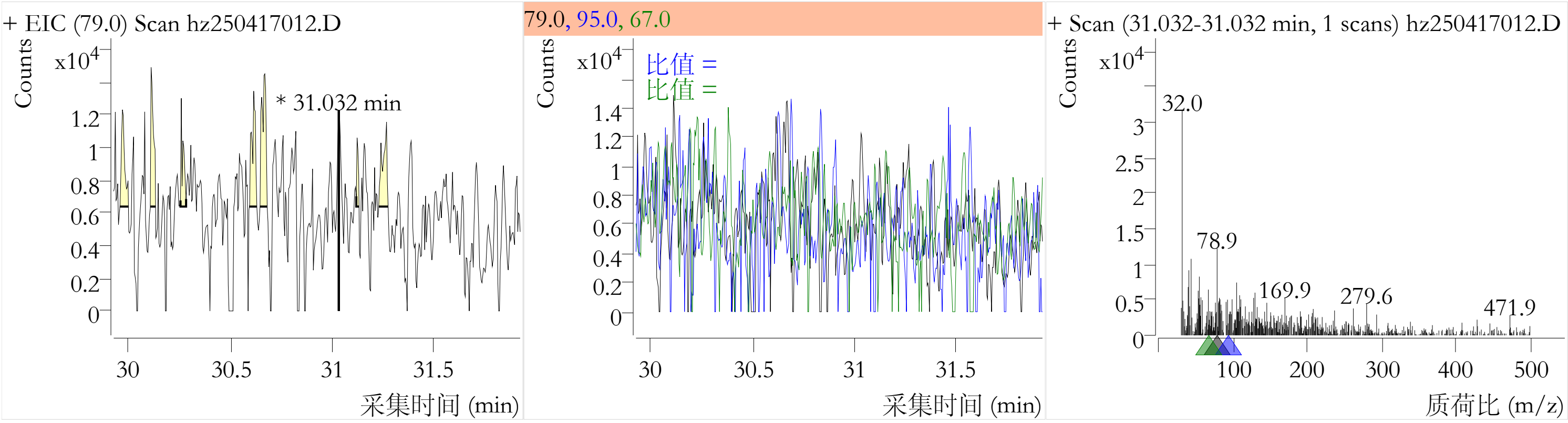

C23:0

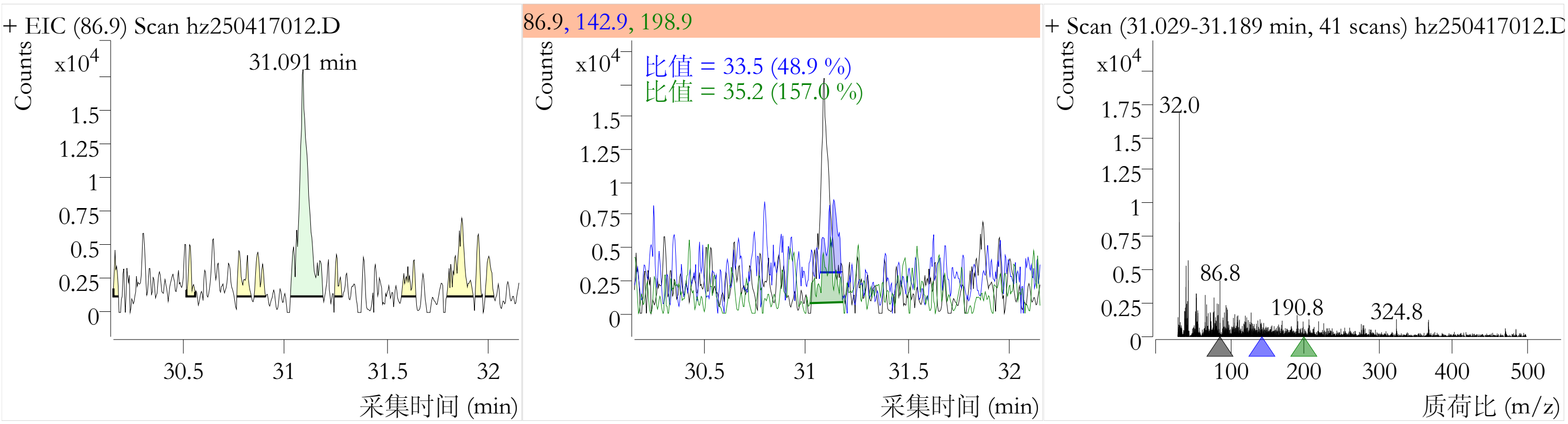

C20:4n6

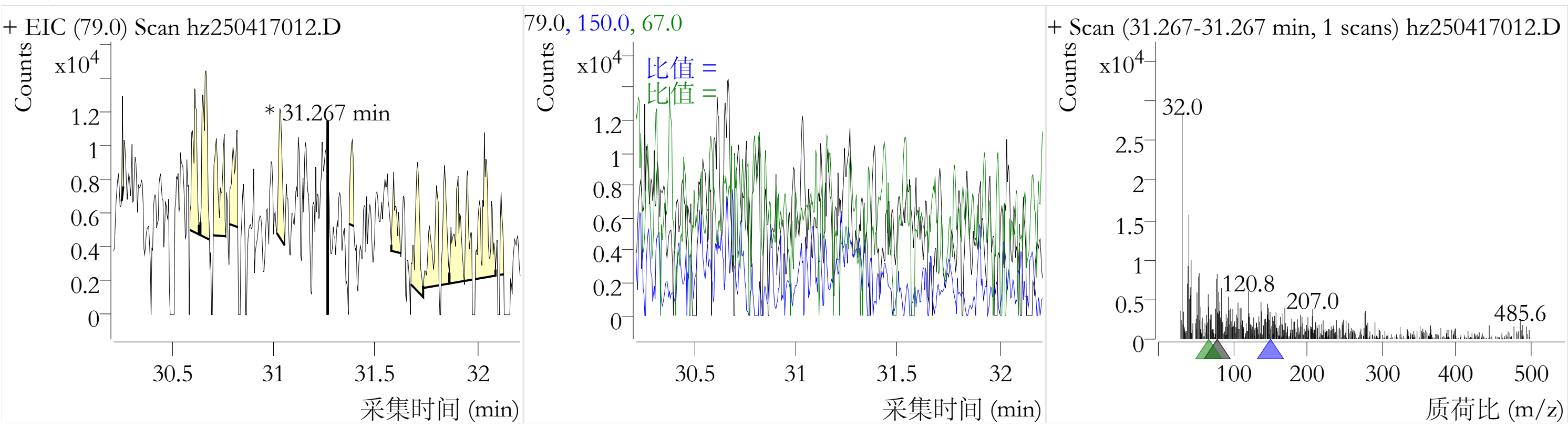

C22:2

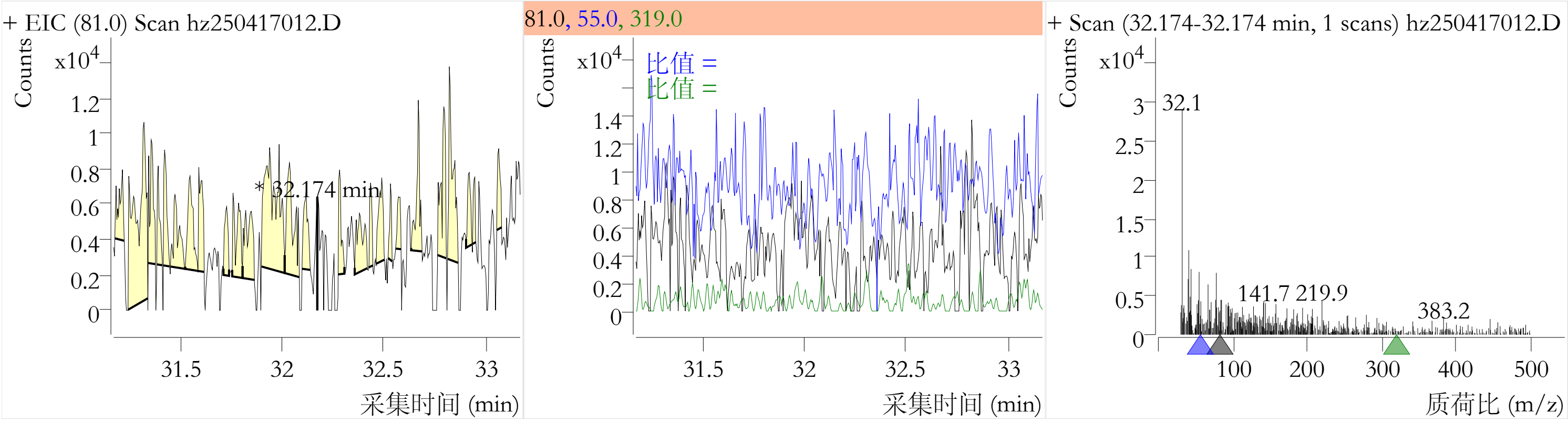

C24:0

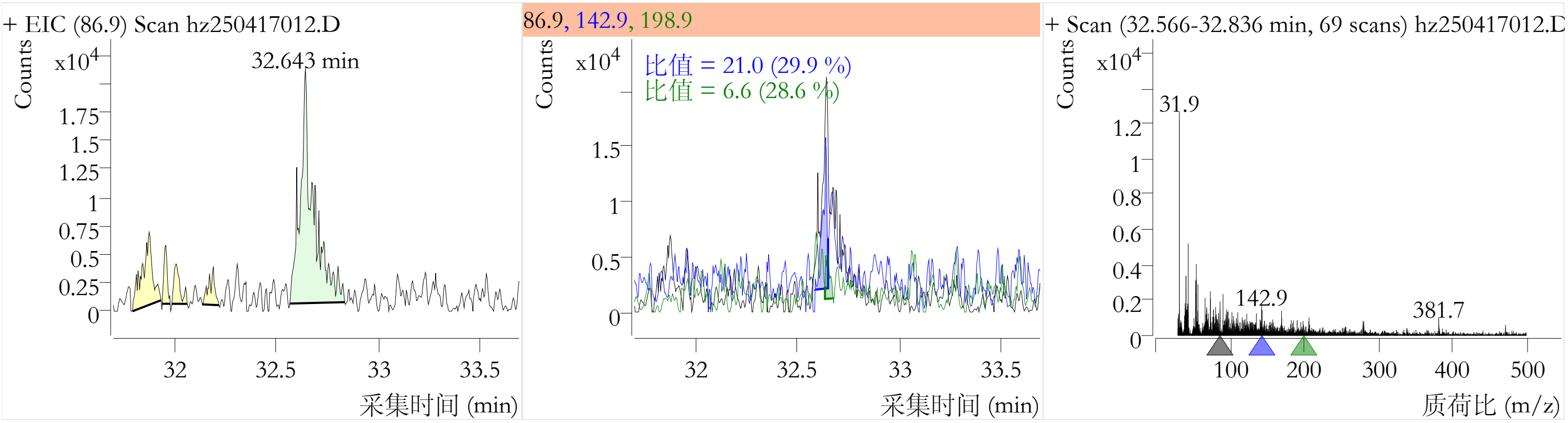

C20:5n3

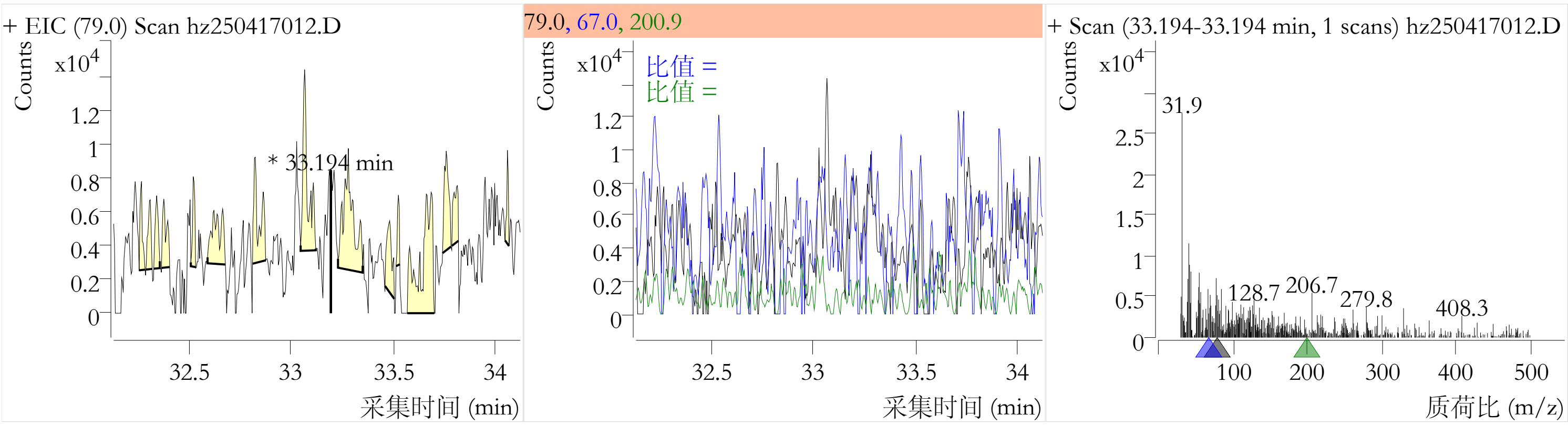

C24:1

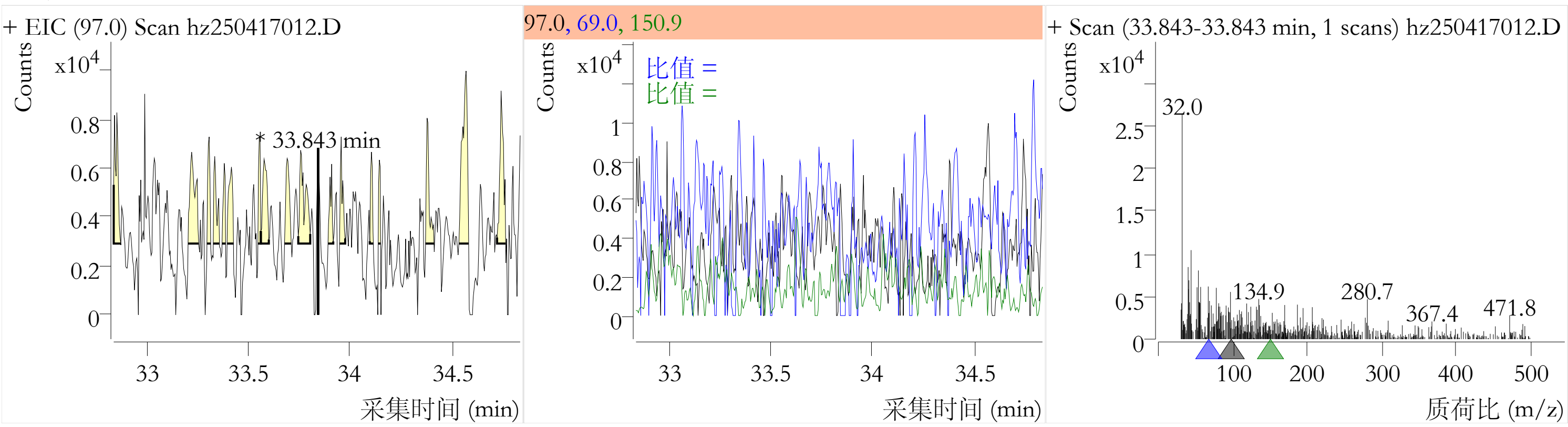

C22:6n3

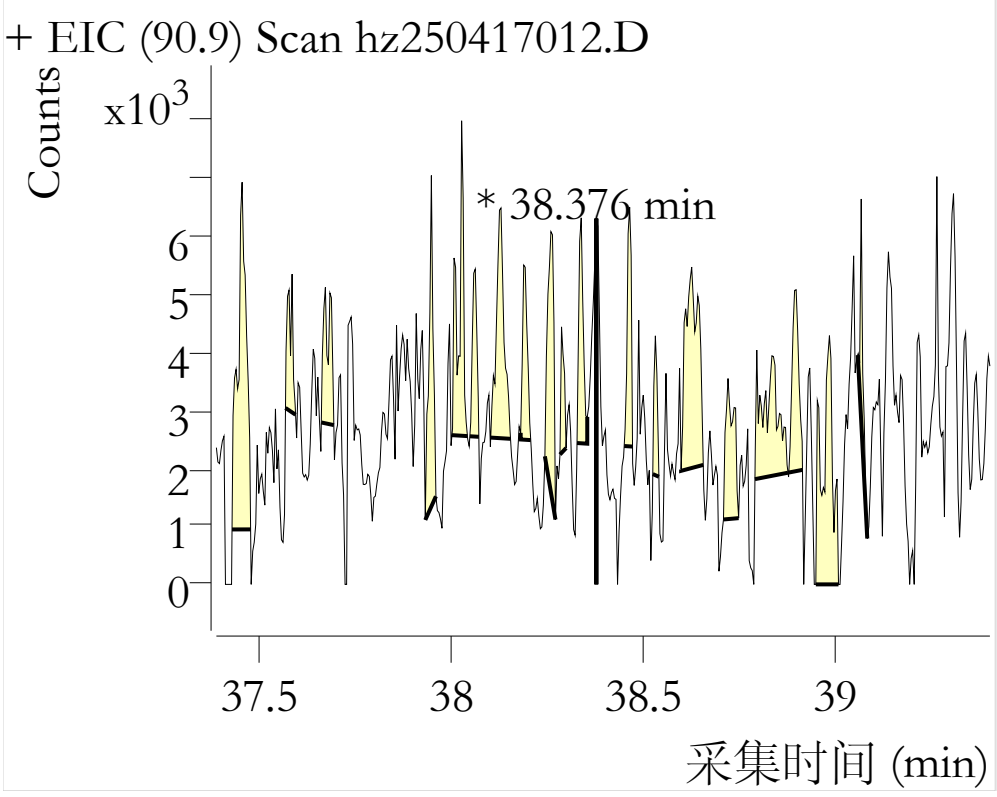
+ EIC (90.9) Scan hz250417012.D
Counts
x10^3
* 38.376 min
6
5
4
3
2
1
0
37.5
38
38.5
39
采集时间 (min)

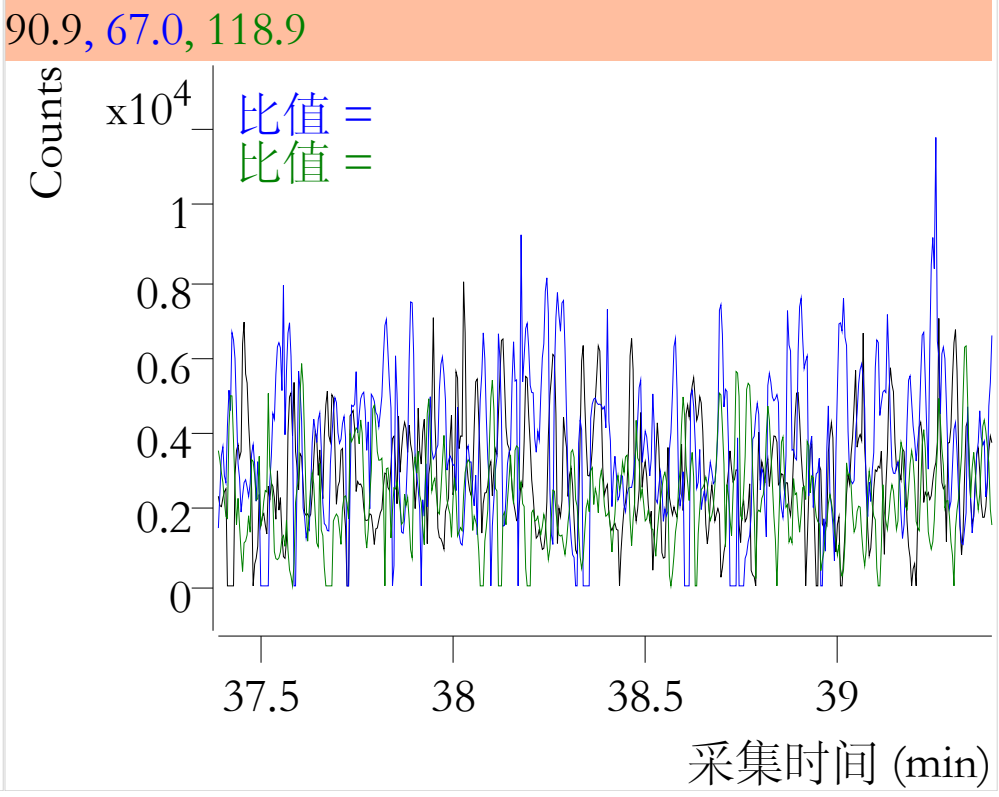
90.9, 67.0, 118.9
Counts
x10^4
比值 =
比值 =
1
0.8
0.6
0.4
0.2
0
37.5
38
38.5
39
采集时间 (min)

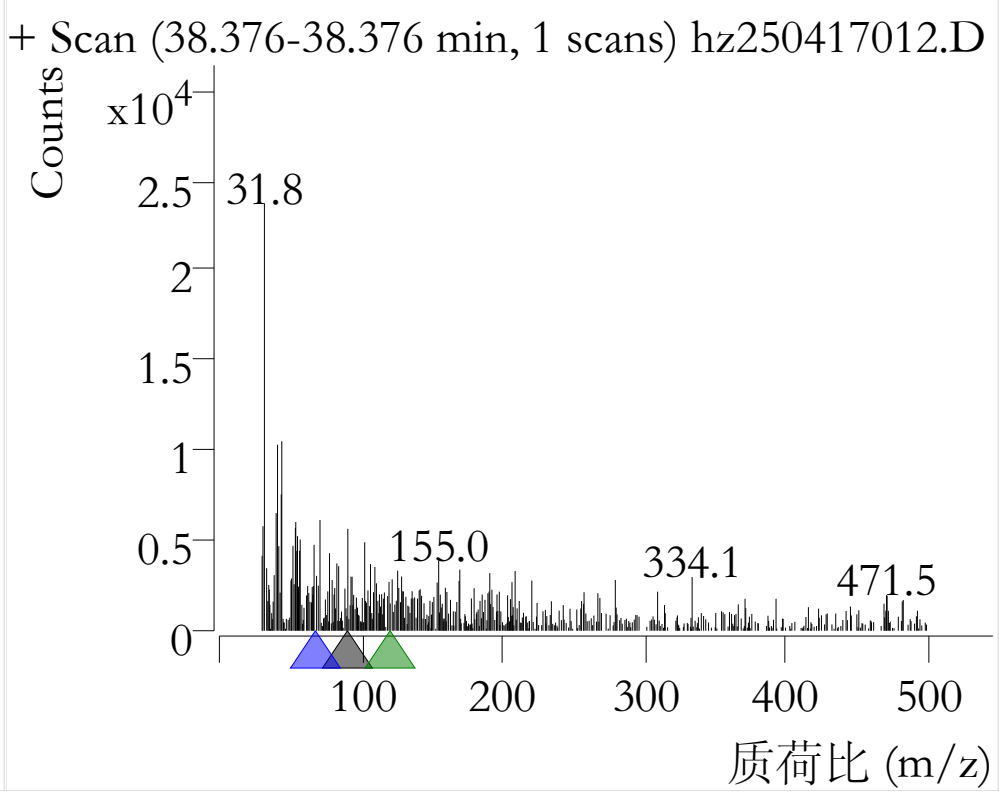
+ Scan (38.376-38.376 min, 1 scans) hz250417012.D
Counts
x10^4
2.5
31.8
2
1.5
1
0.5
155.0
334.1
471.5
0
100
200
300
400
500
质荷比 (m/z)

定量分析报告（根据样品）

Agilent Technologies

**采集日期时间**	2025/5/13 16:09:58	**数据文件**	hz250417013.D
**类型**	样品	**名称**	hz250417013
**稀释**	1	**采集方法文件**	FAME-TEST-SCAN

样品色谱图

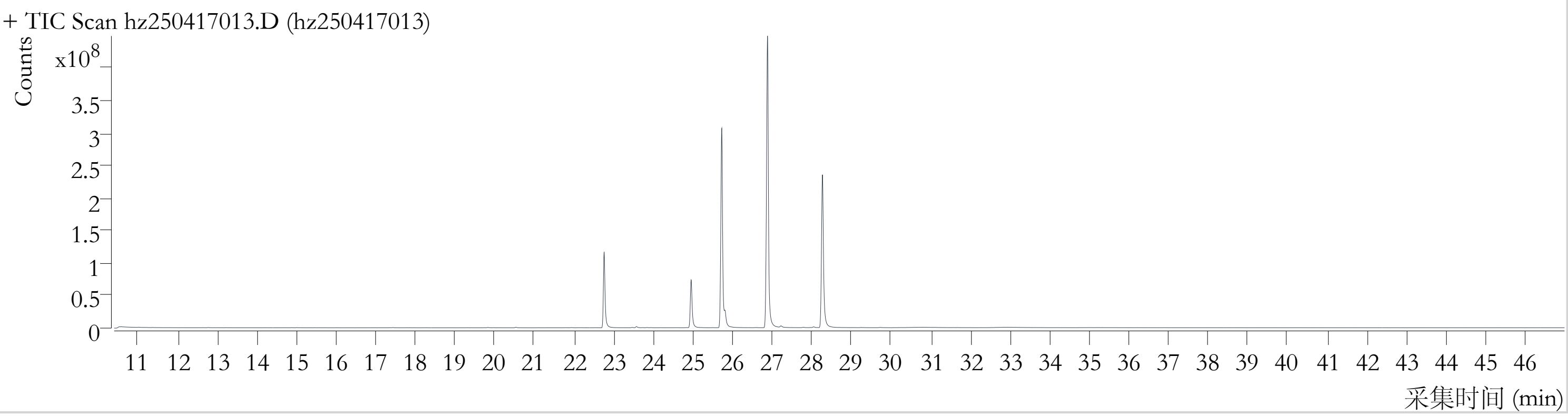

名称	RT	离子对	响应	峰高	最终浓度 单位	比值
C4:0	10.521	74.0	0	2503.04	ND ug/ml	
C6:0	12.030	74.0	0	2585.72	ND ug/ml	
C8:0	13.832	74.0	0	1481.71	ND ug/ml	
C10:0	16.020	74.0	0	4930.89	ND ug/ml	
C11:0	17.142	74.0	0	6204.75	ND ug/ml	
C12:0	18.252	74.0	36567	9858.22	0.0856 ug/ml	27.8
C13:0	19.397	74.0	0	5211.50	ND ug/ml	
C14:0	20.539	74.0	455071	145839.10	1.1299 ug/ml	46.2
C15:0	21.652	74.0	11056	5486.86	0.0286 ug/ml	85.5
C15:1	22.763	55.0	0	2029403.25	ND ug/ml	
C16:0	22.763	87.0	46709842	15456399.44	130.0010 ug/ml	57.3
C16:1	23.579	55.0	194931	64871.29	2.6385 ug/ml	73.9
C17:0	23.845	74.0	120682	55190.90	0.3512 ug/ml	65.5
C17:1	24.955	55.0	0	1323369.88	ND ug/ml	
C18:0	24.955	86.9	29763782	8840620.76	91.0578 ug/ml	63.5
C18:1n9t	25.725	55.0	0	6174352.50	ND ug/ml	
C18:1n9c	25.725	55.0	24986888	6156449.47	359.4810 ug/ml	104.8
C18:2n6t	25.729	81.0	0	5680310.50	ND ug/ml	
C18:2n6c	26.882	80.9	95728989	28044709.21	628.4496 ug/ml	36.1
C20:0	27.222	86.9	779270	226209.72	2.3497 ug/ml	63.0
C18:3n6	27.793	79.0	201975	57805.37	1.2246 ug/ml	2813.9
C20:1	28.043	55.0	145301	38287.66	1.9456 ug/ml	153.5
C18:3n3	28.266	78.9	75572834	20269389.73	356.2991 ug/ml	61.4
C21:0	28.266	86.9	0	2687826.00	ND ug/ml	
C20:2	29.309	81.0	0	15282.00	ND ug/ml	
C22:0	29.712	86.9	152561	30601.12	0.5049 ug/ml	53.6
C20:3n6	30.450	79.0	0	15074.76	ND ug/ml	
C22:1n9	30.673	69.0	0	28518.28	ND ug/ml	
C20:3n3	30.880	79.0	0	17183.13	ND ug/ml	
C23:0	31.103	86.9	59214	12784.94	0.1885 ug/ml	67.9
C20:4n6	31.236	79.0	0	10688.19	ND ug/ml	
C22:2	32.178	81.0	0	9119.13	ND ug/ml	
C24:0	32.639	86.9	70102	21218.17	0.2576 ug/ml	63.0
C20:5n3	32.971	79.0	0	8930.50	ND ug/ml	
C24:1	33.820	97.0	0	8168.21	ND ug/ml	

C4:0

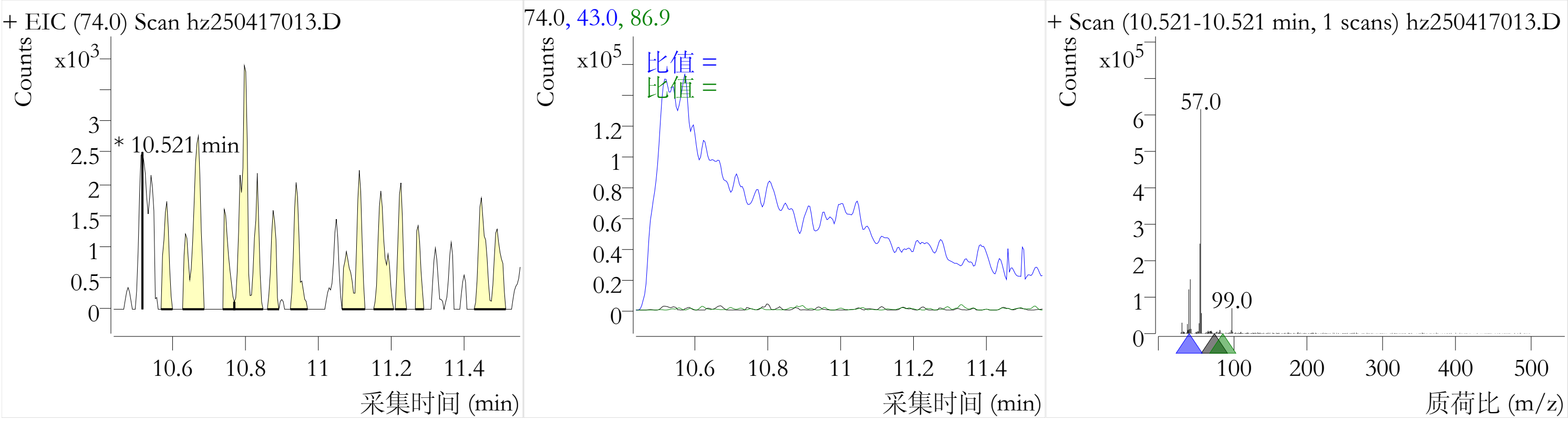

C6:0

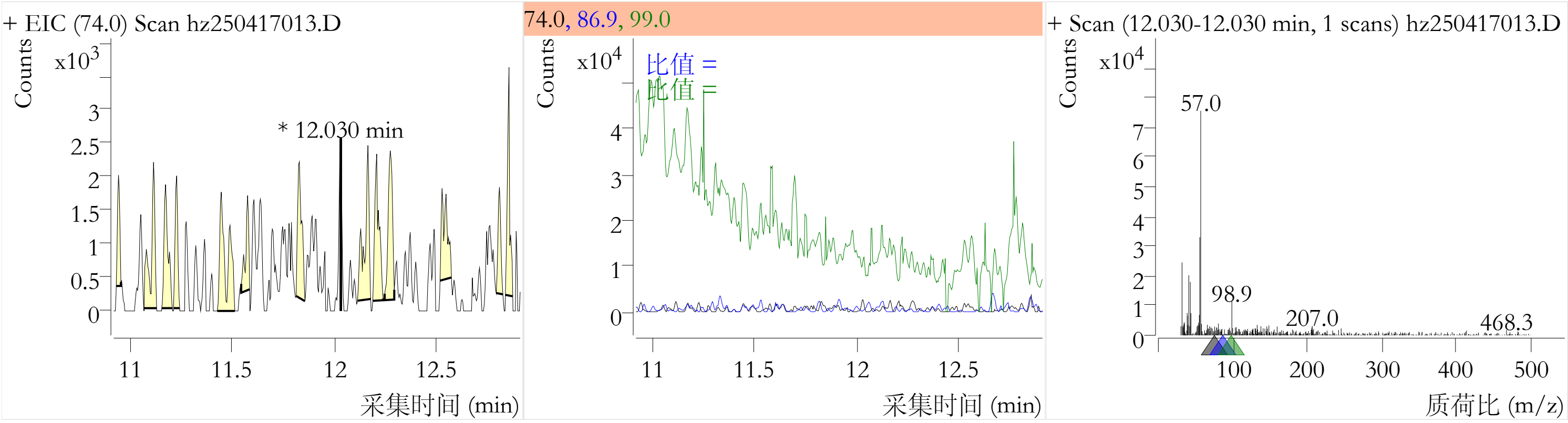

C8:0

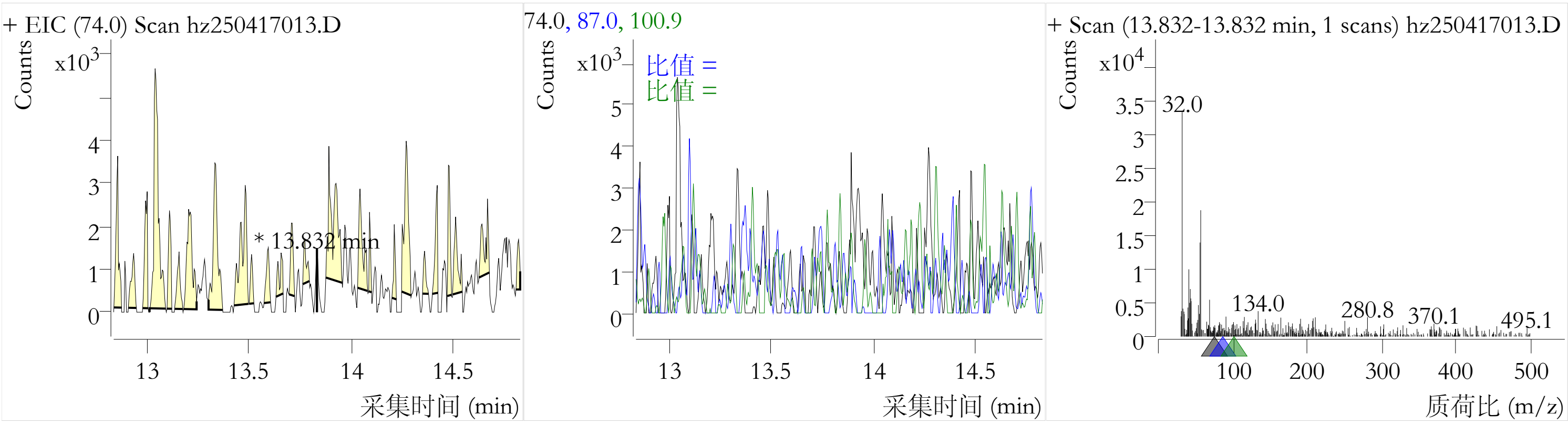

C10:0

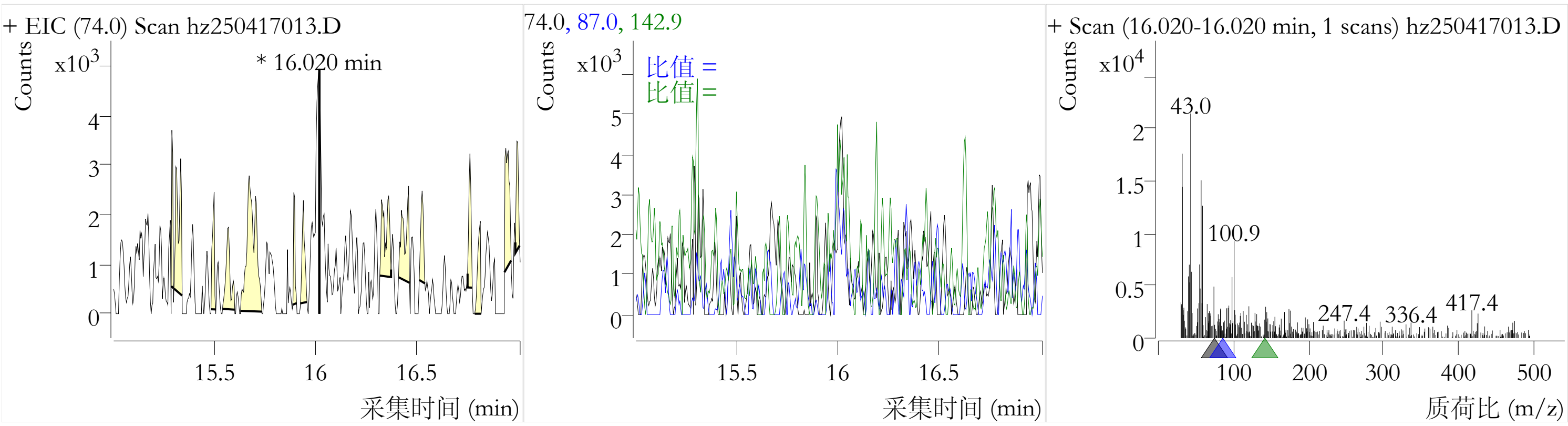

C11:0

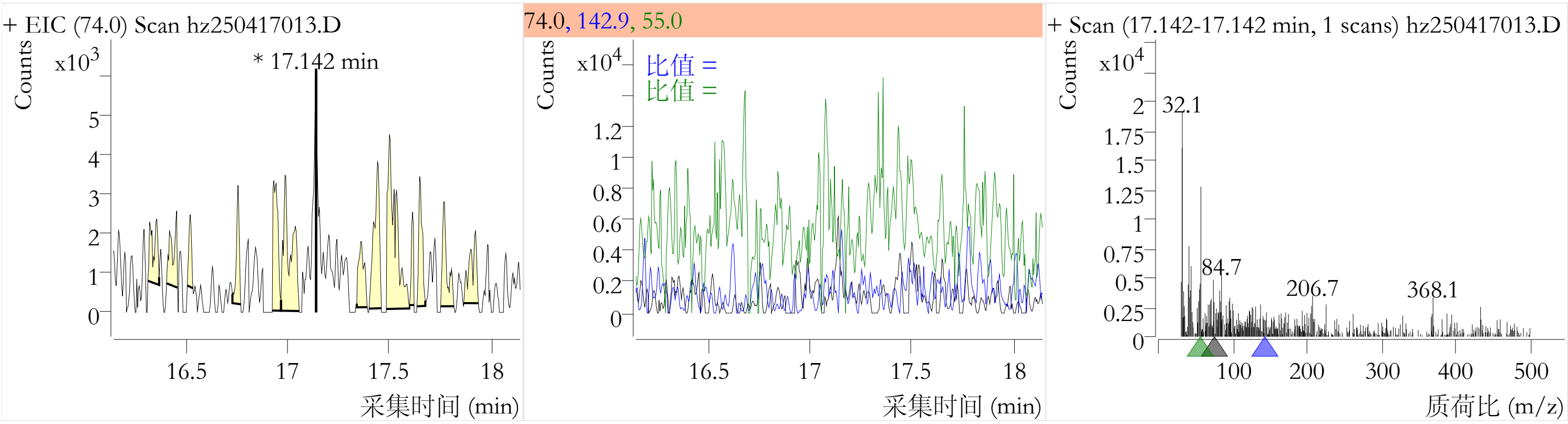

C12:0

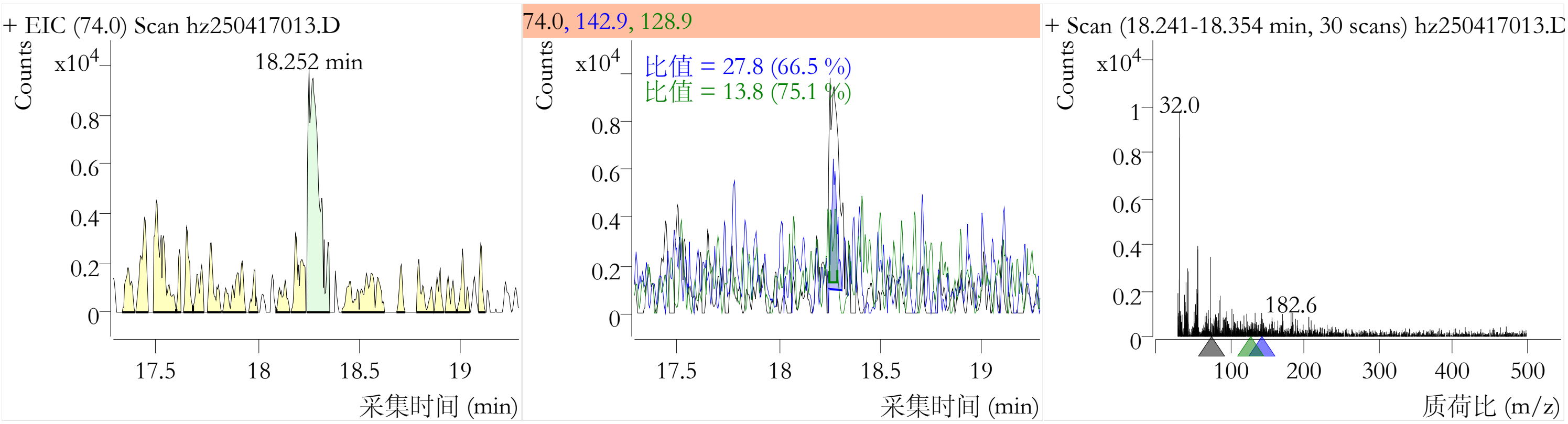

C13:0

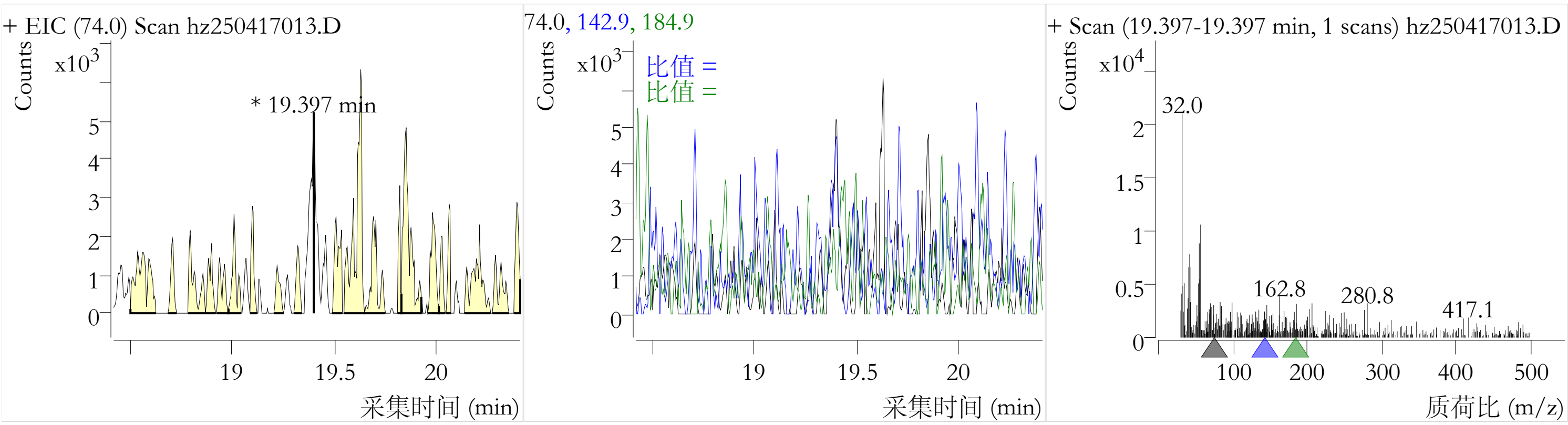

C14:0

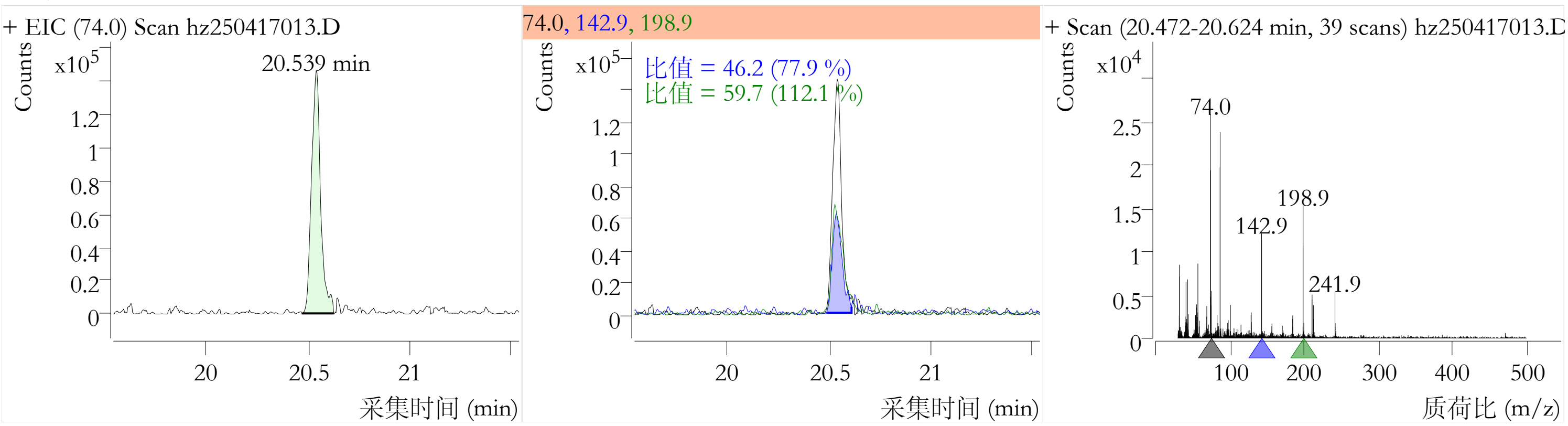

C14:1

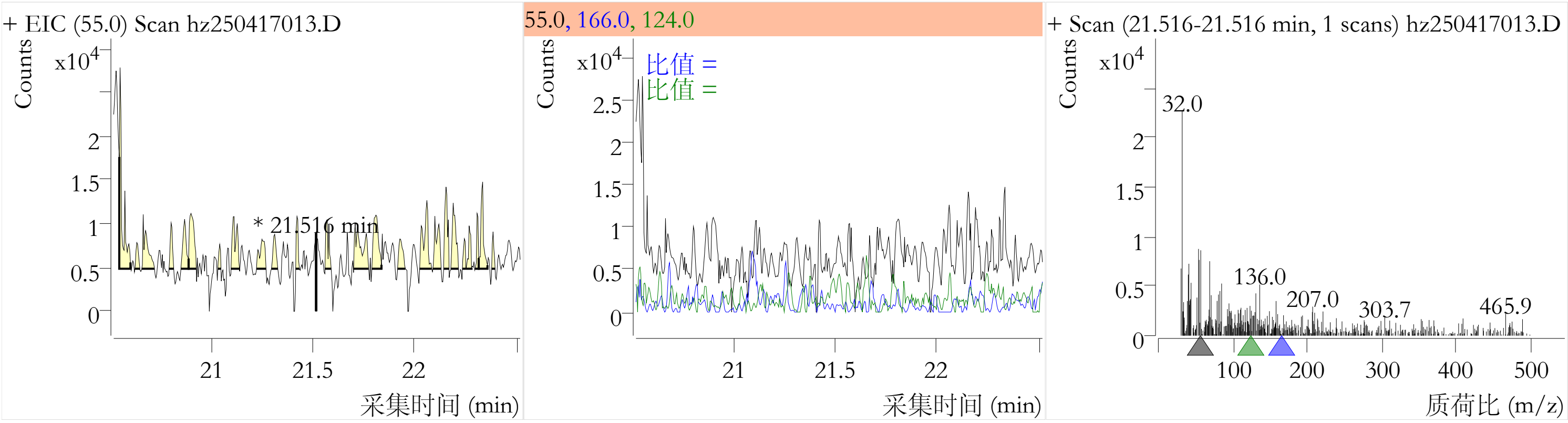

C15:0

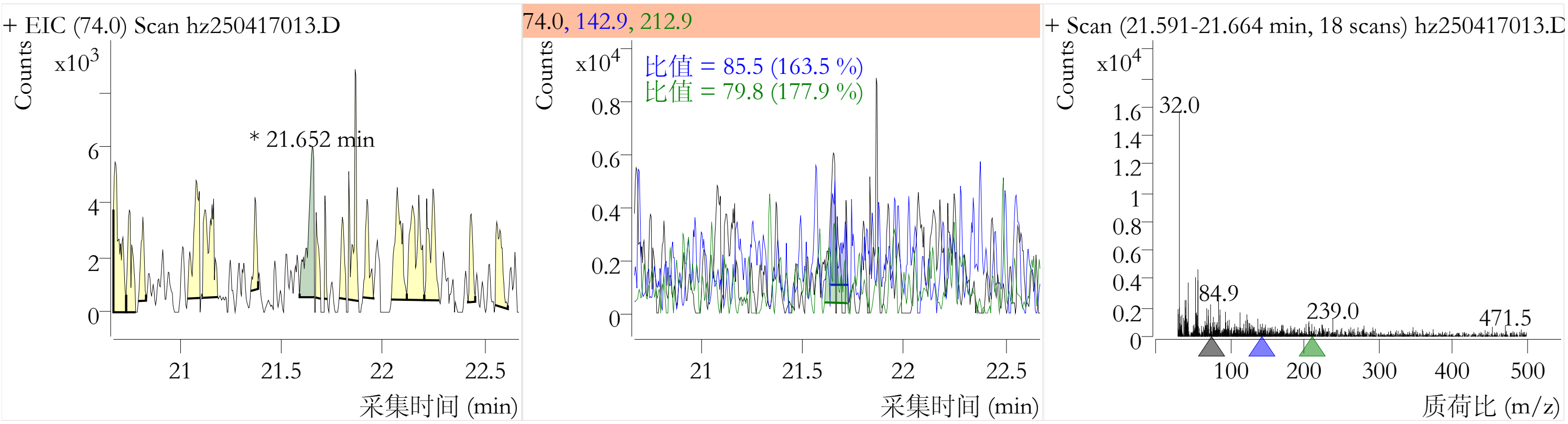

C15:1

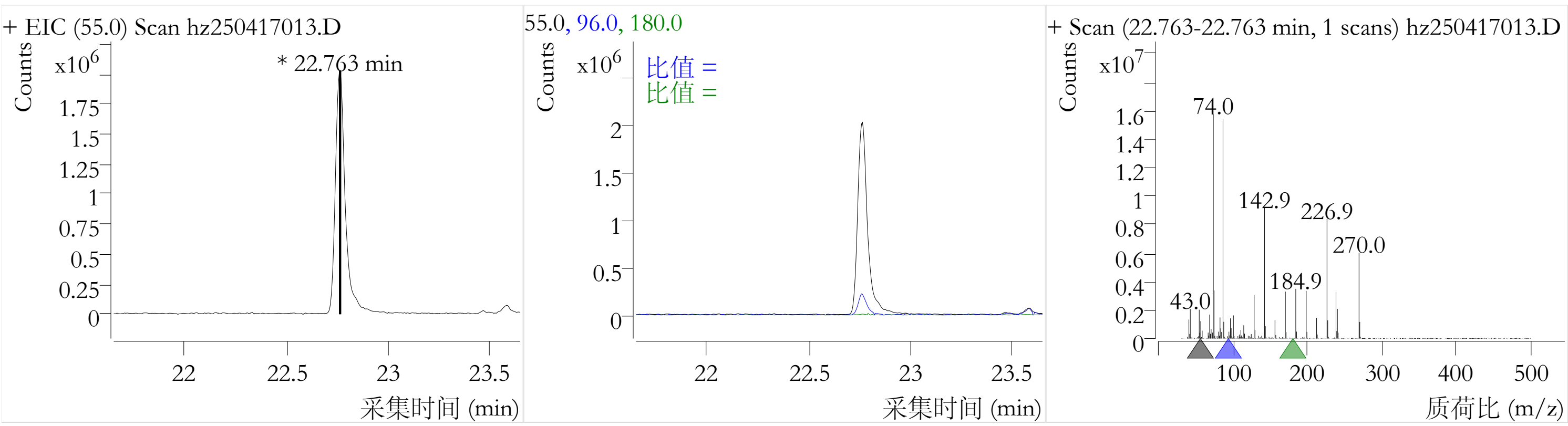

C16:0

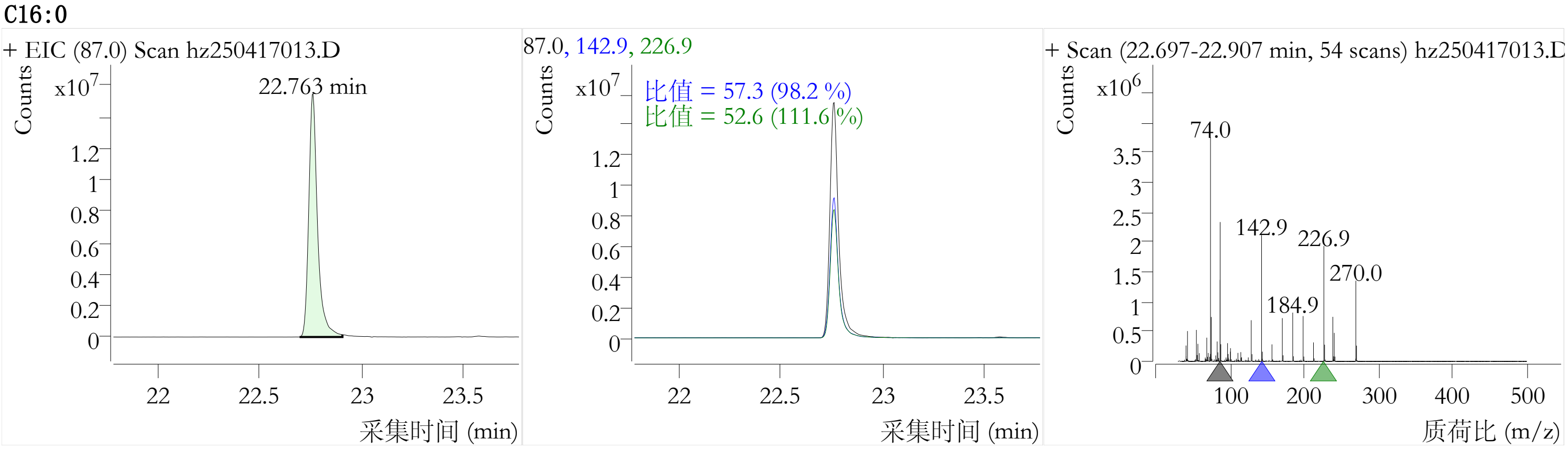

C16:1

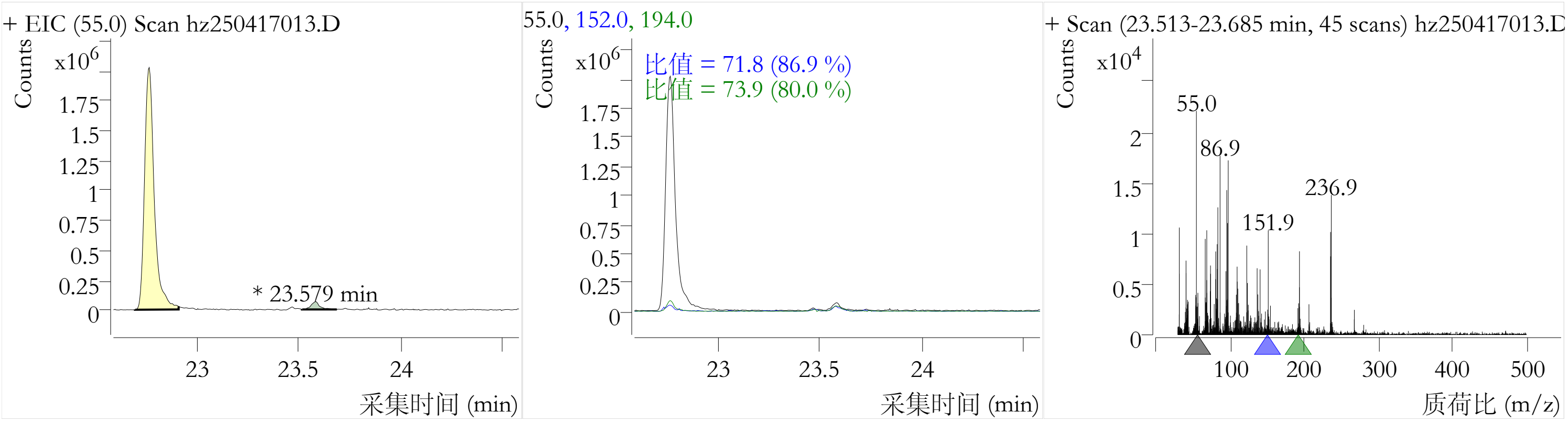

C17:0

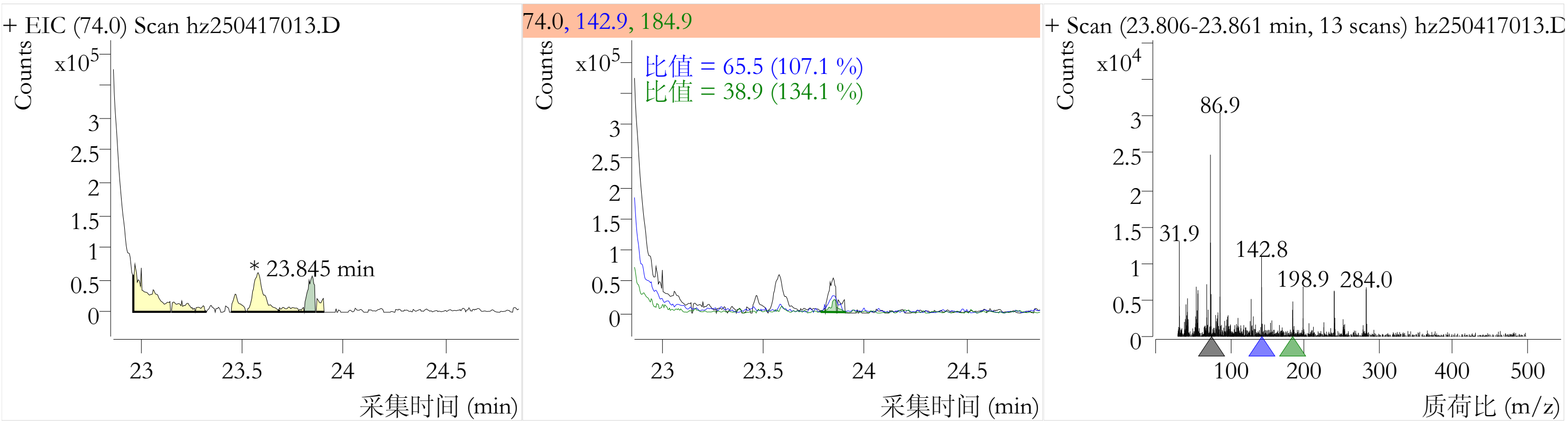

C17:1

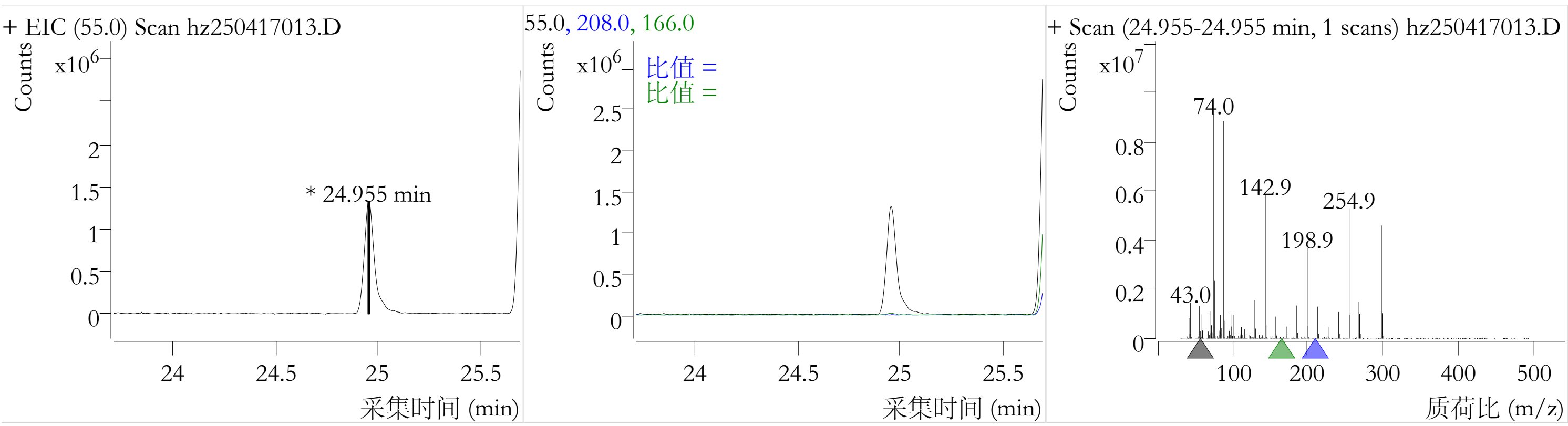

C18:0

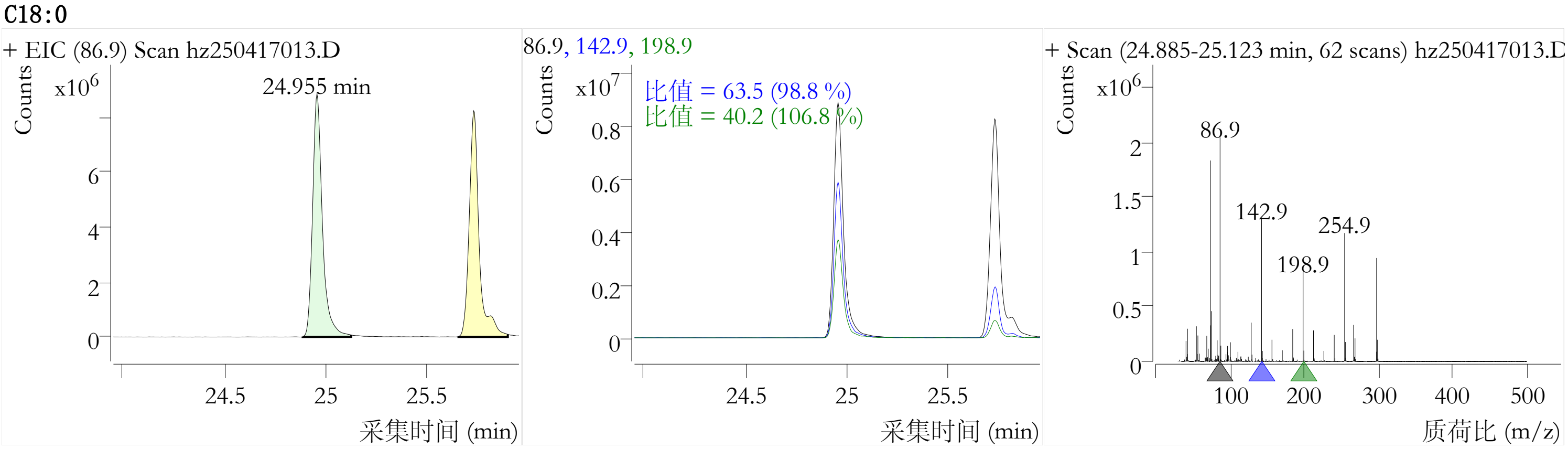

C18:1n9t

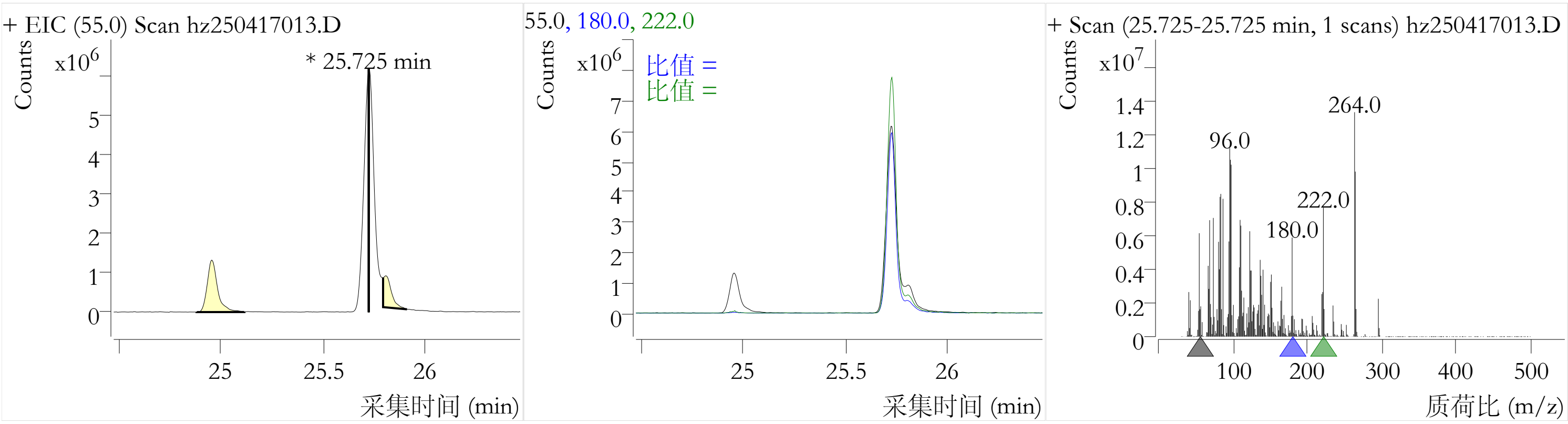

C18:1n9c

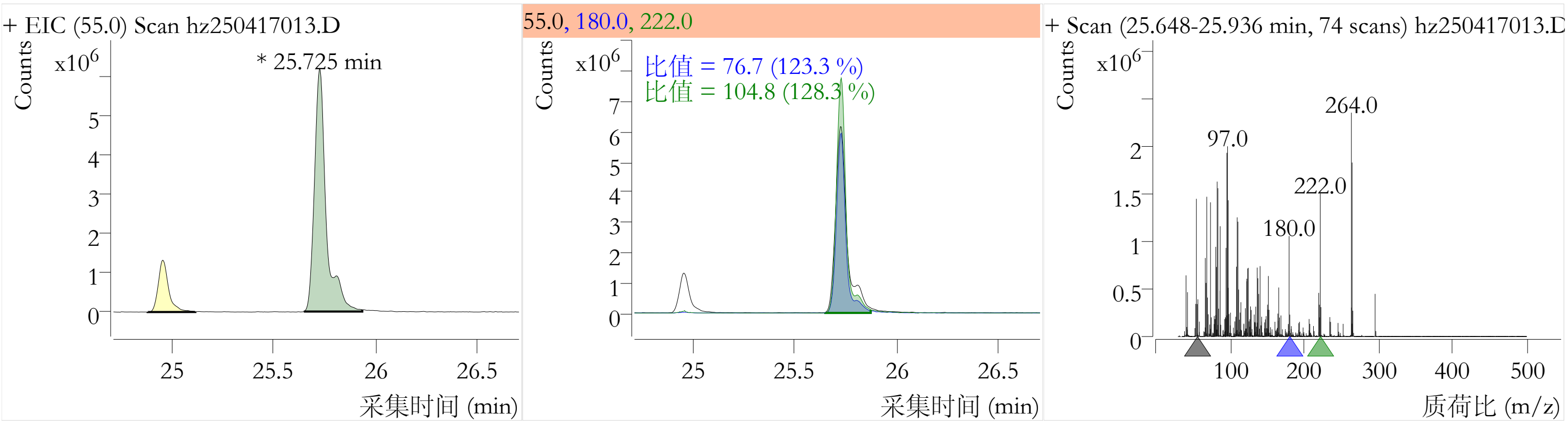

C18:2n6t

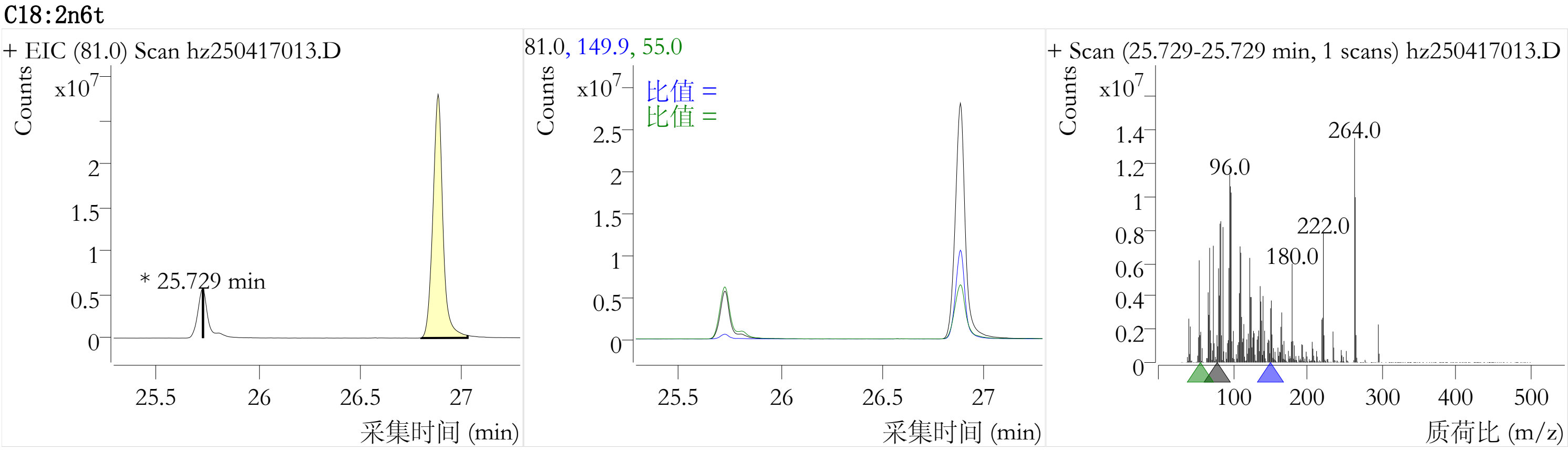

C18:2n6c

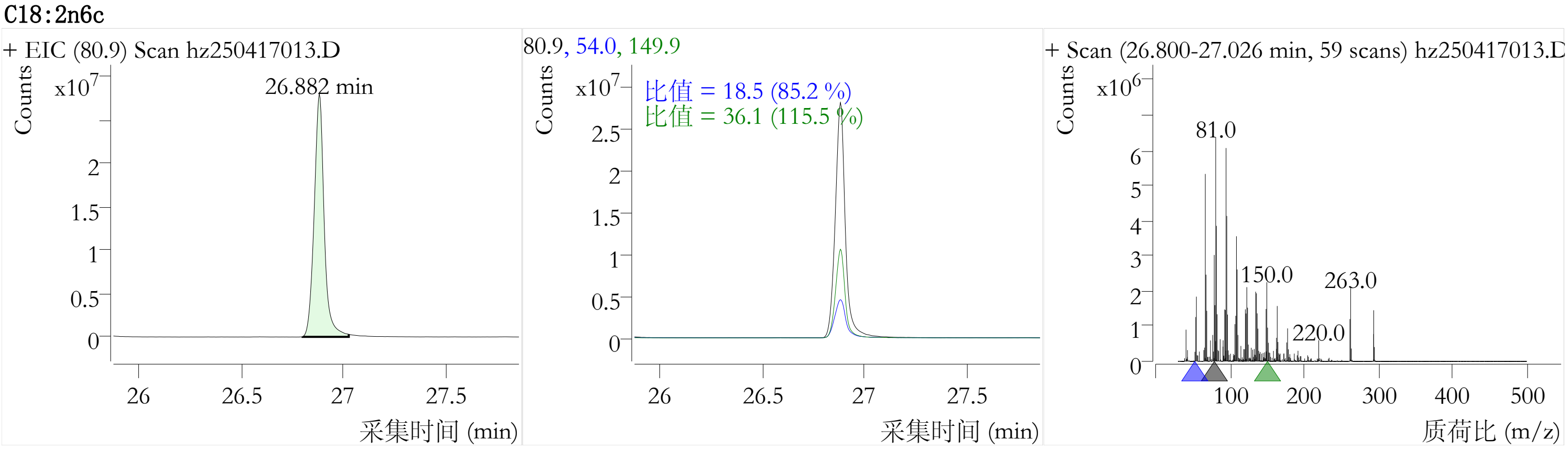

C20:0

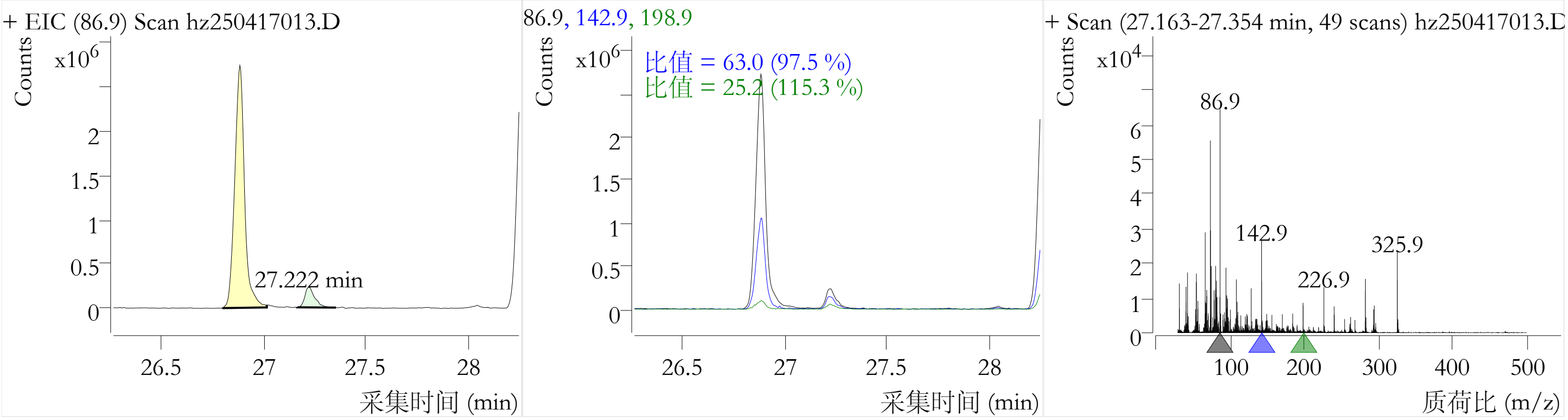

C18:3n6

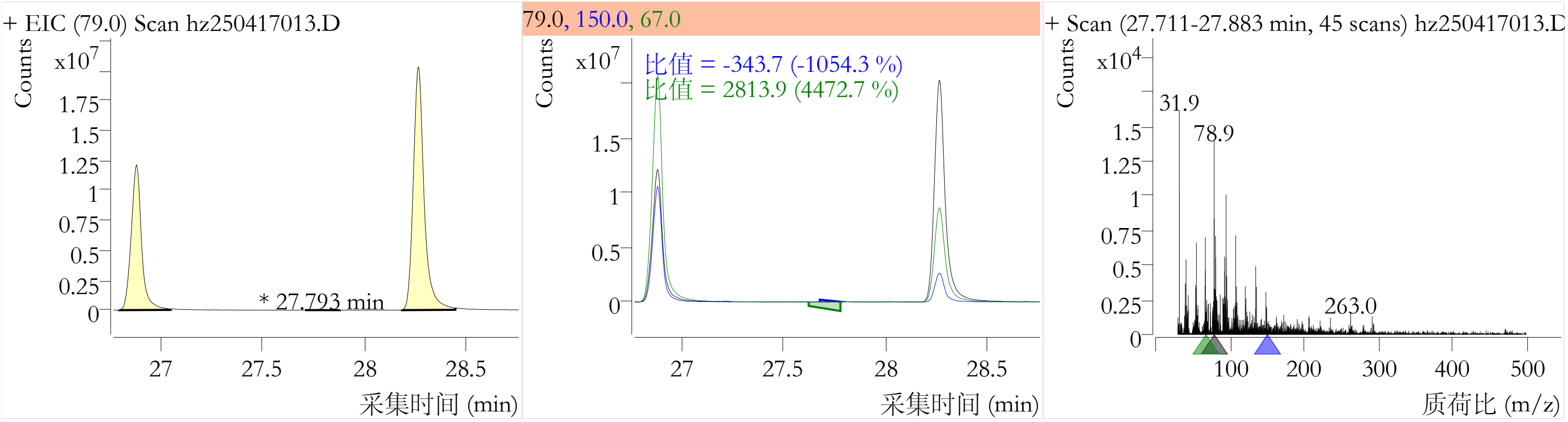

C20:1

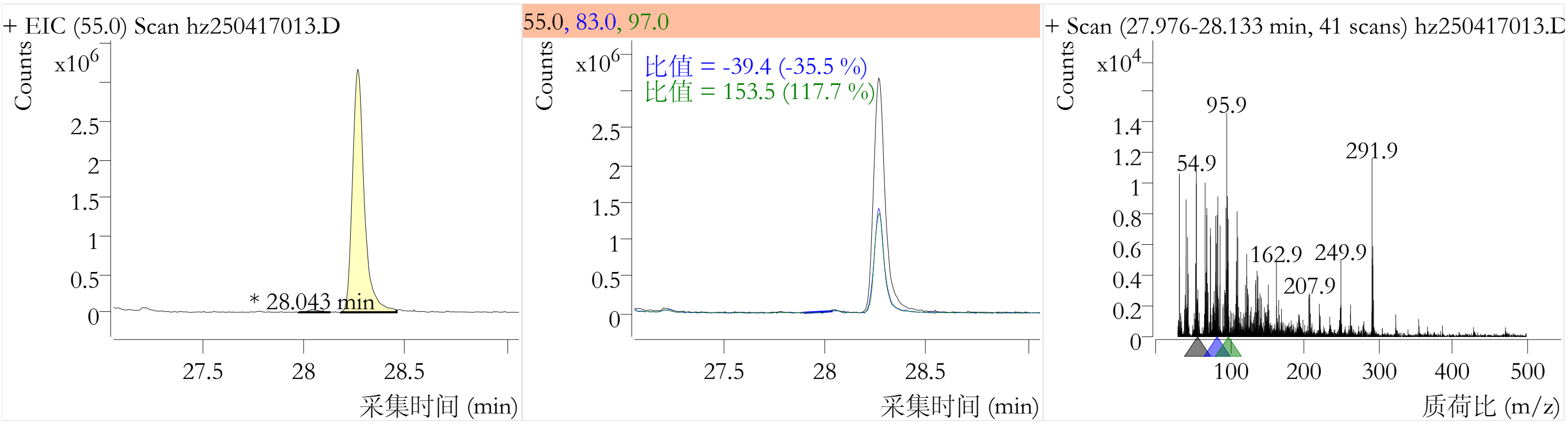

C18:3n3

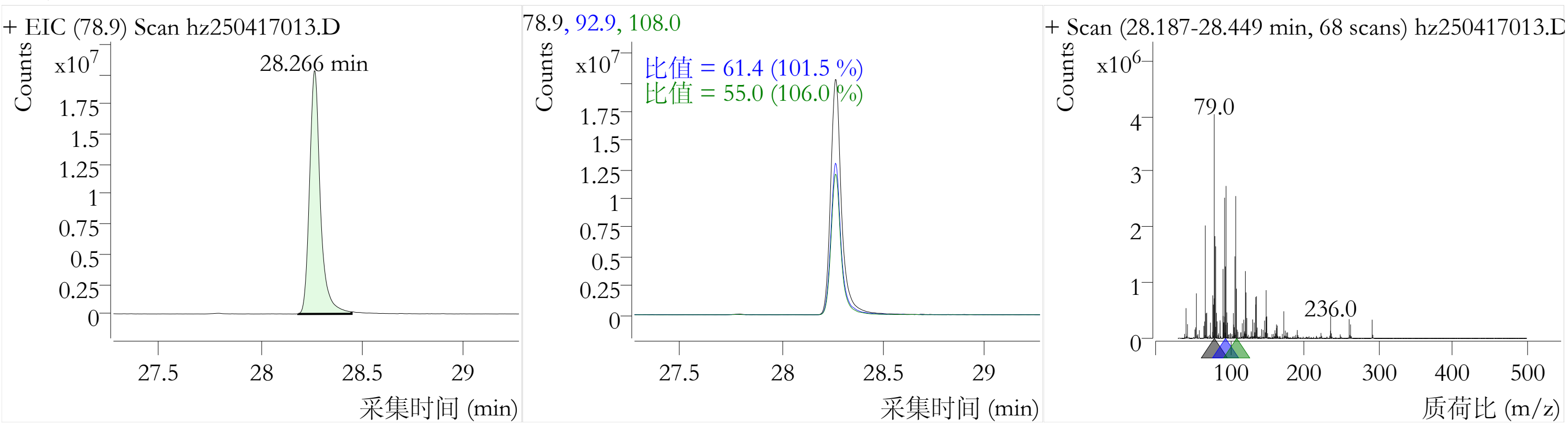

C21:0

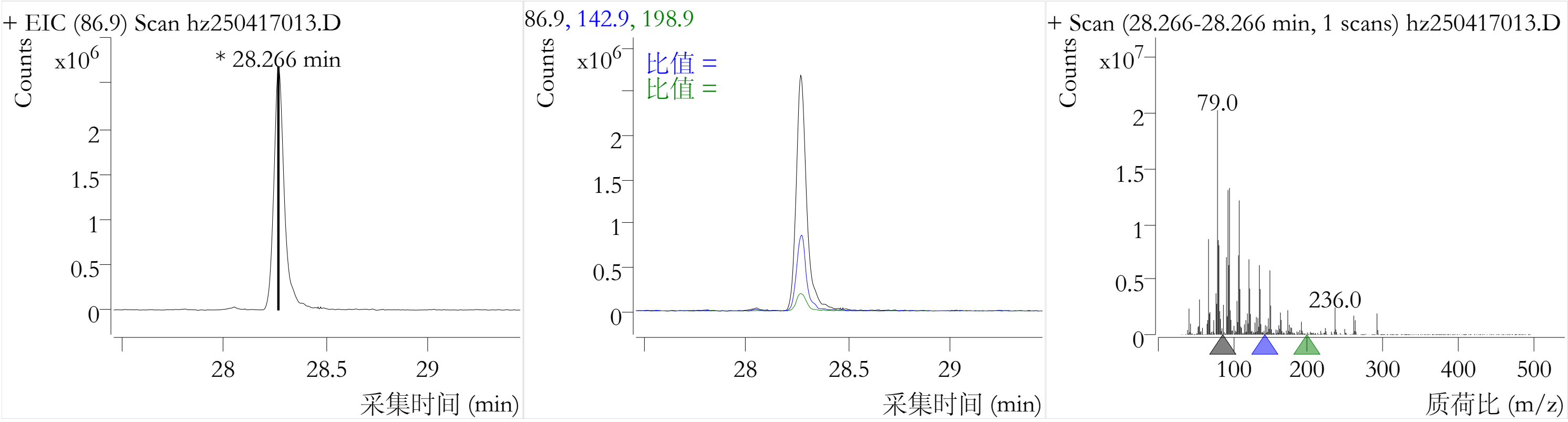

C20:2

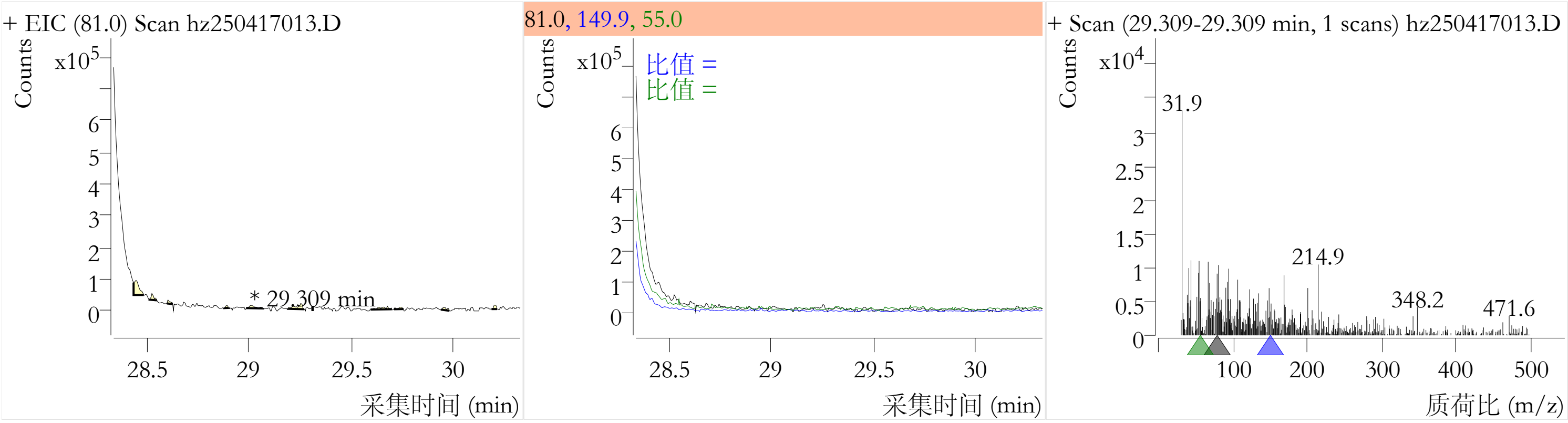

C22:0

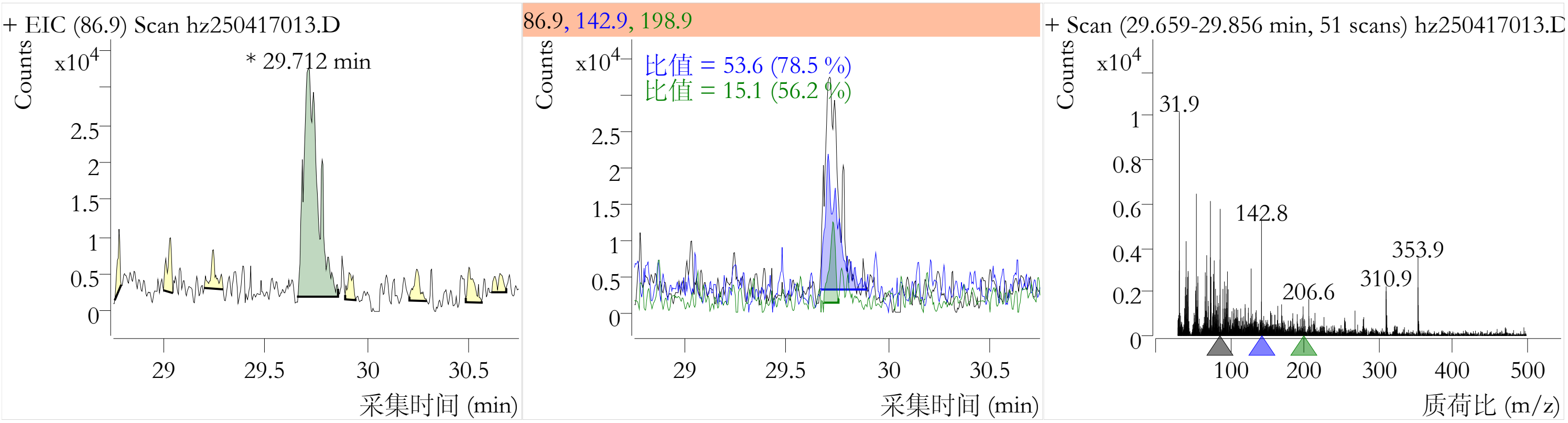

C20:3n6

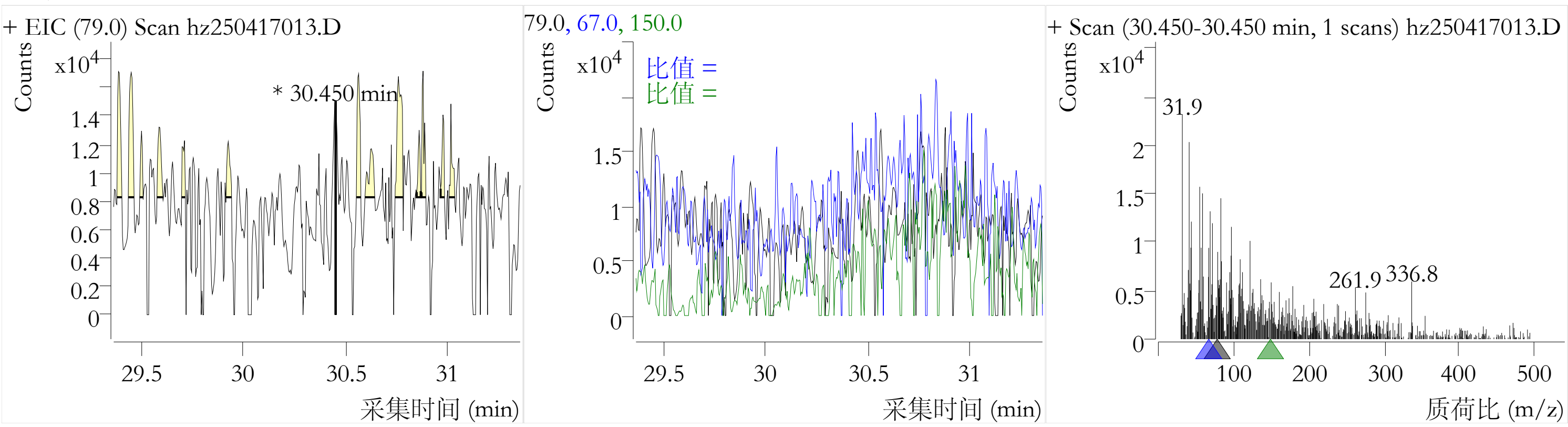

C22:1n9

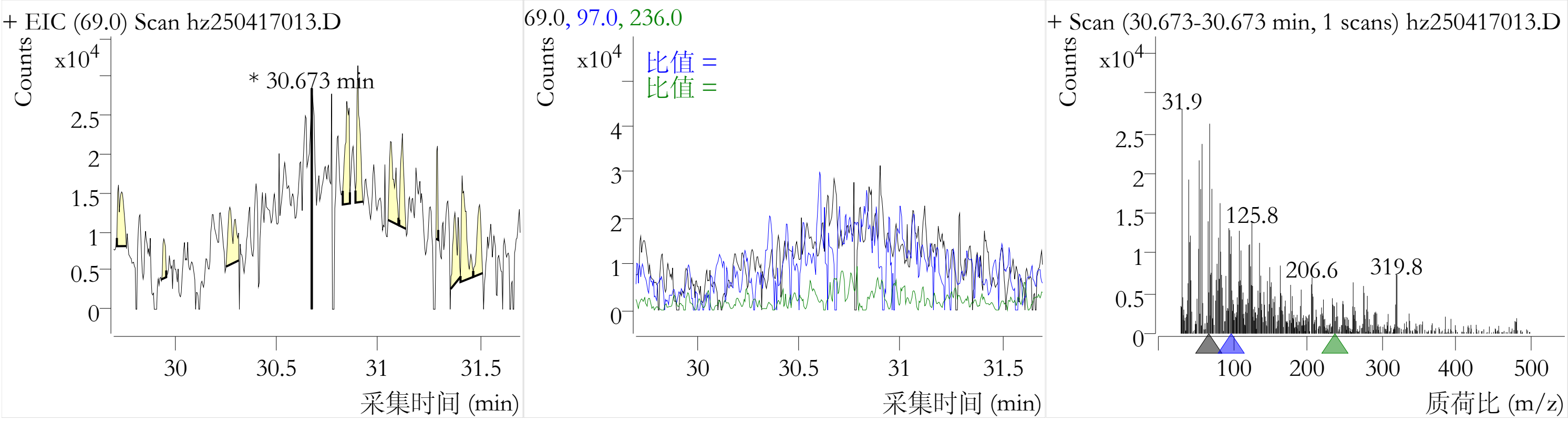

C20:3n3

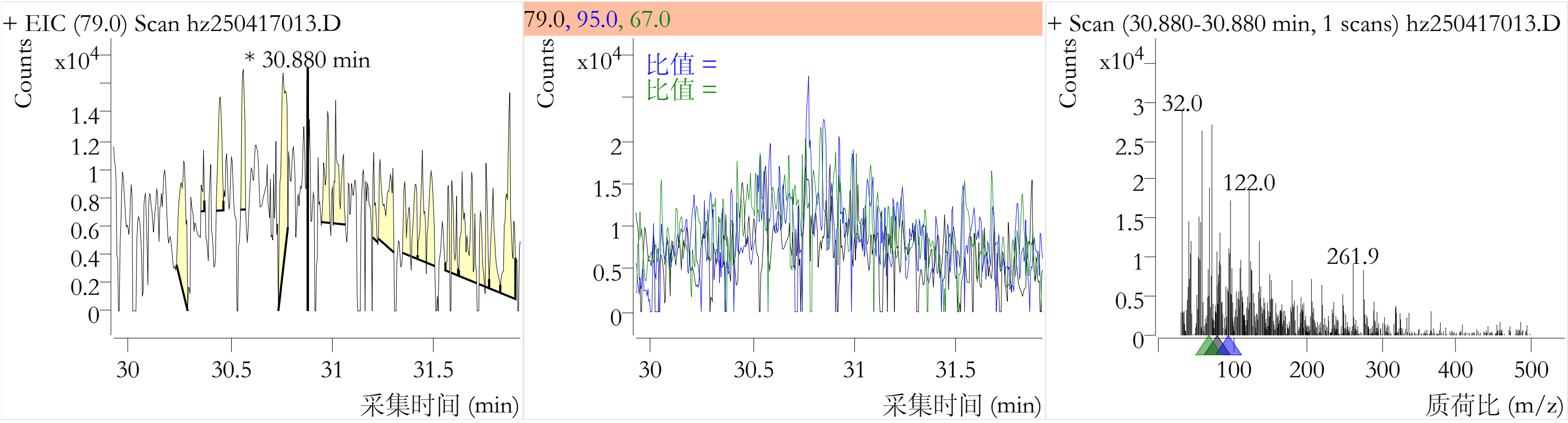

C23:0

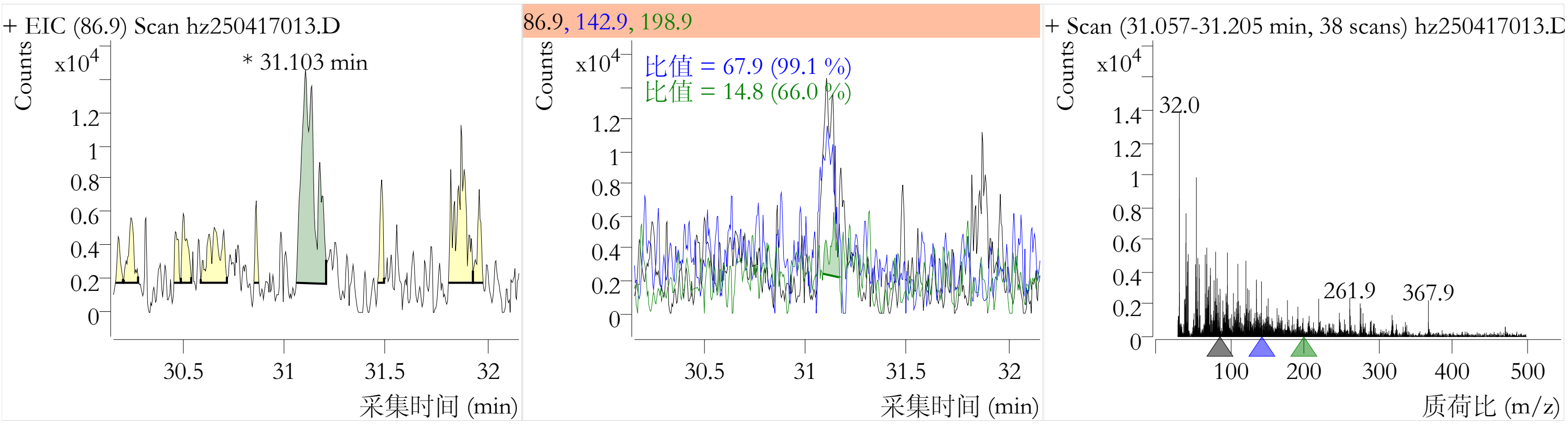

C20:4n6

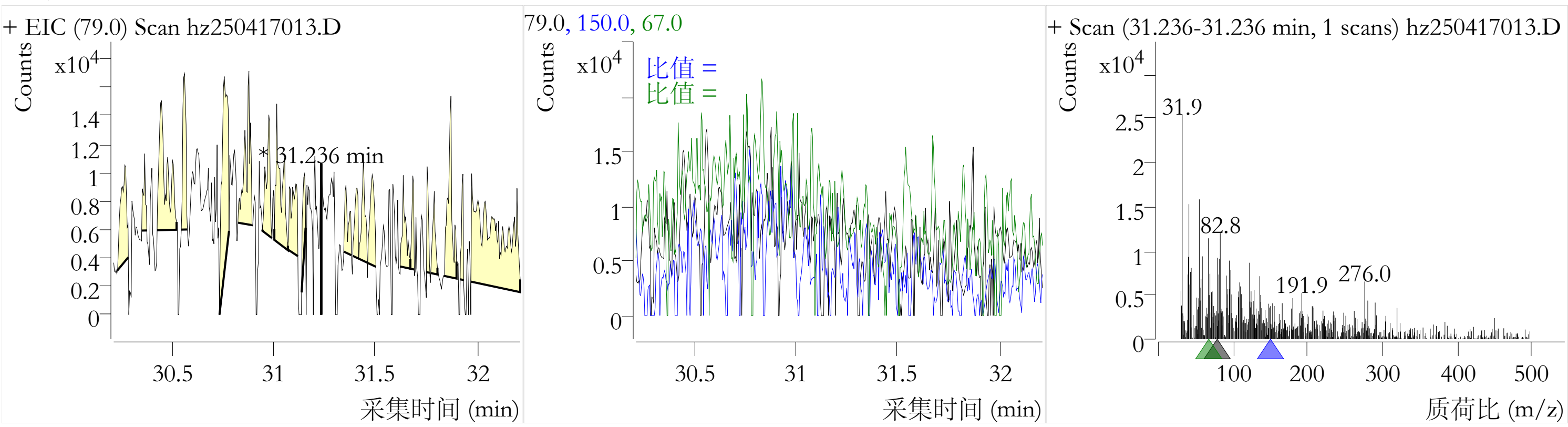

C22:2

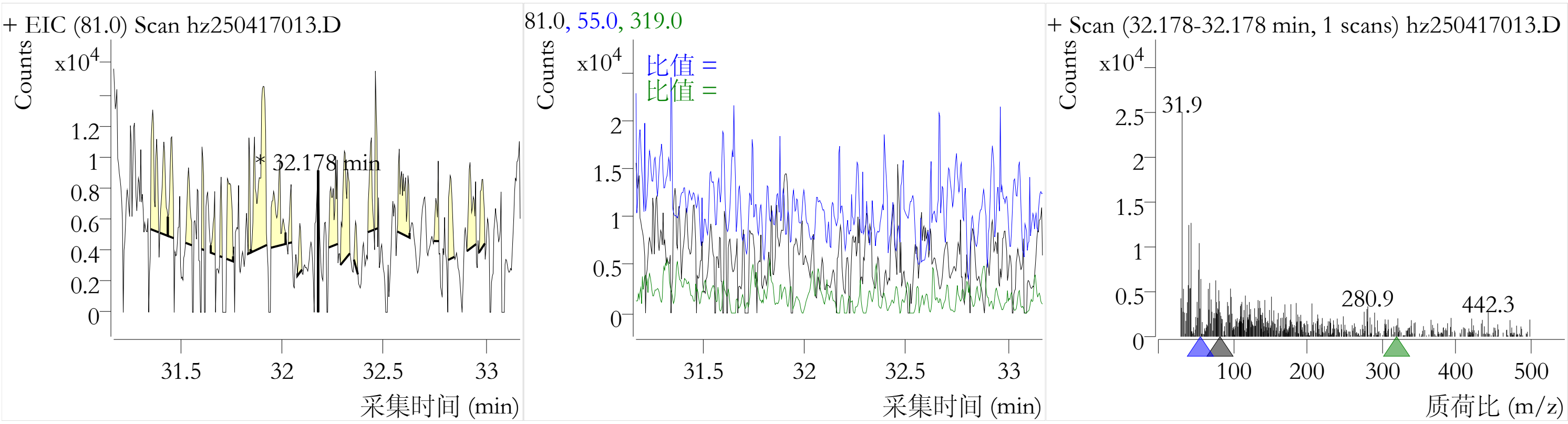

C24:0

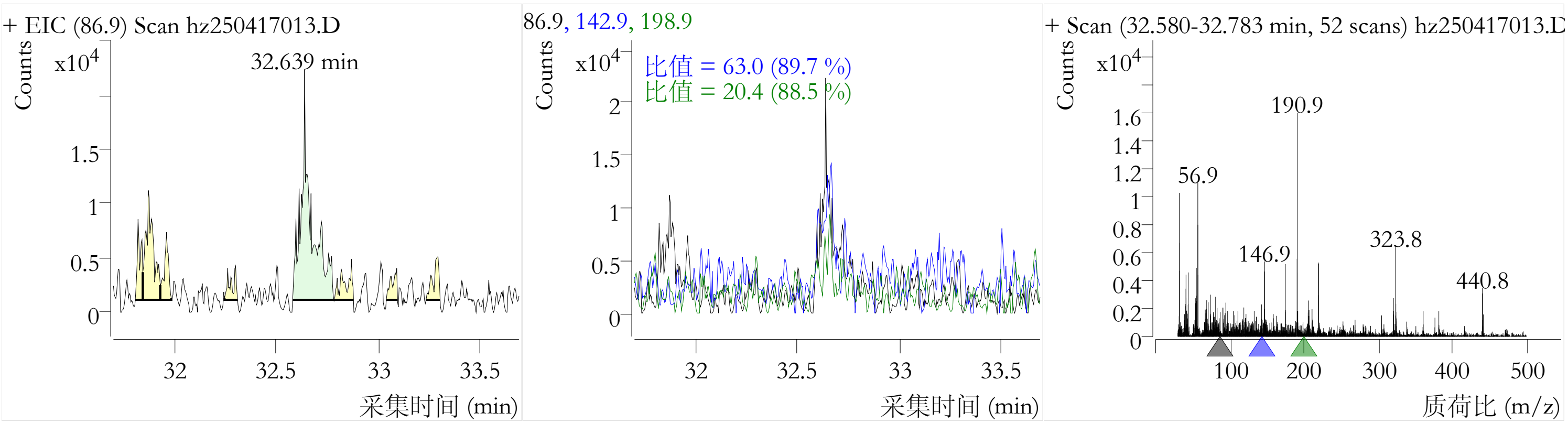

C20:5n3

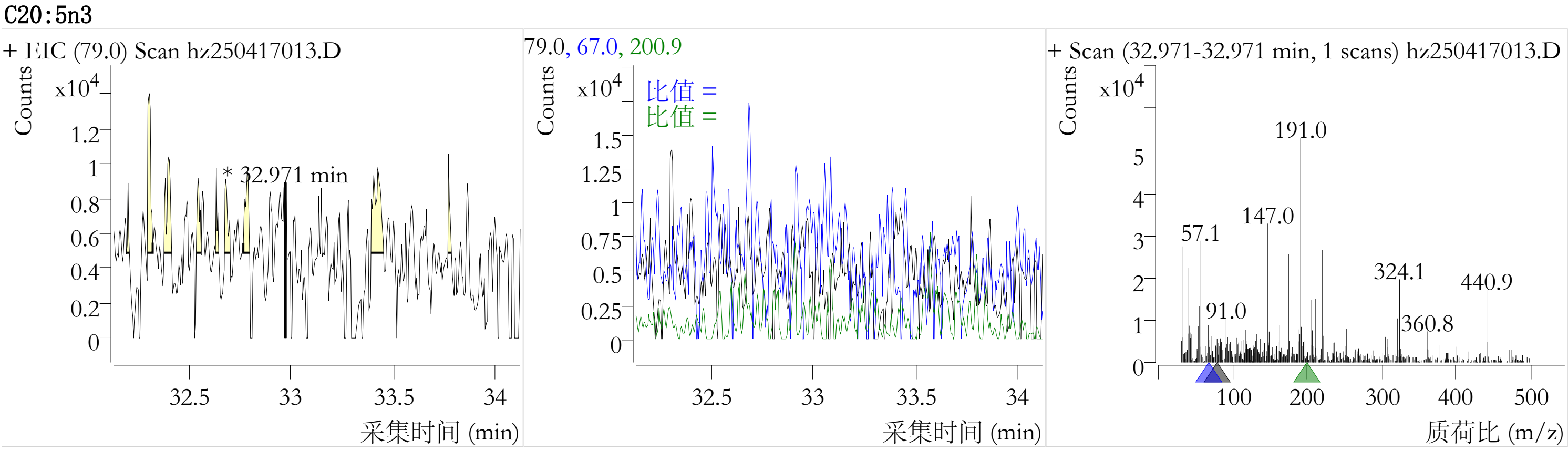

C24:1

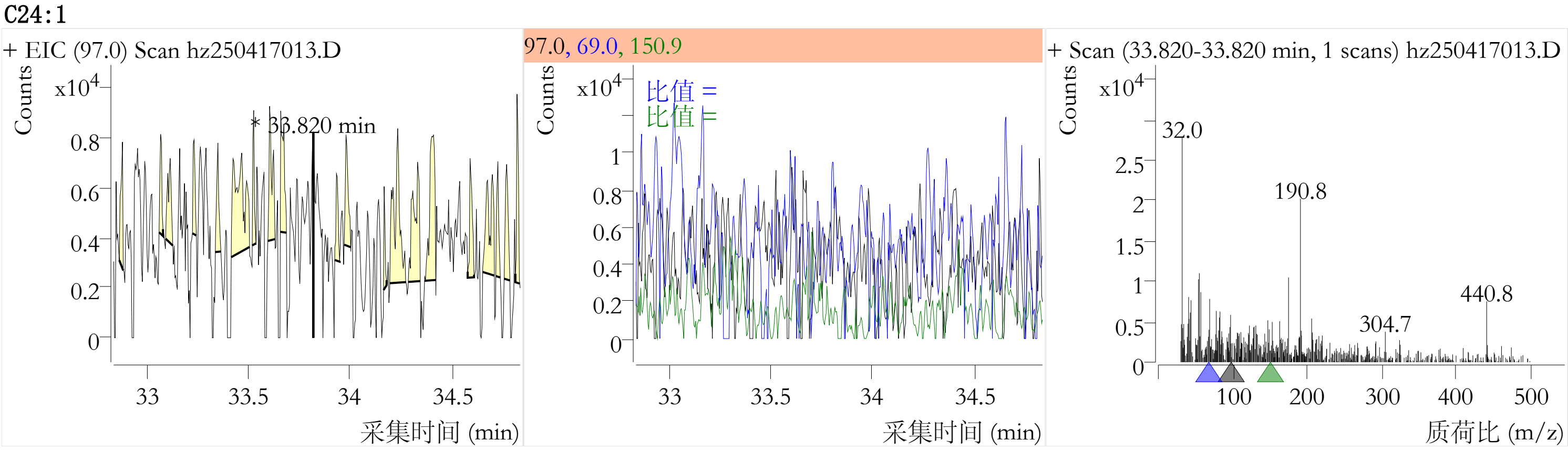

C22:6n3

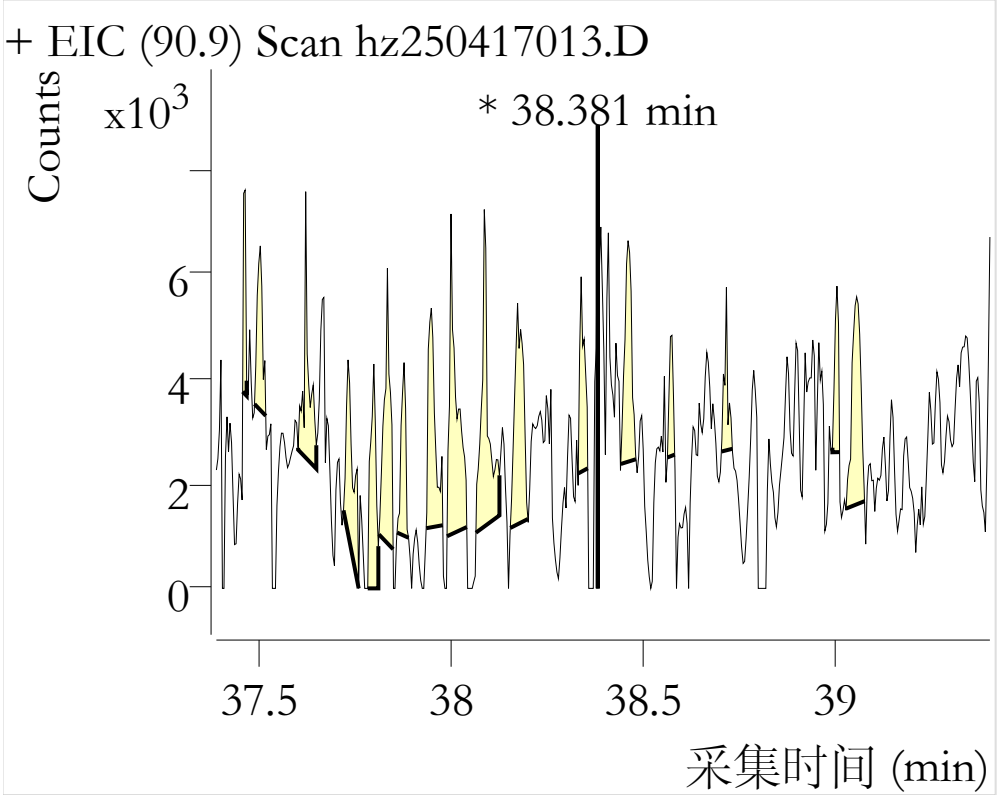

+ EIC (90.9) Scan hz250417013.D
Counts
x10^3
* 38.381 min
6
4
2
0
37.5
38
38.5
39
采集时间 (min)

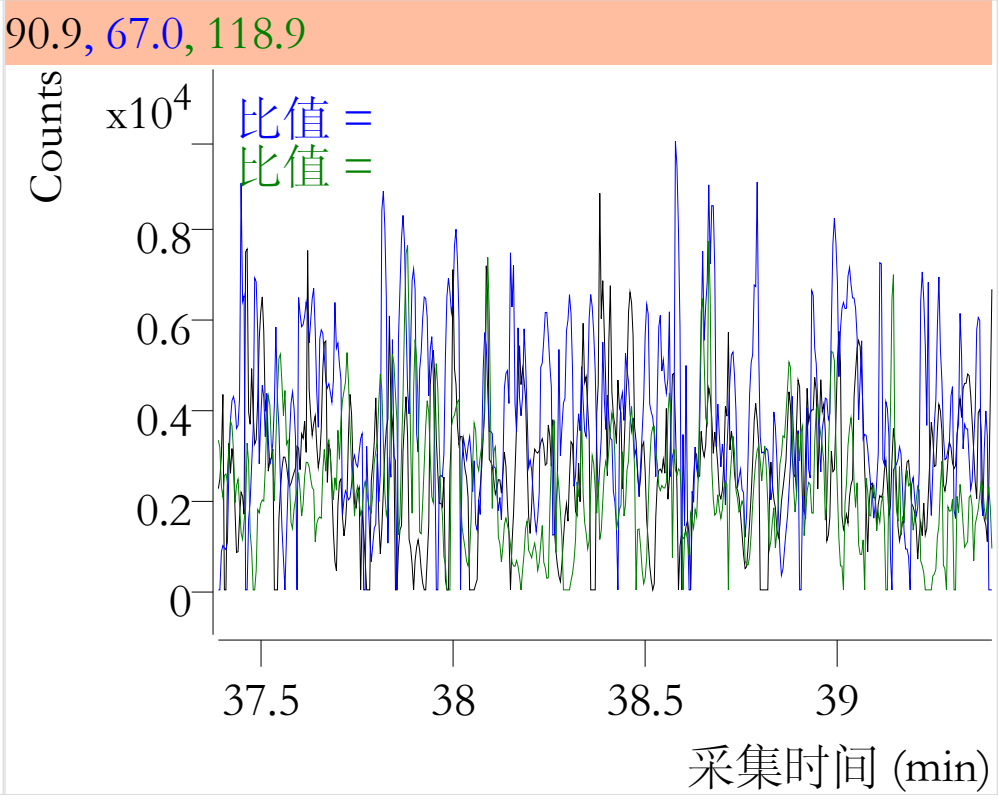

90.9, 67.0, 118.9
Counts
x10^4
比值 =
比值 =
0.8
0.6
0.4
0.2
0
37.5
38
38.5
39
采集时间 (min)

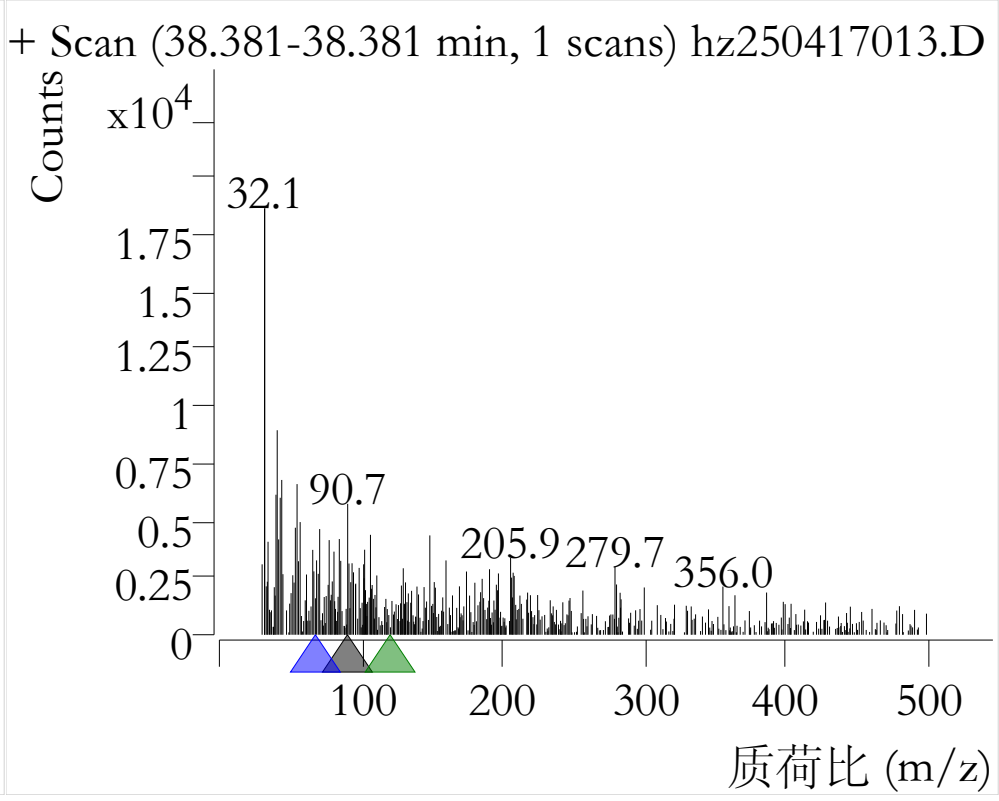

+ Scan (38.381-38.381 min, 1 scans) hz250417013.D
Counts
x10^4
32.1
1.75
1.5
1.25
1
0.75
0.5
0.25
0
90.7
205.9
279.7
356.0
100
200
300
400
500
质荷比 (m/z)
