# Supplementary material for: Monoglyceride-structured rubber seed oil oleogels as a partial cocoa butter replacement in chocolate: physical properties, oxidative stability and applications
Source: Food Chem X. 2026 Oct 11;39:104594. doi: 10.1016/j.fochx.2026.104594 (PMC13683454; doi:10.1016/j.fochx.2026.104594)
Supplement: Supplementary file 8 [file mmc8.pdf]

Original Graph

0.50mm 1% MG-RSO

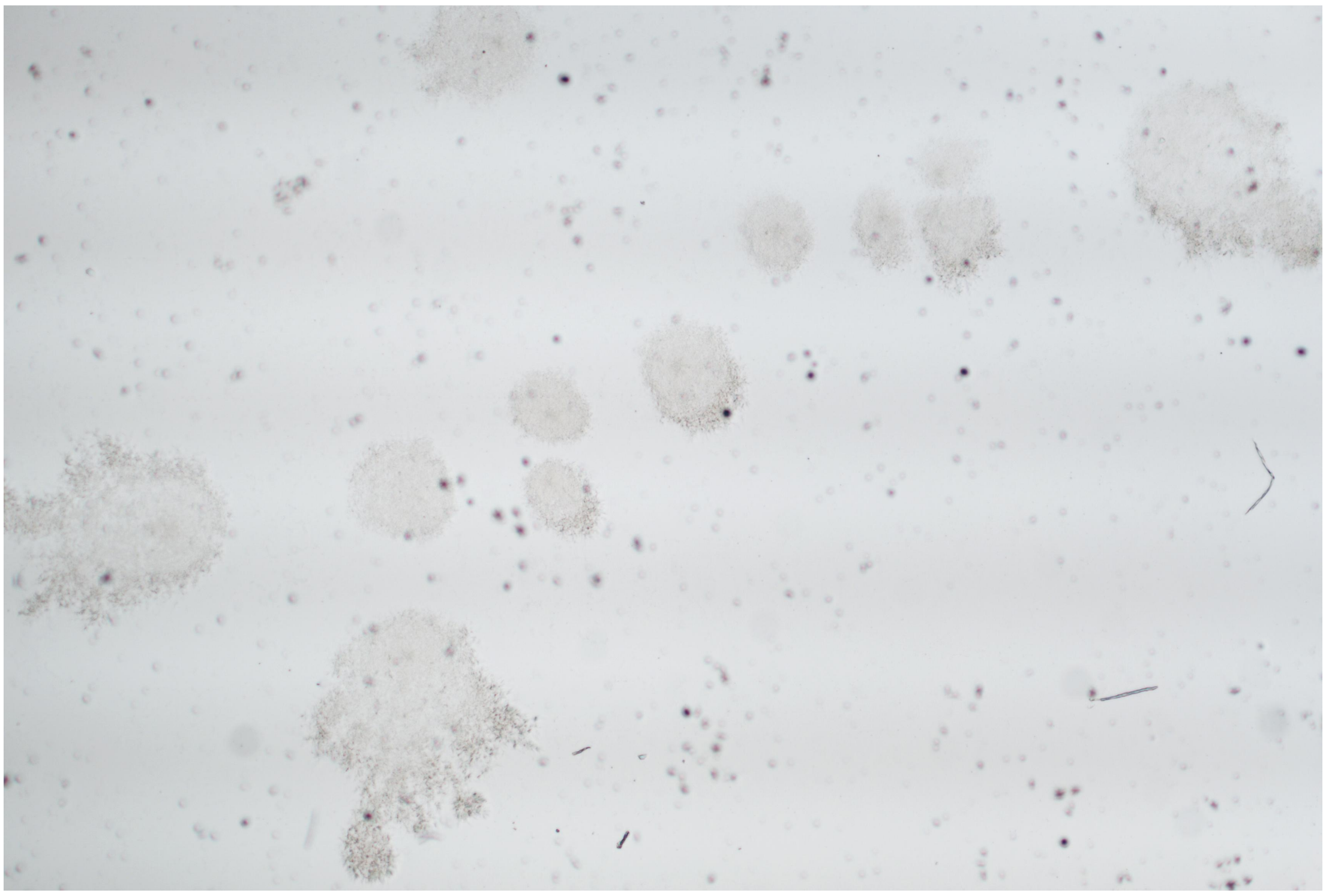

0.50mm 3% MG-RSO

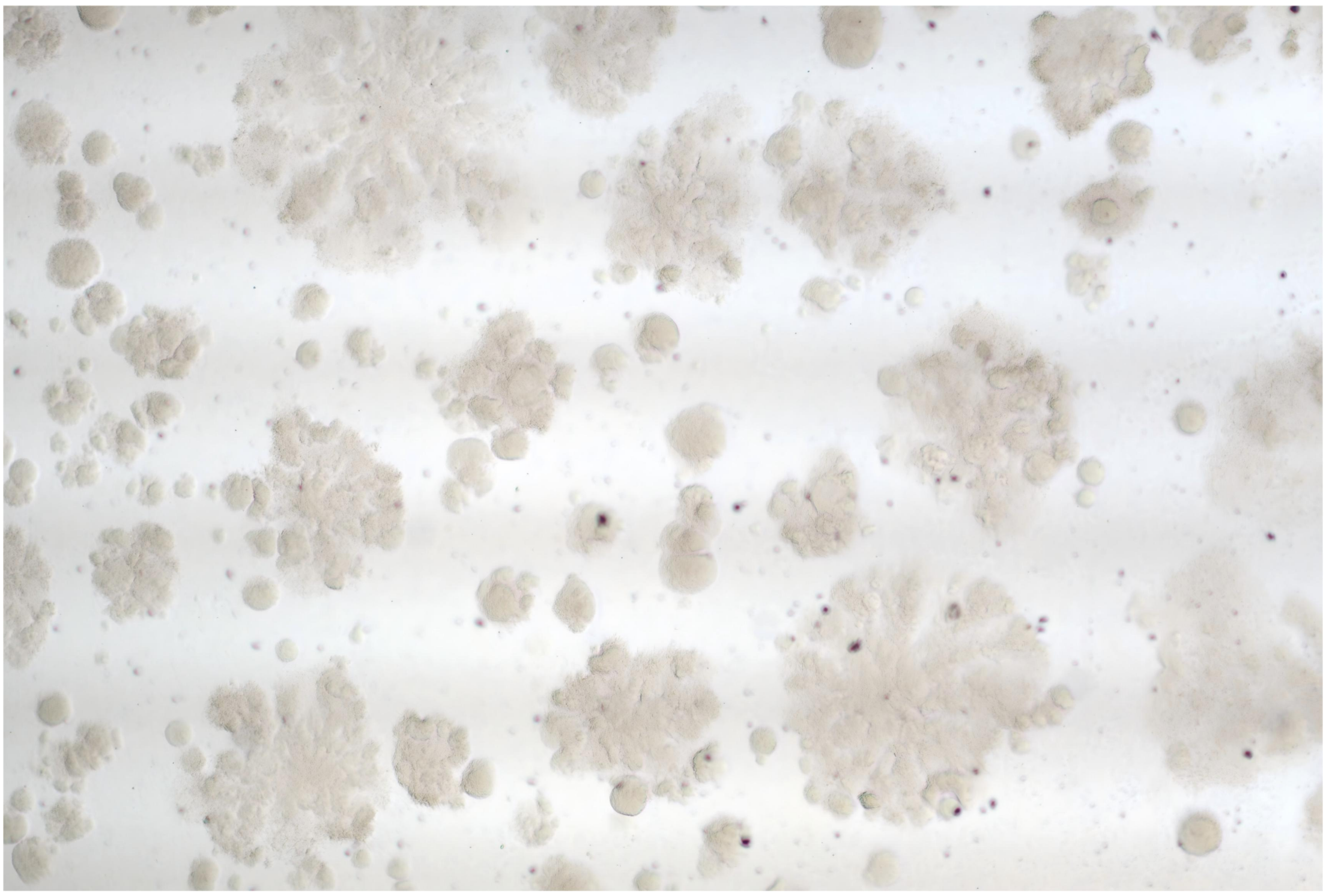

0.50mm 5% MG-RSO

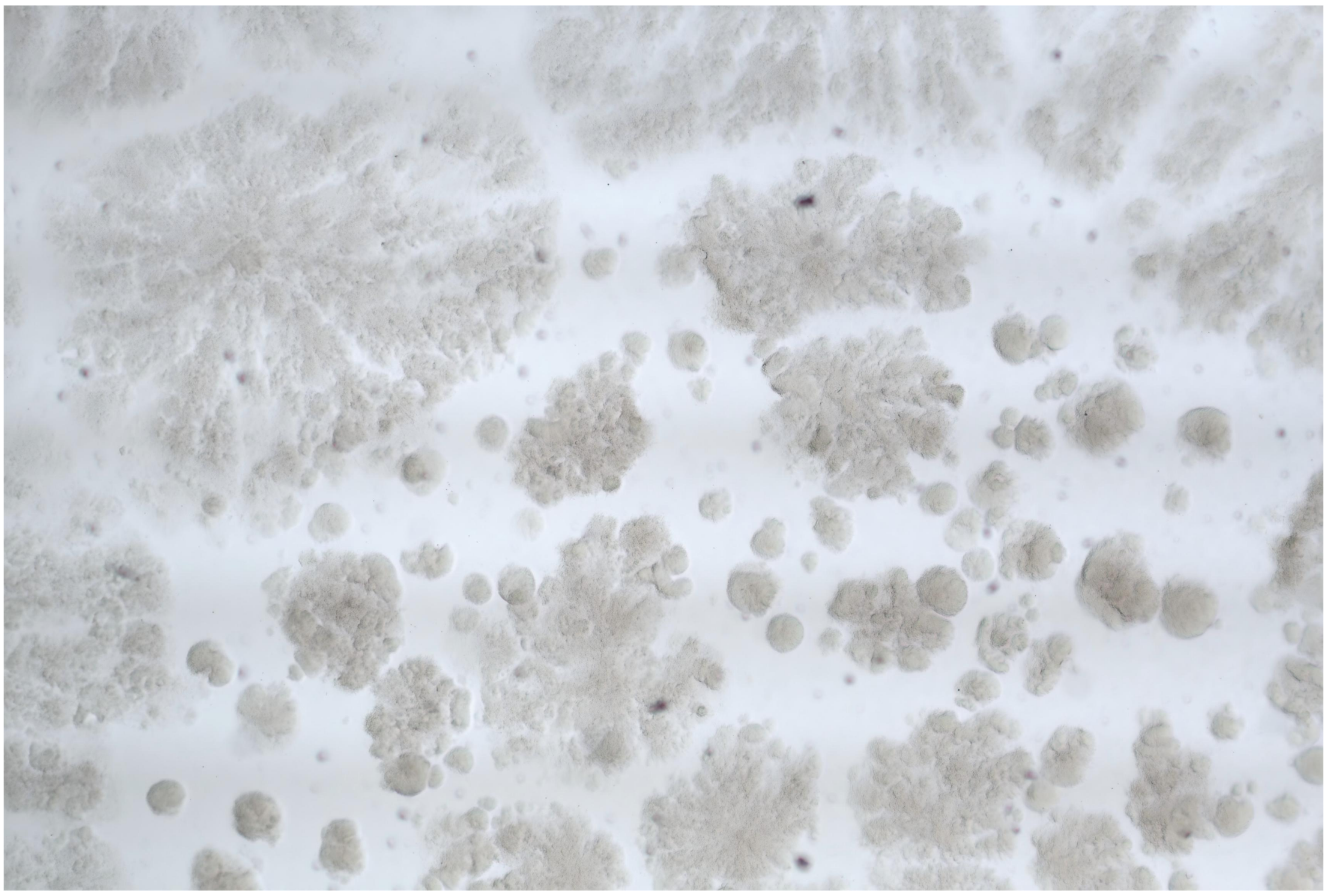

0.50mm 7% MG-RSO

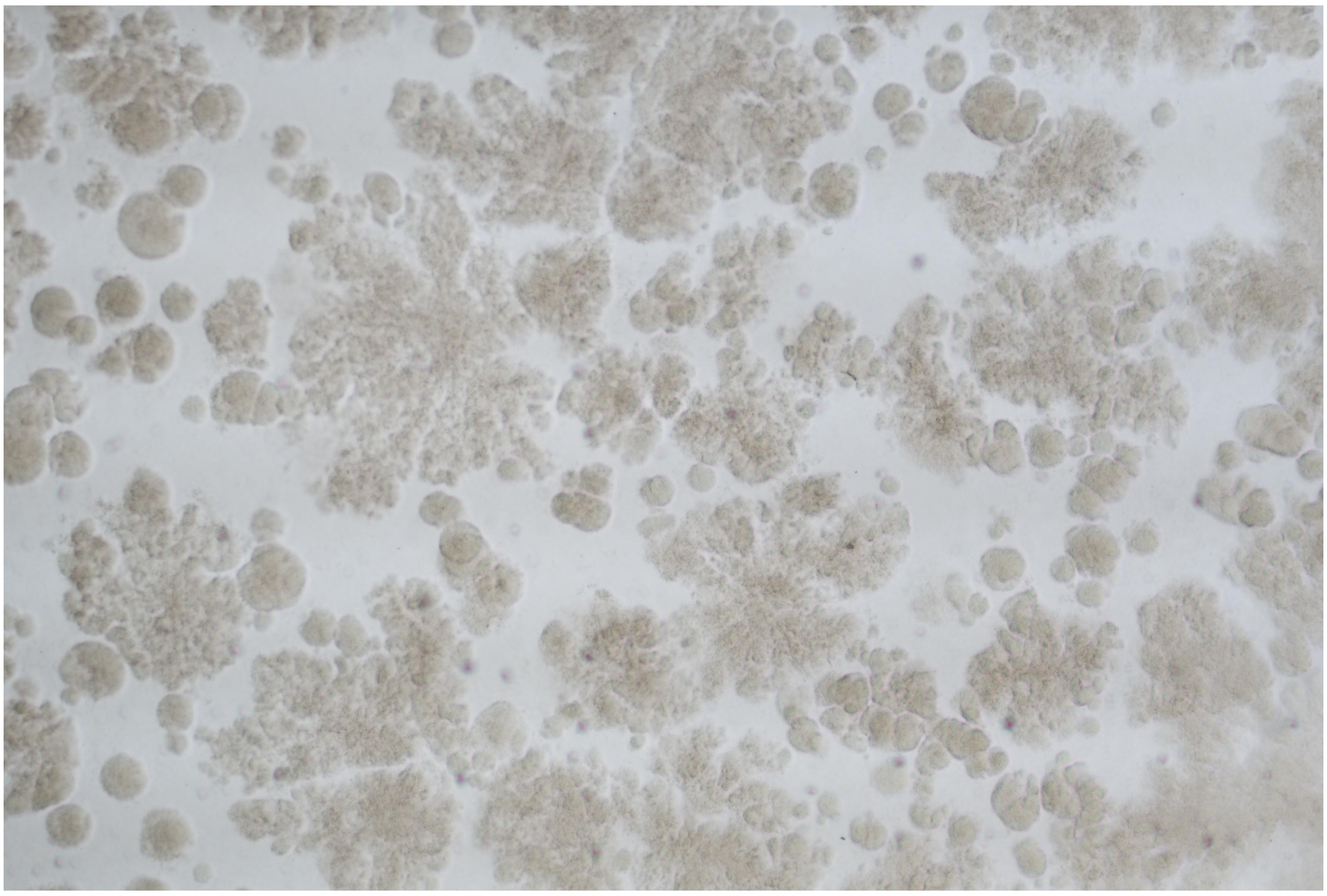

0.50mm 9% MG-RSO

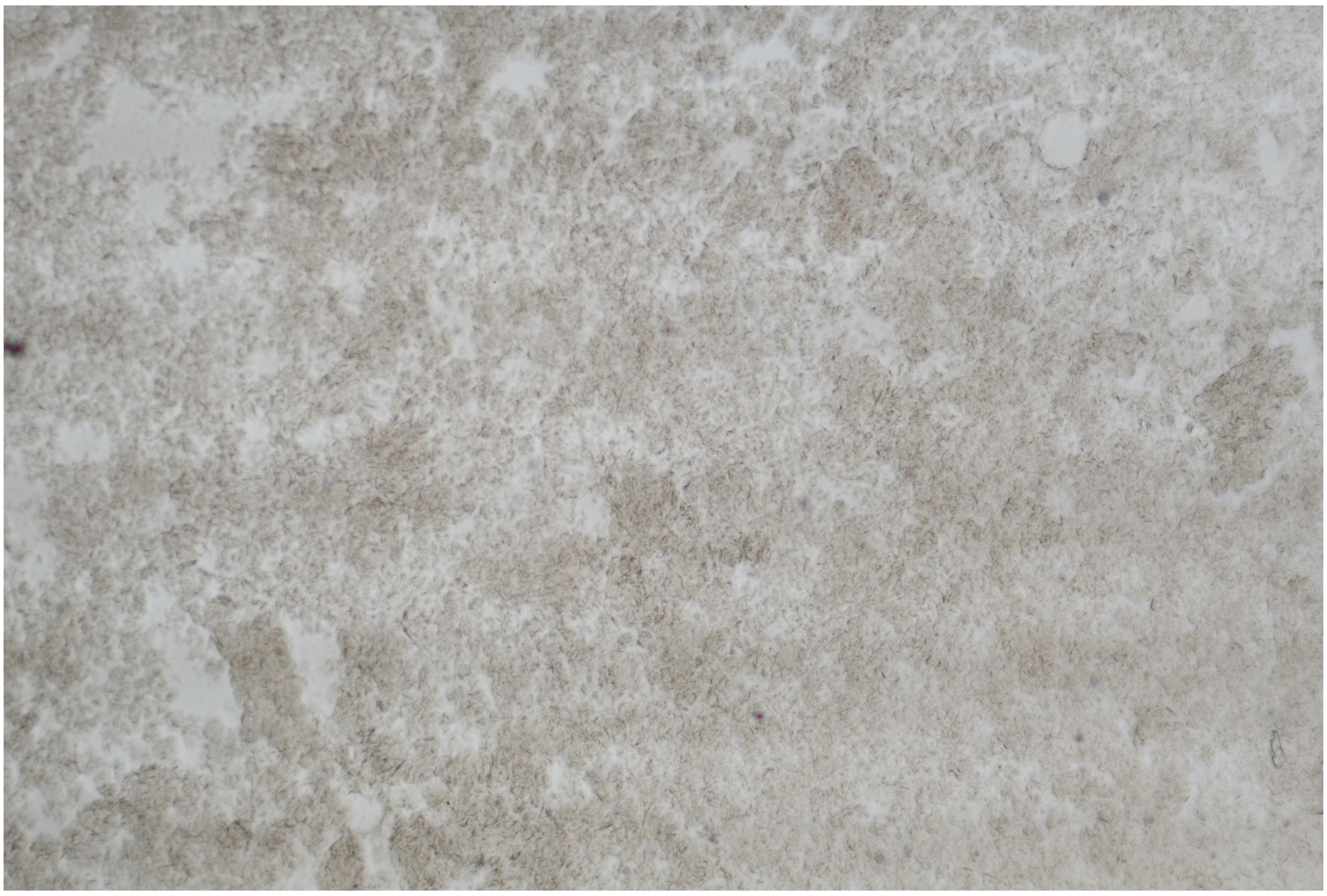

0.50mm 12% MG-RSO

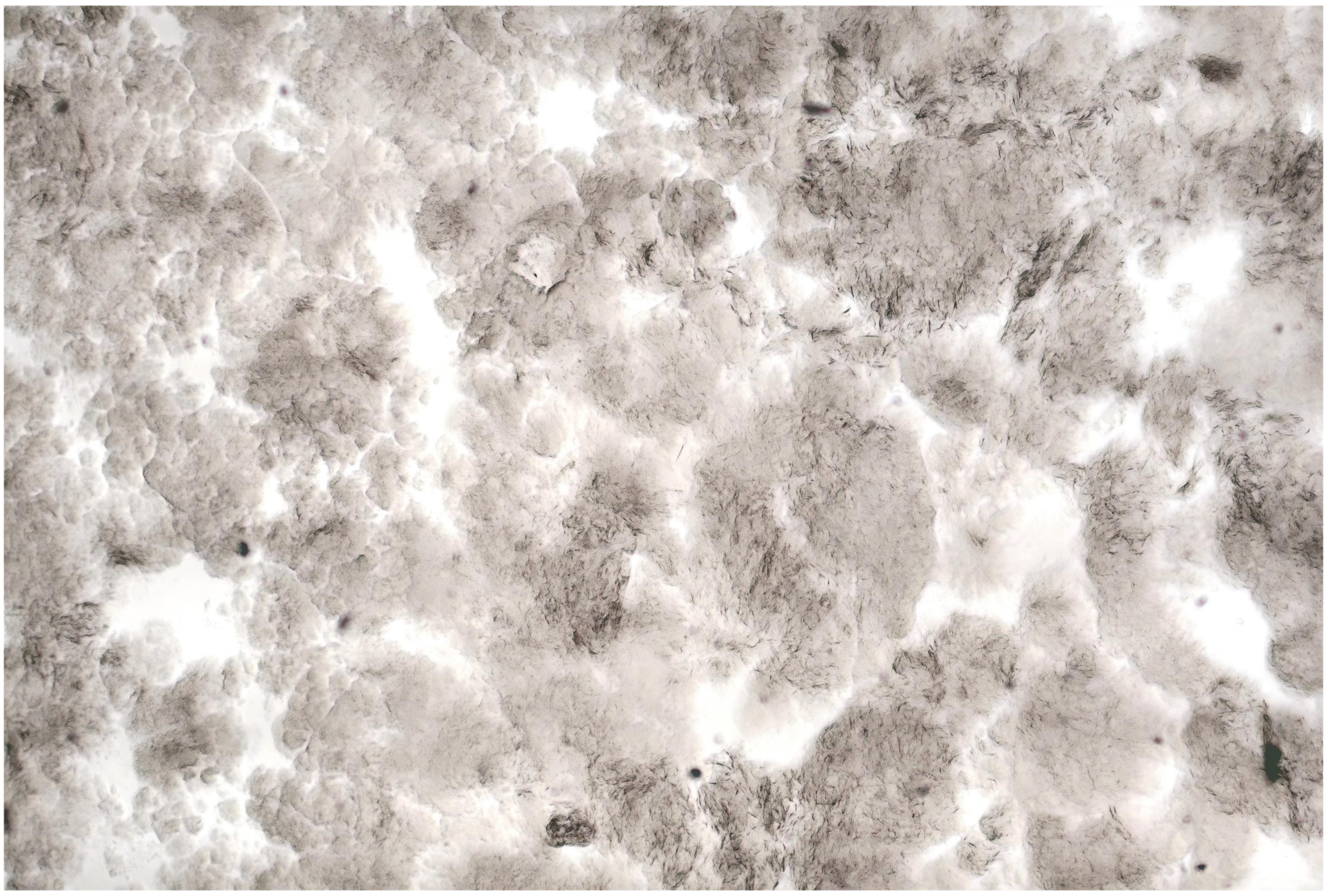

50μm 1% MG-RSO

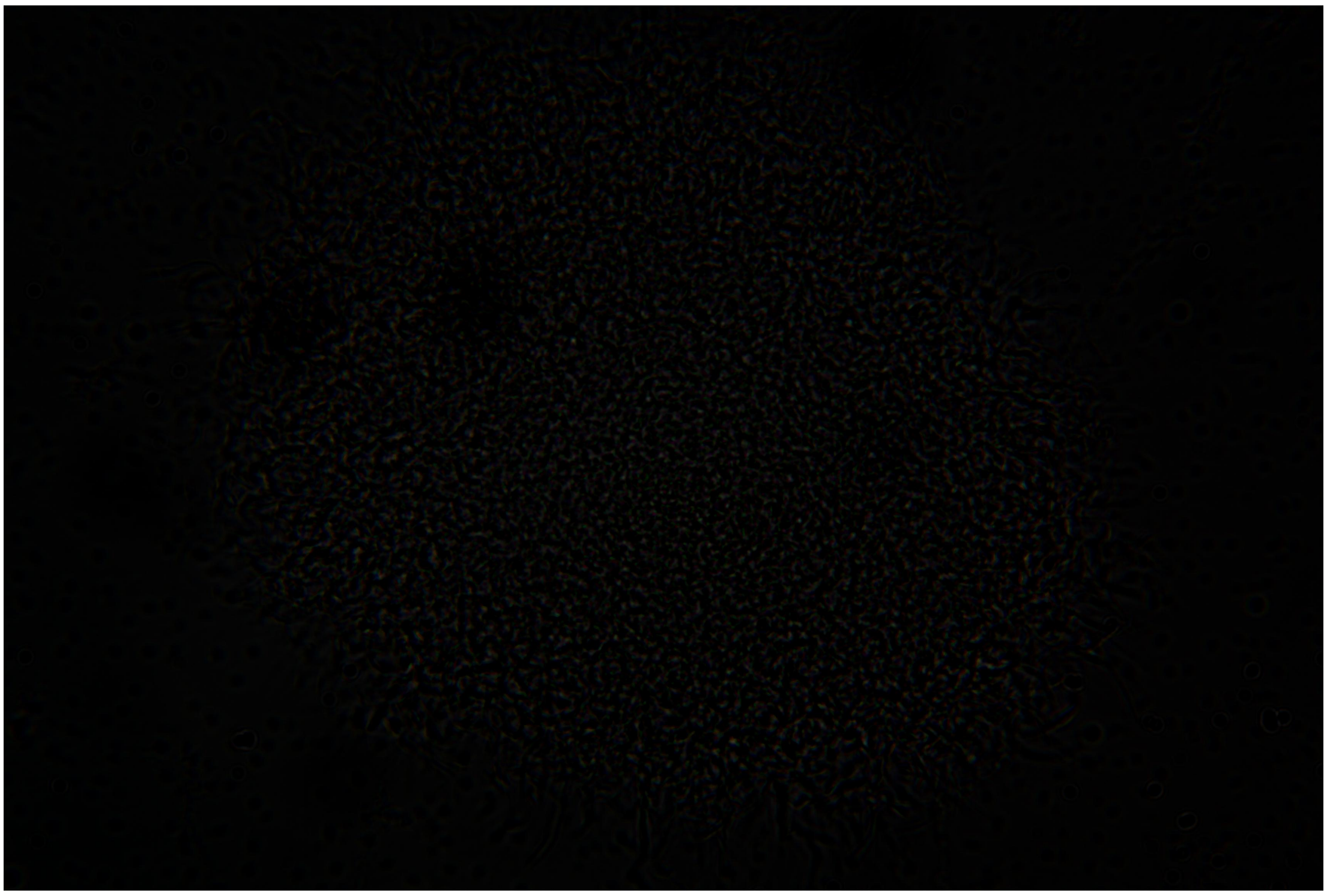

50μm 3% MG-RSO

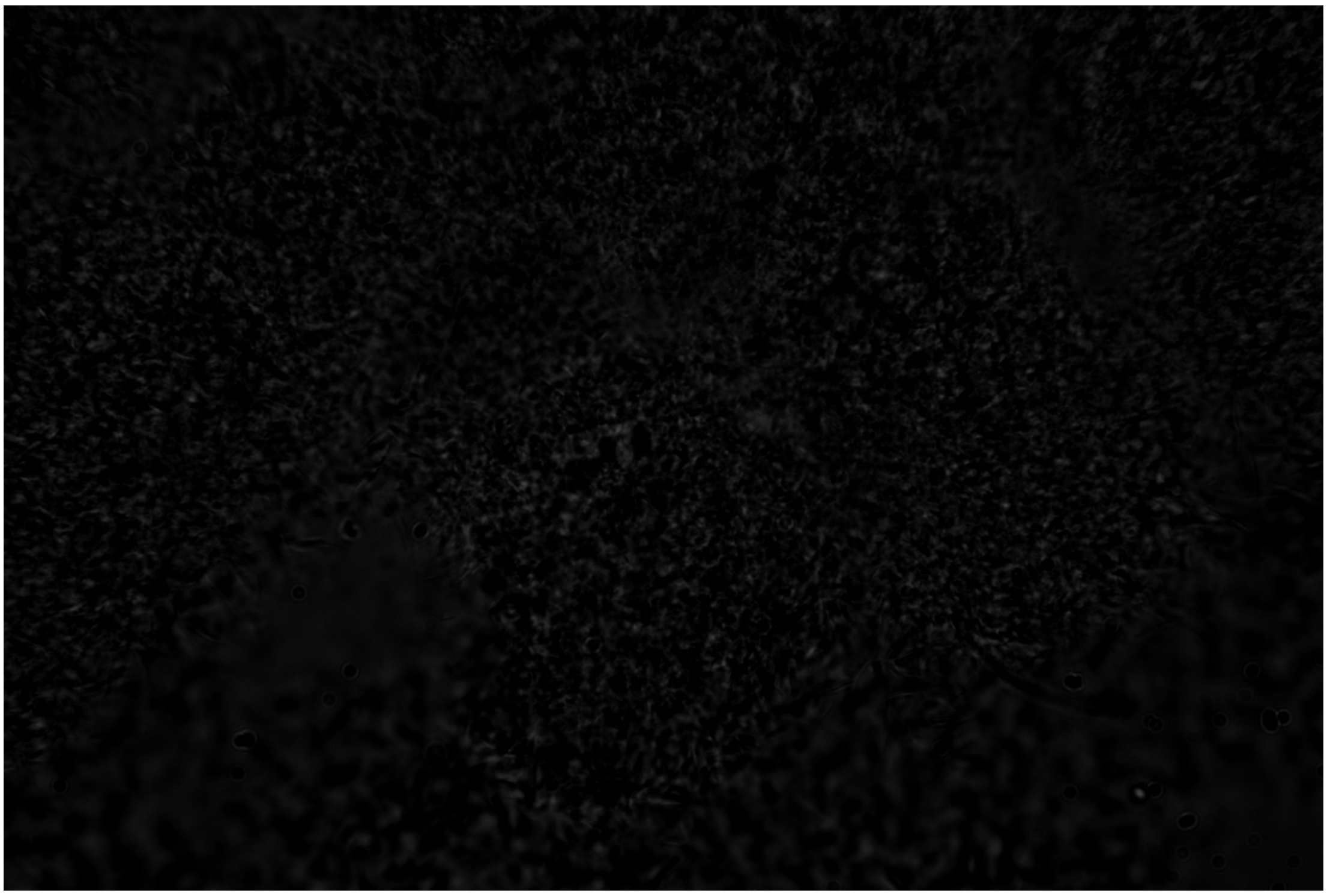

50µm 5% MG-RSO

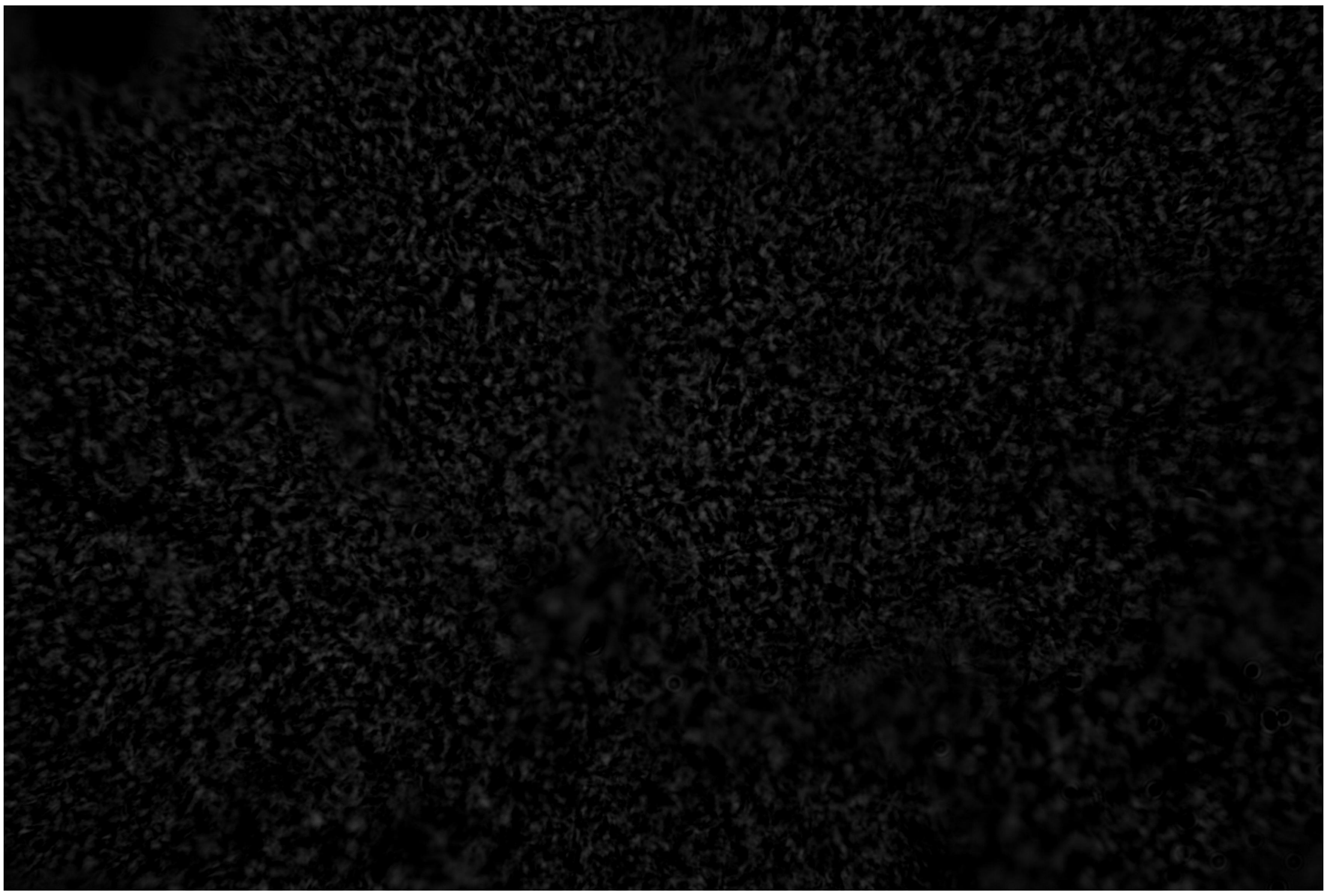

50µm 7% MG-RSO

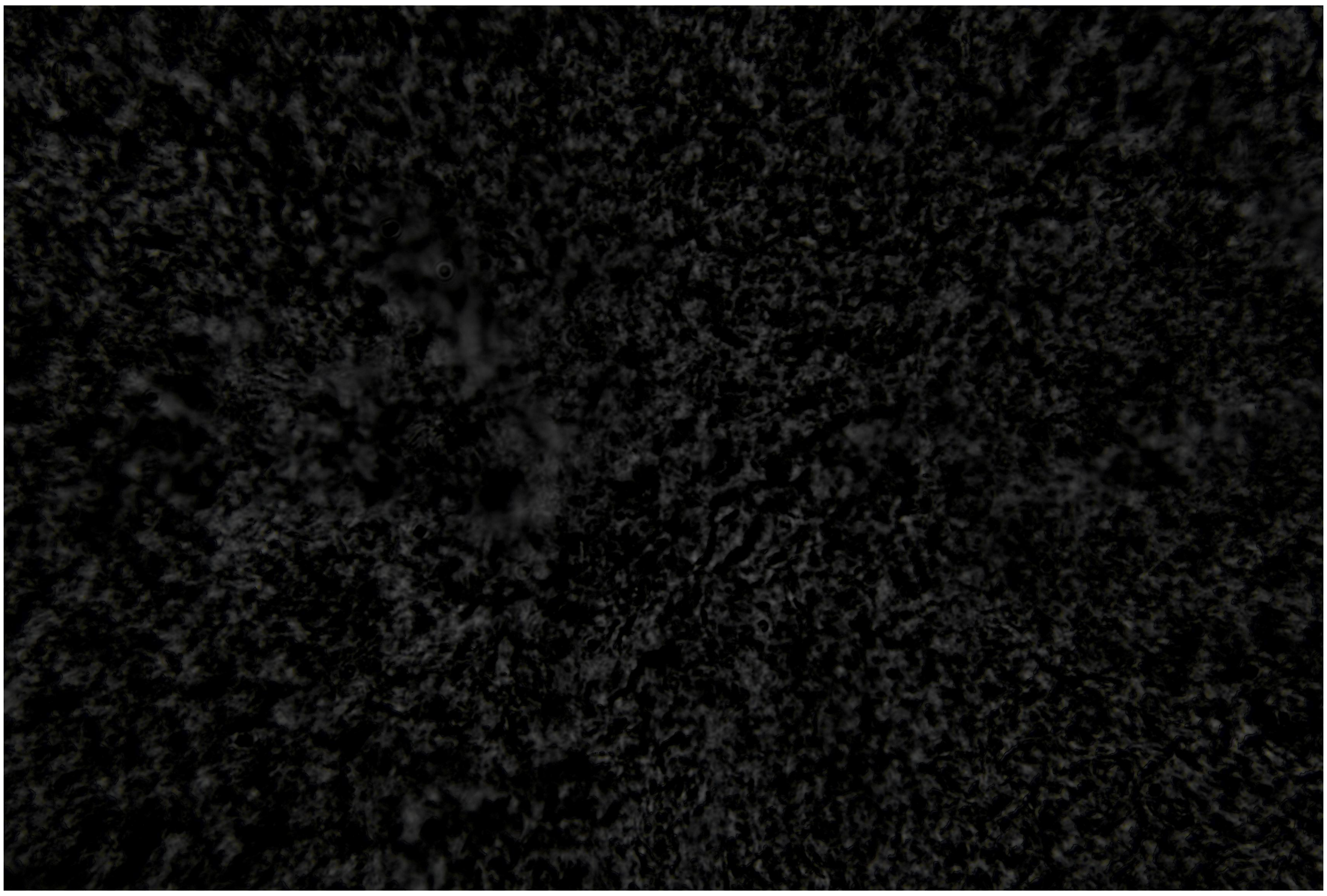

50μm 9% MG-RSO

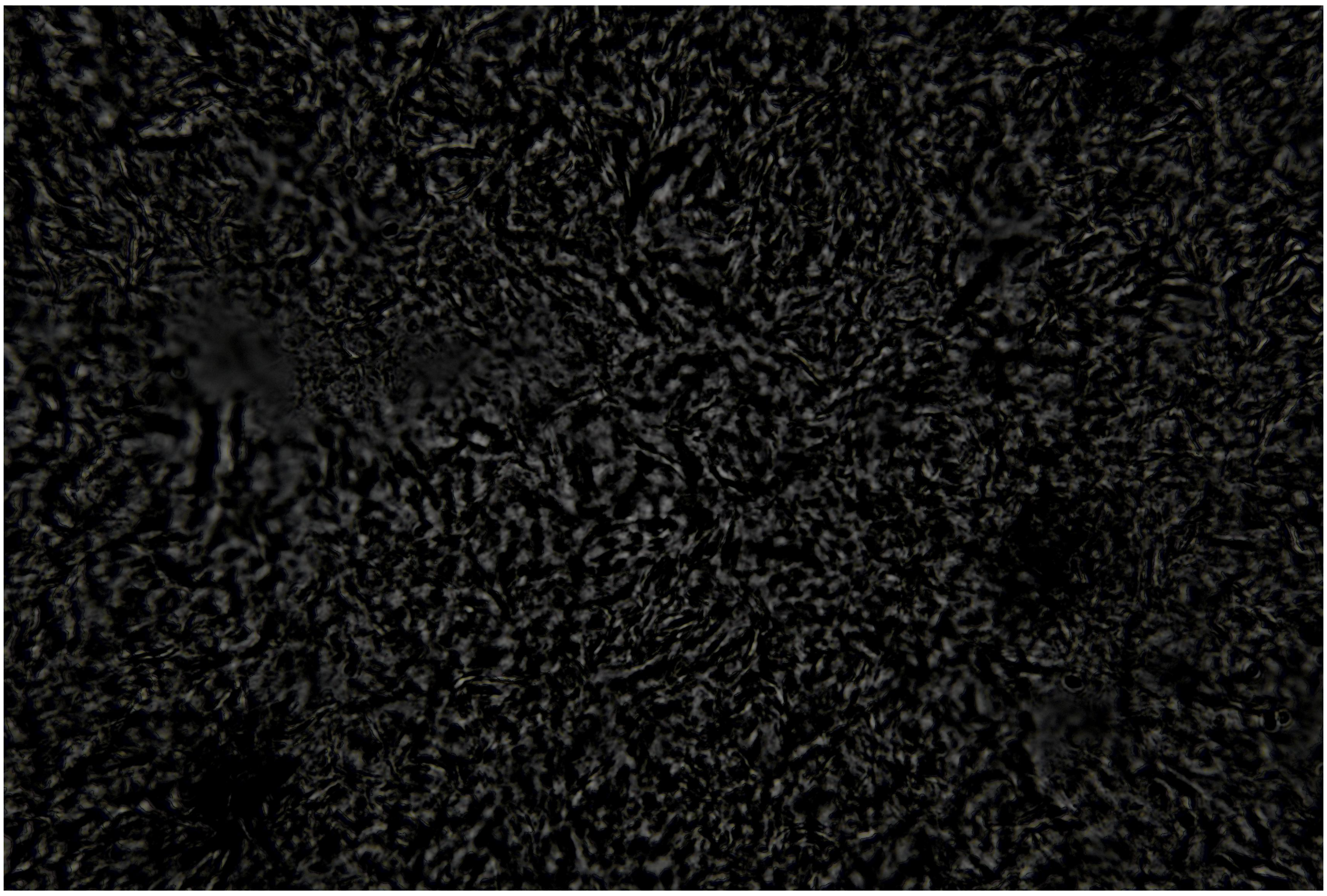

50μm 12% MG-RSO

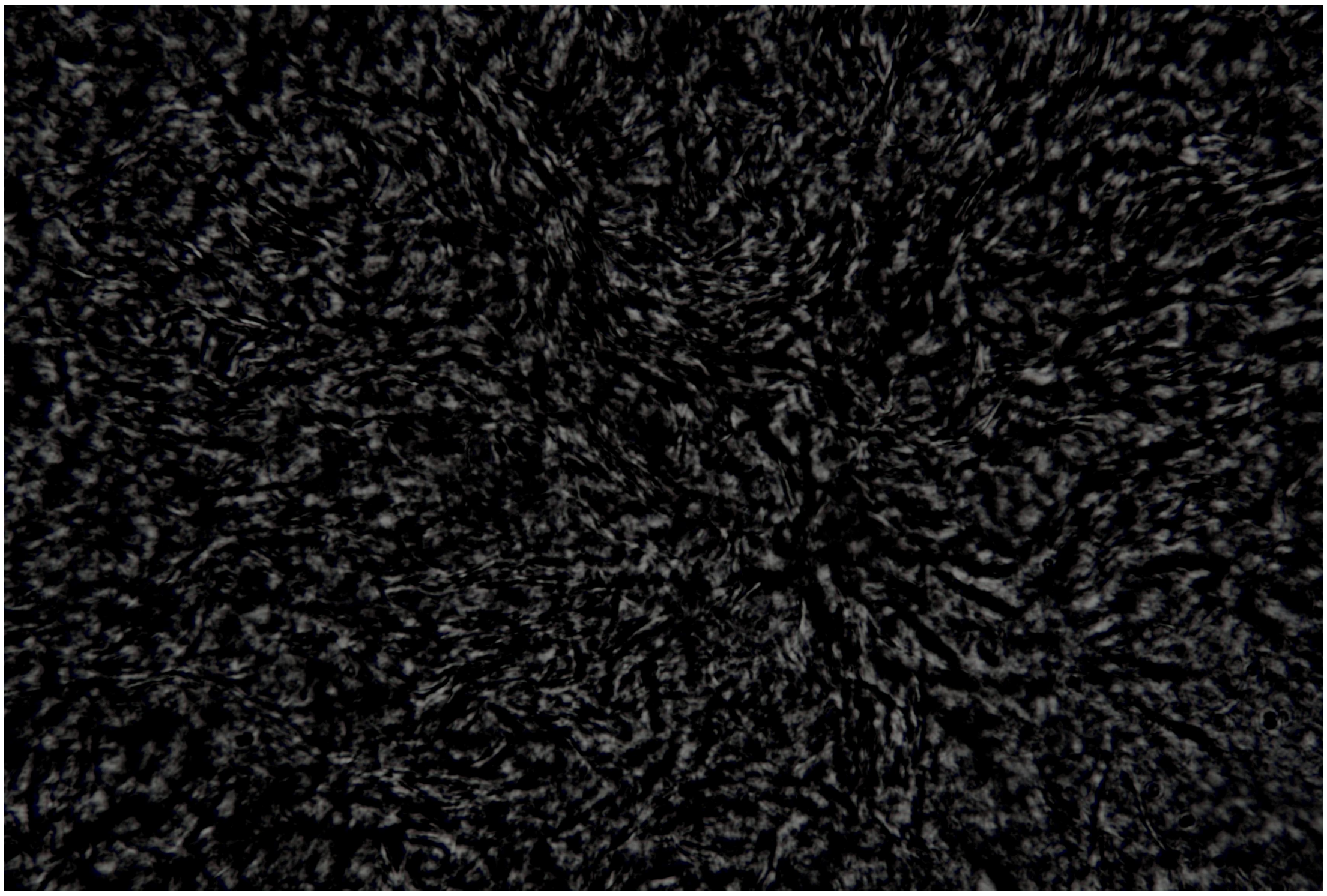

200µm 1% MG-RSO

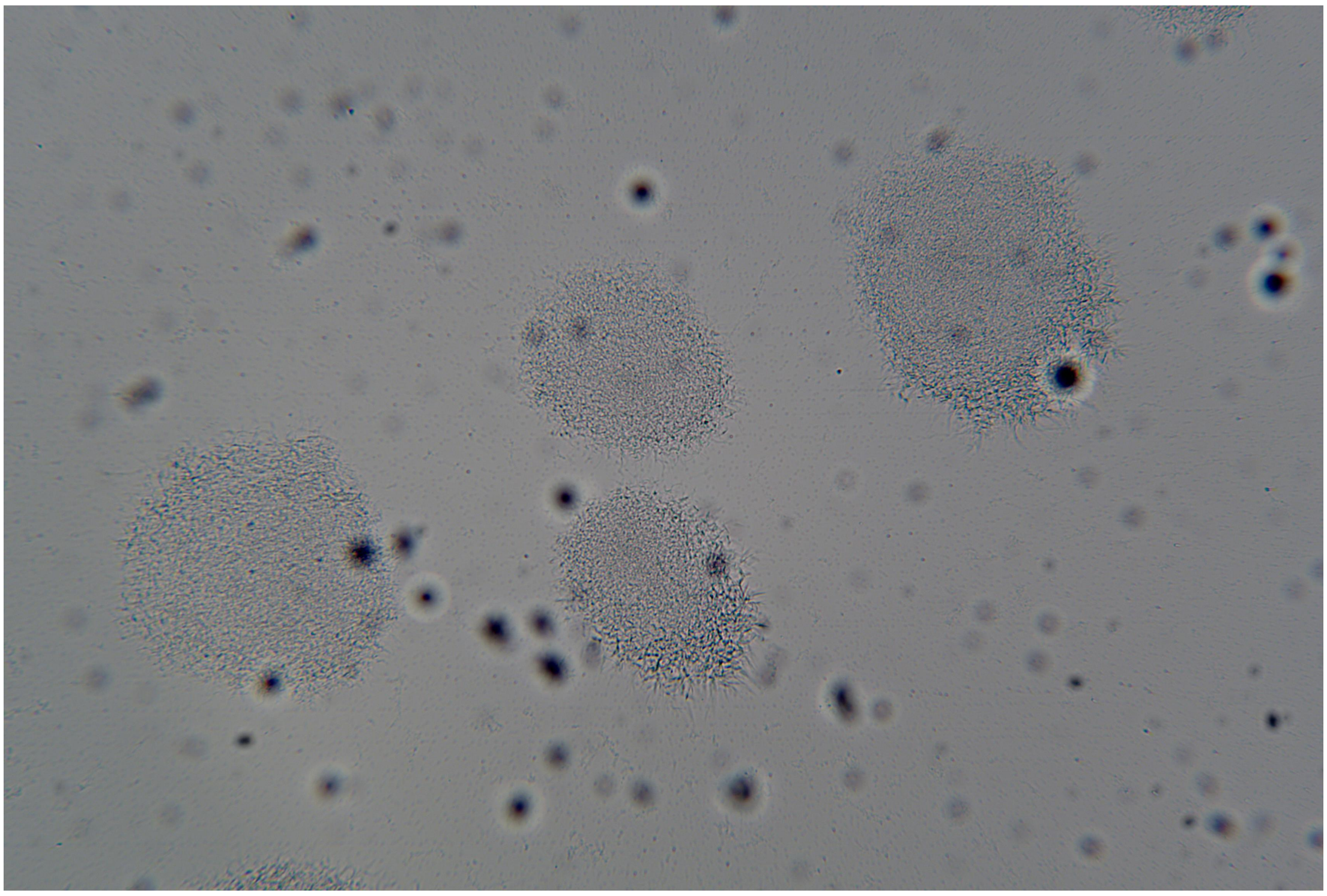

200µm 3% MG-RSO

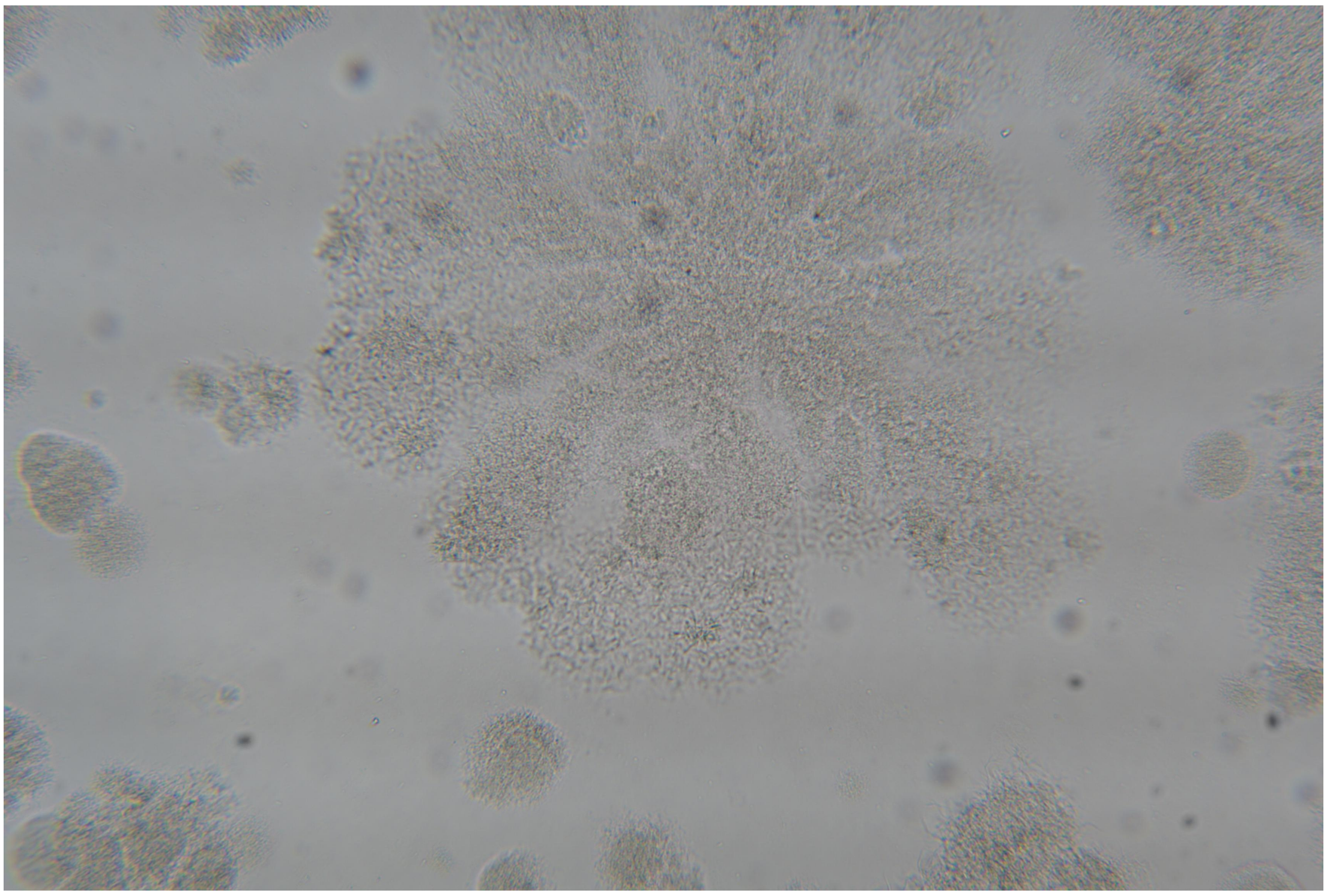

200μm 5% MG-RSO

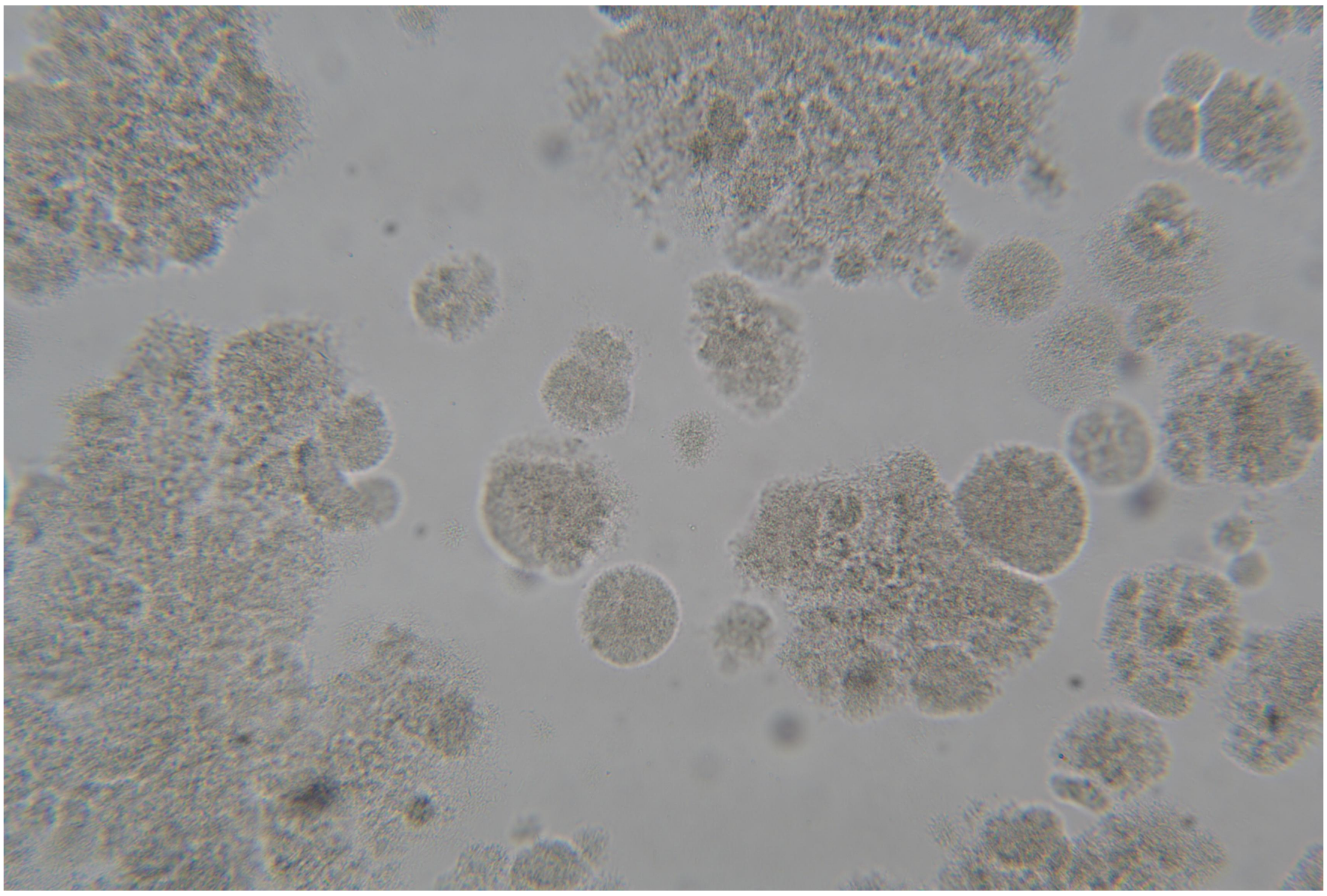

200μm 7% MG-RSO

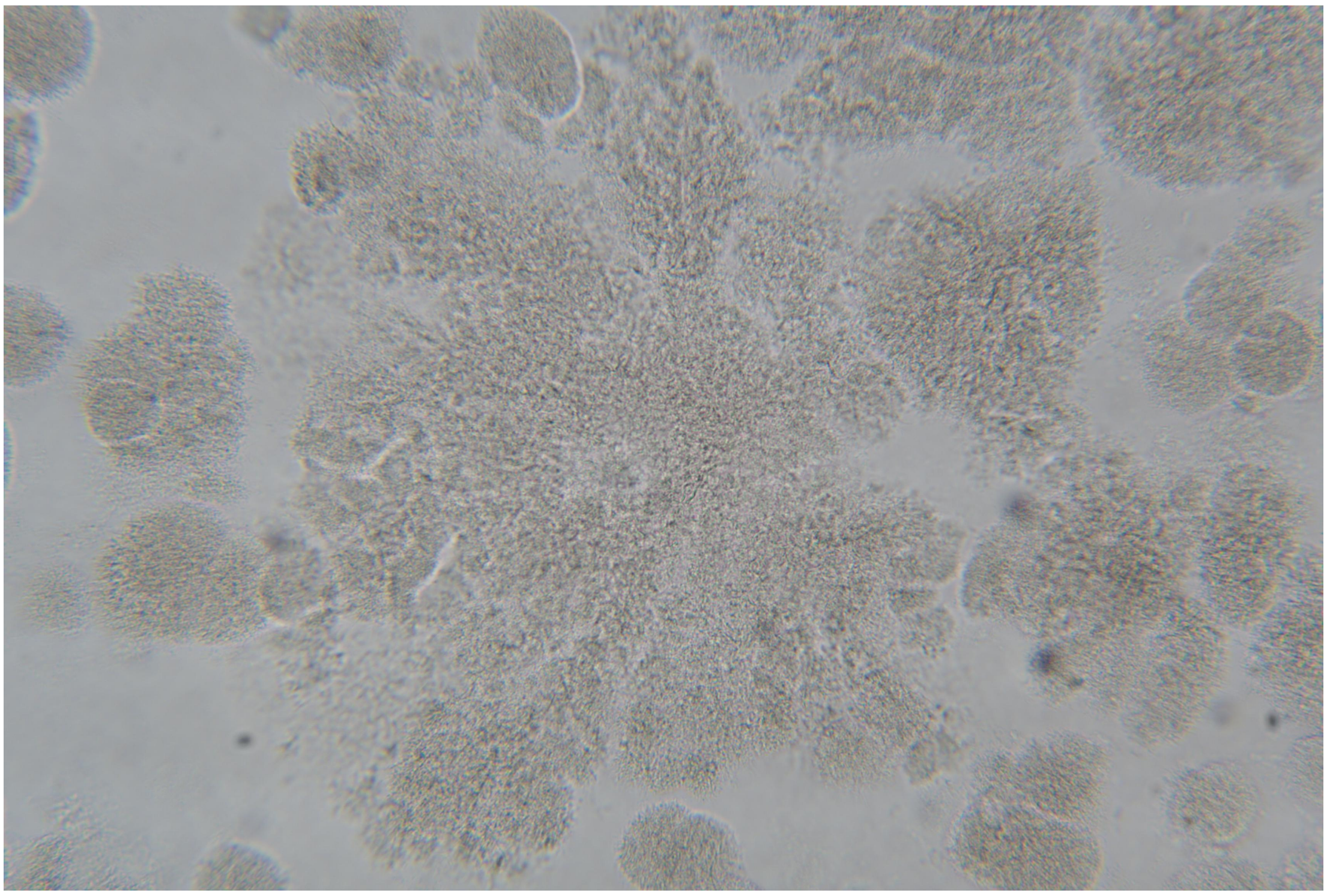

200μm 9% MG-RSO

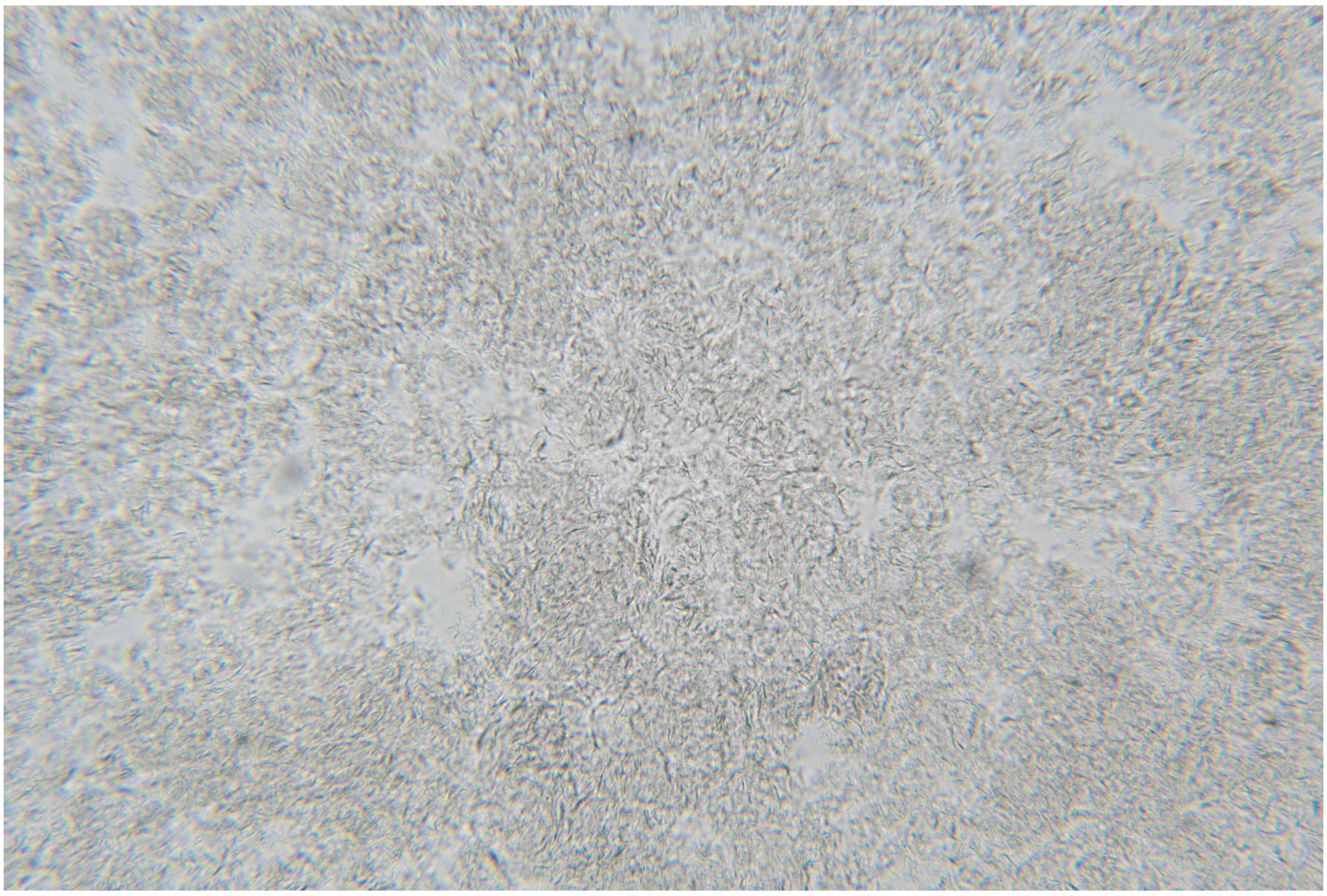

200μm 12% MG-RSO

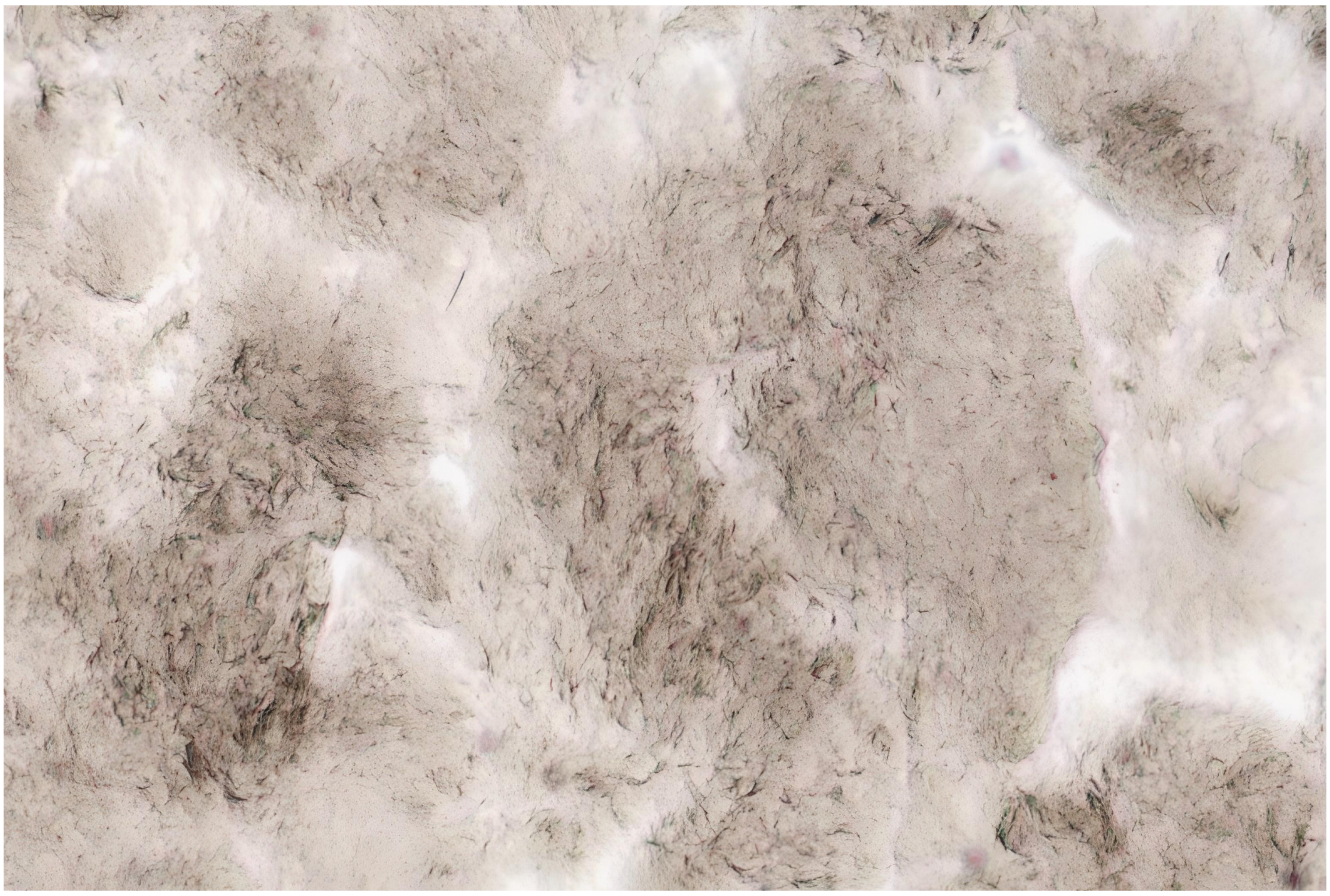

Quantitative analysis results of microscopic images

Analysis Methods：

显微图像定量分析方法说明：	Description of Quantitative Analysis Methods for Microscopic Images:
1. 图像采集：低倍光学显微镜，视野约3.34 × 2.24 mm（7.5 mm²）	1. Image acquisition: low-magnification optical microscope, field of view approximately 3.34 × 2.24 mm (7.5 mm²)
2. 比例尺校准：物镜测微尺1627 px = 1000 µm，即1 px = 0.615 µm	2. Scale calibration: objective micrometer 1627 px = 1000 µm, i.e., 1 px = 0.615 µm
3. 图像预处理：CLAHE对比度增强 + 中值滤波（5×5）去噪	3. Image preprocessing: CLAHE contrast enhancement + median filtering (5×5) for denoising
4. 图像分割：自适应高斯阈值（blockSize=101, C=5），形态学开闭运算清理	4. Image segmentation: adaptive Gaussian thresholding (blockSize=101, C=5), morphological opening and closing operations for cleanup
5. 连通区域分析：8连通，最小面积阈值80 px（≈30 µm²，等效直径≈6.2 µm）	9. Network coverage: percentage of crystal area to total field area
6. 等效直径：d = 2√(A/π)，其中A为区域面积	5. Connected component analysis: 8-connectivity, minimum area threshold 80 px (≈30 µm², equivalent diameter ≈6.2 µm)
7. 长宽比：外接矩形长边/短边	6. Equivalent diameter: d = 2√(A/π), where A is the area of the region
8. 圆形度：4πA/P²，其中P为周长；1=完美圆形，<1=不规则	8. Circularity: 4πA/P², where P is the perimeter; 1 = perfect circle, <1 = irregular
9. 网络覆盖率：晶体面积占视野总面积的百分比	10. Number density: number of crystals / field area (mm²)
10. 数量密度：晶体数/视野面积（mm²）	7. Aspect ratio: major axis / minor axis of the bounding rectangle
注意：高MG浓度（9%、12%）样品中晶体融合形成连续网络，连通区域分析可能将大网络分割为多个子区域，因此数量密度和尺寸在高浓度下应结合覆盖率和形貌描述综合解读。	Note: In samples with high MG concentrations (9%, 12%), crystal fusion forms a continuous network, and connected-component analysis may segment the large network into multiple sub-regions. Therefore, at high concentrations, number density and size should be interpreted in conjunction with coverage and morphology descriptions.

Analysis Results:

Table. Quantitative analysis of MG-RSO oleogel microstructures by light microscopy

MG concentration (wt%)	1%	3%	5%	7%	9%	12%
Crystal number (per field)	1863	1830	1454	1505	773	682
Number density (crystals/mm²)	249	244	194	201	103	91
Network coverage (%)	10.2	19.1	26.9	31.5	43.3	44.5
Mean equivalent diameter (μm)	15.5 ± 15.8	20.9 ± 23.3	25.5 ± 33.2	27.9 ± 34.7	23.7 ± 69.1	21.7 ± 75.7
Median diameter (μm)	11.5	13.8	15.3	14.8	14.1	12.9
Aspect ratio (L/W)	1.37	1.45	1.45	1.43	1.42	1.46
Circularity ($4\pi A/P^2$)	0.57	0.54	0.47	0.48	0.52	0.49
Dominant morphology	Small, sparse, mostly round/oval	Round aggregates + early dendritic clusters	Rosette/dendritic aggregates, moderate density	Dendritic aggregates, partial network formation	Fine, continuous crystalline network	Dense, continuous network with small crystallites
