# Supplementary material for: Monoglyceride-structured rubber seed oil oleogels as a partial cocoa butter replacement in chocolate: physical properties, oxidative stability and applications
Source: Food Chem X. 2026 Oct 11;39:104594. doi: 10.1016/j.fochx.2026.104594 (PMC13683454; doi:10.1016/j.fochx.2026.104594)
Supplement: Supplementary file 9 [file mmc9.pdf]

(19)国家知识产权局

(12)发明专利申请

(10)申请公布号 CN 120937961 A

(43)申请公布日 2025.11.14

(21)申请号 202511211307.0

(22)申请日 2025.08.27

(71)申请人 昆明理工大学

地址 650000 云南省昆明市呈贡区景明南路727号

(72)发明人 马信　汤雅茹　黄执

(74)专利代理机构 成都九鼎天元知识产权代理有限公司 51214

专利代理师 赵丽梅

(51)Int.Cl.

A23G 1/36(2006.01)

A23G 1/46(2006.01)

A23G 1/32(2006.01)

A23G 1/38(2006.01)

权利要求书1页　说明书7页　附图4页

(54)发明名称

一种橡胶树种子油油凝胶及其制备方法和应用

(57)摘要

本发明提供一种橡胶树种子油油凝胶及其制备方法和应用，属于食品加工技术领域，所述橡胶树种子油油凝胶由88%～95%橡胶树种子油和5%～12%单硬脂酸甘油酯组成，制备时先将两者混合，加热至60～80℃搅拌20～40min使凝胶剂溶解，再于4-25℃冷却12～24h固化成型。该油凝胶持油率高达99.87%，热稳定性好且硬度合适，能有效延缓油脂氧化。特别适用于替代食品中的固体脂肪（如可可脂），用于制备健康巧克力等食品。

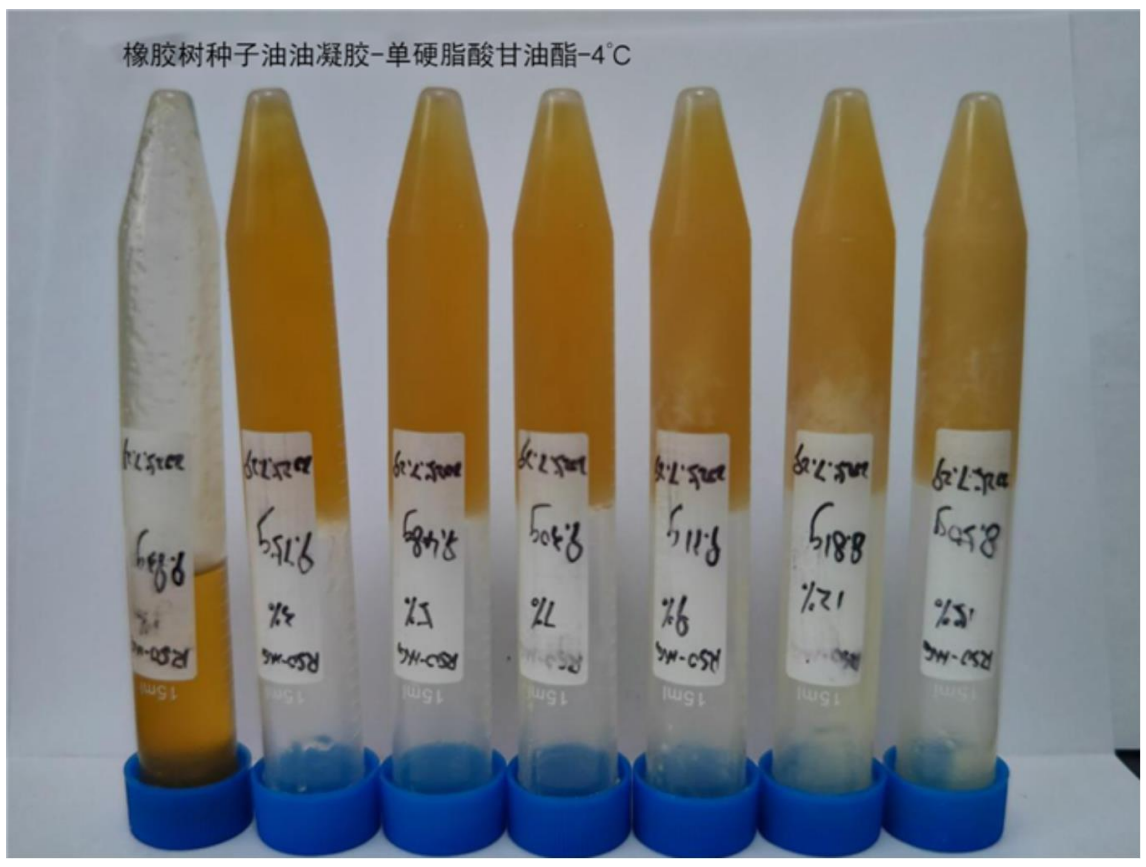

CN 120937961 A

1.一种橡胶树种子油油凝胶,由以下质量百分比的组分组成:88%～95%橡胶树种子油,5%～12%单硬脂酸甘油酯。

2.根据权利要求1所述的油凝胶,其特征在于,所述橡胶树种子油油凝胶由以下质量百分比的组分组成:91%～93%橡胶树种子油和7%～9%单硬脂酸甘油酯。

3.一种权利要求1-2任一项所述的橡胶树种子油油凝胶的制备方法,包括以下步骤:

(1) 将橡胶树种子油与单硬脂酸甘油酯预混;

(2) 将混合物加热至60～80℃,搅拌20～40min至凝胶剂完全溶解;

(3) 将溶解后的混合物冷却至4～25℃,固化12～24h,得到油凝胶。

4.根据权利要求3所述的制备方法,其特征在于:步骤(2)中加热温度为72℃,搅拌时间为40min;步骤(3)中冷却温度为4℃,固化时间为18h。

5.权利要求1-2任一项所述橡胶树种子油油凝胶在食品中的应用。

6.根据权利要求5所述应用,其特征在于,所述食品为巧克力、烘焙油脂或涂抹脂。

7.根据权利要求6所述的应用,其特征在于,在巧克力制作中用橡胶树种子油油凝胶替代可可脂。

8.根据权利要求7所述的应用,其特征在于,橡胶树种子油油凝胶在巧克力中的添加量为巧克力总质量的20%～25%。

9.一种橡胶树种子油油凝胶基巧克力,其特征在于,按重量份计,包含以下组分:45～50份可可液块;8～12份可可粉;20～25份权利要求1-2所述的油凝胶;1.5～2份大豆卵磷脂;7～9份脱脂奶粉。

10.根据权利要求9所述巧克力,其特征在于,还包括调味剂。

一种橡胶树种子油油凝胶及其制备方法和应用

技术领域

[0001] 本申请涉及食品加工技术领域,具体而言,涉及一种橡胶树种子油油凝胶及其制备方法、应用和橡胶树种子油油凝胶基巧克力。

背景技术

[0002] 油凝胶化技术相较于传统的氢化油脂工艺具有显著优势。传统氢化工艺通过高温加热使油脂中的双键与氢加成实现液体油脂固化,而油凝胶化技术则通过添加凝胶因子的物理作用实现固化。该技术首先通过凝胶因子无规则聚集形成一级结构,随后以自组装或结晶方式形成纤维状或片状聚集体构成二级结构,最终形成三维网状结构限制油脂流动。这种方法制备的固体油脂能显著降低饱和脂肪酸和反式脂肪酸含量,避免长期摄入这些物质导致低密度脂蛋白胆固醇升高,从而降低动脉硬化和心脏病风险。

[0003] 凝胶剂的选择对油凝胶性能至关重要。目前常用的凝胶剂包括单硬脂酸甘油酯、植物甾醇和卵磷脂等。根据凝胶机制可分为四类:晶体粒子网络通过结晶与聚集形成网络结构;自组装纤维网络通过一维生长形成纤维网状结构;聚合物网络通过温度变化形成珊瑚状网络;以及间接凝胶网络的乳液模型。不同机制形成的凝胶具有不同的理化特性,可根据具体应用场景选择合适的凝胶因子。

[0004] 橡胶树种子作为天然橡胶产业的副产品,产量丰富但利用率低。其含油量超过50%,经精加工可获得营养丰富的食用油脂。橡胶树种子油富含软脂酸、硬脂酸、油酸、亚油酸和亚麻酸,其中ω-3多不饱和脂肪酸含量超过20%,ω-6多不饱和脂肪酸含量超过40%,且两者比例约为1:2,具有较高的食用价值。科学研究证实该油脂具有降血脂、降血压功能,是一种安全优质的植物油脂。开发橡胶树种子油油凝胶可拓展其应用范围,为消费者提供更健康的食品选择,但目前尚未见相关研究和应用报道。

发明内容

[0005] 本申请的目的在于提供一种橡胶树种子油油凝胶及其制备方法、应用和橡胶树种子油油凝胶基巧克力,具有提高橡胶树种子利用率、避免引入有害脂肪酸、提供健康食品原料的优点。

[0006] 为达到本发明目的,本发明采用以下技术方案:

首先,本发明提供了一种橡胶树种子油油凝胶,其由以下质量百分比的组分组成:88%~95%橡胶树种子油,5%~12%单硬脂酸甘油酯。

[0007] 橡胶树种子油是指从橡胶树种子中提取的植物油,具体可以采用压榨法或溶剂萃取法获得,其含有超过20%的ω-3多不饱和脂肪酸和40%的ω-6多不饱和脂肪酸。单硬脂酸甘油酯是指由甘油与硬脂酸酯化形成的食品级乳化剂,具体可以采用酯交换工艺制备,其分子结构中的疏水基团与亲水基团可促进晶体网络形成。

[0008] 单硬脂酸甘油酯浓度在5%~12%时能够确保凝胶体系在维持三维网状结构的同时保留橡胶树种子油的高营养价值。5%~12%单硬脂酸甘油酯能够促进纤维状聚集体的形成,

避免因凝胶剂含量过低导致结构强度不足或含量过高导致油脂流动性被过度抑制。当橡胶树种子油占比控制在88%～95%时，既能为网络结构提供足够的空间支撑，又能保留油脂中ω-3与ω-6多不饱和脂肪酸的活性。尤其是单硬脂酸甘油酯占比控制在7%～9%时，单硬脂酸甘油酯浓度在5%～12%时能够确保凝胶体系在维持三维网状结构的同时保留橡胶树种子油的高营养价值。5%～12%单硬脂酸甘油酯能够促进纤维状聚集体的形成，避免因凝胶剂含量过低导致结构强度不足或含量过高导致油脂流动性被过度抑制。当橡胶树种子油占比控制在88%～95%时，既能为网络结构提供足够的空间支撑，又能保留油脂中ω-3与ω-6多不饱和脂肪酸的活性。尤其是单硬脂酸甘油酯占比控制在7%～9%时，其结晶行为与油相的相容性达到平衡，形成的网络结构孔隙率适中，可有效限制油脂迁移，具有较好的持油率、硬度和热可逆性。

[0009]　优选地，所述橡胶树种子油油凝胶由以下质量百分比的组分组成：91%～93%橡胶树种子油和7%～9%单硬脂酸甘油酯。在该比例范围，其结晶行为与油相的相容性达到平衡，形成的网络结构孔隙率适中，可有效限制油脂迁移，具有较好的持油率、硬度和热可逆性。

[0010]　优选地，所述橡胶树种子油油凝胶由以下质量百分比的组分组成：93%橡胶树种子油和7%单硬脂酸甘油酯。

[0011]　其次，本发明还提供上述橡胶树种子油油凝胶的制备方法，其包括以下步骤：

(1) 将橡胶树种子油与单硬脂酸甘油酯预混；

(2) 将混合物加热至60-80℃，搅拌20～40min至凝胶剂完全溶解；

(3) 将溶解后的混合物冷却至4-25℃，固化12～24h，得到油凝胶。

[0012]　优选地，步骤(2)中加热温度为72℃，搅拌时间为40min；

优选地，步骤(3)中冷却温度为4℃，固化时间为18h。

[0013]　预混是指将液态油脂与凝胶剂进行初步混合的操作，具体可以采用机械搅拌或超声震荡来实现，该步骤能够确保凝胶剂在油相中均匀分散，避免后续加热过程中局部结块。其中，加热温度60-80℃是指通过外部热源使混合物达到的温控区间，具体可以采用水浴加热或电热套加热来实现，该温度范围既能保证单硬脂酸甘油酯充分溶解，又可避免高温导致油脂氧化分解。其中，搅拌时间20～40min是指维持机械搅拌的持续时间，具体可以采用磁力搅拌器或桨式搅拌器来实现，该时长能够确保凝胶剂分子充分扩散并与油脂形成均相体系。其中，冷却温度4-25℃是指将熔融态混合物降温至的固化区间，具体可以采用自然冷却或冷藏设备来实现，该温度梯度有利于凝胶剂分子定向排列形成三维网络结构。其中，固化时间12～24h是指维持冷却状态的时间跨度，具体可以采用恒温培养箱或冷库来实现，该时段能够保证凝胶网络充分发育并达到稳定状态。

[0014]　在预混阶段通过物理混合使单硬脂酸甘油酯均匀分布于橡胶树种子油中，形成初步分散体系。随后在加热过程中，热能促使凝胶剂分子克服晶格能实现完全溶解，同时搅拌作用加速分子扩散形成均质溶液。当温度降至凝胶剂结晶点时，分子通过氢键或范德华力自组装形成纤维状聚集体，随着温度持续下降，聚集体进一步交联形成三维网状结构，最终将液态油脂包裹固定形成固态凝胶。整个过程无需化学改性，仅通过物理相变实现油脂固化。

[0015]　再次，本发明还提供了上述橡胶树种子油油凝胶在食品中的应用。

[0016]　所述食品中的应用是指将油凝胶作为固体脂肪替代物融入食品加工体系。通过替

代传统氢化油脂或高饱和脂肪成分,能够降低食品中饱和脂肪酸含量,同时利用橡胶树种子油的天然营养成分提升食品健康价值。

[0017] 优选地,所述食品为巧克力、烘焙油脂或涂抹脂。

[0018] 通过替代传统氢化油脂或高饱和脂肪成分,能够降低食品中饱和脂肪酸含量,同时利用橡胶树种子油的天然营养成分提升食品健康价值。

[0019] 优选地,在巧克力制作中用橡胶树种子油油凝胶替代可可脂。

[0020] 优选地,橡胶树种子油油凝胶在巧克力中的添加量为巧克力总质量的20%～25%。

[0021] 最后,本发明还提供了一种橡胶树种子油油凝胶基巧克力,按重量份计,包含以下组分:45～50份可可液块;8～12份可可粉;20～25份权利要求1-2所述的油凝胶;1.5～2份大豆卵磷脂;7～9份脱脂奶粉;

优选地,所述橡胶树种子油油凝胶基巧克力还包含调味剂,

优选地,所述调味剂为香兰素、牛奶香精和枫糖浆。

[0022] 在巧克力制作中,油凝胶替代可可脂,利用其低温固化能力形成光滑质地,同时避免氢化油脂的高温加工需求。凝胶网络对液态油的包裹作用可维持食品形态稳定性,而橡胶树种子油中ω-3与ω-6脂肪酸的合理比例则赋予食品功能性营养特性。

[0023] 本发明有益效果:

本发明采用特定比例的单硬脂酸甘油酯作为凝胶剂,通过油凝胶体系将橡胶树种子油固体凝胶化,可以有效阻止空气在油液中的流动,同时也减少油液与空气的接触,减缓油脂的氧化过程。所得橡胶树油凝胶具有较好的持油率、硬度和热可逆性,可广泛应用于巧克力、烘焙油脂或涂抹脂的食品的制作,不仅为大众提供多一种健康油脂选择,同时也丰富了橡胶树种子油在食品加工领域的应用,具有独特的优势。

附图说明

[0024] 图1为实施例1-5及对比例1-3制备油凝胶在-4℃条件下保存样品照片;

图2为实施例1-5及对比例1-3制备油凝胶在25℃条件下保存样品照片;

图3为对比例4-10制备油凝胶在-4℃条件下保存样品照片;

图4为对比例4-10制备油凝胶在25℃条件下保存样品照片;

图5油凝胶热稳定分析结果:图中RSO-MG(1%wt)为对比例1样品,RSO-MG(3%wt)为对比例2样品,RSO-MG(5%wt)为实施例1样品,RSO-MG(7%wt)为实施例2样品,RSO-MG(9%wt)为实施例3样品,RSO-MG(12%wt)为实施例4样品);

图6油凝胶硬度测试结果;

图7 油凝胶基巧克力照片;

图8油凝胶基巧克力的DSC图。

具体实施方式

[0025] 下面结合附图和实施例对本发明作进一步的详细说明,但不以任何方式对本发明加以限制,基于本发明教导所作的任何变换或改进,均落入本发明的保护范围。

[0026] 实施本发明的过程、条件、试剂、实验方法等,除以下专门提及的内容之外,均为本领域的普遍知识和公知常识,本发明没有特别限制内容。各实施例中未注明具体条件的实

验方法,通常按照常规条件或按照制造厂商所建议的条件。

[0027] 除非另有说明,本说明书中使用的全部专业术语和科学用语的含义均与本发明所属技术领域的技术人员一般理解的含义相同。但如有冲突,以包含定义的本说明书为准。

[0028] 以下为实施例中主要材料来源:

单硬脂酸甘油酯:河南俊一生物科技,100g/袋;

橡胶树种子油:西双版纳华坤生物科技有限责任公司,1L/瓶;

蜂蜡:亳州市福硕堂生物科技有限公司,100g/盒。

[0029] 实施例1

一种橡胶树种子油油凝胶,其由以下质量百分比的组分组成:5%橡胶树种子油,95%单硬脂酸甘油酯。

[0030] 其制备方法如下:

(1) 将橡胶树种子油与单硬脂酸甘油酯预混;

(2) 将混合物加热至72℃,搅拌40min至凝胶剂完全溶解;

(3) 将溶解后的混合物冷却至4℃,固化18h,得到油凝胶。

[0031] 实施例2

一种橡胶树种子油油凝胶,其由以下质量百分比的组分组成:93%橡胶树种子油,7%单硬脂酸甘油酯。

[0032] 其制备方法如下:

(1) 将橡胶树种子油与单硬脂酸甘油酯预混;

(2) 将混合物加热至60℃,搅拌40min至凝胶剂完全溶解;

(3) 将溶解后的混合物冷却至25℃,固化24h,得到油凝胶。

[0033] 实施例3

一种橡胶树种子油油凝胶,其由以下质量百分比的组分组成:91%橡胶树种子油,9%单硬脂酸甘油酯。

[0034] 其制备方法如下:

(1) 将橡胶树种子油与单硬脂酸甘油酯预混;

(2) 将混合物加热至80℃,搅拌20min至凝胶剂完全溶解;

(3) 将溶解后的混合物冷却至4℃,固化12h,得到油凝胶。

[0035] 实施例4

一种橡胶树种子油油凝胶,其由以下质量百分比的组分组成:88%橡胶树种子油,12%单硬脂酸甘油酯。

[0036] 其制备方法如下:

(1) 将橡胶树种子油与单硬脂酸甘油酯预混;

(2) 将混合物加热至72℃,搅拌40min至凝胶剂完全溶解;

(3) 将溶解后的混合物冷却至4℃,固化18h,得到油凝胶。

[0037] 对比例1

与实施例1的区别在于,单硬脂酸甘油酯的量为1%,其余与实施例1一致。

[0038] 对比例2

与实施例1的区别在于,单硬脂酸甘油酯的量为3%,其余与实施例1一致。

[0039] 对比例3

与实施例1的区别在于,单硬脂酸甘油酯的量为15%,其余与实施例1一致。

[0040] 图1-2为实施例1-5及对比例1-3制备油凝胶在-4℃和25℃条件下保存样品照片。

[0041] 对比例4-10

与实施例1的区别在于油凝胶剂为蜂蜡,添加量分别为:1%、3%、5%、7%、9%、12%、15%,制备方法如下:

(1) 将橡胶树种子油与蜂蜡预混;

(2) 将混合物加热至72℃,搅拌1.5h至凝胶剂完全溶解;

(3) 将溶解后的混合物冷却至4℃,固化24h,得到油凝胶。

[0042] 图3-4为对比例4-10制备油凝胶在-4℃和25℃条件下保存样品照片。

[0043] 实施例5 性能测试实验

1 持油率分析

采用离心方法(5000 rpm,15min,25℃)分析各实施例1-4及对比例1-10油凝胶的持油率。结果以通过离心法分离去除游离油后的质量除以油凝胶样品的质量的百分比来表示,计算公式如下:持油率=m/M×100%。式中:m为离心去除游离油后油凝胶样品的质量(g),M为油凝胶样品的质量(g)。结果见表1。

[0044] 表1 实施例1-4及对比例1-10油凝胶持油率

凝胶因子及	RSO油凝胶持油率（%）						
其含量	1%	3%	5%	7%	9%	12%	15%
单硬脂酸甘油酯	24.92	58.95	86.43	97.10	97.84	98.91	99.87
蜂蜡	/	/	62.93	92.33	93.77	94.81	97.93

[0045] 以上结果表明,单硬脂酸甘油酯作为油凝胶剂具有较好的持油率。

[0046] 2热稳定性分析

使用差示扫描量热仪对实施例1-4及对比例1-2制备样品进行热分析样。具体来说,将5-10 mg的油凝胶样品热平衡后,样品在30 mL/min的连续N2环境下以5℃/min的速率加热至85℃,并保持10分钟以消除晶体记忆效应,随后以5℃/min的速率冷却至0℃。通过测量冷却和加热过程中热流与温度的关系,分析样品的结晶过程(85-0℃,5℃/min)和熔化过程(0-85℃,5℃/min)。

[0047] 结果见图5,结果显示,所有样品的熔化曲线都显示了两个峰值:一个较小的峰值出现在低温(5-10℃),另一个较大的峰值则出现在高温(60-70℃),这表明MG以不同的多晶型形式存在。所有样品的冷却曲线都显示在35-55℃下凝固,且具有两个连续峰。多个峰值的存在表明MG以不同的多晶型形式存在,且可以通过温度进行控制。且随着MG含量的增加,RSO油凝胶的熔点也逐渐升高,凝固点也略有降低,从而提升RSO油凝胶的热稳定性。

[0048] 3硬度分析

在常温下,采用配备圆柱探头(P/0.5)的质构仪测定实施例1-4及对比例1-2制备RSO-MG油凝胶的硬度。具体步骤如下:先将10g熔化的样品熔化后置于EP管中,4℃冰箱过夜,使其凝固塑形后的厚度大于20mm。测试时,样品在25℃下热平衡,并按照以下条件进行质构分析:测试探头下降速度为1 mm/s,位移总行程为10mm,力量感应元为5g。最终,凝胶硬度以在测试行程中产生的最大抗力来表示。

[0049] 结果如图6所示,结果表明,当MG浓度从1.0%增加至12.0%时,油凝胶的硬度呈现显著提升态势,最大触发应力由20 g增加至1228 g,这亦表明油凝胶的硬度与MG浓度呈正相关关系。该结果说明MG于油凝胶中构建了相对稳定的三维网络结构,随着MG浓度的升高,橡胶树种子油油凝胶的硬度得到大幅提高。

4长期存放后的持油率测试

测试样品为4℃冰箱封盖存放3个月后的实施例1-4及对比例1-2制备的RSO油凝胶。采用高速离心方法(9000 rpm,15min,25℃)分析RSO油凝胶的持油率。结果以通过离心法分离去除游离油后的质量除以油凝胶样品的质量的百分比来表示,计算公式如下:持油率=m/M×100%。式中:m为离心去除游离油后油凝胶样品的质量(g),M为油凝胶样品的质量(g)。

[0050] 实验结果见表2,结果显示,放置3个月后,随着MG浓度从1.0%增至12.0%,OBC值从18.1%跃升至92.9%。这一结果表明MG在油凝胶中形成了稳定的三维网络结构,即使RSO油凝胶经过长时间的存放,也具有较高的持油率。

[0051] 表2 长期存放后的橡胶树种子油油凝胶持油率

RSO-MG 油凝胶 长期保存持油率测试						
MG浓度	对比例1	对比例2	实施例1	实施例2	实施例3	实施例4
持油率均值	18.11%	40.93%	67.13%	76.27%	82.91%	92.93%

[0052] 以上结果说明,单硬脂酸甘油酯浓度在5%～12%时能够确保凝胶体系在维持三维网状结构的同时保留橡胶树种子油的高营养价值。5%～12%单硬脂酸甘油酯能够促进纤维状聚集体的形成,避免因凝胶剂含量过低导致结构强度不足或含量过高导致油脂流动性被过度抑制。当橡胶树种子油占比控制在88%～95%时,既能为网络结构提供足够的空间支撑,又能保留油脂中ω-3与ω-6多不饱和脂肪酸的活性。尤其是单硬脂酸甘油酯占比控制在7%～9%时,其结晶行为与油相的相容性达到平衡,形成的网络结构孔隙率适中,可有效限制油脂迁移,具有较好的持油率、硬度和热可逆性。

[0053] 实施例6 橡胶树种子油油凝在巧克力制作中的应用

巧克力配方如下:按重量份计,包含可可液块50份;可可粉10分;实施例2制备RSO-MG油凝胶25份;大豆卵磷脂2份;香兰素1份;脱脂奶粉8份;牛奶香精1份;枫糖浆3份。

[0054] 制备方法为:在80℃条件下水浴加热可可液块、油凝胶和可可粉的混合物,完全熔化后继续搅拌至混合完全,得到可可原浆;少量多次加入调味剂(香兰素、牛奶香精、枫糖浆)、大豆卵磷脂、脱脂奶粉,不断搅拌至组分分布均匀,得到巧克力原浆。然后将80℃的巧克力原浆放入冷水中冷却至35℃,取出继续搅拌至27℃,再加热至32℃,多次进行27℃冷

却、32℃加热操作,直至巧克力原浆变得丝滑、黏稠。最后将27℃丝滑的巧克力原浆注入模具后,冷却至室温后,放入-4℃冰箱中再度冷却20min,即可脱模得到含有不同浓度的橡胶树种子油油凝胶基巧克力,巧克力照片如图7。

[0055] 巧克力的熔化行为对其功能性和口感至关重要。因此,使用差示扫描量热法(DSC)来表征巧克力样品从10到85℃的熔化曲线。橡胶树种子油油凝胶基巧克力在冷却和加热过程中的DSC曲线见图8。由DSC曲线可知,0-20℃符合RSO-MG油凝胶的融化曲线,到第二个融化峰时,油凝胶基巧克力于32.11℃开始融化,42.38℃融化完毕,融化峰峰值位于38.84℃,所需焓值在28.35J/g,满足巧克力入口即化的特点(融点36-39℃),可以作为可可脂的优质替代物,健康且营养。

[0056] 实施例7 橡胶树种子油油凝在巧克力制作中的应用

与实施例6的区别在于,其配方,按重量份计,包含可可液块45份;可可粉12分;实施例2制备 RSO-MG油凝胶20份;大豆卵磷脂1.5份;香兰素1份;脱脂奶粉9份;牛奶香精1份;枫糖浆3份。

[0057] 制备方法同实施例6。

[0058] 实施例8橡胶树种子油油凝在巧克力制作中的应用

与实施例6的区别在于,其配方,按重量份计,包含可可液块48份;可可粉8分;实施例2制备 RSO-MG油凝胶23份;大豆卵磷脂1.8份;香兰素1份;脱脂奶粉7份;牛奶香精1份;枫糖浆3份。

[0059] 制备方法同实施例6。

[0060] 最后应说明的是:以上实施例仅用以说明本发明的技术方案,而非对其限制;尽管参照前述实施例对本发明进行了详细的说明,本领域的普通技术人员应当理解:其依然可以对前述各实施例所记载的技术方案进行修改,或者对其中部分技术特征进行等同替换;而这些修改或者替换,并不使相应技术方案的本质脱离本发明各实施例技术方案的精神和范围。

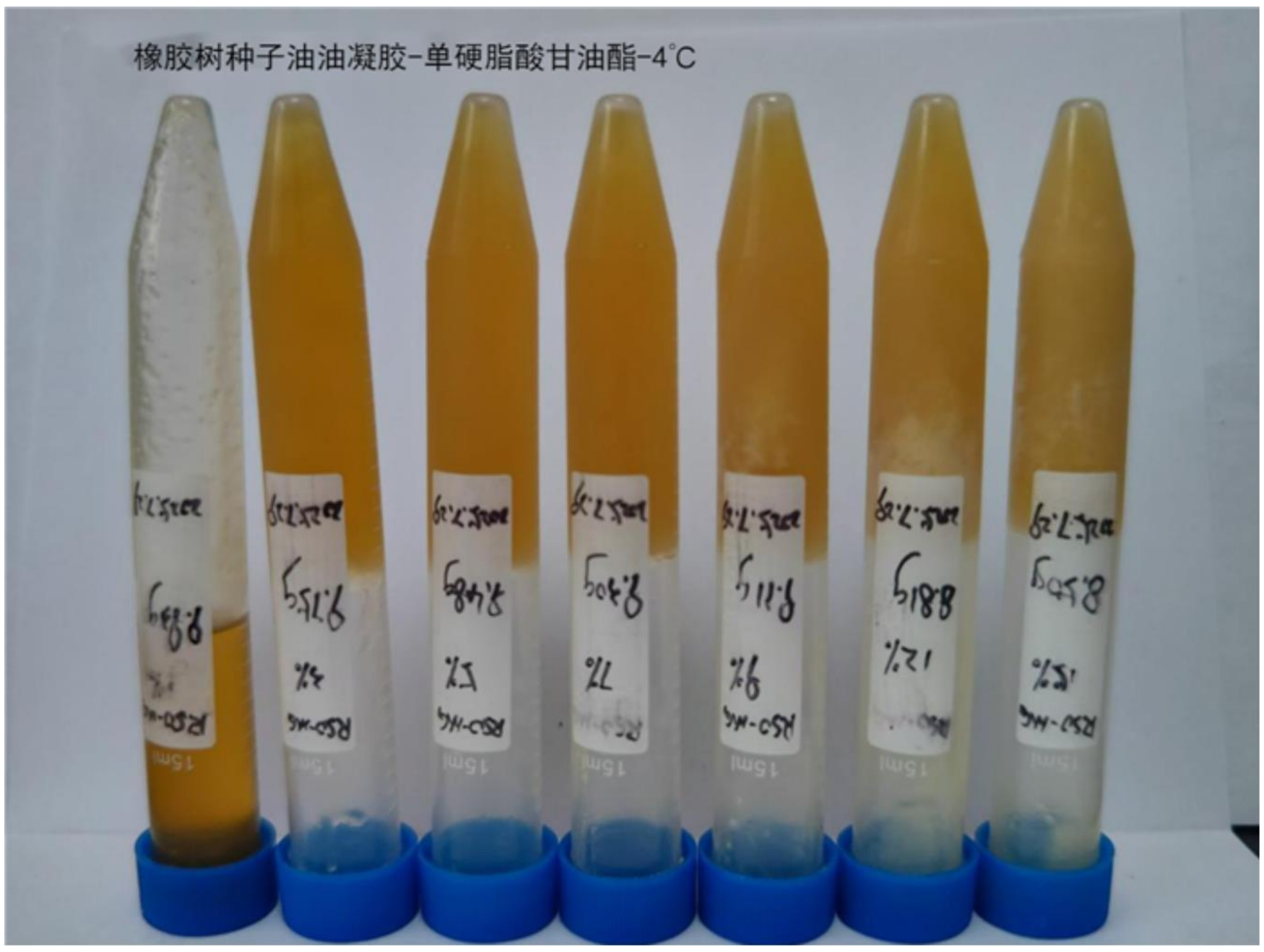

图1

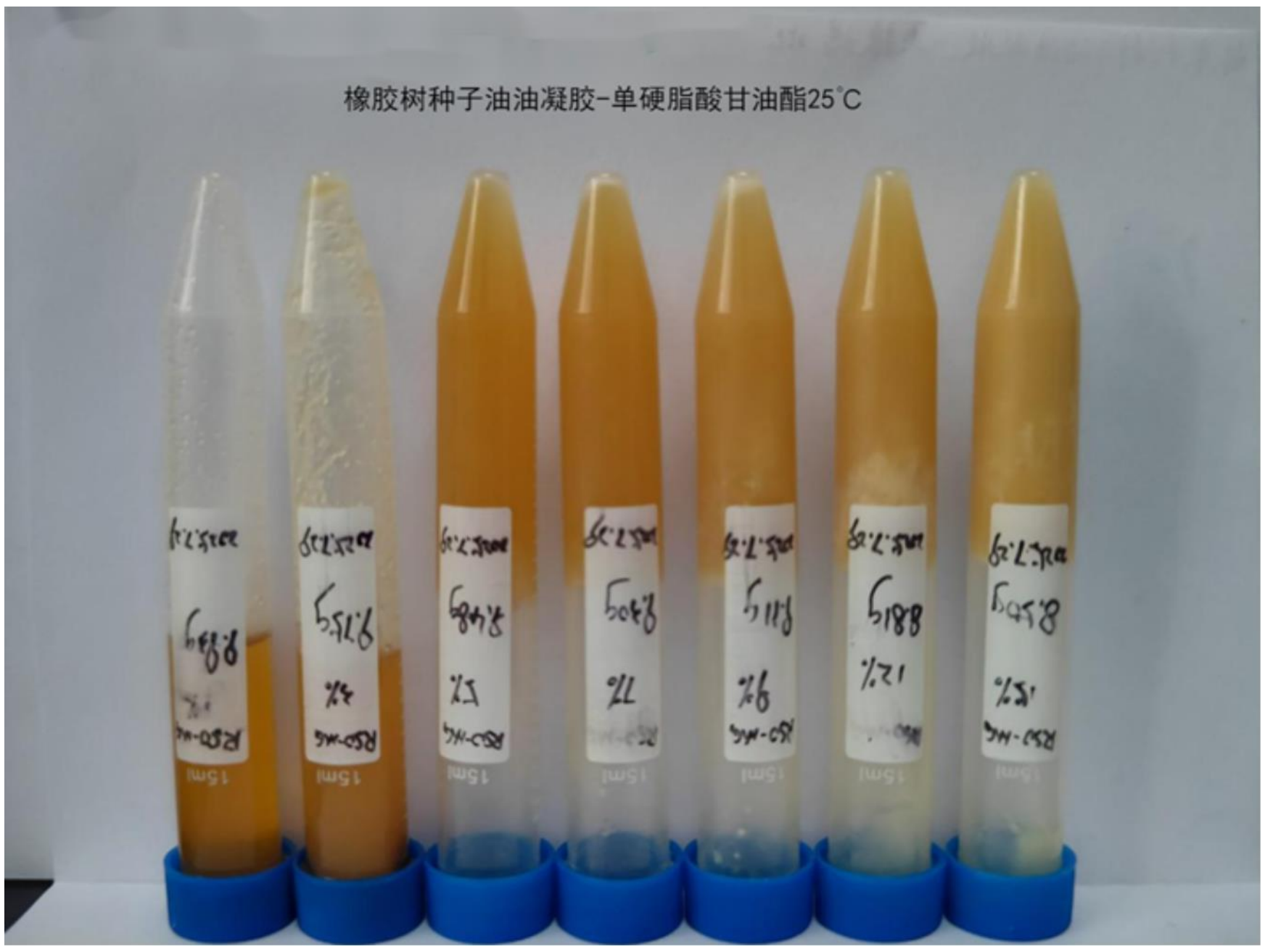

图2

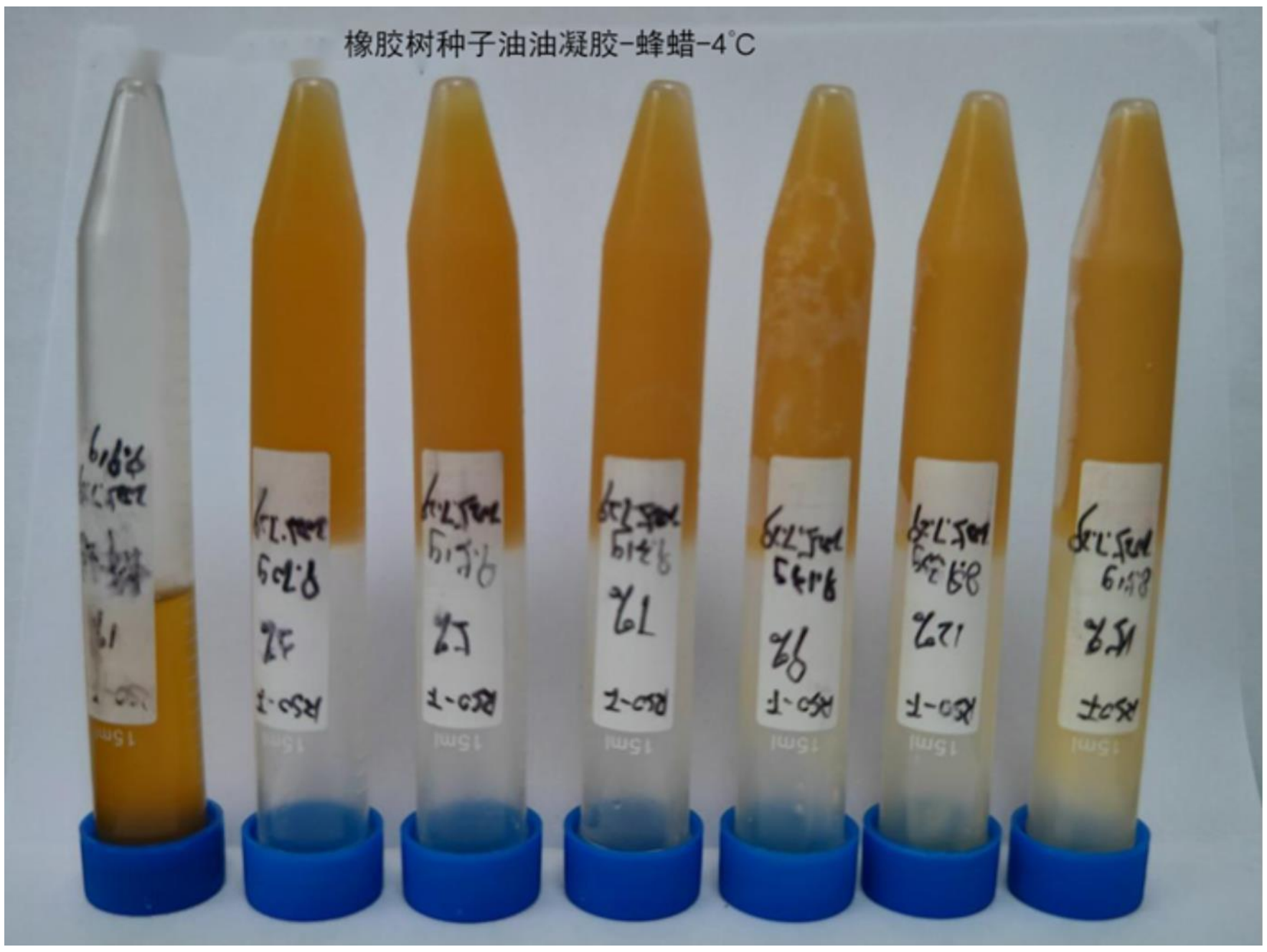

图3

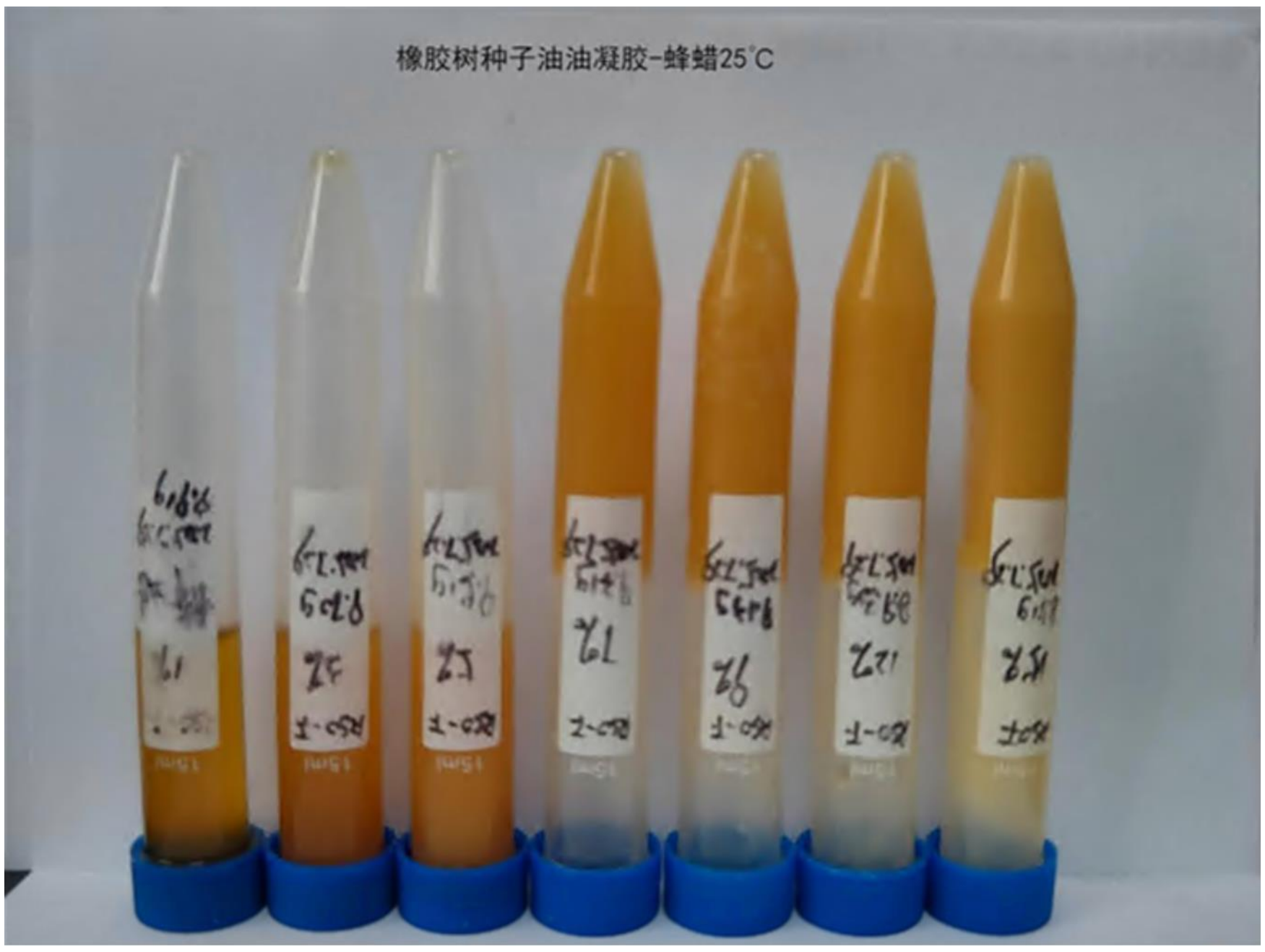

图4

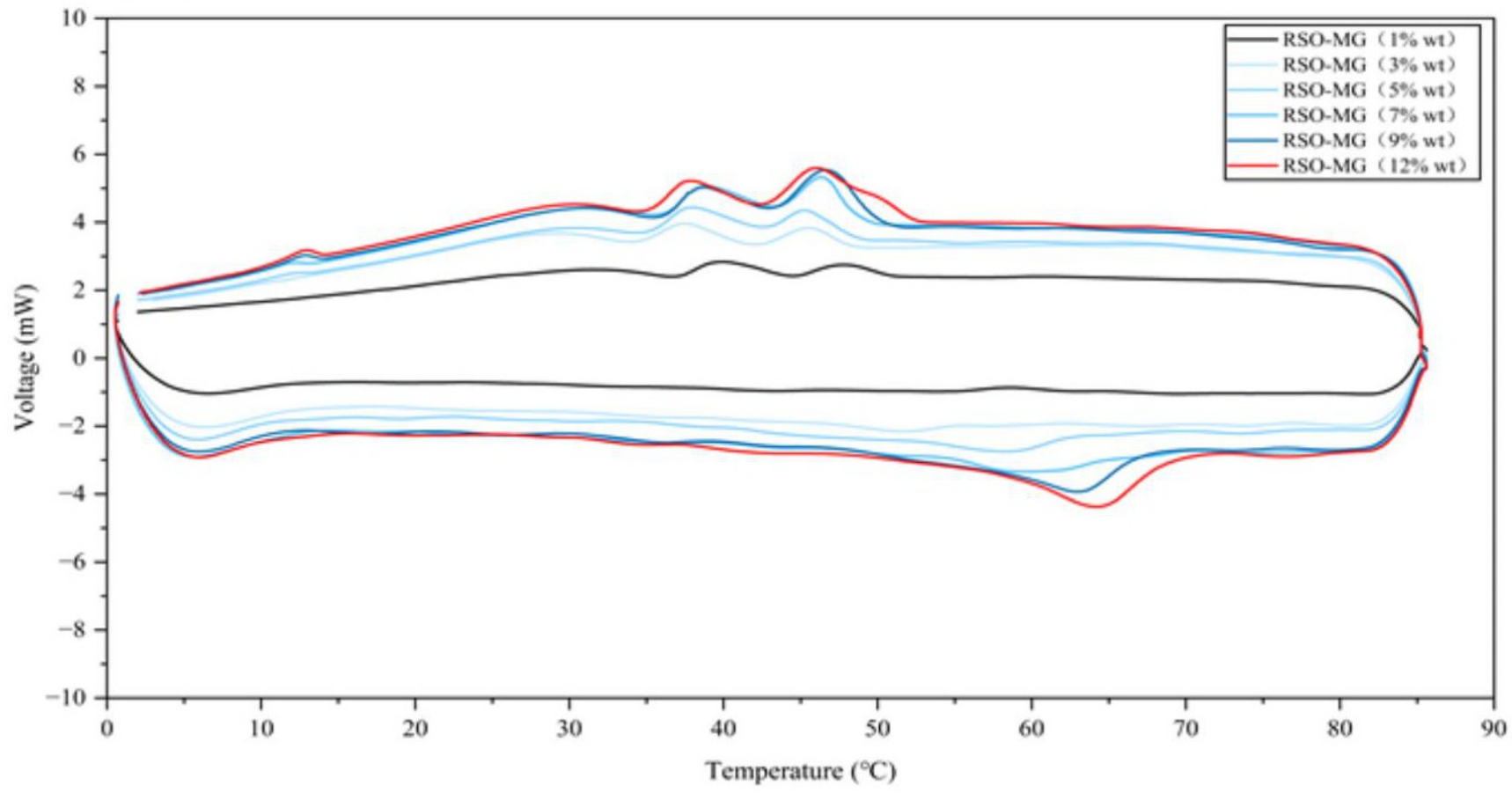
RSO-MG（1% wt）
RSO-MG（3% wt）
RSO-MG（5% wt）
RSO-MG（7% wt）
RSO-MG（9% wt）
RSO-MG（12% wt）
Voltage (mW)
Temperature (°C)

图5

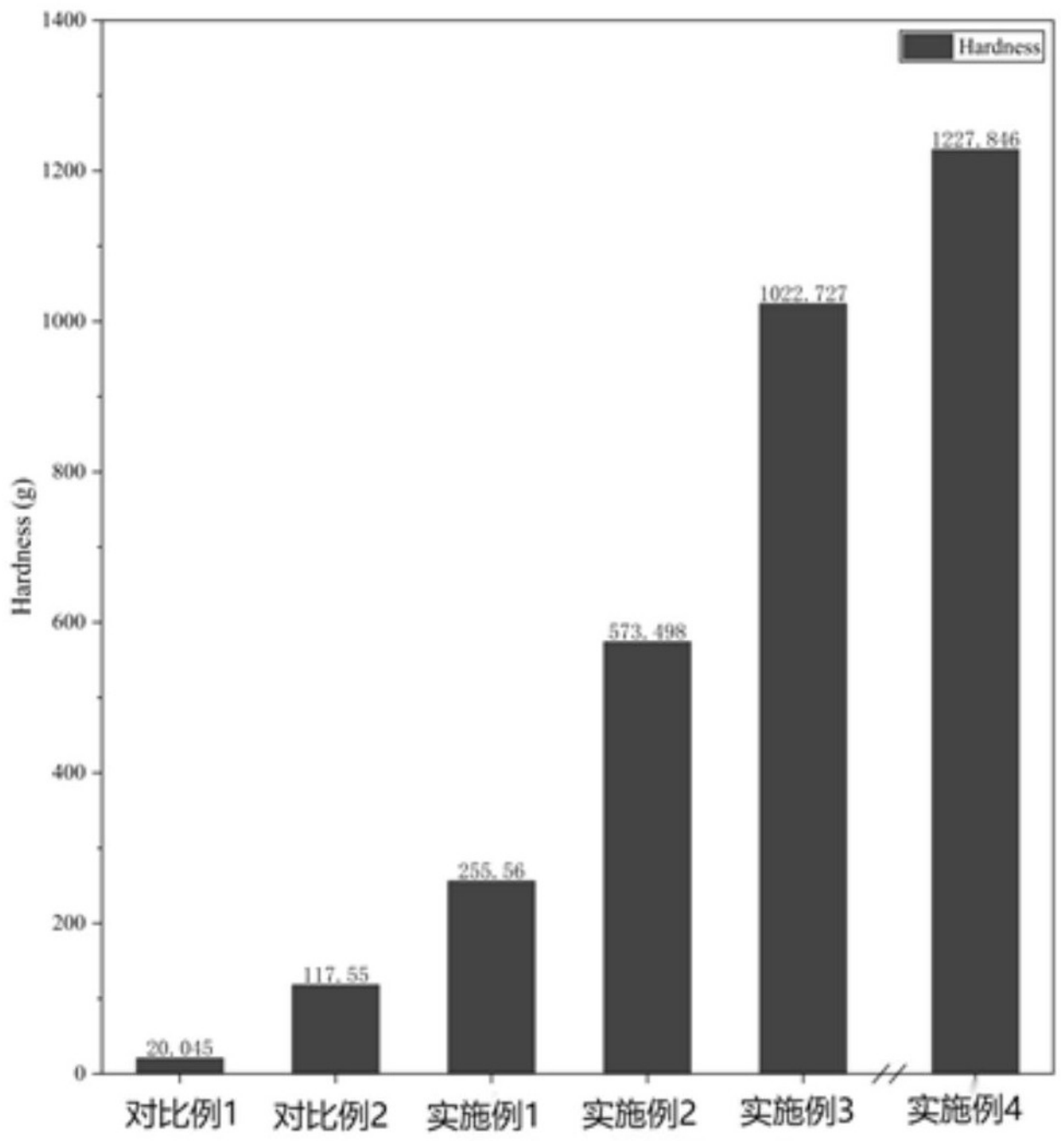
Hardness
Hardness (g)
20.045
117.55
255.56
573.498
1022.727
1227.846
对比例1
对比例2
实施例1
实施例2
实施例3
实施例4

图6

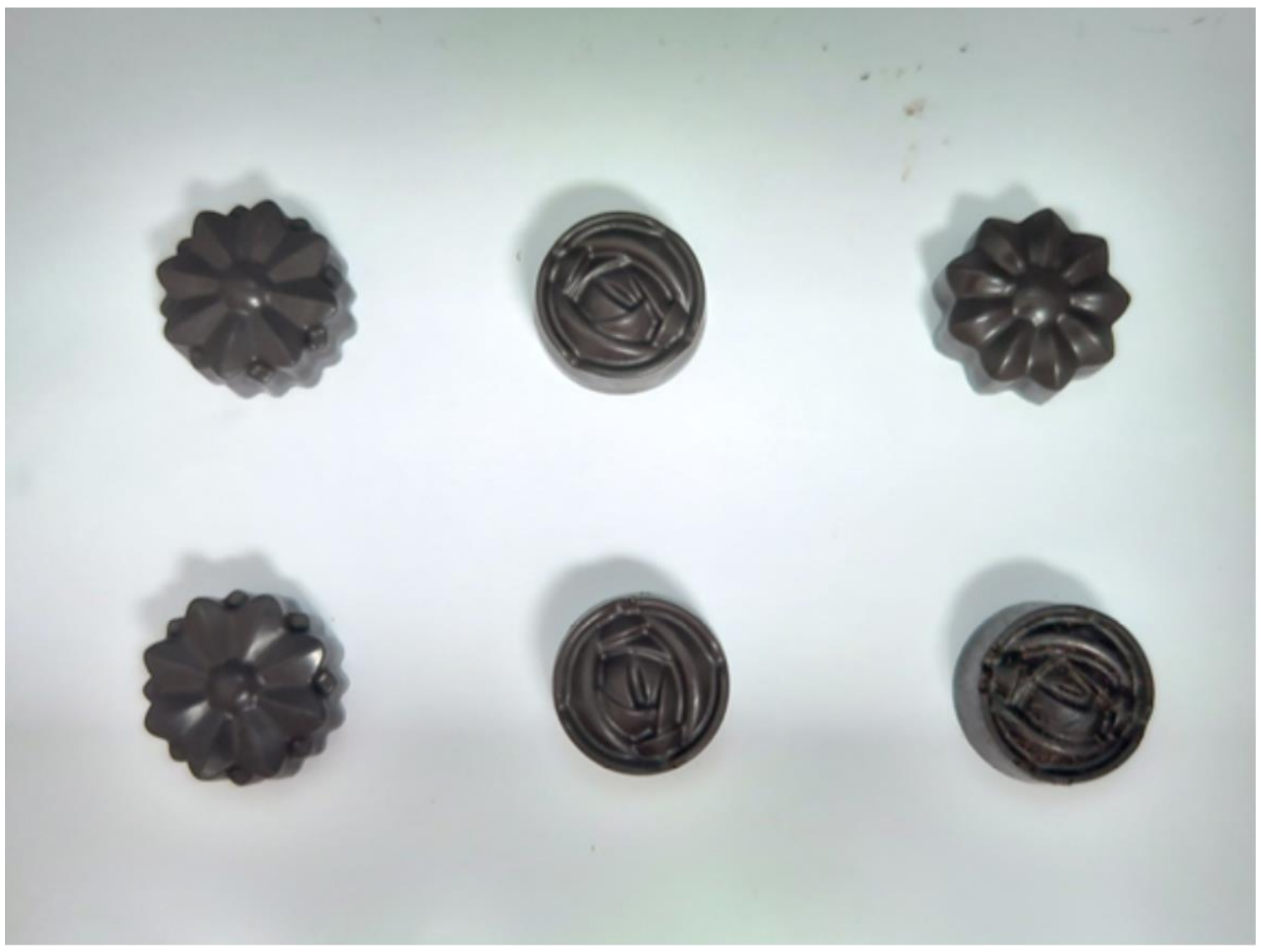

图7

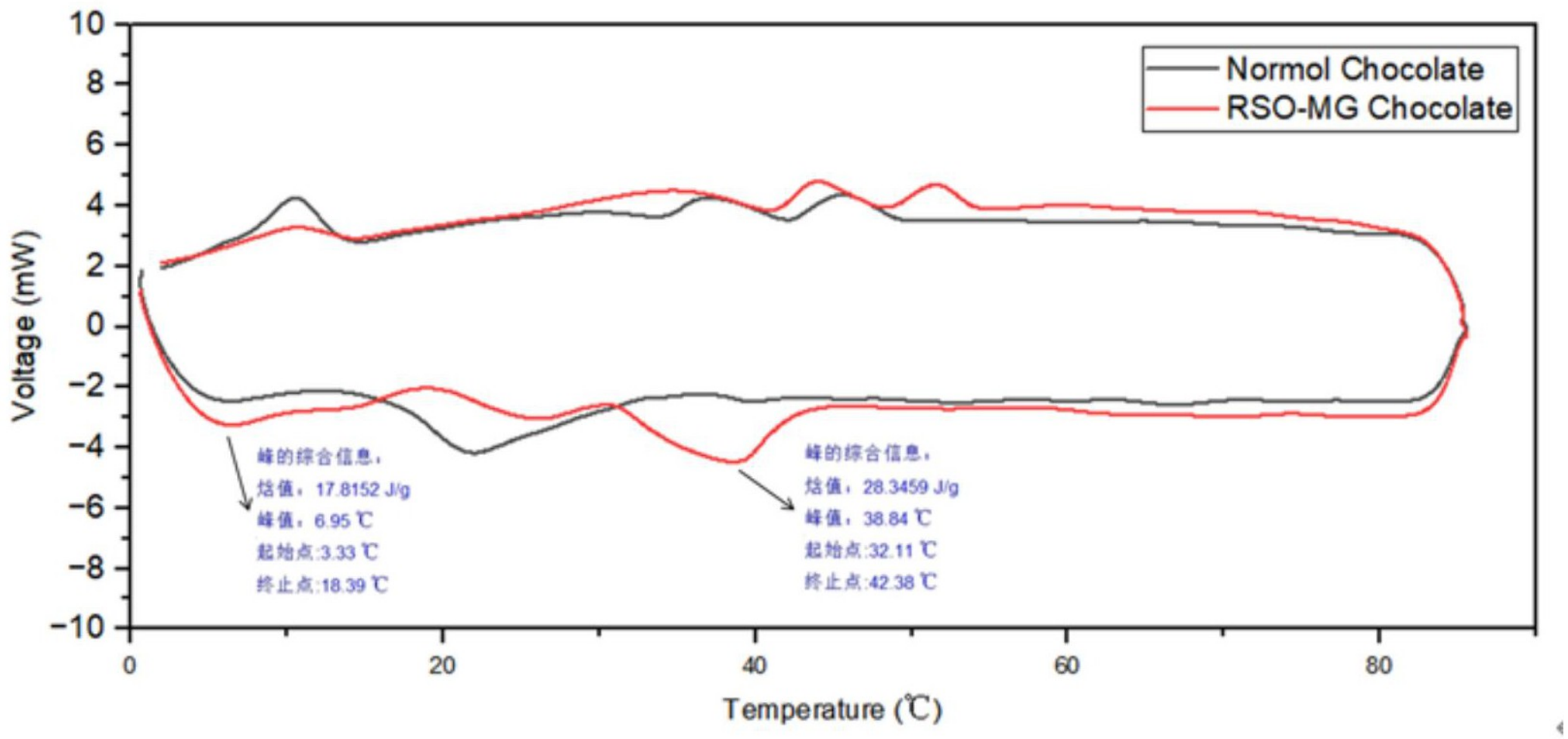
Normol Chocolate
RSO-MG Chocolate
Voltage (mW)
Temperature (℃)
10
8
6
4
2
0
-2
-4
-6
-8
-10
0
20
40
60
80
峰的综合信息，
焓值，17.8152 J/g
峰值，6.95 ℃
起始点:3.33 ℃
终止点:18.39 ℃
峰的综合信息，
焓值，28.3459 J/g
峰值，38.84 ℃
起始点:32.11 ℃
终止点:42.38 ℃

图8
